# A set of multi-entry identification keys to African frugivorous flies (Diptera, Tephritidae)

**DOI:** 10.3897/zookeys.428.7366

**Published:** 2014-07-24

**Authors:** Massimiliano Virgilio, Ian White, Marc De Meyer

**Affiliations:** 1 Royal Museum for Central Africa, Leuvensesteenweg 13, B3080 Tervuren, Belgium; 2 Buxton, UK; previously The Natural History Museum, Cromwell Road, London, SW7 5BD

**Keywords:** Tephritidae, Africa, identification key

## Introduction

Tephritid fruit flies, or "true" fruit flies (Diptera, Tephritidae) include approximately 500 genera and 4800 valid species (Norrbom 2004), whose vast majority (95%) is represented by phytophagous species (reviewed in Aluja and Norrbom 1999). Among them, frugivorous flies represent approximately 25–30% of all tephritid species, occur in tropical and temperate regions of all continents except the Antarctic and are predominantly distributed in five main genera (*Anastrepha* Schiner, *Rhagoletis* Loew, *Ceratitis* MacLeay, *Dacus* Fabricius and *Bactrocera* Macquart). Frugivorous tephritids attack healthy fruit still on the tree. The larvae develop inside the fruit, feed on the plant tissues, and complete their developmental cycle in the soil. A relatively limited number (approximately 100) of frugivorous species are phytophagous pests whose larvae attack pulp and/or seeds of cultivated fruits and crops of agricultural importance. In Africa, damage on commercial fruits and crops is caused mainly by polyphagous species belonging to the genera *Ceratitis*, *Dacus* and *Bactrocera* (De Meyer et al. 2008; White 2006). Other African and closely related genera with fewer taxa are *Capparimyia* Bezzi, *Carpophthoromyia* Austen, *Neoceratitis* Hendel, *Perilampsis* Bezzi and *Trirhithrum* Bezzi, which also include some species of economic significance.

Currently, identification of tephritid flies is a specialized task largely performed by a restricted pool of experienced taxonomists, a group that is constantly becoming smaller due to the well-known problems related to the general loss of taxonomical expertise on insects as well as on most taxonomic groups (Carvalho et al. 2007; Wilson 2000). In the last few decades, globalisation of fruit trade and transport (Aluja and Mangan 2008; Malacrida et al. 2007) has made the need for swift, reliable and accurate identification methods for frugivorous flies even more urgent. For example, in 1995, the erroneous identification of *Bactrocera zonata* as *Bactrocera pallidus* in Egypt produced a three-year delay on implementation of phytosanitary measures and resulted in serious damage to the agricultural productivity of the whole Alexandria region.

The morphological identification of African tephritids largely depends on the use of classical single-entry (dichotomous) keys. These keys are available for most African genera (e.g., White 2006), with the important exception of the genus *Ceratitis*, whose species can only be identified through separate subgeneric keys (De Meyer 1996, 1998, 2000; De Meyer and Freidberg 2006). The main disadvantage of single-entry keys is that species identification inevitably fails whenever the user is not able to select any of the dichotomous character states listed in the key (e.g., due to his inadequate taxonomic expertise, lack of clarity of the key, damaged specimen, etc.). Additionally, the specific terminology used in published keys represents a serious obstacle for non-specialist users who are not particularly acquainted with insect morphology and taxonomy. For these reasons, obtaining the taxonomical expertise that is necessary to identify tephritids using the above mentioned tools has never been an easy task, particularly for African scientists who can only rely on a limited number of comprehensive reference collections in the continent (as, for example, the South African National Collection of Insects, Pretoria - South Africa, the National Museums of Kenya, Nairobi - Kenya, or the International Institute of Tropical Agriculture (IITA), Cotonou - Benin). Molecular techniques represent a partial solution to counteract loss of taxonomical expertise on tephritid flies. DNA barcoding has been proposed as a relatively rapid and effective tool for the identification of fruit flies (Armstrong and Ball 2005). Yet, despite the availability of relatively large reference libraries of DNA barcodes for tephritid fruit flies, this method is still not routinely used for identification mainly due to shortcomings such as the difficulty of resolving important species complexes (Smit et al. 2013; Virgilio et al. 2010) and the incompleteness of reference libraries (Virgilio et al. 2012).

To try and reduce the effects of some of the aforementioned issues, we developed a set of freely available multi-entry identification keys for African fruit flies. The keys provide a professional identification tool that is also accessible to non-specialised morphologists (*i.e.*, people that might be interested in fruit fly identification such as students, technicians, agronomists, quarantine officers, ecologists, farmers, molecular biologists, etc.). Matrixes containing scores for 340 characters from 400 African species belonging to the genera *Bactrocera*, *Capparimyia*, *Carpophthoromyia*, *Ceratitis*, *Dacus*, *Neoceratitis*, *Perilampsis* and *Trirhithrum* were compiled from data sets that were used within the framework of previous taxonomic revisions (De Meyer 1996, 1998, 2000, 2006, 2009; De Meyer and Freidberg 2005, 2006, 2012; White et al. 2003; White 2006; White and Goodger 2009). Scores were transferred into seven separate data sets, imported into LUCID 3.5 (www.lucidcentral.org) and used as the main data sources for the multi-entry identification keys. Species lists and morphological characters were then revised and optimised in order to include only (a) species with valid names under the International Code of Zoological Nomenclature and (b) characters including at least two character states in congeneric species. This generated 7 matrixes with a total of 68352 entries. Additionally, a "pre-key" for genus designation was built *ex novo* by selecting a set of 23 characters that were deemed to be informative for generic separation. A total of 390 taxa were included in seven identification keys for species identification within genus or genus group (*Bactrocera* + *Dacus*, *Capparimyia*, *Carpophthoromyia*, *Ceratitis*, *Neoceratitis*, *Perilampsis*, *Trirhithrum*). For each genus, species of economic importance were assigned to a separate subset (see below).

Different character sets were considered for each genus (range 11-90 characters and 22–204 character states). The complete lists of species, characters, character states and dependencies considered for each key are provided as supplementary files (SF1, SF2). Each character state was scored in LUCID as either "present and common" or "absent" (other options such as "present but rare", "common and misinterpreted" etc. were not implemented). The "not scoped" option was used to generate unfolding keys, *i.e.* keys with characters that are initially not shown but appear only when a pre-defined subset of species remains to be identified. We built unfolding keys whenever character scores were only available for subsets of a maximum of 5 congeneric taxa. Dependencies between characters were also generated. Positive dependencies were defined whenever a character was only meaningful in relation to a previously defined character state (*e.g.* in the *Ceratitis* key, the character "number of frontal setae" is positively dependent from the character state "frontal setae: yes"). Conversely, negative dependencies were generated to discard characters that were not meaningful after a previous character state was selected (*e.g.* in the *Ceratitis* key, the character "females, aculeus tip with small notch" is negatively dependent on the character state "sex: male"). To facilitate identification, characters were grouped into head, thorax, wings, legs and abdomen character sets. The character "sex" was always placed first, in order to reduce the character list by discarding all negative dependencies controlled by the character states "male" and "female".

We considered that the number of morphological characters used in the largest identification keys (*i.e.* keys to *Bactrocera*/*Dacus*, *Ceratitis*, *Trirhithrum*) might also represent an obstacle to non-specialists. Hence, we arbitrarily defined three subsets of characters for these keys including (1) only characters of very straightforward use (included in the subset "step1: use only the most straightforward characters to get a short list of candidate species"), (2) all characters except the ones of most difficult use (subset "step 2: try identification by excluding only the most difficult characters") and (3) all characters, including "easy", "average", and "difficult" ones (subset "step 3: use also difficult characters if step 2 does not bring to species identification"). The user has the possibility of following a three steps identification procedure that considers characters of straightforward use at first, followed by characters of more and more difficult interpretation. This procedure should facilitate identification and reduce the risk of misidentification (particularly if a species can be identified only through step 1 or through step 1 and 2). We also defined a subset for species of economical importance. The use of this subset should speed up the identification of the more commonly trapped / intercepted taxa. When using this subset, identification should be carefully verified *a posteriori* (through the hyperlink to species description, see below) as all the less common species not included in this subset might be erroneously identified as species of economical importance (false positives). Of course, character and species subsets can all be ignored and the user can either arbitrarily score any of the characters available from the full list or use the "best" option provided by the LUCID software which should allow choosing characters with the highest discrimination power (the "best" option can be repeatedly used after eliminating redundant characters through the "prune" option). In any case, being a multi-entry key, the user can always decide either to skip characters, to choose multiple answers whenever he is uncertain about the correct score and/or to restrict the identification only to the most common species.

We tried to make the technical terminology used in the single-entry keys more accessible to non-specialists by adopting a consistent framework of character names and indicating in parentheses alternative names of the same character in the published scientific literature (as it happens for example with the *Ceratitis* subapical / cubital / preapical wing band). We then embedded images that clearly illustrate name and position of each character on the insect body as well as images showing how the same character state looks in different species. An initial set of 2300 images was assembled from the databases of the Royal Museum for Central Africa (RMCA) and of the London Natural History Museum (NHM). Images were grouped according to species name and body part (head, thorax dorsal, thorax lateral, abdomen, wings, legs), divided in groups and, when possible, assigned to each combination of character state and species name. This generated a database of approximately 28000 repeated images (for example, the same thorax image of a particular species was repeatedly used to illustrate postpronotal lobe, scutum and scutellum characters for that species). The large set of embedded images aims at clearly illustrating the morphological variability of the same character state across species. In fact, we consider that many terms used to describe morphological variation (such as "small / large, darker / paler, thicker / thinner etc.") while being straightforward for a tephritid taxonomist (who can rely on the experience accumulated after the examination of large numbers of specimens) are not always clear to non-specialised users. Therefore, we dedicated particular attention to provide multiple images to show, for example, how "narrow" a wing discal band should be, before being considered as "broad" or how "small" a postpronotal spot can be before being scored as "occupying most of postpronotum".

Once a tentative identification is obtained (or when the list of candidate species is reduced to a few taxa), the keys give the possibility of verifying the correspondence between the examined voucher and (1) the species description as it appears in the published scientific literature and (2) images from the RMCA and NHM tephritid collections. Discrepancies between the examined voucher and available images (as it might result from the occurrence of multiple character states for a species) can then be verified through hyperlinks to either the species description or to all character states considered for that species in the LUCID input matrix. Information regarding the taxonomic status, geographic distribution and collection specimens of each taxon is also available through hyperlinks to Encyclopedia of Life (EOL) and to the Belgian Biodiversity Platform (BeBIF, a section of GBIF, the Global Biodiversity Information Facility). Links to the Barcoding of Life Database website (BOLD) allow verifying the availability and geographical coverage of DNA barcodes for each species. In some cases, the available character list will not always allow the unambiguous identification of a taxon (as it happens, for example, with females of the subgenus *Ceratitis (Pterandrus)*). Under these circumstances, the direct comparison of species descriptions and distributions is the best strategy to try and resolve the short list of candidate taxa.

The keys can be accessed online (http://keys.lucidcentral.org/keys/v3/fruitflies/) or freely downloaded and used from a computer hard drive (supplementary files SF3-10). The first option is only recommended for a preliminary overview of the key structure, while downloading and running the keys (e.g. from a memory stick used as a removable device) should allow a faster and more effective use of the software. A quick start guide providing basic information about the key functioning is associated to the downloadable version.

## Supplementary material

Supplementary material 1 List of species, characters and character states considered in each identification key doi: 10.3897/zookeys.428.7366.app1 Data type: multimedia This dataset is made available under the Open Database License (http://opendatacommons.org/licenses/odbl/1.0/). The Open Database License (ODbL) is a license agreement intended to allow users to freely share, modify, and use this Dataset while maintaining this same freedom for others, provided that the original source and author(s) are credited.Massimiliano Virgilio, Ian White, Marc de Meyer

Supplementary material 2 List of positive and negative character dependencies in each identification key doi: 10.3897/zookeys.428.7366.app2 Data type: multimedia This dataset is made available under the Open Database License (http://opendatacommons.org/licenses/odbl/1.0/). The Open Database License (ODbL) is a license agreement intended to allow users to freely share, modify, and use this Dataset while maintaining this same freedom for others, provided that the original source and author(s) are credited.Massimiliano Virgilio, Ian White, Marc de Meyer

Supplementary material 3 Key to genera doi: 10.3897/zookeys.428.7366.app3 Data type: multimedia Explanation note: A set of multi-entry identification keys to African frugivorous flies (Diptera, Tephritidae): key to genera. This dataset is made available under the Open Database License (http://opendatacommons.org/licenses/odbl/1.0/). The Open Database License (ODbL) is a license agreement intended to allow users to freely share, modify, and use this Dataset while maintaining this same freedom for others, provided that the original source and author(s) are credited.Massimiliano Virgilio, Ian White, Marc de Meyer

Supplementary material 4 Key to *Capparimyia* doi: 10.3897/zookeys.428.7366.app4 Data type: multimedia Explanation note: A set of multi-entry identification keys to African frugivorous flies (Diptera, Tephritidae): key to *Capparimyia.* This dataset is made available under the Open Database License (http://opendatacommons.org/licenses/odbl/1.0/). The Open Database License (ODbL) is a license agreement intended to allow users to freely share, modify, and use this Dataset while maintaining this same freedom for others, provided that the original source and author(s) are credited.Massimiliano Virgilio, Ian White, Marc de Meyer

Supplementary material 5 Key to *Carpophthoromyia* doi: 10.3897/zookeys.428.7366.app5 Data type: multimedia Explanation note: A set of multi-entry identification keys to African frugivorous flies (Diptera, Tephritidae): key to *Carpophthoromyia*. This dataset is made available under the Open Database License (http://opendatacommons.org/licenses/odbl/1.0/). The Open Database License (ODbL) is a license agreement intended to allow users to freely share, modify, and use this Dataset while maintaining this same freedom for others, provided that the original source and author(s) are credited.Massimiliano Virgilio, Ian White, Marc de Meyer

Supplementary material 6 Key to *Ceratitis* doi: 10.3897/zookeys.428.7366.app6 Data type: multimedia Explanation note: A set of multi-entry identification keys to African frugivorous flies (Diptera, Tephritidae): key to *Ceratitis.* This dataset is made available under the Open Database License (http://opendatacommons.org/licenses/odbl/1.0/). The Open Database License (ODbL) is a license agreement intended to allow users to freely share, modify, and use this Dataset while maintaining this same freedom for others, provided that the original source and author(s) are credited.Massimiliano Virgilio, Ian White, Marc de Meyer

Supplementary material 7 Key to *Dacus* and *Bactrocera* doi: 10.3897/zookeys.428.7366.app7 Data type: multimedia Explanation note: A set of multi-entry identification keys to African frugivorous flies (Diptera, Tephritidae): key to *Dacus* and *Bactrocera*. This dataset is made available under the Open Database License (http://opendatacommons.org/licenses/odbl/1.0/). The Open Database License (ODbL) is a license agreement intended to allow users to freely share, modify, and use this Dataset while maintaining this same freedom for others, provided that the original source and author(s) are credited.Massimiliano Virgilio, Ian White, Marc de Meyer

Supplementary material 8 Key to *Neoceratitis* doi: 10.3897/zookeys.428.7366.app8 Data type: multimedia Explanation note: A set of multi-entry identification keys to African frugivorous flies (Diptera, Tephritidae): key to *Dacus* and *Neoceratitis*. This dataset is made available under the Open Database License (http://opendatacommons.org/licenses/odbl/1.0/). The Open Database License (ODbL) is a license agreement intended to allow users to freely share, modify, and use this Dataset while maintaining this same freedom for others, provided that the original source and author(s) are credited.Massimiliano Virgilio, Ian White, Marc de Meyer

Supplementary material 9 Key to *Perilampsis* doi: 10.3897/zookeys.428.7366.app9 Data type: multimedia Explanation note: A set of multi-entry identification keys to African frugivorous flies (Diptera, Tephritidae): key to *Dacus* and *Perilampsis*. This dataset is made available under the Open Database License (http://opendatacommons.org/licenses/odbl/1.0/). The Open Database License (ODbL) is a license agreement intended to allow users to freely share, modify, and use this Dataset while maintaining this same freedom for others, provided that the original source and author(s) are credited.Massimiliano Virgilio, Ian White, Marc de Meyer

Supplementary material 10 Key to *Trirhithrum* doi: 10.3897/zookeys.428.7366.app10 Data type: multimedia Explanation note: A set of multi-entry identification keys to African frugivorous flies (Diptera, Tephritidae): key to *Dacus* and *Trirhithrum* This dataset is made available under the Open Database License (http://opendatacommons.org/licenses/odbl/1.0/). The Open Database License (ODbL) is a license agreement intended to allow users to freely share, modify, and use this Dataset while maintaining this same freedom for others, provided that the original source and author(s) are credited.Massimiliano Virgilio, Ian White, Marc de Meyer
