# Supplementary material for: A set of multi-entry identification keys to African frugivorous flies (Diptera, Tephritidae)
Source: Zookeys. 2014 Jul 24;(428):97–108. doi: 10.3897/zookeys.428.7366 (PMC4143993; doi:10.3897/zookeys.428.7366)
Supplement: Supplementary material 4 — Key to Capparimyia [file zookeys-428-097-s004.zip › SF4_ZooKeys_key to Capparimyia/key/SF4_ZooKeys_key to Capparimyia/Media/Html/Capparimyia aenigma.htm]

Capparimyia aenigma sp


***Capparimyia aenigma*** **De Meyer &
Freidberg**

 

Body
length.
G 2.30-3.70
mm E 3.65-4.20
mm;
wing length: 2.45-3.40 mm.

Male

Head. First flagellomere obtuse apically. Arista
short pubescent, rays shorter than width of arista at base. Frontal setae much
thinner than, and subequal in length to, orbital seta, posterior frontal
sometimes less developed than anterior frontal; one short orbital;
ocellar seta 1- 1.5 times as long as ocellar triangle; postocellar seta black,
shorter than lateral vertical seta; eye/medial vertical seta ratio: 1.4-1.8.
Frons convex to flat; not or slightly protuberant. Genal seta and genal setulae
yellow, although setulae sometimes blackish.

Thorax. Scutum
largely shining black, microtrichose area restricted. Black postpronotal spot
confluent with black lateral presutural spot;
latter reaching white presutural band; black scapular spot present and
confluent anteriorly with black lateral presutural spot (in one specimen from
Saudi Arabia scapular spot missing); black acrostichal spot reaching base of
dorsocentral seta, and confluent with black sutural spot. Black presutural
supra-alar spot confluent with black lateral presutural spot; black postsutural
supra-alar and black intra-alar spots confluent. White postsutural vitta
extending posteriorly to base of postsutural supra-alar seta (in Namibian specimens to base of intra-alar seta); white medial
vitta extending anteriorly to transverse suture. Suture between scutum and
scutellum usually completely white. Black apical scutellar spots largely
confluent, with shallow indentation basally. Subscutellum entirely black. Dorsocentral seta aligned anterior to
postsutural supra-alar seta. Anepisternal and anepimeral setae white.�

Wing. Anterior
apical band with window along vein R2+3 uninterrupted; subapical
band occasionally surpassing anterior margin of cell dm; R-M ratio: 0.4-0.9; dm
ratio: 2.0-3.1.

Abdomen. Epandrium in lateral view with lateral surstylus shorter
than epandrium; posterior lobe of lateral surstylus reduced, not extending
posteriorly; medial surstylus directed more posteriorly than median part of
lateral surstylus, partly exposed, and with at least part of prensisetae
exposed.

 

Female

Anepisternal
seta sometimes black; subapical band surpassing anterior margin of cell dm. Tergal-oviscapal measure: 2-2.5. Aculeus apical part
slender, tapered evenly to apex.

 

(Description
after De Meyer & Freidberg, 2005)
