# Supplementary material for: A set of multi-entry identification keys to African frugivorous flies (Diptera, Tephritidae)
Source: Zookeys. 2014 Jul 24;(428):97–108. doi: 10.3897/zookeys.428.7366 (PMC4143993; doi:10.3897/zookeys.428.7366)
Supplement: Supplementary material 4 — Key to Capparimyia [file zookeys-428-097-s004.zip › SF4_ZooKeys_key to Capparimyia/key/SF4_ZooKeys_key to Capparimyia/Media/Html/Capparimyia aristata.htm]

Capparimyia aristata nom


***Capparimyia aristata*** **De Meyer &
Freidberg**

*Pardalaspis bipustulata* Bezzi, 1924b: 104;
preoccupied Bezzi, 1923.

 

Bodylength.
G 3.70-4.00
mm E 4.45 mm; wing
length: 3.40-3.85 mm.

Male

Head. First
flagellomere obtuse apically. Arista with rays twice as long as width of arista
at base. Frontal setae equal to, or longer than, posterior orbital seta,
anterior frontal seta less well developed than posterior; two orbital setae;
ocellar seta black and thin, shorter than ocellar triangle; postocellar seta
whitish yellow; subequal in length to lateral vertical seta; eye/medial
vertical seta ratio: 1.2-1.3. Frons convex; not protuberant. Genal setulae and
genal seta whitish yellow.

Thorax. Scutum
largely microtrichose; black spots reduced. Black postpronotal spot restricted
to base of postpronotal seta, not confluent with black lateral presutural spot;
latter spot anteriorly extending obliquely between lateral and medial scapular
setae, posteriorly more restricted medially, not reaching white presutural
spot; black scapular spot absent; black sutural spot present; black acrostichal
spot not reaching base of dorsocentral seta; black presutural supra-alar spot
separate from black lateral presutural spot; black postsutural supra-alar and
black intra-alar spots separate. White postsutural vitta joining white
prescutellar band; white medial vitta extending anteriorly beyond transverse
suture, almost to base of medial scapular seta. Black apical scutellar spots
widely separated, not reaching base of scutellum. Subscutellum mostly black,
with white or yellow median spot. Dorsocentral seta aligned posterior to or at
level with postsutural supra-alar seta. Anepisternal and anepimeral setae
yellow or black.

Wing. Anterior
apical band with window along vein R2+3 interrupted; subapical band
always surpassing anterior margin of cell dm; R-M ratio: 0.7-0.8; dm ratio:
2.6-2.9.

Abdomen. Male
terminalia in lateral view with lateral surstylus short, shorter than
epandrium; posterior lobe of lateral surstylus reduced, not extending posteriorly;
medial surstylus directed more anteriorly than median part of lateral
surstylus, completely hidden behind lateral surstylus.

 

Female

Tergal-oviscapal
measure: 2. Aculeus with apical part relatively broad, evenly tapered to apex;
tip with preapical step.

 

(Description
after De Meyer & Freidberg, 2005)
