# Supplementary material for: A set of multi-entry identification keys to African frugivorous flies (Diptera, Tephritidae)
Source: Zookeys. 2014 Jul 24;(428):97–108. doi: 10.3897/zookeys.428.7366 (PMC4143993; doi:10.3897/zookeys.428.7366)
Supplement: Supplementary material 4 — Key to Capparimyia [file zookeys-428-097-s004.zip › SF4_ZooKeys_key to Capparimyia/key/SF4_ZooKeys_key to Capparimyia/Media/Html/Capparimyia bipustulata.htm]

Capparimyia bipustulata (Bezzi) (Figs xx-xx)


***Capparimyia bipustulata*** **(Bezzi)**

*Pardalaspis
bipustulata* Bezzi, 1923:
528 

 

Body
length.
G 3.00-4.15
mm E 3.60-4.85
mm;
wing length: 3.30-4.25 mm.

Male

Head. First
flagellomere obtuse apically. Arista short pubescent, rays shorter than width of arista at base. Frontal setae equal to, or longer
than, posterior orbital seta, anterior frontal seta sometimes less well
developed than posterior seta; two orbital setae; ocellar seta present
or absent, if present then black and thin, varying in length from shorter than
to slightly longer than ocellar triangle; postocellar seta yellowish, subequal
in length to lateral vertical seta; eye/medial vertical seta ratio: 1.2-1.5.
Frons convex to flat; not or slightly protuberant. Genal setulae black; genal
seta yellowish.

Thorax. Scutum
largely microtrichose; black spots reduced. Black postpronotal spot usually
confluent with black lateral presutural spot, sometimes narrowly separated or
restricted to base of postpronotal seta; black lateral presutural spot not
extending to white presutural spot, sometimes restricted to anterior margin
adjacent to postpronotum; black scapular spot absent; black sutural spot
present, sometimes only as small spot; black acrostichal spot not or barely
extending to base of dorsocentral seta. Black acrostichal spot, base of
dorsocentral seta and black sutural spot occasionally linked by indistinct brownish line. Black presutural
supra-alar spot separate from black lateral presutural spot; black postsutural
supra-alar and black intra-alar spots separate. White postsutural vitta joining
white prescutellar band; white medial vitta extending anteriorly beyond
transverse suture to, or almost to, base of medial scapular
seta. Black apical scutellar spots
usually widely separated, rarely narrowly separated. Subscutellum mostly black,
with white median spot. Dorsocentral seta aligned posterior to or at level with
postsutural supra-alar seta. Anepisternal and anepimeral setae black, sometimes
anepimeral whitish.

Wing. Anterior
apical band with window along vein R2+3 interrupted; subapical band,
globally surpassing anterior margin of cell dm; R-M ratio: 0.6-0.8; dm ratio:
2.6-3.0.

Abdomen. Epandrium in lateral view with lateral surstylus short,
shorter than epandrium; posterior lobe of lateral surstylus well developed,
extending posteriorly; medial surstylus directed
more anteriorly than median part of lateral surstylus, completely hidden behind
lateral surstylus.

    

Female

Tergal-oviscapal measure: 2.5-3. Aculeus with apical
part relatively broad, evenly tapered to apex.

 

(Description
after De Meyer & Freidberg, 2005)
