# Supplementary material for: A set of multi-entry identification keys to African frugivorous flies (Diptera, Tephritidae)
Source: Zookeys. 2014 Jul 24;(428):97–108. doi: 10.3897/zookeys.428.7366 (PMC4143993; doi:10.3897/zookeys.428.7366)
Supplement: Supplementary material 4 — Key to Capparimyia [file zookeys-428-097-s004.zip › SF4_ZooKeys_key to Capparimyia/key/SF4_ZooKeys_key to Capparimyia/Media/Html/Capparimyia maeruae.htm]

Capparimyia maeruae sp


***Capparimyia maeruae*** **De Meyer &
Freidberg**

Body
length.
G 2.15-2.85
mm E 2.65-3.40
mm;
wing length: 2.15-3.80 mm.

Male

Head. First
flagellomere obtuse apically. Arista short pubescent, rays about as long as
width of arista at base. Frontal setae equal to posterior orbital seta,
sometimes slightly longer; rarely anterior frontal seta much thinner and
shorter than posterior frontal seta; two, rarely one, orbital setae present;
ocellar seta black and thin, about 1.5-2 times as
long as ocellar triangle; postocellar seta whitish yellow; subequal in
length to lateral vertical seta; eye/medial vertical seta ratio: 1.5-1.6. Frons
convex, not protuberant. Genal setulae reddish
brown, genal seta whitish yellow.

Thorax. Scutum
largely microtrichose; black spots reduced. Black postpronotal spot confluent
with black lateral presutural spot or narrowly separated by brownish patch;
black lateral presutural spot extending to white presutural band, sometimes
narrowly so; black scapular spot absent; black sutural spot present, sometimes
only as brownish patch; black acrostichal spot not or barely reaching base of dorsocentral seta, confluent with
black spot or brown patch at mesal end of transverse suture. Black presutural
supra-alar spot confluent with black lateral presutural spot; black postsutural
supra-alar and black intra-alar spots confluent. White postsutural vitta
extending to posterior margin of mesonotum and confluent with white
prescutellar band; white medial vitta extending anteriorly to transverse
suture. Black apical scutellar spots largely separated, confluent with each other at apex, basally extending to base of scutellum by brownish patch.
Subscutellum entirely black. Dorsocentral seta aligned posterior to or
at level with postsutural supra-alar seta. Anepisternal and anepimeral setae
reddish or whitish yellow.

Wing. Anterior
apical band with window along vein R2+3 uninterrupted, distal
portion of band not well demarcated; subapical band always surpassing anterior
margin of cell dm; R-M ratio: 0.8-0.9; dm ratio: 3.0-3.3.

Abdomen.
Epandrium in lateral view with lateral surstylus
short and very broad, shorter than and as broad as epandrium; posterior lobe of
lateral surstylus reduced, not extending posteriorly; medial surstylus directed
more posteriorly than median part of lateral surstylus, with at least part of
prensisetae visible.

 

Female

Distal
portion of anterior apical band well demarcated. Oviscape completely covered by
short, dense setulae and with longer setae apically; setae about three times as
long as setulae. Tergal-oviscapal measure: 3. Aculeus
relatively broad nearly to apex, abruptly tapered to short apex with
preapical step.

 

(Description
after De Meyer & Freidberg, 2005)
