# Supplementary material for: A set of multi-entry identification keys to African frugivorous flies (Diptera, Tephritidae)
Source: Zookeys. 2014 Jul 24;(428):97–108. doi: 10.3897/zookeys.428.7366 (PMC4143993; doi:10.3897/zookeys.428.7366)
Supplement: Supplementary material 4 — Key to Capparimyia [file zookeys-428-097-s004.zip › SF4_ZooKeys_key to Capparimyia/key/SF4_ZooKeys_key to Capparimyia/Media/Html/Capparimyia melanaspis.htm]

Capparimyia melanaspis (Bezzi) (Figs xx-xx)


***Capparimyia melanaspis*** **(Bezzi)**

*Pardalaspis melanaspis* Bezzi, 1920: 229

 

Body
length.
G 2.60-3.85
mm E 3.05-4.50
mm;
wing length: 3.10-3.90 mm.

Male

Head. First
flagellomere obtuse apically. Arista short pubescent, rays shorter than width
of arista at base. Frontal setae equal to posterior orbital seta, sometimes
slightly longer; two orbital setae (in material from
Malindi, Kenya
one orbital seta); ocellar seta black, 2-3 times as long as ocellar
triangle, sometimes asymmetrical; postocellar seta usually black, rarely whitish yellow; subequal in length to
lateral vertical seta; eye/medial vertical seta ratio: 1.3-1.5. Frons convex to
flat; not or slightly protuberant. Genal seta and genal setulae mostly black,
rarely seta yellow.

Thorax. Scutum
largely shining black, microtrichose area restricted. Black postpronotal spot
confluent with black lateral presutural spot;
latter reaching white presutural band; black scapular spot present and
confluent anteriorly with black lateral presutural spot; black acrostichal spot
reaching base of dorsocentral seta and confluent with black sutural spot. Black
presutural supra-alar spot confluent with black lateral presutural spot; black
postsutural supra-alar and black intra-alar spots confluent. White postsutural
vitta extending posteriorly to base of postsutural supra-alar seta (in material from Malindi, Kenya, usually to halfway between base of poststutural supra-alar seta and
base of intra-alar seta); white medial vitta extending anteriorly to or
just beyond transverse suture. Suture between scutellum and scutum usually
black medially. Black apical scutellar spots largely confluent, with shallow
indentation basally. Subscutellum entirely black. Dorsocentral seta aligned
anterior to postsutural supra-alar seta. Anepisternal seta black, anepimeral
seta whitish.

Wing. Anterior
apical band with window along vein R2+3 uninterrupted; subapical
band always surpassing anterior margin of cell dm; R-M ratio: 0.6-0.8; dm
ratio: 2.5-2.8.

Abdomen. Epandrium in lateral view with lateral surstylus longer
than epandrium; posterior lobe of lateral surstylus
reduced, not extending posteriorly; medial surstylus directed more posteriorly,
with at least part of prensisetae visible.

 

Female

Tergal-oviscapal measure: 2-2.5. Aculeus apical part slender,
tapered evenly to apex.

 

(Description
after De Meyer & Freidberg, 2005)
