# Supplementary material for: A set of multi-entry identification keys to African frugivorous flies (Diptera, Tephritidae)
Source: Zookeys. 2014 Jul 24;(428):97–108. doi: 10.3897/zookeys.428.7366 (PMC4143993; doi:10.3897/zookeys.428.7366)
Supplement: Supplementary material 4 — Key to Capparimyia [file zookeys-428-097-s004.zip › SF4_ZooKeys_key to Capparimyia/key/SF4_ZooKeys_key to Capparimyia/Media/Html/Capparimyia mirabilis.htm]

Capparimyia mirabilis sp


***Capparimyia mirabilis*** **De Meyer &
Freidberg**

 

Body
length.
G 2.20-2.45
mm E 2.50-2.75
mm;
wing length: 2.20-2.60 mm.

Male

Head. First flagellomere obtuse
apically. Arista short pubescent, rays shorter than width of arista at base.
Frontal setae equal to posterior orbital seta, sometimes slightly longer; two
orbital setae;
ocellar seta black, twice as long as ocellar triangle; postocellar seta whitish
yellow; about half as long as lateral vertical seta, sometimes subequal in
length; eye/medial vertical seta ratio: 1.2-1.4. Frons flat, slightly protuberant. Genal
setulae black, genal seta yellow.

Thorax. Scutum
largely shining black, microtrichose area restricted. Black postpronotal spot
confluent with black lateral presutural spot; latter reaching white sutural
band; black scapular spot confluent anteriorly with black lateral presutural
spot; black acrostichal spot reaching base of dorsocentral seta and confluent
with black sutural spot. Black presutural supra-alar spot confluent with black
lateral presutural spot; black postsutural supra-alar and black intra-alar
spots confluent. White postsutural vitta extending posteriorly. Black apical
scutellar spots confluent, but with large white median
indentation; spots reaching base of scutellum. Subscutellum entirely black. Dorsocentral seta aligned anterior to
postsutural supra-alar seta. Anepisternal and anepimeral setae white. 

Wing. Anterior
apical band with window along vein R2+3 uninterrupted; subapical
band always surpassing anterior margin of cell dm; R-M ratio: 0.7-0.8; dm
ratio: 2.4-2.9.

Abdomen. Epandrium in lateral view with lateral surstylus short,
about as long as epandrium; posterior lobe of lateral surstylus reduced, not
extending posteriorly; medial surstylus directed more posteriorly than median
part of lateral surstylus, with at least part of prensisetae visible.

    

Female

Oviscape
completely covered by short, dense setulae and with longer setae apically;
setae about three times as long as setulae. Tergal-oviscapal
measure: 2-2.5. Aculeus relatively broad nearly to apex, abruptly constricted
to long apex.

 

(Description
after De Meyer & Freidberg, 2005)
