# Supplementary material for: A set of multi-entry identification keys to African frugivorous flies (Diptera, Tephritidae)
Source: Zookeys. 2014 Jul 24;(428):97–108. doi: 10.3897/zookeys.428.7366 (PMC4143993; doi:10.3897/zookeys.428.7366)
Supplement: Supplementary material 4 — Key to Capparimyia [file zookeys-428-097-s004.zip › SF4_ZooKeys_key to Capparimyia/key/SF4_ZooKeys_key to Capparimyia/Media/Html/Capparimyia savastani.htm]

Capparimyia savastani (Martelli) (Figs xx-xx)


***Capparimyia savastani*** **(Martelli)**

*Ceratitis* *savastani* Martelli, 1910: 2

Body
length.
G 3.10-3.70
mm, E 3.70-4.60; wing
length: 3.30-4.15 mm.

Male

Head. First flagellomere obtuse
apically. Arista short pubescent, rays shorter than width of arista at base.
Frontal setae equal to or longer than posterior orbital seta; two orbital
setae, sometimes posterior seta only half as
long as anterior seta; ocellar seta present or absent, if present then
setula-like, black and thin, shorter than ocellar triangle; postocellar seta
yellowish; subequal in length to lateral vertical seta; eye/medial vertical
seta ratio: 1.3-1.6. Frons convex to flat; not protuberant. Genal setulae and
genal seta mostly whitish yellow, dorsal 2-3 setulae black.

Thorax. Scutum
largely microtrichose; black spots reduced. Black postpronotal spot restricted
to base of postpronotal seta, not joining black lateral presutural spot,
sometimes absent; black lateral presutural spot not reaching white presutural
spot; black scapular spot absent; black sutural spot usually
absent, represented only by brownish area, only
rarely distinct as small black spot; black acrostichal spot extending almost to
base of dorsocentral seta. Black presutural supra-alar spot separate from black
lateral presutural spot; black postsutural supra-alar and black intra-alar
spots separate. White postsutural vitta joining white prescutellar band; white
medial vitta extending anteriorly beyond transverse suture almost to base of
medial scapular seta. Black apical scutellar spots narrowly separated,
sometimes partly confluent; not reaching base of scutellum. Subscutellum mostly
black, with white median spot. Dorsocentral seta aligned posterior to, or at
level with, postsutural supra-alar seta. Anepisternal seta usually black, rarely
whitish yellow or reddish; anepimeral seta whitish yellow or reddish.

Wing. Anterior
apical band with windows shallow; window along vein R2+3 interrupted;
subapical band always surpassing anterior margin of cell dm; R-M ratio:
0.6-0.8; dm ratio: 2.5-3.0.

Abdomen. Epandrium in lateral view with lateral surstylus short,
shorter than epandrium; posterior lobe of lateral surstylus well developed,
extending posteriorly; medial surstylus directed
more anteriorly than median part of lateral surstylus, completely hidden behind
lateral surstylus. �

 

Female.

Tergal-oviscapal measure: 2.5-3. Aculeus apical part
relatively broad, strongly tapered to apex.

 

(Description
after De Meyer & Freidberg, 2005)
