# Supplementary material for: A set of multi-entry identification keys to African frugivorous flies (Diptera, Tephritidae)
Source: Zookeys. 2014 Jul 24;(428):97–108. doi: 10.3897/zookeys.428.7366 (PMC4143993; doi:10.3897/zookeys.428.7366)
Supplement: Supplementary material 4 — Key to Capparimyia [file zookeys-428-097-s004.zip › SF4_ZooKeys_key to Capparimyia/key/SF4_ZooKeys_key to Capparimyia/Media/Html/Capparimyia spatulata.htm]

Capparimyia spatulata sp


***Capparimyia spatulata*** **De Meyer &
Freidberg**

 

Body
length.
G 2.45-2.55
mm, E 2.75-2.85
mm;
wing length: 2.30-2.50 mm.

Male

Head. First
flagellomere acute apically. Arista short pubescent, rays shorter than width of
arista at base. Frontal setae equal to posterior orbital seta, sometimes
slightly longer; two orbital setae; ocellar seta black and thin, shorter than
ocellar triangle; postocellar seta whitish yellow,
equal in length to lateral vertical seta; eye/medial vertical seta ratio:
1.6-1.7. Frons convex; not protuberant. Genal setulae reddish brown, genal seta
whitish yellow or reddish brown.

Thorax. Scutum
largely microtrichose; black spots reduced. Black postpronotal spot confluent
with black lateral presutural spot; latter spot not
reaching white presutural spot; black scapular spot absent; black sutural spot
present, isolated from black acrostichal spot; latter spot not reaching base of dorsocentral seta. Black
presutural supra-alar spot partly confluent with black lateral presutural spot;
black postsutural supra-alar and black intra-alar spots separate or confluent.
White postsutural vitta extending posteriorly to base of intra-alar seta,
narrowly separated from white prescutellar band; white medial vitta extending
anteriorly well beyond transverse suture but not to base of medial scapular
seta. Black apical scutellar spots largely separated, confluent
with each other at apex; basally extending to base
of scutellum by brownish patch. Subscutellum entirely black.
Dorsocentral seta aligned posterior to or at level with postsutural supra-alar
seta. Anepisternal and anepimeral setae white.

Wing. Anterior
apical band with window along vein R2+3 uninterrupted, distal
portion not well demarcated; subapical band always surpassing anterior margin
of cell dm; R-M ratio: 0.6-0.9; dm ratio: 3.1-3.4.

Abdomen. Epandrium in lateral view with lateral surstylus short,
about as long as epandrium; posterior lobe of lateral surstylus reduced, not
extending posteriorly; medial surstylus directed more anteriorly than median part of lateral
surstylus, completely hidden behind lateral surstylus.

 

Female

Thoracic
markings with black spots more extensive, acrostichal spot reaching base of
dorsocentral seta, postsutural supra-alar and intra-alar spots largely
confluent. White lateral postsutural vitta confluent with prescutellar band.
Distal portion of anterior apical band in wing well
demarcated. Oviscape completely covered by
short, dense setulae and with longer setae apically; setae about three times as
long as setulae.Tergal-oviscapal measure: 2.5. Aculeus apical part broad,
rounded at tip.

 

(Description
after De Meyer & Freidberg, 2005)
