# Supplementary material for: A set of multi-entry identification keys to African frugivorous flies (Diptera, Tephritidae)
Source: Zookeys. 2014 Jul 24;(428):97–108. doi: 10.3897/zookeys.428.7366 (PMC4143993; doi:10.3897/zookeys.428.7366)
Supplement: Supplementary material 4 — Key to Capparimyia [file zookeys-428-097-s004.zip › SF4_ZooKeys_key to Capparimyia/key/SF4_ZooKeys_key to Capparimyia/Media/Html/desc_Capparimyia_aristata.html]

Natural Language Description


# A set of multi-entry identification keys to African frugivorous flies (Diptera, Tephritidae)

### Massimiliano Virgilio, Ian White, Marc De Meyer

## Capparimyia aristata

(key to Capparimyia) sex male or female. (key to Capparimyia) head (key to Capparimyia) 2. orbital setae two pairs, (key to Capparimyia) 3. first flagellomere rounded apically, (key to Capparimyia) 4. arista plumose, (key to Capparimyia) 5. ocellar setae short (about twice as long as ocellar tubercle). (key to Capparimyia) thorax (key to Capparimyia) 6. postsutural vitta (1) joining white prescutellar band, (key to Capparimyia) 8. dark scapular spot absent, (key to Capparimyia) 9. dorsocentral setae aligned at or posterior to postsutural supra-alar seta, (key to Capparimyia) 10. postpronotal spot isolated, (key to Capparimyia) 11. black apical scutellar spots separated, (key to Capparimyia) 12. subscutellum divided medially by a pale line, (key to Capparimyia) 13. black sutural spots defined and separated, (key to Capparimyia) 14. anepisternal setae black or reddish to white not black. (key to Capparimyia) abdomen (key to Capparimyia) 15. (females) aculeus tip sinusoid, (key to Capparimyia) 16. (females) sinusoid aculeus tip (1) simple, (key to Capparimyia) 17. (females) sinusoid aculeus tip (2) not narrow, (key to Capparimyia) 18. (males) surstylus longer than epandrium, (key to Capparimyia) 19. (males) posterior lobe of lateral surstylus reduced.
