# Supplementary material for: A set of multi-entry identification keys to African frugivorous flies (Diptera, Tephritidae)
Source: Zookeys. 2014 Jul 24;(428):97–108. doi: 10.3897/zookeys.428.7366 (PMC4143993; doi:10.3897/zookeys.428.7366)
Supplement: Supplementary material 4 — Key to Capparimyia [file zookeys-428-097-s004.zip › SF4_ZooKeys_key to Capparimyia/key/SF4_ZooKeys_key to Capparimyia/Media/Html/desc_Capparimyia_mirabilis.html]

Natural Language Description


# A set of multi-entry identification keys to African frugivorous flies (Diptera, Tephritidae)

### Massimiliano Virgilio, Ian White, Marc De Meyer

## Capparimyia mirabilis

(key to Capparimyia) sex male or female. (key to Capparimyia) head (key to Capparimyia) 2. orbital setae two pairs, (key to Capparimyia) 3. first flagellomere rounded apically, (key to Capparimyia) 4. arista pubescent, (key to Capparimyia) 5. ocellar setae long (about three times as long as ocellar tubercle). (key to Capparimyia) thorax (key to Capparimyia) 6. postsutural vitta (1) not joining white prescutellar band (only reaching base of intra-alar seta or about half way to it), (key to Capparimyia) 7. postsutural vitta (2) reaching intra-alar seta, (key to Capparimyia) 8. dark scapular spot present, (key to Capparimyia) 9. dorsocentral setae aligned anterior to postsutural supra-alar seta, (key to Capparimyia) 10. postpronotal spot confluent, (key to Capparimyia) 11. black apical scutellar spots merged, (key to Capparimyia) 12. subscutellum completely black, (key to Capparimyia) 13. black sutural spots merged, (key to Capparimyia) 14. anepisternal setae black or reddish to white not black. (key to Capparimyia) abdomen (key to Capparimyia) 15. (females) aculeus tip sinusoid, (key to Capparimyia) 16. (females) sinusoid aculeus tip (1) simple, (key to Capparimyia) 17. (females) sinusoid aculeus tip (2) narrow constricted, (key to Capparimyia) 18. (males) surstylus longer than epandrium, (key to Capparimyia) 19. (males) posterior lobe of lateral surstylus reduced.
