# Supplementary figures and images for: A set of multi-entry identification keys to African frugivorous flies (Diptera, Tephritidae) (part 1 of 6)
Source: Zookeys. 2014 Jul 24;(428):97–108. doi: 10.3897/zookeys.428.7366 (PMC4143993; doi:10.3897/zookeys.428.7366)

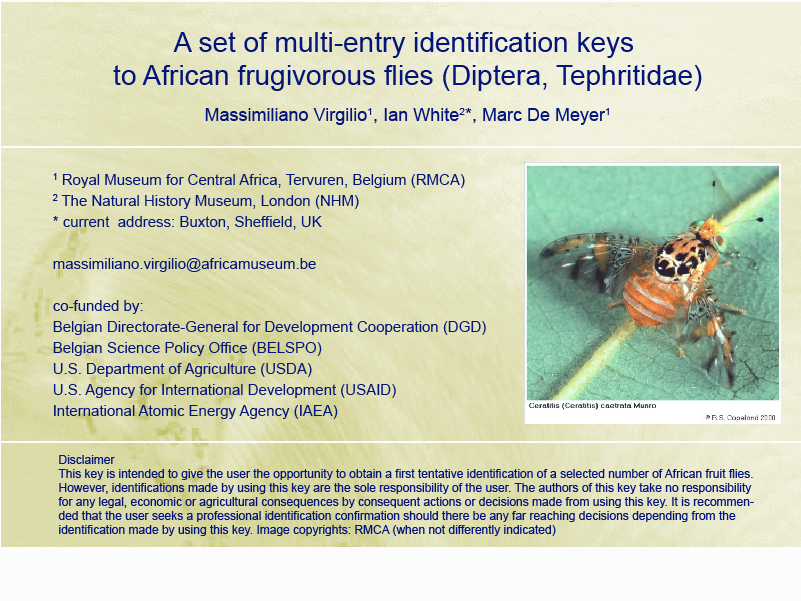

Supplement: Supplementary material 4 — Key to Capparimyia [file zookeys-428-097-s004.zip › SF4_ZooKeys_key to Capparimyia/key/SF4_ZooKeys_key to Capparimyia/Media/Images/0_cover.jpg]

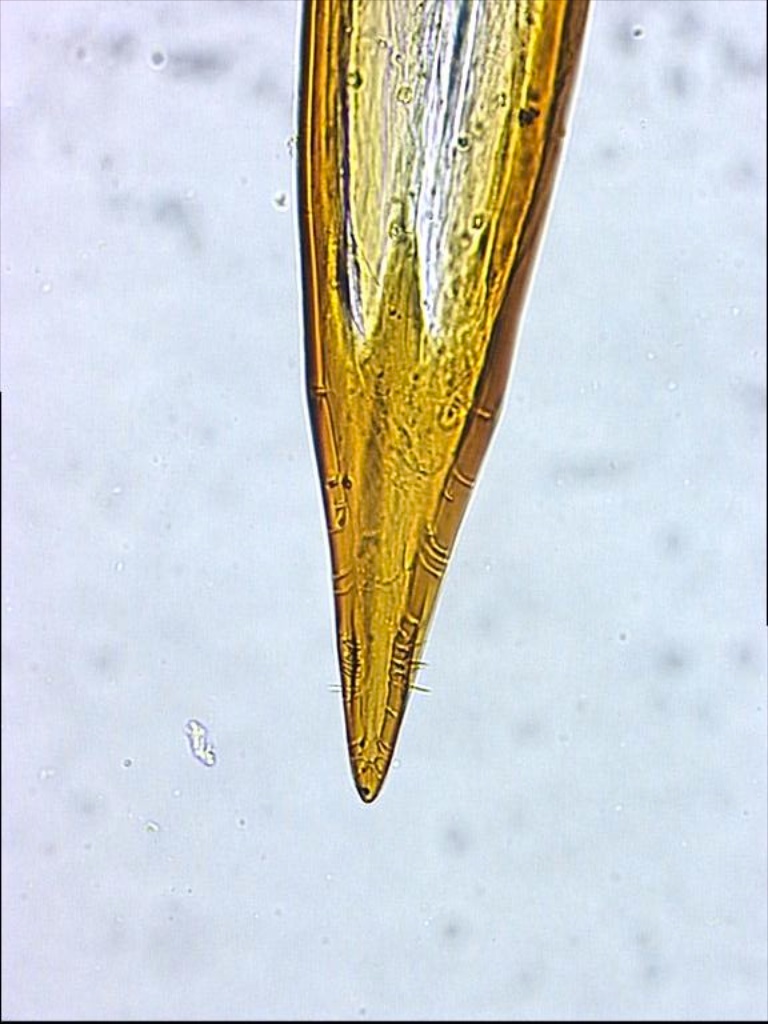

Supplement: Supplementary material 4 — Key to Capparimyia [file zookeys-428-097-s004.zip › SF4_ZooKeys_key to Capparimyia/key/SF4_ZooKeys_key to Capparimyia/Media/Images/104 aculeus apex dorsal (automontage (c) RMCA).jpg]

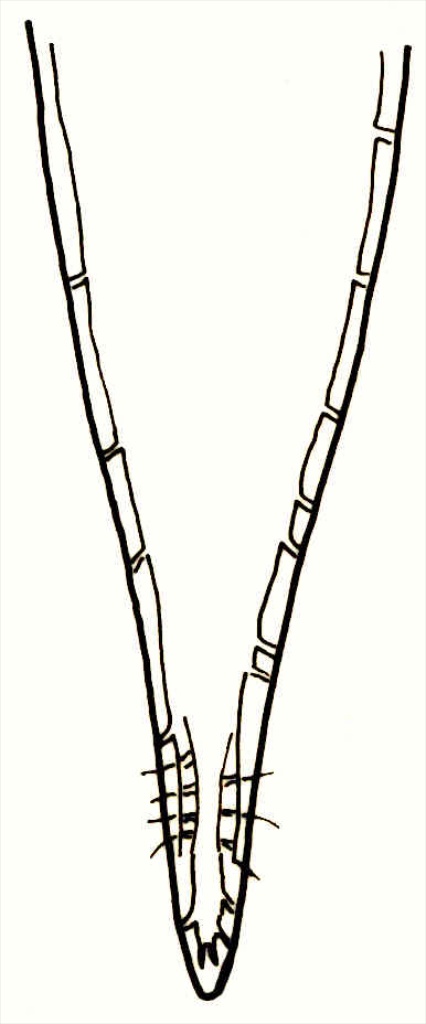

Supplement: Supplementary material 4 — Key to Capparimyia [file zookeys-428-097-s004.zip › SF4_ZooKeys_key to Capparimyia/key/SF4_ZooKeys_key to Capparimyia/Media/Images/104 aculeus apex dorsal (drawing (c) De Meyer & Freidberg).jpg]

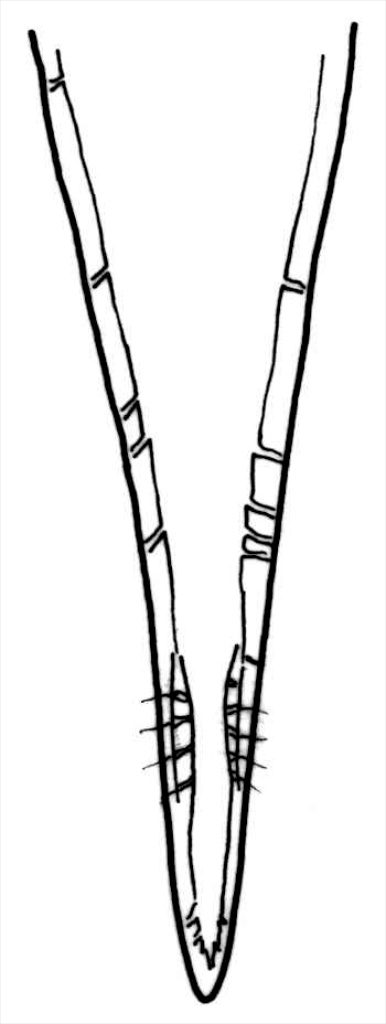

Supplement: Supplementary material 4 — Key to Capparimyia [file zookeys-428-097-s004.zip › SF4_ZooKeys_key to Capparimyia/key/SF4_ZooKeys_key to Capparimyia/Media/Images/104 aculeus apex variant dorsal (drawing (c) De Meyer & Freidberg).jpg]

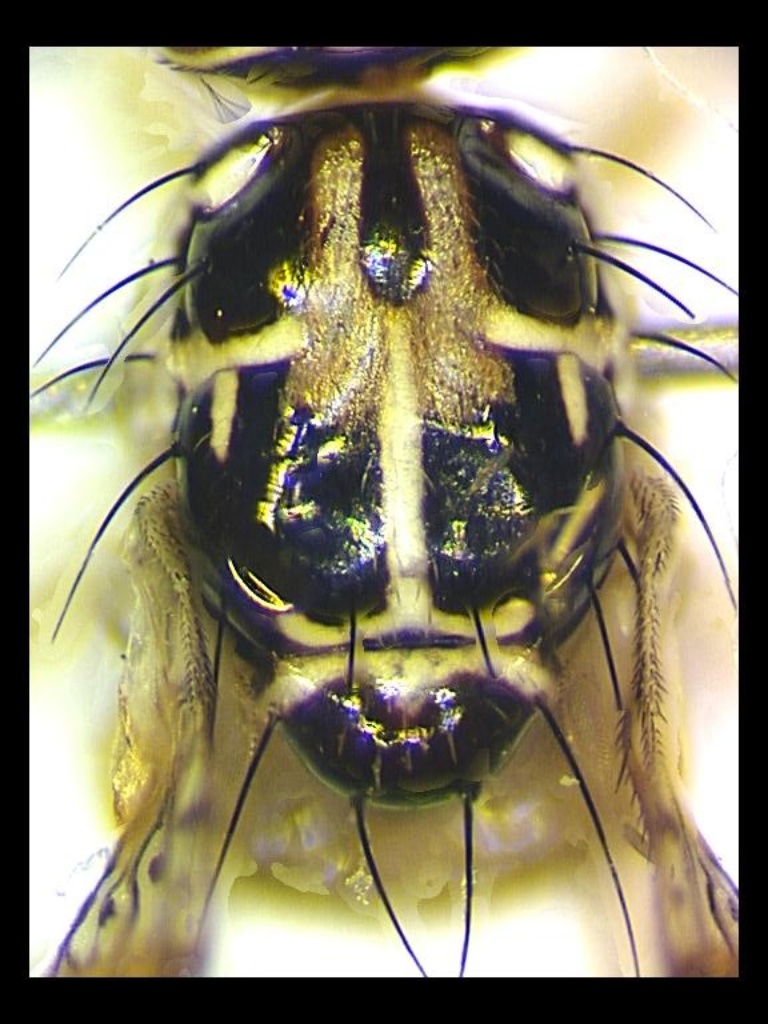

Supplement: Supplementary material 4 — Key to Capparimyia [file zookeys-428-097-s004.zip › SF4_ZooKeys_key to Capparimyia/key/SF4_ZooKeys_key to Capparimyia/Media/Images/104 mesonotum dorsal (automontage (c) RMCA).jpg]

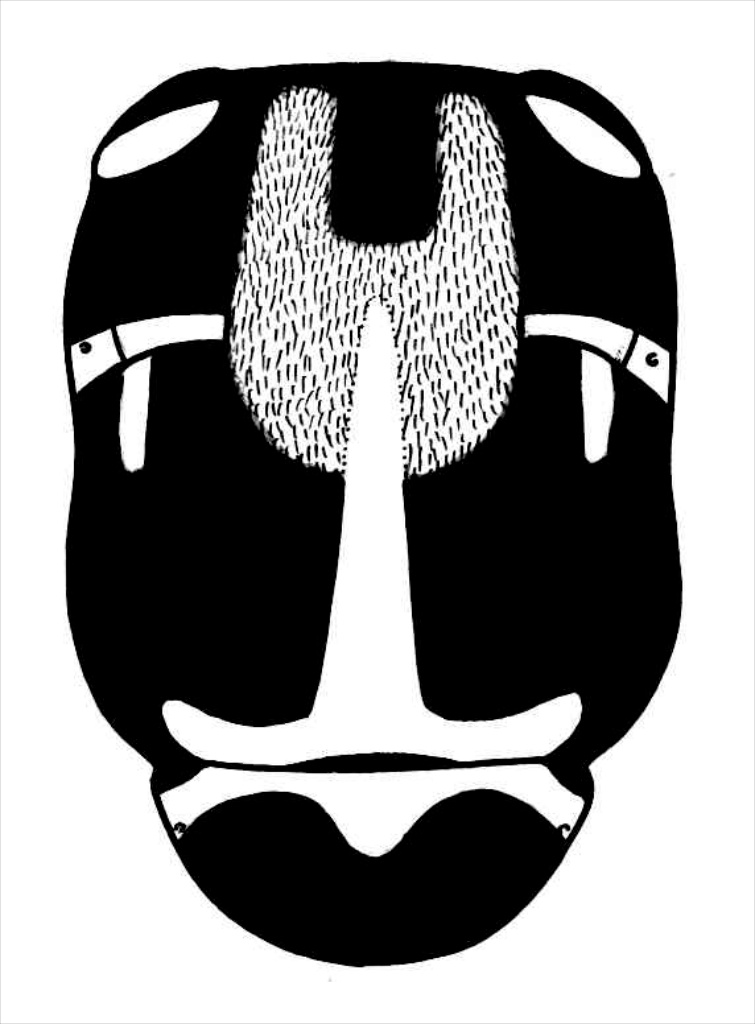

Supplement: Supplementary material 4 — Key to Capparimyia [file zookeys-428-097-s004.zip › SF4_ZooKeys_key to Capparimyia/key/SF4_ZooKeys_key to Capparimyia/Media/Images/104 mesonotum dorsal (drawing (c) De Meyer & Freidberg).jpg]

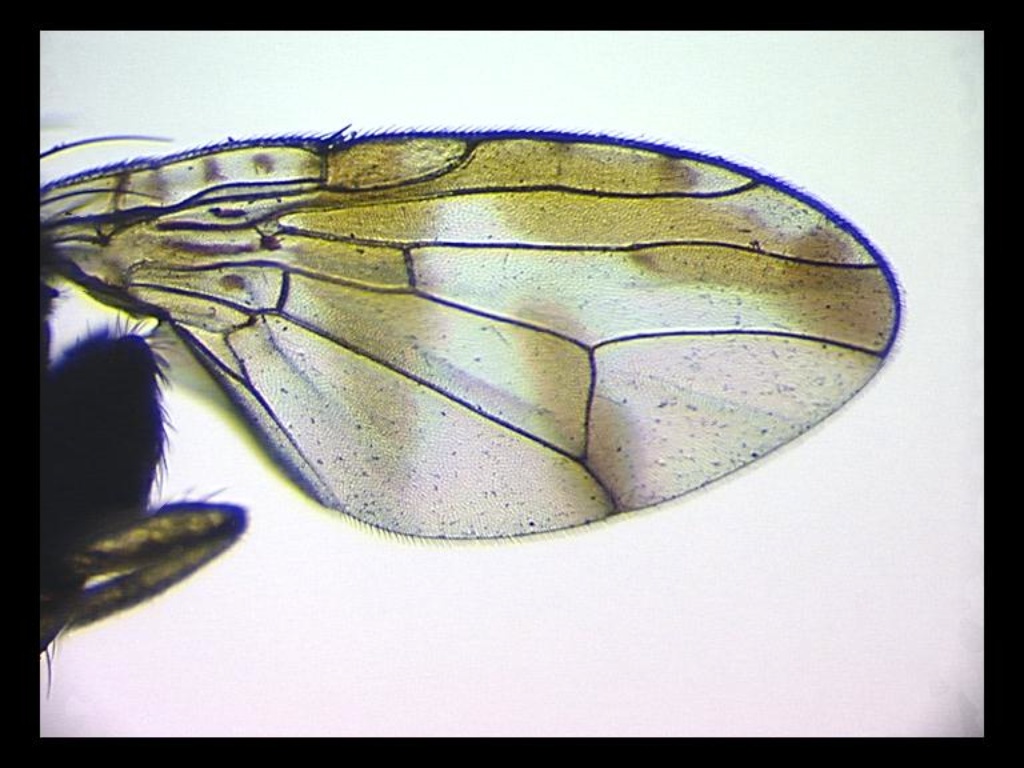

Supplement: Supplementary material 4 — Key to Capparimyia [file zookeys-428-097-s004.zip › SF4_ZooKeys_key to Capparimyia/key/SF4_ZooKeys_key to Capparimyia/Media/Images/104 wing dorsal (automontage (c) RMCA).jpg]

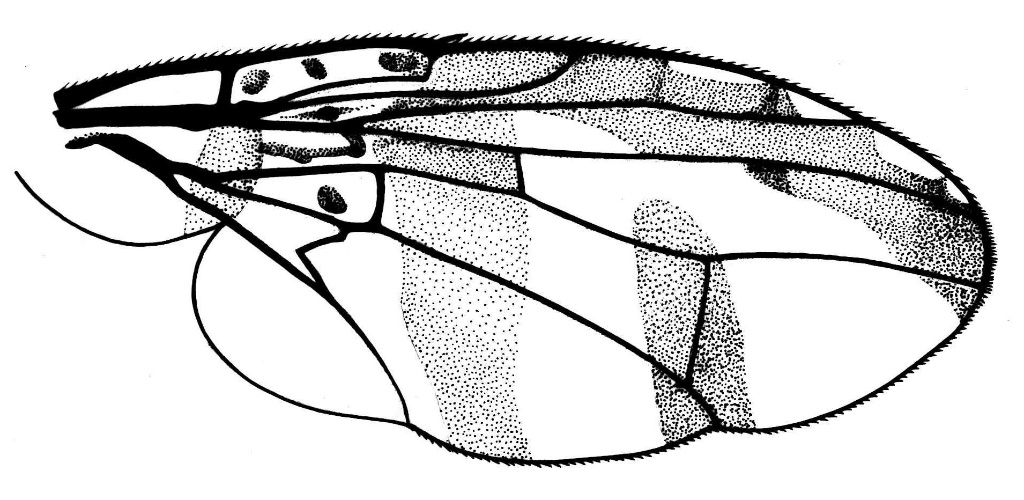

Supplement: Supplementary material 4 — Key to Capparimyia [file zookeys-428-097-s004.zip › SF4_ZooKeys_key to Capparimyia/key/SF4_ZooKeys_key to Capparimyia/Media/Images/104 wing dorsal (drawing (c) De Meyer & Freidberg).jpg]

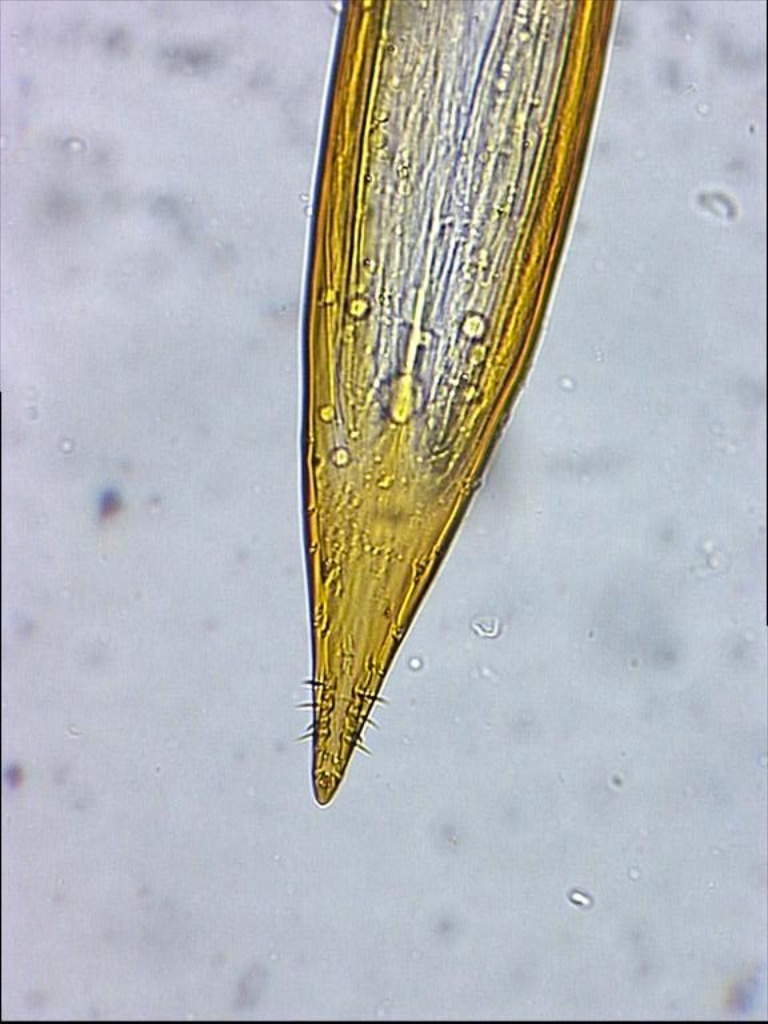

Supplement: Supplementary material 4 — Key to Capparimyia [file zookeys-428-097-s004.zip › SF4_ZooKeys_key to Capparimyia/key/SF4_ZooKeys_key to Capparimyia/Media/Images/108 aculeus apex dorsal (automontage (c) RMCA).jpg]

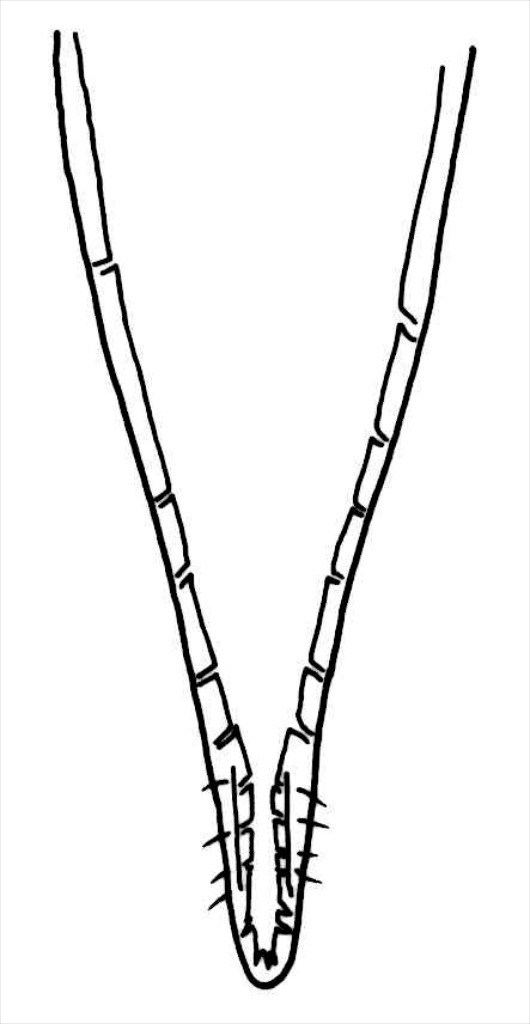

Supplement: Supplementary material 4 — Key to Capparimyia [file zookeys-428-097-s004.zip › SF4_ZooKeys_key to Capparimyia/key/SF4_ZooKeys_key to Capparimyia/Media/Images/108 aculeus apex dorsal (drawing (c) De Meyer & Freidberg).jpg]

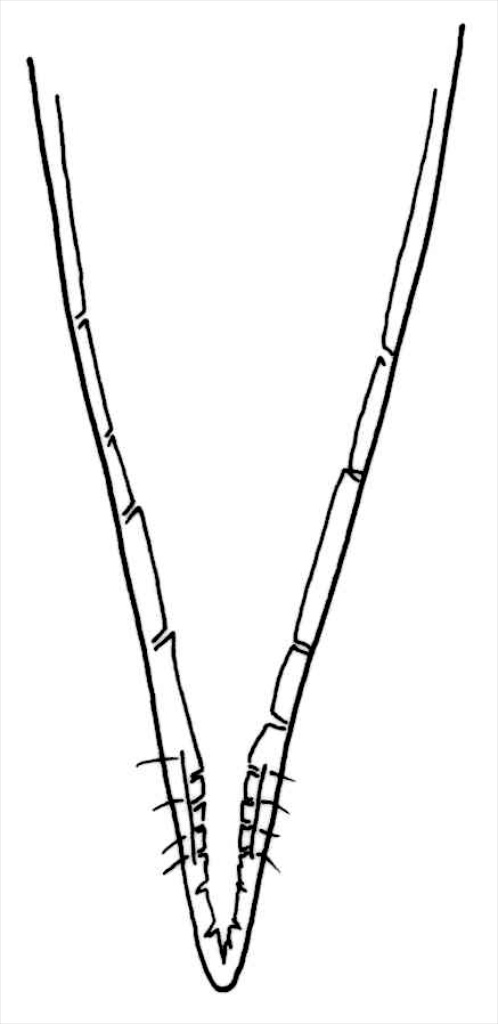

Supplement: Supplementary material 4 — Key to Capparimyia [file zookeys-428-097-s004.zip › SF4_ZooKeys_key to Capparimyia/key/SF4_ZooKeys_key to Capparimyia/Media/Images/108 aculeus apex variant dorsal (drawing (c) De Meyer & Freidberg).jpg]

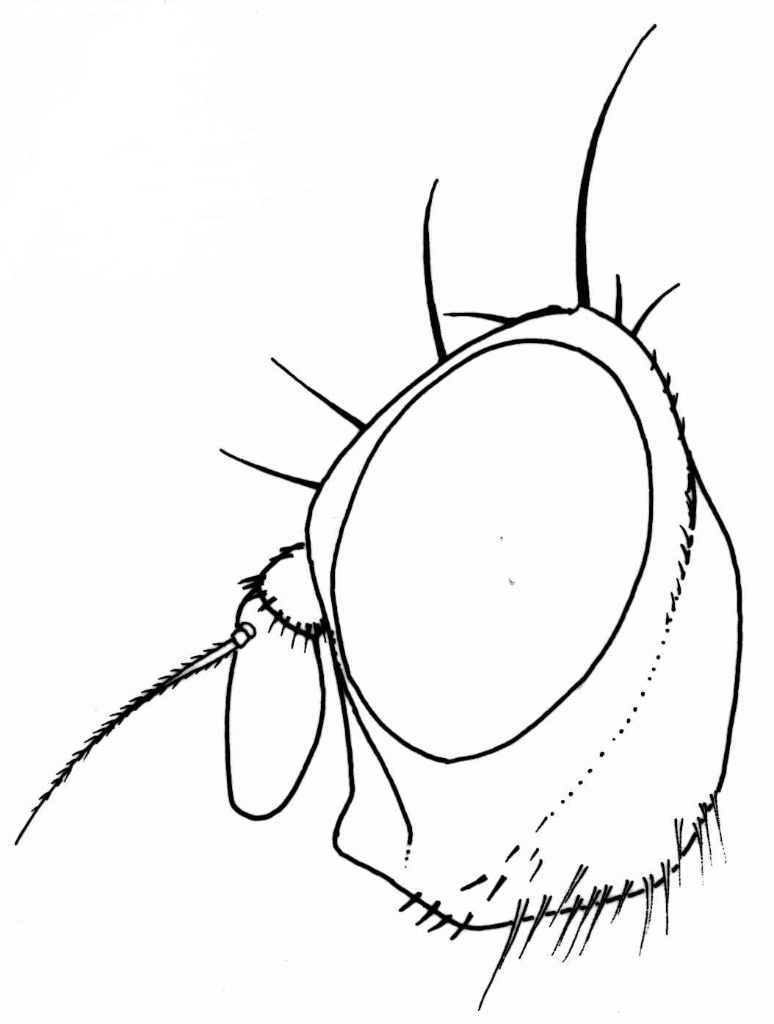

Supplement: Supplementary material 4 — Key to Capparimyia [file zookeys-428-097-s004.zip › SF4_ZooKeys_key to Capparimyia/key/SF4_ZooKeys_key to Capparimyia/Media/Images/108 head lateral (drawing (c) De Meyer & Freidberg).jpg]

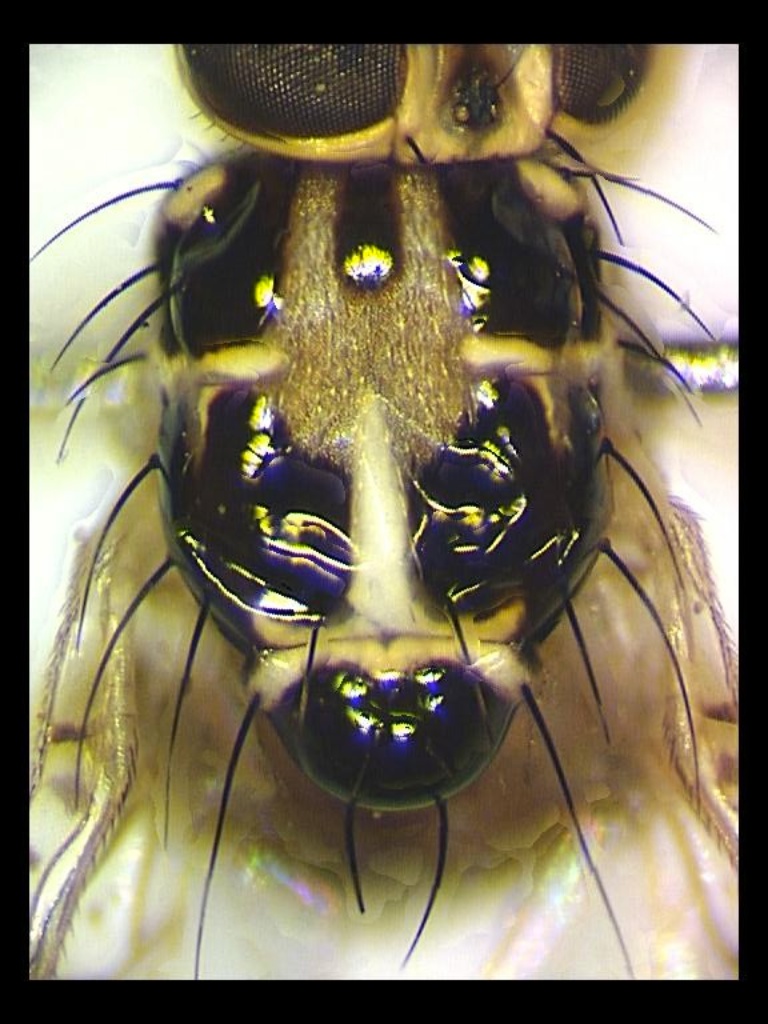

Supplement: Supplementary material 4 — Key to Capparimyia [file zookeys-428-097-s004.zip › SF4_ZooKeys_key to Capparimyia/key/SF4_ZooKeys_key to Capparimyia/Media/Images/108 mesonotum dorsal (automontage (c) RMCA).jpg]

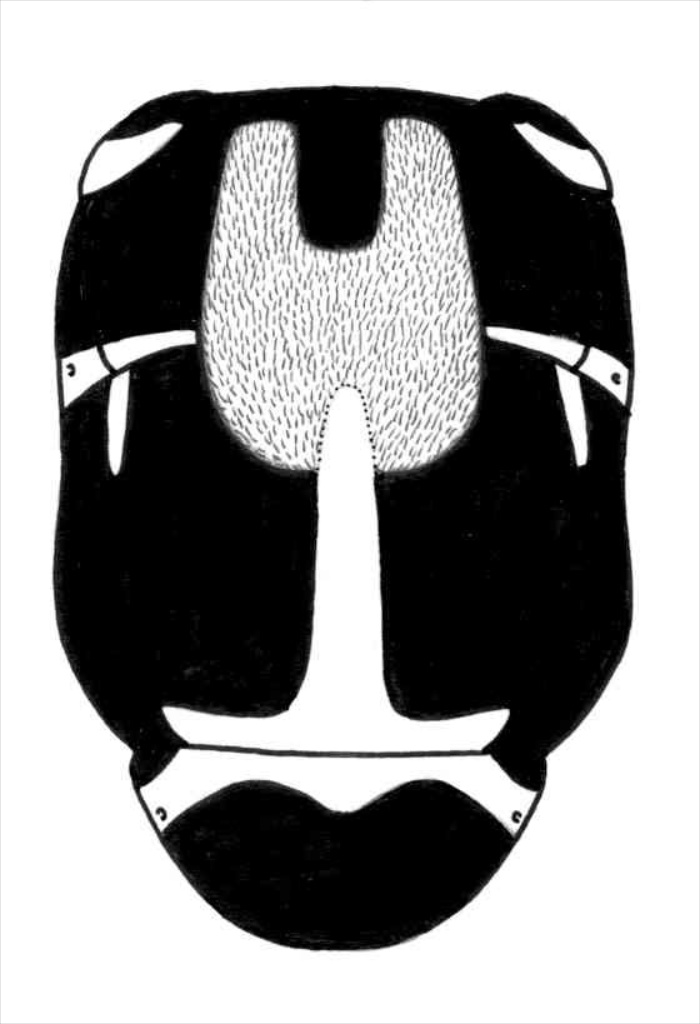

Supplement: Supplementary material 4 — Key to Capparimyia [file zookeys-428-097-s004.zip › SF4_ZooKeys_key to Capparimyia/key/SF4_ZooKeys_key to Capparimyia/Media/Images/108 mesonotum dorsal (drawing (c) De Meyer & Freidberg).jpg]

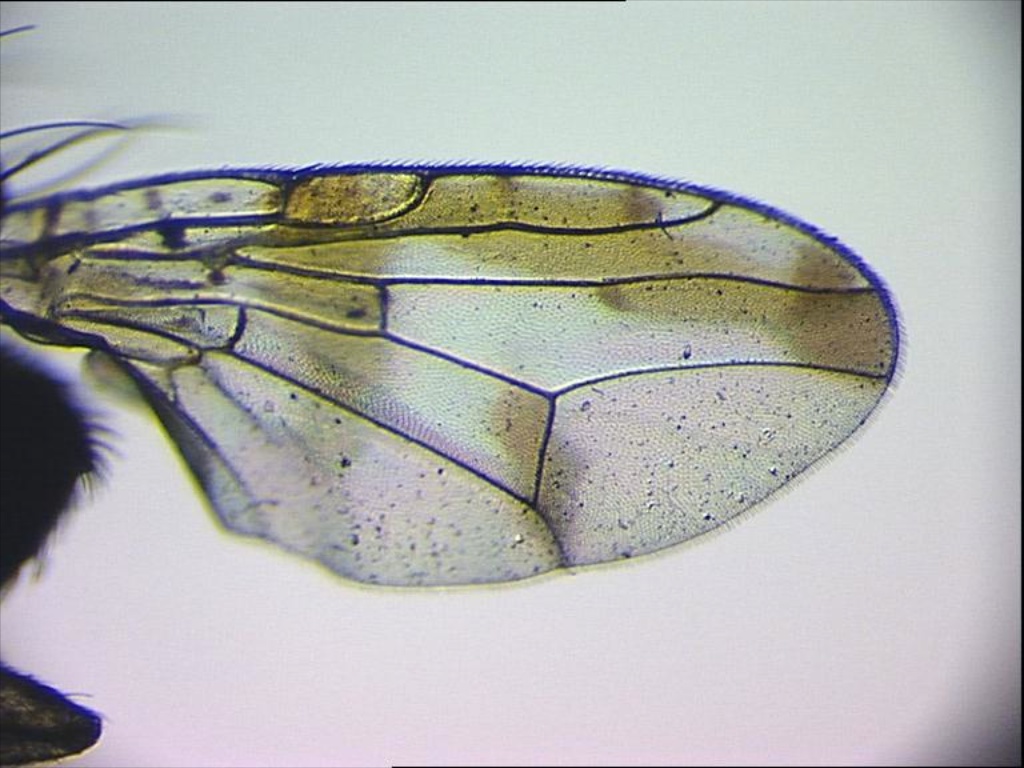

Supplement: Supplementary material 4 — Key to Capparimyia [file zookeys-428-097-s004.zip › SF4_ZooKeys_key to Capparimyia/key/SF4_ZooKeys_key to Capparimyia/Media/Images/108 wing dorsal (automontage (c) RMCA).jpg]

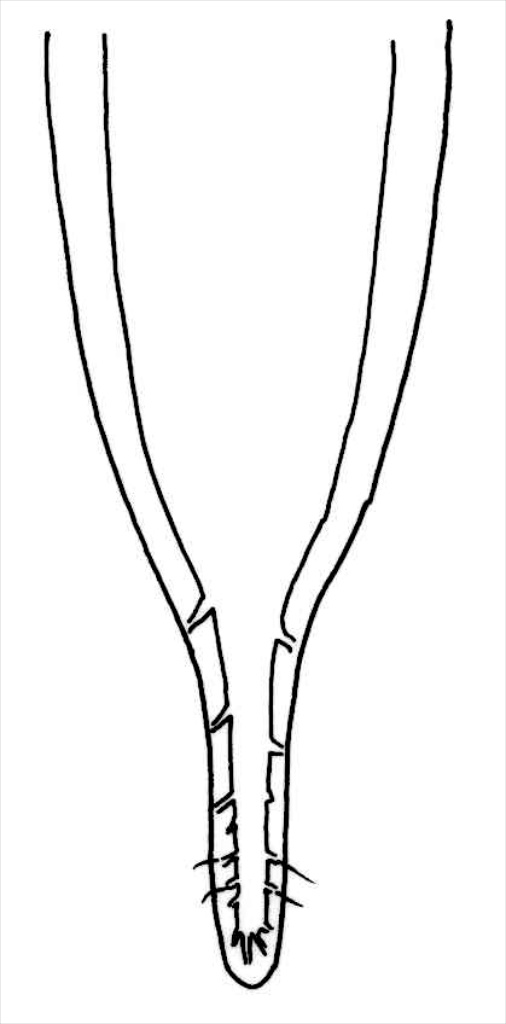

Supplement: Supplementary material 4 — Key to Capparimyia [file zookeys-428-097-s004.zip › SF4_ZooKeys_key to Capparimyia/key/SF4_ZooKeys_key to Capparimyia/Media/Images/109 aculeus apex dorsal (drawing (c) De Meyer & Freidberg).jpg]

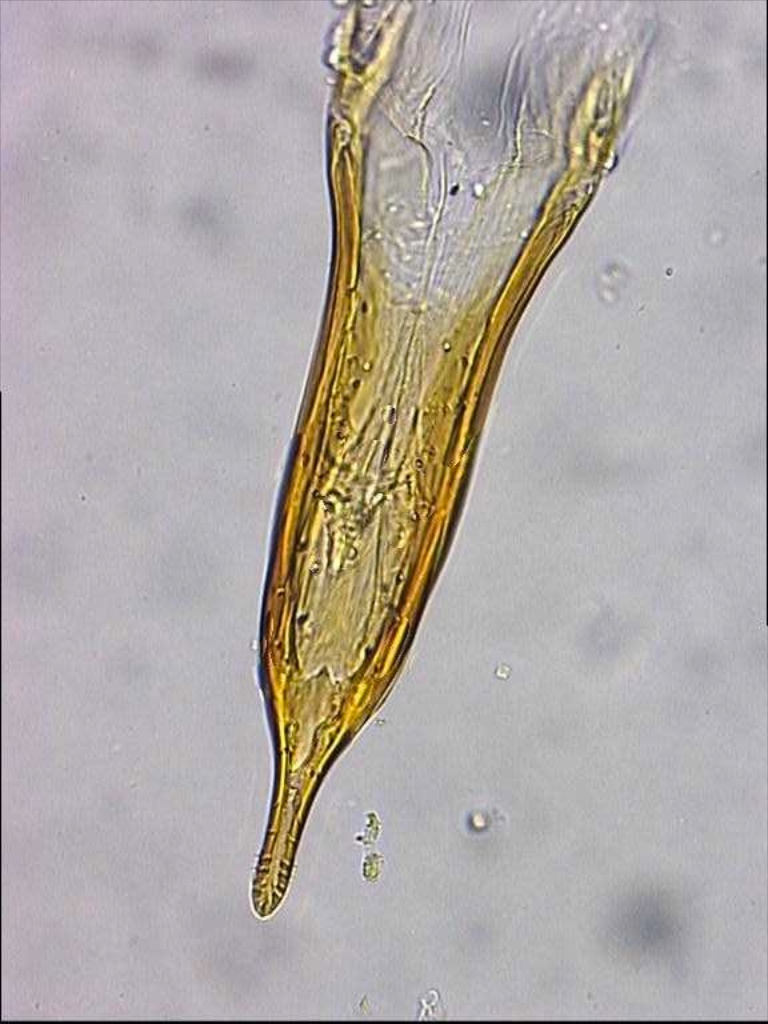

Supplement: Supplementary material 4 — Key to Capparimyia [file zookeys-428-097-s004.zip › SF4_ZooKeys_key to Capparimyia/key/SF4_ZooKeys_key to Capparimyia/Media/Images/109 aculeus dorsal (automontage (c) RMCA).jpg]

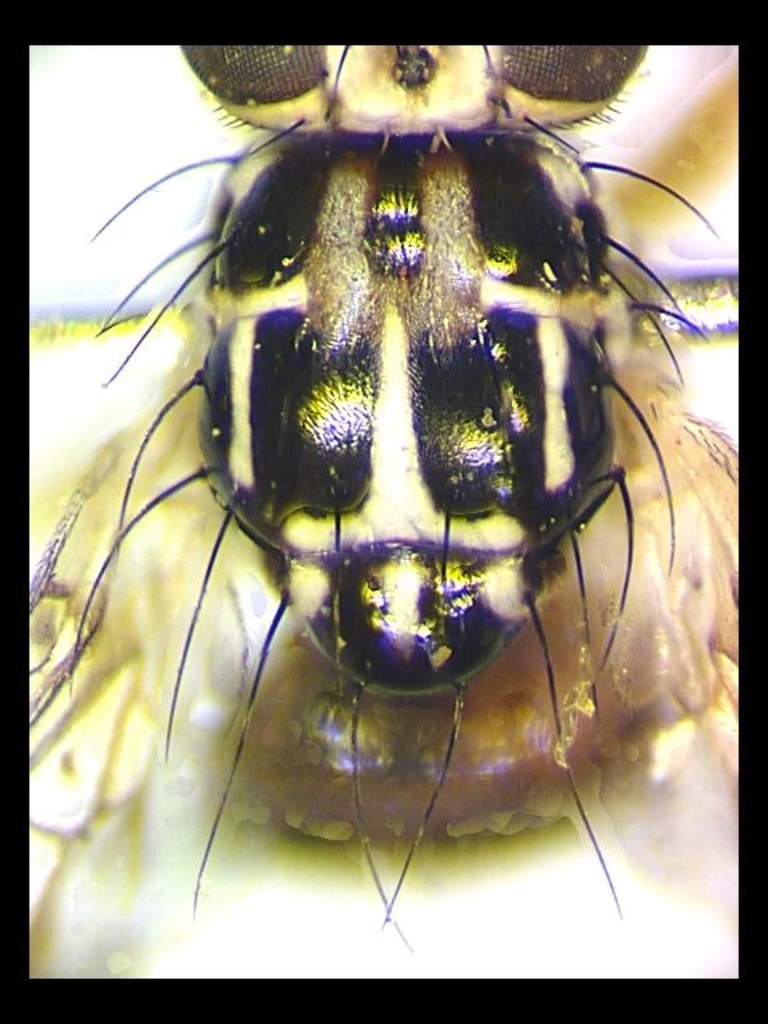

Supplement: Supplementary material 4 — Key to Capparimyia [file zookeys-428-097-s004.zip › SF4_ZooKeys_key to Capparimyia/key/SF4_ZooKeys_key to Capparimyia/Media/Images/109 mesonotum dorsal (automontage (c) RMCA).jpg]

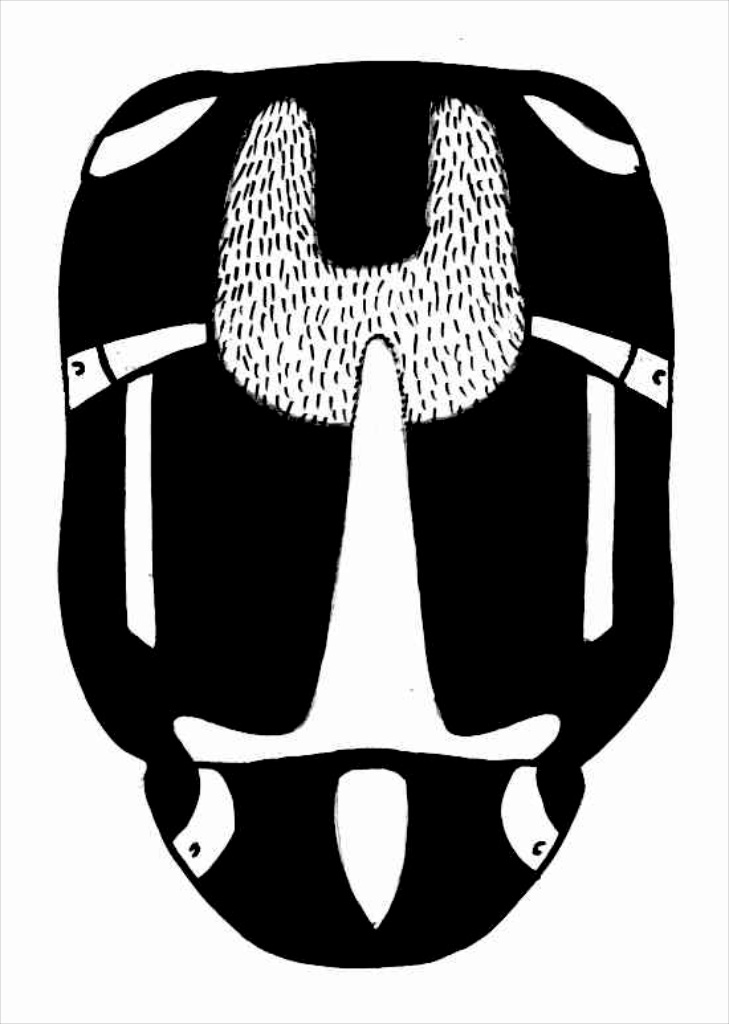

Supplement: Supplementary material 4 — Key to Capparimyia [file zookeys-428-097-s004.zip › SF4_ZooKeys_key to Capparimyia/key/SF4_ZooKeys_key to Capparimyia/Media/Images/109 mesonotum dorsal (drawing (c) De Meyer & Freidberg).jpg]

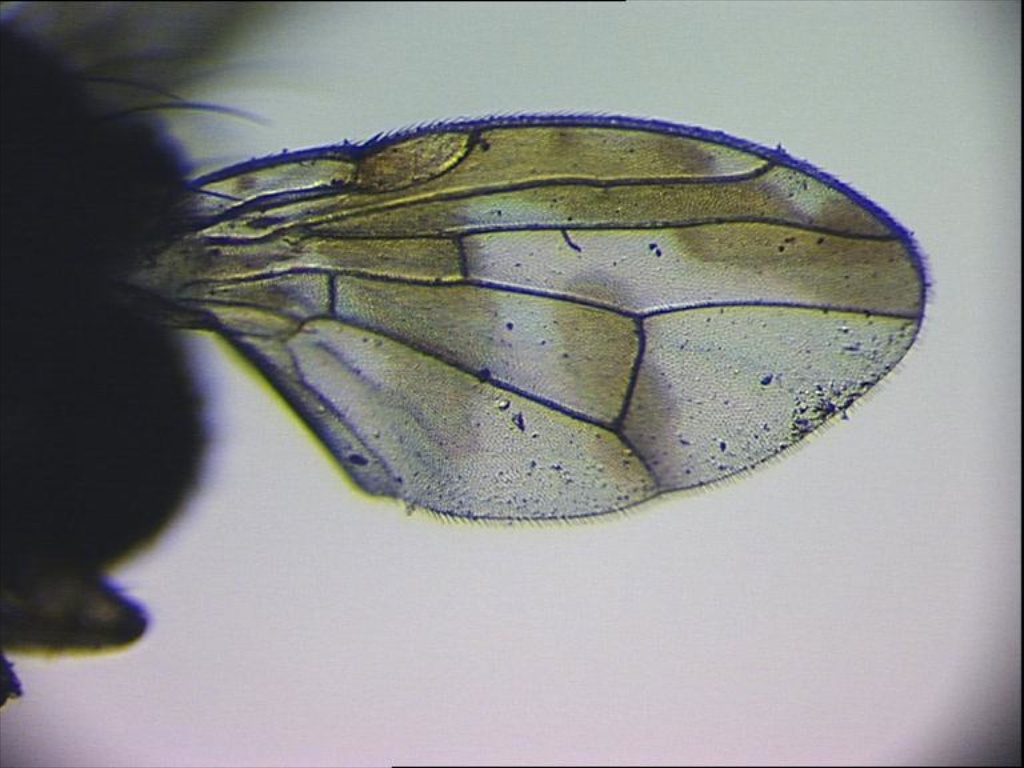

Supplement: Supplementary material 4 — Key to Capparimyia [file zookeys-428-097-s004.zip › SF4_ZooKeys_key to Capparimyia/key/SF4_ZooKeys_key to Capparimyia/Media/Images/109 wind dorsal (automontage (c) RMCA).jpg]

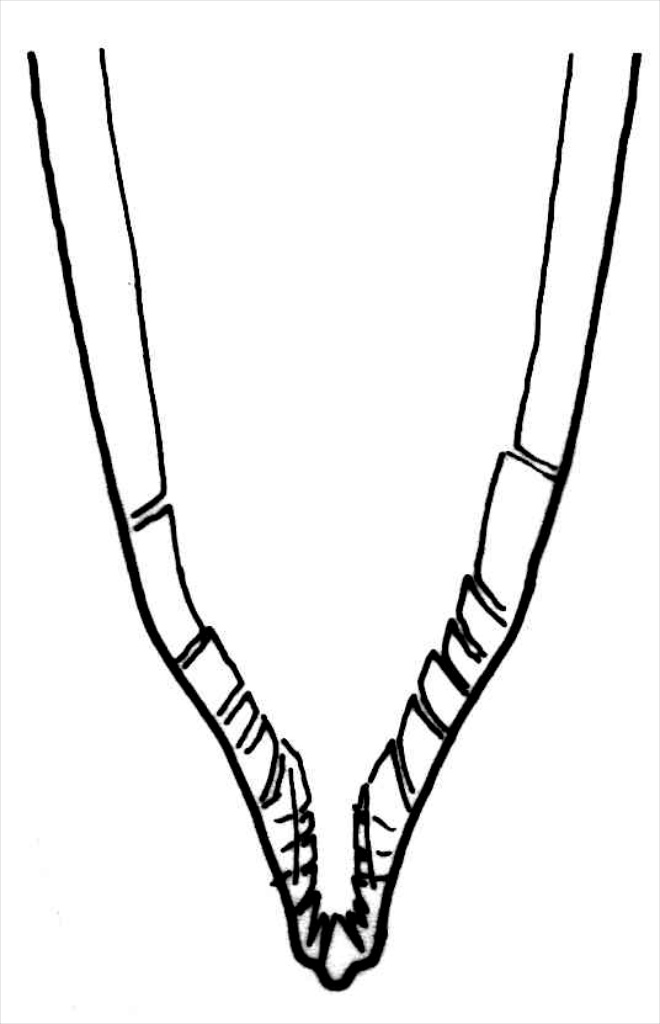

Supplement: Supplementary material 4 — Key to Capparimyia [file zookeys-428-097-s004.zip › SF4_ZooKeys_key to Capparimyia/key/SF4_ZooKeys_key to Capparimyia/Media/Images/110 aculeus apex dorsal (drawing (c) De Meyer & Freidberg).jpg]

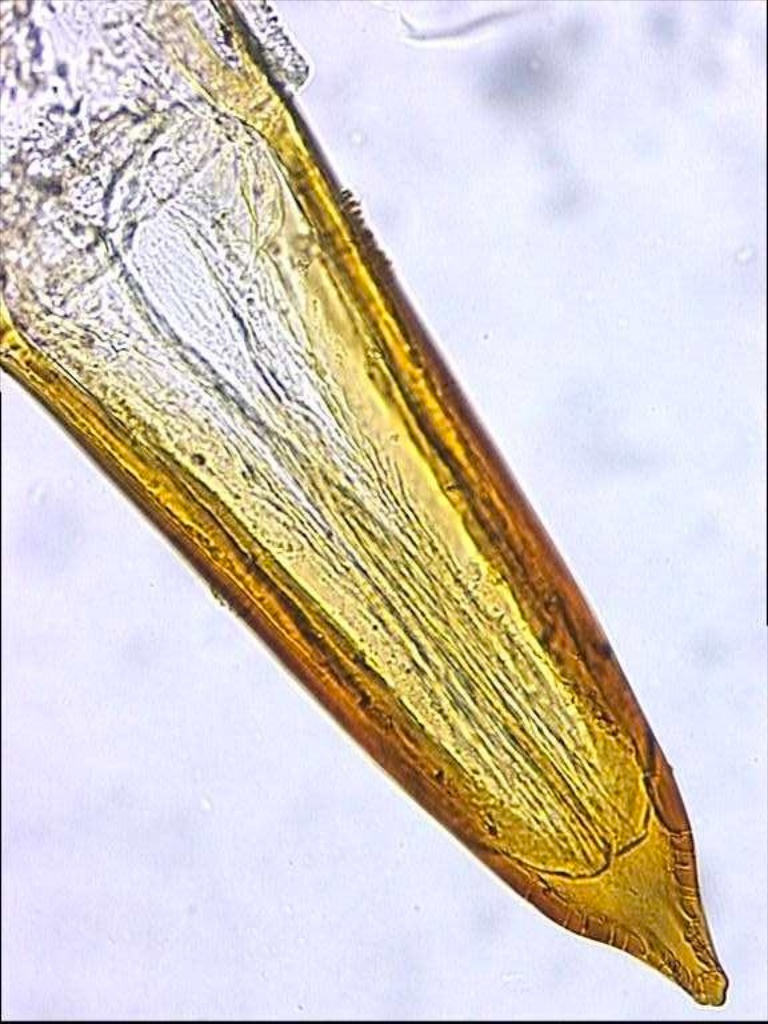

Supplement: Supplementary material 4 — Key to Capparimyia [file zookeys-428-097-s004.zip › SF4_ZooKeys_key to Capparimyia/key/SF4_ZooKeys_key to Capparimyia/Media/Images/110 aculeus dorsal (automontage (c) RMCA).jpg]

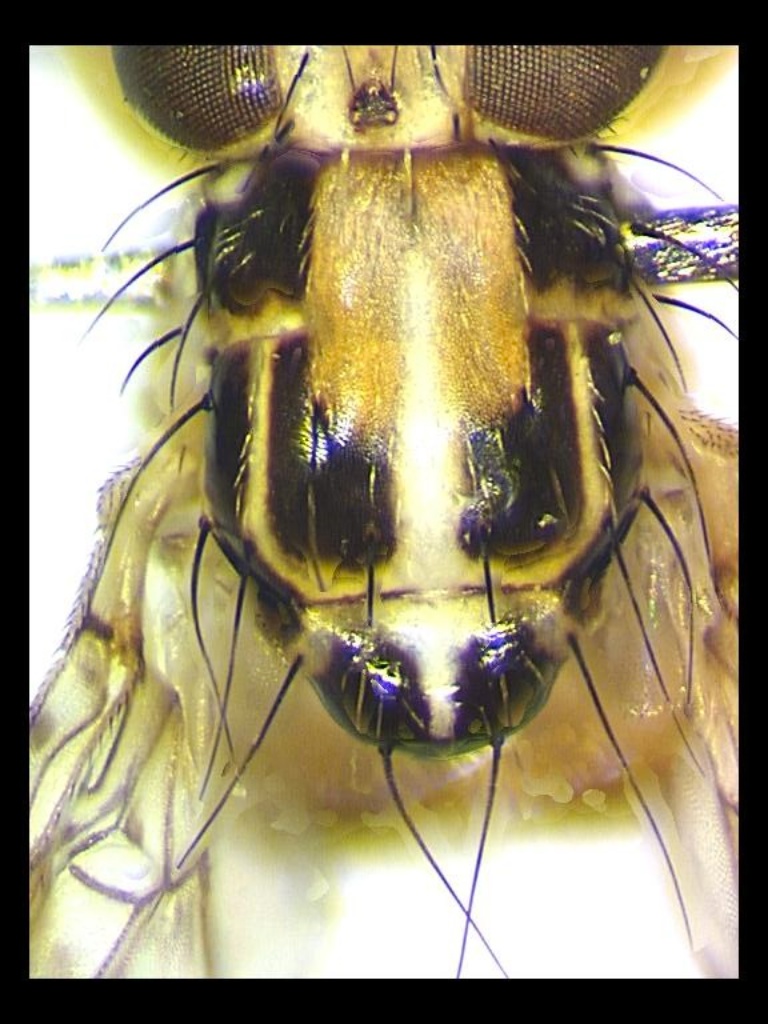

Supplement: Supplementary material 4 — Key to Capparimyia [file zookeys-428-097-s004.zip › SF4_ZooKeys_key to Capparimyia/key/SF4_ZooKeys_key to Capparimyia/Media/Images/110 mesonotum dorsal (automontage (c) RMCA).jpg]

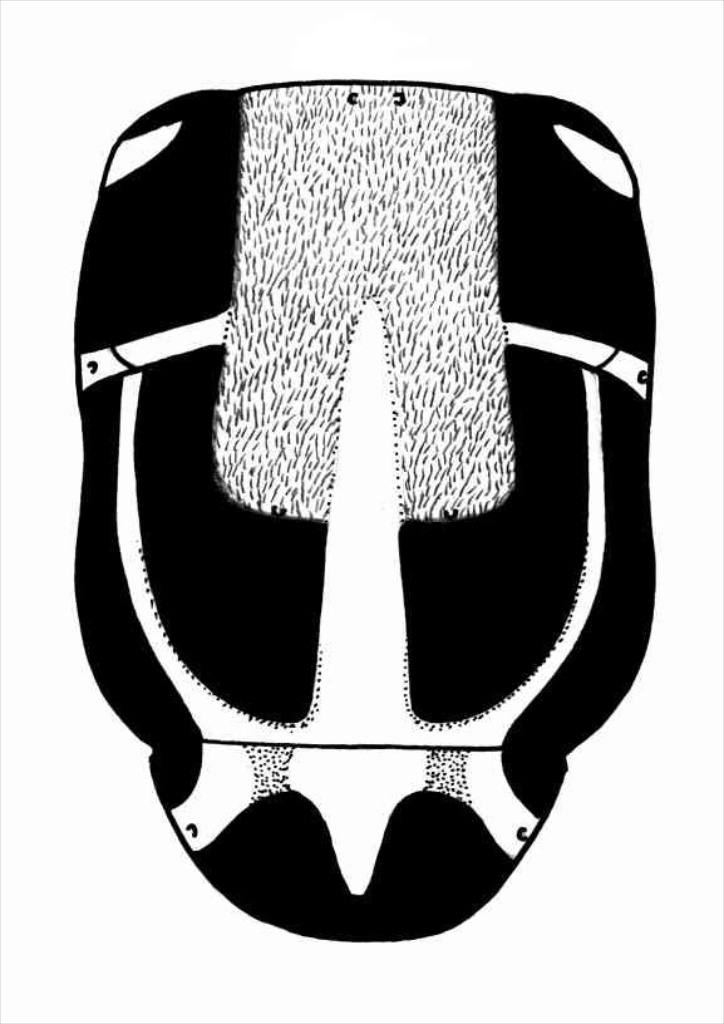

Supplement: Supplementary material 4 — Key to Capparimyia [file zookeys-428-097-s004.zip › SF4_ZooKeys_key to Capparimyia/key/SF4_ZooKeys_key to Capparimyia/Media/Images/110 mesonotum dorsal (drawing (c) De Meyer & Freidberg).jpg]

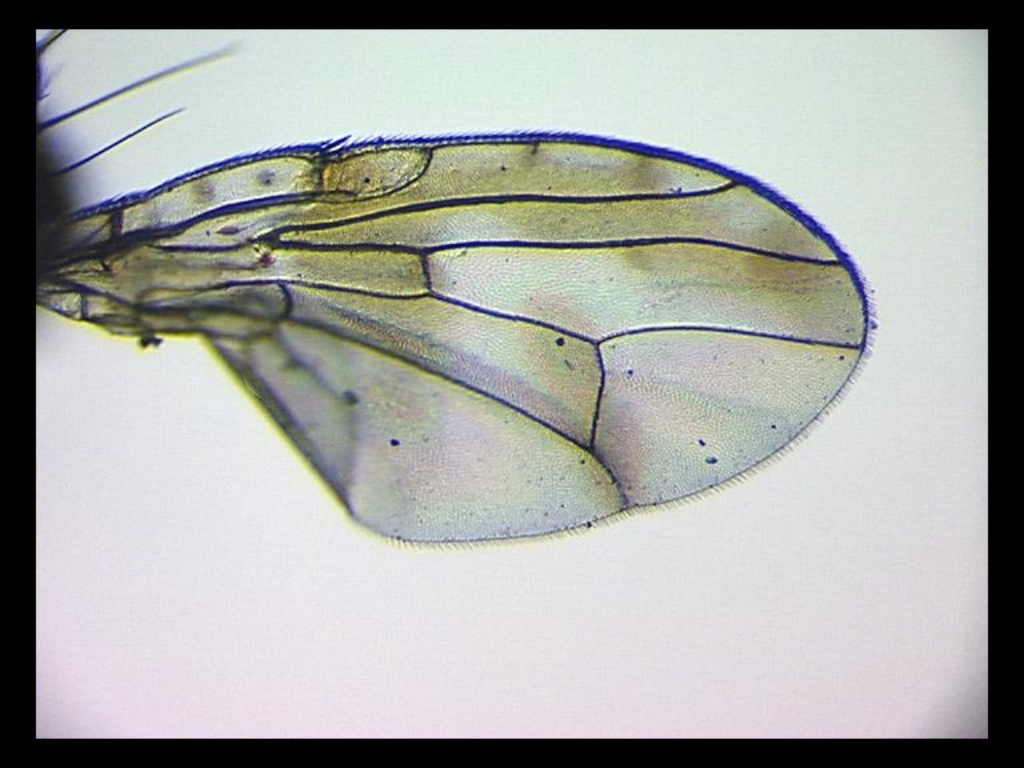

Supplement: Supplementary material 4 — Key to Capparimyia [file zookeys-428-097-s004.zip › SF4_ZooKeys_key to Capparimyia/key/SF4_ZooKeys_key to Capparimyia/Media/Images/110 wing dorsal (automontage (c) RMCA).jpg]

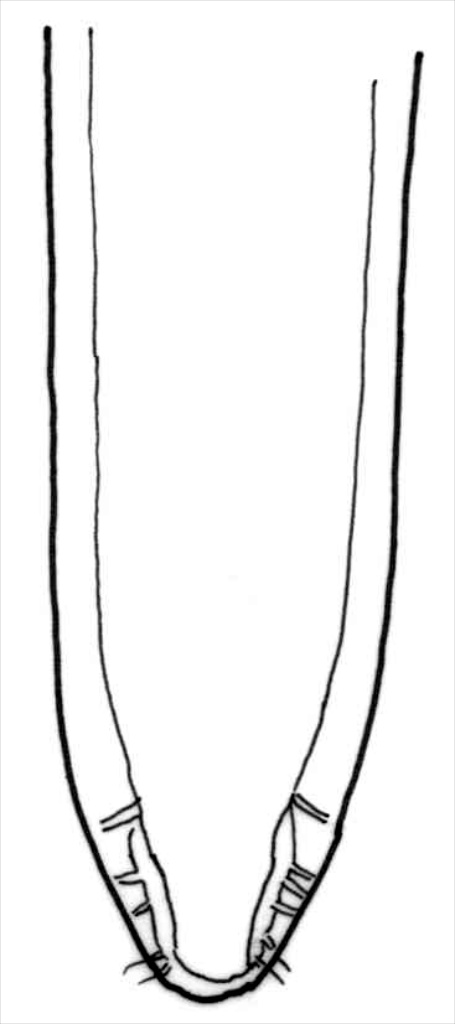

Supplement: Supplementary material 4 — Key to Capparimyia [file zookeys-428-097-s004.zip › SF4_ZooKeys_key to Capparimyia/key/SF4_ZooKeys_key to Capparimyia/Media/Images/111 aculeus apex dorsal (drawing (c) De Meyer & Freidberg).jpg]

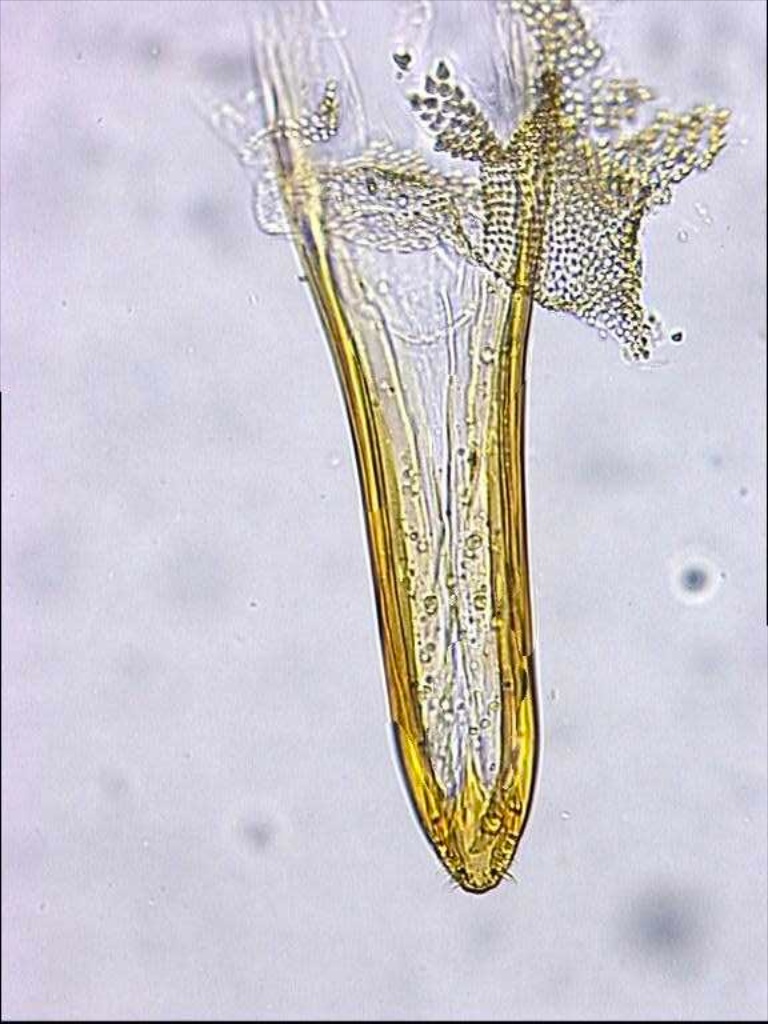

Supplement: Supplementary material 4 — Key to Capparimyia [file zookeys-428-097-s004.zip › SF4_ZooKeys_key to Capparimyia/key/SF4_ZooKeys_key to Capparimyia/Media/Images/111 aculeus dorsal (automontage (c) RMCA).jpg]

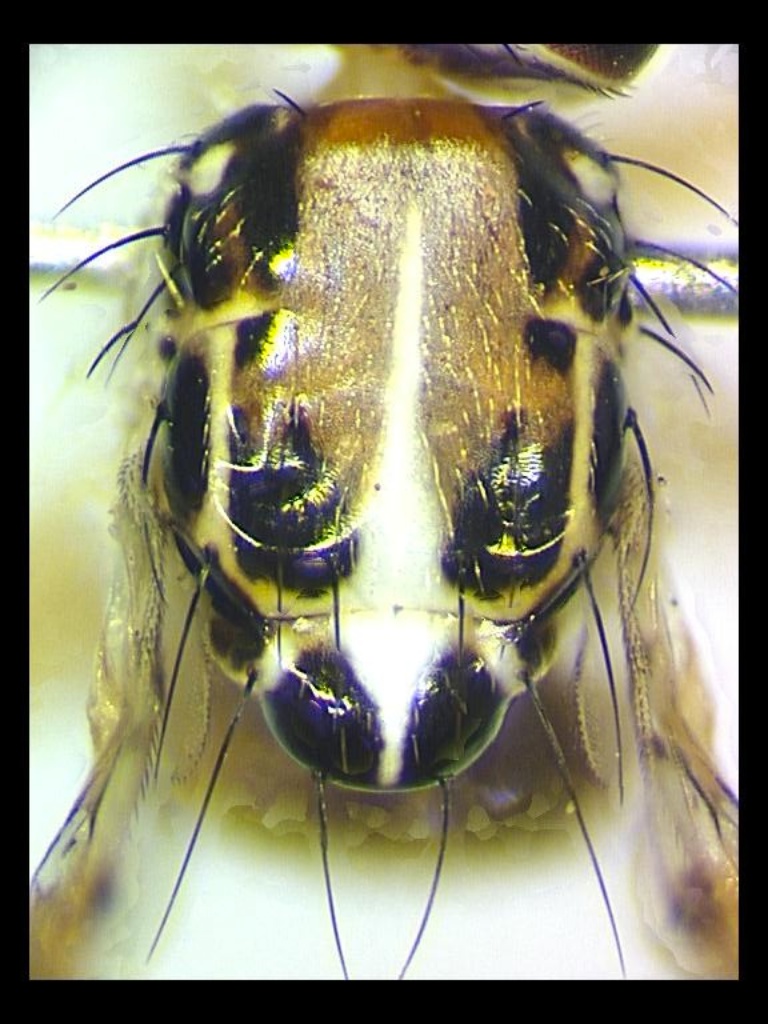

Supplement: Supplementary material 4 — Key to Capparimyia [file zookeys-428-097-s004.zip › SF4_ZooKeys_key to Capparimyia/key/SF4_ZooKeys_key to Capparimyia/Media/Images/111 mesonotum dorsal (automontage (c) RMCA).jpg]

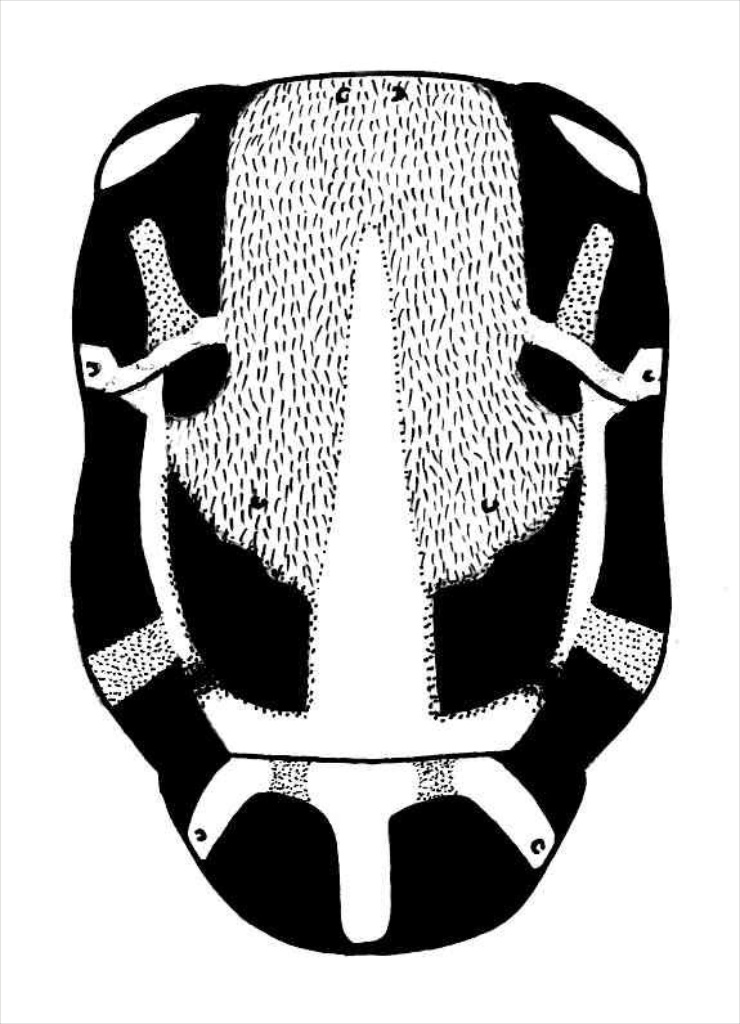

Supplement: Supplementary material 4 — Key to Capparimyia [file zookeys-428-097-s004.zip › SF4_ZooKeys_key to Capparimyia/key/SF4_ZooKeys_key to Capparimyia/Media/Images/111 mesonotum dorsal (drawing (c) De Meyer & Freidberg).jpg]

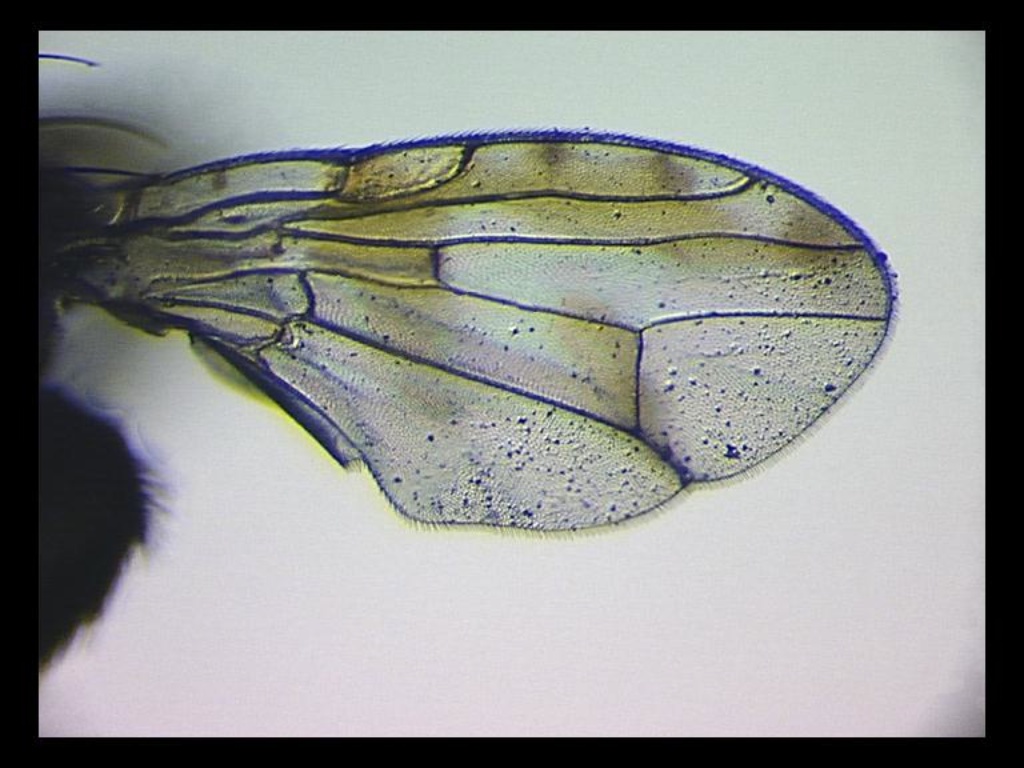

Supplement: Supplementary material 4 — Key to Capparimyia [file zookeys-428-097-s004.zip › SF4_ZooKeys_key to Capparimyia/key/SF4_ZooKeys_key to Capparimyia/Media/Images/111 wing dorsal (automontage (c) RMCA).jpg]

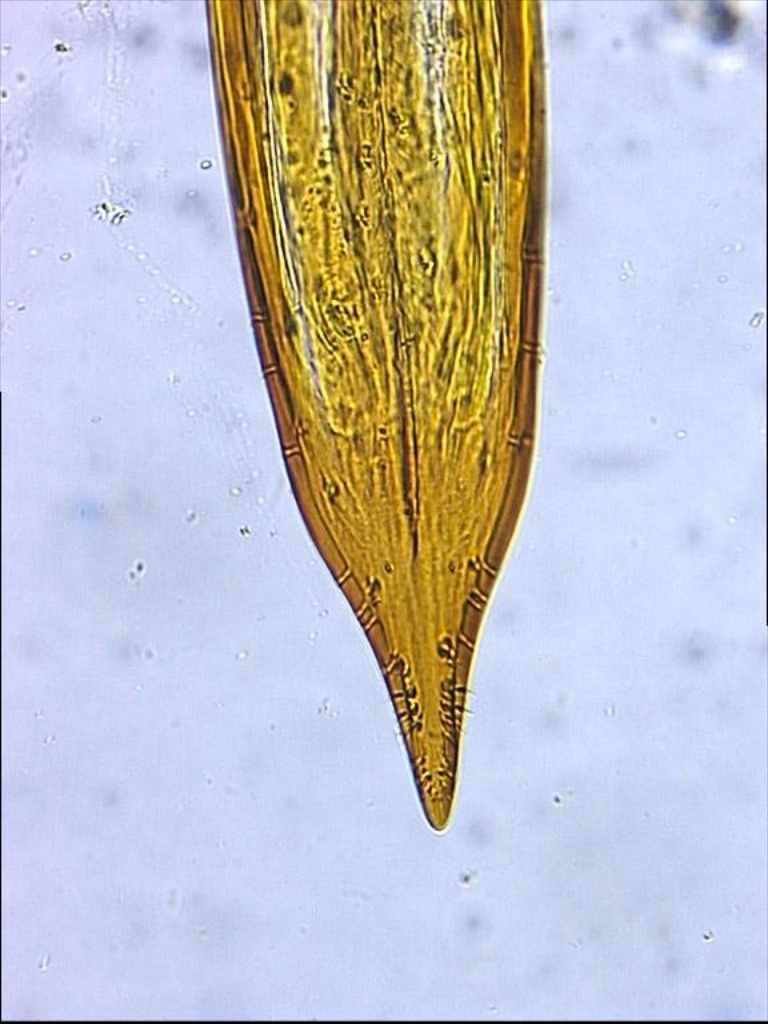

Supplement: Supplementary material 4 — Key to Capparimyia [file zookeys-428-097-s004.zip › SF4_ZooKeys_key to Capparimyia/key/SF4_ZooKeys_key to Capparimyia/Media/Images/112 aculeus apex dorsal (automontage (c) RMCA).jpg]

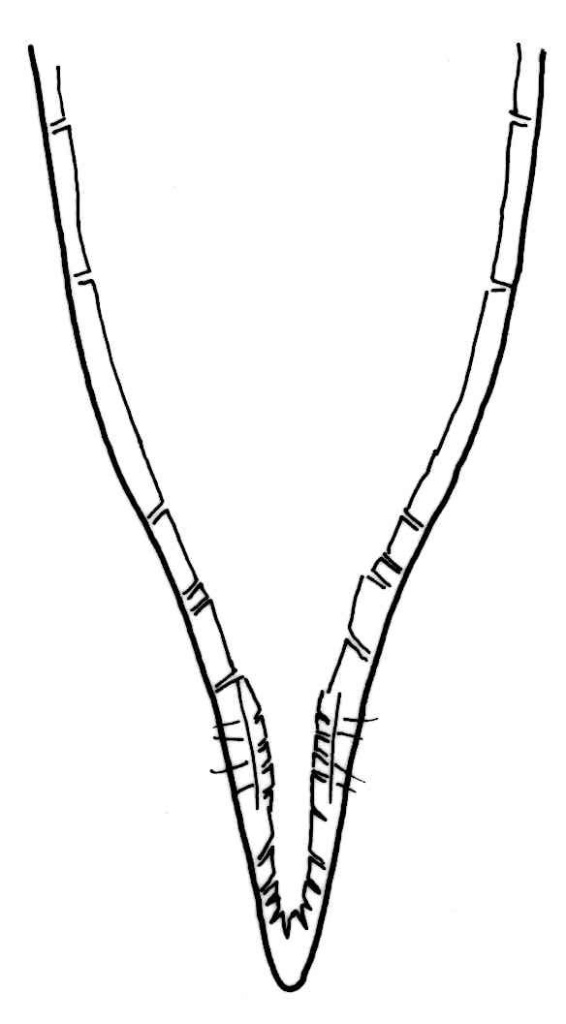

Supplement: Supplementary material 4 — Key to Capparimyia [file zookeys-428-097-s004.zip › SF4_ZooKeys_key to Capparimyia/key/SF4_ZooKeys_key to Capparimyia/Media/Images/112 aculeus apex dorsal (drawing (c) De Meyer & Freidberg).jpg]

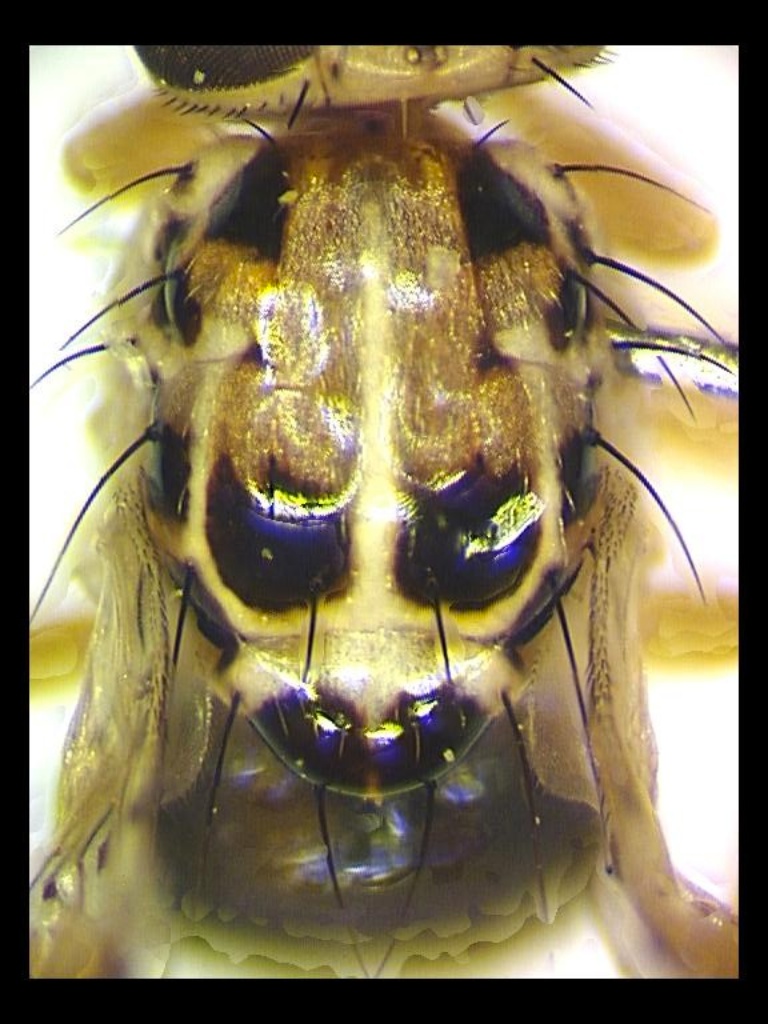

Supplement: Supplementary material 4 — Key to Capparimyia [file zookeys-428-097-s004.zip › SF4_ZooKeys_key to Capparimyia/key/SF4_ZooKeys_key to Capparimyia/Media/Images/112 mesonotum dorsal (automontage (c) RMCA).jpg]

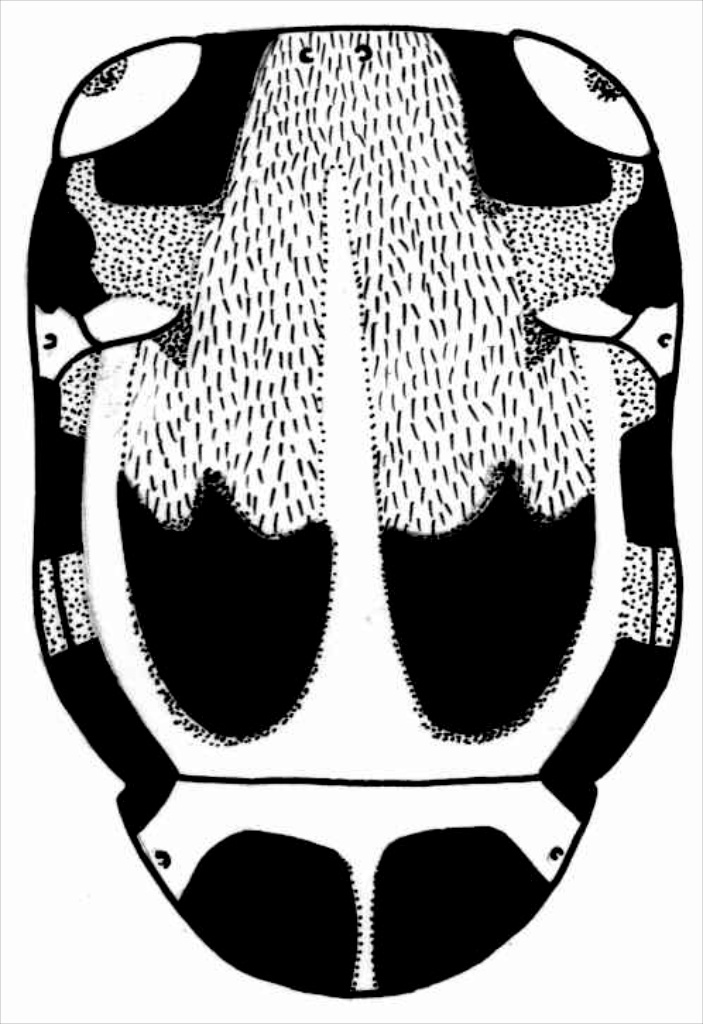

Supplement: Supplementary material 4 — Key to Capparimyia [file zookeys-428-097-s004.zip › SF4_ZooKeys_key to Capparimyia/key/SF4_ZooKeys_key to Capparimyia/Media/Images/112 mesonotum dorsal (drawing (c) De Meyer & Freidberg).jpg]

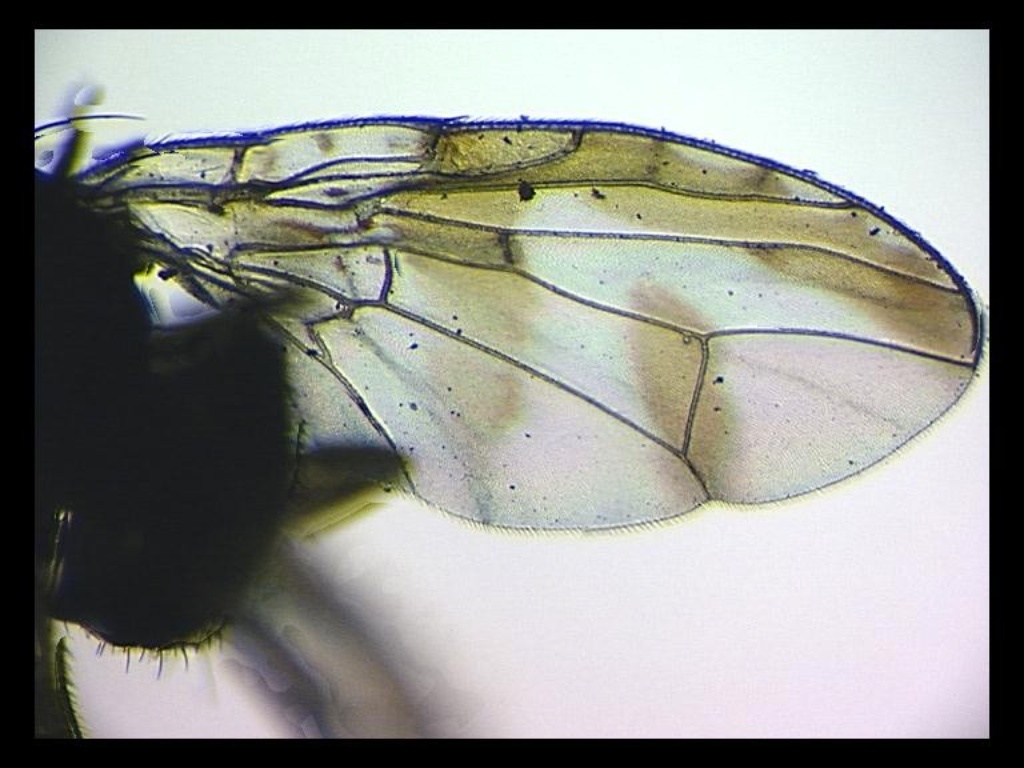

Supplement: Supplementary material 4 — Key to Capparimyia [file zookeys-428-097-s004.zip › SF4_ZooKeys_key to Capparimyia/key/SF4_ZooKeys_key to Capparimyia/Media/Images/112 wing dorsal (automontage (c) RMCA).jpg]

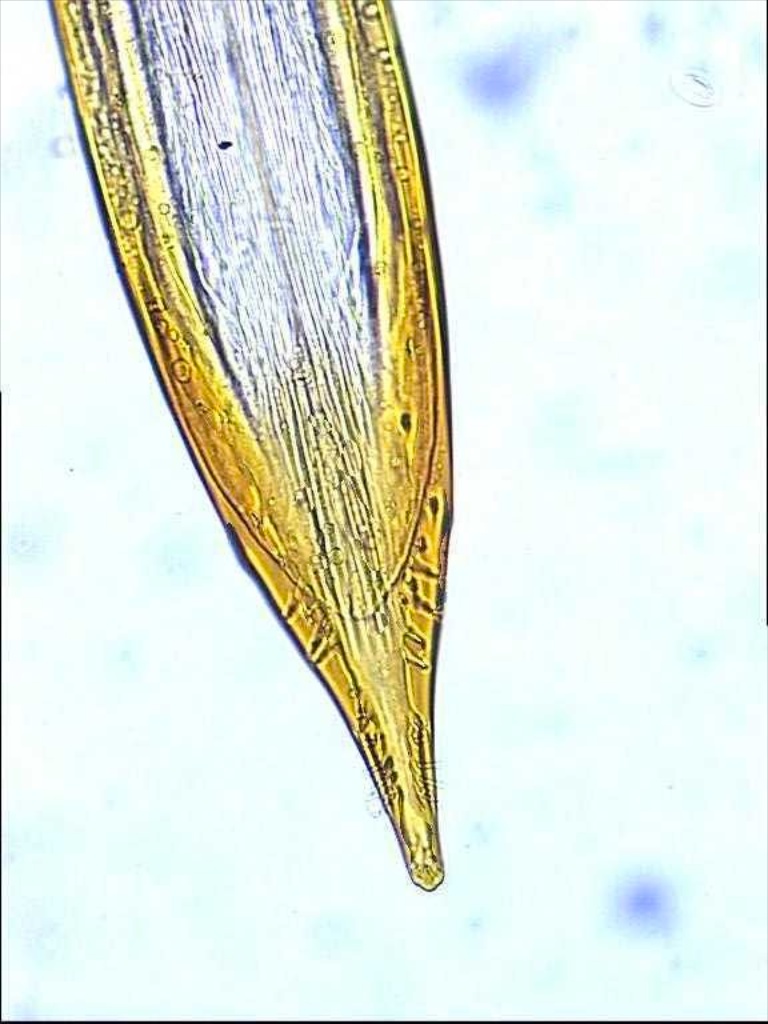

Supplement: Supplementary material 4 — Key to Capparimyia [file zookeys-428-097-s004.zip › SF4_ZooKeys_key to Capparimyia/key/SF4_ZooKeys_key to Capparimyia/Media/Images/113 aculeus apex dorsal (automontage (c) RMCA).jpg]

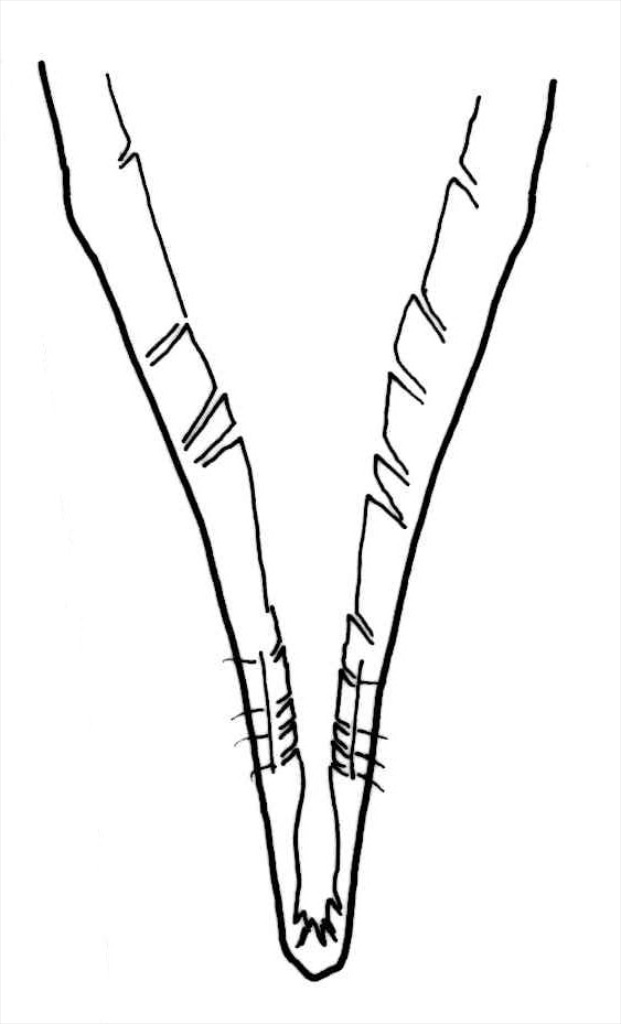

Supplement: Supplementary material 4 — Key to Capparimyia [file zookeys-428-097-s004.zip › SF4_ZooKeys_key to Capparimyia/key/SF4_ZooKeys_key to Capparimyia/Media/Images/113 aculeus apex dorsal (drawing (c) De Meyer & Freidberg).jpg]

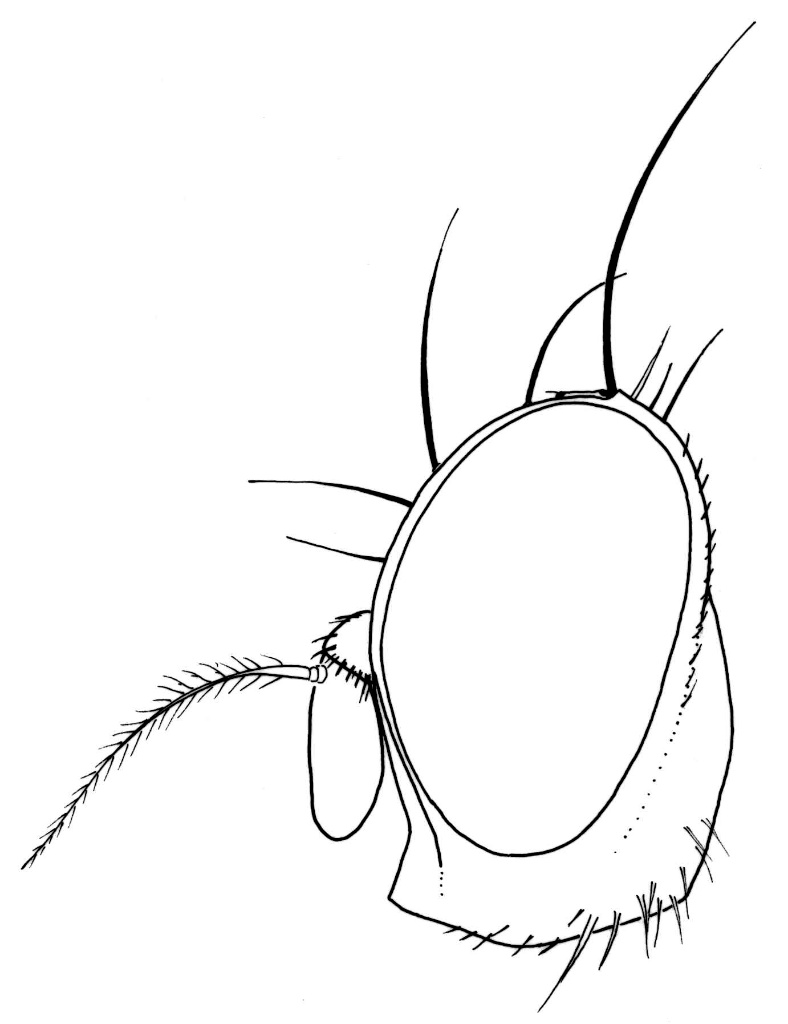

Supplement: Supplementary material 4 — Key to Capparimyia [file zookeys-428-097-s004.zip › SF4_ZooKeys_key to Capparimyia/key/SF4_ZooKeys_key to Capparimyia/Media/Images/113 head lateral (drawing (c) De Meyer & Freidberg).jpg]

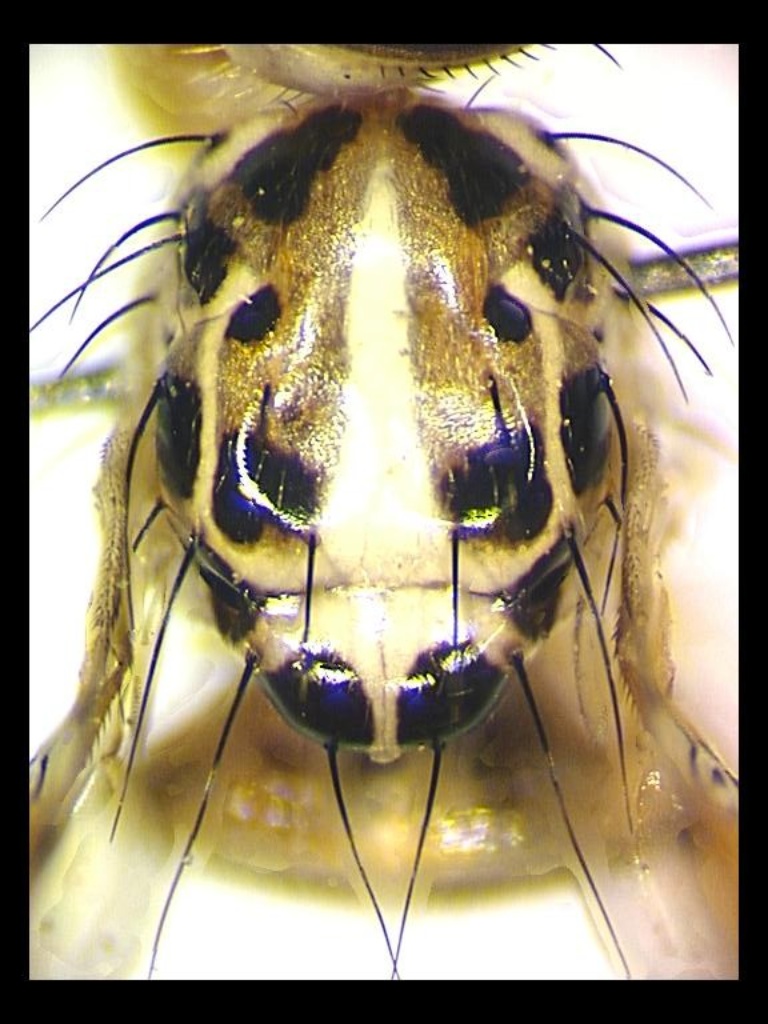

Supplement: Supplementary material 4 — Key to Capparimyia [file zookeys-428-097-s004.zip › SF4_ZooKeys_key to Capparimyia/key/SF4_ZooKeys_key to Capparimyia/Media/Images/113 mesonotum dorsal (automontage (c) RMCA).jpg]

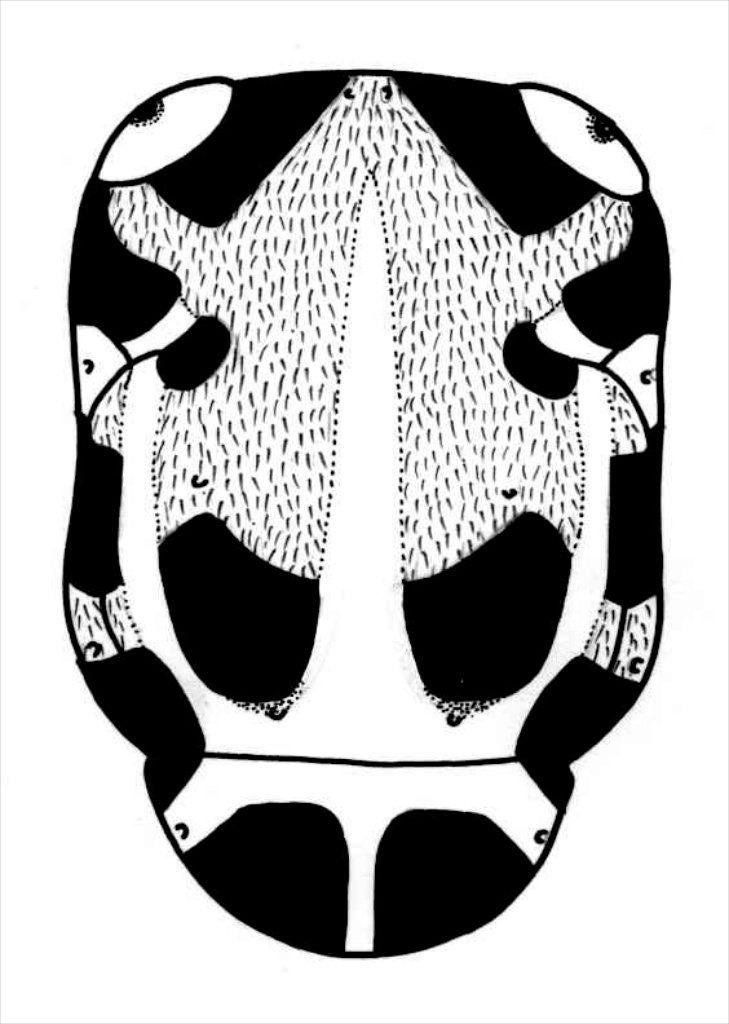

Supplement: Supplementary material 4 — Key to Capparimyia [file zookeys-428-097-s004.zip › SF4_ZooKeys_key to Capparimyia/key/SF4_ZooKeys_key to Capparimyia/Media/Images/113 mesonotum dorsal (drawing (c) De Meyer & Freidberg).jpg]

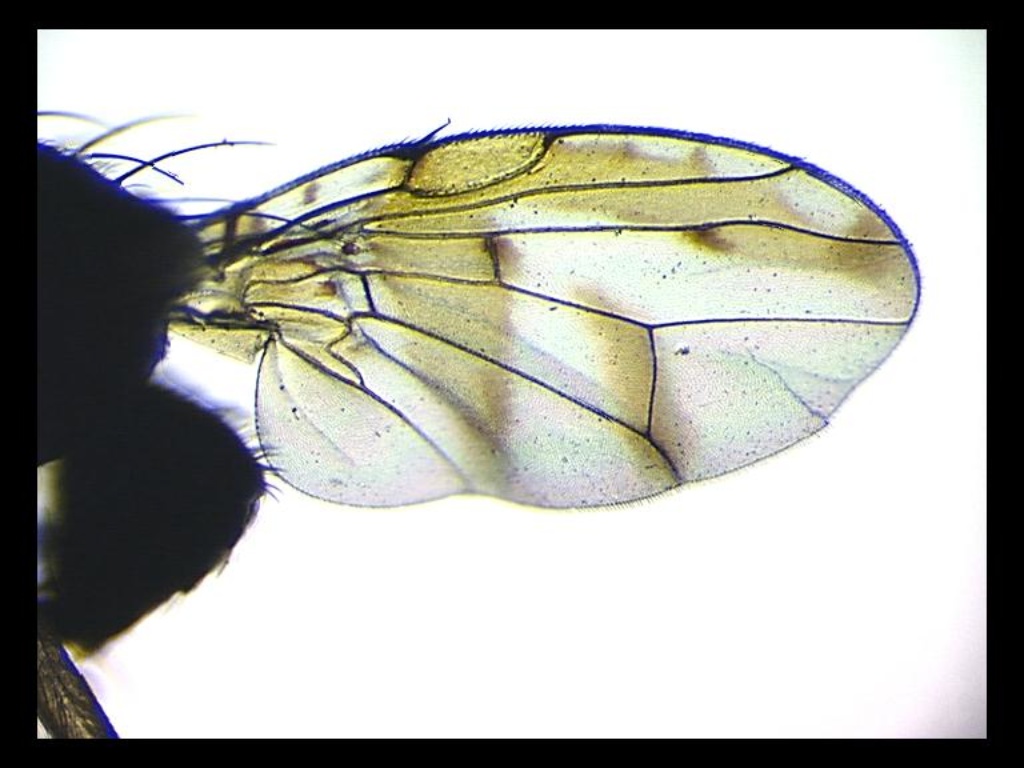

Supplement: Supplementary material 4 — Key to Capparimyia [file zookeys-428-097-s004.zip › SF4_ZooKeys_key to Capparimyia/key/SF4_ZooKeys_key to Capparimyia/Media/Images/113 wing dorsal (automontage (c) RMCA).jpg]

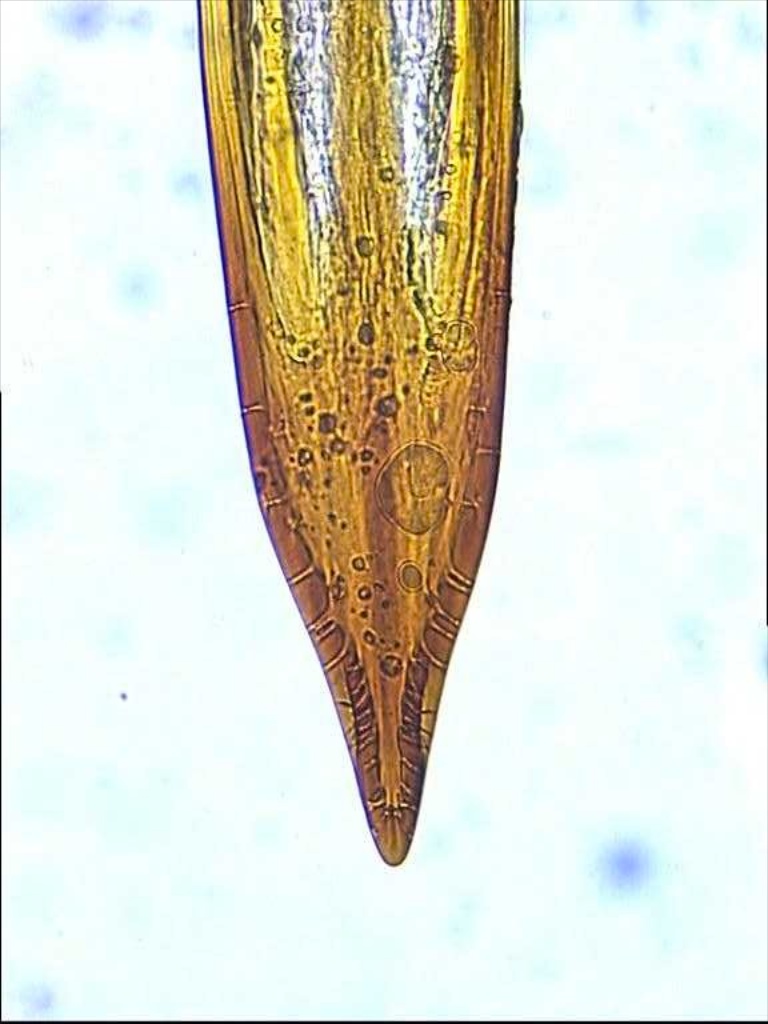

Supplement: Supplementary material 4 — Key to Capparimyia [file zookeys-428-097-s004.zip › SF4_ZooKeys_key to Capparimyia/key/SF4_ZooKeys_key to Capparimyia/Media/Images/114 aculeus apex dorsal (automontage (c) RMCA).jpg]

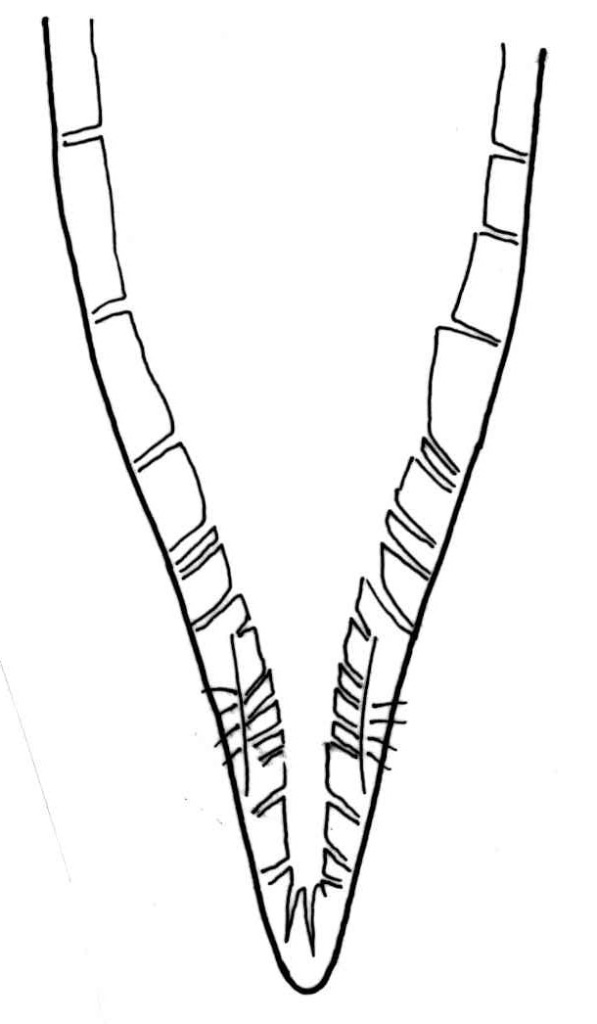

Supplement: Supplementary material 4 — Key to Capparimyia [file zookeys-428-097-s004.zip › SF4_ZooKeys_key to Capparimyia/key/SF4_ZooKeys_key to Capparimyia/Media/Images/114 aculeus apex dorsal (drawing (c) De Meyer & Freidberg).jpg]

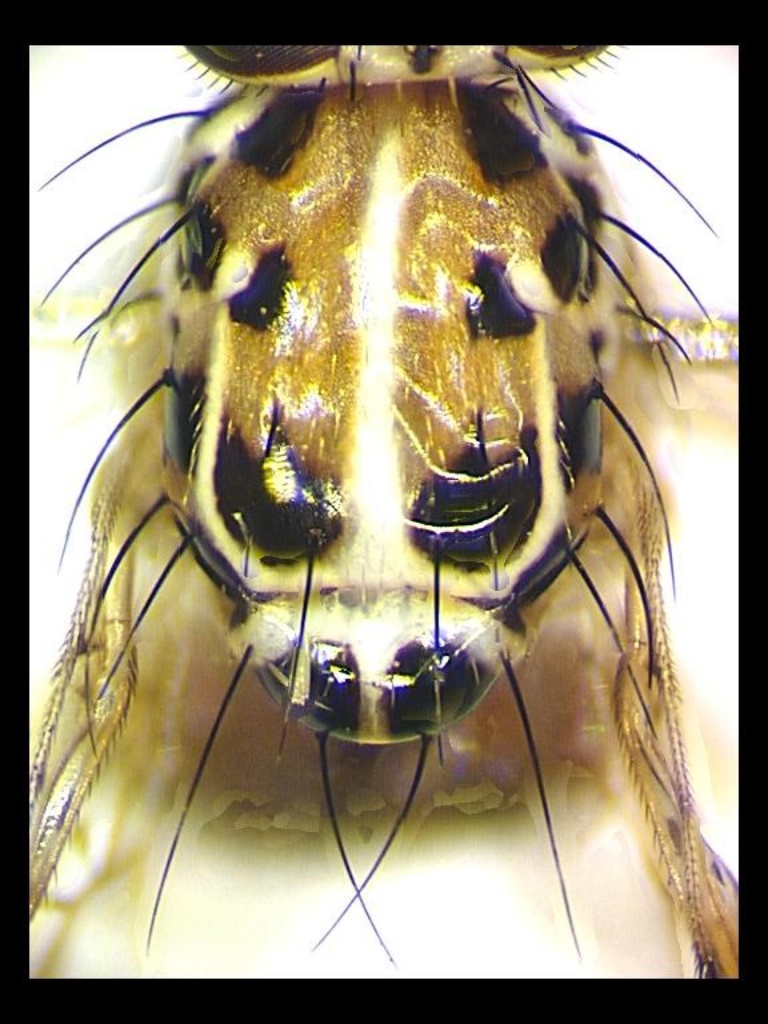

Supplement: Supplementary material 4 — Key to Capparimyia [file zookeys-428-097-s004.zip › SF4_ZooKeys_key to Capparimyia/key/SF4_ZooKeys_key to Capparimyia/Media/Images/114 mesonotum dorsal (automontage (c) RMCA).jpg]

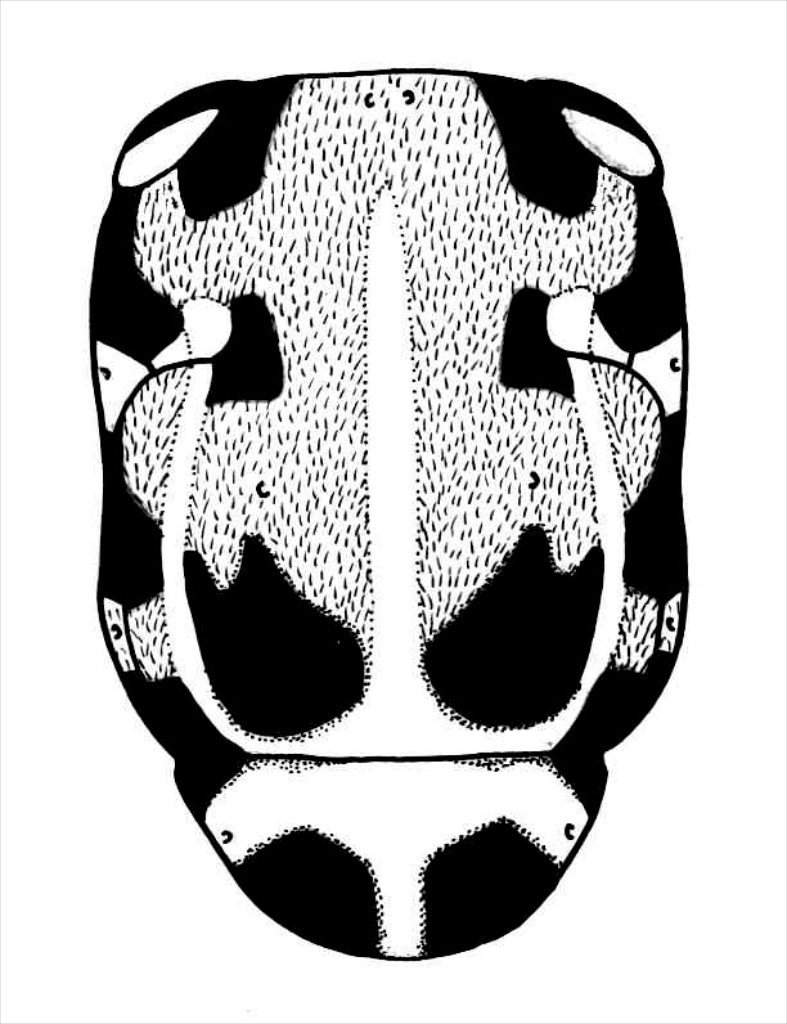

Supplement: Supplementary material 4 — Key to Capparimyia [file zookeys-428-097-s004.zip › SF4_ZooKeys_key to Capparimyia/key/SF4_ZooKeys_key to Capparimyia/Media/Images/114 mesonotum dorsal (drawing (c) De Meyer & Freidberg).jpg]

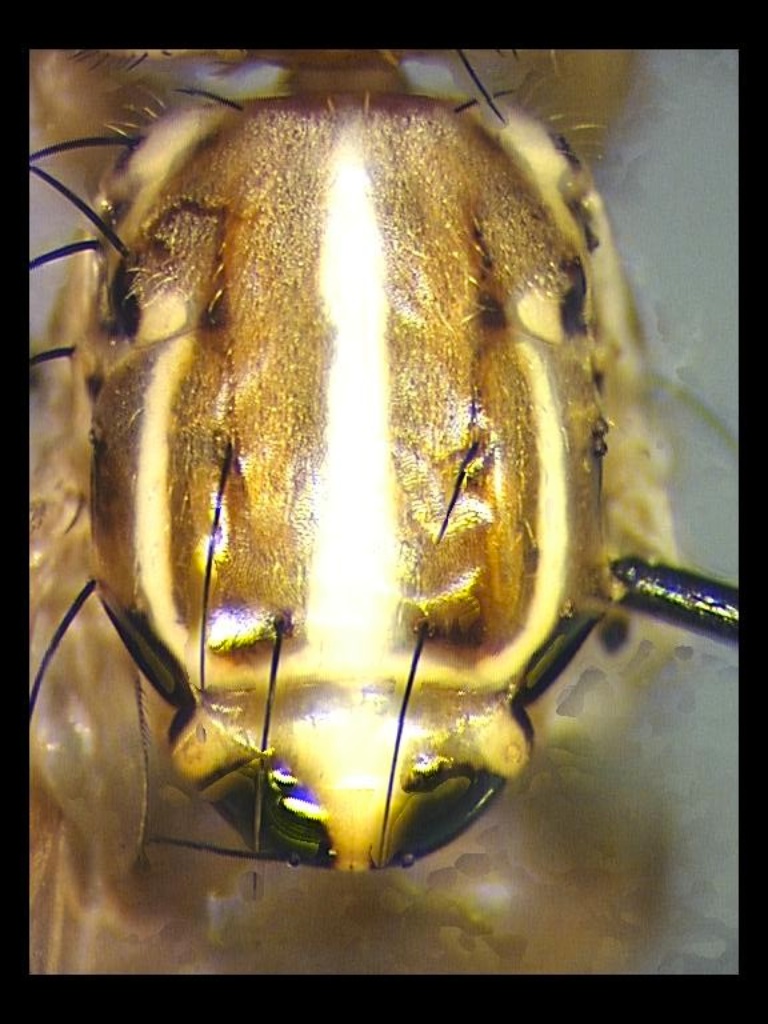

Supplement: Supplementary material 4 — Key to Capparimyia [file zookeys-428-097-s004.zip › SF4_ZooKeys_key to Capparimyia/key/SF4_ZooKeys_key to Capparimyia/Media/Images/114 mesonotum variant dorsal (automontage (c) RMCA).jpg]

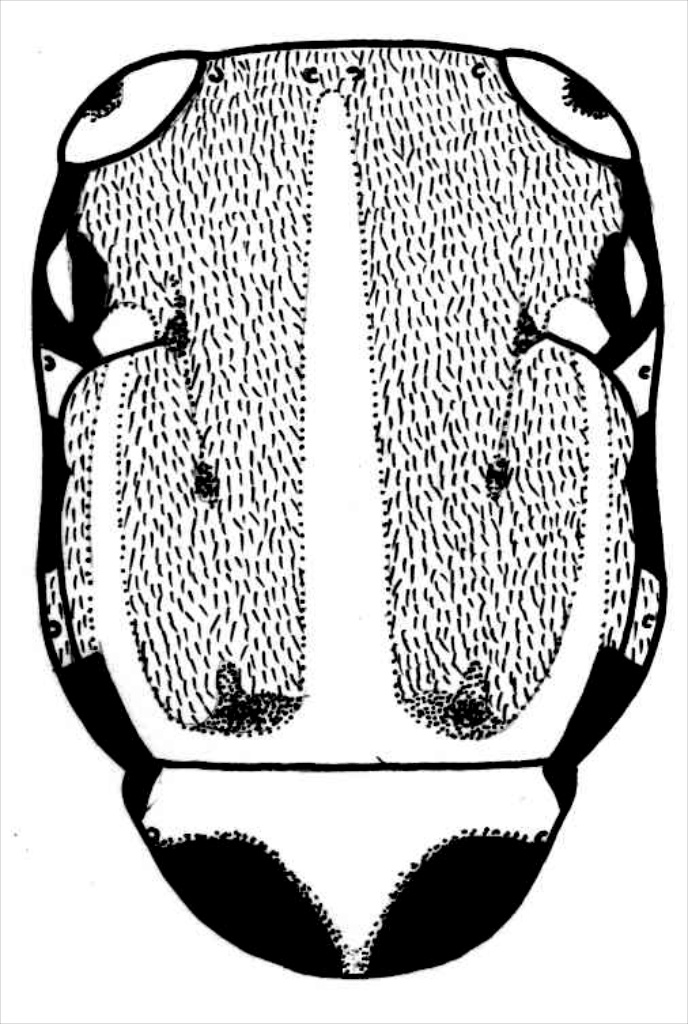

Supplement: Supplementary material 4 — Key to Capparimyia [file zookeys-428-097-s004.zip › SF4_ZooKeys_key to Capparimyia/key/SF4_ZooKeys_key to Capparimyia/Media/Images/114 mesonotum variant dorsal (drawing (c) De Meyer & Freidberg).jpg]

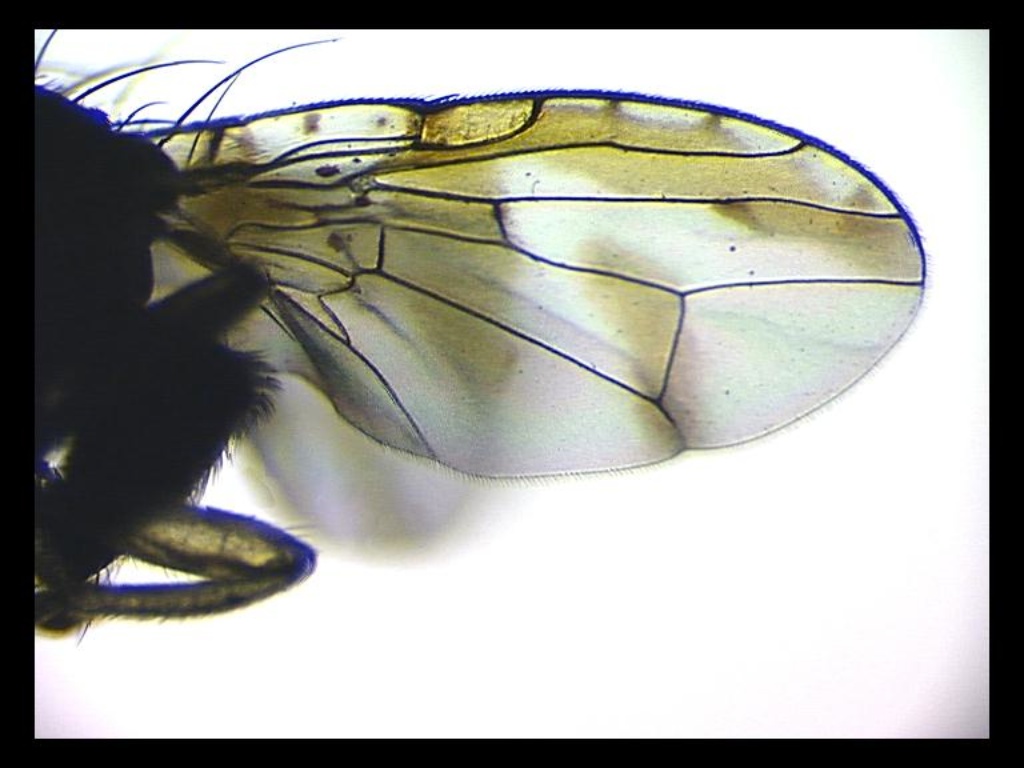

Supplement: Supplementary material 4 — Key to Capparimyia [file zookeys-428-097-s004.zip › SF4_ZooKeys_key to Capparimyia/key/SF4_ZooKeys_key to Capparimyia/Media/Images/114 wing dorsal (automontage (c) RMCA).jpg]

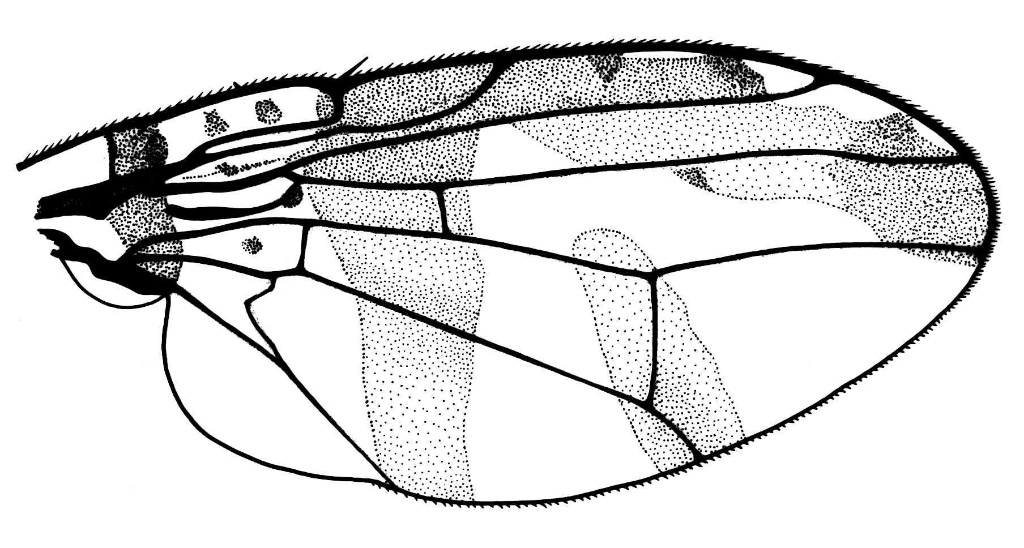

Supplement: Supplementary material 4 — Key to Capparimyia [file zookeys-428-097-s004.zip › SF4_ZooKeys_key to Capparimyia/key/SF4_ZooKeys_key to Capparimyia/Media/Images/114 wing dorsal (drawing (c) De Meyer & Freidberg).jpg]

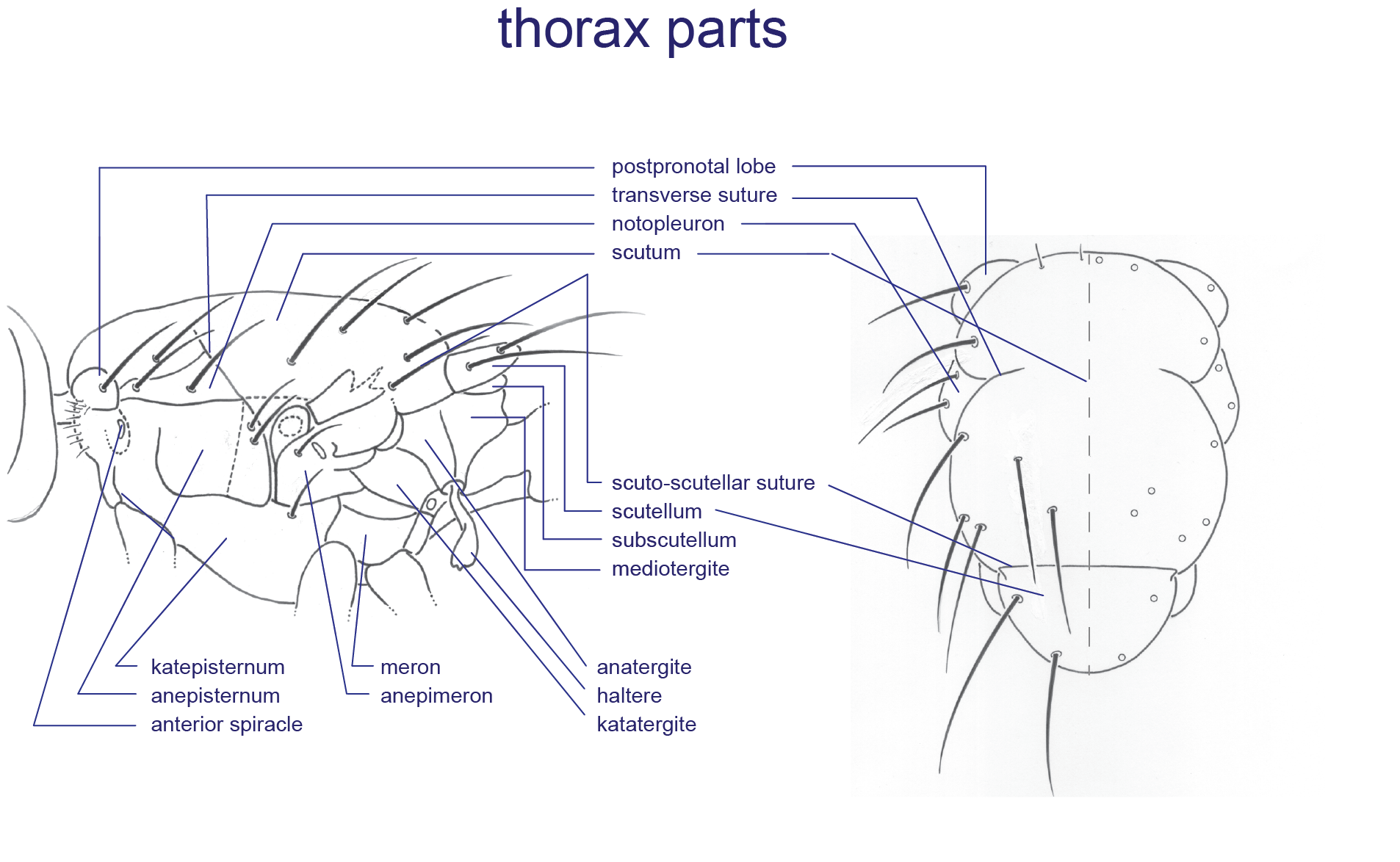

Supplement: Supplementary material 4 — Key to Capparimyia [file zookeys-428-097-s004.zip › SF4_ZooKeys_key to Capparimyia/key/SF4_ZooKeys_key to Capparimyia/Media/Images/char10_thorax_parts.png]

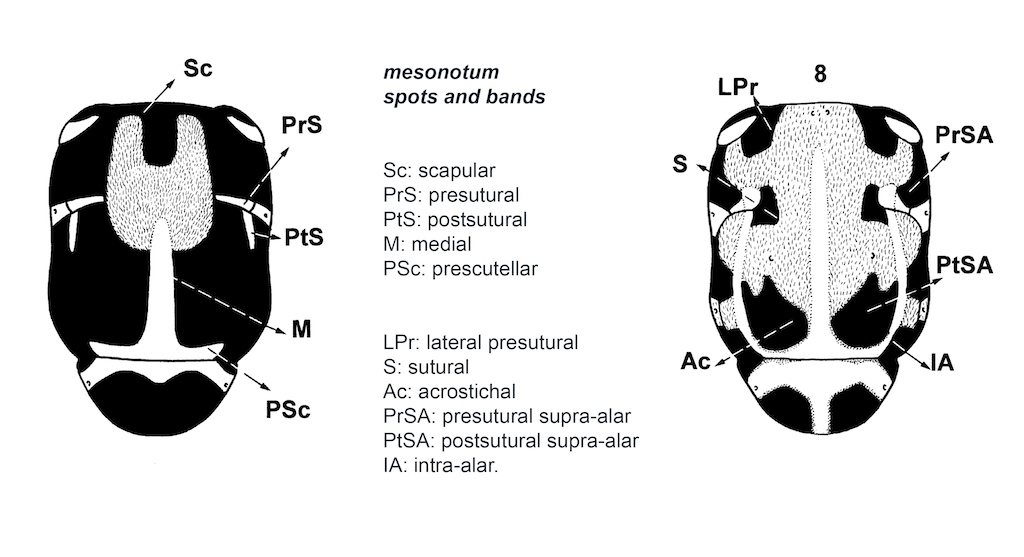

Supplement: Supplementary material 4 — Key to Capparimyia [file zookeys-428-097-s004.zip › SF4_ZooKeys_key to Capparimyia/key/SF4_ZooKeys_key to Capparimyia/Media/Images/char13_Capparimya_mesonotum spots and bands.jpg]

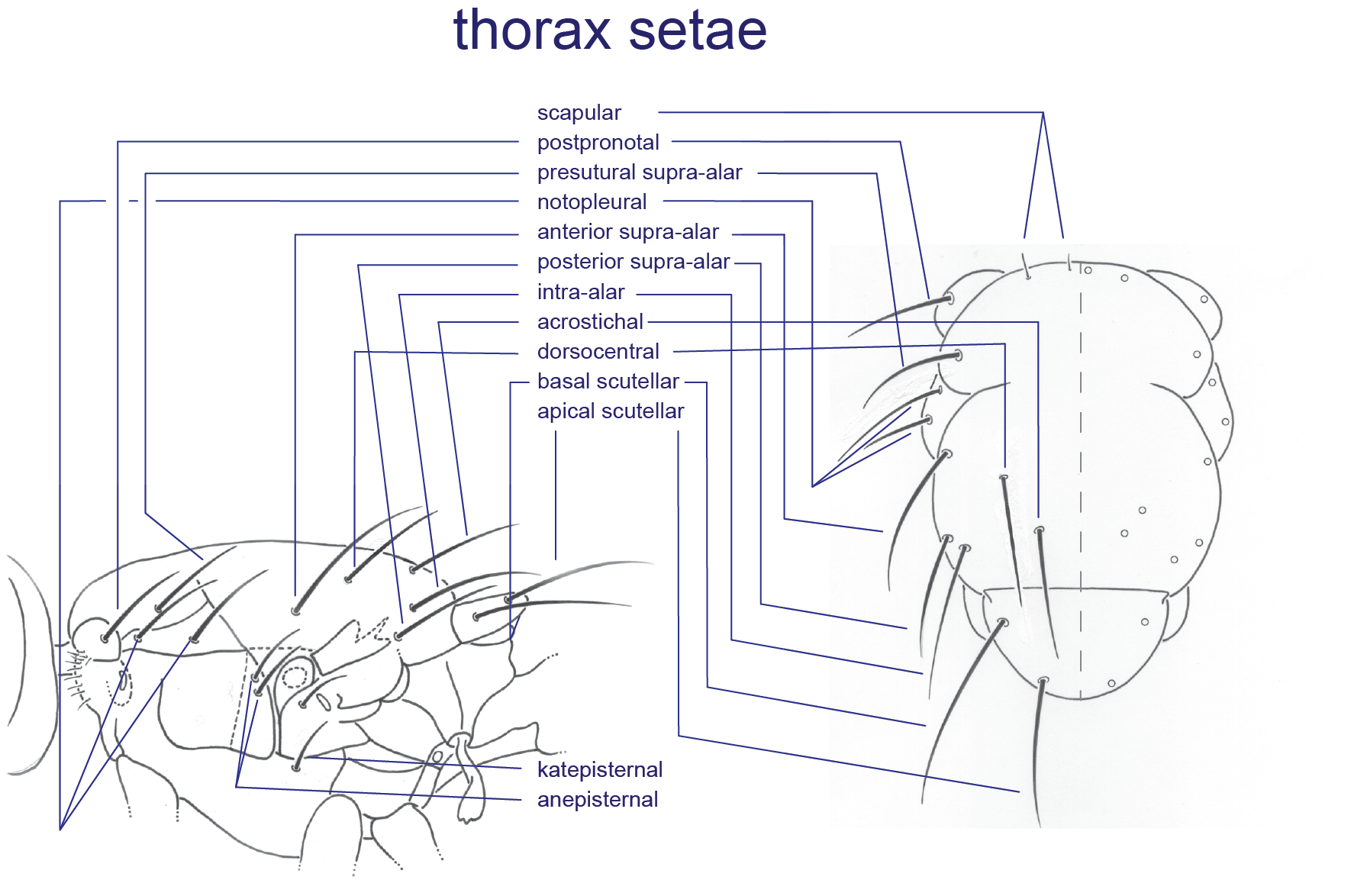

Supplement: Supplementary material 4 — Key to Capparimyia [file zookeys-428-097-s004.zip › SF4_ZooKeys_key to Capparimyia/key/SF4_ZooKeys_key to Capparimyia/Media/Images/char14_thorax_setae.png]

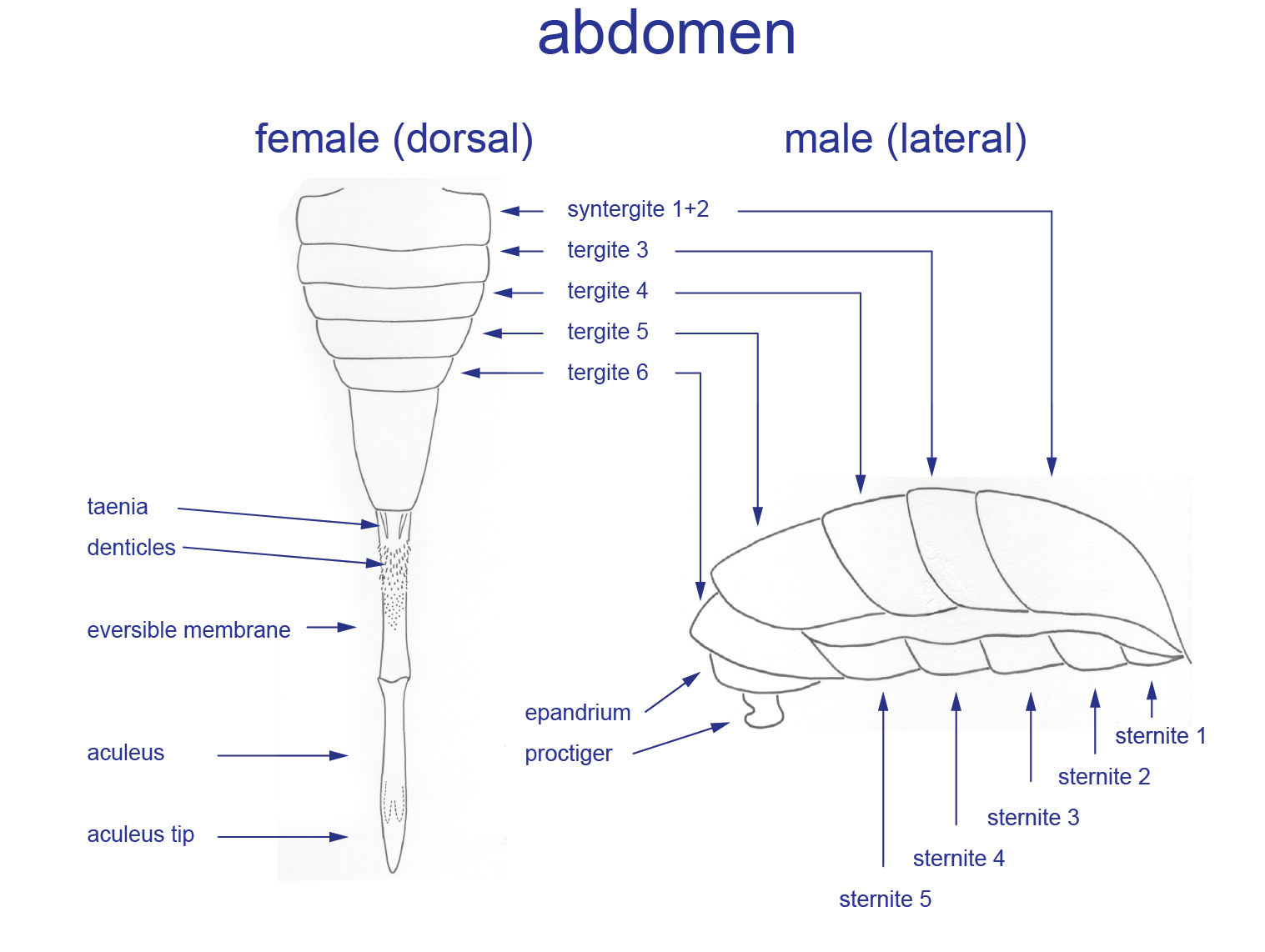

Supplement: Supplementary material 4 — Key to Capparimyia [file zookeys-428-097-s004.zip › SF4_ZooKeys_key to Capparimyia/key/SF4_ZooKeys_key to Capparimyia/Media/Images/char15_abdomen_M_F.png]

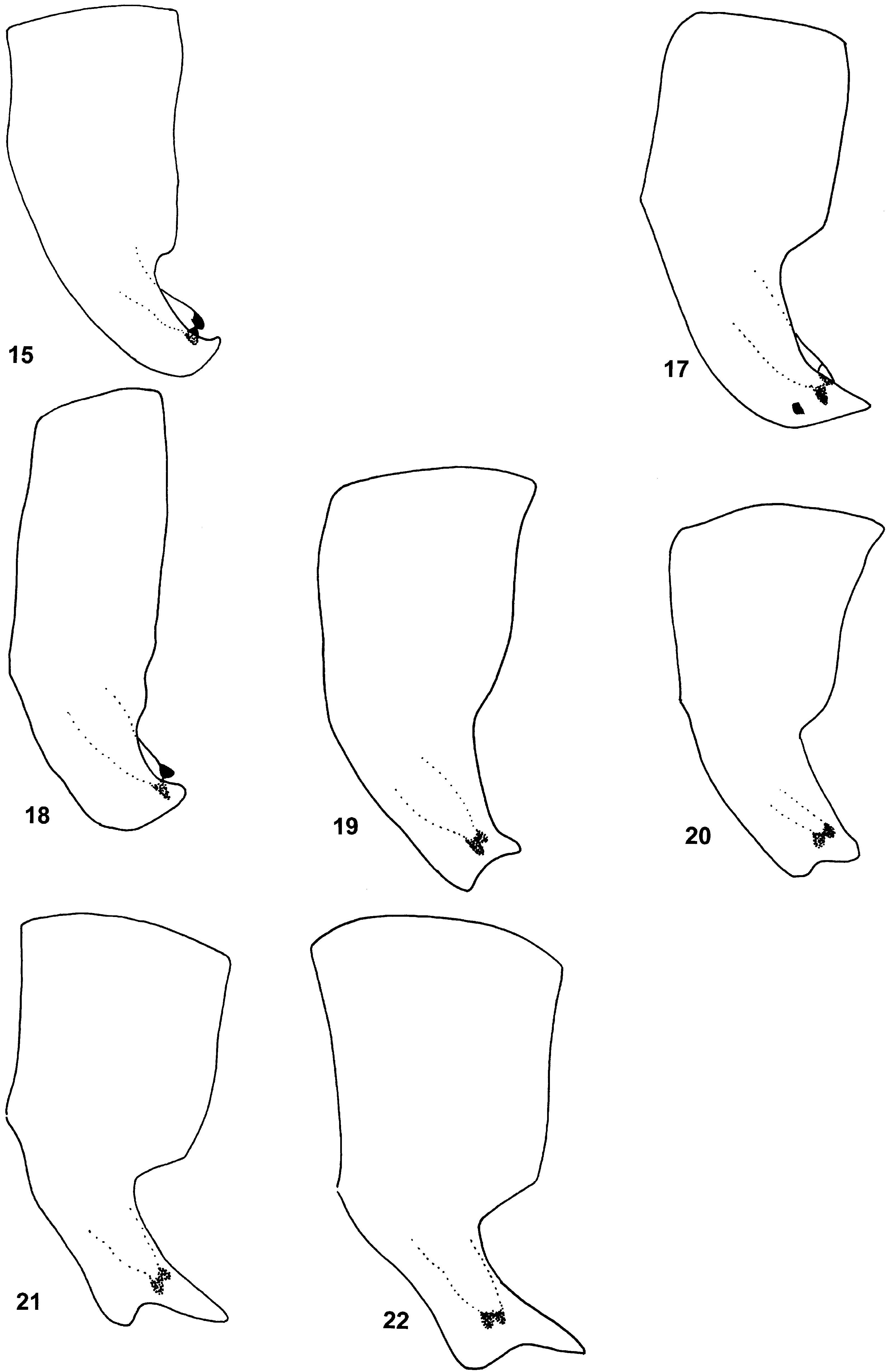

Supplement: Supplementary material 4 — Key to Capparimyia [file zookeys-428-097-s004.zip › SF4_ZooKeys_key to Capparimyia/key/SF4_ZooKeys_key to Capparimyia/Media/Images/char18_equal.jpg]

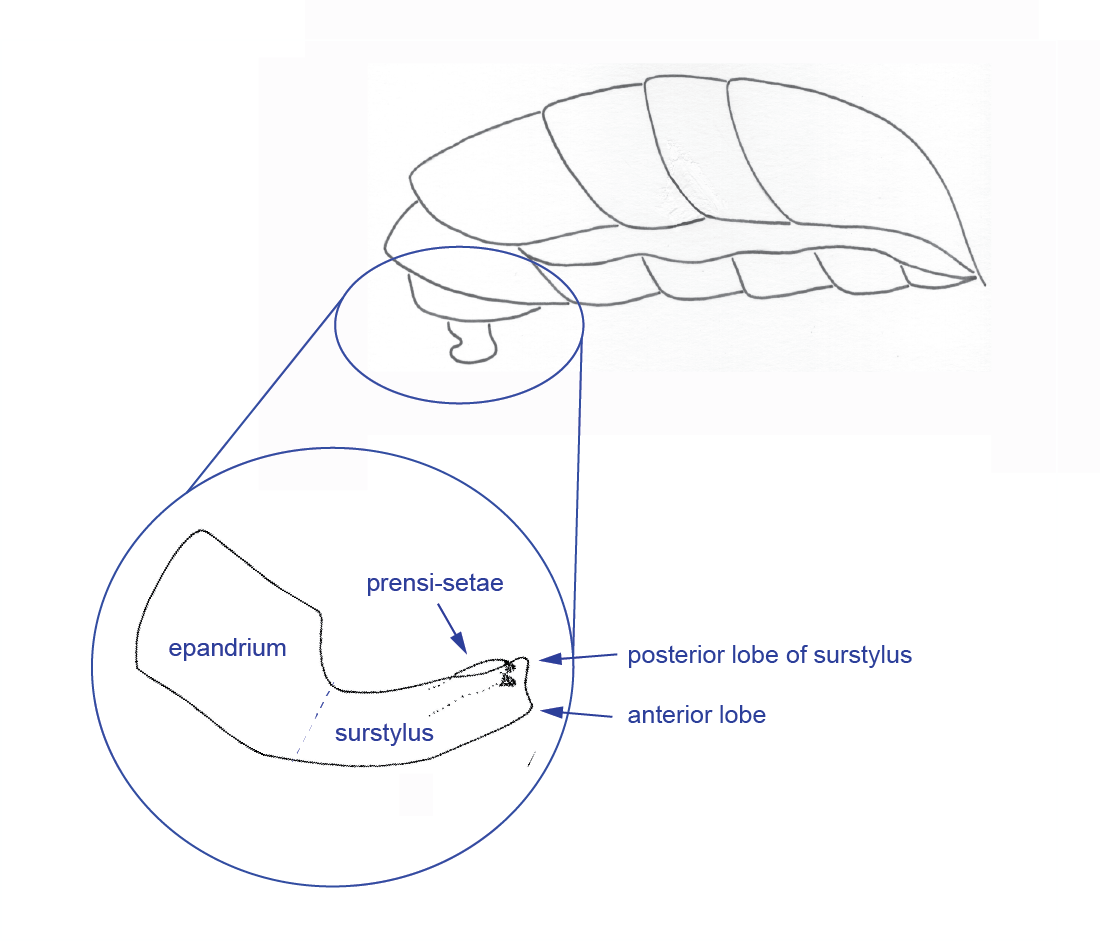

Supplement: Supplementary material 4 — Key to Capparimyia [file zookeys-428-097-s004.zip › SF4_ZooKeys_key to Capparimyia/key/SF4_ZooKeys_key to Capparimyia/Media/Images/char18_general.png]

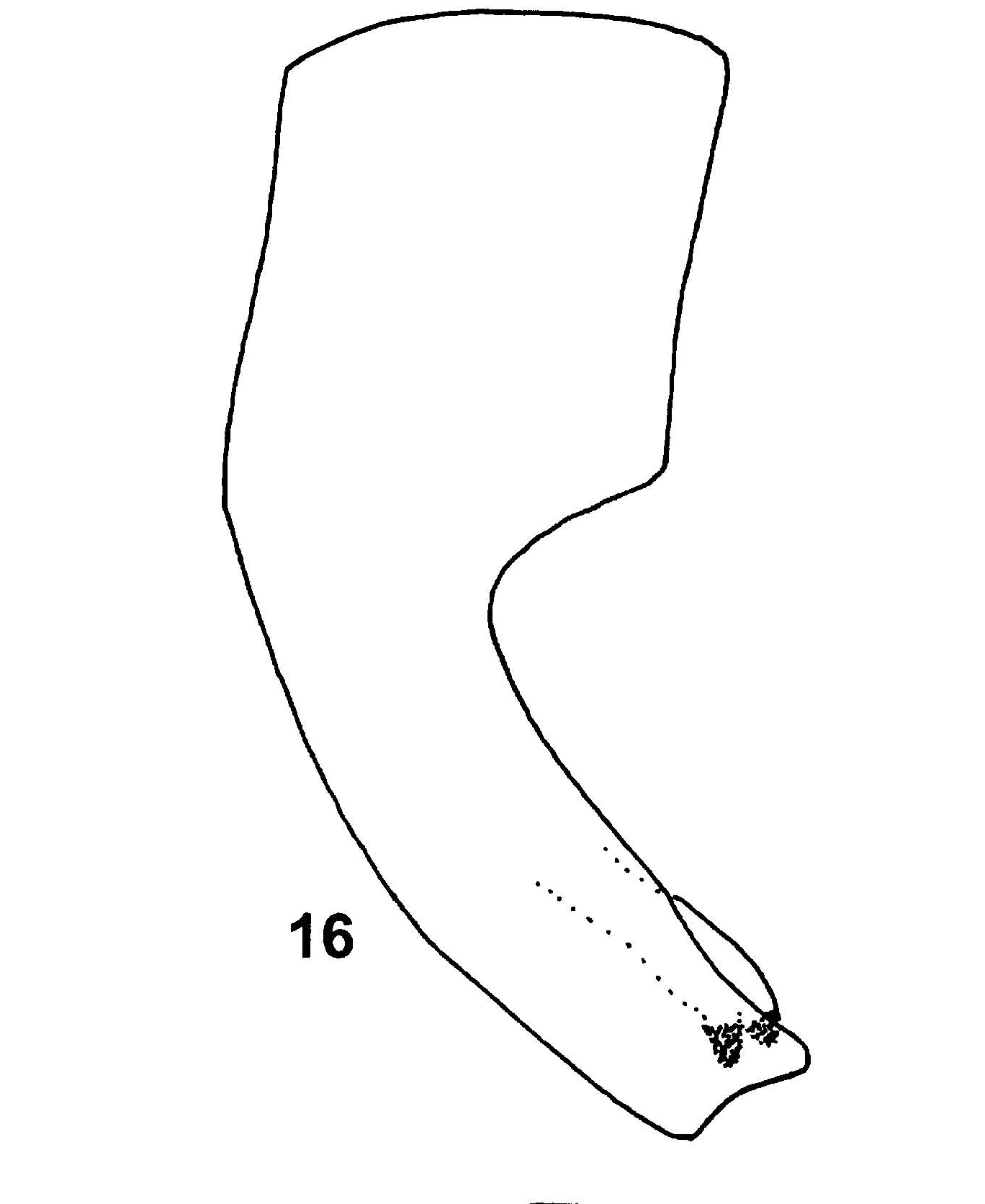

Supplement: Supplementary material 4 — Key to Capparimyia [file zookeys-428-097-s004.zip › SF4_ZooKeys_key to Capparimyia/key/SF4_ZooKeys_key to Capparimyia/Media/Images/char18_longer.jpg]

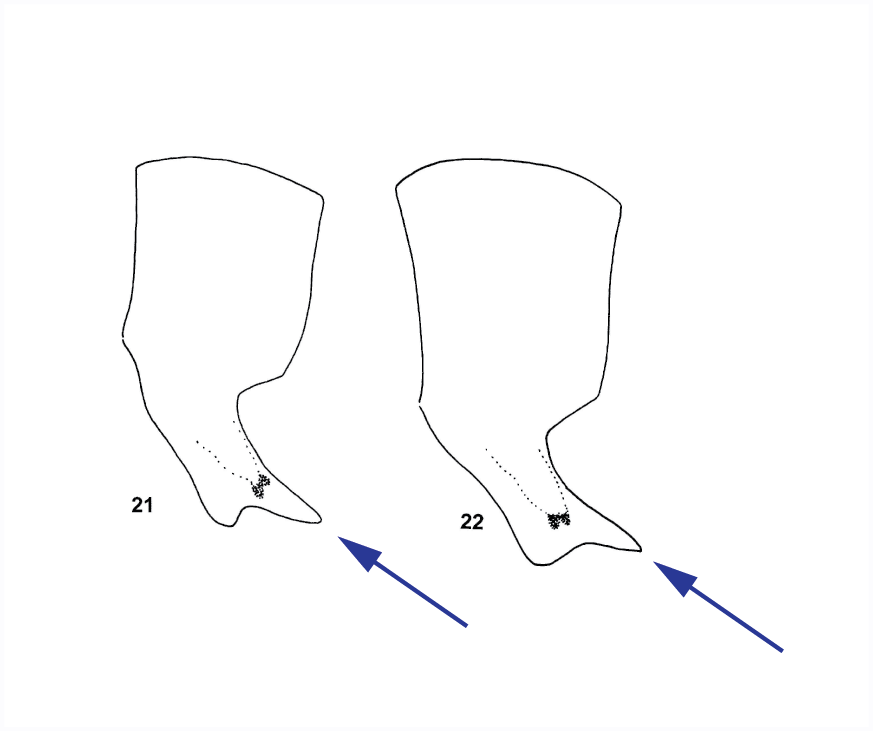

Supplement: Supplementary material 4 — Key to Capparimyia [file zookeys-428-097-s004.zip › SF4_ZooKeys_key to Capparimyia/key/SF4_ZooKeys_key to Capparimyia/Media/Images/char19_extended.png]

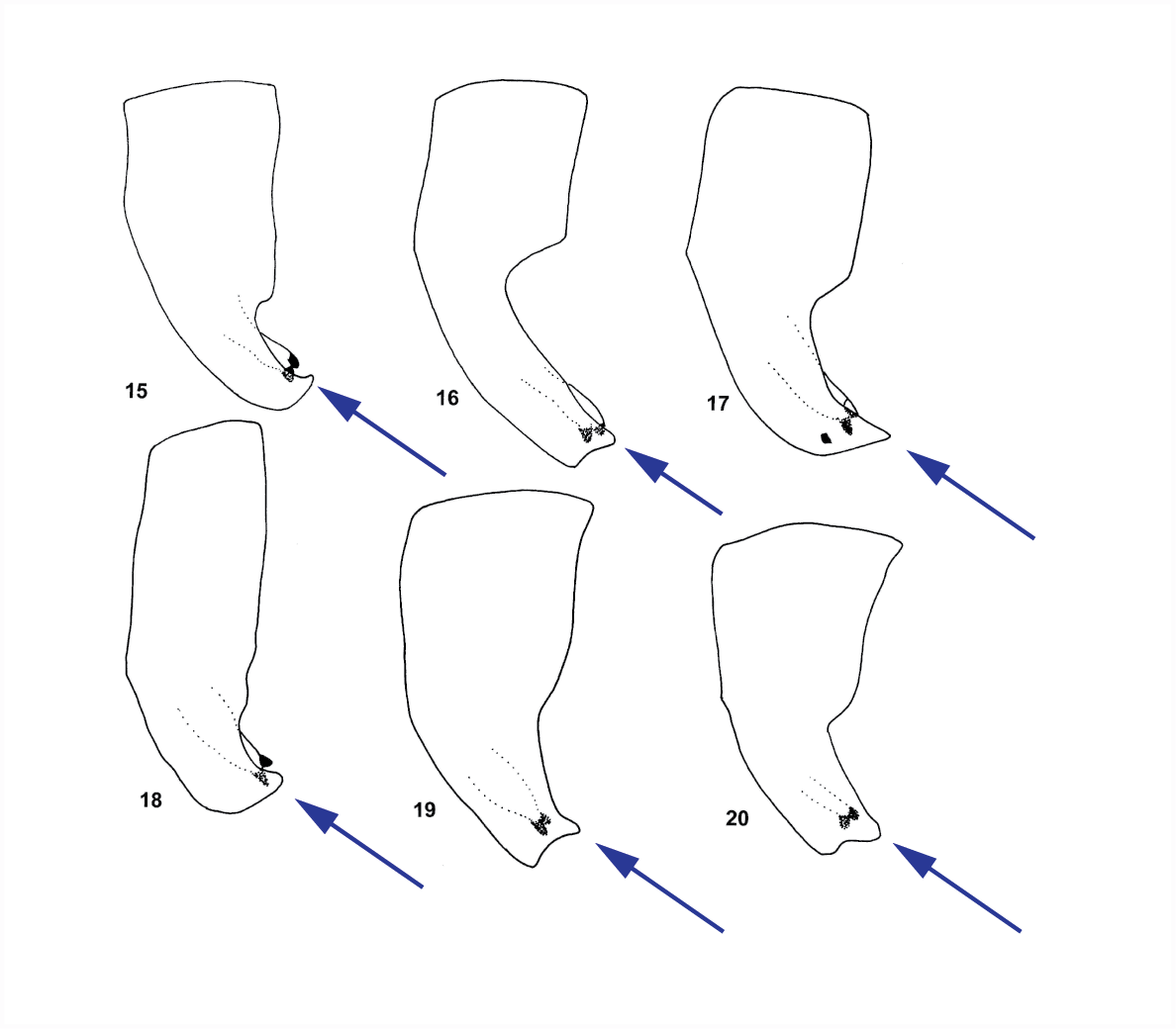

Supplement: Supplementary material 4 — Key to Capparimyia [file zookeys-428-097-s004.zip › SF4_ZooKeys_key to Capparimyia/key/SF4_ZooKeys_key to Capparimyia/Media/Images/char19_reduced.png]

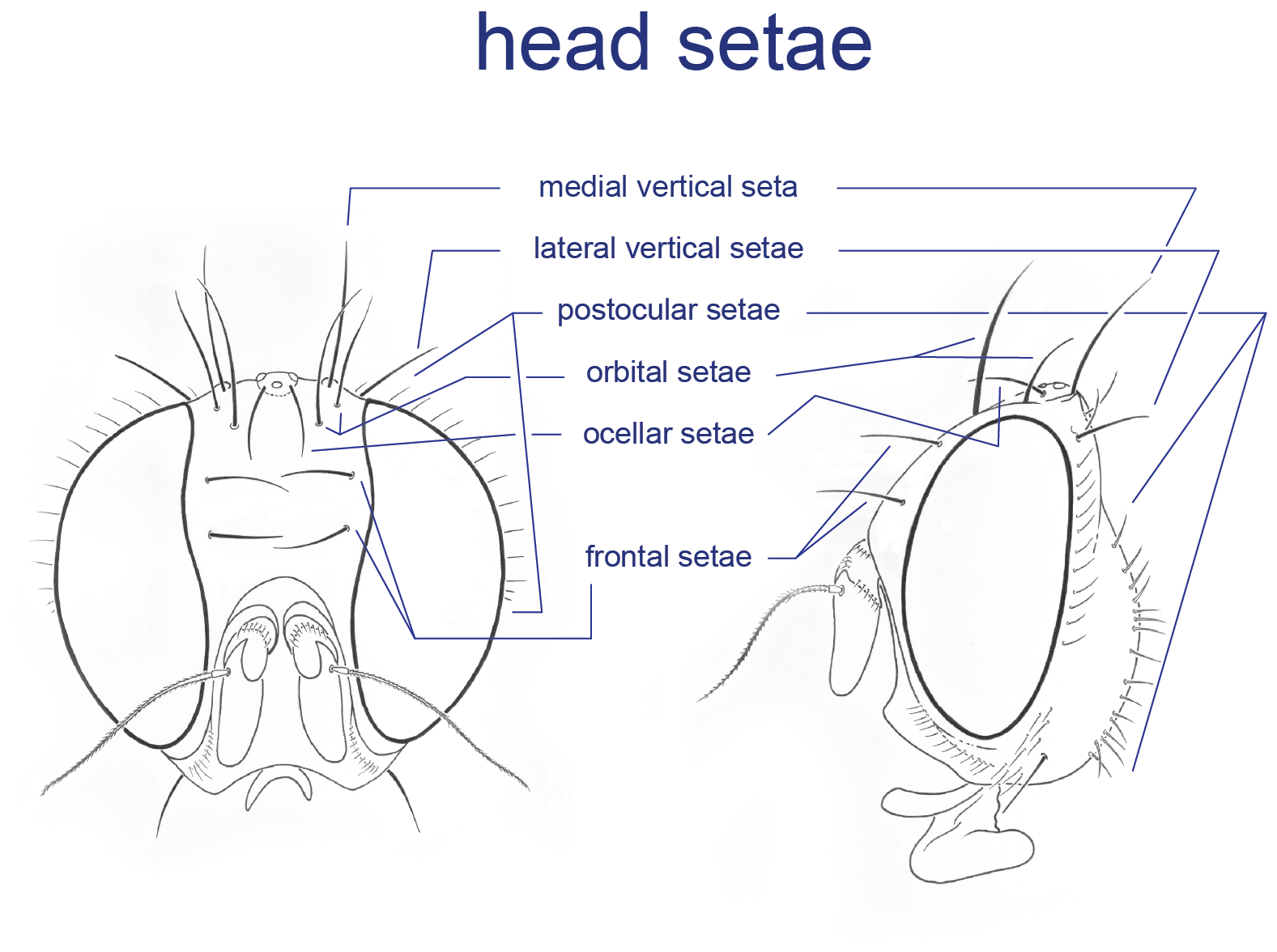

Supplement: Supplementary material 4 — Key to Capparimyia [file zookeys-428-097-s004.zip › SF4_ZooKeys_key to Capparimyia/key/SF4_ZooKeys_key to Capparimyia/Media/Images/char2_head_setae.png]

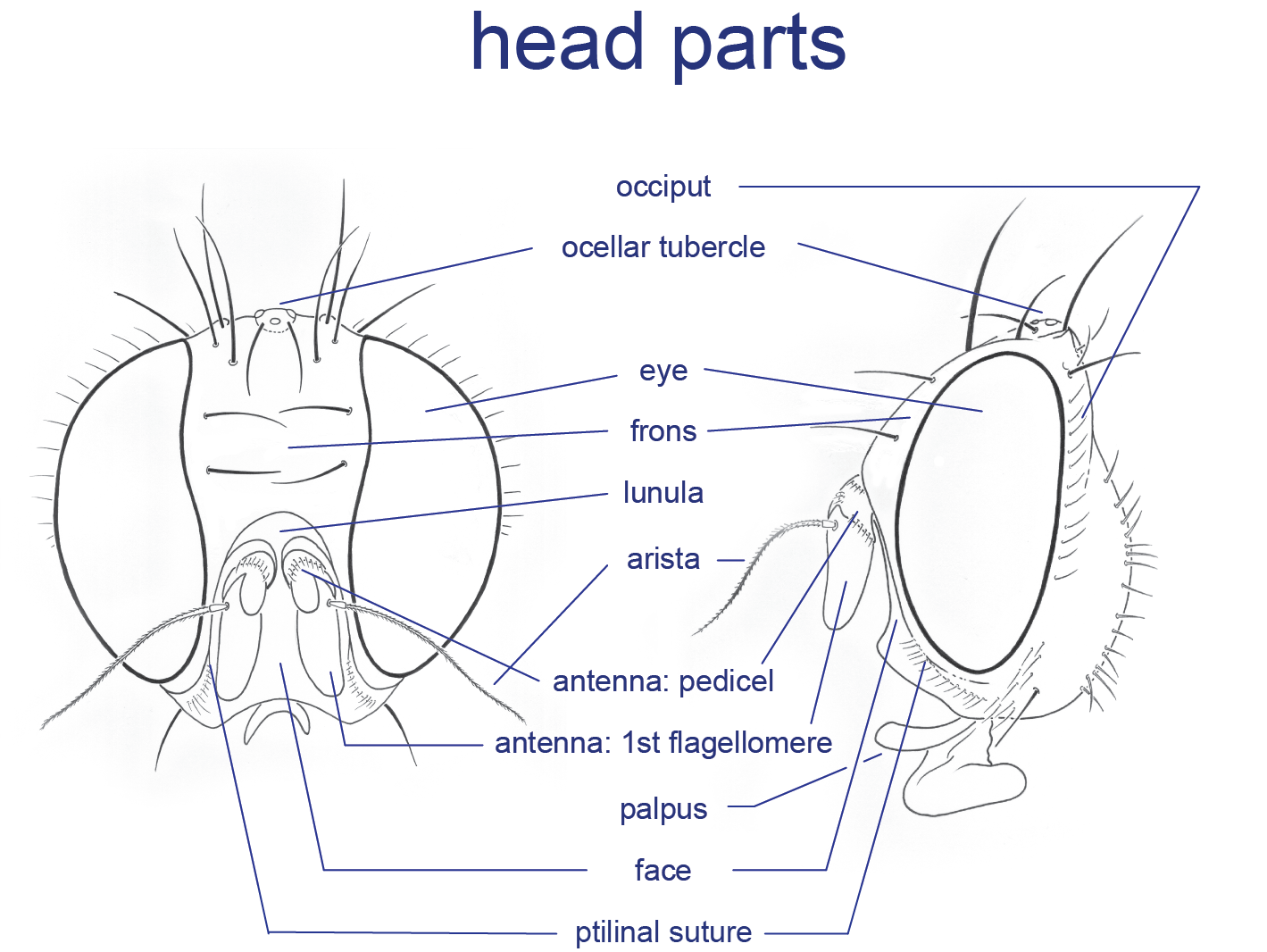

Supplement: Supplementary material 4 — Key to Capparimyia [file zookeys-428-097-s004.zip › SF4_ZooKeys_key to Capparimyia/key/SF4_ZooKeys_key to Capparimyia/Media/Images/char3_head_parts.png]

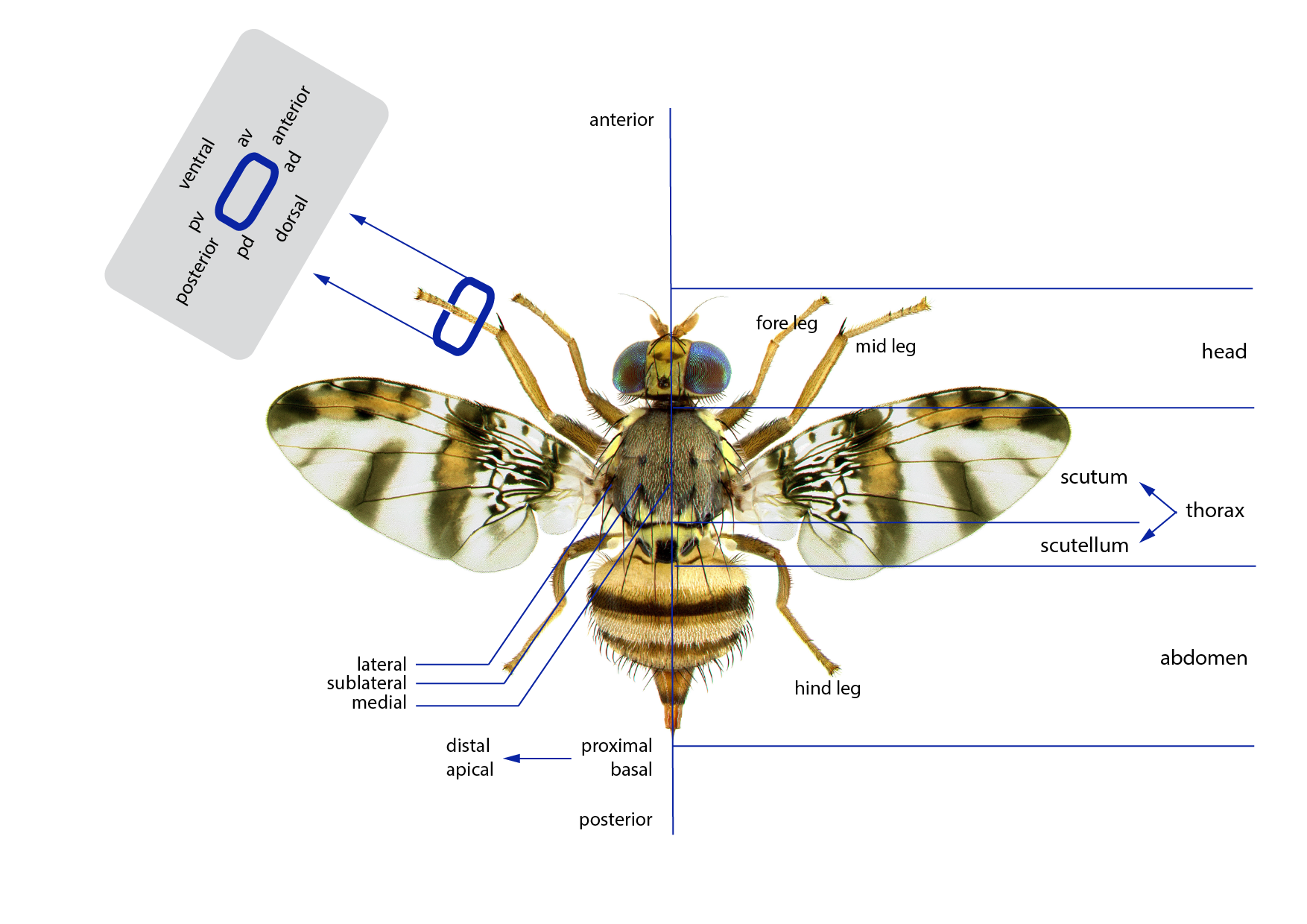

Supplement: Supplementary material 4 — Key to Capparimyia [file zookeys-428-097-s004.zip › SF4_ZooKeys_key to Capparimyia/key/SF4_ZooKeys_key to Capparimyia/Media/Images/general_abdomen_orientation.png]

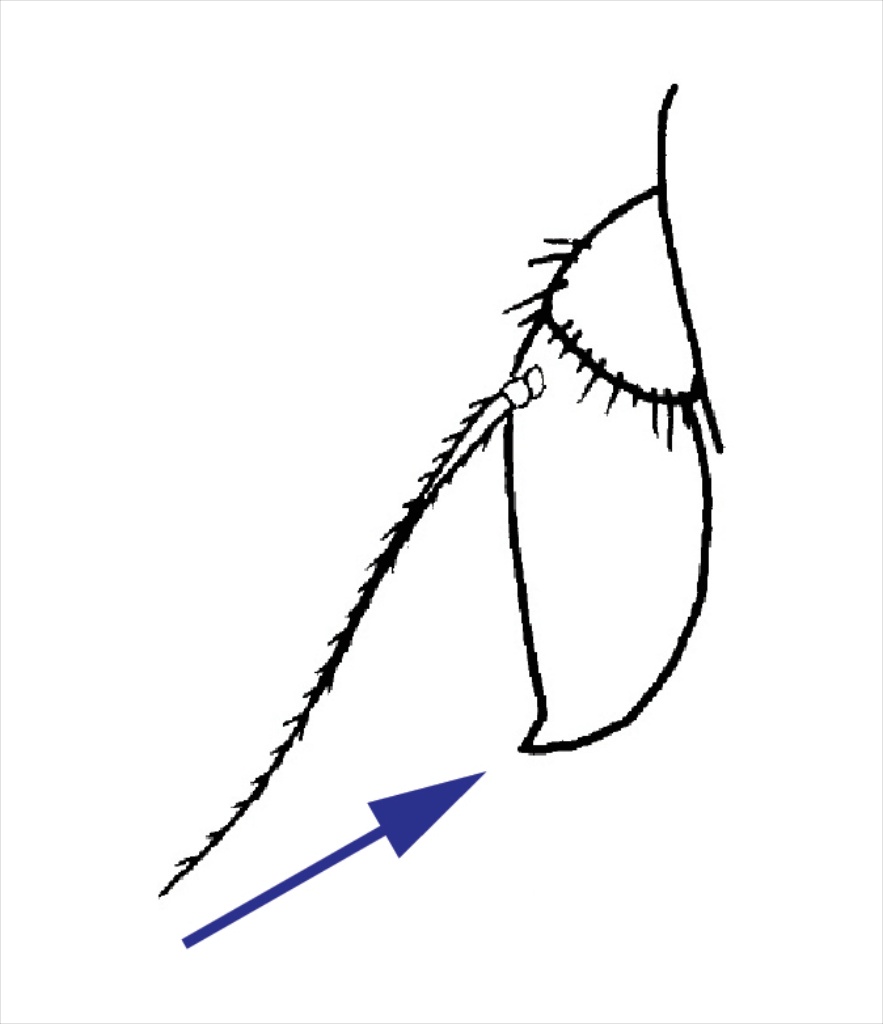

Supplement: Supplementary material 4 — Key to Capparimyia [file zookeys-428-097-s004.zip › SF4_ZooKeys_key to Capparimyia/key/SF4_ZooKeys_key to Capparimyia/Media/Images/head_1stflagellomere_pointed.jpg]

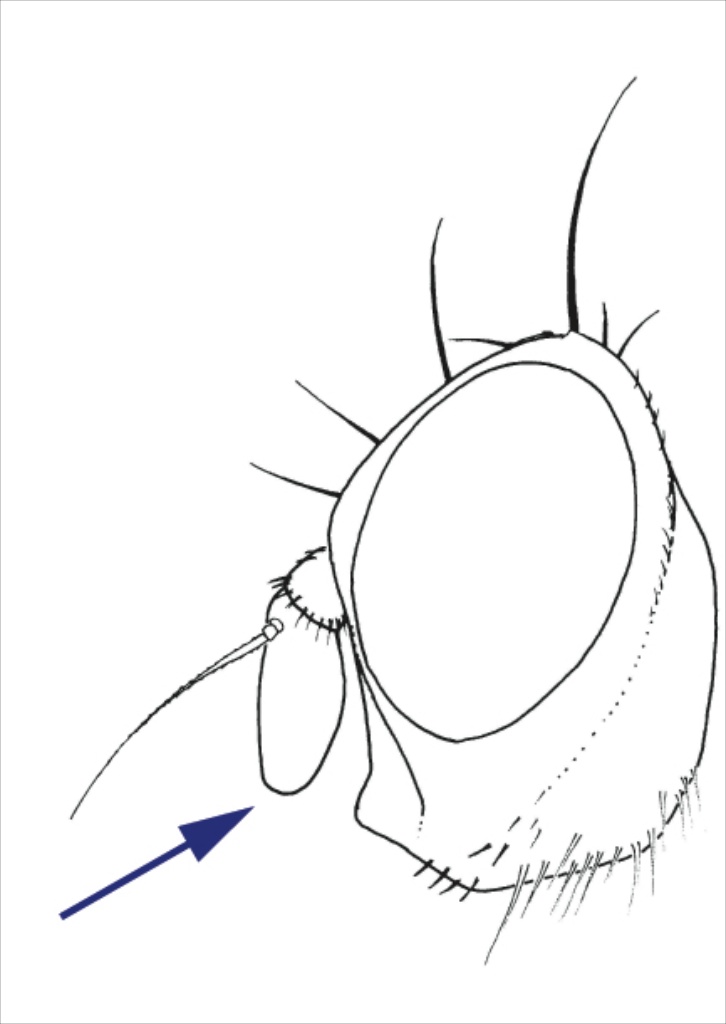

Supplement: Supplementary material 4 — Key to Capparimyia [file zookeys-428-097-s004.zip › SF4_ZooKeys_key to Capparimyia/key/SF4_ZooKeys_key to Capparimyia/Media/Images/head_1stflagellomere_rounded.jpg]

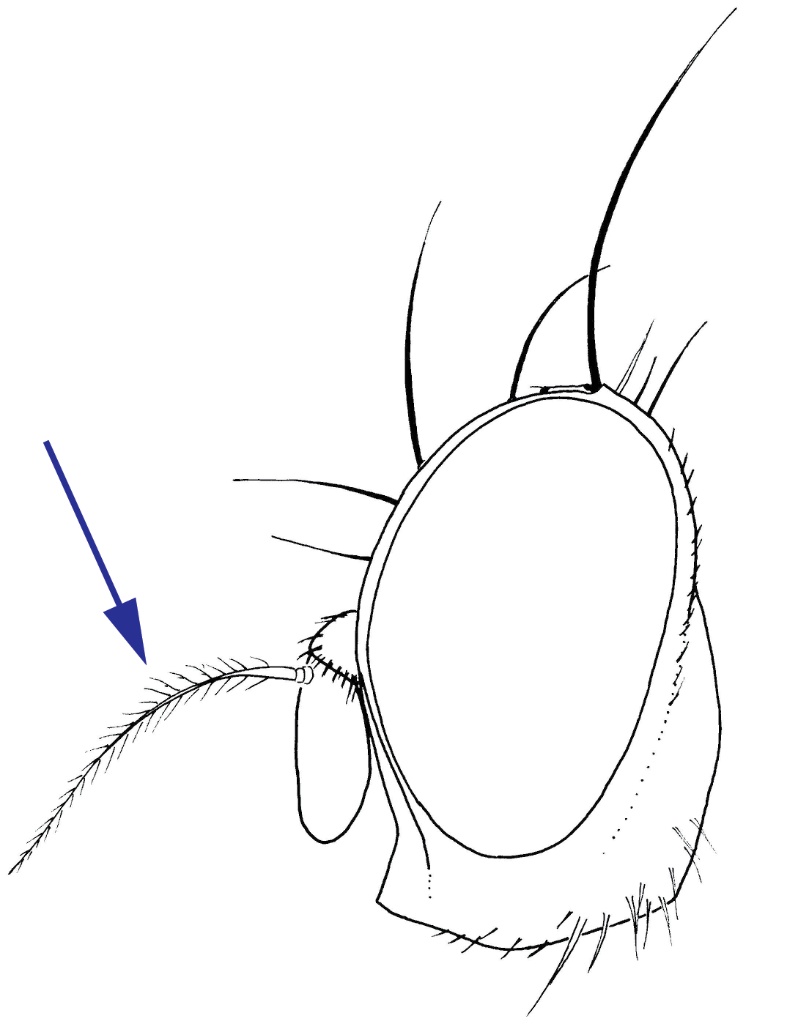

Supplement: Supplementary material 4 — Key to Capparimyia [file zookeys-428-097-s004.zip › SF4_ZooKeys_key to Capparimyia/key/SF4_ZooKeys_key to Capparimyia/Media/Images/head_arista_plumose.jpg]

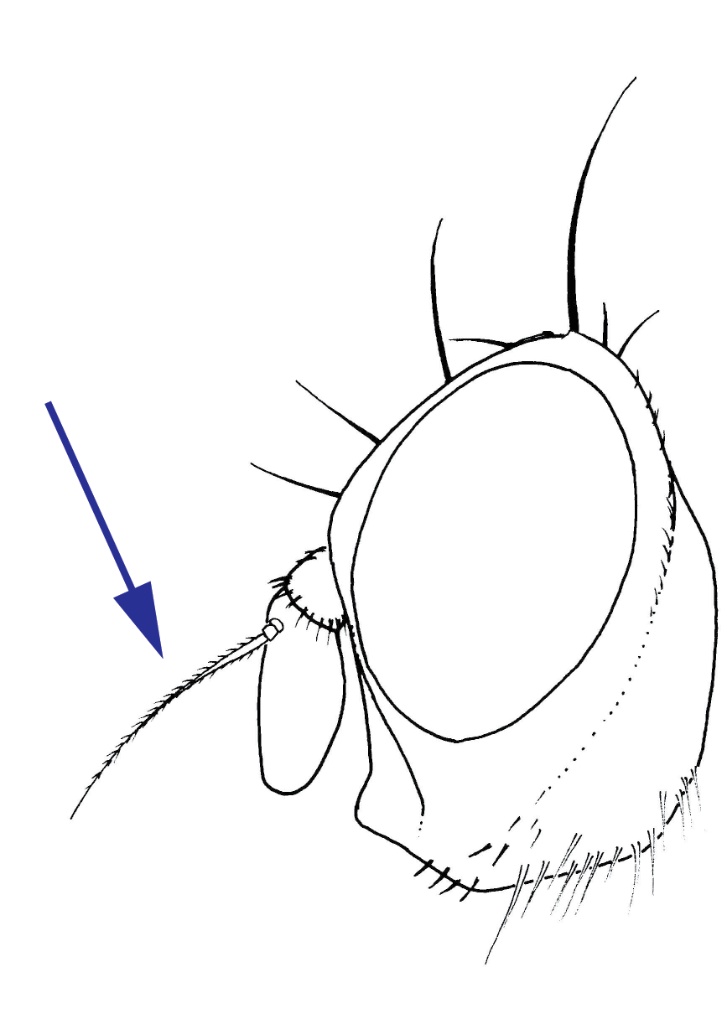

Supplement: Supplementary material 4 — Key to Capparimyia [file zookeys-428-097-s004.zip › SF4_ZooKeys_key to Capparimyia/key/SF4_ZooKeys_key to Capparimyia/Media/Images/head_arista_pubescent.jpg]

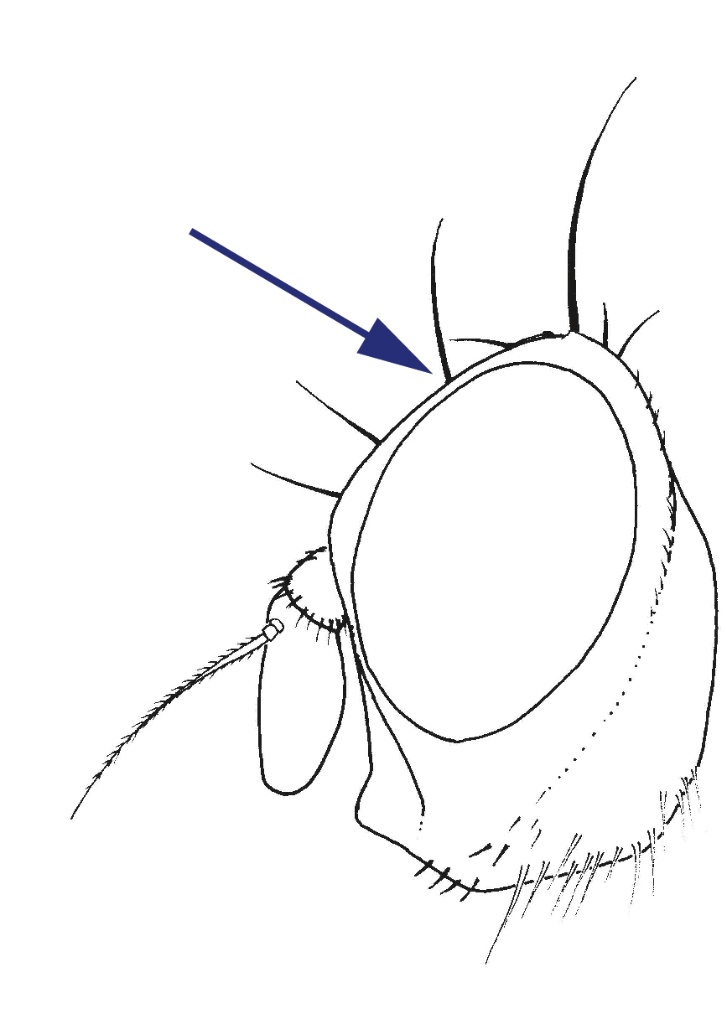

Supplement: Supplementary material 4 — Key to Capparimyia [file zookeys-428-097-s004.zip › SF4_ZooKeys_key to Capparimyia/key/SF4_ZooKeys_key to Capparimyia/Media/Images/head_orbitalseta_one.jpg]

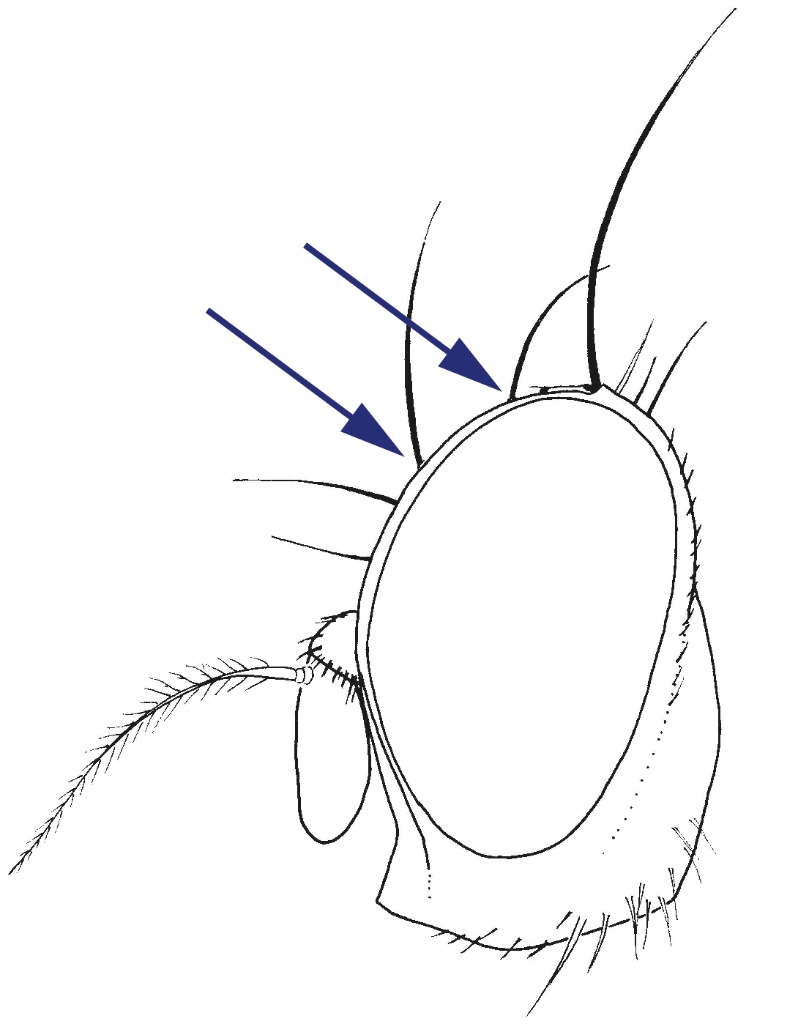

Supplement: Supplementary material 4 — Key to Capparimyia [file zookeys-428-097-s004.zip › SF4_ZooKeys_key to Capparimyia/key/SF4_ZooKeys_key to Capparimyia/Media/Images/head_orbitalseta_two.jpg]

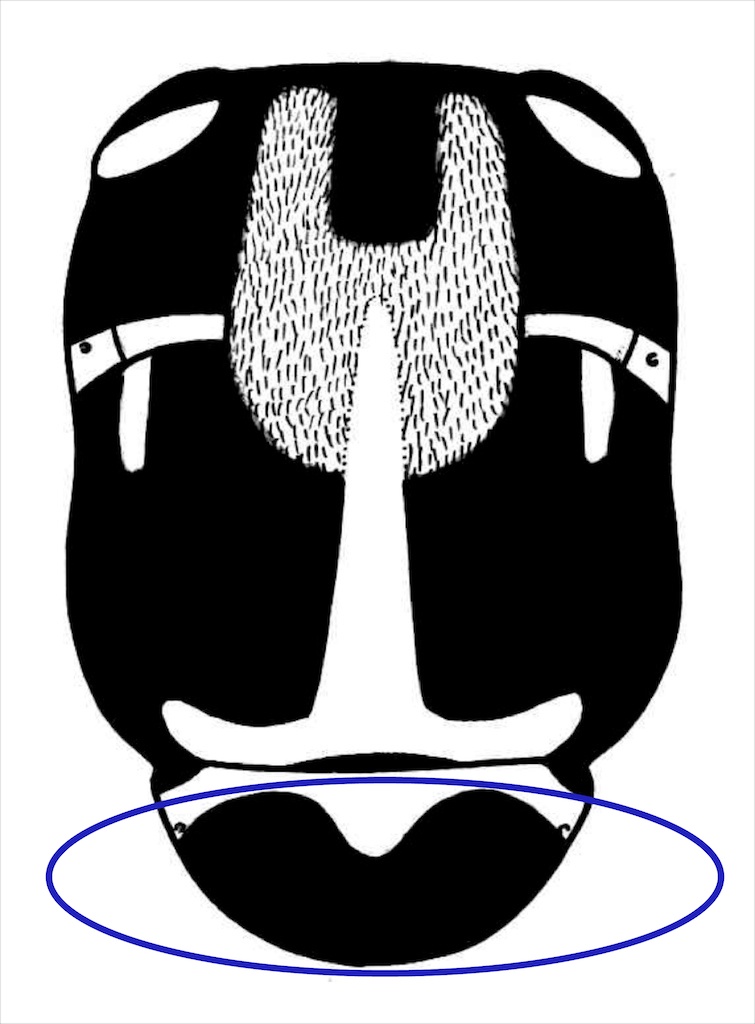

Supplement: Supplementary material 4 — Key to Capparimyia [file zookeys-428-097-s004.zip › SF4_ZooKeys_key to Capparimyia/key/SF4_ZooKeys_key to Capparimyia/Media/Images/thorax_apicscutspots_merged_104 mesonotum dorsal (drawing (c) De Meyer & Freidberg)_ps (1).jpg]

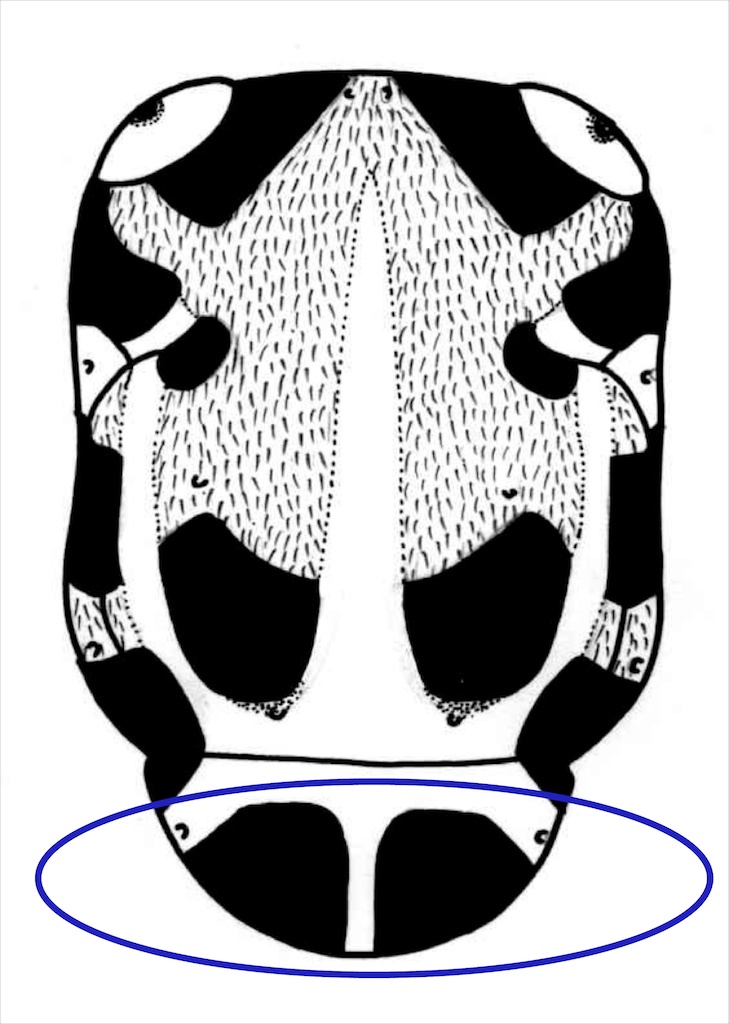

Supplement: Supplementary material 4 — Key to Capparimyia [file zookeys-428-097-s004.zip › SF4_ZooKeys_key to Capparimyia/key/SF4_ZooKeys_key to Capparimyia/Media/Images/thorax_apicscutspots_separated_113 mesonotum dorsal (drawing (c) De Meyer & Freidberg)_ps (1).jpg]

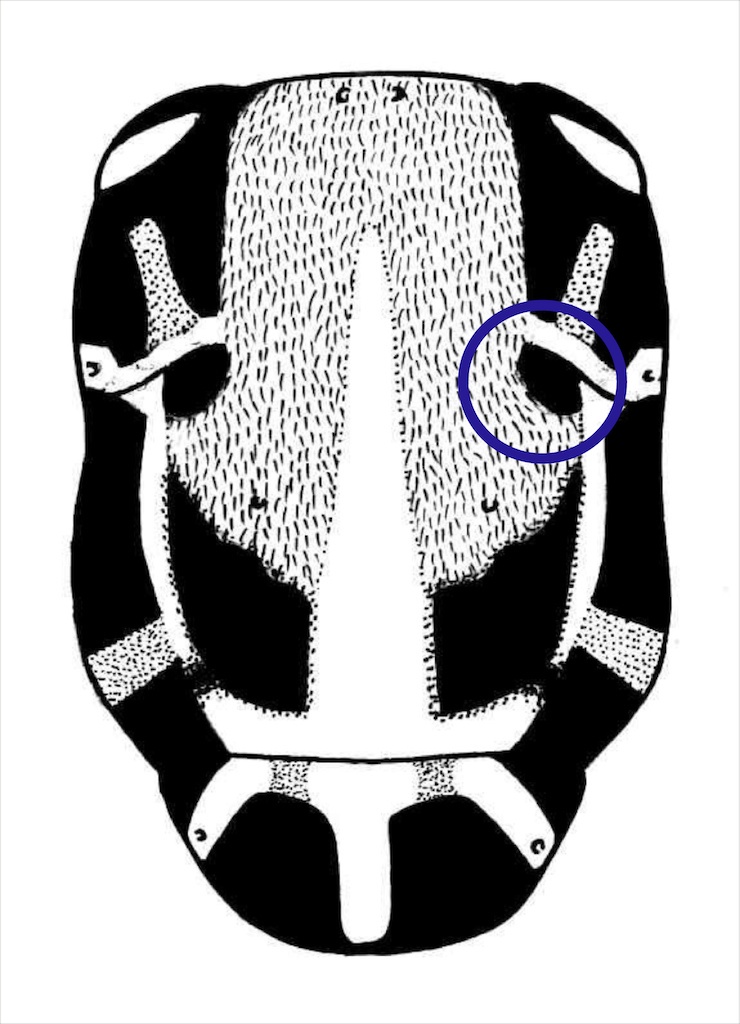

Supplement: Supplementary material 4 — Key to Capparimyia [file zookeys-428-097-s004.zip › SF4_ZooKeys_key to Capparimyia/key/SF4_ZooKeys_key to Capparimyia/Media/Images/thorax_blacksutspots_definedbutseparated_111 mesonotum dorsal (drawing (c) De Meyer & Freidberg)_ps.jpg]

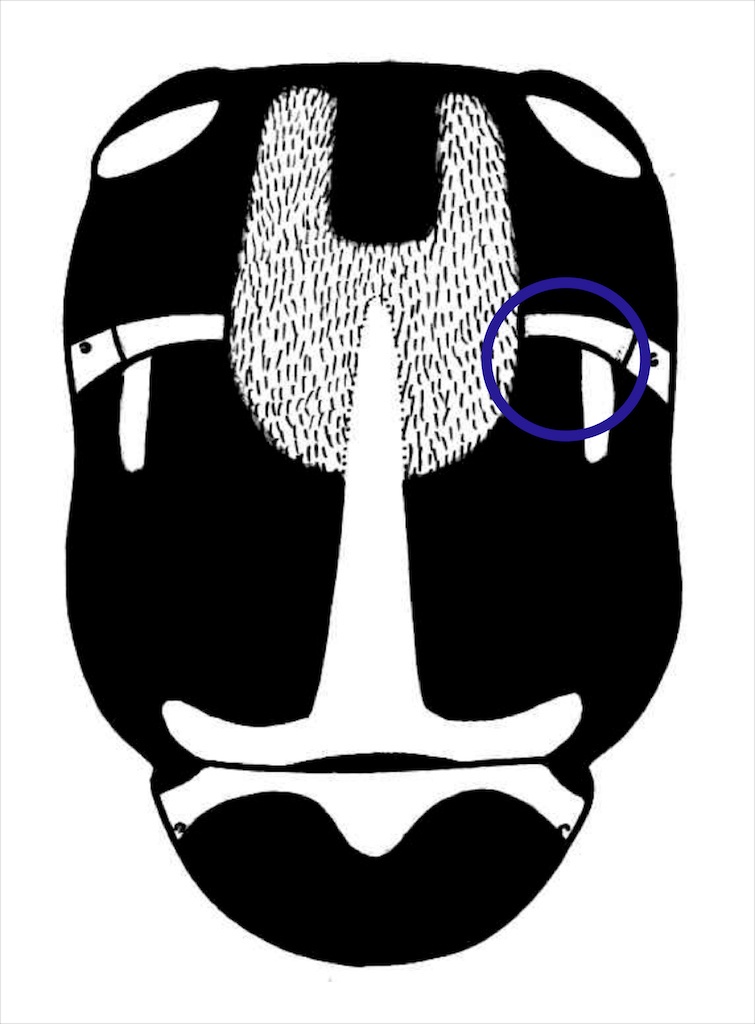

Supplement: Supplementary material 4 — Key to Capparimyia [file zookeys-428-097-s004.zip › SF4_ZooKeys_key to Capparimyia/key/SF4_ZooKeys_key to Capparimyia/Media/Images/thorax_blacksutspots_merged_104 mesonotum dorsal (drawing (c) De Meyer & Freidberg)_ps.jpg]

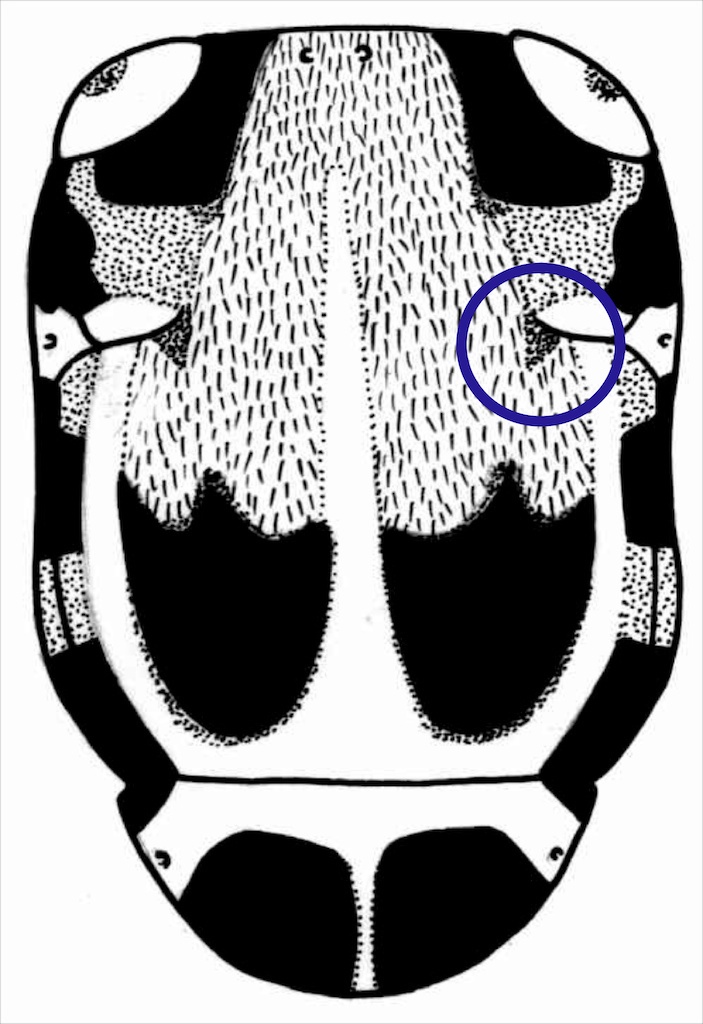

Supplement: Supplementary material 4 — Key to Capparimyia [file zookeys-428-097-s004.zip › SF4_ZooKeys_key to Capparimyia/key/SF4_ZooKeys_key to Capparimyia/Media/Images/thorax_blacksutspots_notdefinedbutseparated_112 mesonotum dorsal (drawing (c) De Meyer & Freidberg)_ps.jpg]

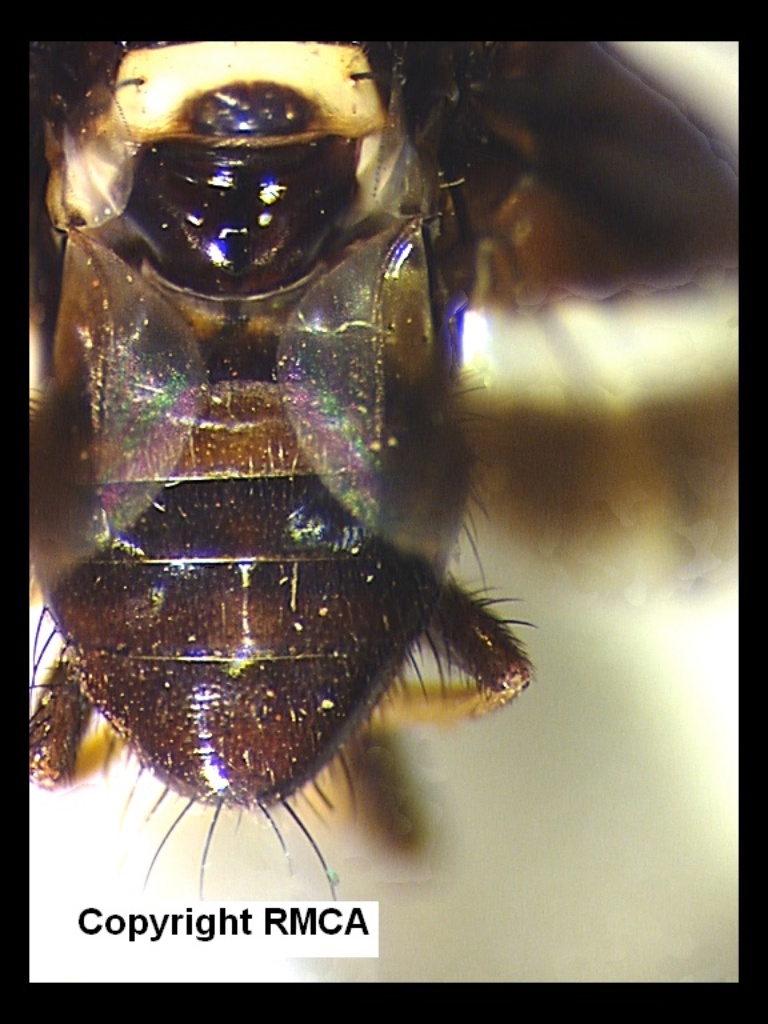

Supplement: Supplementary material 5 — Key to Carpophthoromyia [file zookeys-428-097-s005.zip › SF5_ZooKeys_key to Carpophthoromyia/key/SF5_ZooKeys_key to Carpophthoromyia/Media/Images/159 abdomen dorsal (automontage (c) RMCA).jpg]

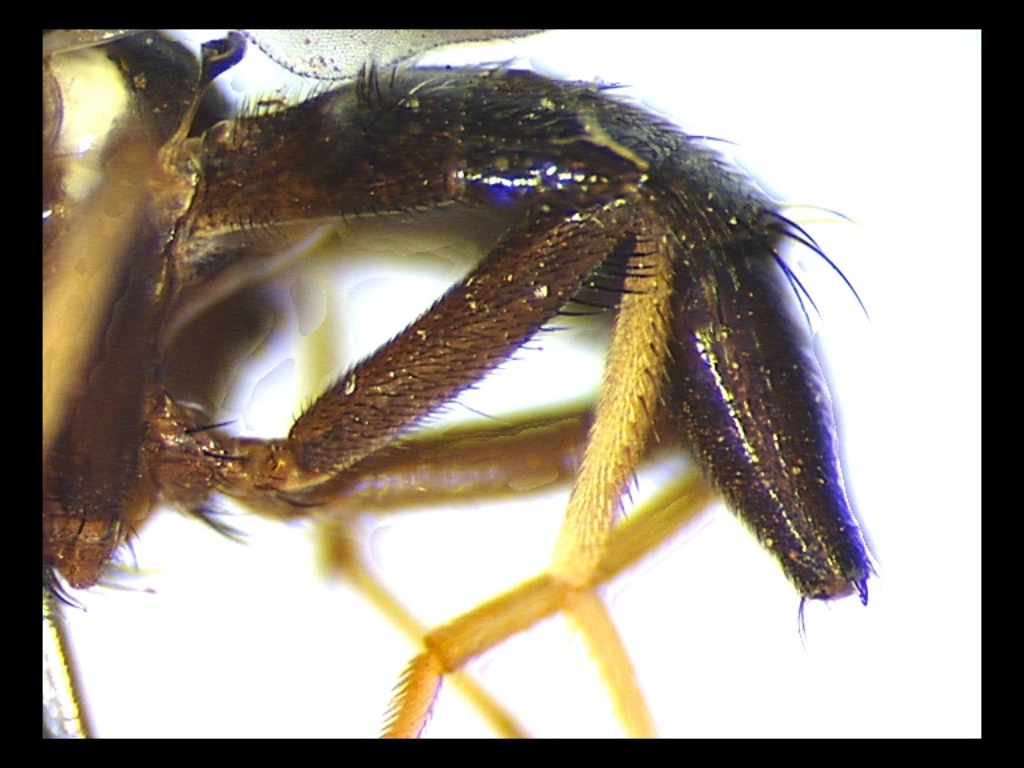

Supplement: Supplementary material 5 — Key to Carpophthoromyia [file zookeys-428-097-s005.zip › SF5_ZooKeys_key to Carpophthoromyia/key/SF5_ZooKeys_key to Carpophthoromyia/Media/Images/159 abdomen lateral (automontage (c) RMCA).jpg]

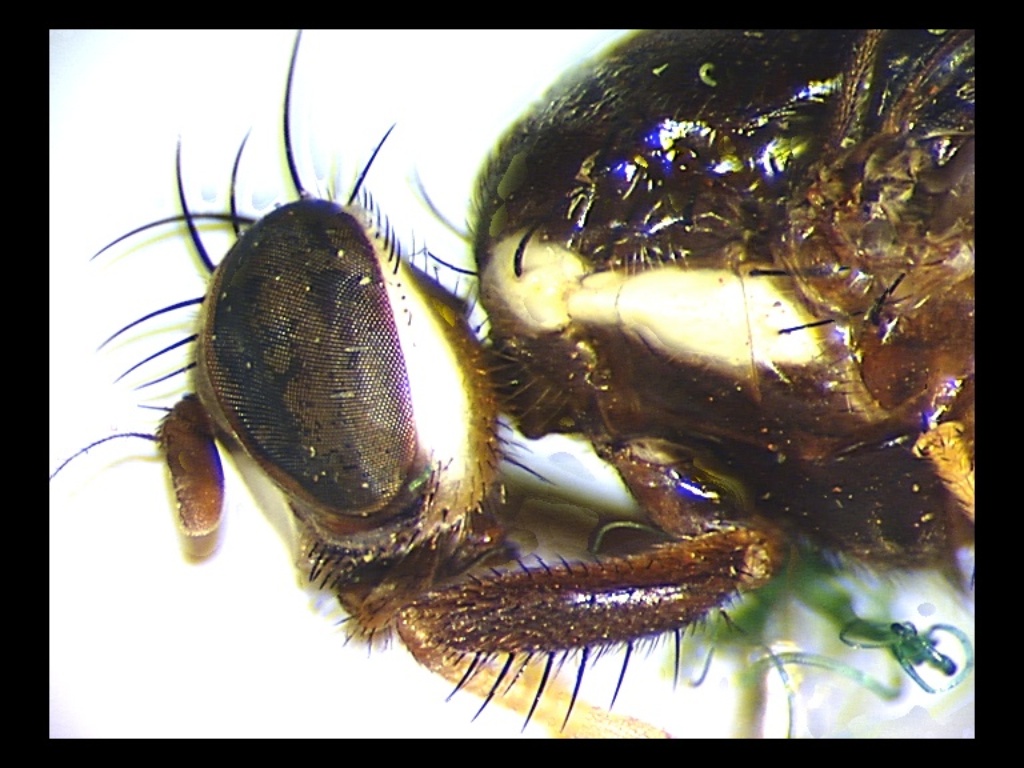

Supplement: Supplementary material 5 — Key to Carpophthoromyia [file zookeys-428-097-s005.zip › SF5_ZooKeys_key to Carpophthoromyia/key/SF5_ZooKeys_key to Carpophthoromyia/Media/Images/159 head and thorax lateral (automontage (c) RMCA).jpg]

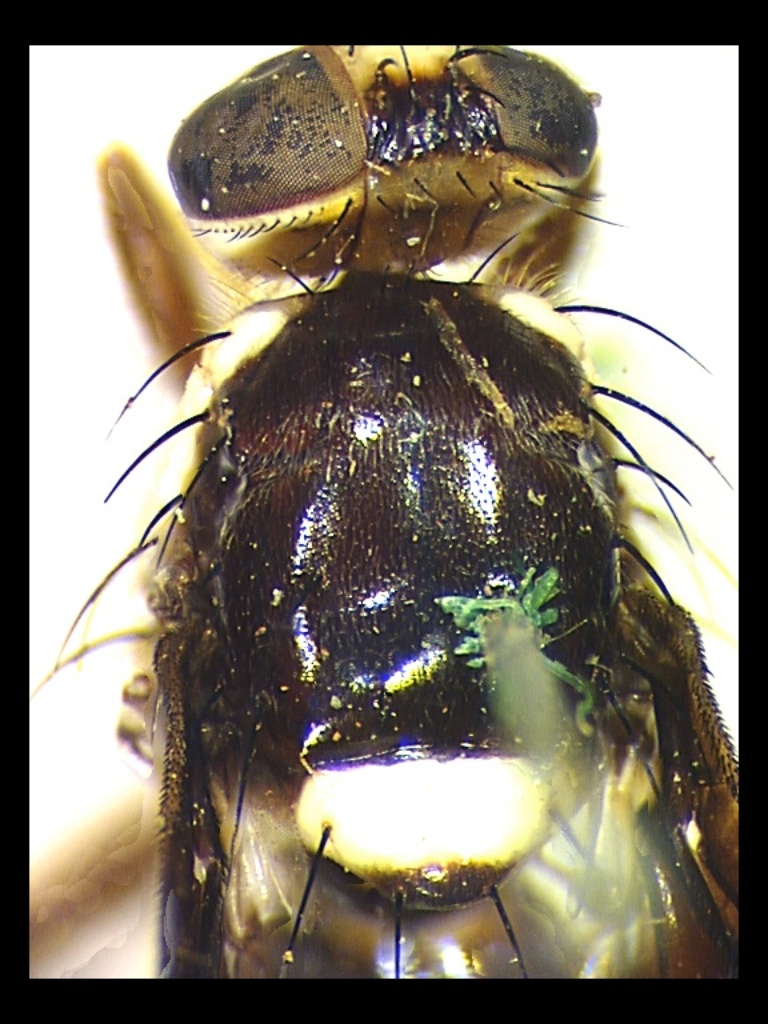

Supplement: Supplementary material 5 — Key to Carpophthoromyia [file zookeys-428-097-s005.zip › SF5_ZooKeys_key to Carpophthoromyia/key/SF5_ZooKeys_key to Carpophthoromyia/Media/Images/159 mesonotum dorsal (automontage (c) RMCA).jpg]

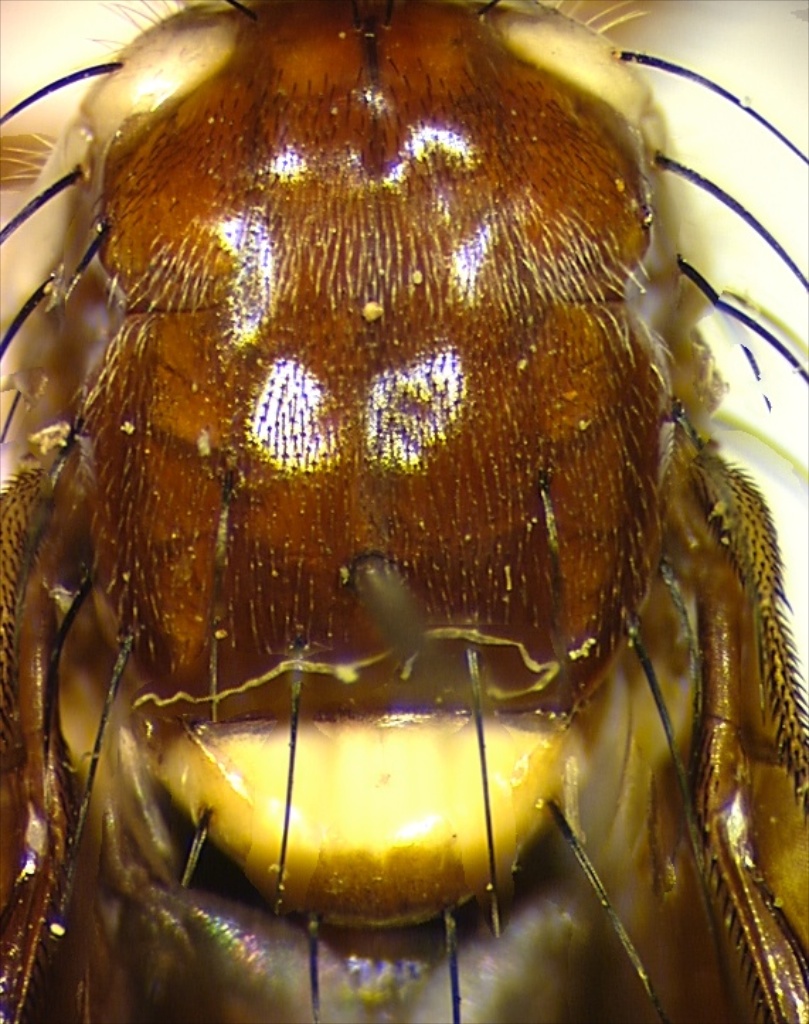

Supplement: Supplementary material 5 — Key to Carpophthoromyia [file zookeys-428-097-s005.zip › SF5_ZooKeys_key to Carpophthoromyia/key/SF5_ZooKeys_key to Carpophthoromyia/Media/Images/159 mesonotum dorsal2 (automontage (c) RMCA).jpg]

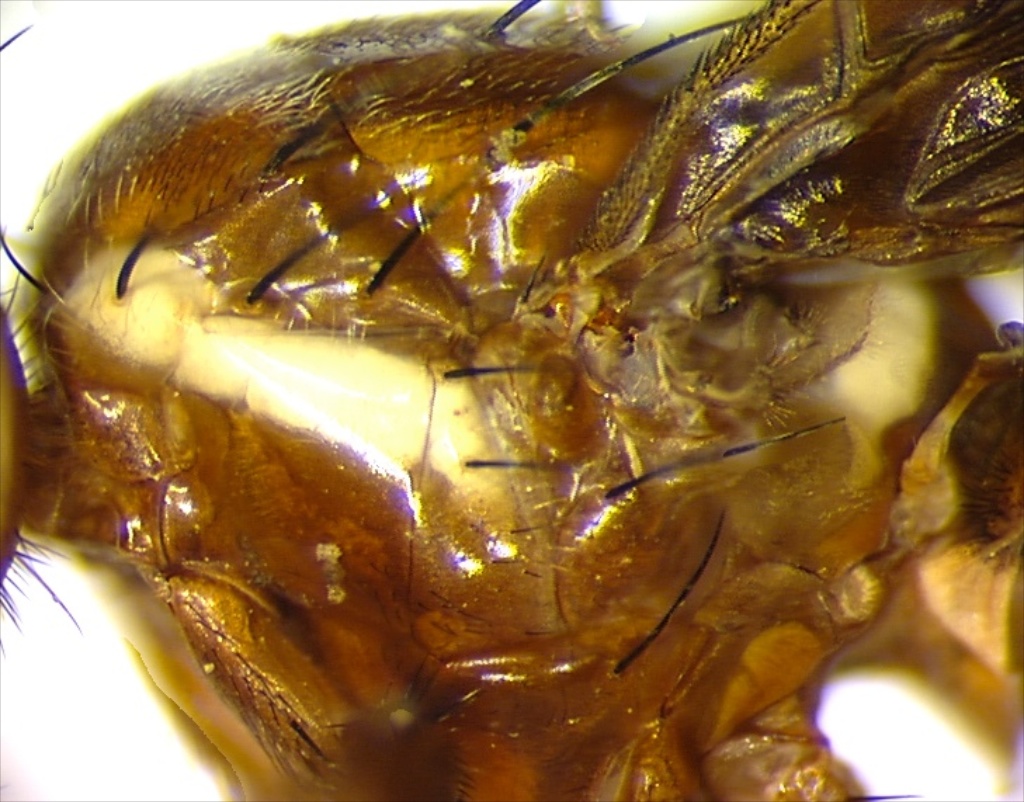

Supplement: Supplementary material 5 — Key to Carpophthoromyia [file zookeys-428-097-s005.zip › SF5_ZooKeys_key to Carpophthoromyia/key/SF5_ZooKeys_key to Carpophthoromyia/Media/Images/159 thorax lateral (automontage (c) RMCA).jpg]

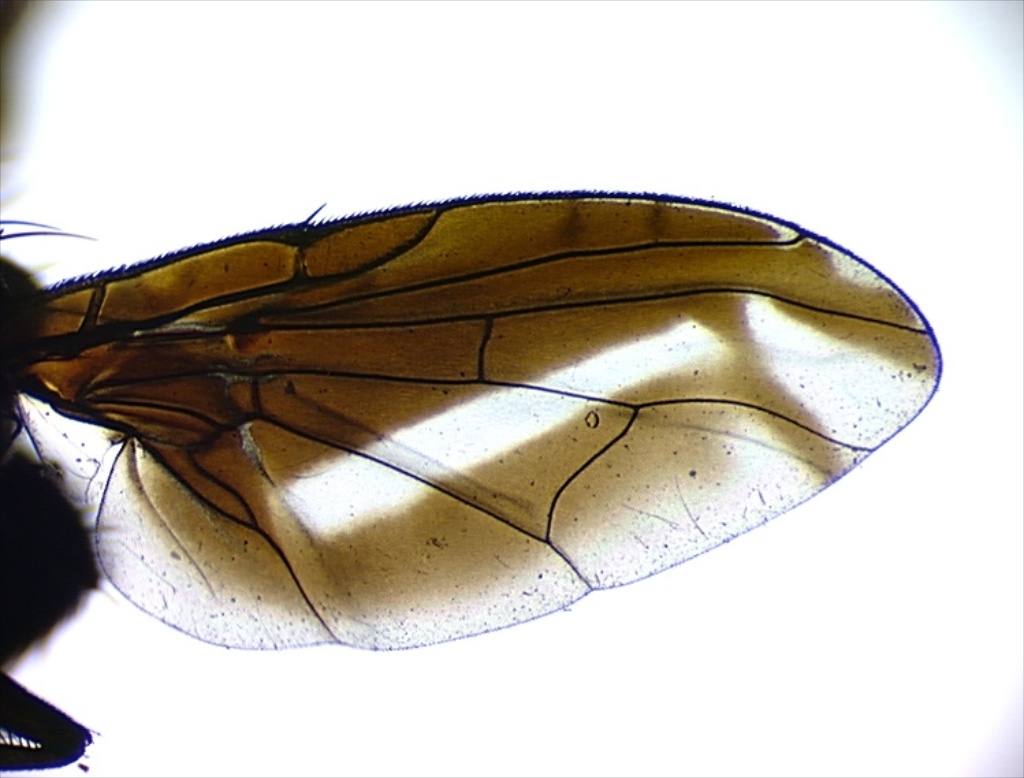

Supplement: Supplementary material 5 — Key to Carpophthoromyia [file zookeys-428-097-s005.zip › SF5_ZooKeys_key to Carpophthoromyia/key/SF5_ZooKeys_key to Carpophthoromyia/Media/Images/159 wing dorsal (automontage (c) RMCA).jpg]

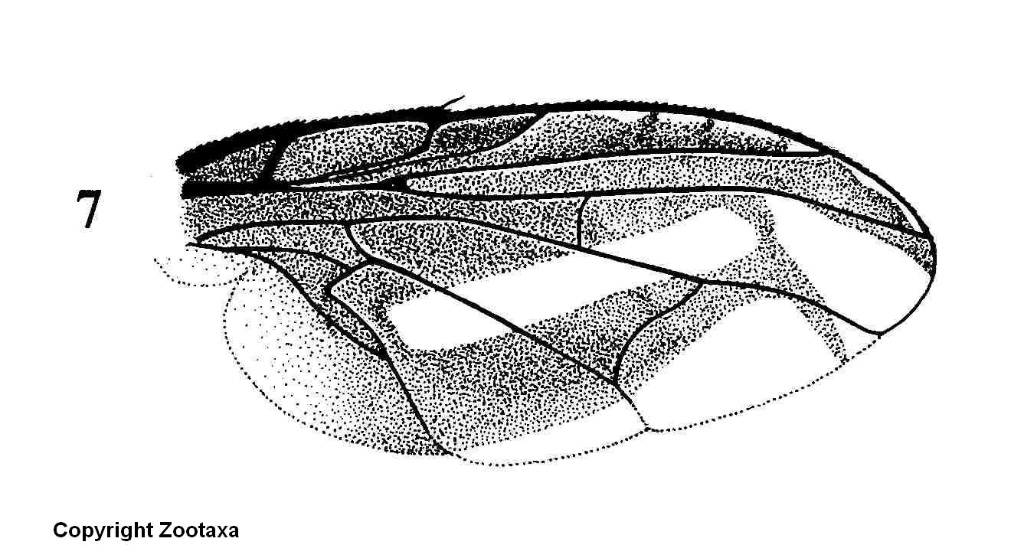

Supplement: Supplementary material 5 — Key to Carpophthoromyia [file zookeys-428-097-s005.zip › SF5_ZooKeys_key to Carpophthoromyia/key/SF5_ZooKeys_key to Carpophthoromyia/Media/Images/159 wing dorsal (drawing (c) Zootaxa).jpg]

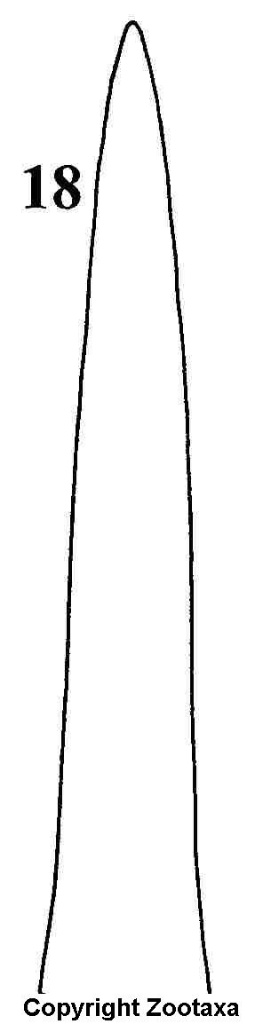

Supplement: Supplementary material 5 — Key to Carpophthoromyia [file zookeys-428-097-s005.zip › SF5_ZooKeys_key to Carpophthoromyia/key/SF5_ZooKeys_key to Carpophthoromyia/Media/Images/355 aculeus dorsal (drawing (c) Zootaxa).jpg]

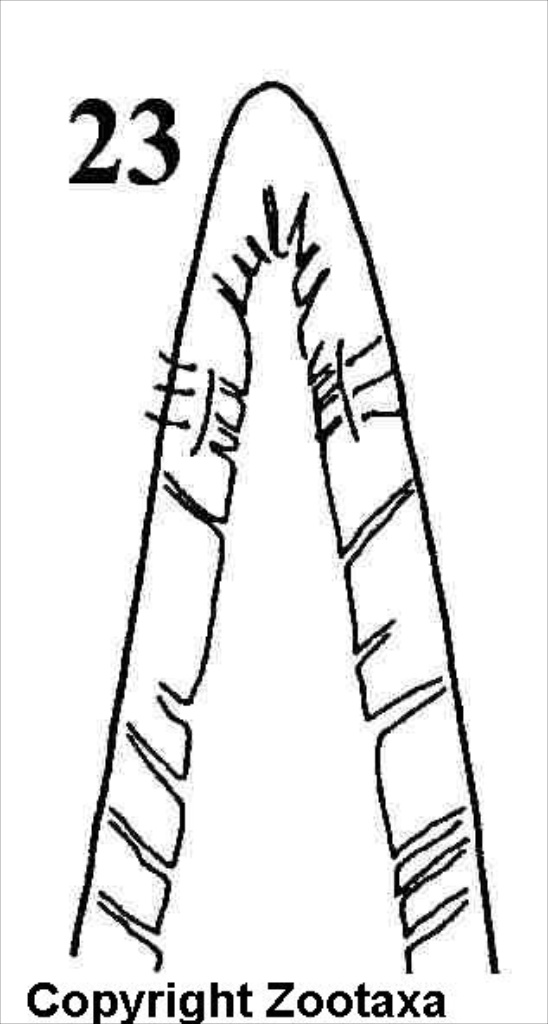

Supplement: Supplementary material 5 — Key to Carpophthoromyia [file zookeys-428-097-s005.zip › SF5_ZooKeys_key to Carpophthoromyia/key/SF5_ZooKeys_key to Carpophthoromyia/Media/Images/355 aculeus tip dorsal (drawing (c) Zootaxa).jpg]

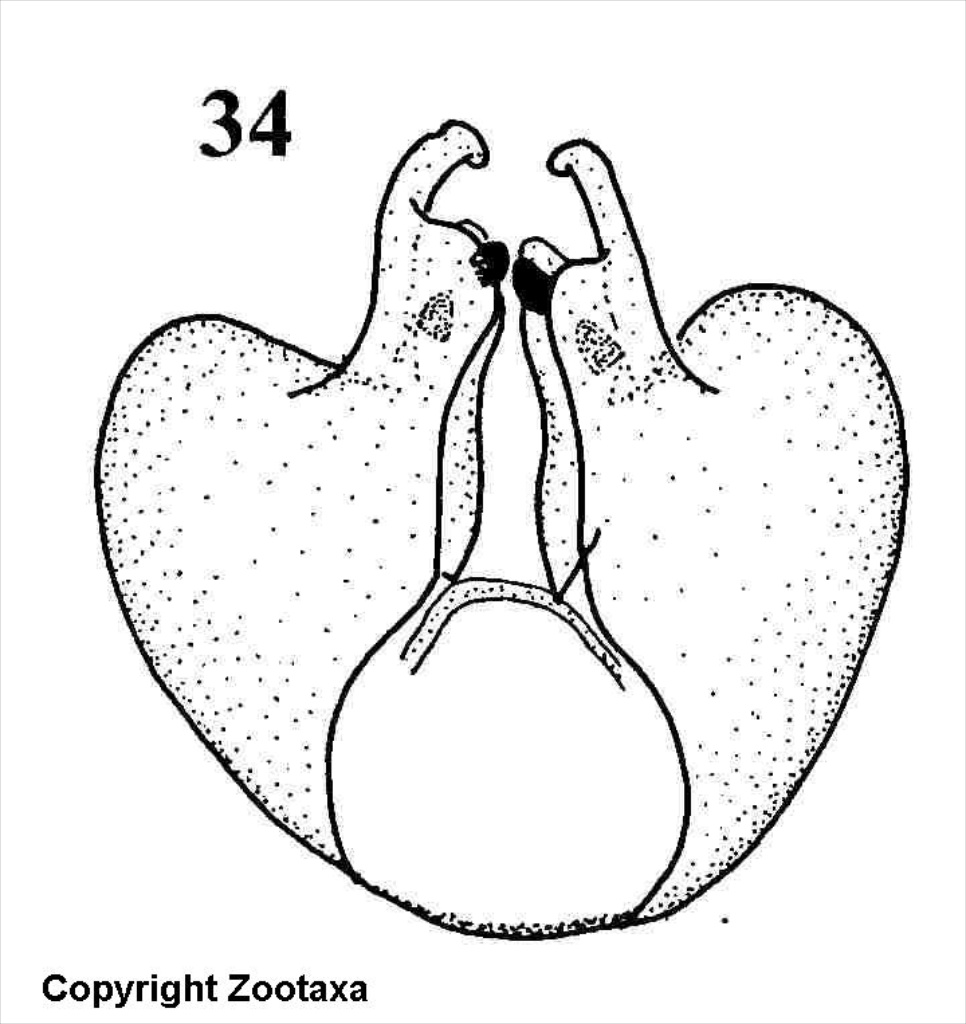

Supplement: Supplementary material 5 — Key to Carpophthoromyia [file zookeys-428-097-s005.zip › SF5_ZooKeys_key to Carpophthoromyia/key/SF5_ZooKeys_key to Carpophthoromyia/Media/Images/355 epandrium posterior (drawing (c) Zootaxa).jpg]

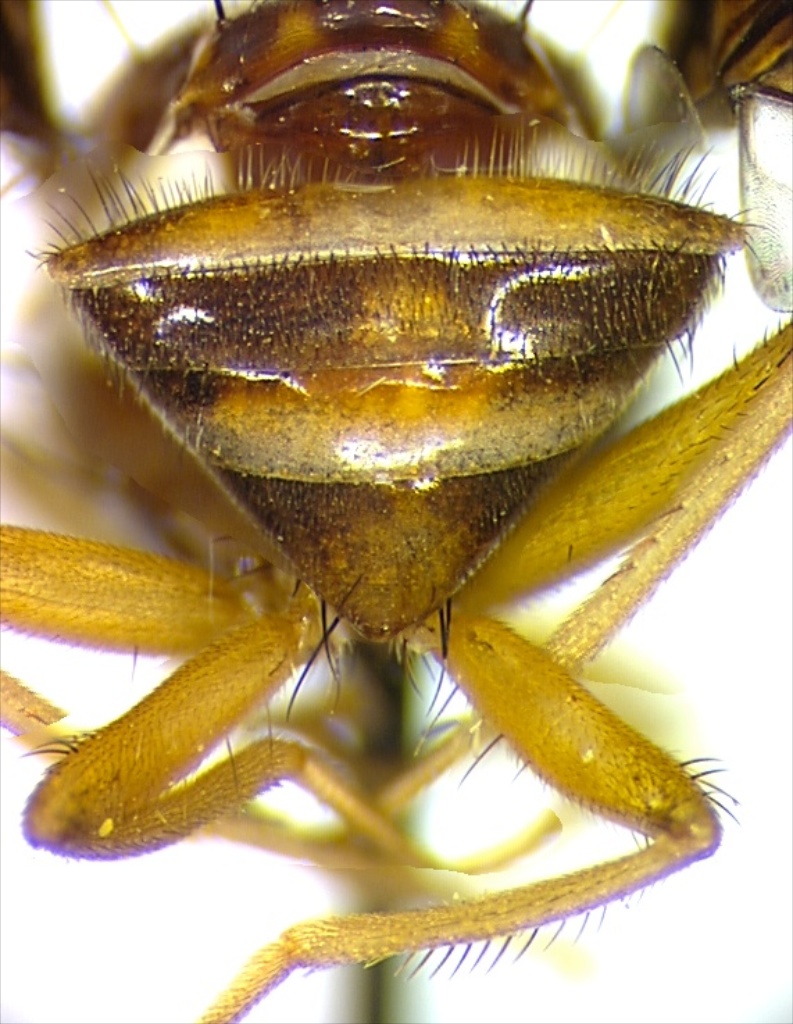

Supplement: Supplementary material 5 — Key to Carpophthoromyia [file zookeys-428-097-s005.zip › SF5_ZooKeys_key to Carpophthoromyia/key/SF5_ZooKeys_key to Carpophthoromyia/Media/Images/356 abdomen dorsal (automontage (c) RMCA).jpg]

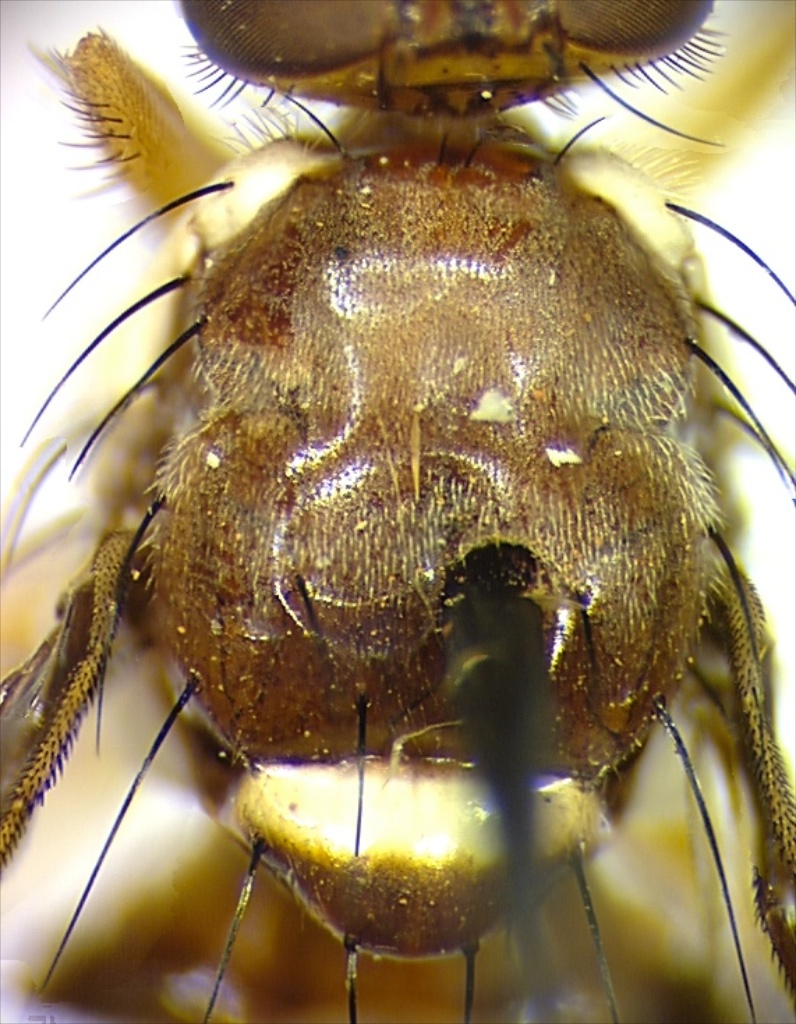

Supplement: Supplementary material 5 — Key to Carpophthoromyia [file zookeys-428-097-s005.zip › SF5_ZooKeys_key to Carpophthoromyia/key/SF5_ZooKeys_key to Carpophthoromyia/Media/Images/356 mesonotum dorsal (automontage (c) RMCA).jpg]

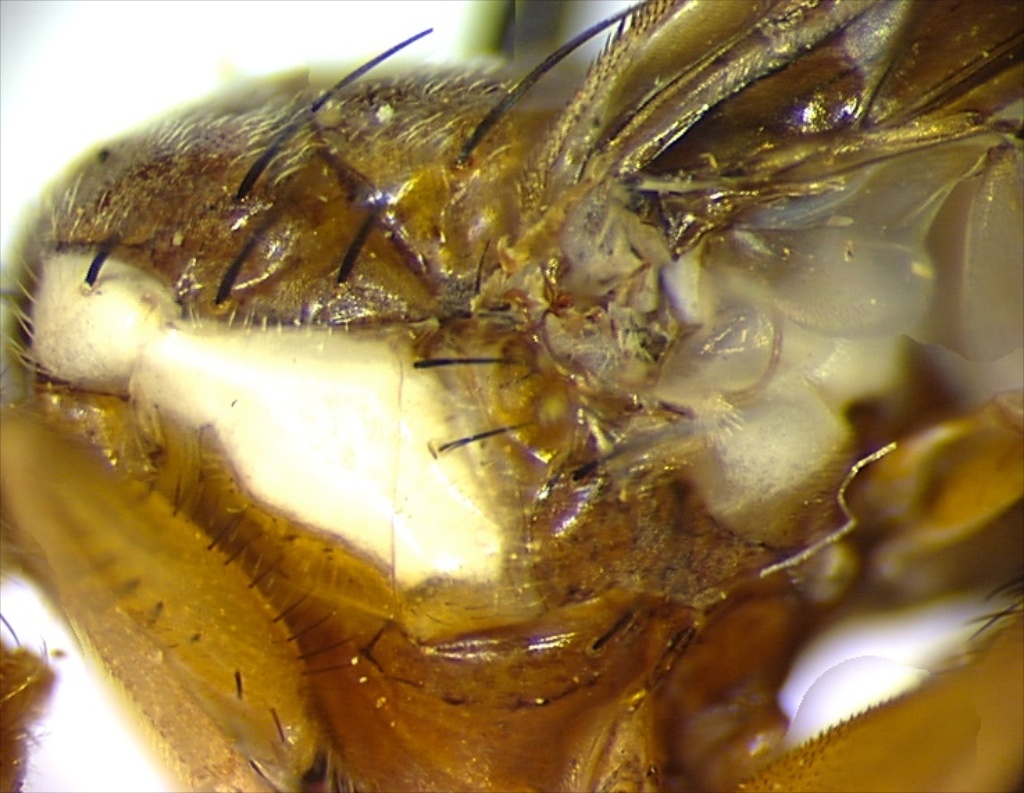

Supplement: Supplementary material 5 — Key to Carpophthoromyia [file zookeys-428-097-s005.zip › SF5_ZooKeys_key to Carpophthoromyia/key/SF5_ZooKeys_key to Carpophthoromyia/Media/Images/356 thorax lateral (automontage (c) RMCA).jpg]

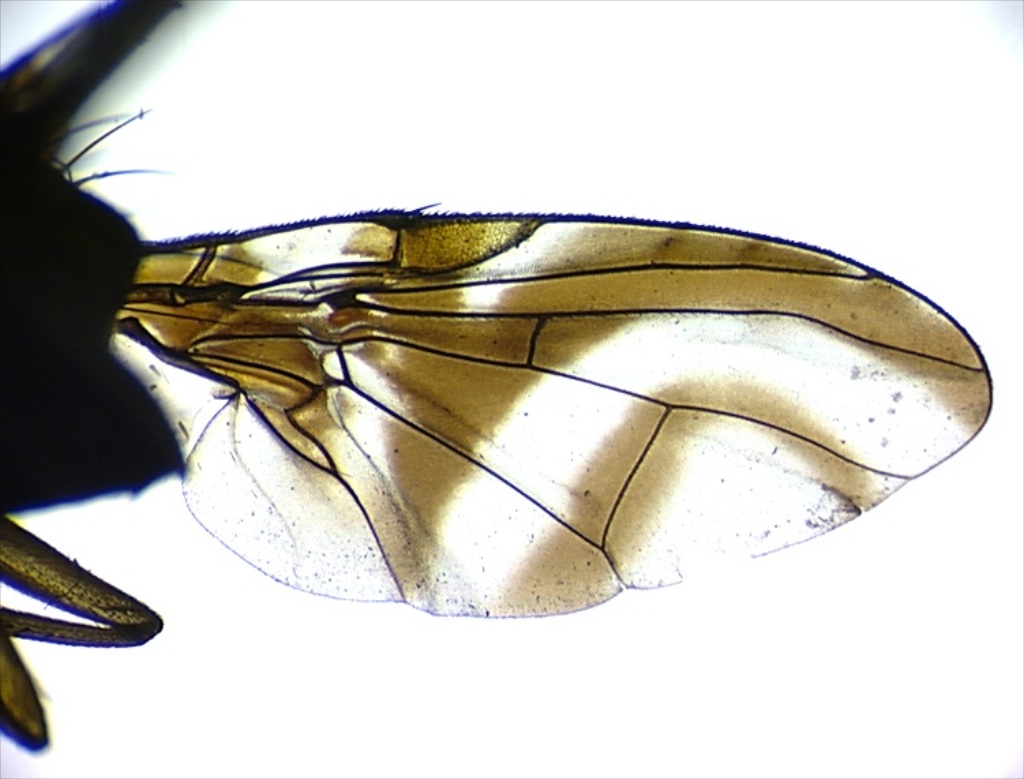

Supplement: Supplementary material 5 — Key to Carpophthoromyia [file zookeys-428-097-s005.zip › SF5_ZooKeys_key to Carpophthoromyia/key/SF5_ZooKeys_key to Carpophthoromyia/Media/Images/356 wing dorsal (automontage (c) RMCA).jpg]

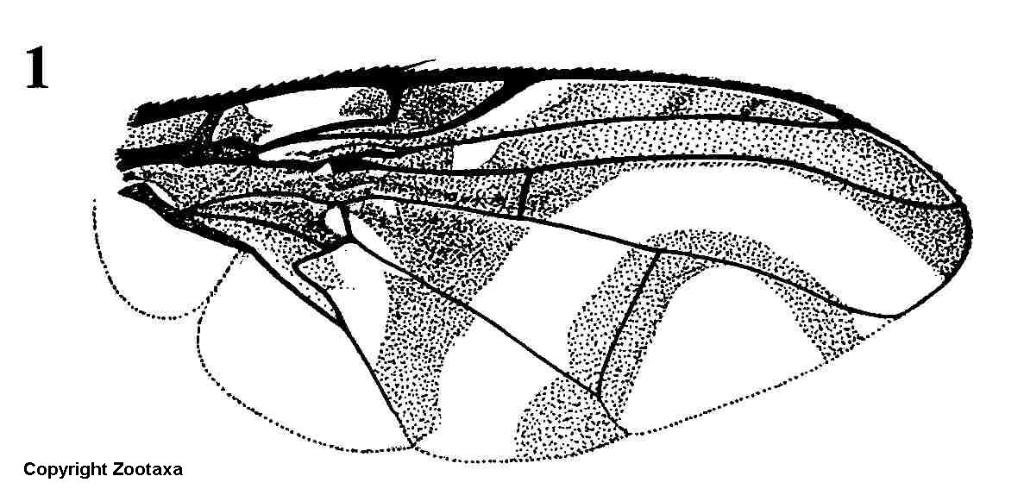

Supplement: Supplementary material 5 — Key to Carpophthoromyia [file zookeys-428-097-s005.zip › SF5_ZooKeys_key to Carpophthoromyia/key/SF5_ZooKeys_key to Carpophthoromyia/Media/Images/356 wing dorsal (drawing (c) Zootaxa).jpg]

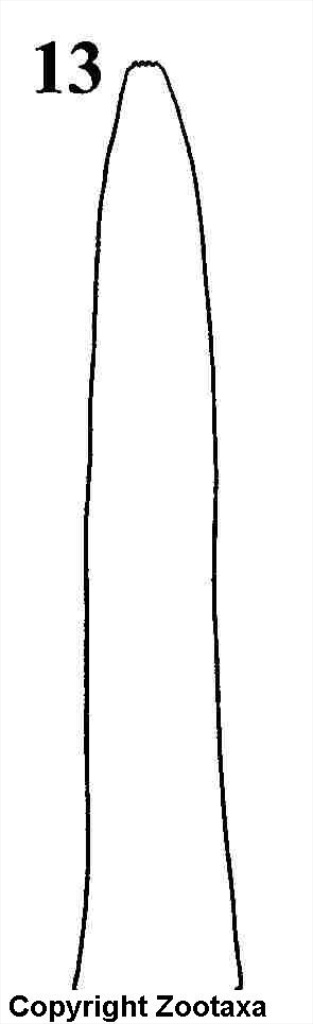

Supplement: Supplementary material 5 — Key to Carpophthoromyia [file zookeys-428-097-s005.zip › SF5_ZooKeys_key to Carpophthoromyia/key/SF5_ZooKeys_key to Carpophthoromyia/Media/Images/357 aculeus dorsal (drawing (c) Zootaxa).jpg]

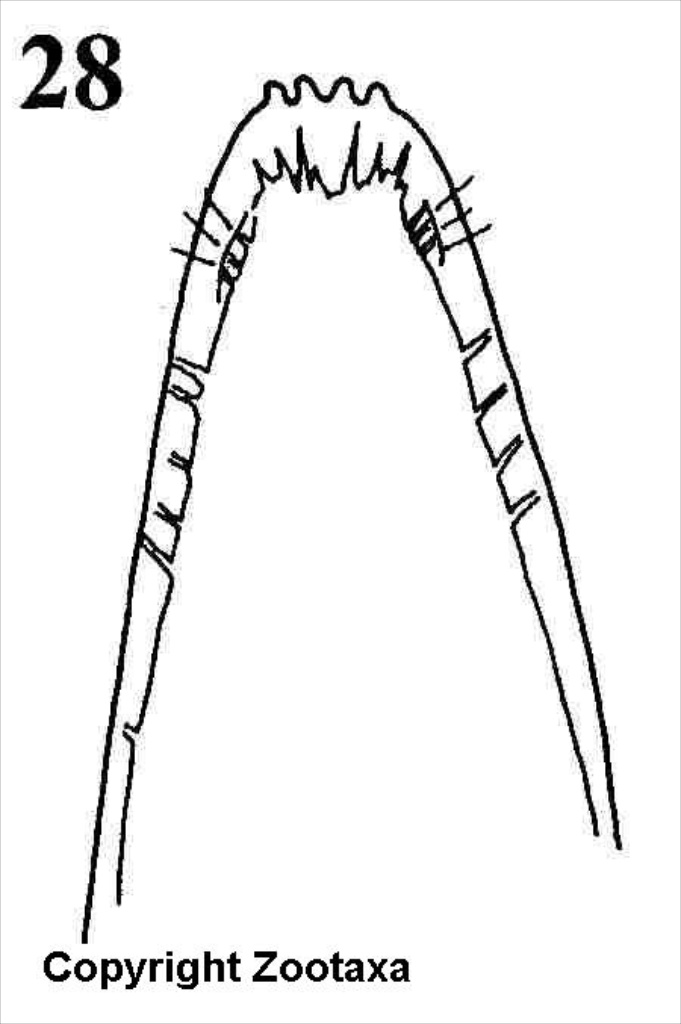

Supplement: Supplementary material 5 — Key to Carpophthoromyia [file zookeys-428-097-s005.zip › SF5_ZooKeys_key to Carpophthoromyia/key/SF5_ZooKeys_key to Carpophthoromyia/Media/Images/357 aculeus tip dorsal (drawing (c) Zootaxa).jpg]

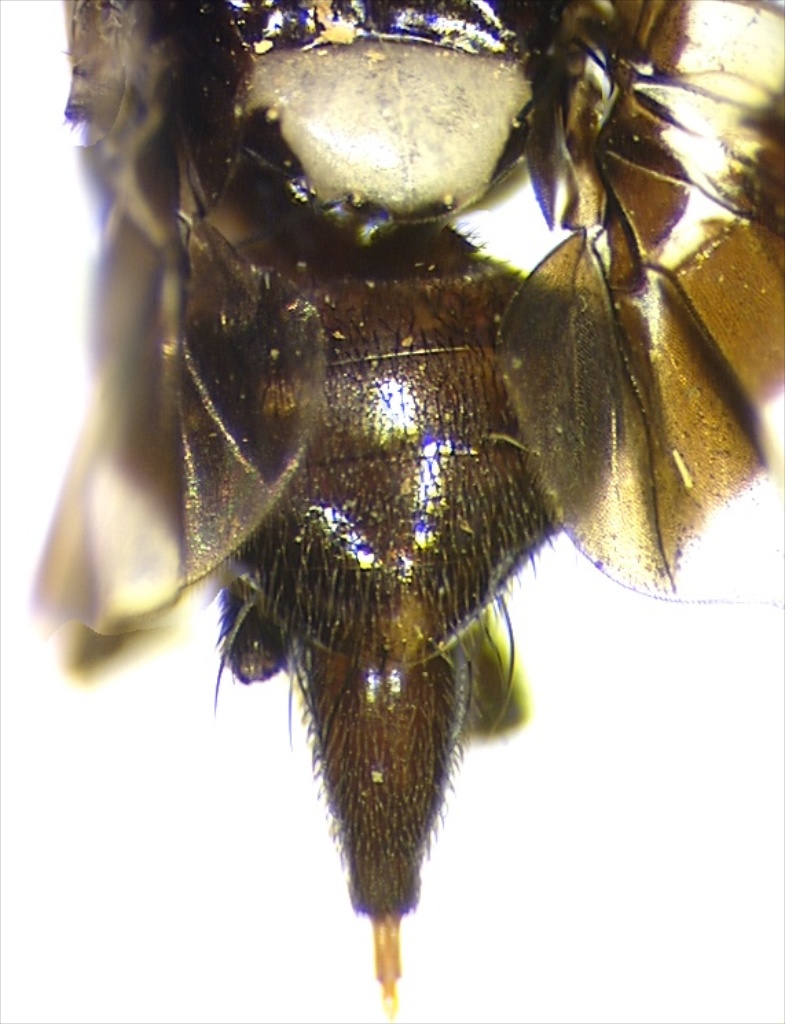

Supplement: Supplementary material 5 — Key to Carpophthoromyia [file zookeys-428-097-s005.zip › SF5_ZooKeys_key to Carpophthoromyia/key/SF5_ZooKeys_key to Carpophthoromyia/Media/Images/358 abdomen dorsal (automontage (c) RMCA).jpg]

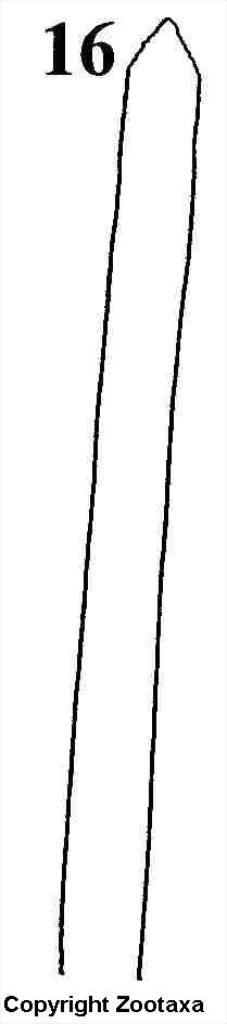

Supplement: Supplementary material 5 — Key to Carpophthoromyia [file zookeys-428-097-s005.zip › SF5_ZooKeys_key to Carpophthoromyia/key/SF5_ZooKeys_key to Carpophthoromyia/Media/Images/358 aculeus dorsal (drawing (c) Zootaxa).jpg]

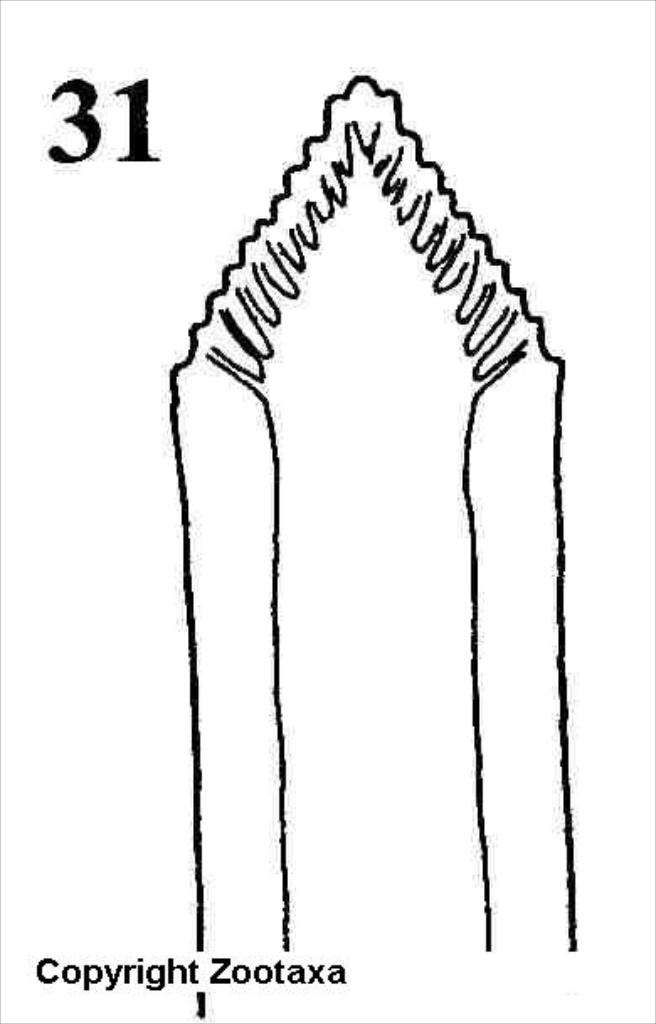

Supplement: Supplementary material 5 — Key to Carpophthoromyia [file zookeys-428-097-s005.zip › SF5_ZooKeys_key to Carpophthoromyia/key/SF5_ZooKeys_key to Carpophthoromyia/Media/Images/358 aculeus tip dorsal (drawing (c) Zootaxa).jpg]

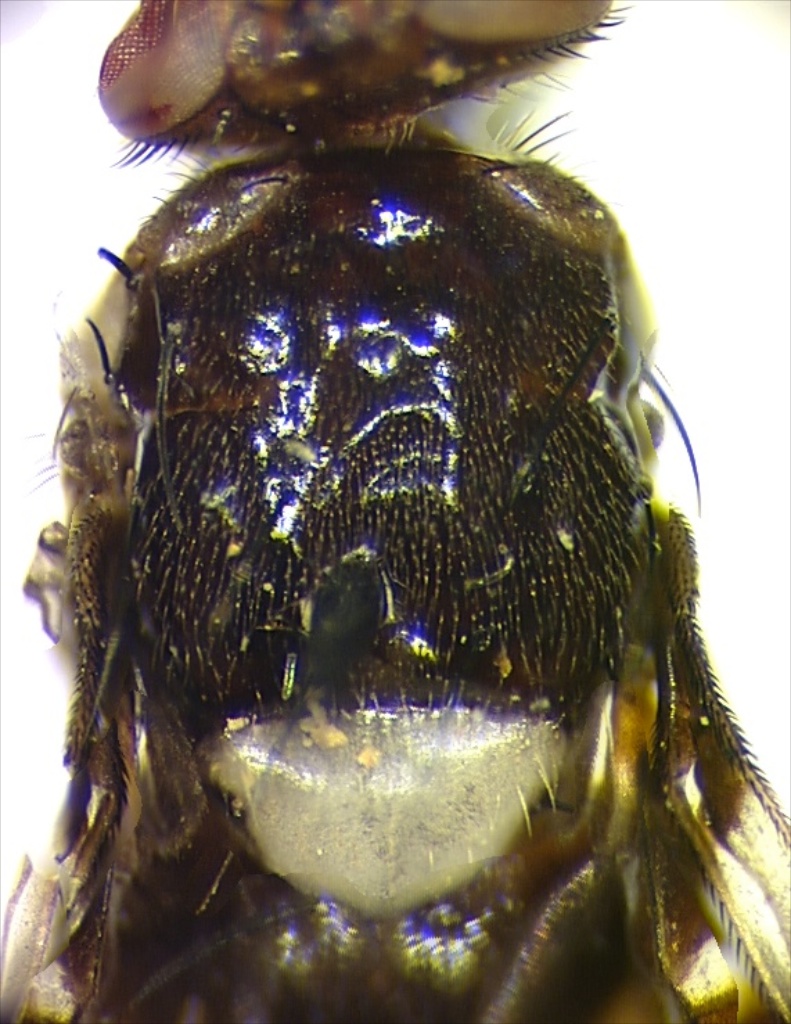

Supplement: Supplementary material 5 — Key to Carpophthoromyia [file zookeys-428-097-s005.zip › SF5_ZooKeys_key to Carpophthoromyia/key/SF5_ZooKeys_key to Carpophthoromyia/Media/Images/358 mesonotum dorsal (automontage (c) RMCA).jpg]

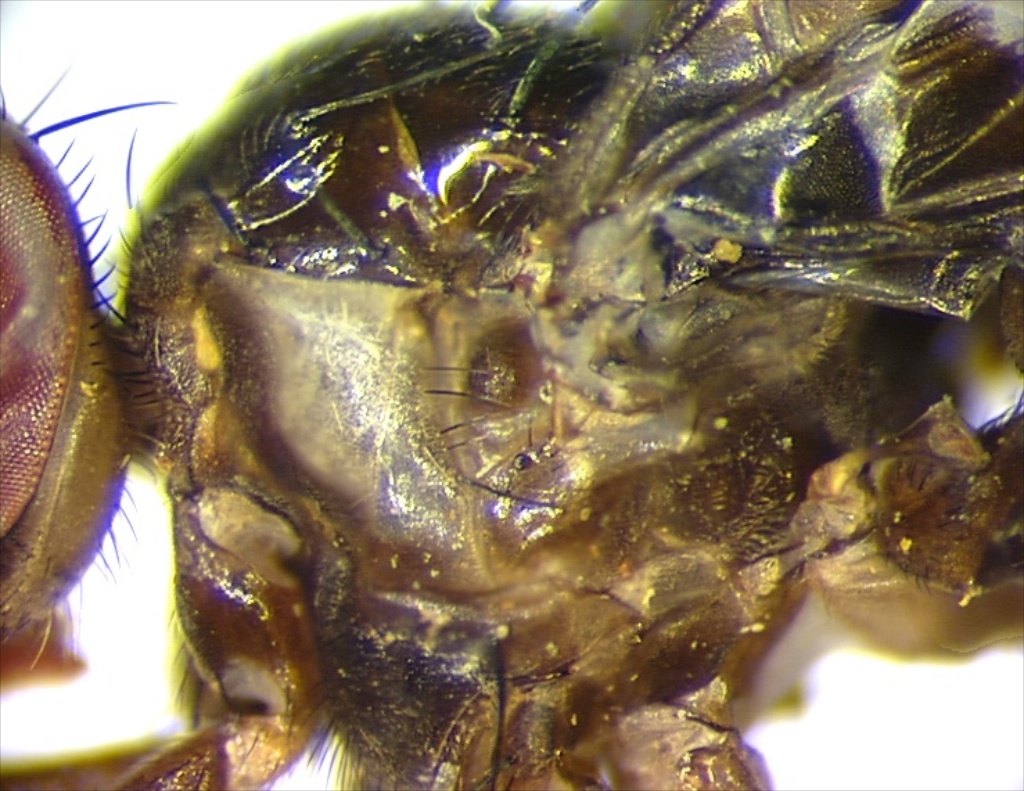

Supplement: Supplementary material 5 — Key to Carpophthoromyia [file zookeys-428-097-s005.zip › SF5_ZooKeys_key to Carpophthoromyia/key/SF5_ZooKeys_key to Carpophthoromyia/Media/Images/358 thorax lateral (automontage (c) RMCA).jpg]

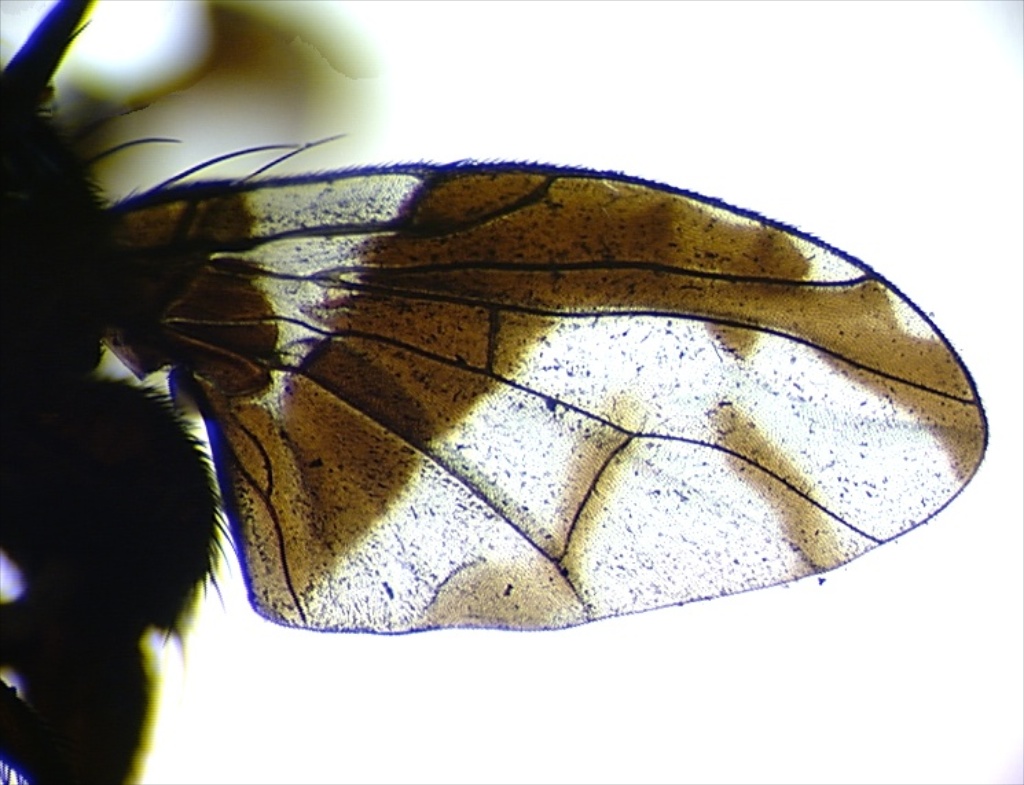

Supplement: Supplementary material 5 — Key to Carpophthoromyia [file zookeys-428-097-s005.zip › SF5_ZooKeys_key to Carpophthoromyia/key/SF5_ZooKeys_key to Carpophthoromyia/Media/Images/358 wing dorsal (automontage (c) RMCA).jpg]

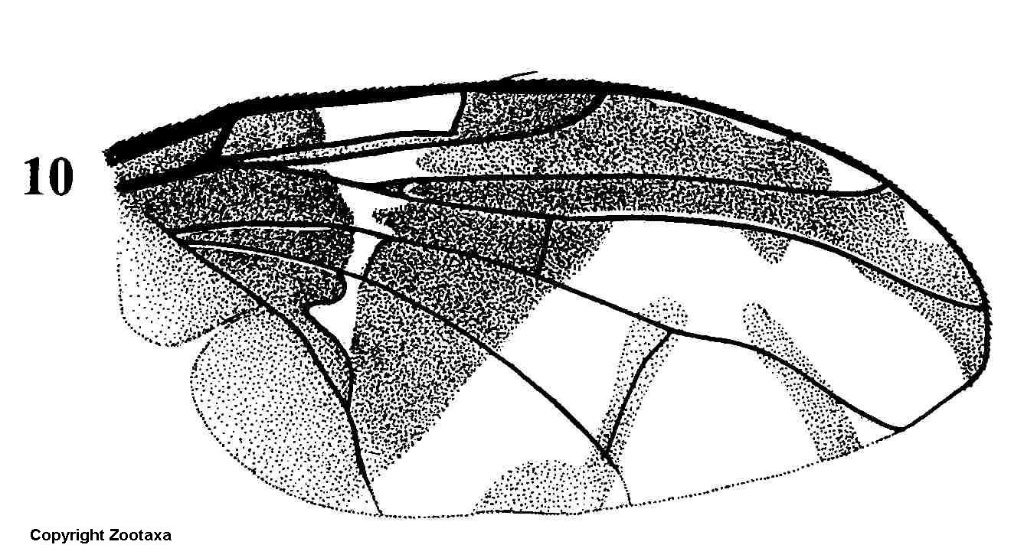

Supplement: Supplementary material 5 — Key to Carpophthoromyia [file zookeys-428-097-s005.zip › SF5_ZooKeys_key to Carpophthoromyia/key/SF5_ZooKeys_key to Carpophthoromyia/Media/Images/358 wing dorsal (drawing (c) Zootaxa).jpg]

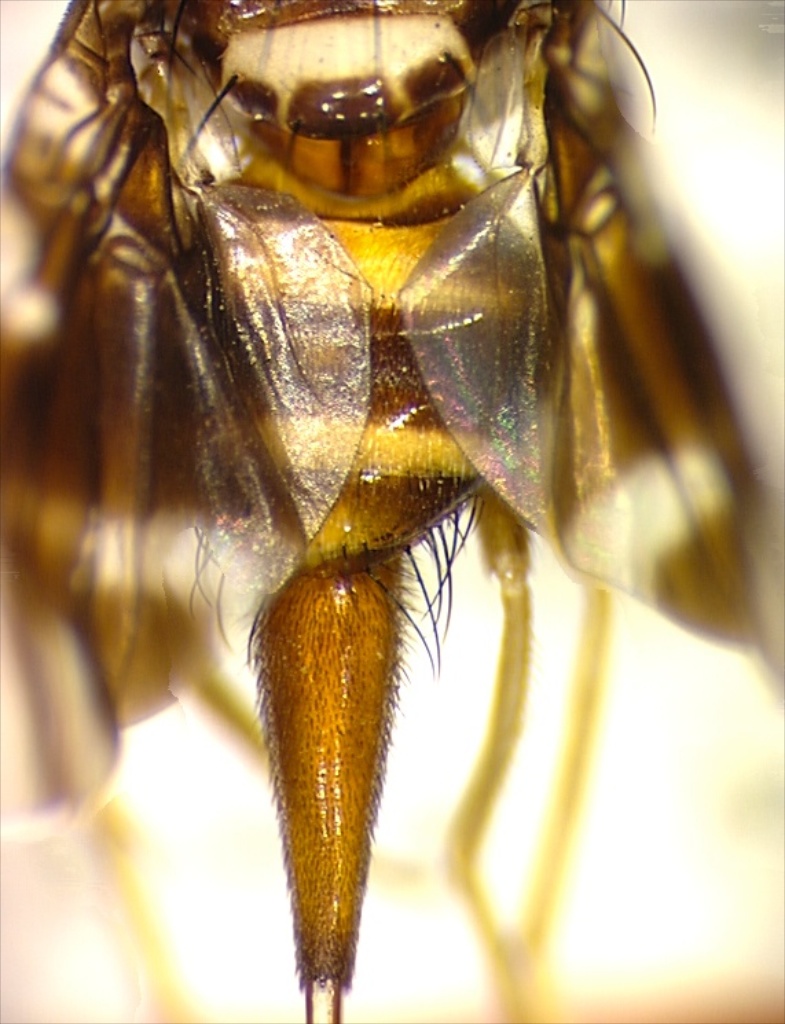

Supplement: Supplementary material 5 — Key to Carpophthoromyia [file zookeys-428-097-s005.zip › SF5_ZooKeys_key to Carpophthoromyia/key/SF5_ZooKeys_key to Carpophthoromyia/Media/Images/359 abdomen dorsal (automontage (c) RMCA).jpg]

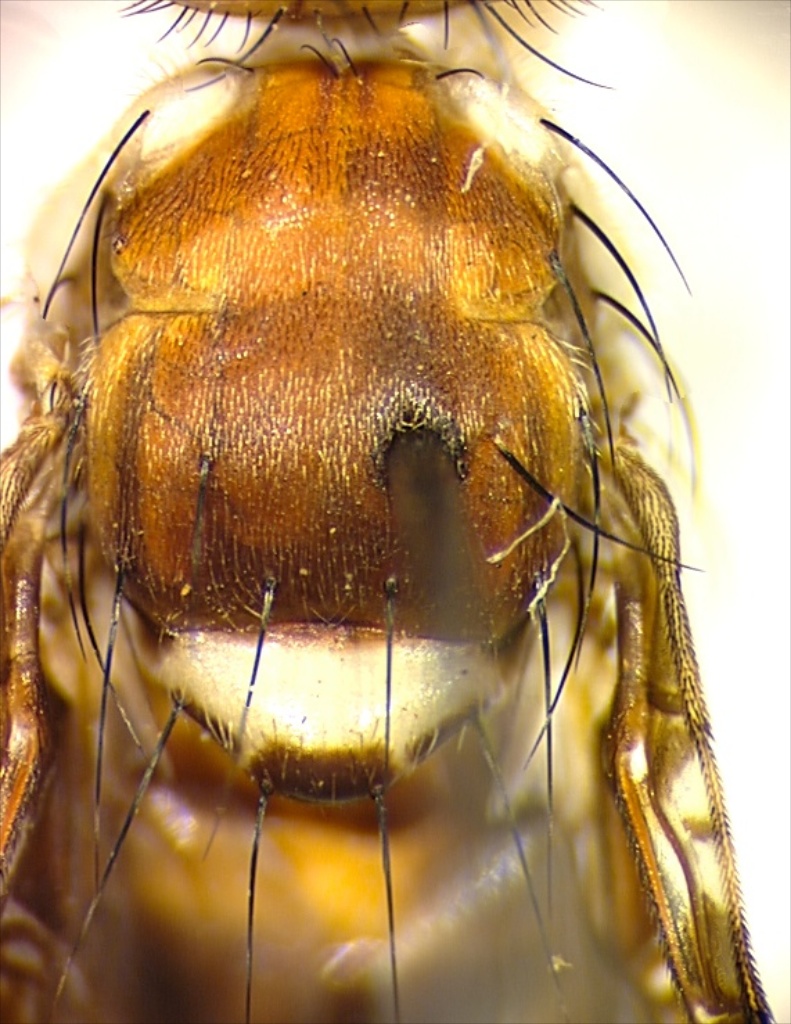

Supplement: Supplementary material 5 — Key to Carpophthoromyia [file zookeys-428-097-s005.zip › SF5_ZooKeys_key to Carpophthoromyia/key/SF5_ZooKeys_key to Carpophthoromyia/Media/Images/359 mesonotum dorsal (automontage (c) RMCA).jpg]

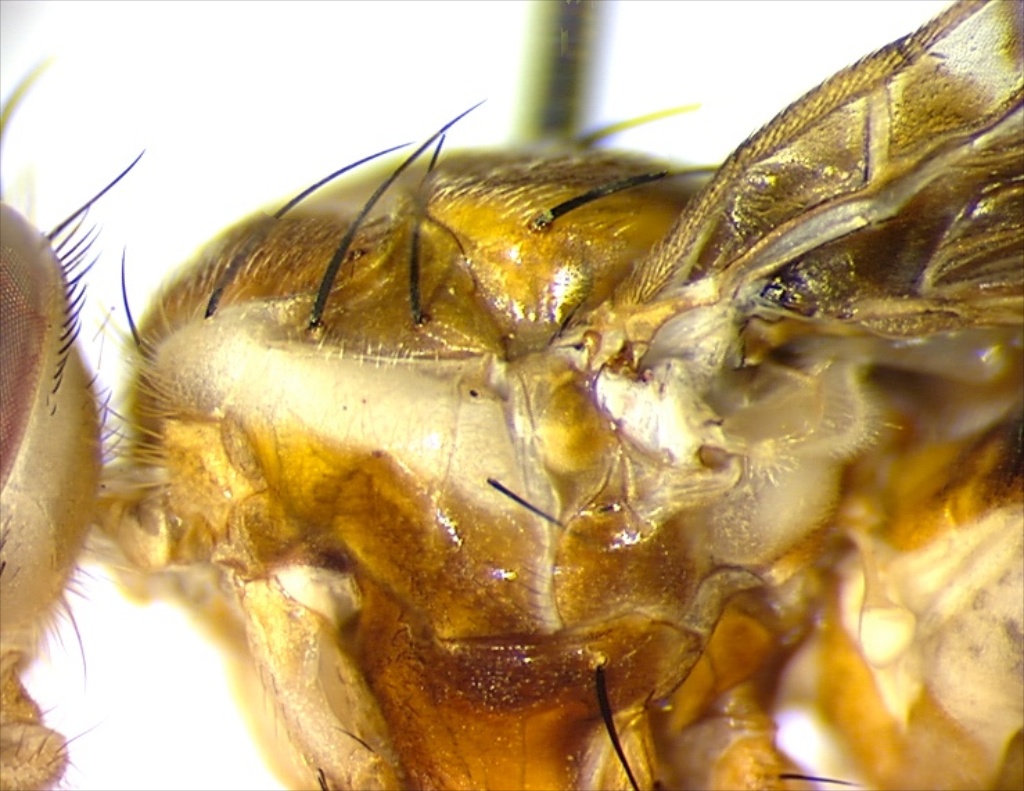

Supplement: Supplementary material 5 — Key to Carpophthoromyia [file zookeys-428-097-s005.zip › SF5_ZooKeys_key to Carpophthoromyia/key/SF5_ZooKeys_key to Carpophthoromyia/Media/Images/359 thorax lateral (automontage (c) RMCA).jpg]
