# Supplementary material for: A set of multi-entry identification keys to African frugivorous flies (Diptera, Tephritidae)
Source: Zookeys. 2014 Jul 24;(428):97–108. doi: 10.3897/zookeys.428.7366 (PMC4143993; doi:10.3897/zookeys.428.7366)
Supplement: Supplementary material 5 — Key to Carpophthoromyia [file zookeys-428-097-s005.zip › SF5_ZooKeys_key to Carpophthoromyia/key/SF5_ZooKeys_key to Carpophthoromyia/Media/Html/Carpophthoromyia debeckeri.htm]

***Carpophthoromyia debeckeri*** **De
Meyer, 2006**

 

*Carpophthoromyia debeckeri* De
Meyer, 2006: 7

 

Body length: 6.4 (6.0-6.8) mm; wing length 7.36
(7.04-7.68) mm.

 

Head. Antennal segments yellow to orange. Arista short pubescent,
longest rays less than half width of first flagellomere. Frons white to yellow,
upper part darker. Two frontals placed on almost straight line, with anterior
frontal at most 1.5 times as far from the inner eye margin than posterior
frontal; two orbitals. Face white.

 

Thorax.
Scutum shining orange-brown; black setulae, except for two broad transverse
bands with silvery setulae; one anteriorly of transverse suture, continuing
posteriorly along lateral margins to base of postsutural supraalars or almost
so, second one near dorsocentrals. Postpronotum white. Anepisternum with white
band with lower margin reaching posteroventral corner or almost so; with pale
setulae, two anepisternals. Anatergite and katatergite white. Scutellum white,
apical half with three brown spots, largely merged with area in between spots
yellow-brown. Subscutellum orange-brown.

 

Wing
(Fig. 1). Hyaline indentation in cell c, with few black patches and streaks;
also basal part of cells dm and cu2 partly hyaline. Hyaline indentation near junction of vein C with
apical part of vein R1 reaching R4+5. S-band and inverted V-band completely separate. S-band without
subapical tooth. Crossvein DM-Cu straight. R-M ratio 1.18-1.37.

 

Legs. Yellow to yellow-orange.

 

Abdomen.
Shining orange-brown, posterior margin to half of tergites 2-4 more white to
yellow, tergite 5 orange; with black setulae, tergites 2-4 with silvery setulae
and microtrichosity along white to yellow band. Spermatheca cylindrical.
Female. Terminalia, oviscape about as long as abdominal tergites; shining
orange, with black setulae. Aculeus orange, cylindrical, about 15 times longer
than wide; aculeus tip slightly downcurved.

 

(description after De Meyer, 2006)
