# Supplementary material for: A set of multi-entry identification keys to African frugivorous flies (Diptera, Tephritidae)
Source: Zookeys. 2014 Jul 24;(428):97–108. doi: 10.3897/zookeys.428.7366 (PMC4143993; doi:10.3897/zookeys.428.7366)
Supplement: Supplementary material 5 — Key to Carpophthoromyia [file zookeys-428-097-s005.zip › SF5_ZooKeys_key to Carpophthoromyia/key/SF5_ZooKeys_key to Carpophthoromyia/Media/Html/Carpophthoromyia dimidiata.htm]

***Carpophthoromyia dimidiata*** **Bezzi, 1924**

 

*Carpophthoromyia dimidiata* Bezzi, 1924a: 474.

 

Body length: 5.84 (4.64-6.40) mm; wing
length 6.12 (5.12-7.04) mm

 

Head. Antennal segments reddish brown,
sometimes darker brown; arista with medium long rays, longest ones slightly
more than half the width of first flagellomere. Frons white to yellow, upper
third (area in between orbitals to upper margin ocellar triangle) brown. Three
frontals placed on oblique line, with anterior frontal 2.5 times as far from
the inner eye margin than posterior frontal; two orbitals. Area of antennal
base with brown patches. Face white, parafacial area and gena darker brown.

 

Thorax. Scutum shining orange-brown to black-brown; black setulae,
except for one broad transverse band with silvery setulae anteriorly of
transverse suture. Postpronotum white. Anepisternum with white band with lower
margin usually reaching to halfway posterior margin of anepisternum, sometimes
reaching lower; with pale setulae, lower third with black setulae, two
anepisternals. Anatergite white, occasionally black; katatergite black, only
dorsally slightly white. Scutellum white, ventrally with brown apical spot reaching
halfway between basal and apical scutellar, variable in dorsal view, usually
apical spots as broad as in ventral view, sometimes reduced but still visible
and reaching beyond base of apical scutellars. Subscutellum black.

 

Wing (Fig. 7). Anterior margin without
hyaline indentations in cells c or sc. S-band and inverted V-band fused basally
near vein A1+Cu2 and
subapically between veins R4+5 and
M. Crossvein DM-Cu strongly sinuous. R-M ratio 1.17-1.32.

 

Legs. Orange-brown to black-brown, tibia and tarsal
segments yellow, at most basal margin of tibiae slightly darkened.

 

Abdomen. Shining black-brown; with black setulae,
tergite 2 reddish brown and with silvery setulae along posterior half;
sometimes tergites 4 and/or 5 partly more reddish brown. Spermatheca
cylindrical (Fig. 36).

 

Female.
Terminalia, oviscape at least as long as abdominal tergites; shining black
brown, with black setulae. Aculeus yellow to orange, cylindrical, about 15 to
20 times longer than wide; aculeus tip darker orange and slightly downcurved.

 

(Description after De Meyer, 2006)
