# Supplementary material for: A set of multi-entry identification keys to African frugivorous flies (Diptera, Tephritidae)
Source: Zookeys. 2014 Jul 24;(428):97–108. doi: 10.3897/zookeys.428.7366 (PMC4143993; doi:10.3897/zookeys.428.7366)
Supplement: Supplementary material 5 — Key to Carpophthoromyia [file zookeys-428-097-s005.zip › SF5_ZooKeys_key to Carpophthoromyia/key/SF5_ZooKeys_key to Carpophthoromyia/Media/Html/Carpophthoromyia dividua.htm]

***Carpophthoromyia dividua*** **De Meyer, 2006**

 

*Carpophthoromyia dividua* De Meyer, 2006: 7

 

Body length: 4.83 (4.08-5.36)mm; wing length 5.04
(4.40-6.16)mm

 

Head.
Antennal segments brown. Arista distinctly plumose; longest rays longer than
width of first flagellomere. Frons white to white-yellow, longitudinal brown
band for entire length from ocellar triangle to antennal base, equal to width
of ocellar triangle. Three frontals placed on oblique line, with anterior
frontal at least 3 times as far from the inner eye margin than posterior
frontal; two orbitals, posterior one poorly developed, rarely missing. Distance
between posterior frontal and anterior orbital is equal to or larger than
distance between anterior and posterior orbital. Face white, gena darker brown.

 

Thorax.
Scutum shining black-brown; black setulae, without transverse bands of silvery
setulae. Postpronotum white. Anepisternum with white to yellow band with lower
margin reaching to lower fourth of posterior margin; with pale setulae, lower
fourth black setulae sometimes more extensively so along posterior margin, two
anepisternals. Katatergite and anatergite both white. Scutellum white, ventrally
with 3 brown apical spots, not visible in dorsal view. Subscutellum black.

 

Wing.
Pattern similar to that of C. pseudotritea (see fig. 9). Hyaline indentation
near junction of vein C with apical part of vein R1, reaching vein
R4+5, sometimes
continuing slightly beyond vein. S-band and inverted V-band not fused;
sometimes V-band less strongly developed near subapical tooth, almost divided
in two parts. S-band with small subapical tooth. Crossvein straight or slightly
sinuous. R-M ratio 1.33-1.36.

 

Legs.
Brown, tibia, tarsal segments and extreme apex of front femur yellow, at most
basal margin of tibiae slightly darkened.

 

Abdomen. Shining black-brown, tergite 4 with median
yellow spot and/or silvery microtrichosity posteriorly; with black setulae.
Spermatheca ovoid in apical part, base slender.

 

Male.
Terminalia (Fig. 34), epandrium heart-shape in posterior view; posterior lobe
of lateral surstylus elongated and curved at apex.

 

Female.
Terminalia, oviscape shorter than abdomen; shining black-brown. Aculeus orange;
flattened, about 5 times longer than wide (Fig. 18); tip simply pointed (Fig.
23).

 

(description after De Meyer, 2006)
