# Supplementary material for: A set of multi-entry identification keys to African frugivorous flies (Diptera, Tephritidae)
Source: Zookeys. 2014 Jul 24;(428):97–108. doi: 10.3897/zookeys.428.7366 (PMC4143993; doi:10.3897/zookeys.428.7366)
Supplement: Supplementary material 5 — Key to Carpophthoromyia [file zookeys-428-097-s005.zip › SF5_ZooKeys_key to Carpophthoromyia/key/SF5_ZooKeys_key to Carpophthoromyia/Media/Html/Carpophthoromyia flavofasciata.htm]

***Carpophthoromyia flavofasciata*** **De Meyer, 2006**

 

*Carpophthoromyia flavofasciata* De Meyer, 2006: 7

 

Body length: 5.44mm; wing length 5.68mm

 

Head. Antennal segments orange. Arista
distinctly plumose; longest rays longer than width of first flagellomere. Frons
white, longitudinal brown band for entire length from ocellar triangle to
antennal base, equal to width of distance between anterior orbitals. Two or
three frontals placed on oblique line, with anterior frontal at least 3 times
as far from the inner eye margin than posterior frontal in the case of three
frontals; two orbitals. Distance between posterior frontal and anterior orbital
is shorter than distance between anterior and posterior orbital. Face white,
gena darker brown.

 

Thorax. Scutum shining black-brown, along transverse suture with
distinct yellow-white fascia; black setulae, without transverse bands of
silvery setulae. Postpronotum white. Anepisternum with white to yellow band
with lower margin reaching to lower fifth of posterior margin; with pale
setulae, lower fifth with black setulae, two anepisternals. Katatergite and
anatergite both white. Scutellum white, ventrally with 3 brown apical spots,
not visible in dorsal view. Subscutellum black.

 

Wing. Pattern similar to that of C.
pseudotritea (see fig. 9). Hyaline indentation near junction of vein C with
apical part of vein R1, reaching to vein R4+5.
S-band and inverted V-band not fused. S-band with small subapical tooth.
Crossvein DM-Cu straight. R-M ratio 1.14.

 

Legs. Brown, tibia and tarsal segments yellow.

 

Abdomen. Shining black-brown, tergite 2
and 4 along posterior half with yellow band; with black setulae, tergite 2 and
4 along postior band with white setulae and microtrichosity. Spermatheca ovoid
in apical part, base slender.

 

Female. Terminalia, oviscape shorter than abdomen;
shining black-brown. Aculeus orange, flattened (Fig. 13), not cylindrical,
about 5 times longer than wide; tip blunt, rounded with small teeth (Fig. 28).

 

(description after De Meyer, 2006)
