# Supplementary material for: A set of multi-entry identification keys to African frugivorous flies (Diptera, Tephritidae)
Source: Zookeys. 2014 Jul 24;(428):97–108. doi: 10.3897/zookeys.428.7366 (PMC4143993; doi:10.3897/zookeys.428.7366)
Supplement: Supplementary material 5 — Key to Carpophthoromyia [file zookeys-428-097-s005.zip › SF5_ZooKeys_key to Carpophthoromyia/key/SF5_ZooKeys_key to Carpophthoromyia/Media/Html/Carpophthoromyia interrupta.htm]

***Carpophthoromyia interrupta*** **De Meyer, 2006**

 

*Carpophthoromyia interrupta* De Meyer, 2006: 7

 

Body length: 4.64 (4.20-5.02)mm; wing length 4.80
(4.62-4.72)mm

 

Head. Antennal segments brown. Arista distinctly
plumose, longest rays longer than width of first flagellomere. Frons yellow;
upper third (area in between orbitals to upper margin ocellar triangle)
slightly darker yellow. Two frontals placed on slight oblique line, with
anterior frontal 1.5 times as far from the inner eye margin than posterior
frontal; two orbitals. Face white to yellow, gena brown; area near antennal
base brown.

 

Thorax.
Scutum shining black-brown, along transverse suture more pale brown; black
setulae, without transverse bands of silvery setulae. Postpronotum brown, same
ground colour as scutum. Anepisternum with white to yellow band not reaching
postpronotum, starting at level with anterior notopleural seta; lower margin
reaching lower fourth of posterior margin or almost reaching posteroventral
corner of anepisternum; with pale setulae, posteriorly with black setulae; one
anepisternal. Anatergite and katatergite brown. Scutellum white, ventrally with
3 brown apical spots, not visible in dorsal view. Subscutellum black. Wing
(Fig. 10). One hyaline indentation in cell c, without darker markings; very
deep, reaching cells bm or cu2. S-band and inverted V-band not
fused. S-band with distinct subapical tooth. Legs of inverted V-band not
touching in cell r4+5, divided in two parts (subapical band
and posterior apical band). Crossvein DM-Cu slightly sinuous. R-M ratio
1.00-1.15.

 

Legs. Brown, tarsal segments and fore tibia yellow; mid
and hind tibia brown basally, gradually paler colour with only apical third
completely yellow. Abdomen. Shining black-brown, tergite 5 with median yellow
spot; with black setulae. Spermatheca ovoid apical but based thickened (Fig.
38).

 

Female terminalia, oviscape about as long as abdominal
tergites, cylindrical; shining black-brown, with black setulae. Aculeus yellow
to orange, flat (Fig. 16), about 10 times longer than wide; aculeus tip
triangular, serrate (Fig. 31).

 

(description after De Meyer, 2006)
