# Supplementary material for: A set of multi-entry identification keys to African frugivorous flies (Diptera, Tephritidae)
Source: Zookeys. 2014 Jul 24;(428):97–108. doi: 10.3897/zookeys.428.7366 (PMC4143993; doi:10.3897/zookeys.428.7366)
Supplement: Supplementary material 5 — Key to Carpophthoromyia [file zookeys-428-097-s005.zip › SF5_ZooKeys_key to Carpophthoromyia/key/SF5_ZooKeys_key to Carpophthoromyia/Media/Html/Carpophthoromyia litterata.htm]

***Carpophthoromyia litterata*** **(Munro, 1933)**

 

*Trirhithrum litteratum*Munro, 1933: 33.

 

Body length: 7.20 (6.80-7.60)mm; wing length 7.64 (7.44-7.92)mm.

 

Head. Antennal segments yellow.
Arista short pubescent to medium long pilose, longest
rays one-third to half width of first flagellomere.
Frons yellow. Three frontals placed almost on straight line, with anterior
frontal at most 1.5 times as far from the inner eye margin than posterior
frontal; two orbitals. Face white, gena yellow.

 

Thorax. Scutum shining brown to
orange-brown; black setulae, except for two broad transverse bands with silvery
setulae: one anteriorly of transverse suture continuing posteriorly along
lateral margin to postsutural supraalars,
second partly incomplete band between dorsocentrals. Postpronotum white. Anepisternum with white to yellow band with lower margin reaching to lower
third or halfway posterior margin; with pale setulae, lower fourth to third
posteriorly with black setulae, two anepisternals.
Katatergite and anatergite white. Scutellum white, apical third with three separate brown
spots, occasionally partly merged. Subscutellum brown
to yellow-brown

 

Wing (Figs. 2-3). Hyaline indentation in cell c, with dark brown spots
and streaks. Additional hyaline indentation near junction of vein C with apical
part of vein R1, reaching R4+5. S-band with subapical tooth. S-band
and inverted V-band largely fused along cells cu2 and dm, dm and anterior part of r4+5 in male; in female partly separated in cu2 and dm. Crossvein DM-Cu straight or slightly sinuous. R-M ratio 1.86-1.88.

 

Legs. Yellow, femora darker
yellow-brown in female.

 

Abdomen. Shining brown, tergite
1 yellow-orange along anterior half; tergites 2 and 4
along posterior half to two-thirds yellow to yellow-orange, sometimes tergite 2 largely and tergite 5
in median part posteriorly yellow-orange; with black setulae, tergites 2 and 4 with silvery setulae and microtrichosity along yellow-orange band. Spermatheca
ovoid in apical part, base slender.

 

Female terminalia, oviscape shining yellow-orange; longer than abdominal tergites. Aculeus
orange, cylindrical, about 25 times longer than wide; tip simply pointed,
slightly downcurved.

 

(description after De
Meyer, 2006)
