# Supplementary material for: A set of multi-entry identification keys to African frugivorous flies (Diptera, Tephritidae)
Source: Zookeys. 2014 Jul 24;(428):97–108. doi: 10.3897/zookeys.428.7366 (PMC4143993; doi:10.3897/zookeys.428.7366)
Supplement: Supplementary material 5 — Key to Carpophthoromyia [file zookeys-428-097-s005.zip › SF5_ZooKeys_key to Carpophthoromyia/key/SF5_ZooKeys_key to Carpophthoromyia/Media/Html/Carpophthoromyia nigribasis.htm]

Microsoft Word - 360\_descr.doc


***Carpophthoromyia*** ***nigribasis* (Enderlein, 1920)**

*Ceratitis nigribasis* Enderlein, 1920: 346.

Body length: 6.40 (6.24-6.50)mm; wing
length 6.80 (6.60-6.90)mm. Head. Antennal segments red-brown. Arista medium
long pilose, longest rays about half width of first flagellomere. Frons dark
yellow. Apparently three frontals (broken and frons largely greased in type,
bases of frontals not clear; non-type specimens three frontals on oblique line,
with anterior frontal 2 times as far from the inner eye margin than posterior
frontal); two orbitals. Face white to yellow, gena brown. Thorax. Scutum
shining brown; with apparently black setulae in holotype rubbed off; in
non-type material black setulae, except for one transverse band with silvery
setulae, anteriorly of transverse suture. Postpronotum white to yellow.
Anepisternum with white to yellow band with lower margin reaching
posteroventral corner; setulae in holotype rubbed off, in non-type specimens
white to yellow band reaching lower fourth of posterior margin, pale setulae,
along lower fourth few black setulae; two anepisternals. Katatergite and
anatergite white to yellow. Scutellum completely white to yellow, in non-type
specimens ventrally with small brownish patch between apical scutellars.
Subscutellum black-brown. Wing. Similar to pattern observed in C. pseudotritea
(see Fig. 9). Hyaline indentation near junction of vein C with apical part of
vein R1, reaching R4+5.
S-band and inverted V-band separate (in non-type material touching basally in
cell cu2); S-band with subapical tooth. Crossvein DM-Cu slightly sinuous (in
non-type material more strongly so). R-M ratio 1.33-1.65. Legs black-brown,
tibiae and tarsal segments yellow. Abdomen. Shining brown, tergite 2 along
posterior half to two-thirds yellow to yellow-orange, tergites 4 and 5
posteriorly more orange-brown; with black setulae, apparently tergite 2 silvery
setulae and microtrichosity along yellow-orange band. Female terminalia,
oviscape shining black-brown; about as long as abdominal tergites; cylindrical.
Aculeus not dissected, hidden in holotype.

(description after De Meyer,
2006)
