# Supplementary material for: A set of multi-entry identification keys to African frugivorous flies (Diptera, Tephritidae)
Source: Zookeys. 2014 Jul 24;(428):97–108. doi: 10.3897/zookeys.428.7366 (PMC4143993; doi:10.3897/zookeys.428.7366)
Supplement: Supplementary material 5 — Key to Carpophthoromyia [file zookeys-428-097-s005.zip › SF5_ZooKeys_key to Carpophthoromyia/key/SF5_ZooKeys_key to Carpophthoromyia/Media/Html/Carpophthoromyia procera.htm]

Microsoft Word - 361\_descr.doc


***Carpophthoromyia procera*** **(Enderlein, 1920)**

*Ceratitis procera* Enderlein, 1920: 345.

Body length: 7.86 (7.20-8.64)mm; wing
length 7.80 (6.40-8.40)mm. Head. Antennal segments yellow to orange. Arista
medium long pilose, longest rays usually slightly more than half the width of
first flagellomere but never equal to width. Frons yellow, upper third (area in
between orbitals to upper margin ocellar triangle) brown. Three frontals placed
on oblique line, with anterior frontal 1.5-2 times as far from the inner eye
margin than posterior frontal; two orbitals. Face white, gena dark brown.
Thorax. Scutum shining black-brown; black setulae, except for two broad
transverse band with silvery setulae; one anteriorly of transverse suture,
continuing posteriorly along lateral margins to base of postsutural supraalars
or slightly beyond, second one near dorsocentrals; third smaller region with
silvery setulae between postpronotal lobes. Postpronotum white. Anepisternum
with white band with lower margin reaching posteroventral corner or almost so;
with pale setulae, lower fourth with black setulae, two anepisternals.
Anatergite and katatergite white. Scutellum completely white. Subscutellum
black-brown. Wing (Fig. 4). Hyaline indentation in cell c, with black patches
and streaks. Hyaline indentation near junction of vein C with apical part of
vein R1, reaching R4+5.
S-band and inverted V-band completely separate. S-band with small trace of
subapical tooth, rarely (one specimen: Lolodorf locality in MNHU collection)
tooth extending to inverted V-band. Crossvein DM-Cu slightly sinuous. R-M ratio
1.35-1.70. Legs. Reddish brown, tibia and tarsal segments yellow. Abdomen.
Shining brown, posterior half of tergites 2-4 more yellow-orange, sometimes
tergites 1-2 largely or completely orange; with black setulae, tergites 2-4
with silvery setulae and microtrichosity along yellow-orange band; tergite 5
more reddish brown. Spermatheca ovoid in apical part, base slender. Female
terminalia, oviscape at least as long as abdominal tergites; shining brown,
with black setulae. Aculeus orange, cylindrical, about 10 times longer than
wide; aculeus tip darker orange and slightly downcurved.

(description after De Meyer,
2006)

This document was
created with Win2PDF available at http://www.daneprairie.com. The unregistered
version of Win2PDF is for evaluation or non-commercial use only.
