# Supplementary material for: A set of multi-entry identification keys to African frugivorous flies (Diptera, Tephritidae)
Source: Zookeys. 2014 Jul 24;(428):97–108. doi: 10.3897/zookeys.428.7366 (PMC4143993; doi:10.3897/zookeys.428.7366)
Supplement: Supplementary material 5 — Key to Carpophthoromyia [file zookeys-428-097-s005.zip › SF5_ZooKeys_key to Carpophthoromyia/key/SF5_ZooKeys_key to Carpophthoromyia/Media/Html/Carpophthoromyia pseudotritea.htm]

Microsoft Word - 362\_descr.doc


***Carpophthoromyia*** ***pseudotritea* Bezzi, 1918**

*Carpophthoromyia pseudotritea* Bezzi, 1918: 225.

Body length: 4.87 (4.00-6.00)mm; wing
length 4.93 (4.00-5.89)mm. Head. Antennal segments dark yellow to brown. Arista
distinctly plumose; longest rays longer than width of first flagellomere. Frons
white to yellow, longitudinal yellow-brown band for entire length from ocellar
triangle to antennal base, equal to width between anterior orbitals. Three
frontals placed on oblique line, with anterior frontal at least 3 times as far
from the inner eye margin than posterior frontal; two orbitals, posterior one shorter
than anterior one but well developed. Distance between posterior frontal and
anterior orbital distinctly shorter than distance between anterior and
posterior orbital. Face white to yellow, gena darker brown. Thorax. Scutum
shining black-brown, along transverse suture often yellow-brown, sometimes with
faint yellow fascia; black setulae, without transverse bands of silvery
setulae. Postpronotum white. Anepisternum with white to yellow band with lower
margin reaching to lower fourth of posterior margin; with pale setulae, lower
fourth black setulae sometimes more extensively so along posterior margin, two
anepisternals. Katatergite and anatergite both white. Scutellum white,
ventrally with 3 brown apical spots, not visible in dorsal view. Subscutellum black.
Wing (Fig. 9). Hyaline indentation near junction of vein C with apical part of
vein R1, reaching vein R4+5, sometimes continuing slightly beyond vein. S-band and inverted
V-band not fused; rarely V-band less strongly developed near subapical tooth,
almost divided in two parts. S-band with small subapical tooth. Crossvein DM-Cu
straight or slightly sinuous. R-M ratio 1.40. Legs. Brown, tibia, tarsal
segments and extreme apex of front femur yellow, at most basal margin of tibiae
slightly darkened. Abdomen. Shining black-brown, tergite 4 with median yellow
spot and/or silvery microtrichosity posteriorly; with black setulae.
Spermatheca ovoid in apical part, base slender. Female terminalia, oviscape
shorter than abdomen; shining blackbrown. Aculeus orange, flattened, about 5
times longer than wide (Fig. 20); tip simply pointed (Fig. 24). Male terminalia
(Fig. 35), epandrium rounded in posterior view; posterior lobe of lateral
surstylus elongated and almost straight; medial surstylus at apex pointed.

(description after De Meyer,
2006)
