# Supplementary material for: A set of multi-entry identification keys to African frugivorous flies (Diptera, Tephritidae)
Source: Zookeys. 2014 Jul 24;(428):97–108. doi: 10.3897/zookeys.428.7366 (PMC4143993; doi:10.3897/zookeys.428.7366)
Supplement: Supplementary material 5 — Key to Carpophthoromyia [file zookeys-428-097-s005.zip › SF5_ZooKeys_key to Carpophthoromyia/key/SF5_ZooKeys_key to Carpophthoromyia/Media/Html/Carpophthoromyia radulata.htm]

Microsoft Word - 363\_descr.doc


***Carpophthoromyia radulata*****De Meyer, 2006**

*Carpophthoromyia radulata* De Meyer, 2006: 7

Body length: 5.20mm; wing length 5.20mm.
Head. Antennal segments brown. Arista distinctly plumose; longest rays longer
than width of first flagellomere. Frons white to yellow, longitudinal brown
band for entire length from ocellar triangle to antennal base, equal to width
of distance between anterior orbitals. Three frontals placed on oblique line,
with anterior frontal at least 3 times as far from the inner eye margin than
posterior frontal; two orbitals. Distance between posterior frontal and
anterior orbital is shorter than distance between anterior and posterior
orbital. Face white, gena darker brown. Thorax. Scutum shining black-brown,
along transverse suture yellow-brown; black setulae, without transverse bands
of silvery setulae. Postpronotum white. Anepisternum with white to yellow band
with lower margin reaching to lower fourth of posterior margin; with pale
setulae, lower fourth with black setulae, two anepisternals. Katatergite and
anatergite both white. Scutellum white, ventrally with 3 brown apical spots,
not visible in dorsal view. Subscutellum black. Wing. Pattern similar to that
of C. pseudotritea (see fig. 9). Hyaline indentation near junction of vein C
with apical part of vein R1, reaching well beyond vein R4+5, to halfway between R4+5 and M.
S-band and inverted V-band not fused. S-band with small subapical tooth.
Crossvein DM-Cu straight. R-M ratio 1.15. Legs. Brown, tibia and tarsal
segments yellow. Abdomen. Shining black-brown; with black setulae. Spermatheca
ovoid in apical part, base slender. Male. Unknown Female. Terminalia, oviscape
shorter than abdomen; shining black-brown. Aculeus orange, flattened (Fig. 14),
about 5 times longer than wide; tip truncate with small protuberances (Fig.
29).

(description after De Meyer,
2006)
