# Supplementary material for: A set of multi-entry identification keys to African frugivorous flies (Diptera, Tephritidae)
Source: Zookeys. 2014 Jul 24;(428):97–108. doi: 10.3897/zookeys.428.7366 (PMC4143993; doi:10.3897/zookeys.428.7366)
Supplement: Supplementary material 5 — Key to Carpophthoromyia [file zookeys-428-097-s005.zip › SF5_ZooKeys_key to Carpophthoromyia/key/SF5_ZooKeys_key to Carpophthoromyia/Media/Html/Carpophthoromyia schoutedeni.htm]

Microsoft Word - 364\_descr.doc


***Carpophthoromyia
schoutedeni*** **De Meyer, 2006**

*Carpophthoromyia schoutedeni* De Meyer, 2006: 7

Body length: 4.32 (4.24-4.40)mm; wing
length 5.08 (4.96-5.41)mm. Head. Antennal segments orange. Arista distinctly
plumose, longest rays longer than width of first flagellomere. Frons white to
yellow. Two frontals placed on oblique line, with anterior frontal 1.5 times as
far from the inner eye margin than posterior frontal; two orbitals. Face white
to yellow. Thorax. Scutum shining black-brown, along transverse suture more
yellow-brown; pale setulae. Postpronotum white. Anepisternum with white band,
lower margin reaching posteroventral corner; with pale setulae, posterior
margin in male with few longer black setulae; one anepisternal. Anatergite and
katatergite white. Scutellum white, ventrally with 3 brown apical spots, not
visible in dorsal view. Subscutellum black. Wing (Fig. 11). One hyaline
indentation in cell c, with singly dark spot; very deep, reaching cells bm or
bcu. S-band and inverted V-band not fused. S-band with subapical tooth.
Crossvein DM-Cu slightly sinuous. R-M ratio 1.20-1.24. Legs yellow; fore femur
yellow, mid and hind femora brown, basally darker yellow. Abdomen. Shining
black-brown, tergites 1-2 largely yellow with brown patches, tergites 3-5 with
median yellow spot; with mixed black and pale setulae, tergite 4 along
posterior half with silvery microtrichosity. Spermatheca ovoid in apical part,
base slender (Fig. 37). Female terminalia, oviscape about as long as abdominal
tergites, cylindrical; shining black-brown, with black setulae. Aculeus yellow
to orange, flattened (Fig. 17), about 20 times longer than wide; aculeus tip
triangular, serrate; below serrated tip with small lateral notches (Fig. 32).

(description after De
Meyer, 2006)
