# Supplementary material for: A set of multi-entry identification keys to African frugivorous flies (Diptera, Tephritidae)
Source: Zookeys. 2014 Jul 24;(428):97–108. doi: 10.3897/zookeys.428.7366 (PMC4143993; doi:10.3897/zookeys.428.7366)
Supplement: Supplementary material 5 — Key to Carpophthoromyia [file zookeys-428-097-s005.zip › SF5_ZooKeys_key to Carpophthoromyia/key/SF5_ZooKeys_key to Carpophthoromyia/Media/Html/Carpophthoromyia scutellata.htm]

Microsoft Word - 365\_descr.doc


***Carpophthoromyia******scutellata*** **(Walker, 1853)**

*Trypeta scutellata* Walker, 1853: 384

Body
length: 7.29 (6.40-8.48)mm; wing length 7.08 (6.40-7.80)mm. Head. Antennal
segments yellow to orange. Arista short to medium long pilose, longest rays
about half width of first flagellomere. Frons usually completely yellow. Three
frontals placed on slight oblique line, with anterior frontal at most

1.5 times as far from
the inner eye margin than posterior frontal; two orbitals. Face white, gena
dark yellow. Thorax. Scutum shining black-brown; black setulae, except for two
broad transverse band with silvery setulae; one anteriorly of transverse
suture, continuing posteriorly along lateral margins to base of postsutural
supraalars or slightly beyond, second one near dorsocentrals; third smaller
region of silvery setulae between postpronotal lobes; sometimes bands partly
fused. Postpronotum white. Anepisternum with white band with lower margin
reaching lower fourth of posterior margin; with pale setulae, lower fourth with
black setulae, two anepisternals. Anatergite and katatergite white. Scutellum
completely white. Subscutellum black-brown. Wing. Pattern similar to that of C.
procera (see fig. 4). Hyaline indentation in cell c, with black patches and
streaks. Hyaline indentation near junction of vein C with apical part of vein R1, reaching R4+5. S-band and inverted
V-band completely separate. S-band with small trace of subapical tooth, or
tooth completely absent. Crossvein DM-Cu straight or slightly sinuous. R-M
ratio 1.33-1.67. Legs. Completely yellow in male; female with femora brownish,
except fore femur yellow anteriorly. Abdomen. Shining brown, posterior half of
tergites 2-4 more yellow-orange, sometimes tergites 1-2 largely or completely
orange; with black setulae, tergites 2-4 with silvery setulae and
microtrichosity along yellow-orange band; tergite 5 more reddish brown.
Spermatheca ovoid in apical part, base slender. Female terminalia, oviscape at
least as long as abdominal tergites; shining brown, with black setulae. Aculeus
orange, cylindrical, about 25 times longer than wide; aculeus tip darker orange
and slightly downcurved.

(description
after De Meyer, 2006)
