# Supplementary material for: A set of multi-entry identification keys to African frugivorous flies (Diptera, Tephritidae)
Source: Zookeys. 2014 Jul 24;(428):97–108. doi: 10.3897/zookeys.428.7366 (PMC4143993; doi:10.3897/zookeys.428.7366)
Supplement: Supplementary material 5 — Key to Carpophthoromyia [file zookeys-428-097-s005.zip › SF5_ZooKeys_key to Carpophthoromyia/key/SF5_ZooKeys_key to Carpophthoromyia/Media/Html/Carpophthoromyia speciosa.htm]

Microsoft Word - 366\_descr.doc


***Carpophthoromyia*** ***speciosa* Hancock, 1984**

*Carpophthoromyia speciosa* Hancock, 1984 : 291.

Body length: 5.82 (5.04-6.24)mm; wing
length 6.25 (5.44-6.72)mm. Head. Antennal segments red-brown. Arista with short
pubescent to medium long pilose, longest rays at most half the width of first
flagellomere. Frons yellow, upper third (area in between orbitals to upper
margin ocellar triangle) brown; near antennal base with brown patches,
sometimes brown patches more pronounced and extending along frontals. Three
frontals placed on slight oblique line, with anterior frontal 1.5-2 times as
far from the inner eye margin than posterior frontal; two orbitals. Face white
to yellow, parafacial area and gena darker brown. Thorax. Scutum shining
black-brown; black setulae, except for two broad transverse bands with silvery
setulae, one anteriorly of transverse suture continuing posteriorly along
lateral margin to postsutural supraalars, second at dorsocentrals. Postpronotum
white to yellow. Anepisternum with white to yellow band; reaching lower fifth
of posterior margin; pale setulae, along lower fifth few black setulae, two
anepisternals. Katatergite and anatergite white to yellow. Scutellum white to
yellow, apical third with three black separate or merged spots. Subscutellum
black. Wing (Fig. 5). Basal part with some paler streaks but not clear hyaline
indentation. Hyaline indentation near junction of vein C with apical part of
vein R1 reaching R4+5.
S-band and inverted V-band fused basally and subapically, creating a hyaline
spot that runs obliquely from cu2, over posterior part of dm and narrowly
into r4+5. No subapical tooth. Crossvein DM-Cu
almost straight. R-M ratio 1.57-1.59. Legs. Reddish brown; tibiae and tarsal
segments yellow. Abdomen. Shining black-brown; tergites 2 and 4 yellow to
orange along posterior third to half; black setulae, tergites 2 and 4 with silvery
setulae and microtrichosity along yellow-orange band; Tergite 5 more reddish
brown along apical two-thirds. Spermatheca cylindrical. Female terminalia,
oviscape orange-red to red-brown, as long as abdominal tergites; aculeus
orange-red, stout, about 15 times longer than wide, cylindrical, tip simply
pointed, slightly downcurved.

(description after De Meyer,
2006)
