# Supplementary material for: A set of multi-entry identification keys to African frugivorous flies (Diptera, Tephritidae)
Source: Zookeys. 2014 Jul 24;(428):97–108. doi: 10.3897/zookeys.428.7366 (PMC4143993; doi:10.3897/zookeys.428.7366)
Supplement: Supplementary material 5 — Key to Carpophthoromyia [file zookeys-428-097-s005.zip › SF5_ZooKeys_key to Carpophthoromyia/key/SF5_ZooKeys_key to Carpophthoromyia/Media/Html/Carpophthoromyia tessmanni.htm]

Microsoft Word - 367\_descr.doc


***Carpophthoromyia tessmanni*** **(Enderlein, 1920)**

*Ceratitis tessmanni* Enderlein, 1920: 345.

Body length: 4.86 (4.32-5.44)mm; wing
length 5.04 (4.72-5.68)mm. Head. Antennal segments orange. Arista distinctly
plumose, longest rays longer than width of first flagellomere. Frons yellow.
Two frontals, sometimes third, poorly developed, anterior one; placed on
oblique line with anterior frontal 3 times as far from the inner eye margin
than posterior frontal; two orbitals. Face white to yellow, sometimes gena
darker yellow. Thorax. Scutum shining black-brown, along transverse suture
sometimes more yellow; black setulae, except for one broad transverse band with
silvery setulae anteriorly of transverse suture; sometimes silvery setulae
extended anteriorly along centre of presutural area; transverse suture with
yellow fascia. Postpronotum white. Anepisternum largely white to yellow, only
lower fourth dark yellow to yellow-brown; with pale setulae, ventral fourth
posteriorly with black setulae; two anepisternals. Anatergite and katatergite
white. Scutellum white, ventrally with 3 brown apical spots, not visible in
dorsal view. Subscutellum black. Wing. (Fig. 6). Basal part with streaks and
patches (especially near base of cell dm). One hyaline indentation near
junction of vein C with apical part of vein R1;
reaching halfway R4+5 and M. S-band and inverted V-band not
fused. S-band with subapical tooth, sometimes small. Inverted V-band partly
separated anteriorly in male. Crossvein DM-Cu straight. R-M ratio 1.30-1.39.
Legs. Yellow; in female femora red-brown. Abdomen. Shining black-brown, with
black setulae. Tergite 2 posteriorly more orange, tergite 4 with median yellow
spot posteriorly and with white setulae and microtrichosity on spot.
Spermatheca ovoid in apical part, base slender. Female terminalia, oviscape
about as long as abdominal tergites; shining black-brown, with black setulae.
Aculeus yellow to orange, flattened; about 8 times longer than wide (Fig. 19),
aculeus tip simply pointed (Fig. 25), straight.

(description after De Meyer,
2006)
