# Supplementary material for: A set of multi-entry identification keys to African frugivorous flies (Diptera, Tephritidae)
Source: Zookeys. 2014 Jul 24;(428):97–108. doi: 10.3897/zookeys.428.7366 (PMC4143993; doi:10.3897/zookeys.428.7366)
Supplement: Supplementary material 5 — Key to Carpophthoromyia [file zookeys-428-097-s005.zip › SF5_ZooKeys_key to Carpophthoromyia/key/SF5_ZooKeys_key to Carpophthoromyia/Media/Html/Carpophthoromyia tritea.htm]

Microsoft Word - 368\_descr.doc


***Carpophthoromyia*** ***tritea* (Walker, 1849)**

*Trypeta tritea*
Walker, 1849: 1034.

Body length: 3.87 (3.68-4.00)mm; wing
length 4.27 (4.16-4.32)mm. Head. Antennal segments brown. Arista distinctly
plumose, longest rays longer than width of first flagellomere. Frons yellow;
upper third (area in between orbitals to upper margin ocellar triangle)
sometimes darker yellow. Two frontals placed on oblique line, with anterior
frontal 2 times as far from the inner eye margin than posterior frontal; two
orbitals. Face white to yellow, gena and sometimes antennal groove brown; near
antennal base darker yellow to brown patches. Thorax. Scutum shining
black-brown, along transverse suture more yellow-brown; black setulae, without
transverse bands of silvery setulae. Postpronotum pale brown, slightly paler in
ground colour than scutum. Anepisternum with white to yellow band not reaching
postpronotum, starting at level with anterior notopleural seta; lower margin to
lower third of posterior margin of anepisternum; with pale setulae, ventrally
and posteriorly with black setulae; one anepisternal. Anatergite and
katatergite brown. Scutellum white, ventrally with 3 brown apical spots, not
visible in dorsal view. Subscutellum black. Wing (Fig. 12). One hyaline
indentation in cell c, without darker markings; very deep, reaching cells bm or
cu2. S-band and inverted V-band not fused.
S-band with very small subapical tooth. Crossvein DM-Cu straight or slightly
sinuous. R­M ratio 0.91-0.94. Legs. Brown, tarsal segments and fore tibia
yellow; mid and hind tibia brown basally, gradually paler colour with only
apical third completely yellow. Abdomen. Shining black-brown, tergite 5 with
median yellow spot, posteriorly wider; with black setulae. Spermatheca ovoid in
apical part, base slender. Female terminalia, oviscape about as long as
abdominal tergites, cylindrical; shining black-brown, with black setulae.
Aculeus yellow to orange, slender (Fig. 15), flat, about 20 times longer than
wide; aculeus tip straight, pointed, and serrate (Fig. 33).

(description after De Meyer,
2006)
