# Supplementary material for: A set of multi-entry identification keys to African frugivorous flies (Diptera, Tephritidae)
Source: Zookeys. 2014 Jul 24;(428):97–108. doi: 10.3897/zookeys.428.7366 (PMC4143993; doi:10.3897/zookeys.428.7366)
Supplement: Supplementary material 5 — Key to Carpophthoromyia [file zookeys-428-097-s005.zip › SF5_ZooKeys_key to Carpophthoromyia/key/SF5_ZooKeys_key to Carpophthoromyia/Media/Html/Carpophthoromyia virgata.htm]

Microsoft Word - 369\_descr.doc


***Carpophthoromyia virgata*** **De Meyer, 2006**

*Carpophthoromyia virgata* De Meyer, 2006: 7

Body length: 5.20 (5.00-5.40)mm; wing
length 5.85 (5.80-5.90)mm. Head. Antennal segments yellow-brown to dark brown.
Arista distinctly plumose; longest rays longer than width of first
flagellomere. Frons white to yellow, longitudinal brown band for entire length
from ocellar triangle to antennal base, equal to width of ocellar triangle. Two
frontals placed on oblique line, with anterior frontal about twice as far from
the inner eye margin than posterior frontal; two orbitals. Distance between
posterior frontal and anterior orbital is equal to distance between anterior
and posterior orbital. Face white, gena darker brown.

Thorax.
Scutum shining black-brown, along transverse suture with obscure yellow-white
fascia; black setulae, with transverse band of silvery setulae along transverse
suture. Postpronotum white. Anepisternum with white to yellow band with lower
margin reaching to lower fifth of posterior margin; with pale setulae, lower
fourth with black setulae, two anepisternals. Katatergite and anatergite both
white. Scutellum white, ventrally with 3 brown apical spots, not visible in
dorsal view. Subscutellum black. Wing. Pattern similar to that of C.
pseudotritea (see fig. 9). Hyaline indentation near junction of vein C with
apical part of vein R1, reaching to vein R4+5. S-band and inverted V-band not fused. S-band with small
subapical tooth. Crossvein DM-Cu straight. R-M ratio 1.44. Legs brown, tibia
and tarsal segments yellow. Abdomen. Shining black-brown, tergite 4 along
posterior half with yellow fascia medially, narrowing laterally and not
reaching lateral margins; with black setulae, tergite 4 with white setulae
medially. Spermatheca ovoid in apical part, base slender. Female terminalia,
oviscape shorter than abdomen; shining black-brown. Aculeus orange, flattened
(Fig. 21), about 5 times longer than wide; tip with lateral protuberances (Fig.
30).

(description after De
Meyer, 2006)
