# Supplementary material for: A set of multi-entry identification keys to African frugivorous flies (Diptera, Tephritidae)
Source: Zookeys. 2014 Jul 24;(428):97–108. doi: 10.3897/zookeys.428.7366 (PMC4143993; doi:10.3897/zookeys.428.7366)
Supplement: Supplementary material 5 — Key to Carpophthoromyia [file zookeys-428-097-s005.zip › SF5_ZooKeys_key to Carpophthoromyia/key/SF5_ZooKeys_key to Carpophthoromyia/Media/Html/Carpophthoromyia vittata.htm]

Microsoft Word - 370\_descr.doc


***Carpophthoromyia vittata*** **(Fabricius, 1794)**

*Musca vittata*
Fabricius, 1794: 355.

Body length: 6.01 (5.20-6.80)mm; wing
length 6.27 (5.20-7.12)mm. Head. Antennal segments reddish brown. Arista with
medium long rays, longest ones slightly more than half the width of first
flagellomere. Frons yellow, upper third (area in between orbitals to upper
margin ocellar triangle) sometimes brown, occasionally frons completely reddish
brown. Three frontals placed on oblique line, with anterior frontal 2.5 times
as far from the inner eye margin than posterior frontal; two orbitals. Face
white to yellow, upper margin near antennal base and sometimes gena darker
brown. Thorax. Scutum shining black-brown; black setulae, except for one broad
transverse band with silvery setulae anteriorly of transverse suture, median
part of transverse band broader. Postpronotum white to yellow. Anepisternum
with white to yellow band with lower margin reaching posteroventral corner or
almost so; with pale setulae, lower margin to lower third with black setulae,
two anepisternals. Katatergite black, anatergite largely white, sometimes both
white. Scutellum white to yellow, ventrally with brown apical spot, not passing
beyond apical scutellars and not visible in dorsal view, sometimes divided
medially and/or hardly discernible. Subscutellum black. Wing (Fig. 8). Anterior
margin without hyaline indentations in cells c or sc. S-band and inverted
V-band fused basally near vein A1+Cu2; separate
subapically between veins R4+5 and M. S-band with subapical tooth.
Crossvein DM-Cu strongly sinuous. R-M ratio 1.12-1.45. Legs dark brown to
reddish brown, tibia and tarsal segments yellow, at most basal margin of tibia
slightly darkened. Abdomen. Shining black-brown; with black setulae, tergite 2
with brown patches and/or silvery setulae along posterior half. Spermatheca
cylindrical. Female terminalia: oviscape as long as abdomen; shining black
brown, with black setulae. Aculeus yellow to orange, cylindrical, about 10 to
20 times longer than wide (Fig. 22); aculeus tip darker orange and slightly
downcurved (Figs. 26­27).

(description after De Meyer,
2006)
