# Supplementary material for: A set of multi-entry identification keys to African frugivorous flies (Diptera, Tephritidae)
Source: Zookeys. 2014 Jul 24;(428):97–108. doi: 10.3897/zookeys.428.7366 (PMC4143993; doi:10.3897/zookeys.428.7366)
Supplement: Supplementary material 5 — Key to Carpophthoromyia [file zookeys-428-097-s005.zip › SF5_ZooKeys_key to Carpophthoromyia/key/SF5_ZooKeys_key to Carpophthoromyia/Media/Html/desc_Carpophthoromyia_dimidiata.html]

Natural Language Description


# A set of multi-entry identification keys to African frugivorous flies (Diptera, Tephritidae)

### Massimiliano Virgilio, Ian White, Marc De Meyer

## Carpophthoromyia dimidiata

(key to Carpophthoromyia) sex male or female. (key to Carpophthoromyia) head (key to Carpophthoromyia) 1. frontal setae three, (key to Carpophthoromyia) 2. frons longitudinal brown band absent, (key to Carpophthoromyia) 3. head setae distance posterior frontal - anterior orbital setae shorter than distance anterior - posterior orbital setae. (key to Carpophthoromyia) thorax (key to Carpophthoromyia) 4. postpronotal lobe completely white, (key to Carpophthoromyia) 5. scutellum white with one apical spot, (key to Carpophthoromyia) 7. scutellum (one apical spot) single apical spot extending beyond apical scutellar setae, (key to Carpophthoromyia) 8. transverse band(s) of silvery setulae along transverse suture present, (key to Carpophthoromyia) 9. yellow fasciae on transverse suture absent. (key to Carpophthoromyia) wings (key to Carpophthoromyia) 10. anterior apical band complete;, (key to Carpophthoromyia) 11. crossvein dm-cu sinusoid, (key to Carpophthoromyia) 12. V-band and S-band linked in middle of cell r4+5, (key to Carpophthoromyia) 13. inverted V-band complete. (key to Carpophthoromyia) abdomen (key to Carpophthoromyia) 14. (females) aculeus tip simply pointed, (key to Carpophthoromyia) 17. (females) aculeus shape rounded-cylindrical.
