# Supplementary material for: A set of multi-entry identification keys to African frugivorous flies (Diptera, Tephritidae)
Source: Zookeys. 2014 Jul 24;(428):97–108. doi: 10.3897/zookeys.428.7366 (PMC4143993; doi:10.3897/zookeys.428.7366)
Supplement: Supplementary material 5 — Key to Carpophthoromyia [file zookeys-428-097-s005.zip › SF5_ZooKeys_key to Carpophthoromyia/key/SF5_ZooKeys_key to Carpophthoromyia/Media/Html/desc_Carpophthoromyia_flavofasciata.html]

Natural Language Description


# A set of multi-entry identification keys to African frugivorous flies (Diptera, Tephritidae)

### Massimiliano Virgilio, Ian White, Marc De Meyer

## Carpophthoromyia flavofasciata

(key to Carpophthoromyia) sex male or female. (key to Carpophthoromyia) head (key to Carpophthoromyia) 1. frontal setae three, (key to Carpophthoromyia) 2. frons longitudinal brown band present, (key to Carpophthoromyia) 3. head setae distance posterior frontal - anterior orbital setae shorter than distance anterior - posterior orbital setae. (key to Carpophthoromyia) thorax (key to Carpophthoromyia) 4. postpronotal lobe completely white, (key to Carpophthoromyia) 5. scutellum white with three apical spots, (key to Carpophthoromyia) 6. scutellum (three apical spots) apical spots not visible in dorsal view, restricted to ventral surface, (key to Carpophthoromyia) 8. transverse band(s) of silvery setulae along transverse suture absent, (key to Carpophthoromyia) 9. yellow fasciae on transverse suture present. (key to Carpophthoromyia) wings (key to Carpophthoromyia) 10. anterior apical band with hyaline indentation(s), (key to Carpophthoromyia) 11. crossvein dm-cu straight, (key to Carpophthoromyia) 12. V-band and S-band not touching in cell r4+5 or dm, (key to Carpophthoromyia) 13. inverted V-band complete, (key to Carpophthoromyia) 18. anterior apical band with hyaline indentation near junction C and apical part of vein R1. (key to Carpophthoromyia) abdomen (key to Carpophthoromyia) 14. (females) aculeus tip with ornamentations, (key to Carpophthoromyia) 15. (females) ornamented aculeus tip not serrated, other ornamentation, (key to Carpophthoromyia) 16. (females) non serrated ornament dentated apically, (key to Carpophthoromyia) 17. (females) aculeus shape flattened.
