# Supplementary figures and images for: A set of multi-entry identification keys to African frugivorous flies (Diptera, Tephritidae) (part 2 of 6)
Source: Zookeys. 2014 Jul 24;(428):97–108. doi: 10.3897/zookeys.428.7366 (PMC4143993; doi:10.3897/zookeys.428.7366)

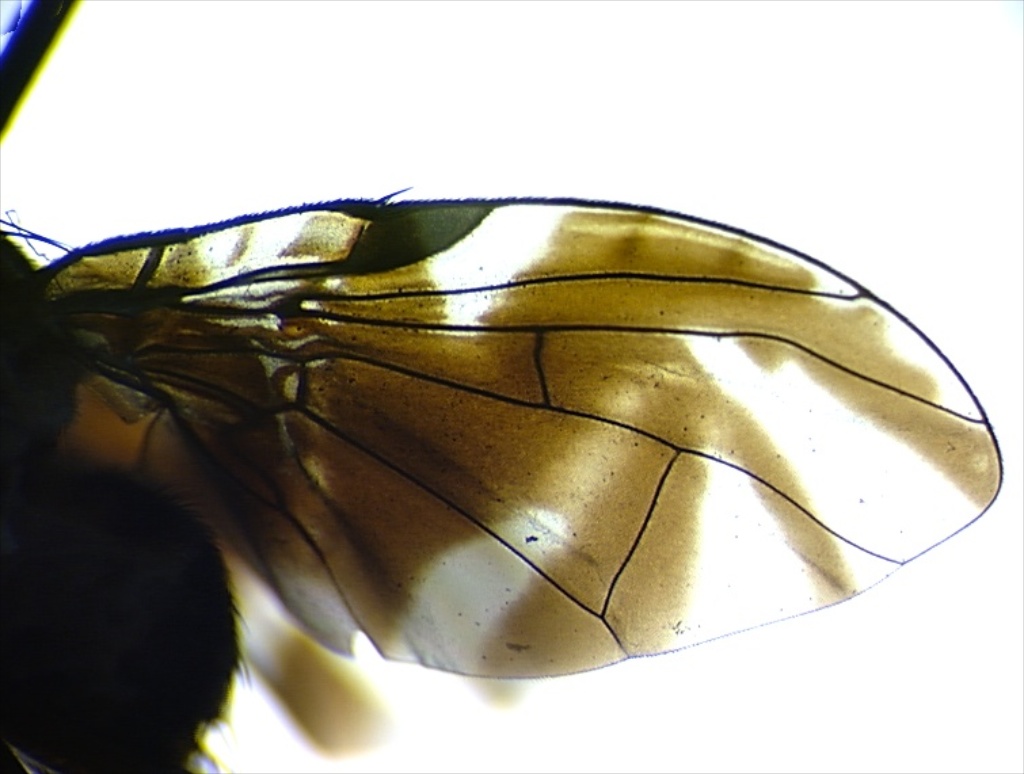

Supplement: Supplementary material 5 — Key to Carpophthoromyia [file zookeys-428-097-s005.zip › SF5_ZooKeys_key to Carpophthoromyia/key/SF5_ZooKeys_key to Carpophthoromyia/Media/Images/359 wing female dorsal (automontage (c) RMCA).jpg]

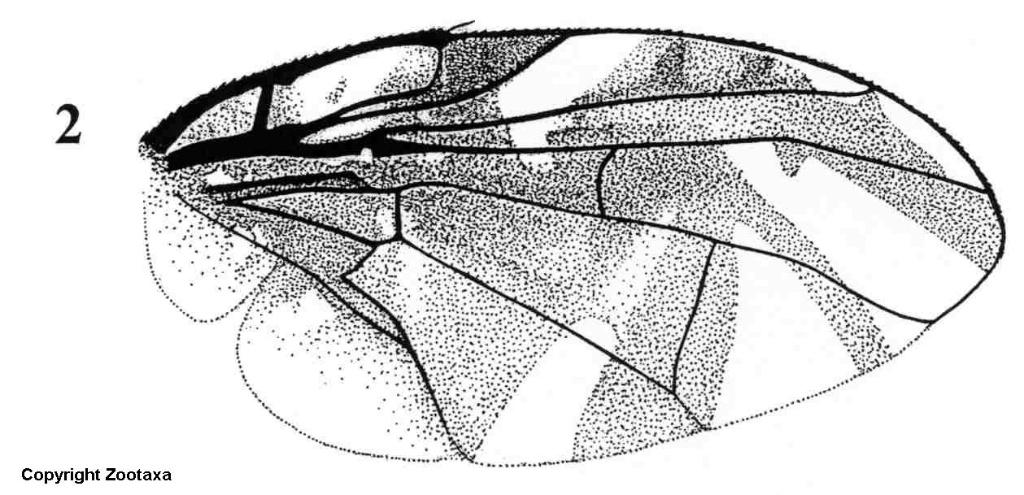

Supplement: Supplementary material 5 — Key to Carpophthoromyia [file zookeys-428-097-s005.zip › SF5_ZooKeys_key to Carpophthoromyia/key/SF5_ZooKeys_key to Carpophthoromyia/Media/Images/359 wing female dorsal (drawing (c) Zootaxa).jpg]

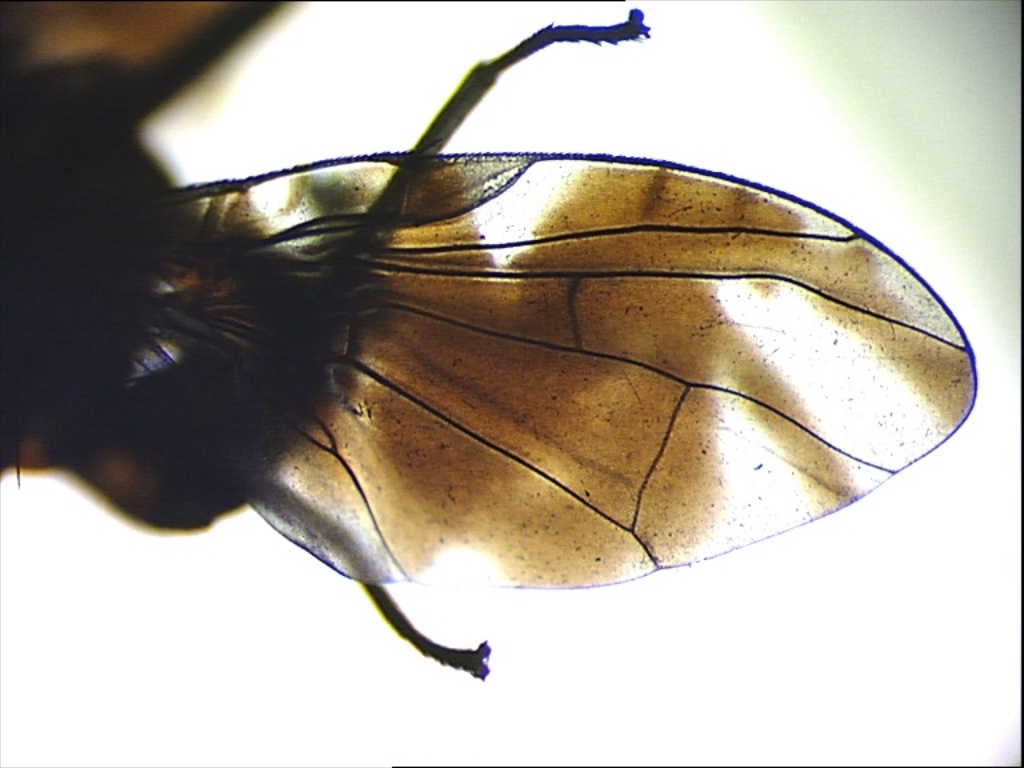

Supplement: Supplementary material 5 — Key to Carpophthoromyia [file zookeys-428-097-s005.zip › SF5_ZooKeys_key to Carpophthoromyia/key/SF5_ZooKeys_key to Carpophthoromyia/Media/Images/359 wing male dorsal (automontage (c) RMCA).jpg]

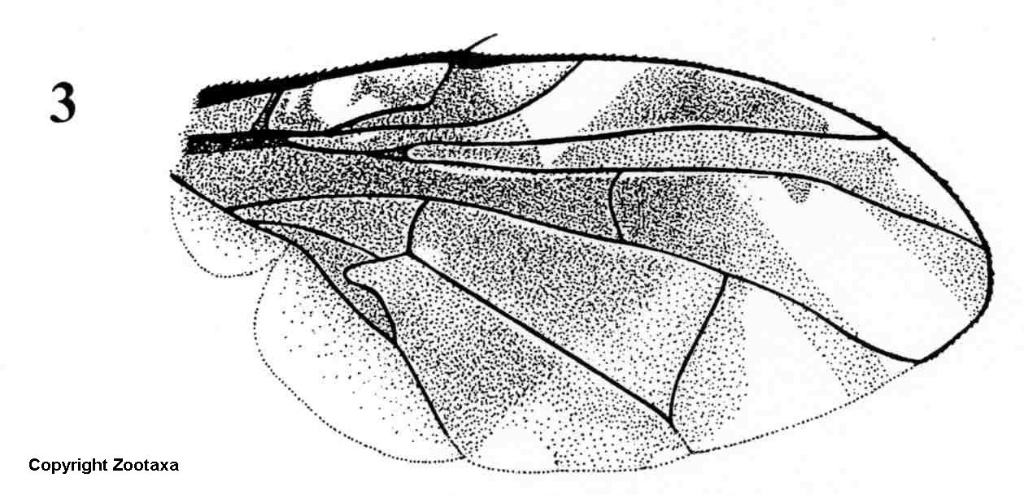

Supplement: Supplementary material 5 — Key to Carpophthoromyia [file zookeys-428-097-s005.zip › SF5_ZooKeys_key to Carpophthoromyia/key/SF5_ZooKeys_key to Carpophthoromyia/Media/Images/359 wing male dorsal (drawing (c) Zootaxa).jpg]

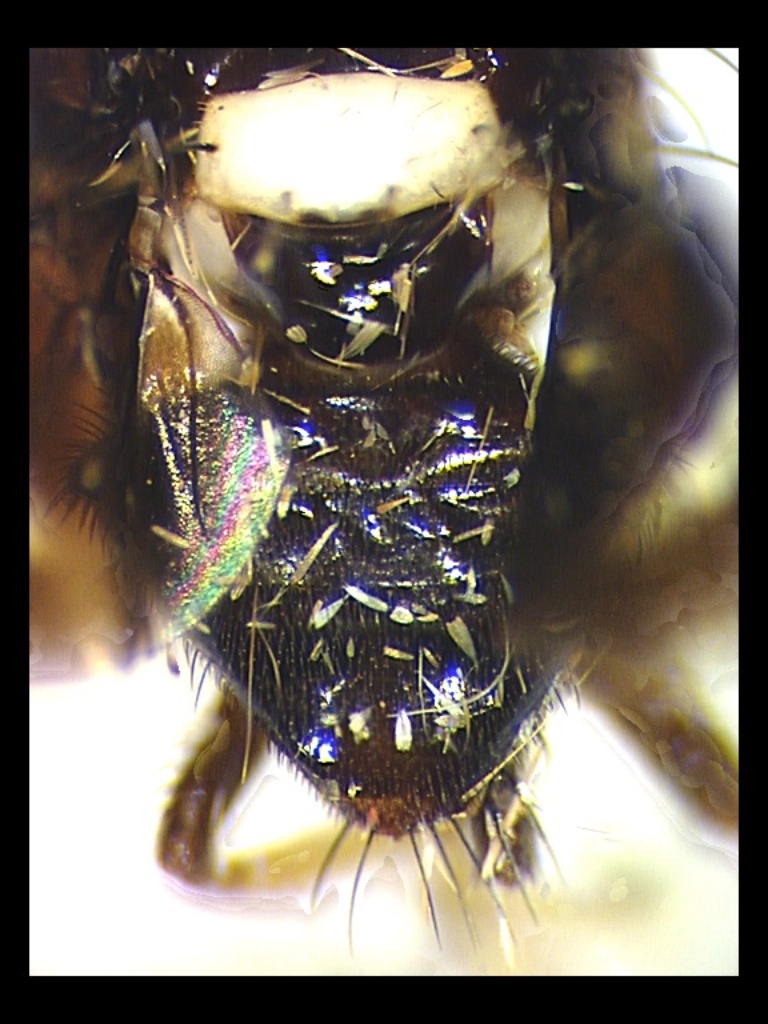

Supplement: Supplementary material 5 — Key to Carpophthoromyia [file zookeys-428-097-s005.zip › SF5_ZooKeys_key to Carpophthoromyia/key/SF5_ZooKeys_key to Carpophthoromyia/Media/Images/360 abdomen dorsal (automontage (c) RMCA).jpg]

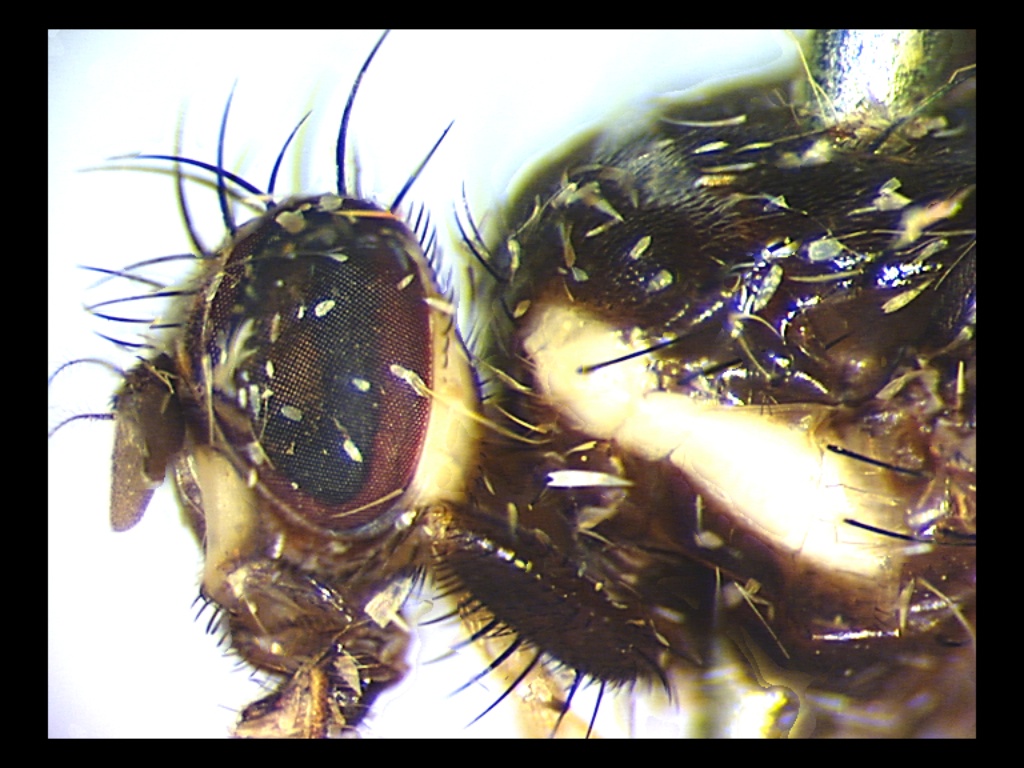

Supplement: Supplementary material 5 — Key to Carpophthoromyia [file zookeys-428-097-s005.zip › SF5_ZooKeys_key to Carpophthoromyia/key/SF5_ZooKeys_key to Carpophthoromyia/Media/Images/360 head and thorax lateral (automontage (c) RMCA) (1).jpg]

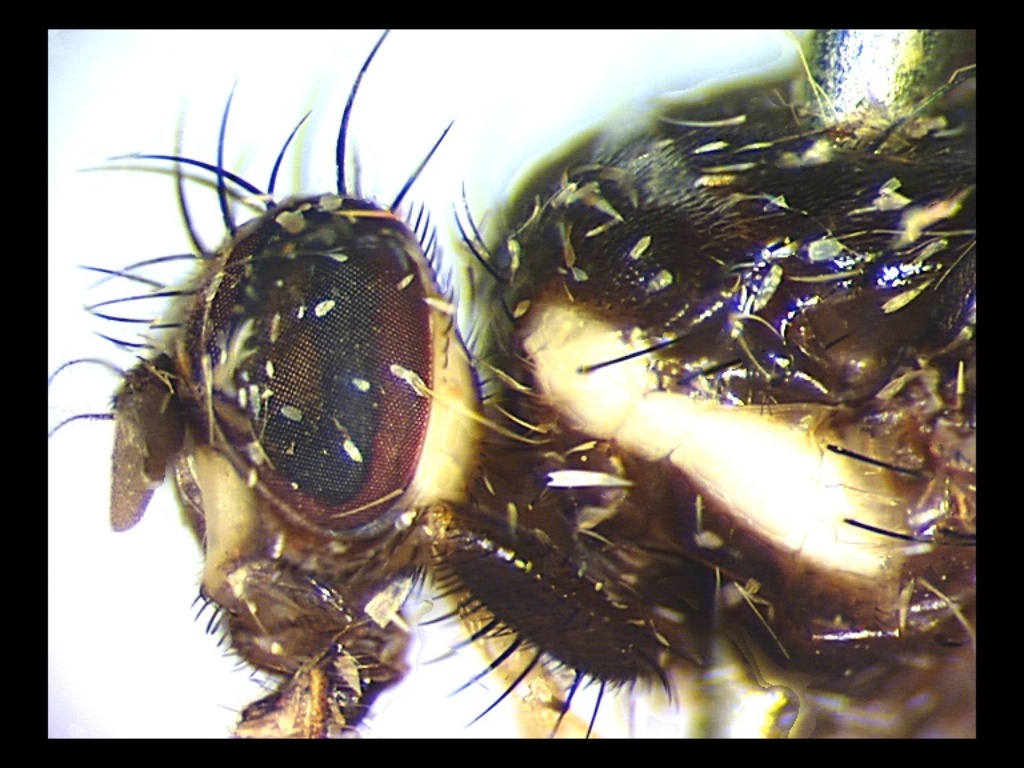

Supplement: Supplementary material 5 — Key to Carpophthoromyia [file zookeys-428-097-s005.zip › SF5_ZooKeys_key to Carpophthoromyia/key/SF5_ZooKeys_key to Carpophthoromyia/Media/Images/360 head and thorax lateral (automontage (c) RMCA).jpg]

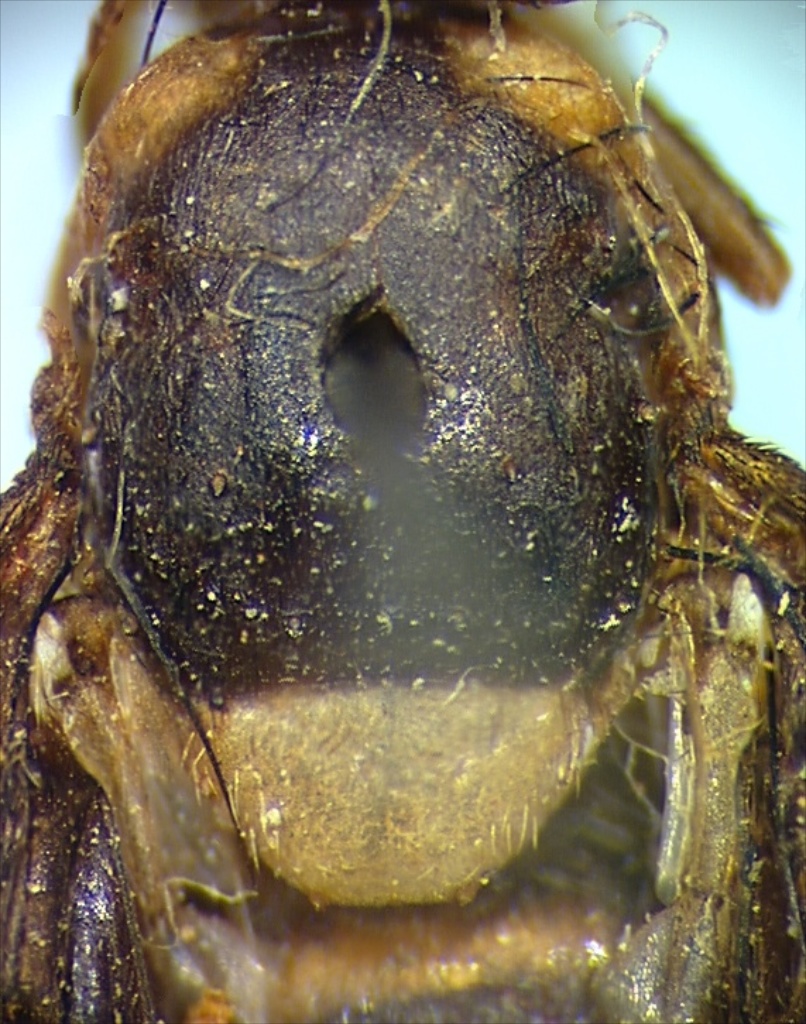

Supplement: Supplementary material 5 — Key to Carpophthoromyia [file zookeys-428-097-s005.zip › SF5_ZooKeys_key to Carpophthoromyia/key/SF5_ZooKeys_key to Carpophthoromyia/Media/Images/360 mesonotum dorsal (automontage (c) RMCA).jpg]

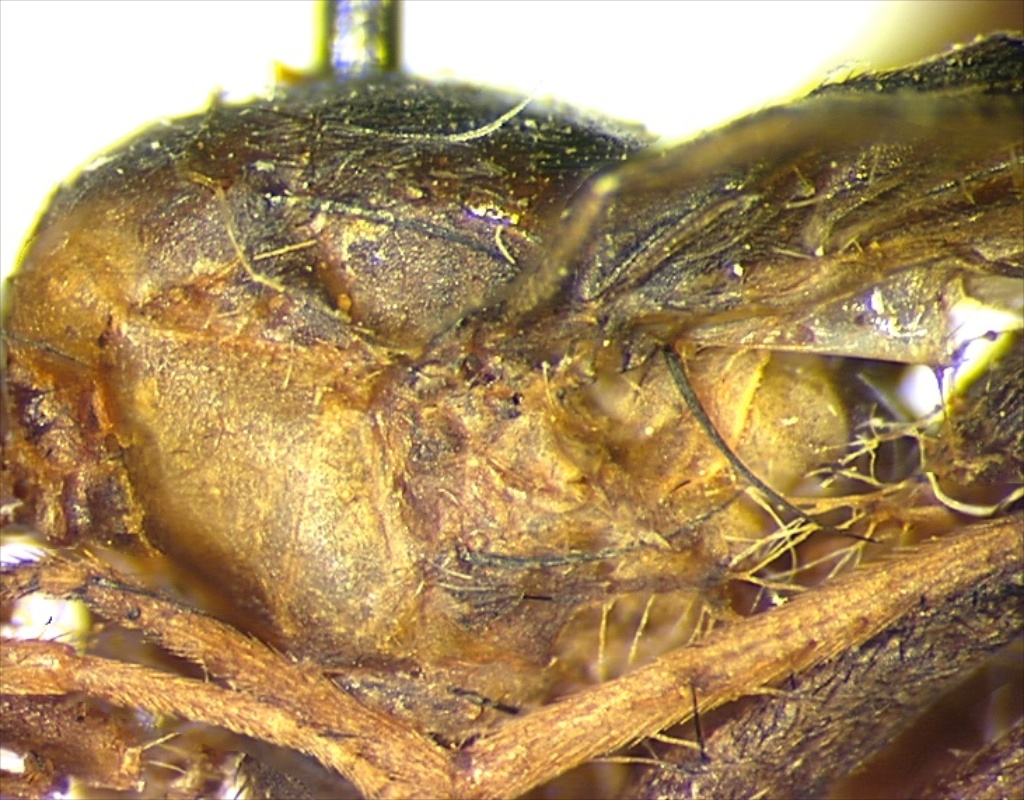

Supplement: Supplementary material 5 — Key to Carpophthoromyia [file zookeys-428-097-s005.zip › SF5_ZooKeys_key to Carpophthoromyia/key/SF5_ZooKeys_key to Carpophthoromyia/Media/Images/360 thorax lateral (automontage (c) RMCA).jpg]

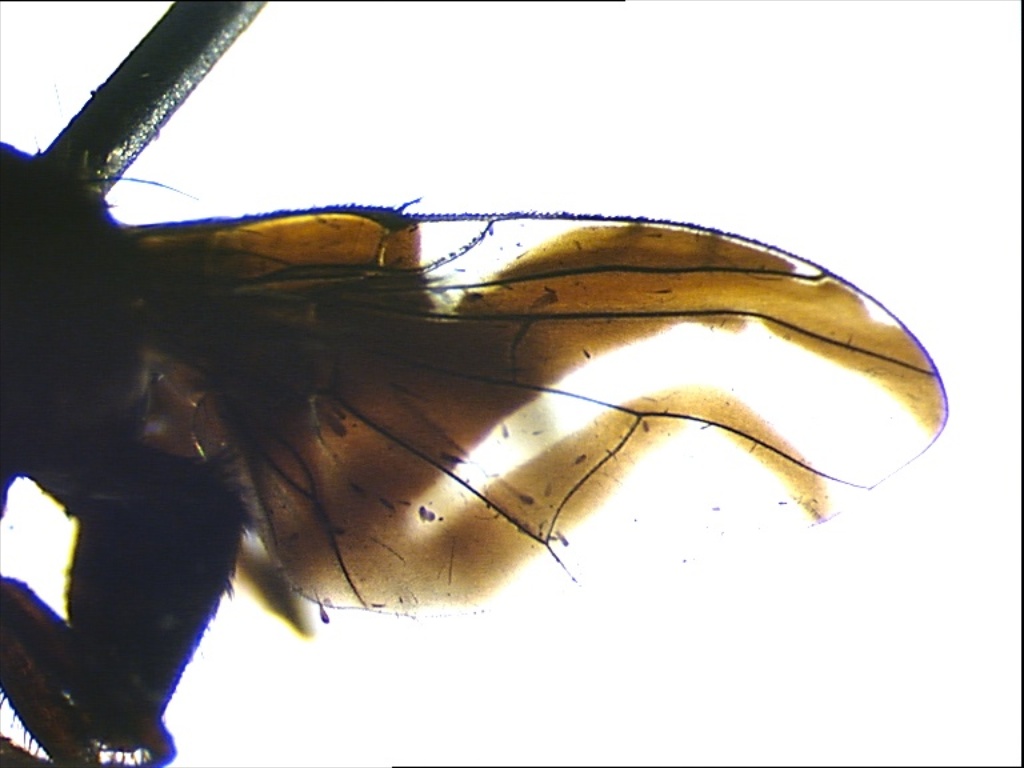

Supplement: Supplementary material 5 — Key to Carpophthoromyia [file zookeys-428-097-s005.zip › SF5_ZooKeys_key to Carpophthoromyia/key/SF5_ZooKeys_key to Carpophthoromyia/Media/Images/360 wing dorsal (automontage (c) RMCA).jpg]

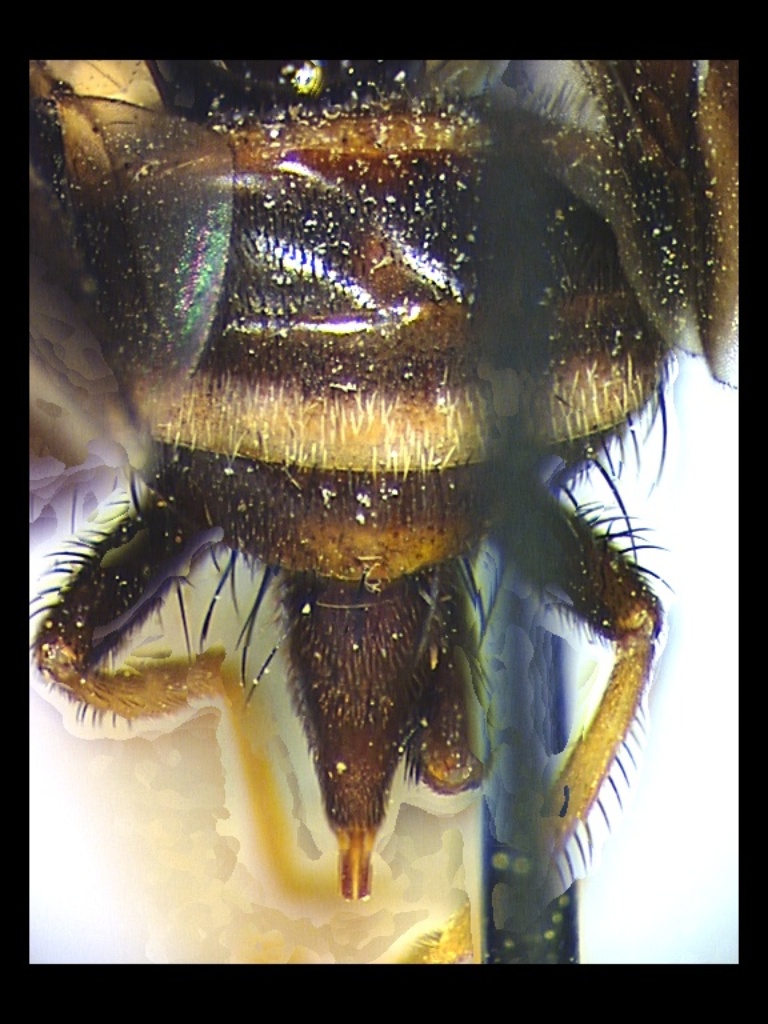

Supplement: Supplementary material 5 — Key to Carpophthoromyia [file zookeys-428-097-s005.zip › SF5_ZooKeys_key to Carpophthoromyia/key/SF5_ZooKeys_key to Carpophthoromyia/Media/Images/361 abdomen female dorsal (automontage (c) RMCA).jpg]

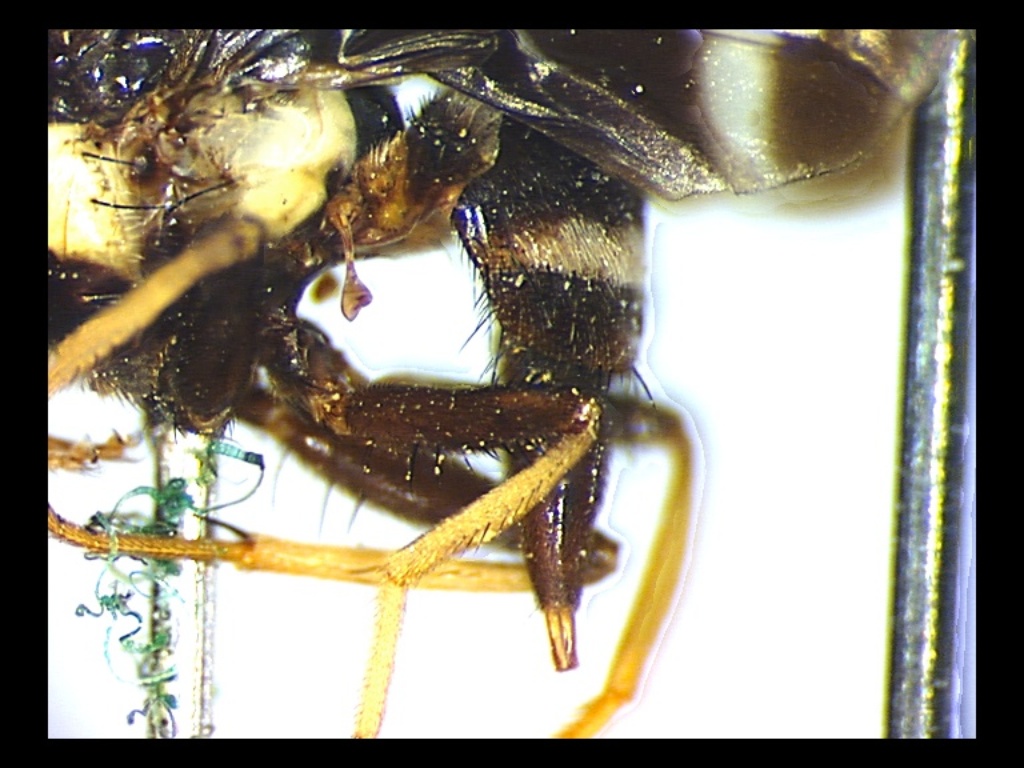

Supplement: Supplementary material 5 — Key to Carpophthoromyia [file zookeys-428-097-s005.zip › SF5_ZooKeys_key to Carpophthoromyia/key/SF5_ZooKeys_key to Carpophthoromyia/Media/Images/361 abdomen female lateral (automontage (c) RMCA).jpg]

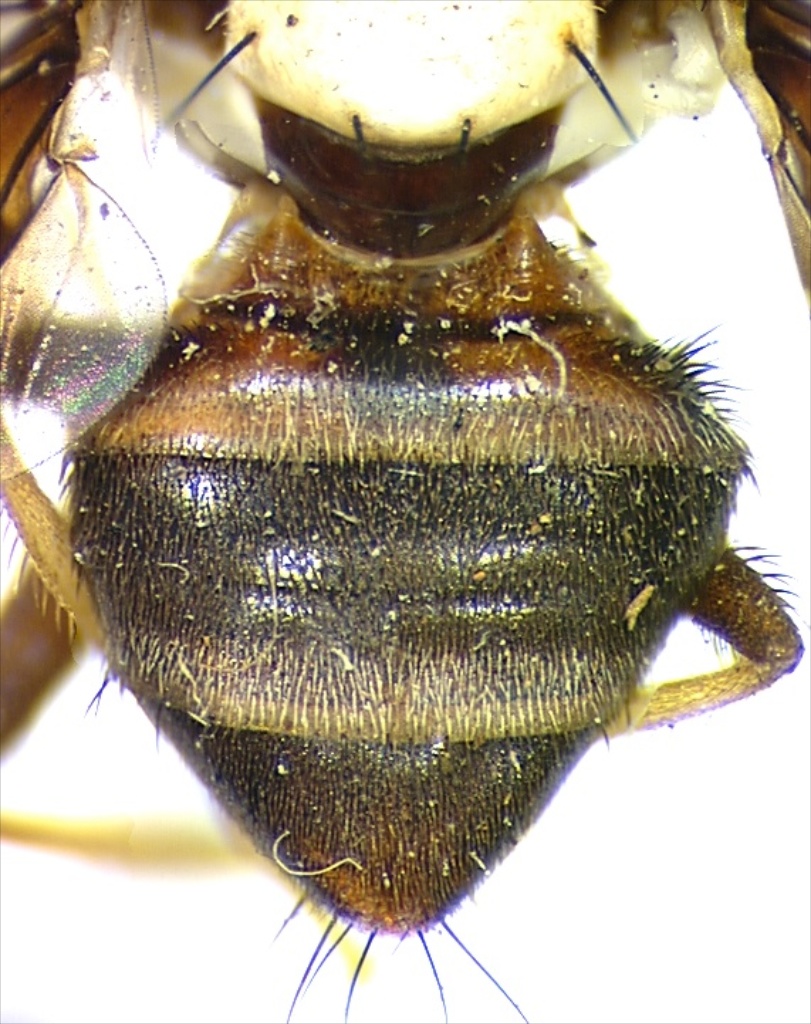

Supplement: Supplementary material 5 — Key to Carpophthoromyia [file zookeys-428-097-s005.zip › SF5_ZooKeys_key to Carpophthoromyia/key/SF5_ZooKeys_key to Carpophthoromyia/Media/Images/361 abdomen male dorsal (automontage (c) RMCA).jpg]

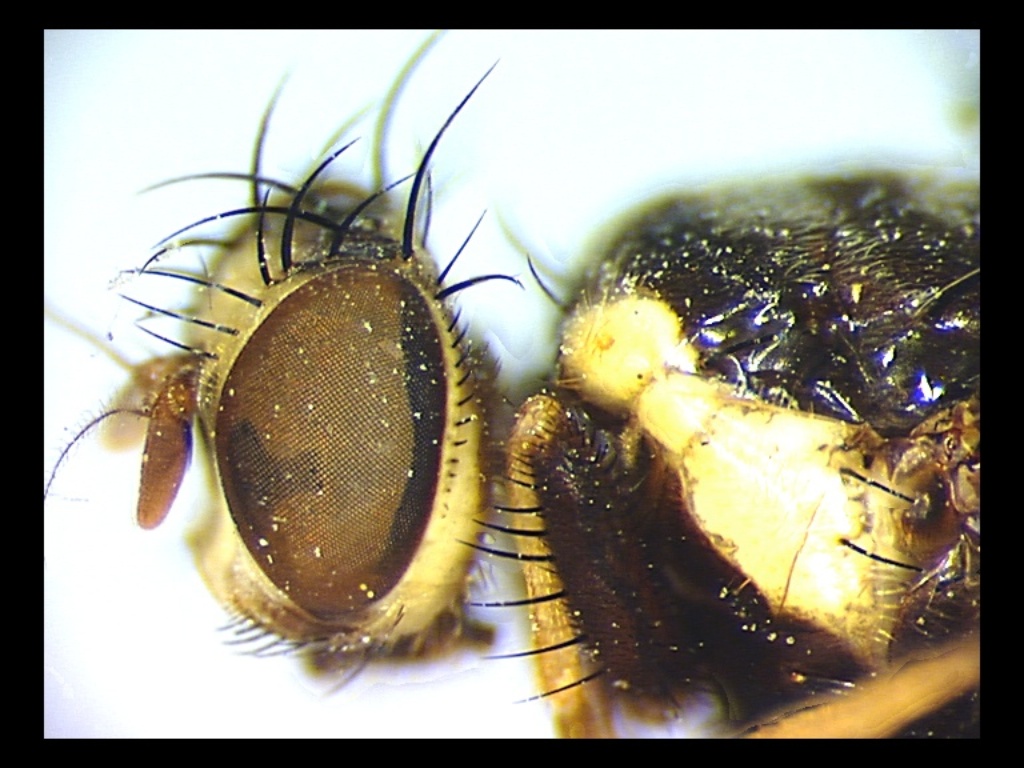

Supplement: Supplementary material 5 — Key to Carpophthoromyia [file zookeys-428-097-s005.zip › SF5_ZooKeys_key to Carpophthoromyia/key/SF5_ZooKeys_key to Carpophthoromyia/Media/Images/361 head and thorax lateral (automontage (c) RMCA).jpg]

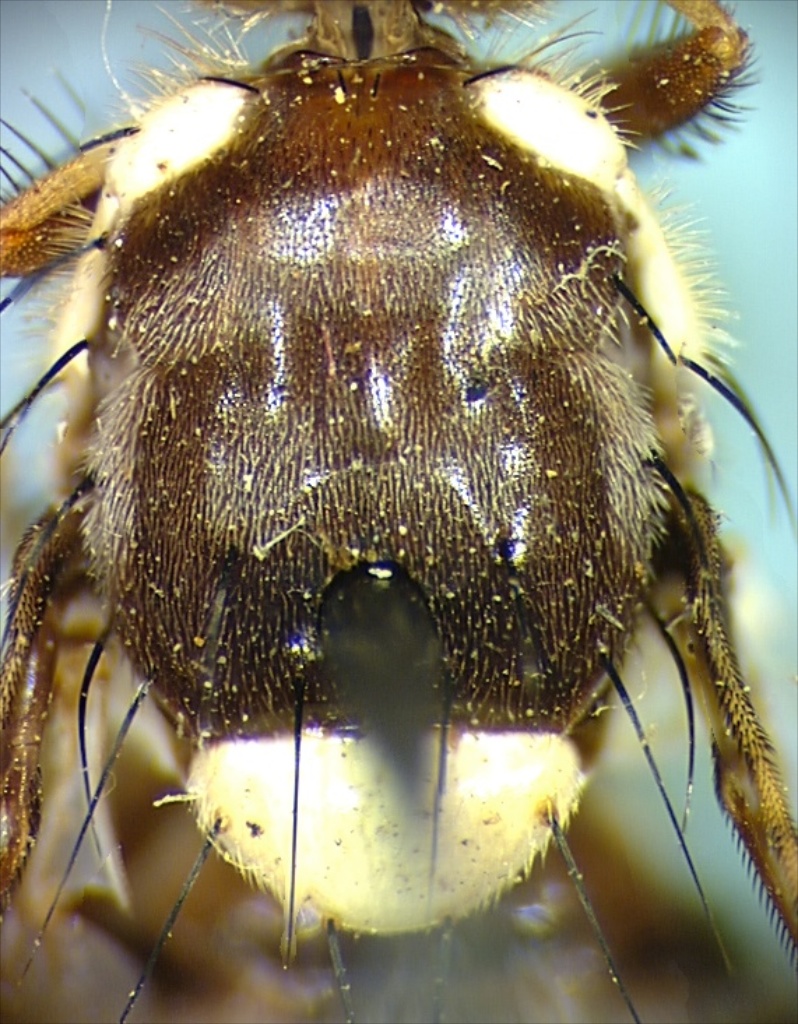

Supplement: Supplementary material 5 — Key to Carpophthoromyia [file zookeys-428-097-s005.zip › SF5_ZooKeys_key to Carpophthoromyia/key/SF5_ZooKeys_key to Carpophthoromyia/Media/Images/361 mesonotum dorsal (automontage (c) RMCA).jpg]

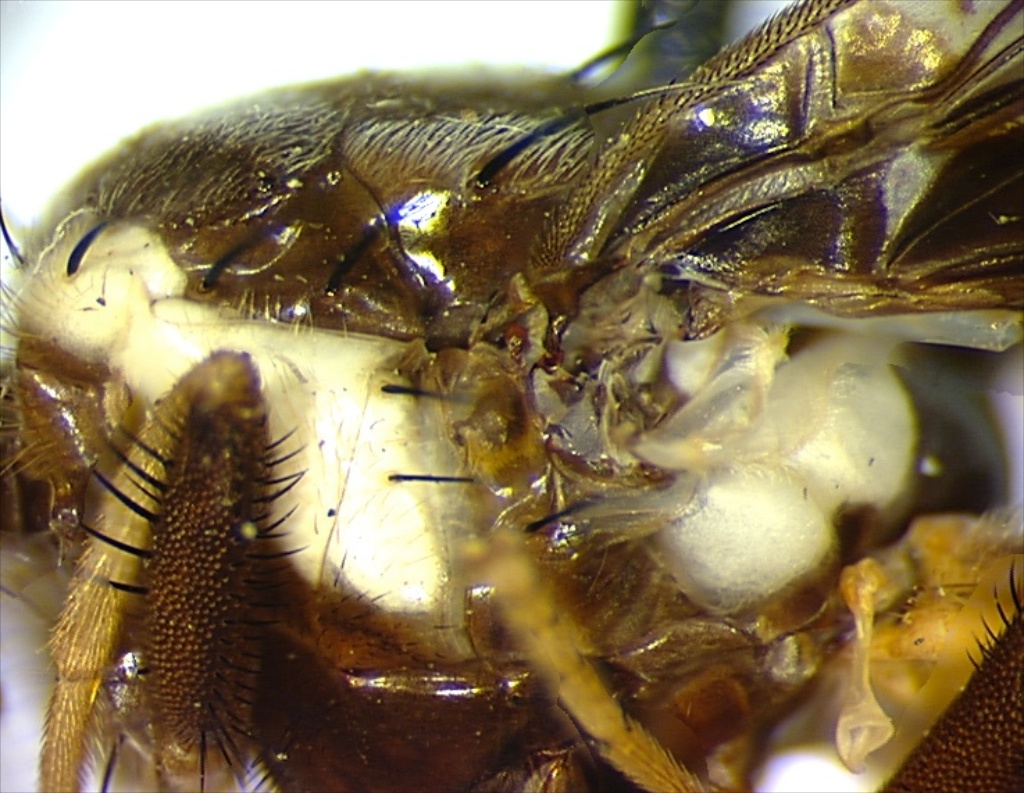

Supplement: Supplementary material 5 — Key to Carpophthoromyia [file zookeys-428-097-s005.zip › SF5_ZooKeys_key to Carpophthoromyia/key/SF5_ZooKeys_key to Carpophthoromyia/Media/Images/361 thorax lateral (automontage (c) RMCA).jpg]

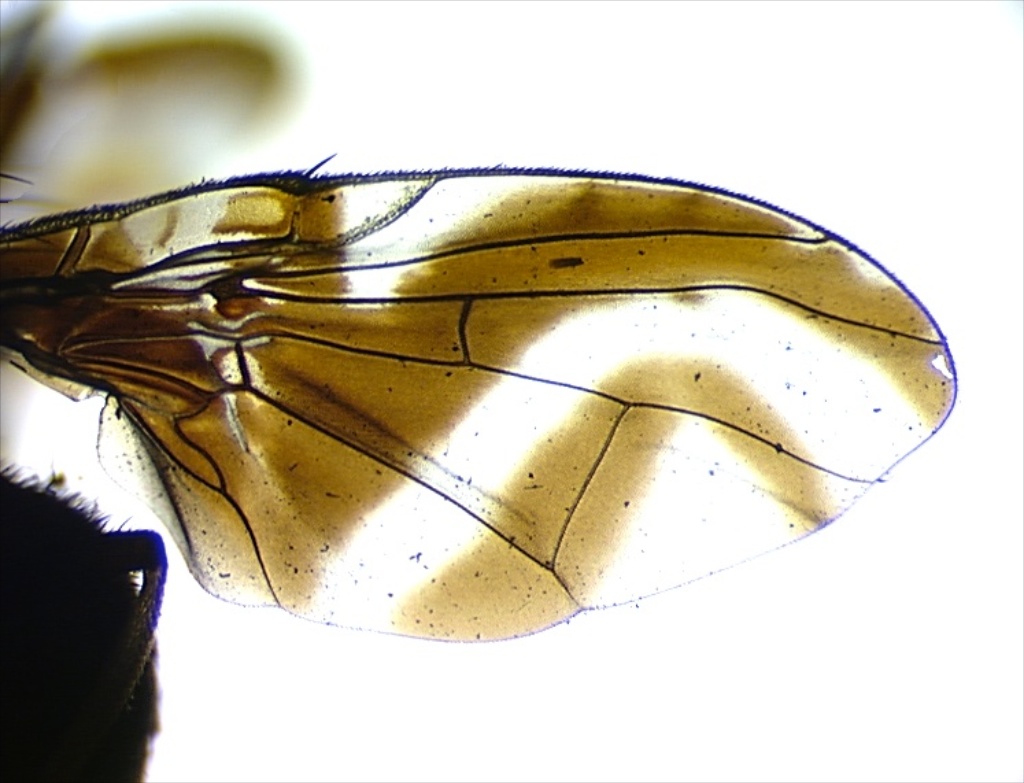

Supplement: Supplementary material 5 — Key to Carpophthoromyia [file zookeys-428-097-s005.zip › SF5_ZooKeys_key to Carpophthoromyia/key/SF5_ZooKeys_key to Carpophthoromyia/Media/Images/361 wing dorsal (automontage (c) RMCA).jpg]

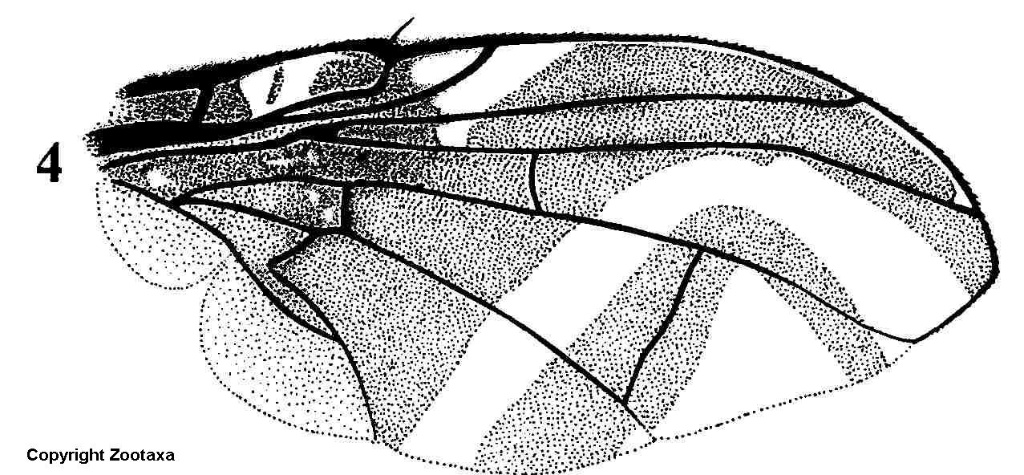

Supplement: Supplementary material 5 — Key to Carpophthoromyia [file zookeys-428-097-s005.zip › SF5_ZooKeys_key to Carpophthoromyia/key/SF5_ZooKeys_key to Carpophthoromyia/Media/Images/361 wing dorsal (drawing (c) Zootaxa).jpg]

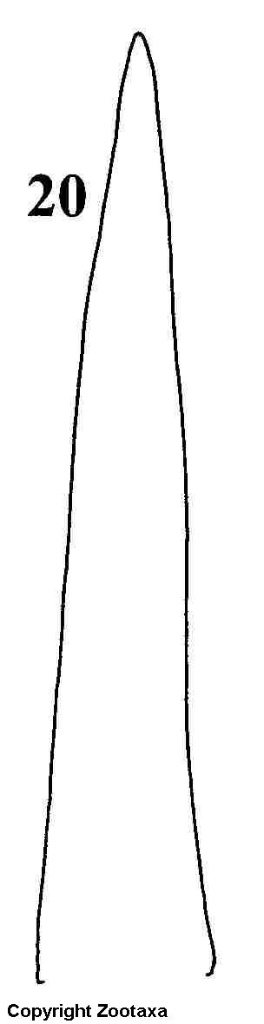

Supplement: Supplementary material 5 — Key to Carpophthoromyia [file zookeys-428-097-s005.zip › SF5_ZooKeys_key to Carpophthoromyia/key/SF5_ZooKeys_key to Carpophthoromyia/Media/Images/362 aculeus dorsal (drawing (c) Zootaxa).jpg]

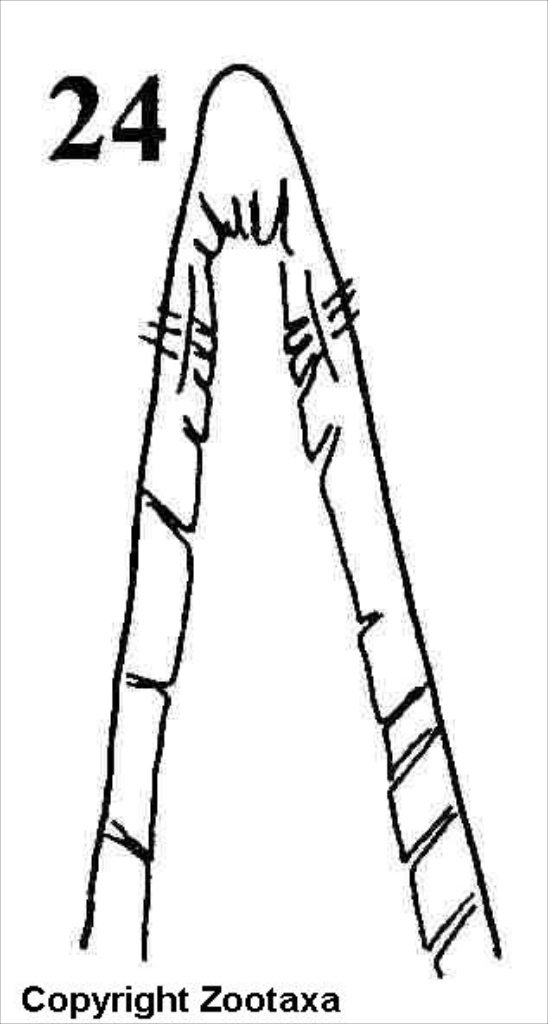

Supplement: Supplementary material 5 — Key to Carpophthoromyia [file zookeys-428-097-s005.zip › SF5_ZooKeys_key to Carpophthoromyia/key/SF5_ZooKeys_key to Carpophthoromyia/Media/Images/362 aculeus tip dorsal (drawing (c) Zootaxa).jpg]

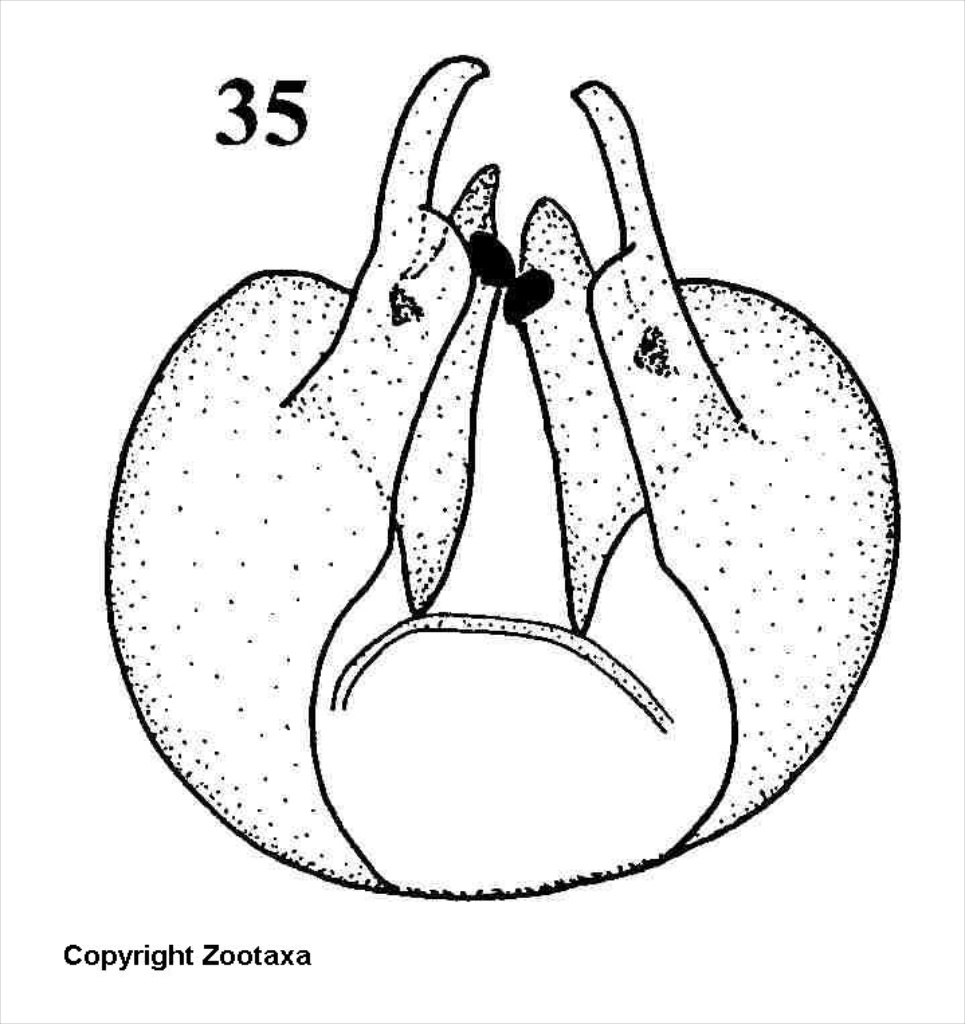

Supplement: Supplementary material 5 — Key to Carpophthoromyia [file zookeys-428-097-s005.zip › SF5_ZooKeys_key to Carpophthoromyia/key/SF5_ZooKeys_key to Carpophthoromyia/Media/Images/362 epandrium posterior (drawing (c) Zootaxa).jpg]

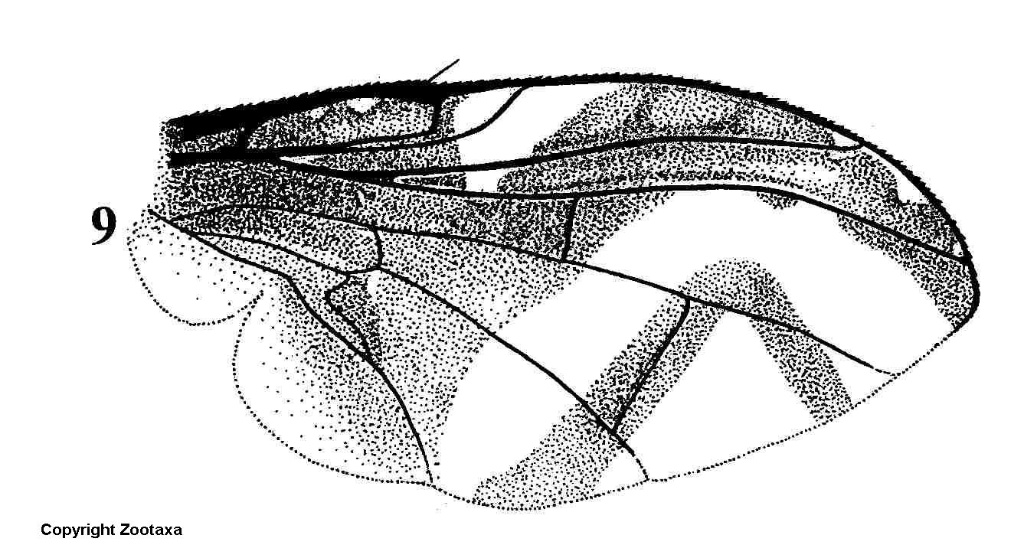

Supplement: Supplementary material 5 — Key to Carpophthoromyia [file zookeys-428-097-s005.zip › SF5_ZooKeys_key to Carpophthoromyia/key/SF5_ZooKeys_key to Carpophthoromyia/Media/Images/362 wing dorsal (drawing (c) Zootaxa).jpg]

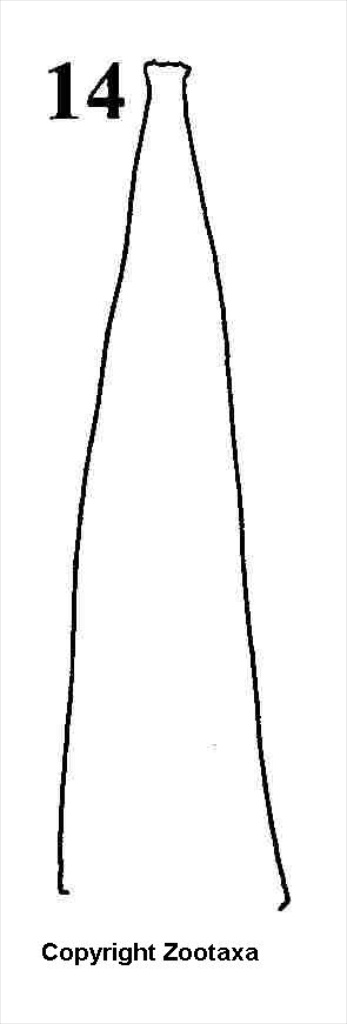

Supplement: Supplementary material 5 — Key to Carpophthoromyia [file zookeys-428-097-s005.zip › SF5_ZooKeys_key to Carpophthoromyia/key/SF5_ZooKeys_key to Carpophthoromyia/Media/Images/363 aculeus dorsal (drawing (c) Zootaxa).jpg]

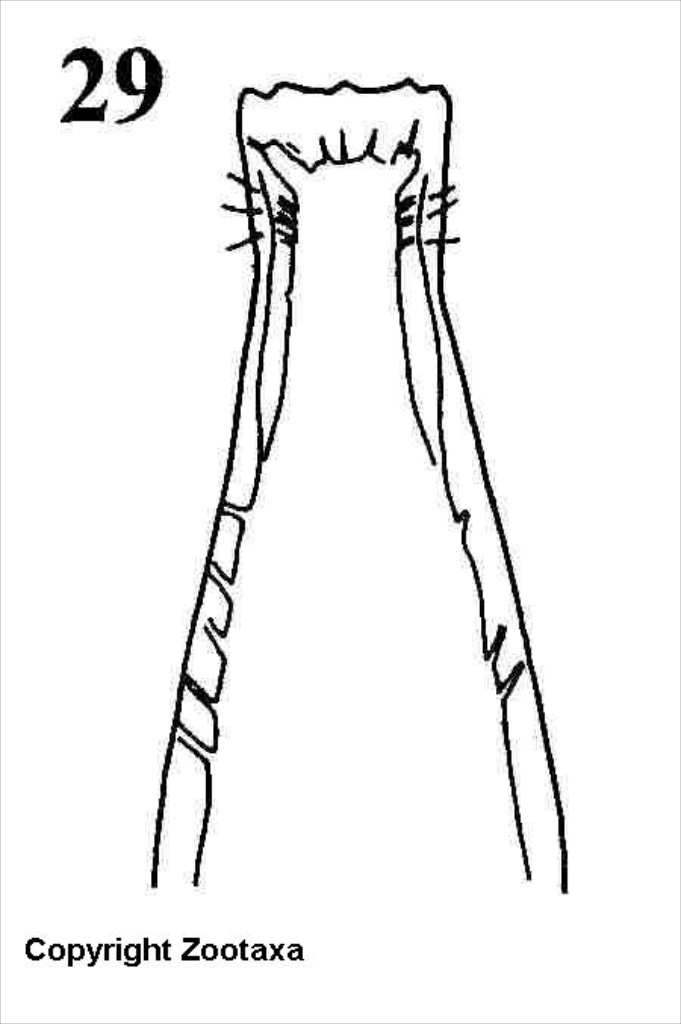

Supplement: Supplementary material 5 — Key to Carpophthoromyia [file zookeys-428-097-s005.zip › SF5_ZooKeys_key to Carpophthoromyia/key/SF5_ZooKeys_key to Carpophthoromyia/Media/Images/363 aculeus tip dorsal (drawing (c) Zootaxa).jpg]

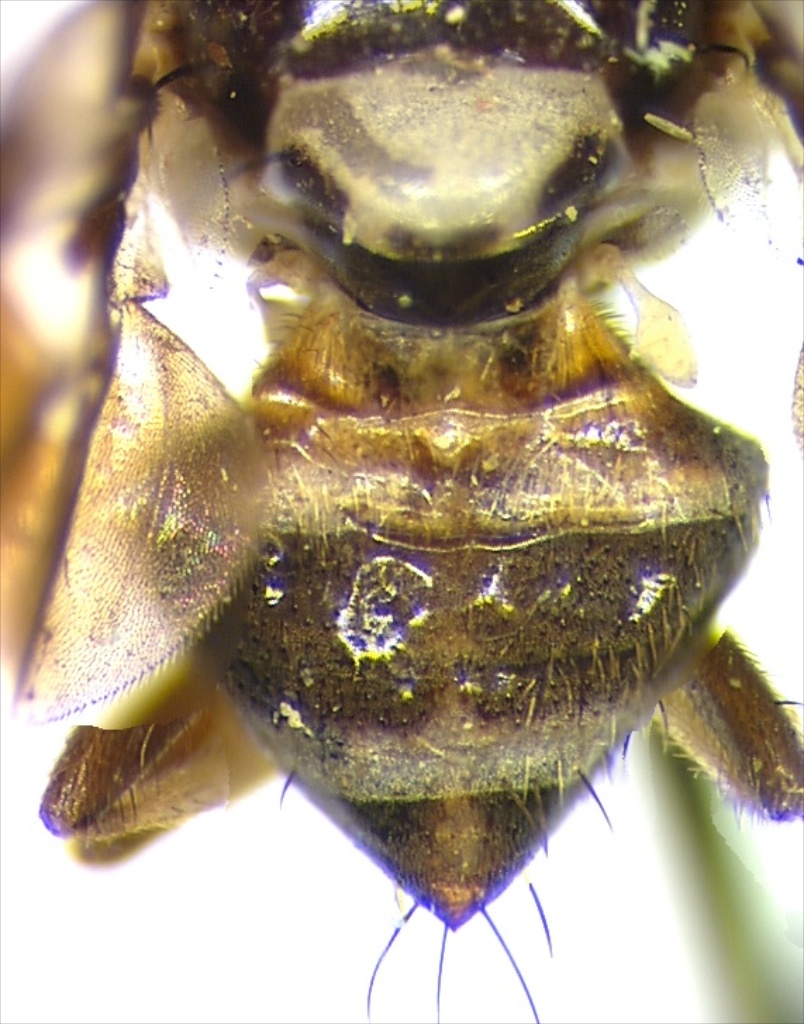

Supplement: Supplementary material 5 — Key to Carpophthoromyia [file zookeys-428-097-s005.zip › SF5_ZooKeys_key to Carpophthoromyia/key/SF5_ZooKeys_key to Carpophthoromyia/Media/Images/364 abdomen dorsal (automontage (c) RMCA).jpg]

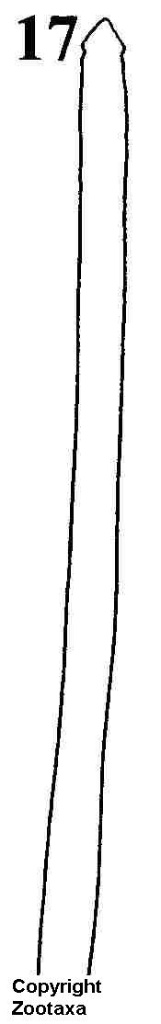

Supplement: Supplementary material 5 — Key to Carpophthoromyia [file zookeys-428-097-s005.zip › SF5_ZooKeys_key to Carpophthoromyia/key/SF5_ZooKeys_key to Carpophthoromyia/Media/Images/364 aculeus dorsal (drawing (c) Zootaxa).jpg]

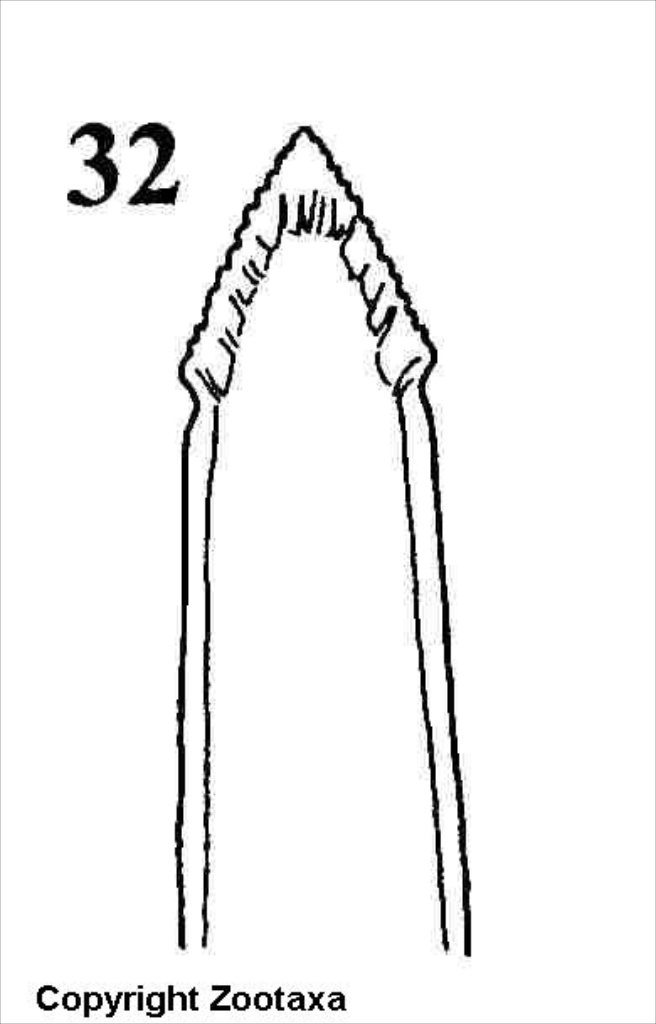

Supplement: Supplementary material 5 — Key to Carpophthoromyia [file zookeys-428-097-s005.zip › SF5_ZooKeys_key to Carpophthoromyia/key/SF5_ZooKeys_key to Carpophthoromyia/Media/Images/364 aculeus tip dorsal (drawing (c) Zootaxa).jpg]

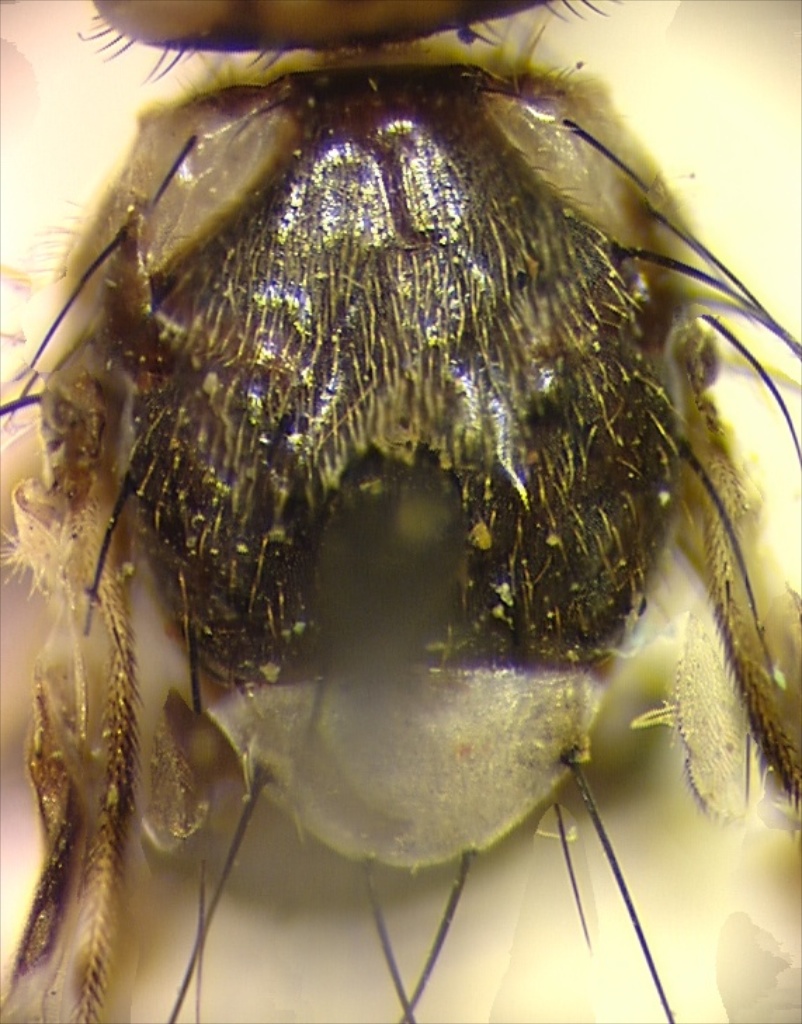

Supplement: Supplementary material 5 — Key to Carpophthoromyia [file zookeys-428-097-s005.zip › SF5_ZooKeys_key to Carpophthoromyia/key/SF5_ZooKeys_key to Carpophthoromyia/Media/Images/364 mesonotum dorsal (automontage (c) RMCA).jpg]

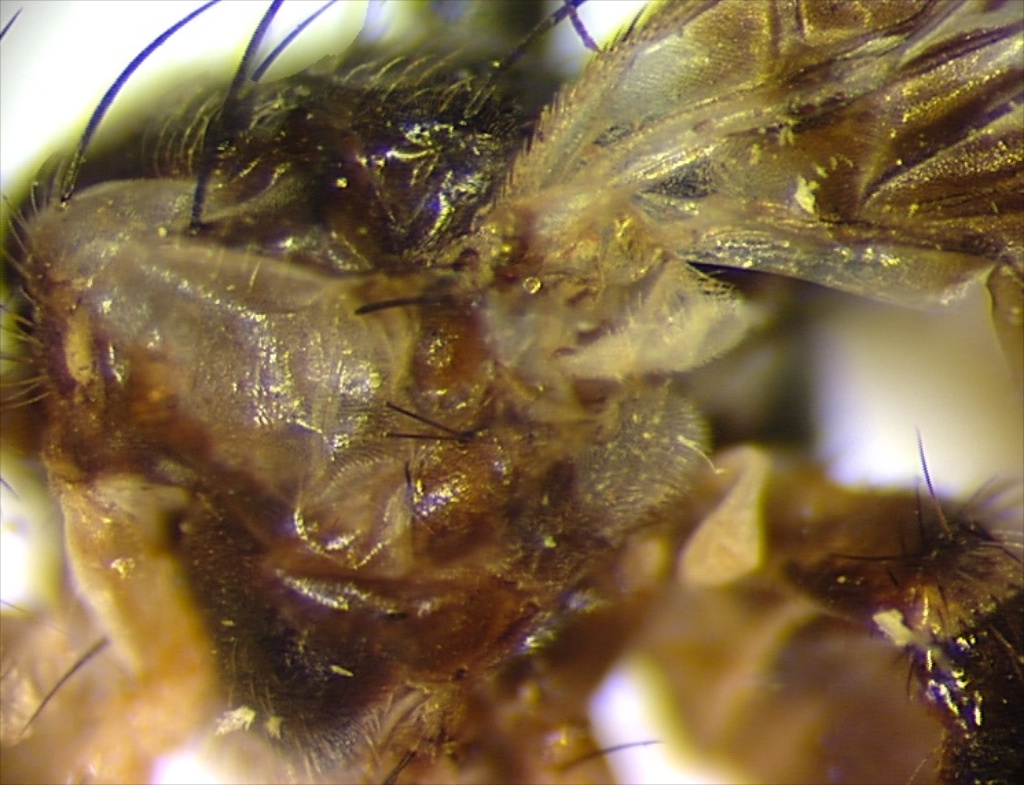

Supplement: Supplementary material 5 — Key to Carpophthoromyia [file zookeys-428-097-s005.zip › SF5_ZooKeys_key to Carpophthoromyia/key/SF5_ZooKeys_key to Carpophthoromyia/Media/Images/364 thorax lateral (automontage (c) RMCA).jpg]

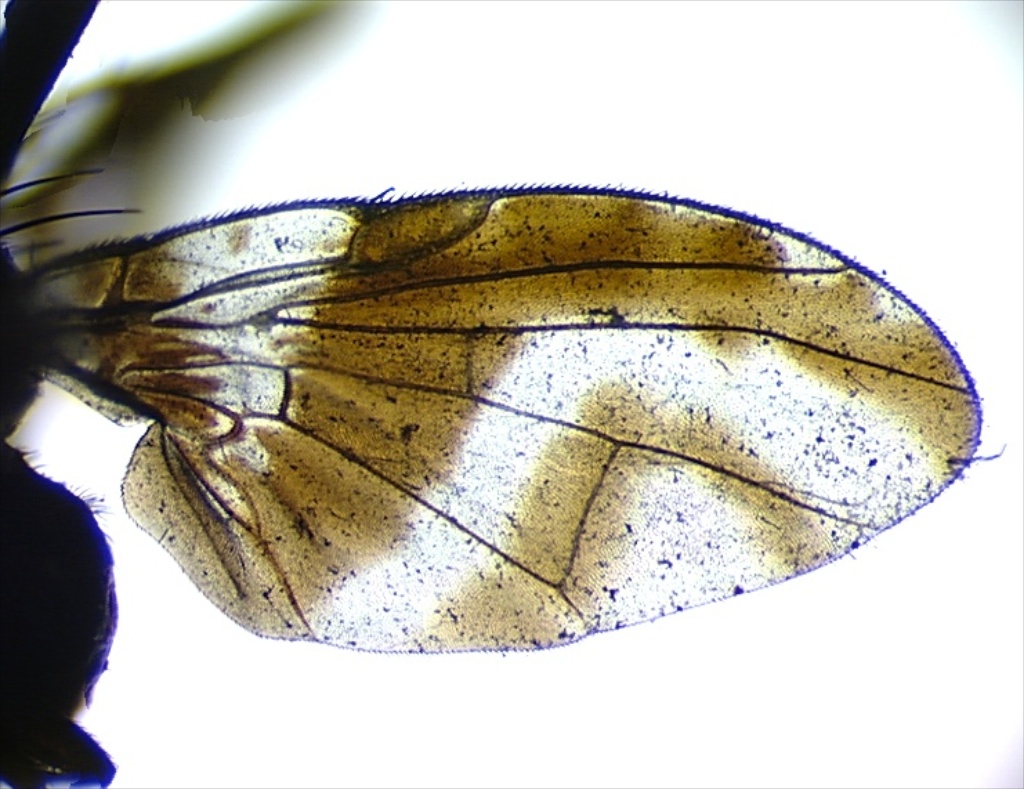

Supplement: Supplementary material 5 — Key to Carpophthoromyia [file zookeys-428-097-s005.zip › SF5_ZooKeys_key to Carpophthoromyia/key/SF5_ZooKeys_key to Carpophthoromyia/Media/Images/364 wing dorsal (automontage (c) RMCA).jpg]

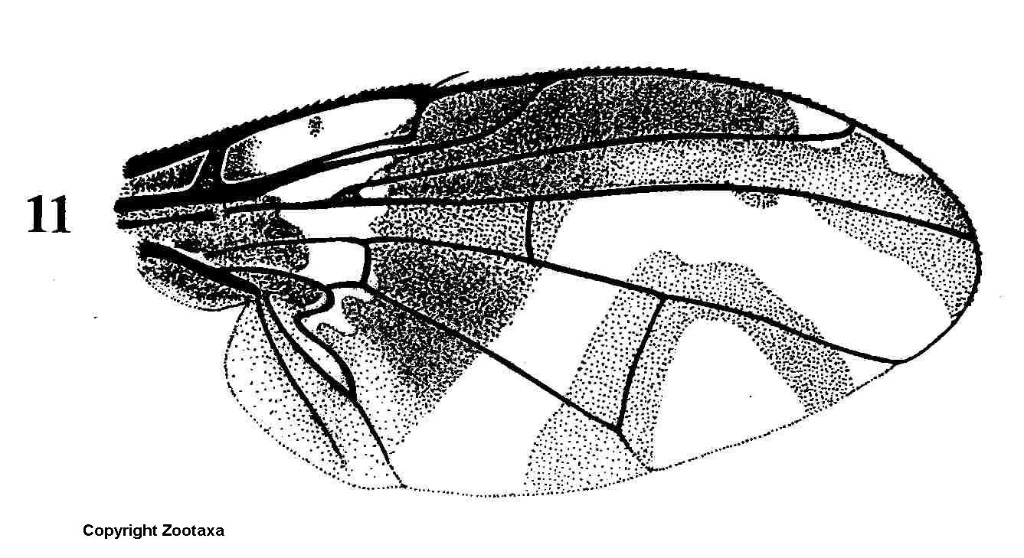

Supplement: Supplementary material 5 — Key to Carpophthoromyia [file zookeys-428-097-s005.zip › SF5_ZooKeys_key to Carpophthoromyia/key/SF5_ZooKeys_key to Carpophthoromyia/Media/Images/364 wing dorsal (drawing (c) Zootaxa).jpg]

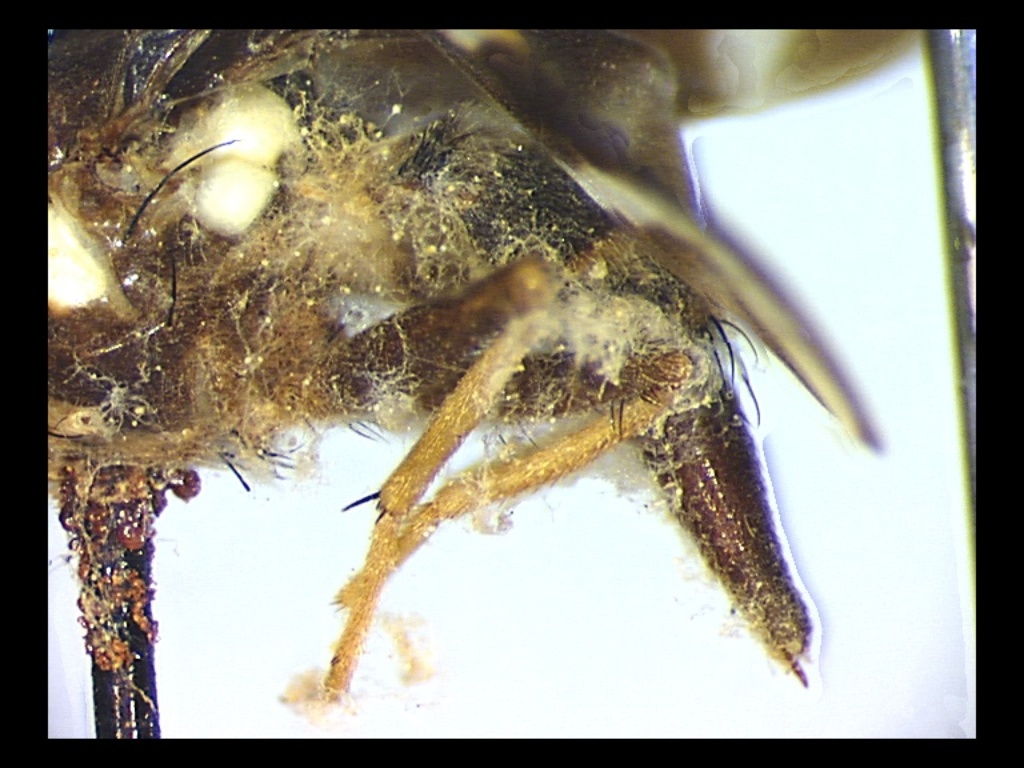

Supplement: Supplementary material 5 — Key to Carpophthoromyia [file zookeys-428-097-s005.zip › SF5_ZooKeys_key to Carpophthoromyia/key/SF5_ZooKeys_key to Carpophthoromyia/Media/Images/365 abdomen lateral (automontage (c) RMCA).jpg]

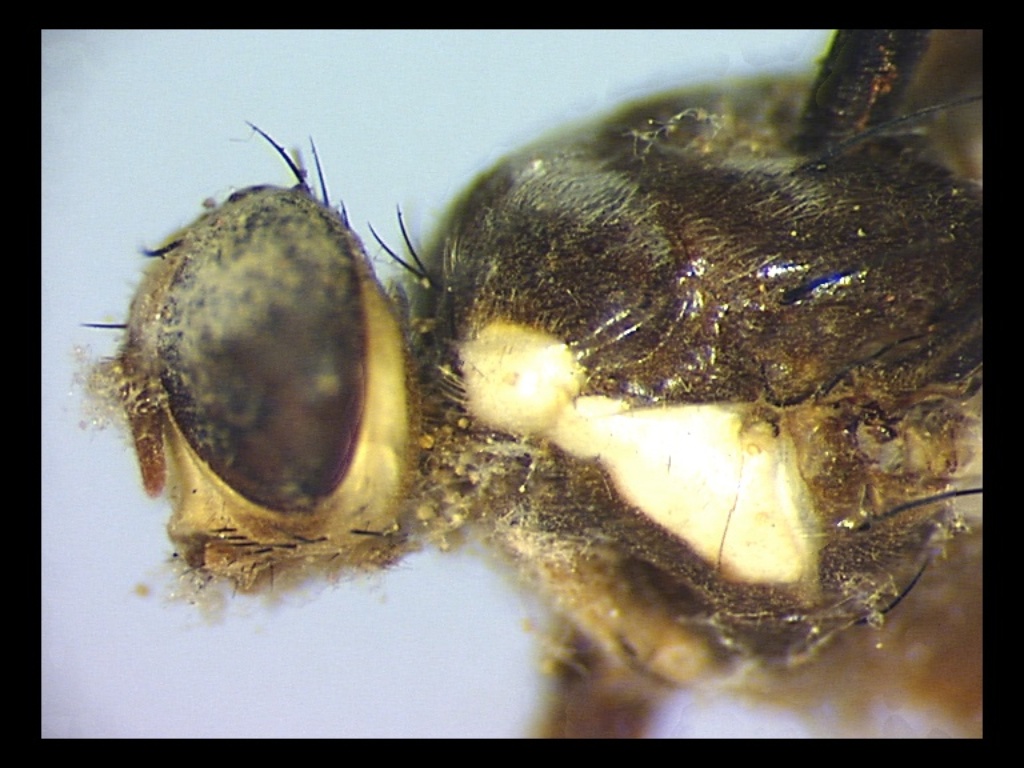

Supplement: Supplementary material 5 — Key to Carpophthoromyia [file zookeys-428-097-s005.zip › SF5_ZooKeys_key to Carpophthoromyia/key/SF5_ZooKeys_key to Carpophthoromyia/Media/Images/365 head and thorax lateral (automontage (c) RMCA).jpg]

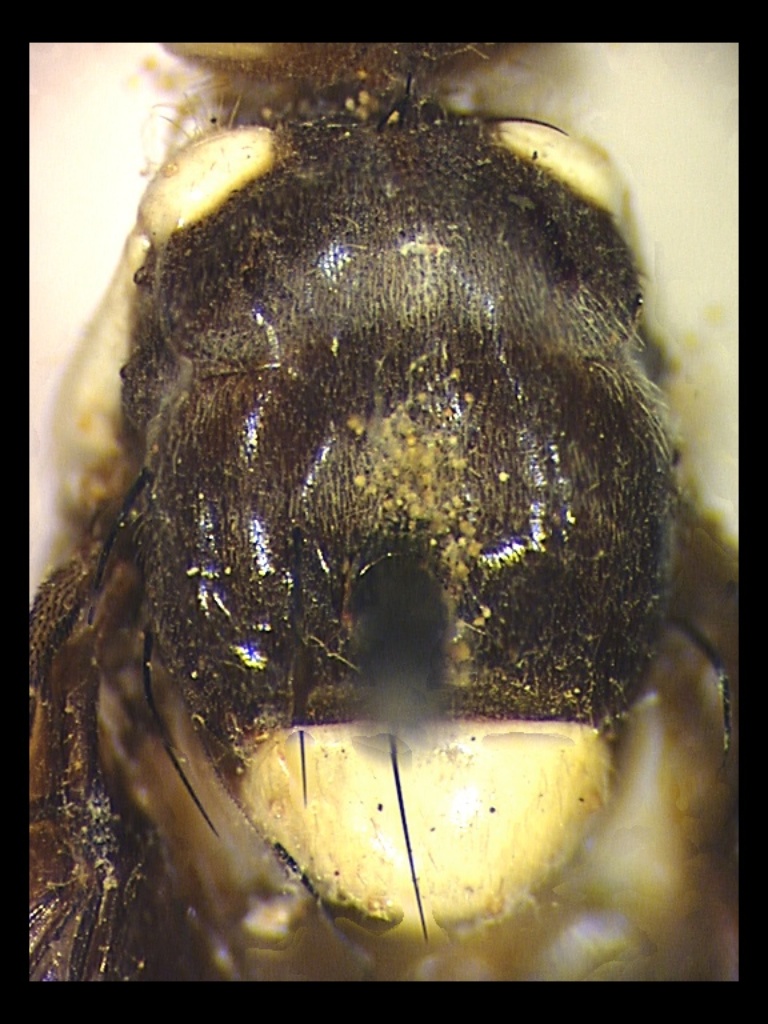

Supplement: Supplementary material 5 — Key to Carpophthoromyia [file zookeys-428-097-s005.zip › SF5_ZooKeys_key to Carpophthoromyia/key/SF5_ZooKeys_key to Carpophthoromyia/Media/Images/365 mesonotum dorsal (automontage (c) RMCA).jpg]

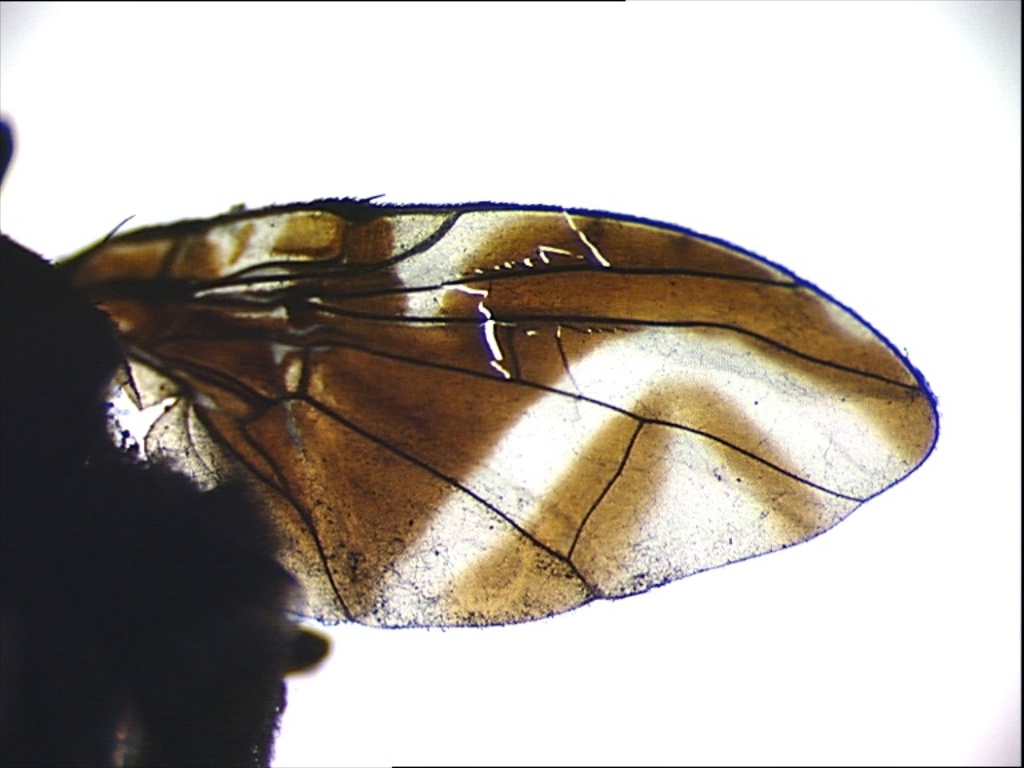

Supplement: Supplementary material 5 — Key to Carpophthoromyia [file zookeys-428-097-s005.zip › SF5_ZooKeys_key to Carpophthoromyia/key/SF5_ZooKeys_key to Carpophthoromyia/Media/Images/365 wing dorsal (automontage (c) RMCA).jpg]

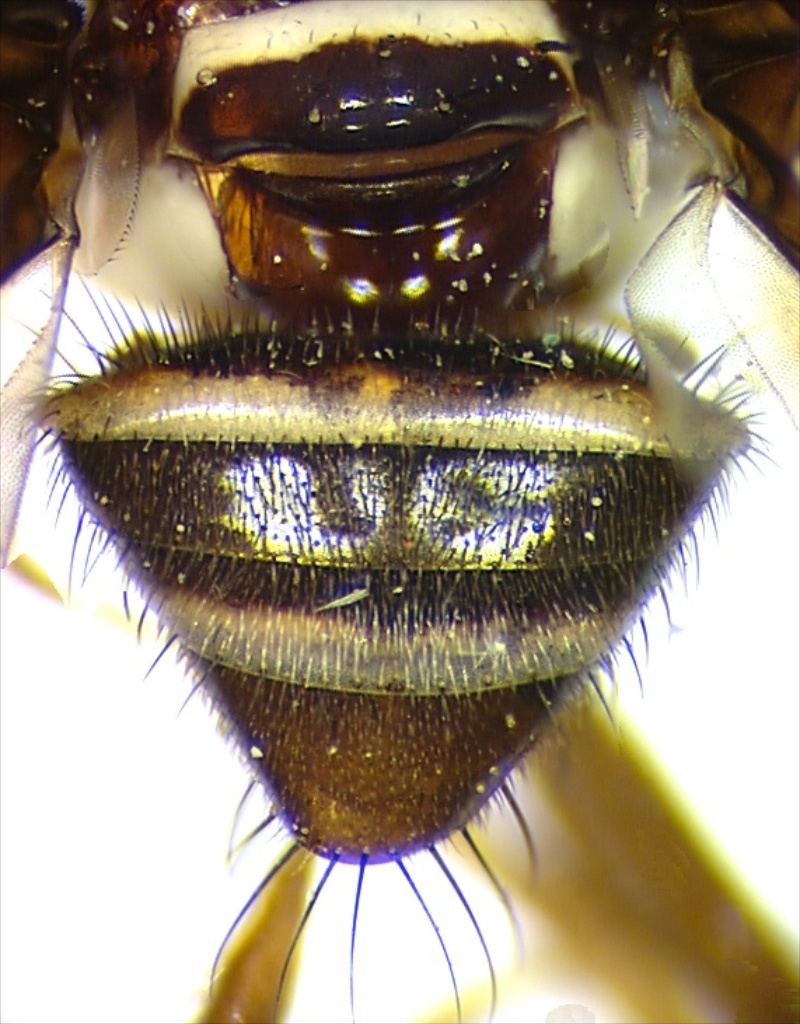

Supplement: Supplementary material 5 — Key to Carpophthoromyia [file zookeys-428-097-s005.zip › SF5_ZooKeys_key to Carpophthoromyia/key/SF5_ZooKeys_key to Carpophthoromyia/Media/Images/366 abdomen dorsal (automontage (c) RMCA).jpg]

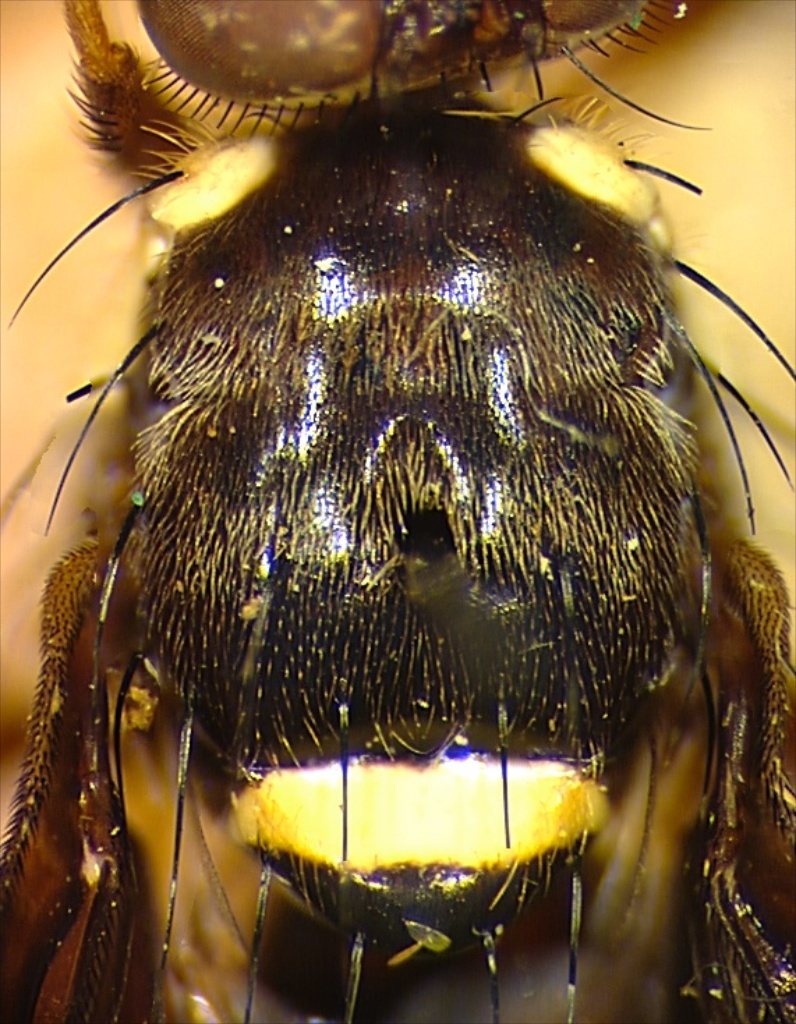

Supplement: Supplementary material 5 — Key to Carpophthoromyia [file zookeys-428-097-s005.zip › SF5_ZooKeys_key to Carpophthoromyia/key/SF5_ZooKeys_key to Carpophthoromyia/Media/Images/366 mesonotum dorsal (automontage (c) RMCA).jpg]

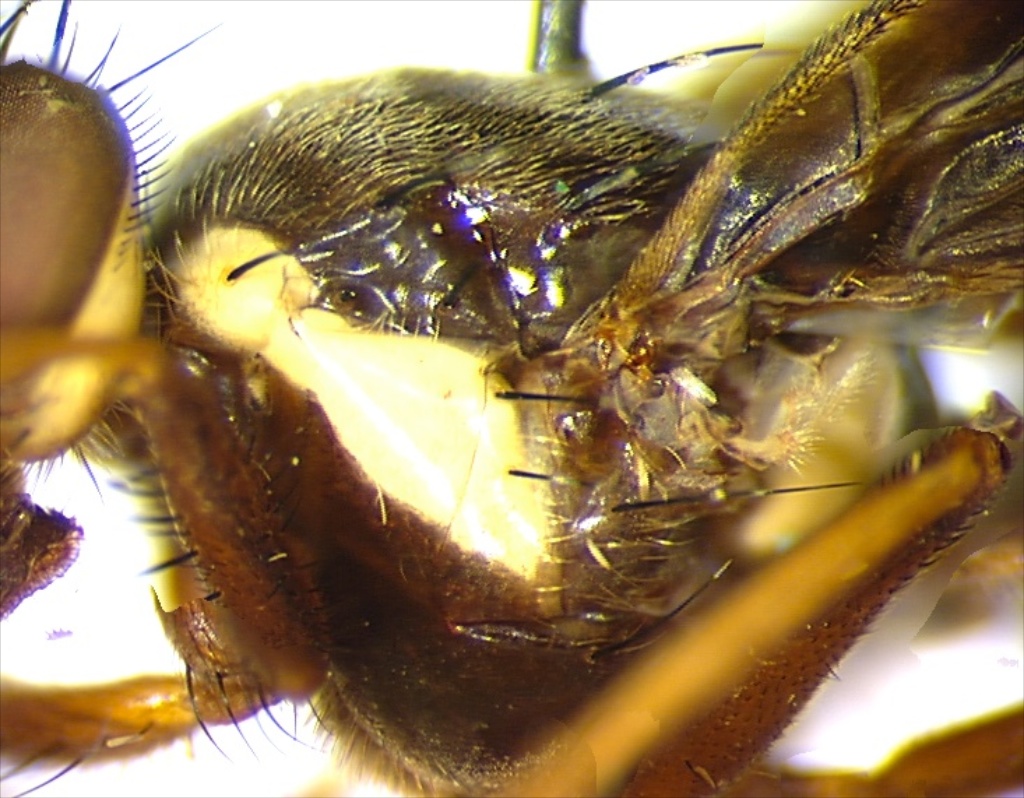

Supplement: Supplementary material 5 — Key to Carpophthoromyia [file zookeys-428-097-s005.zip › SF5_ZooKeys_key to Carpophthoromyia/key/SF5_ZooKeys_key to Carpophthoromyia/Media/Images/366 thorax lateral (automontage (c) RMCA).jpg]

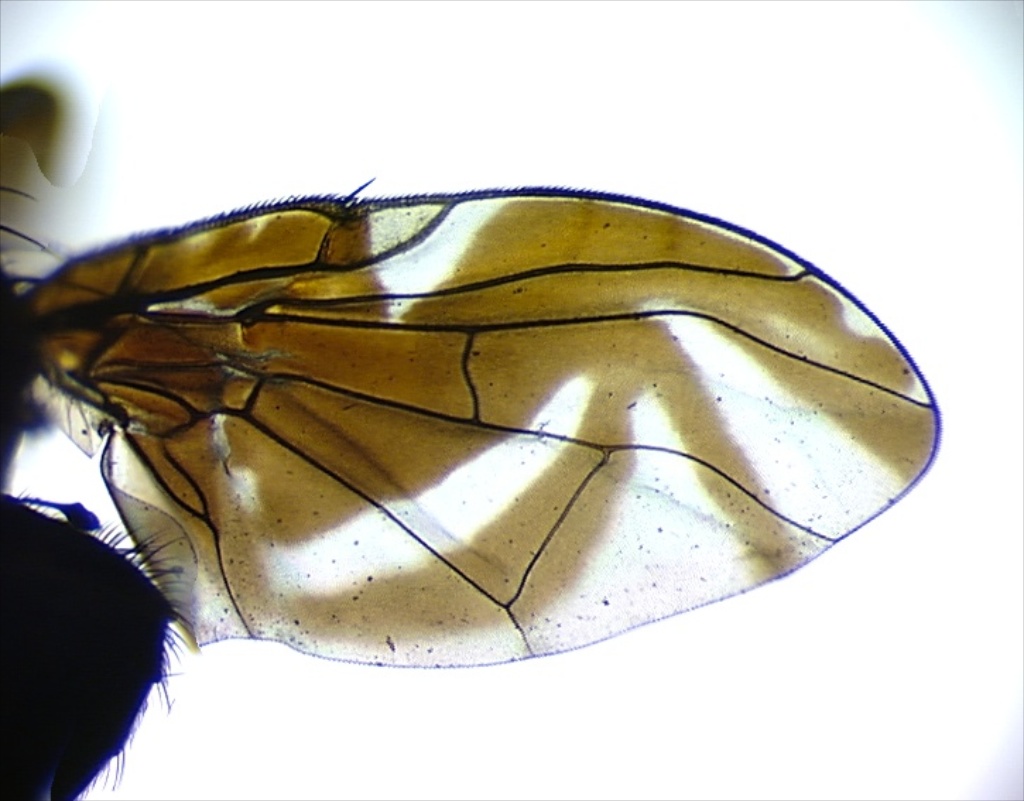

Supplement: Supplementary material 5 — Key to Carpophthoromyia [file zookeys-428-097-s005.zip › SF5_ZooKeys_key to Carpophthoromyia/key/SF5_ZooKeys_key to Carpophthoromyia/Media/Images/366 wing dorsal (automontage (c) RMCA).jpg]

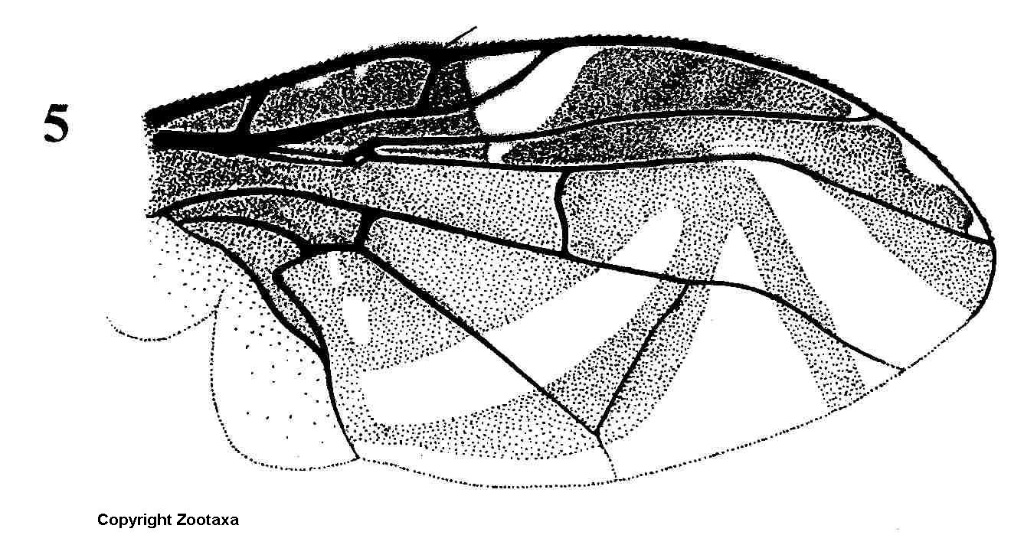

Supplement: Supplementary material 5 — Key to Carpophthoromyia [file zookeys-428-097-s005.zip › SF5_ZooKeys_key to Carpophthoromyia/key/SF5_ZooKeys_key to Carpophthoromyia/Media/Images/366 wing dorsal (drawing (c) Zootaxa).jpg]

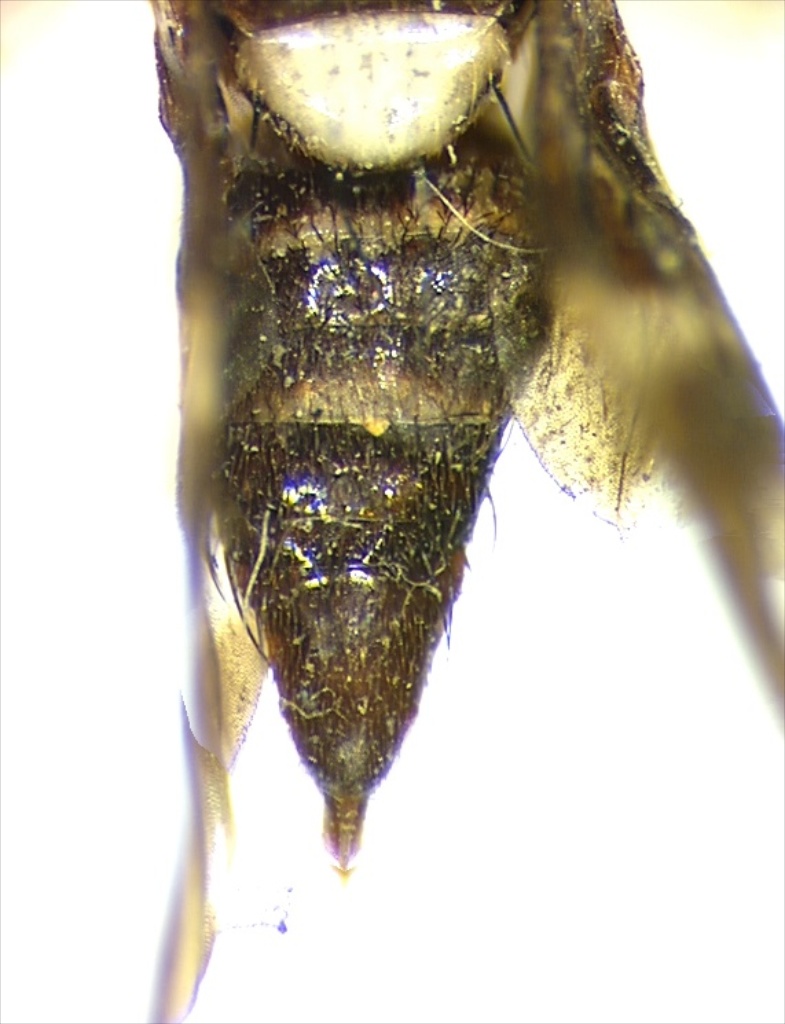

Supplement: Supplementary material 5 — Key to Carpophthoromyia [file zookeys-428-097-s005.zip › SF5_ZooKeys_key to Carpophthoromyia/key/SF5_ZooKeys_key to Carpophthoromyia/Media/Images/367 abdomen lateral (automontage (c) RMCA).jpg]

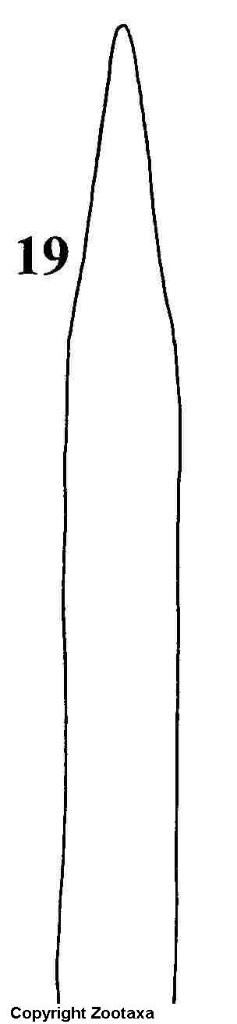

Supplement: Supplementary material 5 — Key to Carpophthoromyia [file zookeys-428-097-s005.zip › SF5_ZooKeys_key to Carpophthoromyia/key/SF5_ZooKeys_key to Carpophthoromyia/Media/Images/367 aculeus dorsal (drawing (c) Zootaxa).jpg]

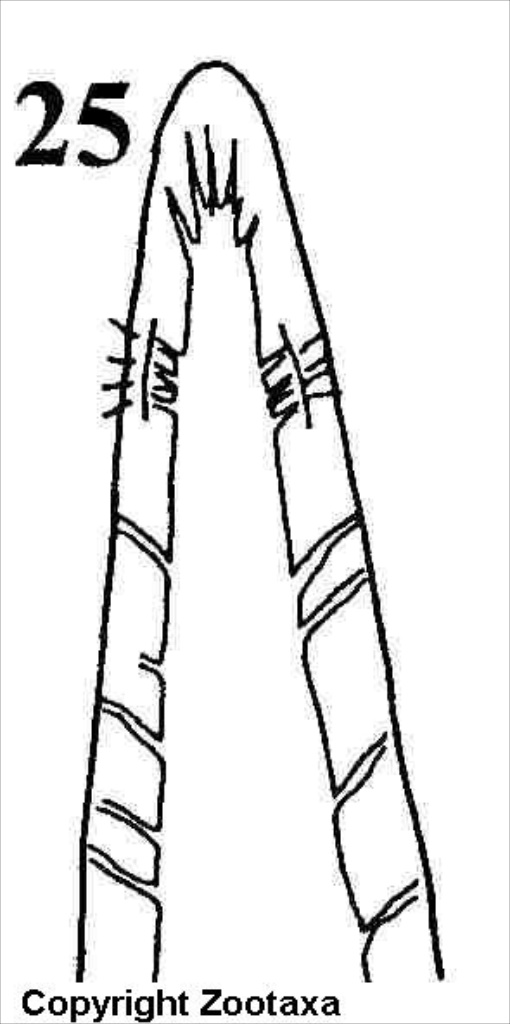

Supplement: Supplementary material 5 — Key to Carpophthoromyia [file zookeys-428-097-s005.zip › SF5_ZooKeys_key to Carpophthoromyia/key/SF5_ZooKeys_key to Carpophthoromyia/Media/Images/367 aculeus tip dorsal (drawing (c) Zootaxa).jpg]

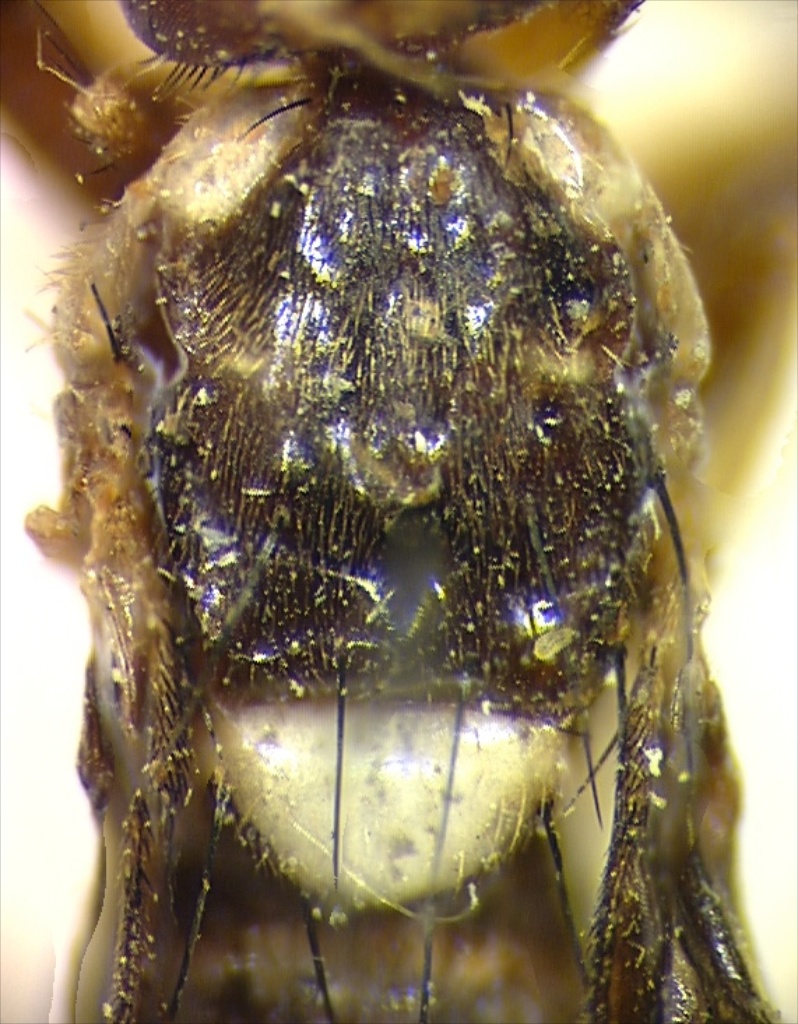

Supplement: Supplementary material 5 — Key to Carpophthoromyia [file zookeys-428-097-s005.zip › SF5_ZooKeys_key to Carpophthoromyia/key/SF5_ZooKeys_key to Carpophthoromyia/Media/Images/367 mesonotum dorsal (automontage (c) RMCA).jpg]

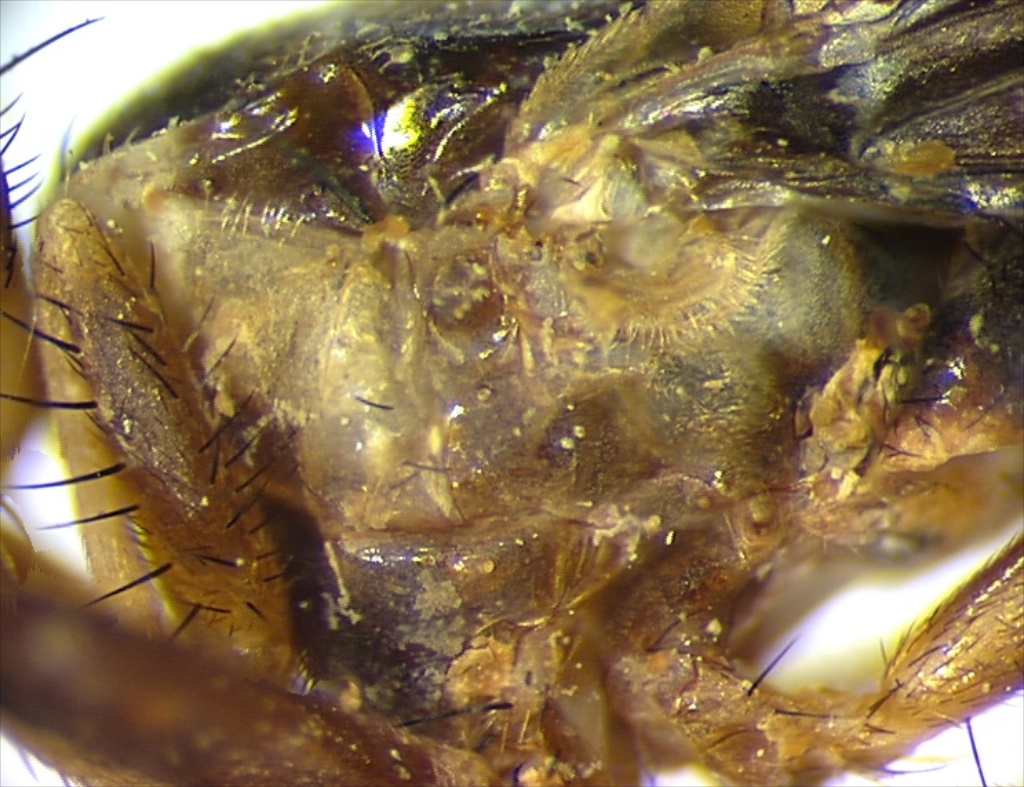

Supplement: Supplementary material 5 — Key to Carpophthoromyia [file zookeys-428-097-s005.zip › SF5_ZooKeys_key to Carpophthoromyia/key/SF5_ZooKeys_key to Carpophthoromyia/Media/Images/367 thorax lateral (automontage (c) RMCA).jpg]

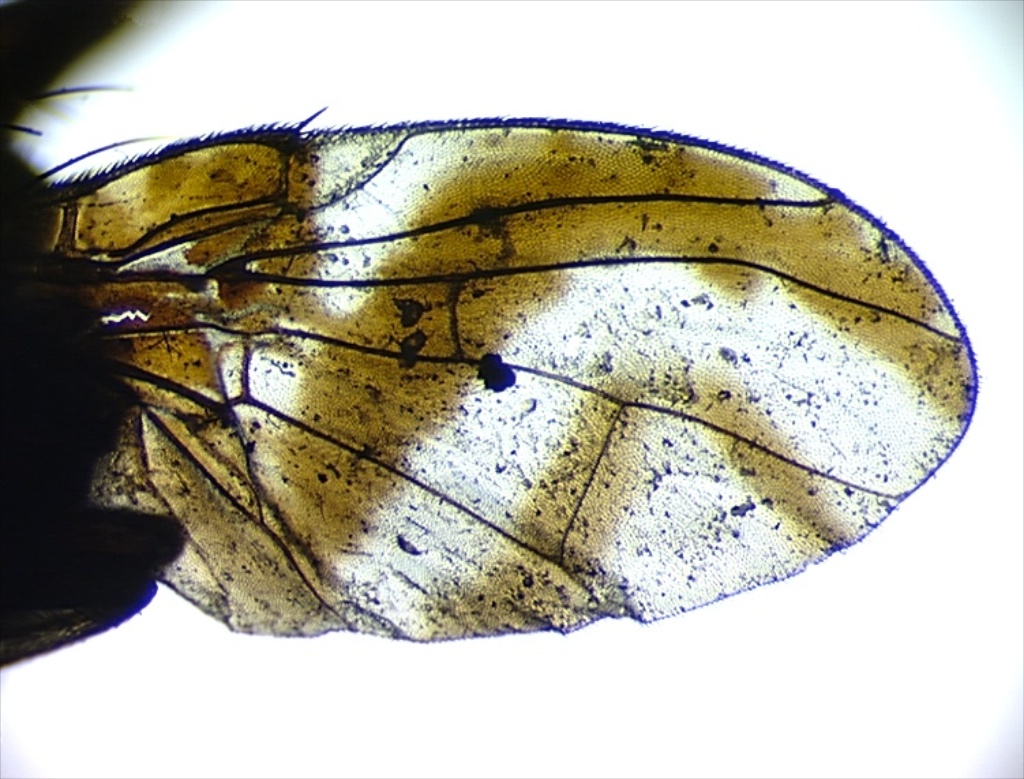

Supplement: Supplementary material 5 — Key to Carpophthoromyia [file zookeys-428-097-s005.zip › SF5_ZooKeys_key to Carpophthoromyia/key/SF5_ZooKeys_key to Carpophthoromyia/Media/Images/367 wing dorsal (automontage (c) RMCA).jpg]

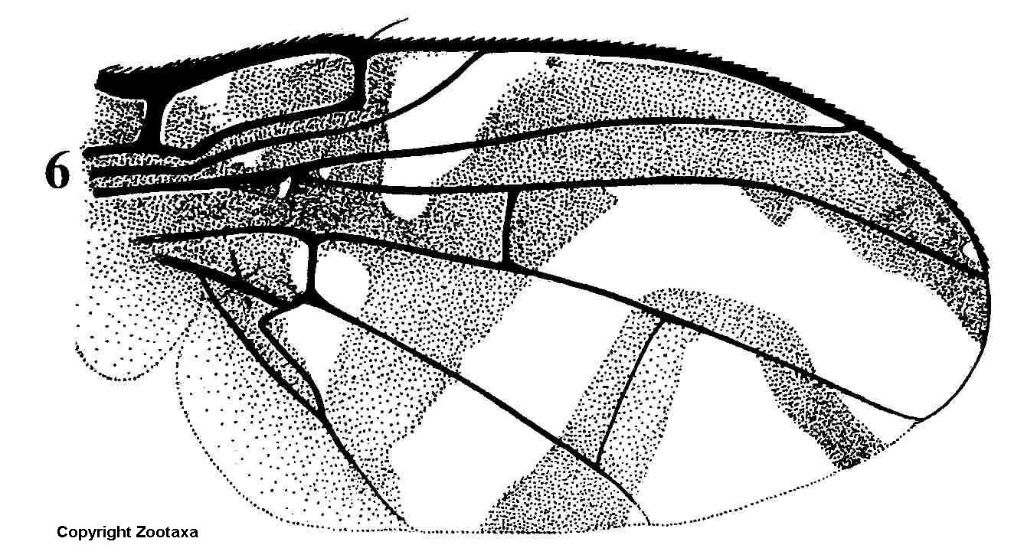

Supplement: Supplementary material 5 — Key to Carpophthoromyia [file zookeys-428-097-s005.zip › SF5_ZooKeys_key to Carpophthoromyia/key/SF5_ZooKeys_key to Carpophthoromyia/Media/Images/367 wing dorsal (drawing (c) Zootaxa).jpg]

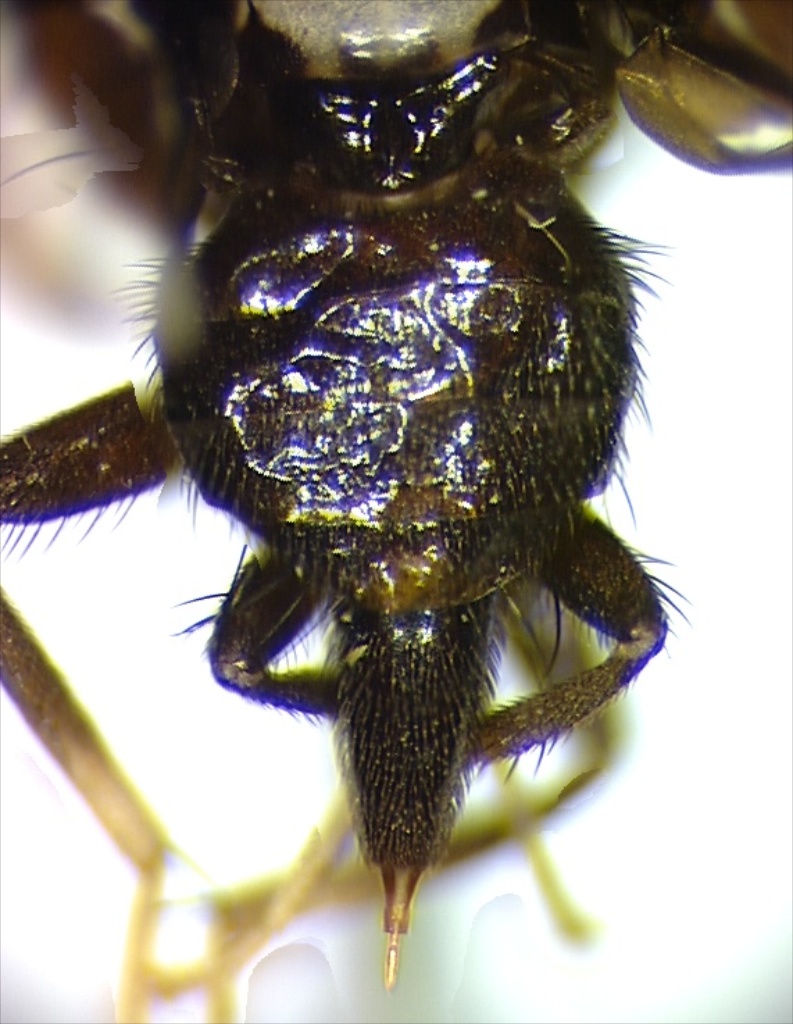

Supplement: Supplementary material 5 — Key to Carpophthoromyia [file zookeys-428-097-s005.zip › SF5_ZooKeys_key to Carpophthoromyia/key/SF5_ZooKeys_key to Carpophthoromyia/Media/Images/368 abdomen dorsal (automontage (c) RMCA).jpg]

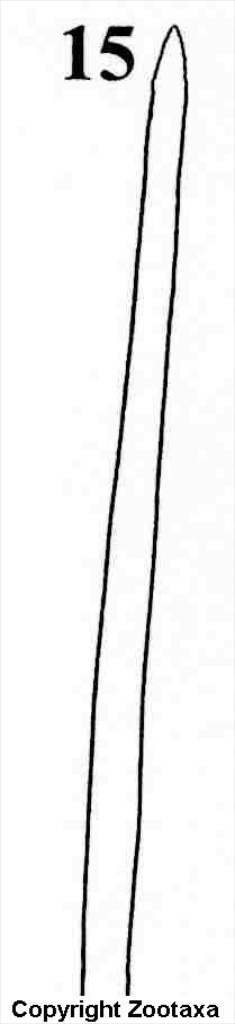

Supplement: Supplementary material 5 — Key to Carpophthoromyia [file zookeys-428-097-s005.zip › SF5_ZooKeys_key to Carpophthoromyia/key/SF5_ZooKeys_key to Carpophthoromyia/Media/Images/368 aculeus dorsal (drawing (c) Zootaxa).jpg]

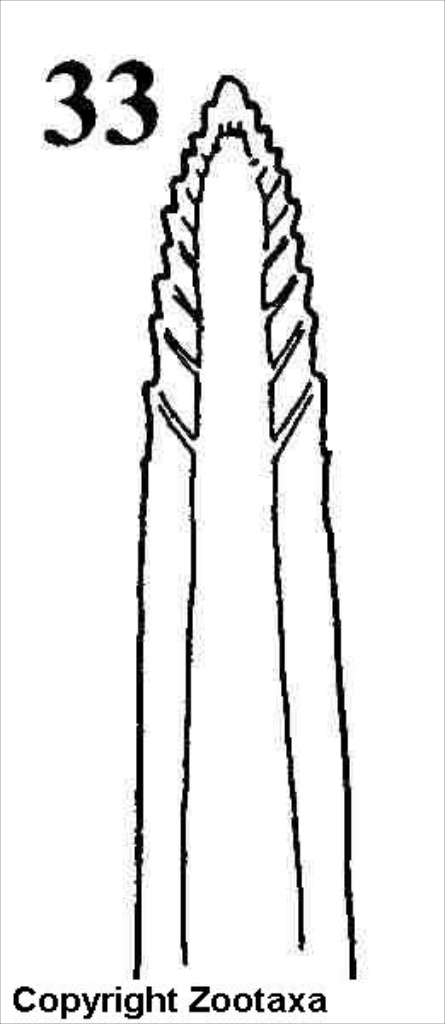

Supplement: Supplementary material 5 — Key to Carpophthoromyia [file zookeys-428-097-s005.zip › SF5_ZooKeys_key to Carpophthoromyia/key/SF5_ZooKeys_key to Carpophthoromyia/Media/Images/368 aculeus tip dorsal (drawing (c) Zootaxa).jpg]

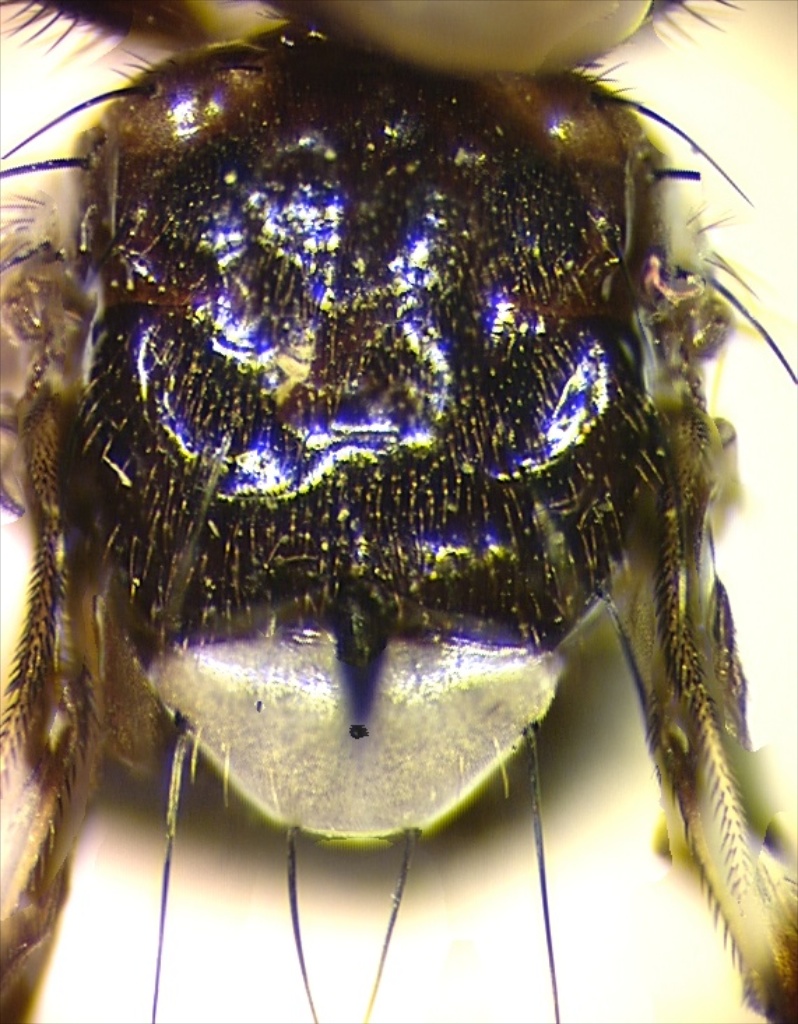

Supplement: Supplementary material 5 — Key to Carpophthoromyia [file zookeys-428-097-s005.zip › SF5_ZooKeys_key to Carpophthoromyia/key/SF5_ZooKeys_key to Carpophthoromyia/Media/Images/368 mesonotum dorsal (automontage (c) RMCA).jpg]

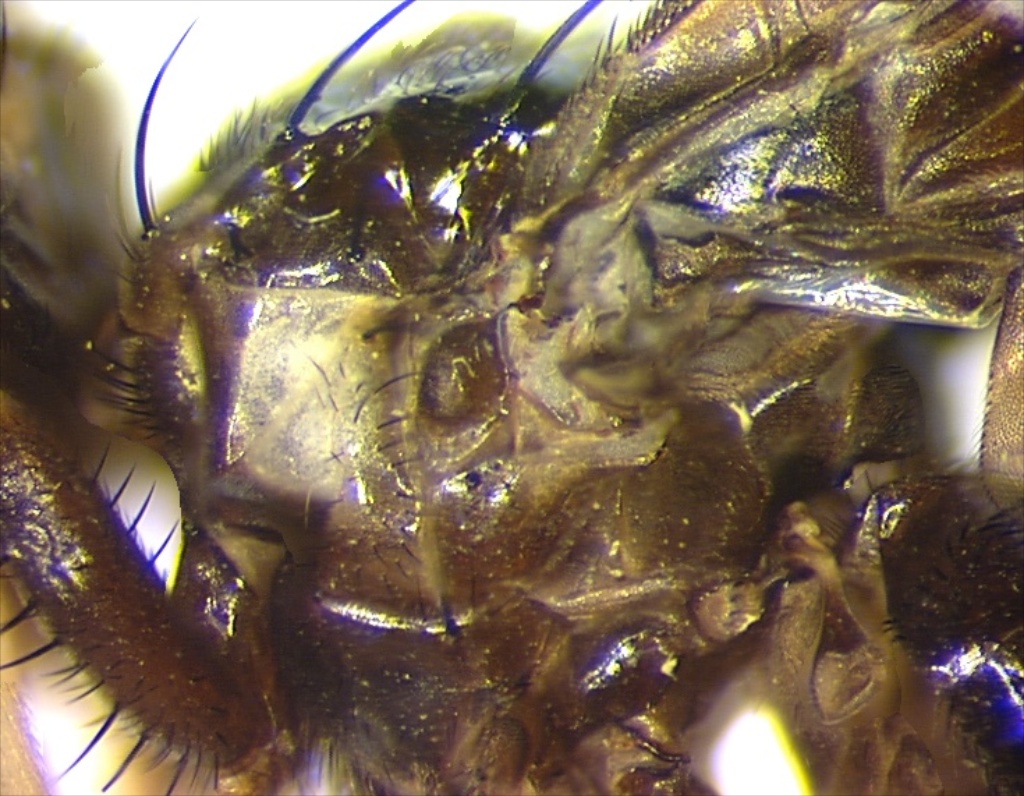

Supplement: Supplementary material 5 — Key to Carpophthoromyia [file zookeys-428-097-s005.zip › SF5_ZooKeys_key to Carpophthoromyia/key/SF5_ZooKeys_key to Carpophthoromyia/Media/Images/368 thorax lateral (automontage (c) RMCA).jpg]

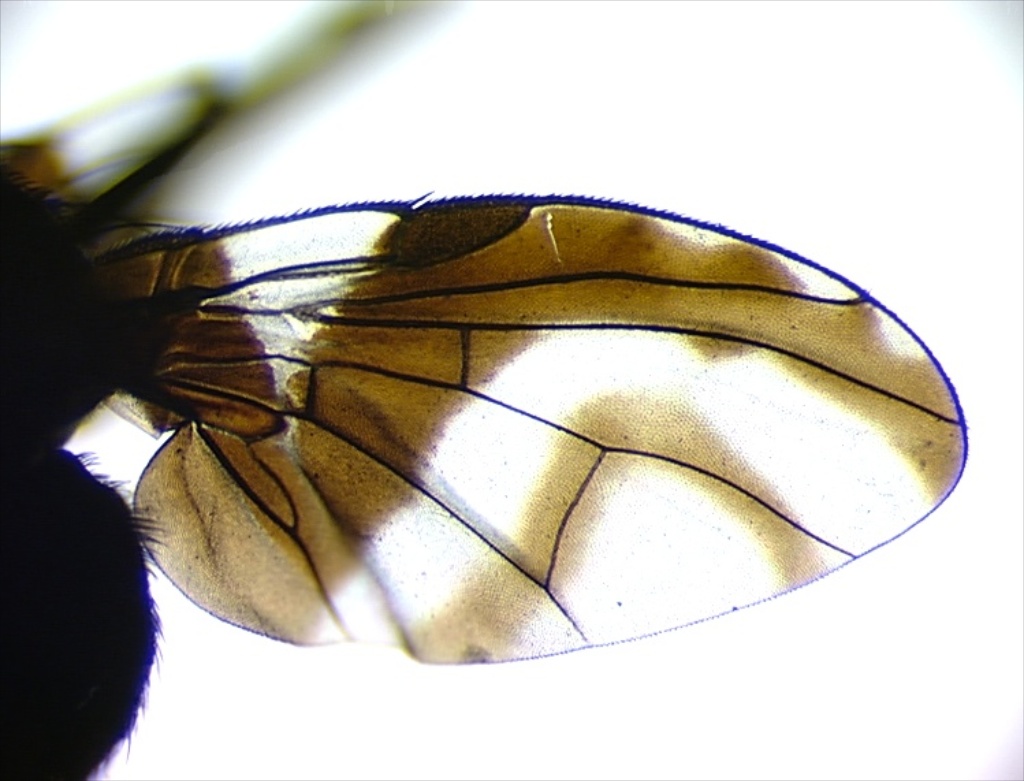

Supplement: Supplementary material 5 — Key to Carpophthoromyia [file zookeys-428-097-s005.zip › SF5_ZooKeys_key to Carpophthoromyia/key/SF5_ZooKeys_key to Carpophthoromyia/Media/Images/368 wing dorsal (automontage (c) RMCA).jpg]

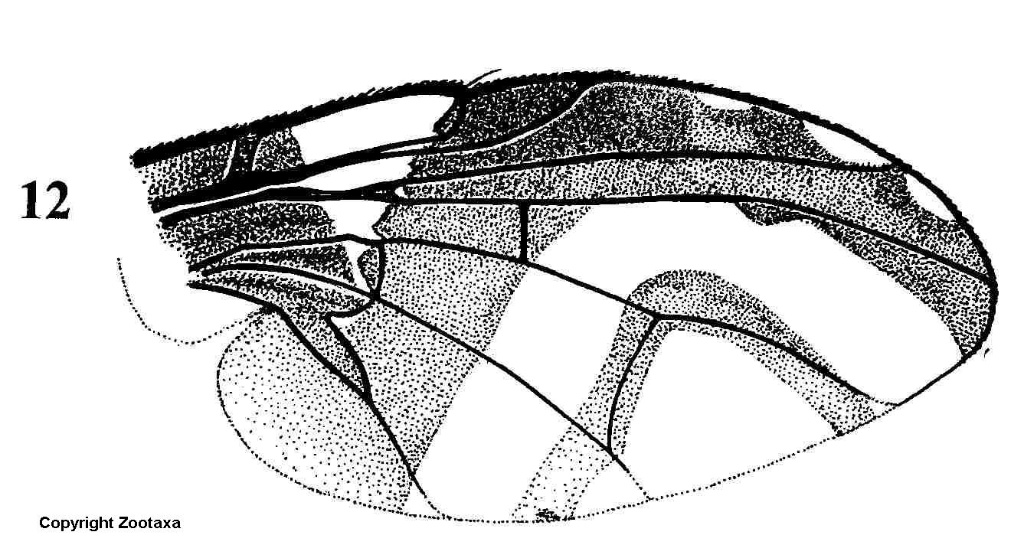

Supplement: Supplementary material 5 — Key to Carpophthoromyia [file zookeys-428-097-s005.zip › SF5_ZooKeys_key to Carpophthoromyia/key/SF5_ZooKeys_key to Carpophthoromyia/Media/Images/368 wing dorsal (drawing (c) Zootaxa).jpg]

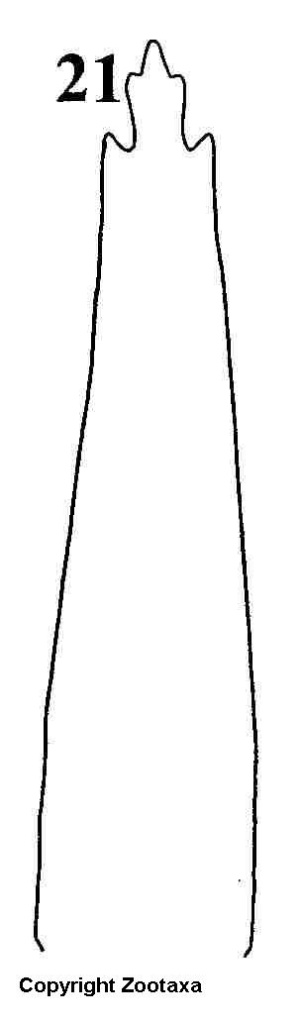

Supplement: Supplementary material 5 — Key to Carpophthoromyia [file zookeys-428-097-s005.zip › SF5_ZooKeys_key to Carpophthoromyia/key/SF5_ZooKeys_key to Carpophthoromyia/Media/Images/369 aculeus dorsal (drawing (c) Zootaxa).jpg]

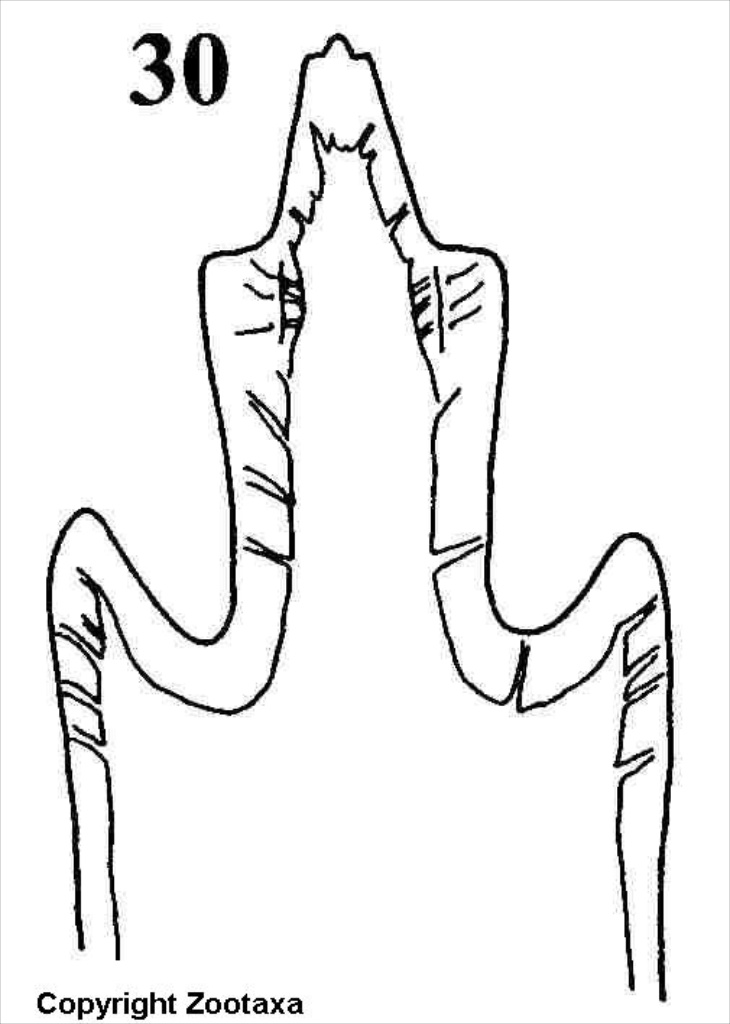

Supplement: Supplementary material 5 — Key to Carpophthoromyia [file zookeys-428-097-s005.zip › SF5_ZooKeys_key to Carpophthoromyia/key/SF5_ZooKeys_key to Carpophthoromyia/Media/Images/369 aculeus tip dorsal (drawing (c) Zootaxa).jpg]

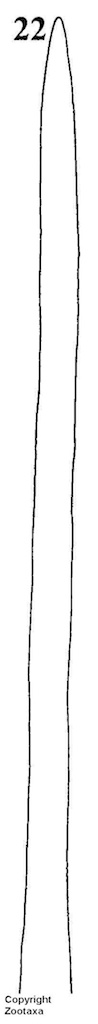

Supplement: Supplementary material 5 — Key to Carpophthoromyia [file zookeys-428-097-s005.zip › SF5_ZooKeys_key to Carpophthoromyia/key/SF5_ZooKeys_key to Carpophthoromyia/Media/Images/370 aculeus dorsal (drawing (c) Zootaxa).jpg]

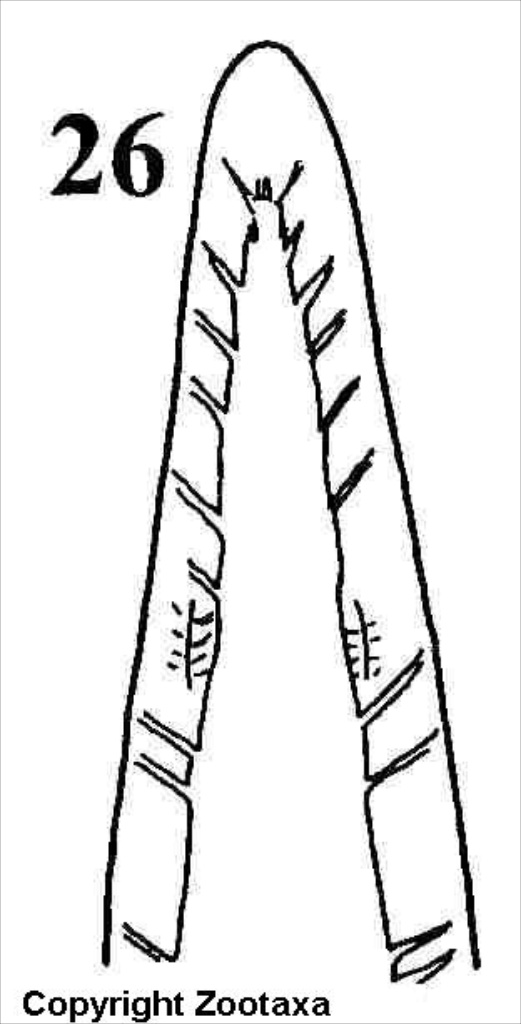

Supplement: Supplementary material 5 — Key to Carpophthoromyia [file zookeys-428-097-s005.zip › SF5_ZooKeys_key to Carpophthoromyia/key/SF5_ZooKeys_key to Carpophthoromyia/Media/Images/370 aculeus tip dorsal (drawing (c) Zootaxa).jpg]

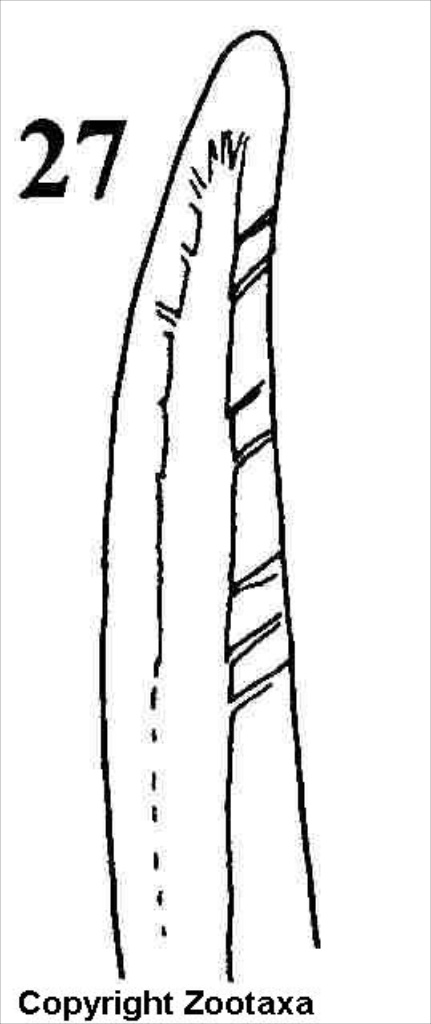

Supplement: Supplementary material 5 — Key to Carpophthoromyia [file zookeys-428-097-s005.zip › SF5_ZooKeys_key to Carpophthoromyia/key/SF5_ZooKeys_key to Carpophthoromyia/Media/Images/370 aculeus tip lateral (drawing (c) Zootaxa).jpg]

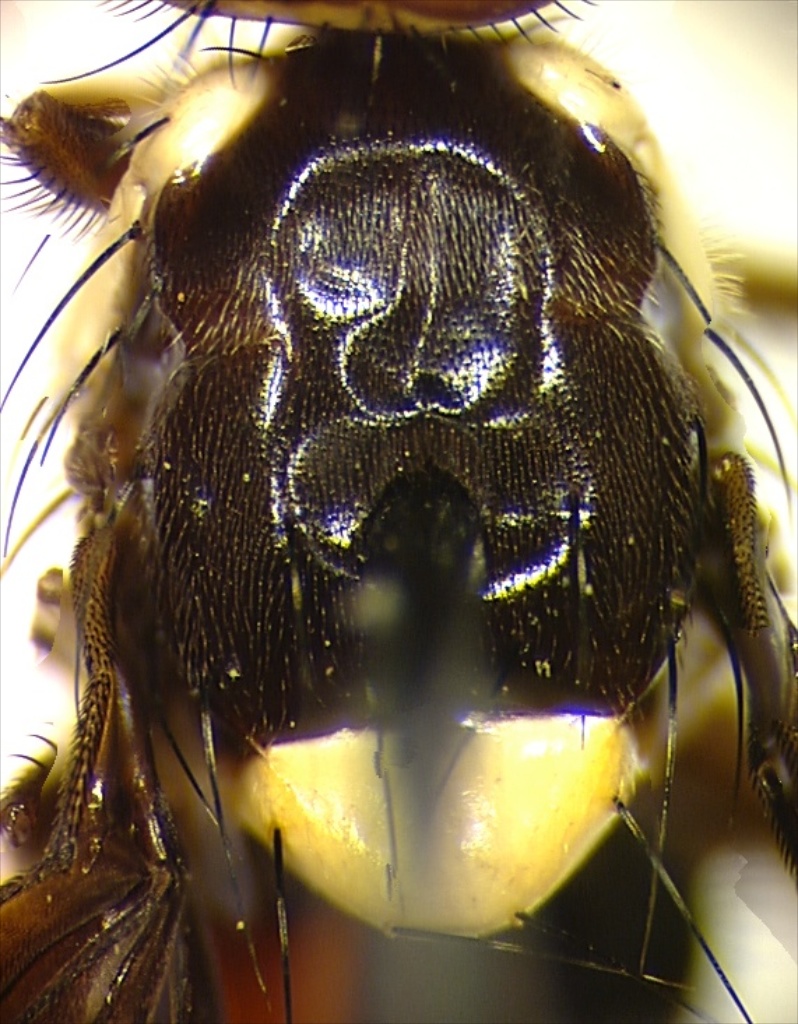

Supplement: Supplementary material 5 — Key to Carpophthoromyia [file zookeys-428-097-s005.zip › SF5_ZooKeys_key to Carpophthoromyia/key/SF5_ZooKeys_key to Carpophthoromyia/Media/Images/370 mesonotum dorsal (automontage (c) RMCA).jpg]

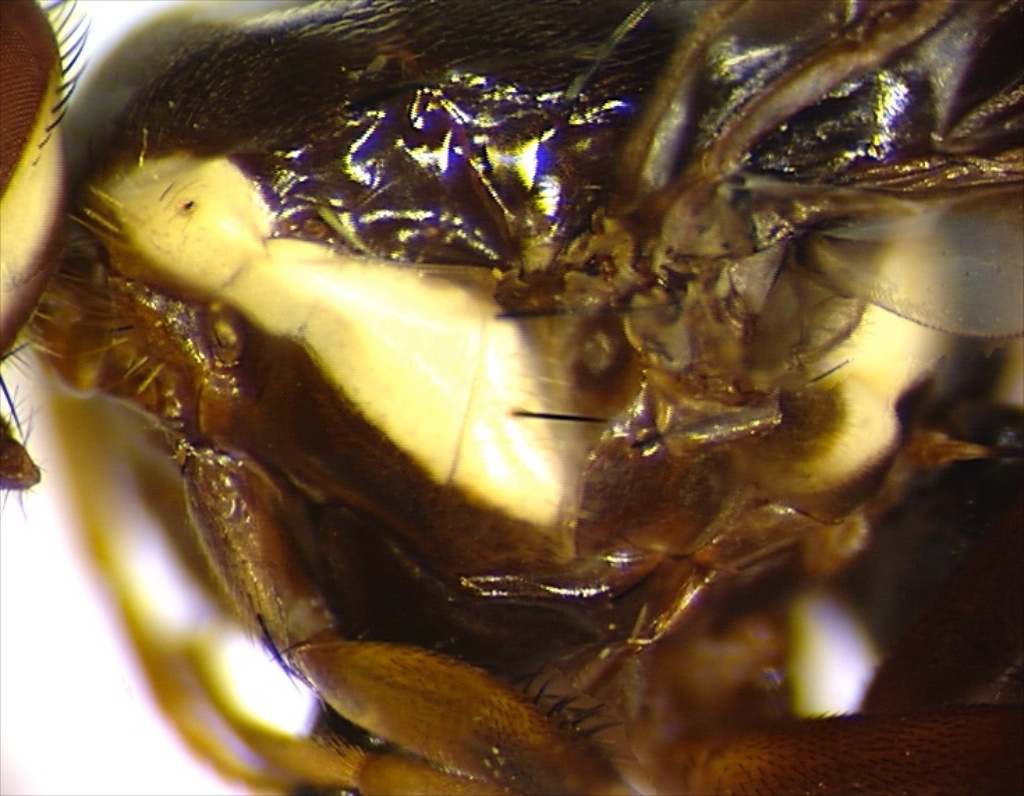

Supplement: Supplementary material 5 — Key to Carpophthoromyia [file zookeys-428-097-s005.zip › SF5_ZooKeys_key to Carpophthoromyia/key/SF5_ZooKeys_key to Carpophthoromyia/Media/Images/370 thorax lateral (automontage (c) RMCA).jpg]

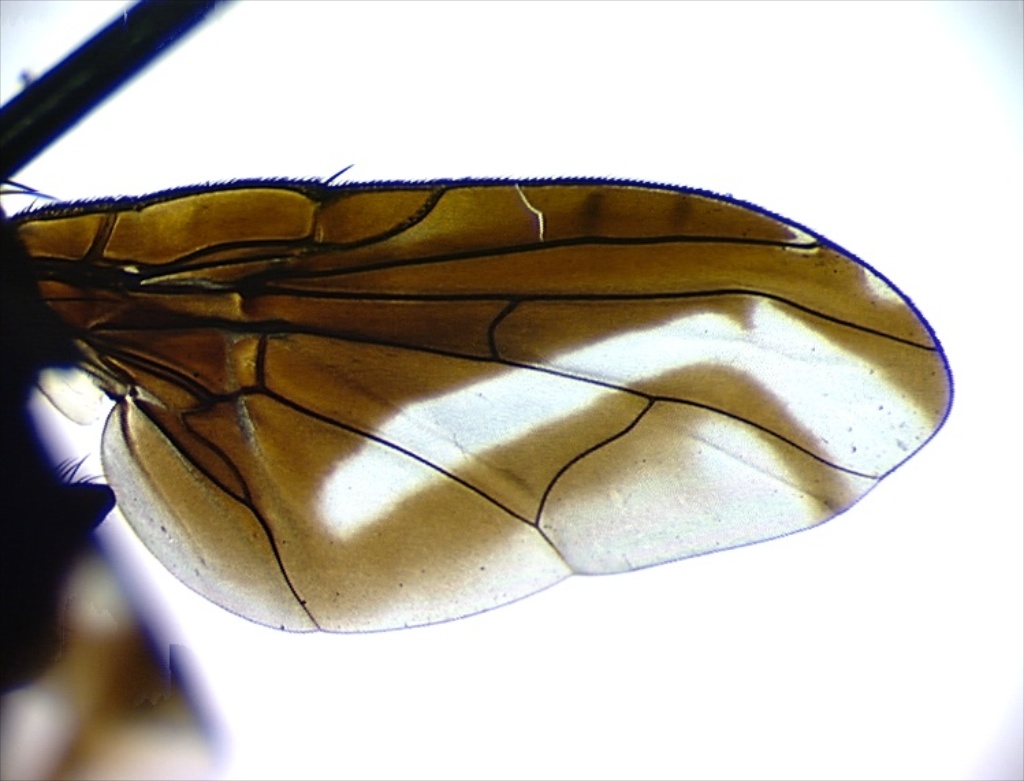

Supplement: Supplementary material 5 — Key to Carpophthoromyia [file zookeys-428-097-s005.zip › SF5_ZooKeys_key to Carpophthoromyia/key/SF5_ZooKeys_key to Carpophthoromyia/Media/Images/370 wing dorsal (automontage (c) RMCA).jpg]

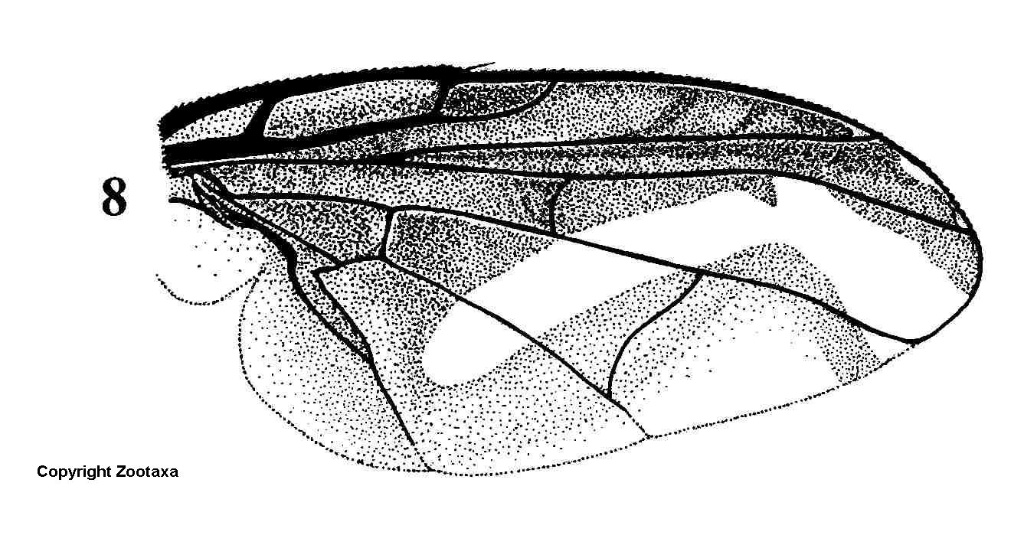

Supplement: Supplementary material 5 — Key to Carpophthoromyia [file zookeys-428-097-s005.zip › SF5_ZooKeys_key to Carpophthoromyia/key/SF5_ZooKeys_key to Carpophthoromyia/Media/Images/370 wing dorsal (drawing (c) Zootaxa).jpg]

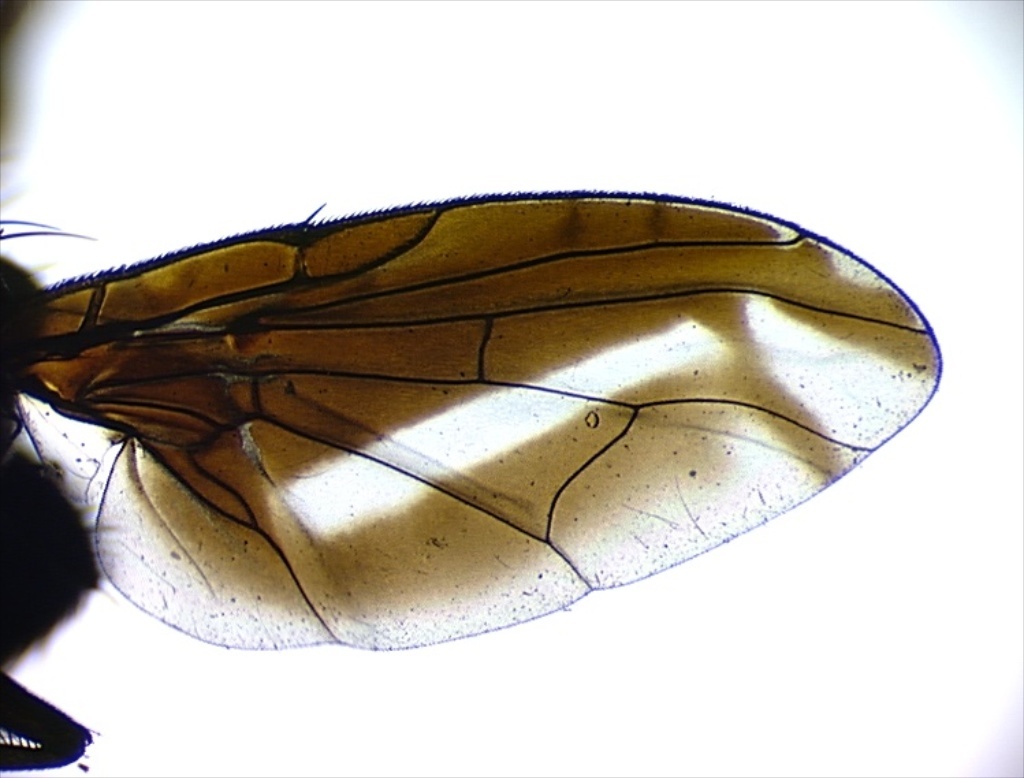

Supplement: Supplementary material 5 — Key to Carpophthoromyia [file zookeys-428-097-s005.zip › SF5_ZooKeys_key to Carpophthoromyia/key/SF5_ZooKeys_key to Carpophthoromyia/Media/Images/char10_complete_159 wing dorsal (automontage (c) RMCA).jpg]

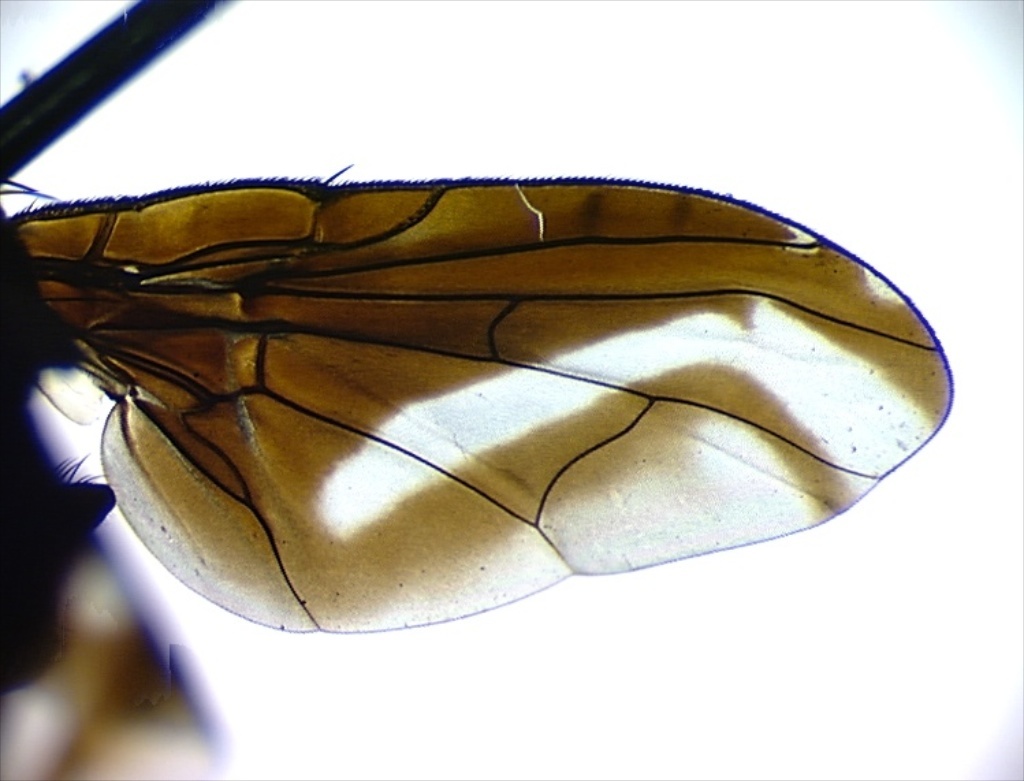

Supplement: Supplementary material 5 — Key to Carpophthoromyia [file zookeys-428-097-s005.zip › SF5_ZooKeys_key to Carpophthoromyia/key/SF5_ZooKeys_key to Carpophthoromyia/Media/Images/char10_complete_370 wing dorsal (automontage (c) RMCA).jpg]

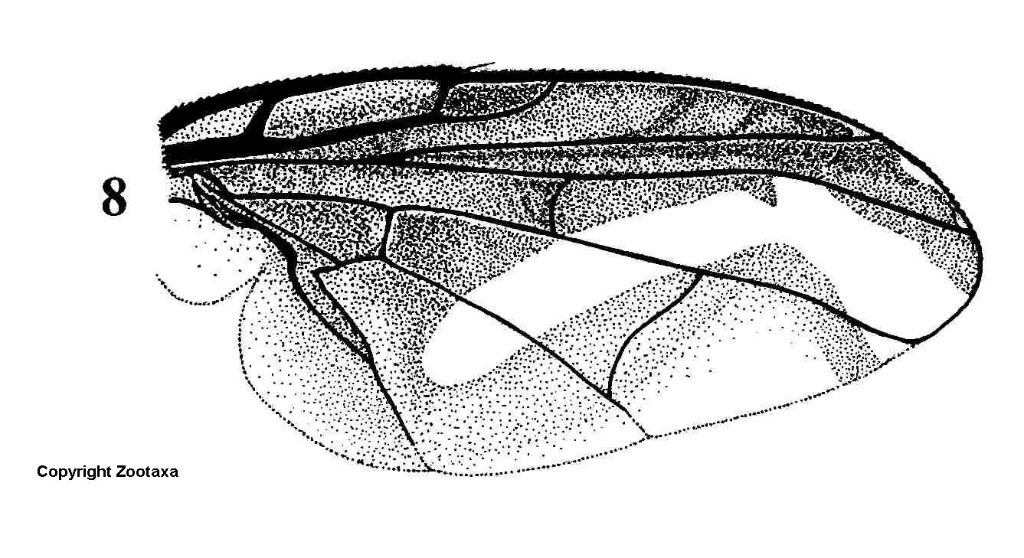

Supplement: Supplementary material 5 — Key to Carpophthoromyia [file zookeys-428-097-s005.zip › SF5_ZooKeys_key to Carpophthoromyia/key/SF5_ZooKeys_key to Carpophthoromyia/Media/Images/char10_complete_370 wing dorsal (drawing (c) Zootaxa).jpg]

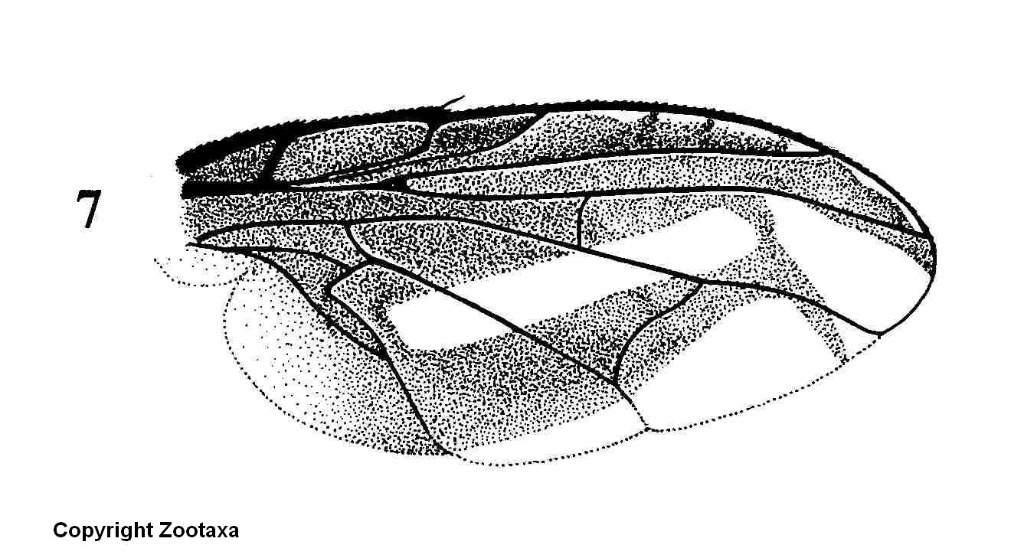

Supplement: Supplementary material 5 — Key to Carpophthoromyia [file zookeys-428-097-s005.zip › SF5_ZooKeys_key to Carpophthoromyia/key/SF5_ZooKeys_key to Carpophthoromyia/Media/Images/char10_indentation_159 wing dorsal (drawing (c) Zootaxa).jpg]

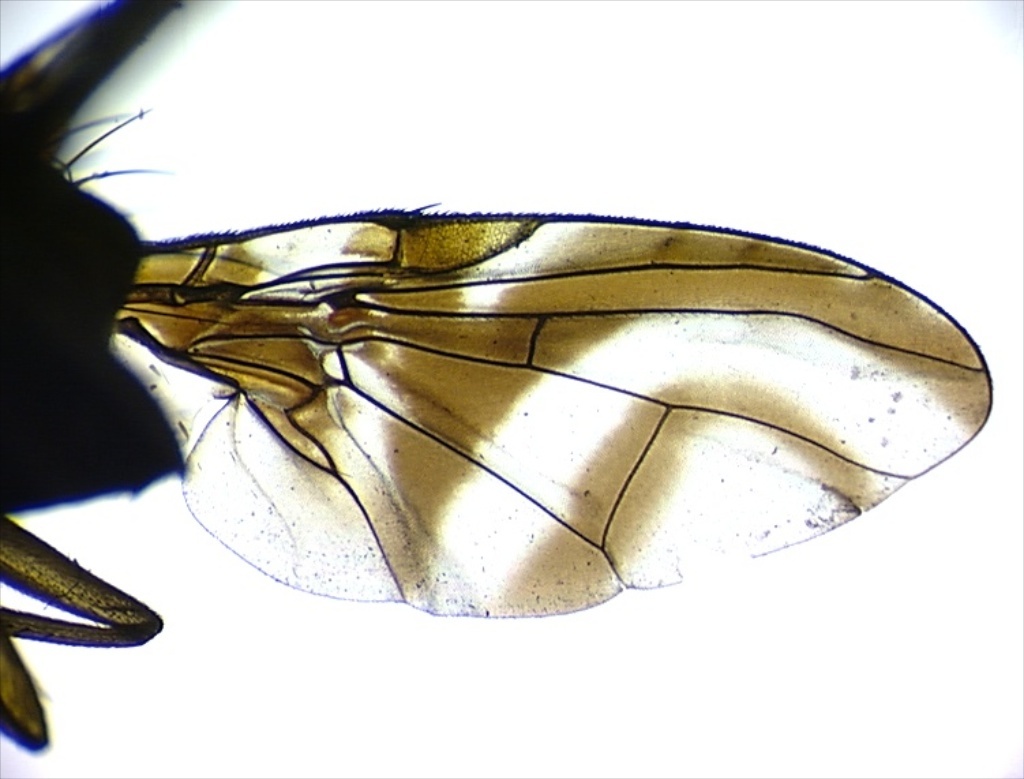

Supplement: Supplementary material 5 — Key to Carpophthoromyia [file zookeys-428-097-s005.zip › SF5_ZooKeys_key to Carpophthoromyia/key/SF5_ZooKeys_key to Carpophthoromyia/Media/Images/char10_indentation_356 wing dorsal (automontage (c) RMCA).jpg]

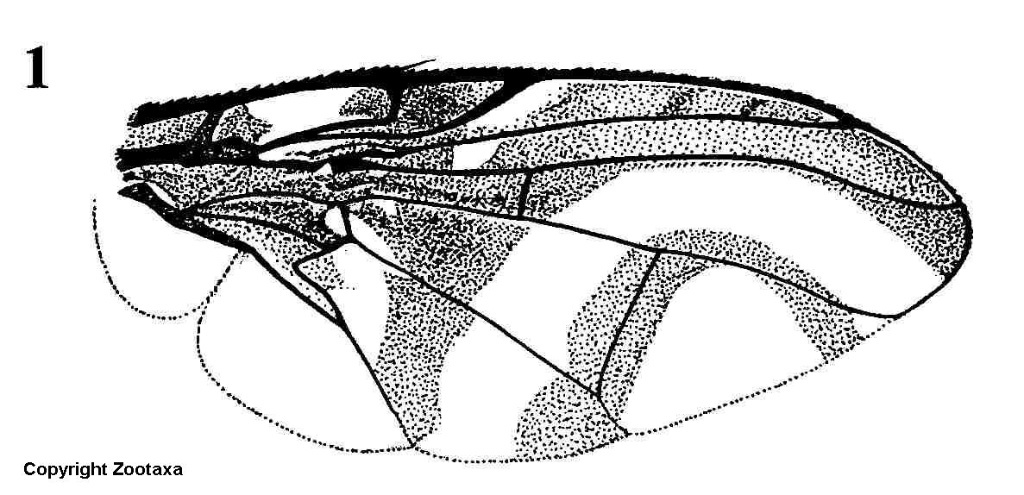

Supplement: Supplementary material 5 — Key to Carpophthoromyia [file zookeys-428-097-s005.zip › SF5_ZooKeys_key to Carpophthoromyia/key/SF5_ZooKeys_key to Carpophthoromyia/Media/Images/char10_indentation_356 wing dorsal (drawing (c) Zootaxa).jpg]

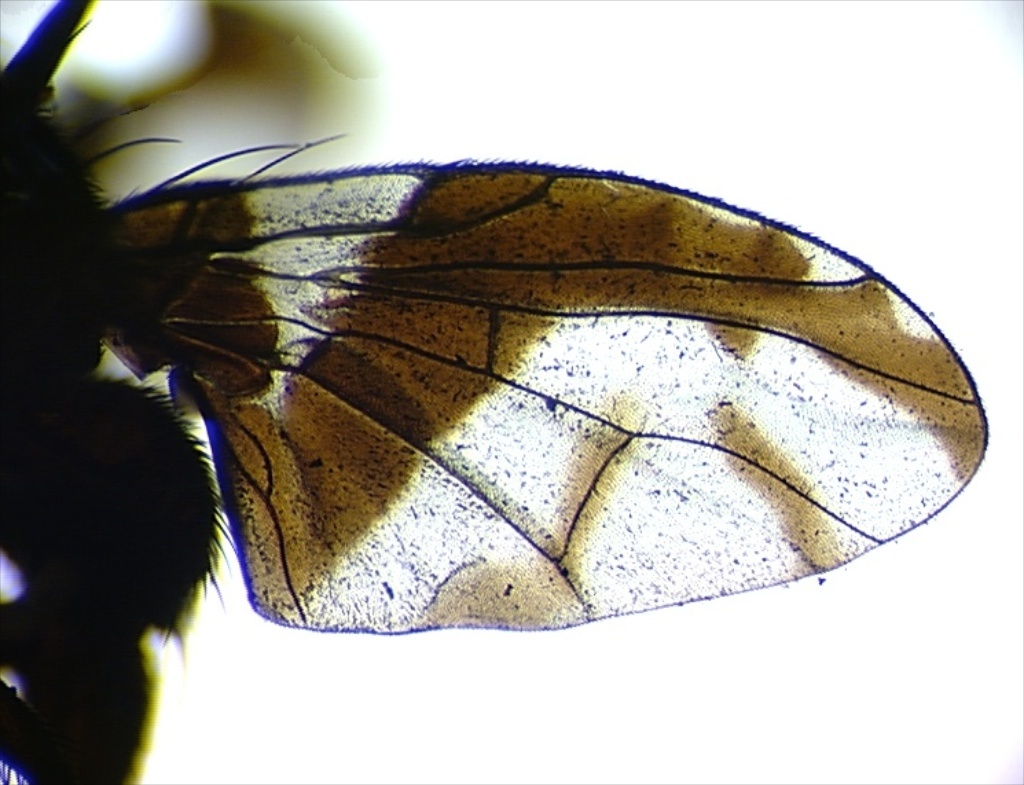

Supplement: Supplementary material 5 — Key to Carpophthoromyia [file zookeys-428-097-s005.zip › SF5_ZooKeys_key to Carpophthoromyia/key/SF5_ZooKeys_key to Carpophthoromyia/Media/Images/char10_indentation_358 wing dorsal (automontage (c) RMCA).jpg]

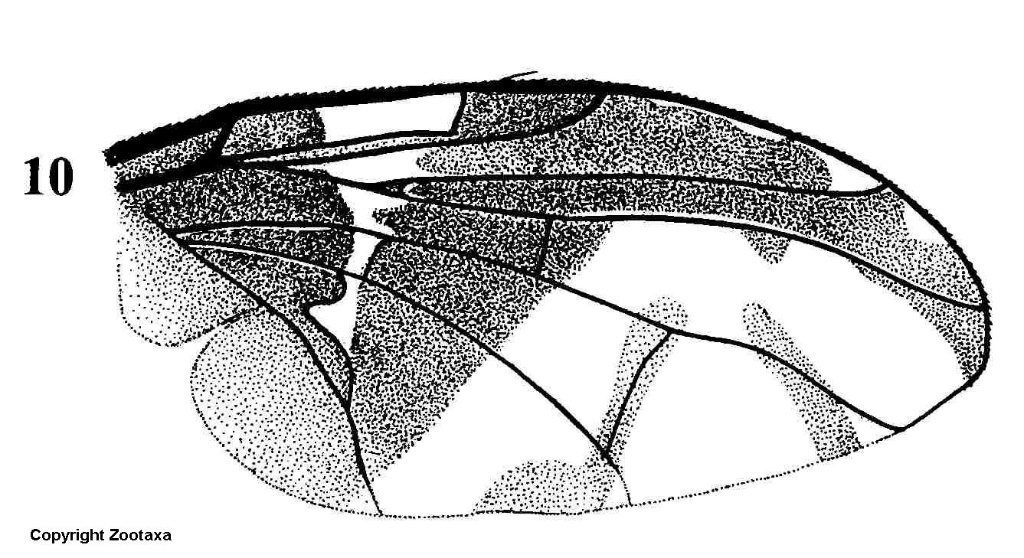

Supplement: Supplementary material 5 — Key to Carpophthoromyia [file zookeys-428-097-s005.zip › SF5_ZooKeys_key to Carpophthoromyia/key/SF5_ZooKeys_key to Carpophthoromyia/Media/Images/char10_indentation_358 wing dorsal (drawing (c) Zootaxa).jpg]

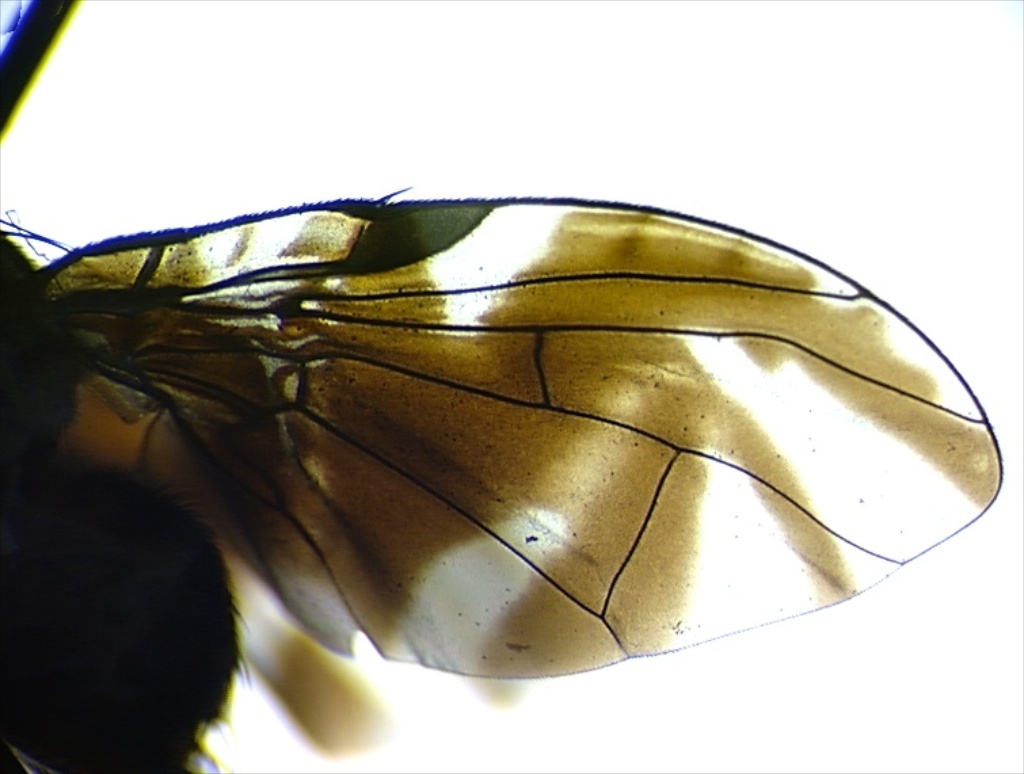

Supplement: Supplementary material 5 — Key to Carpophthoromyia [file zookeys-428-097-s005.zip › SF5_ZooKeys_key to Carpophthoromyia/key/SF5_ZooKeys_key to Carpophthoromyia/Media/Images/char10_indentation_359 wing female dorsal (automontage (c) RMCA).jpg]

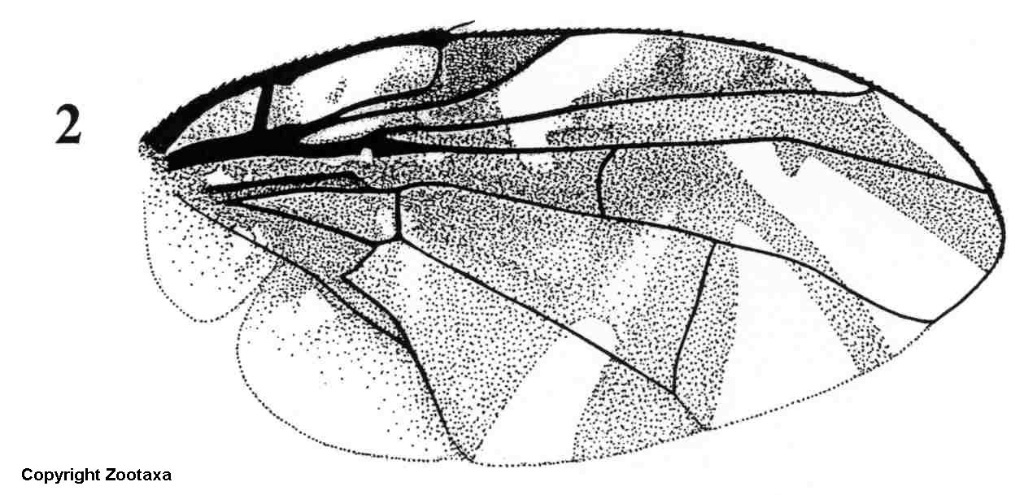

Supplement: Supplementary material 5 — Key to Carpophthoromyia [file zookeys-428-097-s005.zip › SF5_ZooKeys_key to Carpophthoromyia/key/SF5_ZooKeys_key to Carpophthoromyia/Media/Images/char10_indentation_359 wing female dorsal (drawing (c) Zootaxa).jpg]

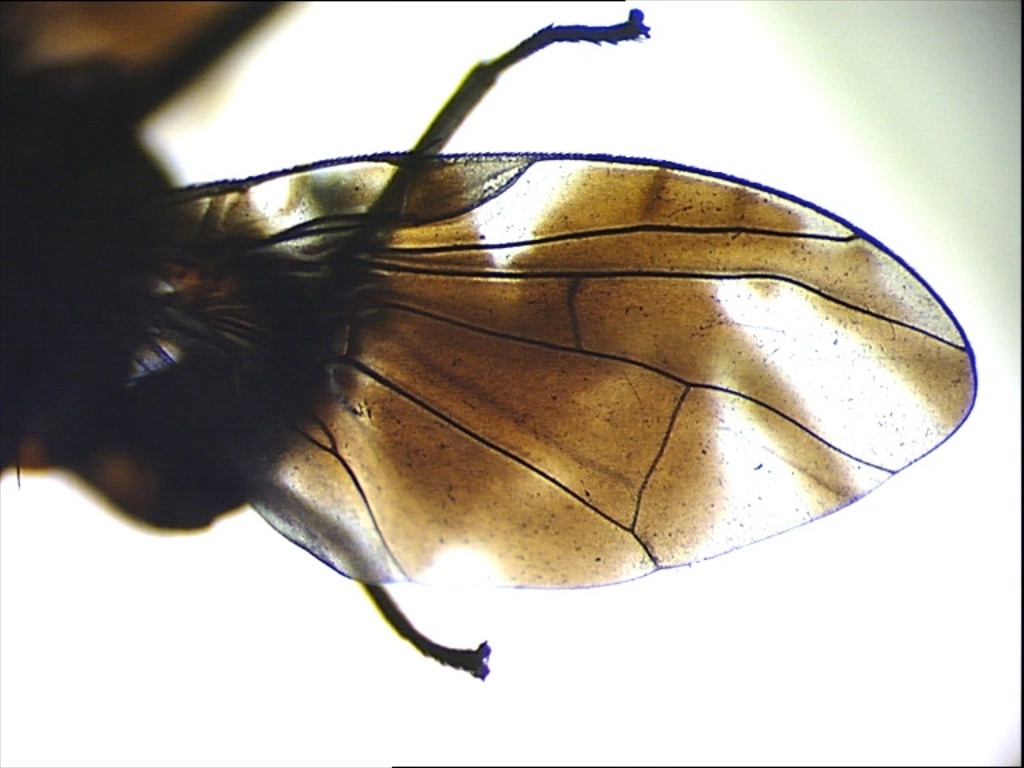

Supplement: Supplementary material 5 — Key to Carpophthoromyia [file zookeys-428-097-s005.zip › SF5_ZooKeys_key to Carpophthoromyia/key/SF5_ZooKeys_key to Carpophthoromyia/Media/Images/char10_indentation_359 wing male dorsal (automontage (c) RMCA).jpg]

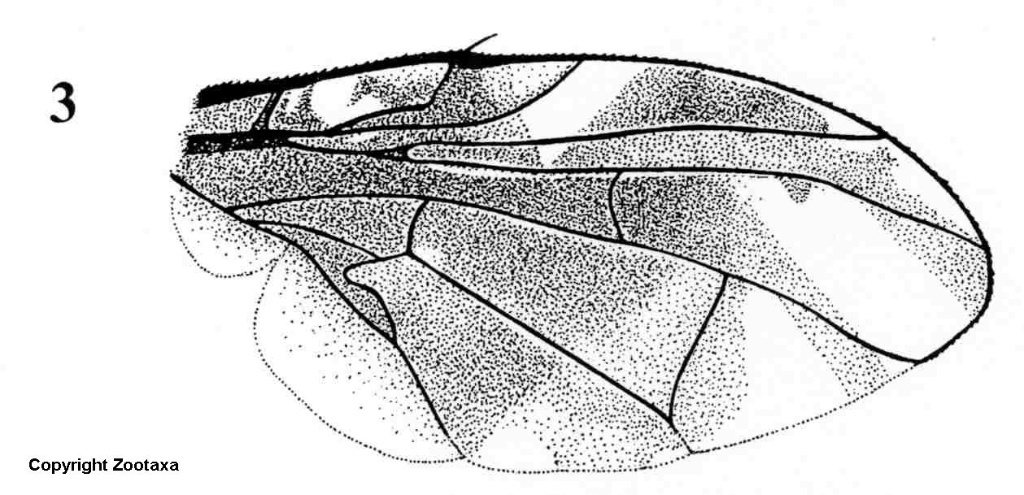

Supplement: Supplementary material 5 — Key to Carpophthoromyia [file zookeys-428-097-s005.zip › SF5_ZooKeys_key to Carpophthoromyia/key/SF5_ZooKeys_key to Carpophthoromyia/Media/Images/char10_indentation_359 wing male dorsal (drawing (c) Zootaxa).jpg]

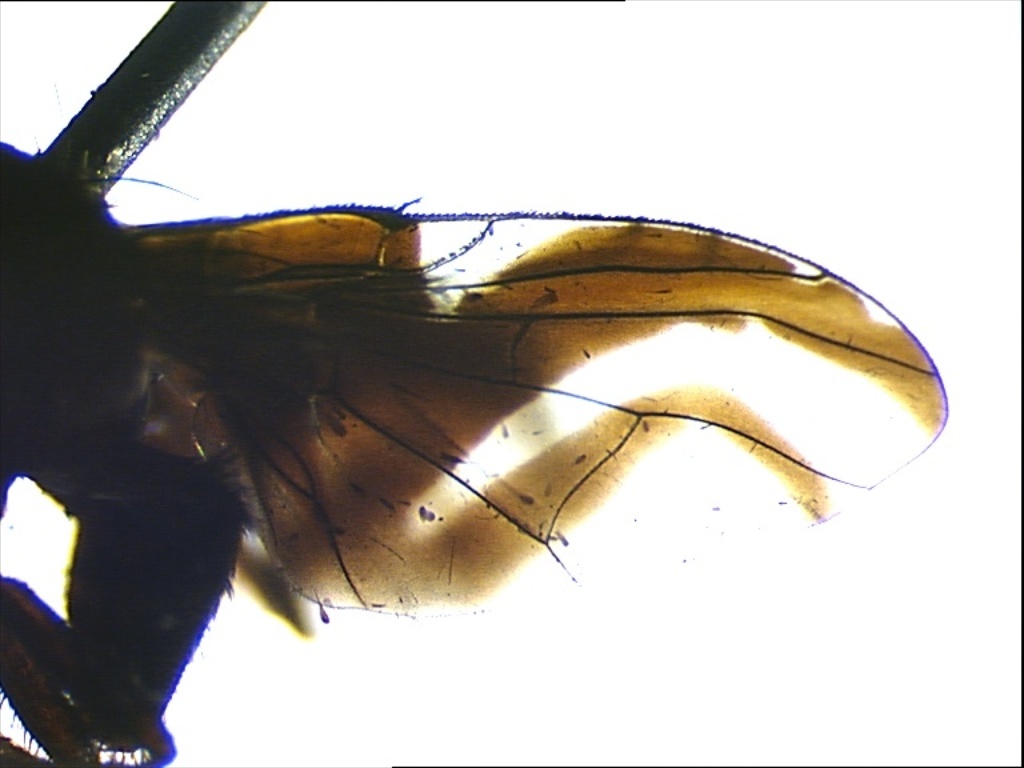

Supplement: Supplementary material 5 — Key to Carpophthoromyia [file zookeys-428-097-s005.zip › SF5_ZooKeys_key to Carpophthoromyia/key/SF5_ZooKeys_key to Carpophthoromyia/Media/Images/char10_indentation_360 wing dorsal (automontage (c) RMCA).jpg]

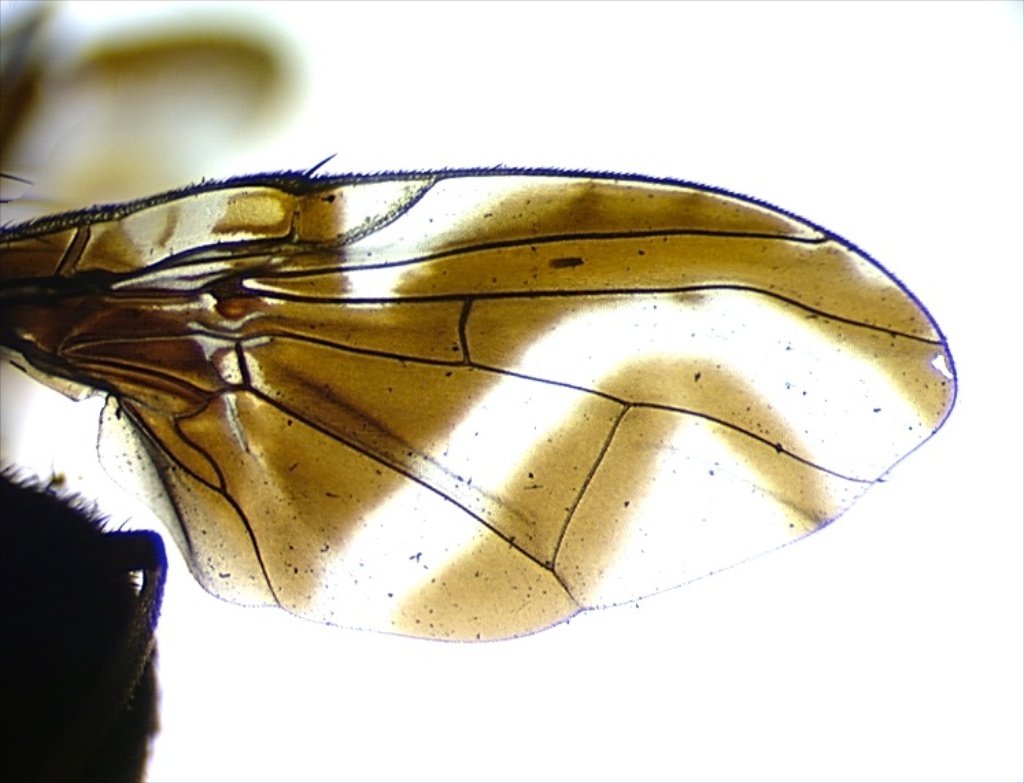

Supplement: Supplementary material 5 — Key to Carpophthoromyia [file zookeys-428-097-s005.zip › SF5_ZooKeys_key to Carpophthoromyia/key/SF5_ZooKeys_key to Carpophthoromyia/Media/Images/char10_indentation_361 wing dorsal (automontage (c) RMCA).jpg]

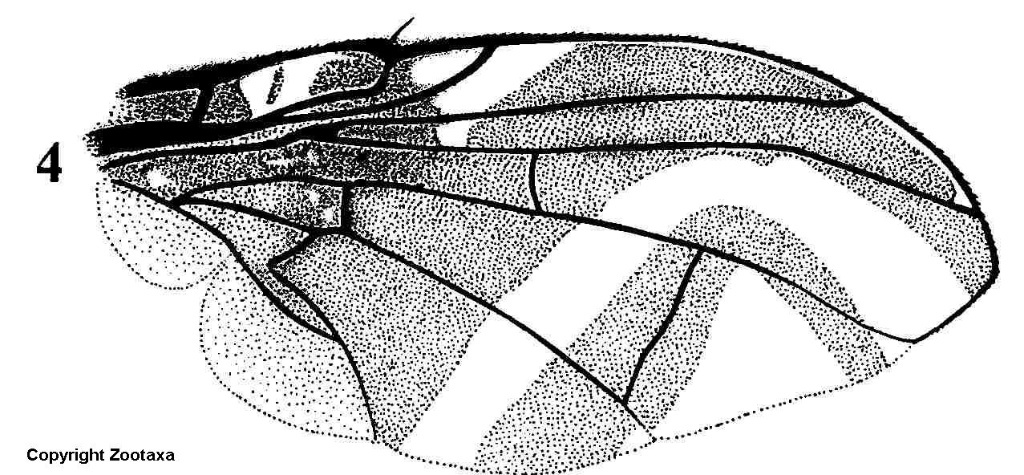

Supplement: Supplementary material 5 — Key to Carpophthoromyia [file zookeys-428-097-s005.zip › SF5_ZooKeys_key to Carpophthoromyia/key/SF5_ZooKeys_key to Carpophthoromyia/Media/Images/char10_indentation_361 wing dorsal (drawing (c) Zootaxa).jpg]

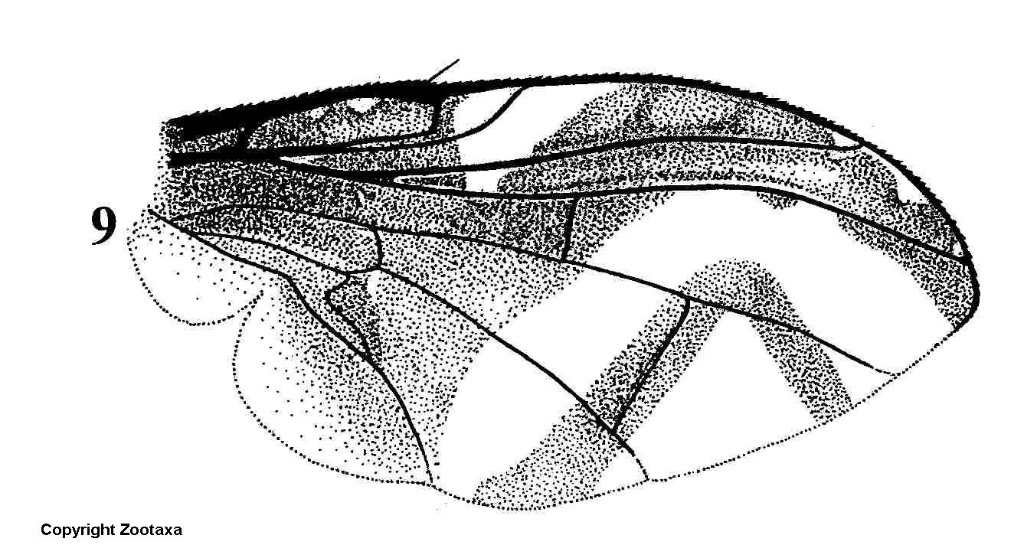

Supplement: Supplementary material 5 — Key to Carpophthoromyia [file zookeys-428-097-s005.zip › SF5_ZooKeys_key to Carpophthoromyia/key/SF5_ZooKeys_key to Carpophthoromyia/Media/Images/char10_indentation_362 wing dorsal (drawing (c) Zootaxa).jpg]

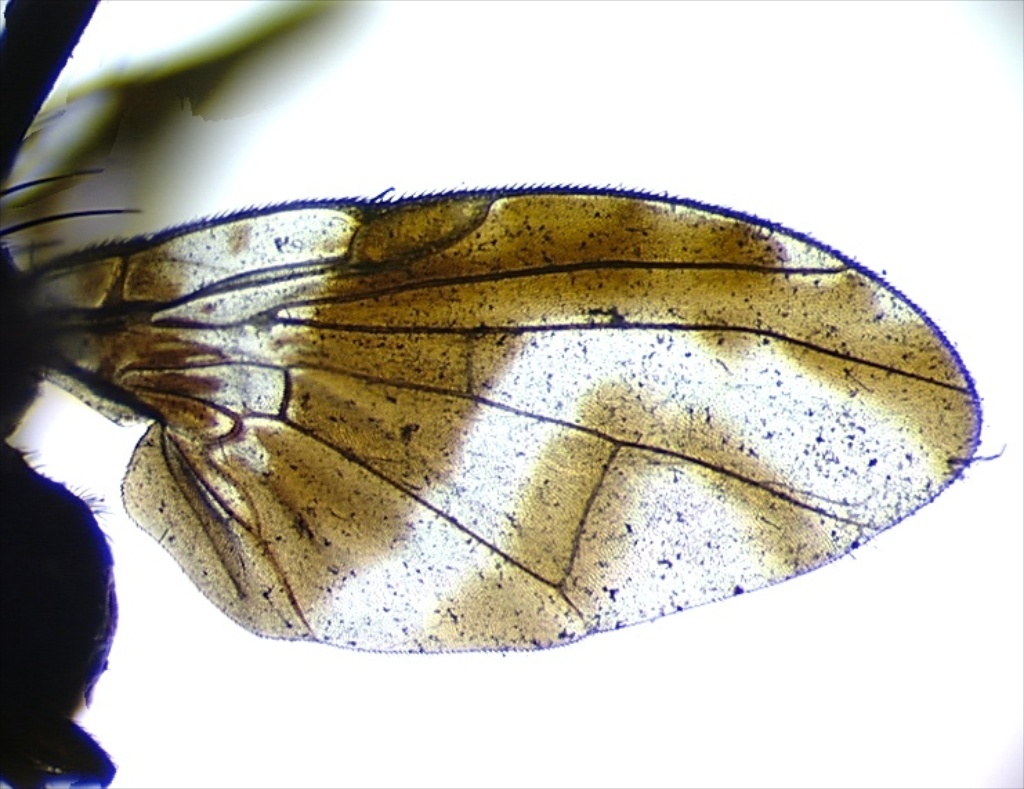

Supplement: Supplementary material 5 — Key to Carpophthoromyia [file zookeys-428-097-s005.zip › SF5_ZooKeys_key to Carpophthoromyia/key/SF5_ZooKeys_key to Carpophthoromyia/Media/Images/char10_indentation_364 wing dorsal (automontage (c) RMCA).jpg]

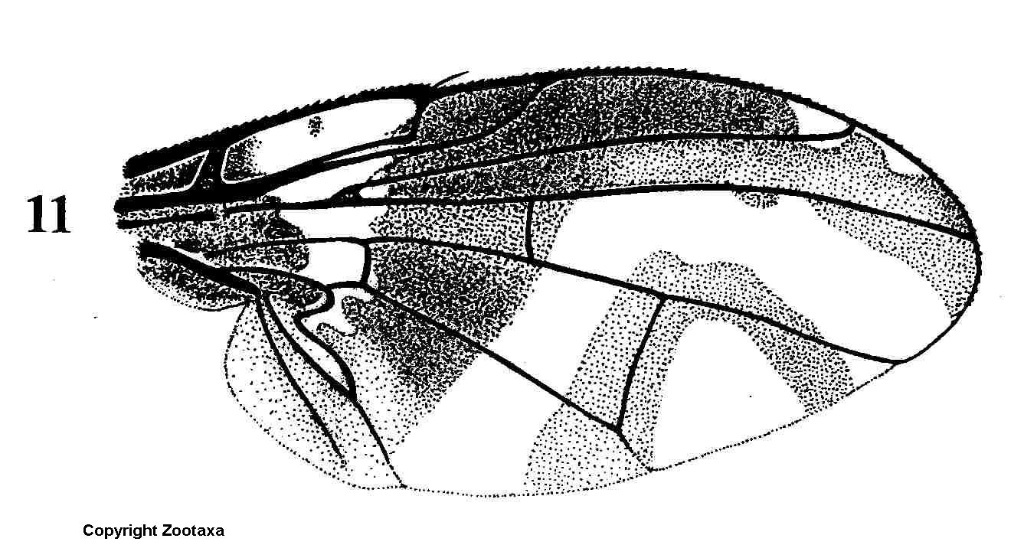

Supplement: Supplementary material 5 — Key to Carpophthoromyia [file zookeys-428-097-s005.zip › SF5_ZooKeys_key to Carpophthoromyia/key/SF5_ZooKeys_key to Carpophthoromyia/Media/Images/char10_indentation_364 wing dorsal (drawing (c) Zootaxa).jpg]

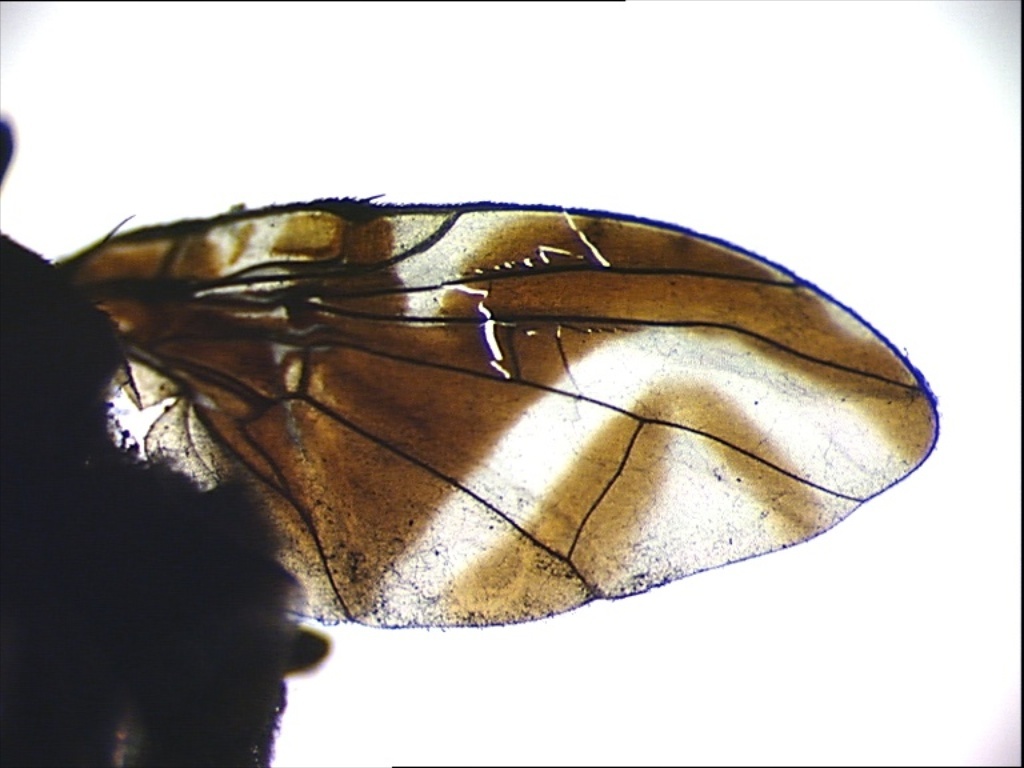

Supplement: Supplementary material 5 — Key to Carpophthoromyia [file zookeys-428-097-s005.zip › SF5_ZooKeys_key to Carpophthoromyia/key/SF5_ZooKeys_key to Carpophthoromyia/Media/Images/char10_indentation_365 wing dorsal (automontage (c) RMCA).jpg]

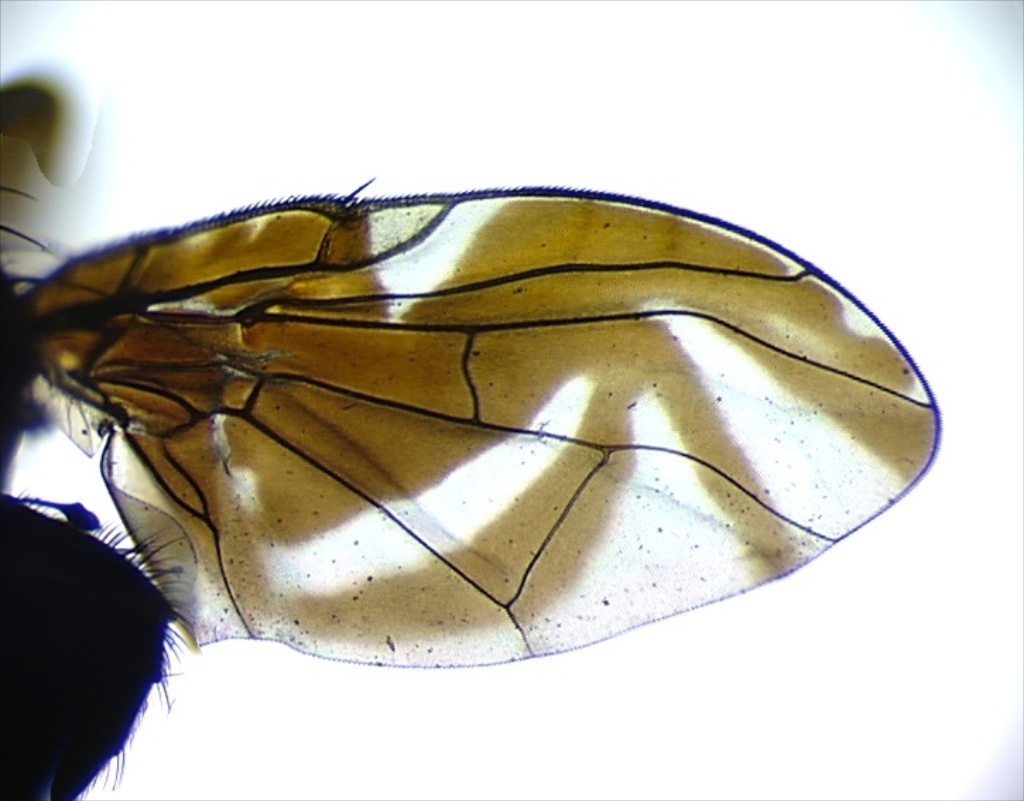

Supplement: Supplementary material 5 — Key to Carpophthoromyia [file zookeys-428-097-s005.zip › SF5_ZooKeys_key to Carpophthoromyia/key/SF5_ZooKeys_key to Carpophthoromyia/Media/Images/char10_indentation_366 wing dorsal (automontage (c) RMCA).jpg]

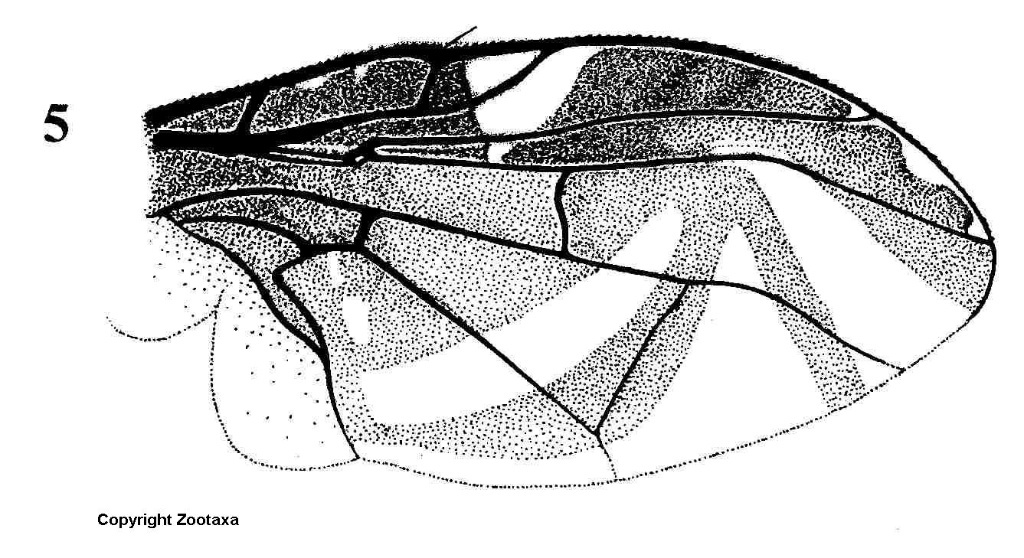

Supplement: Supplementary material 5 — Key to Carpophthoromyia [file zookeys-428-097-s005.zip › SF5_ZooKeys_key to Carpophthoromyia/key/SF5_ZooKeys_key to Carpophthoromyia/Media/Images/char10_indentation_366 wing dorsal (drawing (c) Zootaxa).jpg]

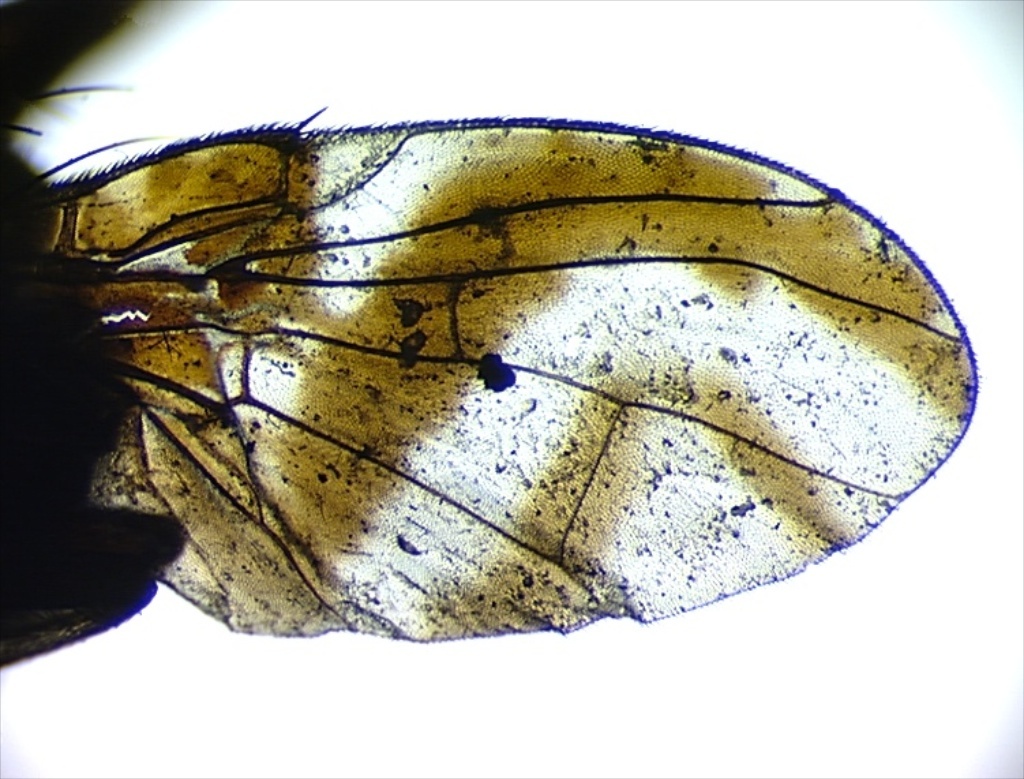

Supplement: Supplementary material 5 — Key to Carpophthoromyia [file zookeys-428-097-s005.zip › SF5_ZooKeys_key to Carpophthoromyia/key/SF5_ZooKeys_key to Carpophthoromyia/Media/Images/char10_indentation_367 wing dorsal (automontage (c) RMCA).jpg]

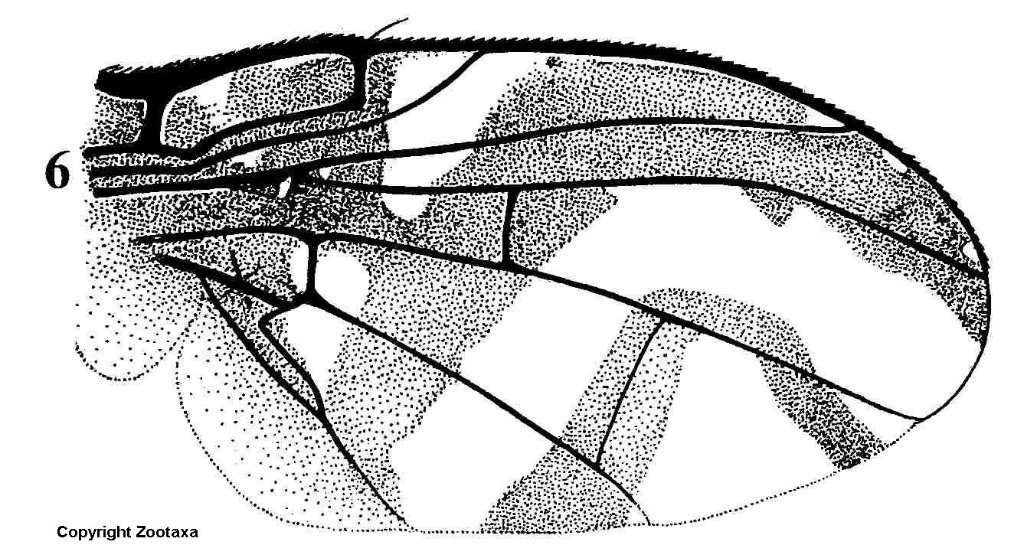

Supplement: Supplementary material 5 — Key to Carpophthoromyia [file zookeys-428-097-s005.zip › SF5_ZooKeys_key to Carpophthoromyia/key/SF5_ZooKeys_key to Carpophthoromyia/Media/Images/char10_indentation_367 wing dorsal (drawing (c) Zootaxa).jpg]

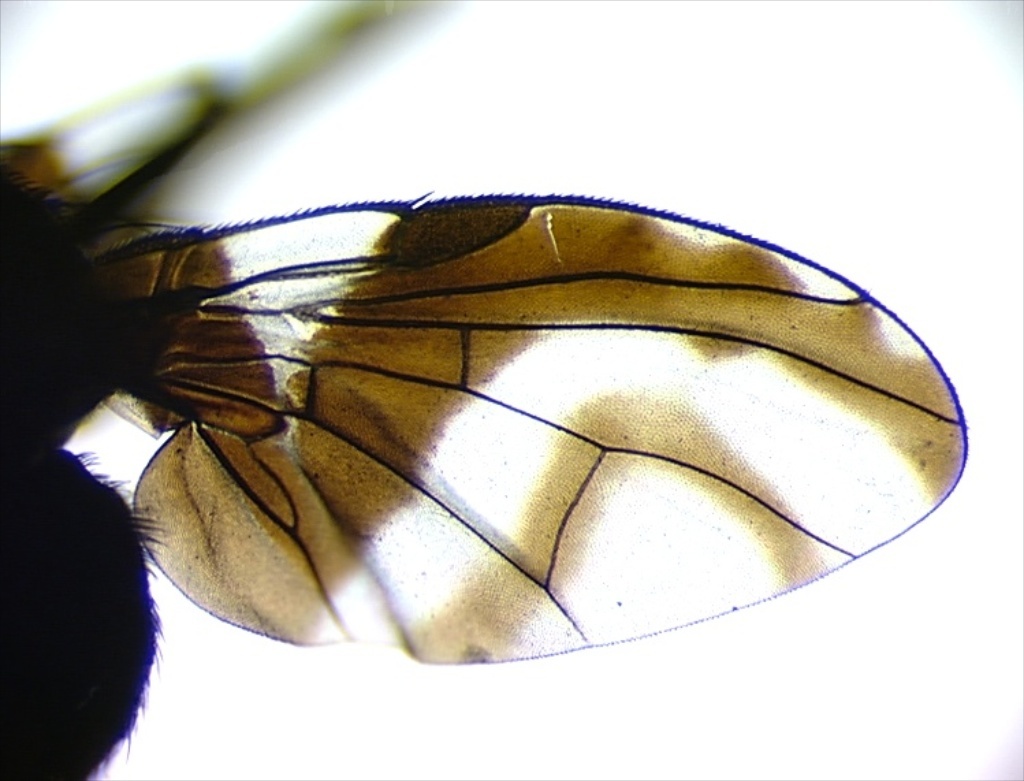

Supplement: Supplementary material 5 — Key to Carpophthoromyia [file zookeys-428-097-s005.zip › SF5_ZooKeys_key to Carpophthoromyia/key/SF5_ZooKeys_key to Carpophthoromyia/Media/Images/char10_indentation_368 wing dorsal (automontage (c) RMCA).jpg]

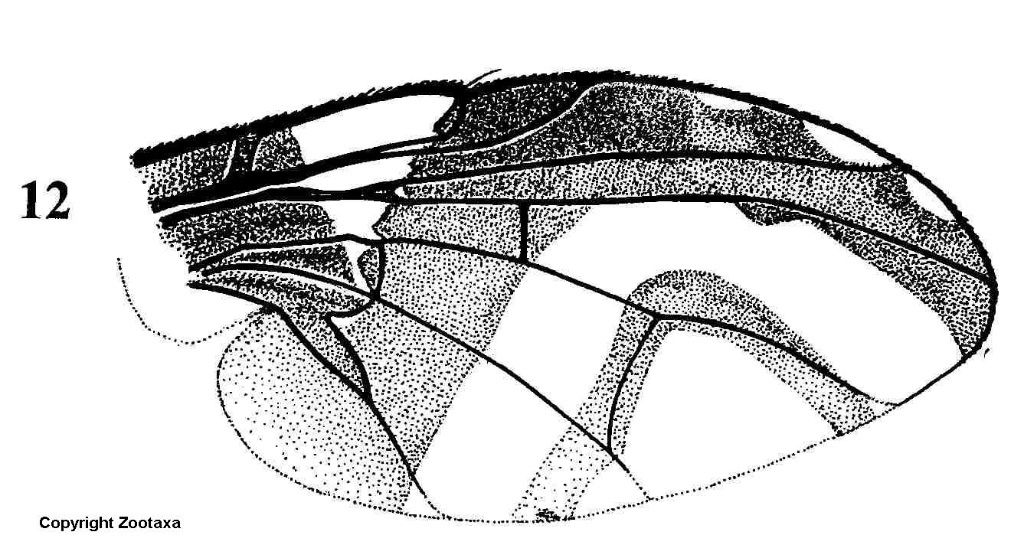

Supplement: Supplementary material 5 — Key to Carpophthoromyia [file zookeys-428-097-s005.zip › SF5_ZooKeys_key to Carpophthoromyia/key/SF5_ZooKeys_key to Carpophthoromyia/Media/Images/char10_indentation_368 wing dorsal (drawing (c) Zootaxa).jpg]

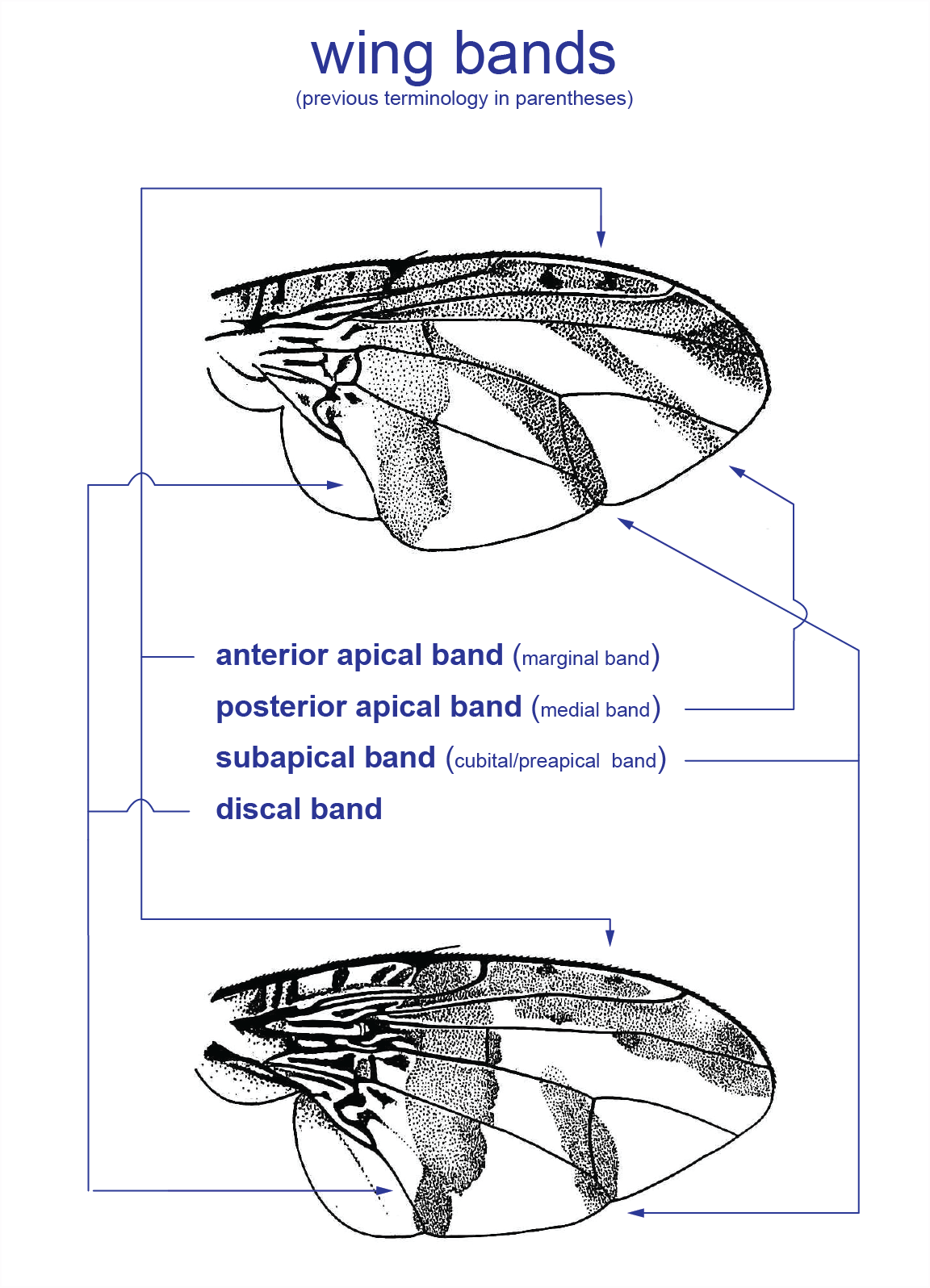

Supplement: Supplementary material 5 — Key to Carpophthoromyia [file zookeys-428-097-s005.zip › SF5_ZooKeys_key to Carpophthoromyia/key/SF5_ZooKeys_key to Carpophthoromyia/Media/Images/char10_wing_bands_Ceratitis.png]

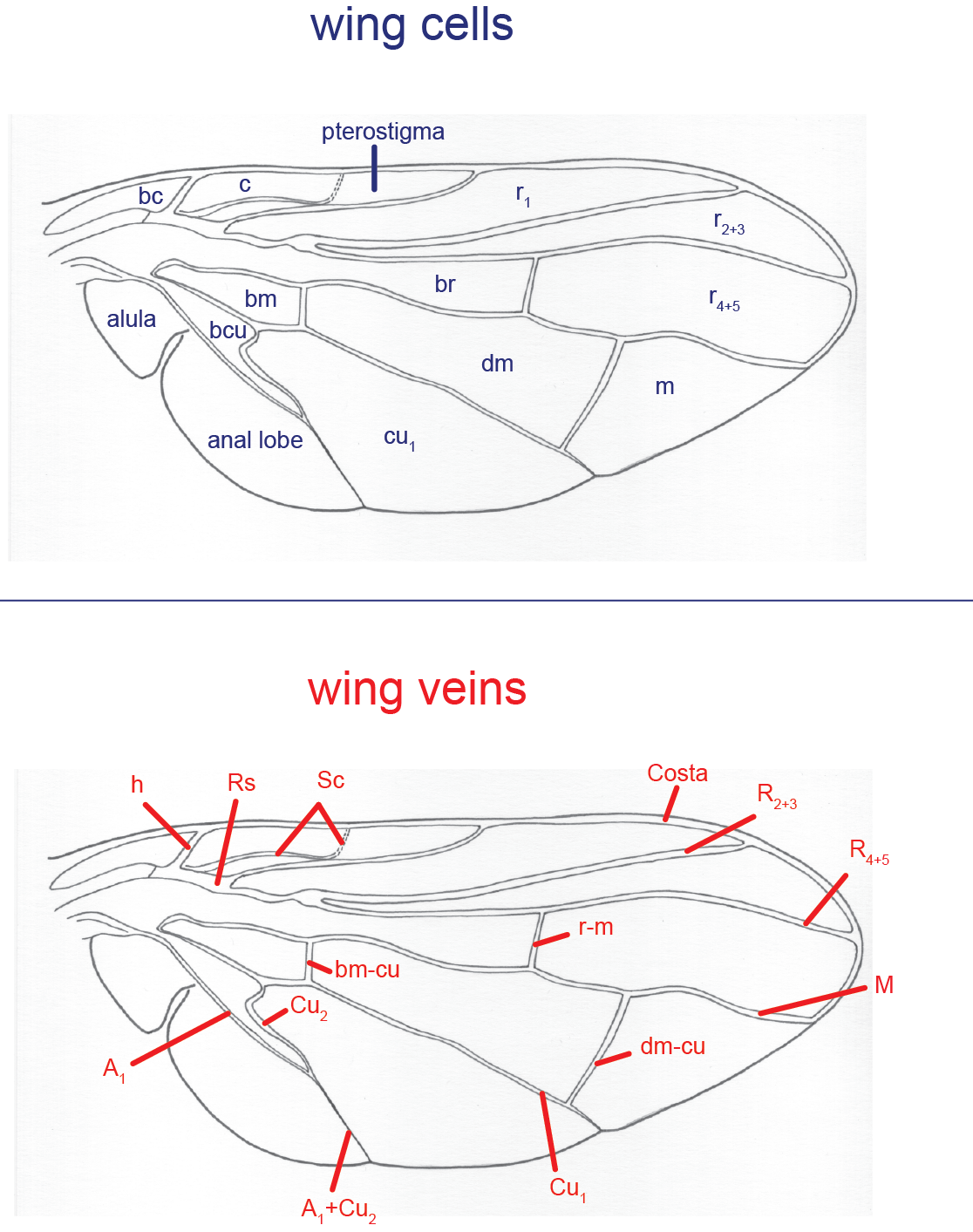

Supplement: Supplementary material 5 — Key to Carpophthoromyia [file zookeys-428-097-s005.zip › SF5_ZooKeys_key to Carpophthoromyia/key/SF5_ZooKeys_key to Carpophthoromyia/Media/Images/char11_wing_cells_veins.png]

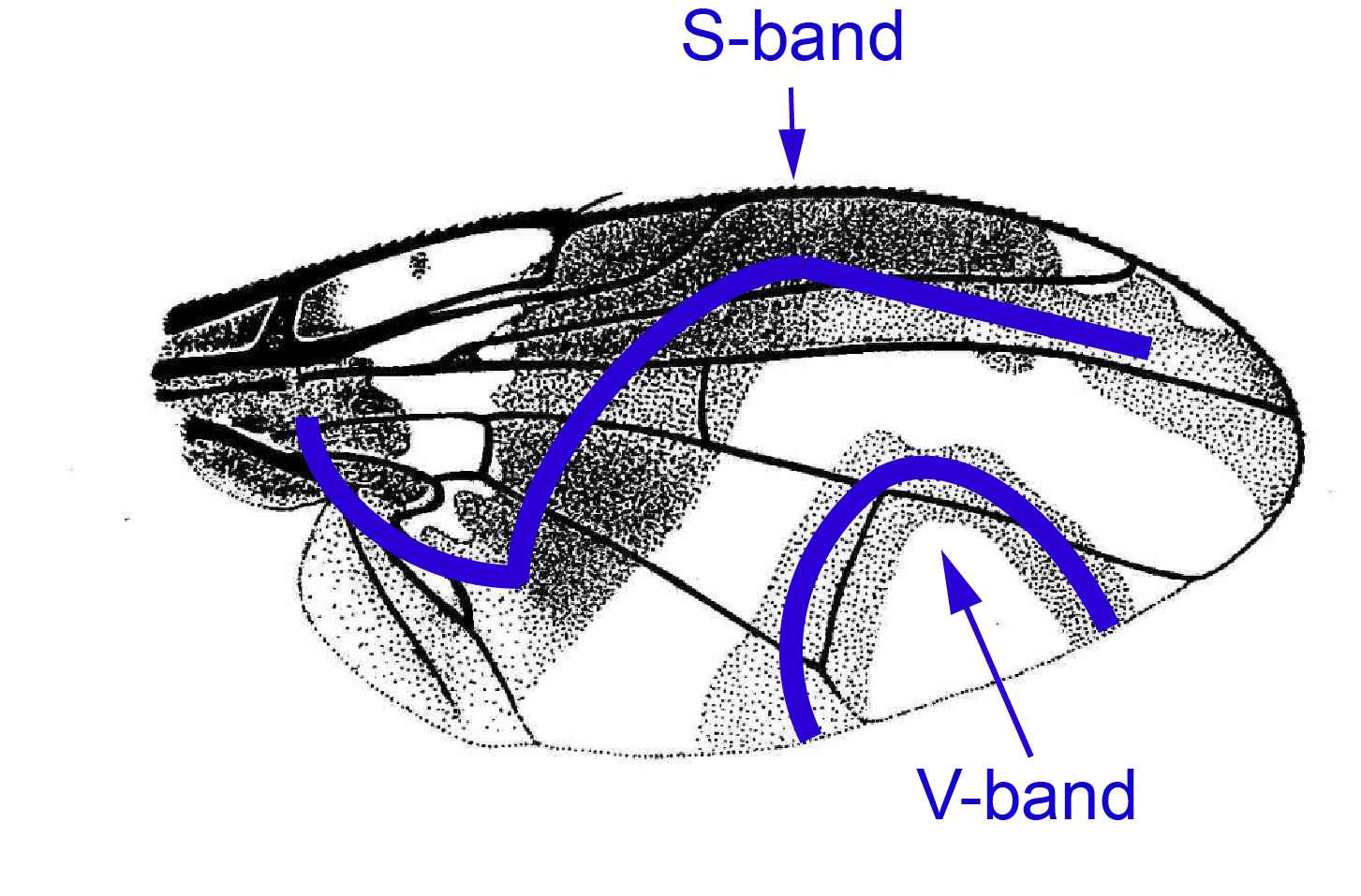

Supplement: Supplementary material 5 — Key to Carpophthoromyia [file zookeys-428-097-s005.zip › SF5_ZooKeys_key to Carpophthoromyia/key/SF5_ZooKeys_key to Carpophthoromyia/Media/Images/char12_Carpo_wing_bands_364 wing dorsal (drawing (c) Zootaxa).png]

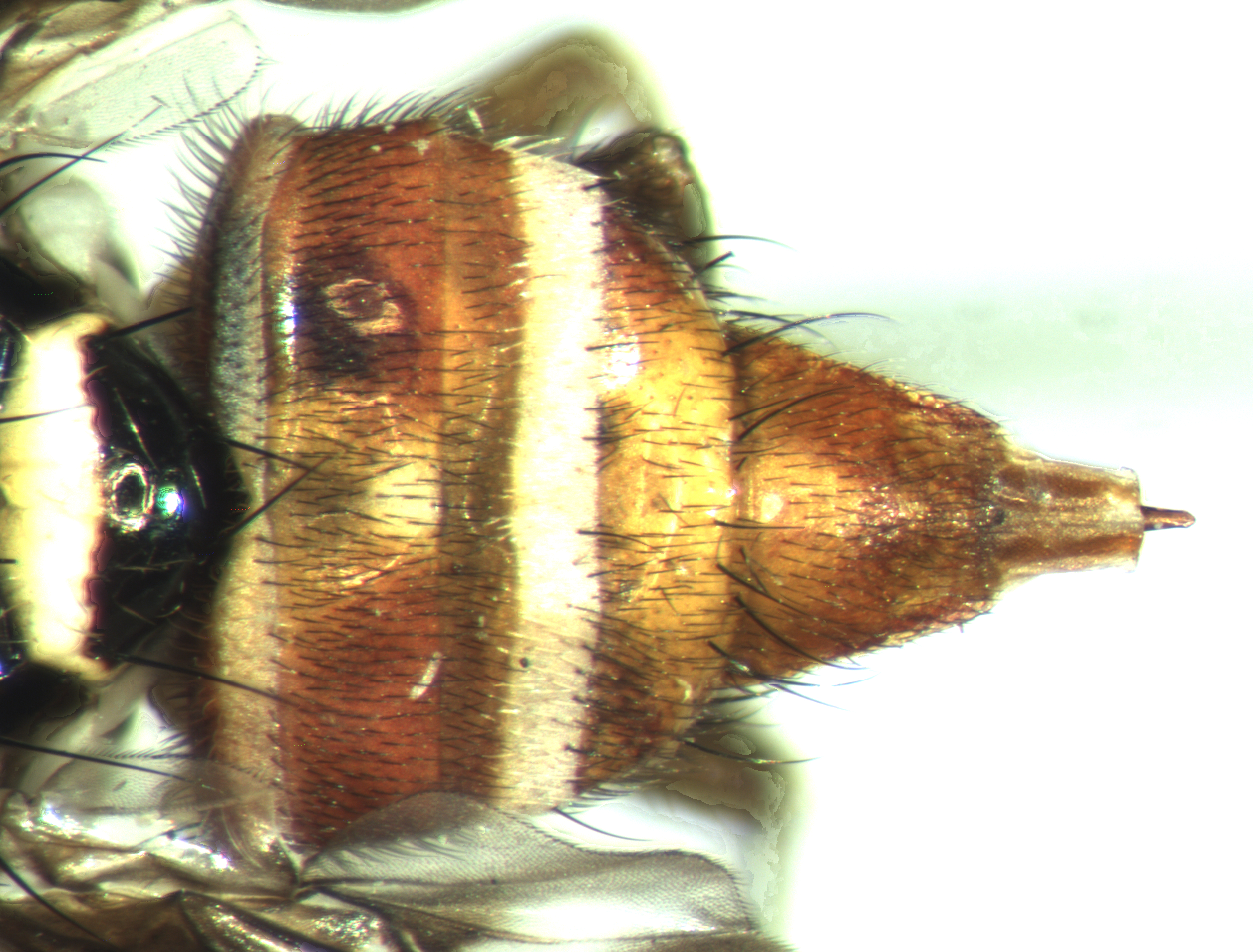

Supplement: Supplementary material 8 — Key to Neoceratitis [file zookeys-428-097-s008.zip › SF8_ZooKeys_key to Neoceratitis/key/SF8_key to Neoceratitis/Media/Images/158 abdomen female dorsal (automontage (c) RMCA).jpg]

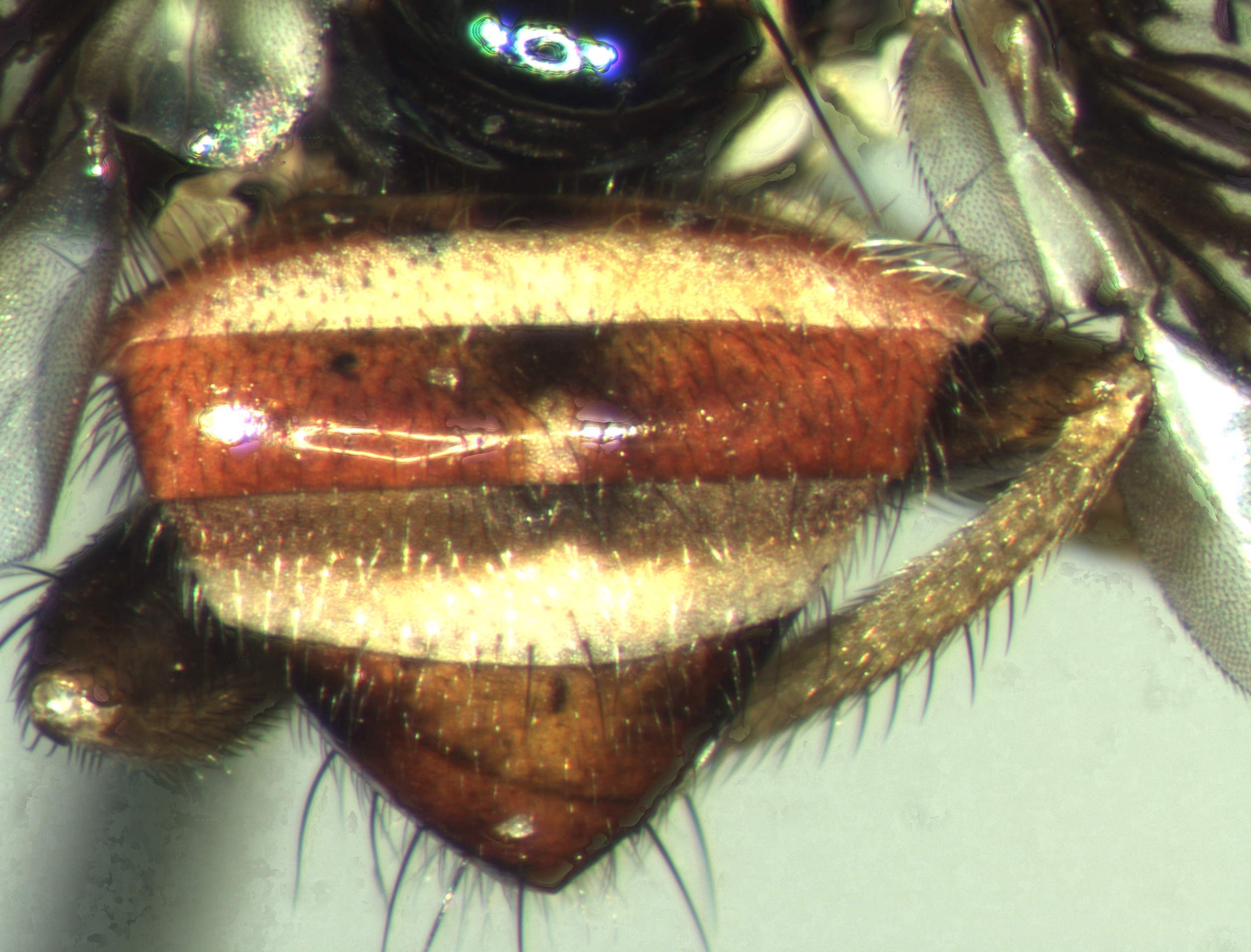

Supplement: Supplementary material 8 — Key to Neoceratitis [file zookeys-428-097-s008.zip › SF8_ZooKeys_key to Neoceratitis/key/SF8_key to Neoceratitis/Media/Images/158 abdomen male dorsal (automontage (c) RMCA).jpg]

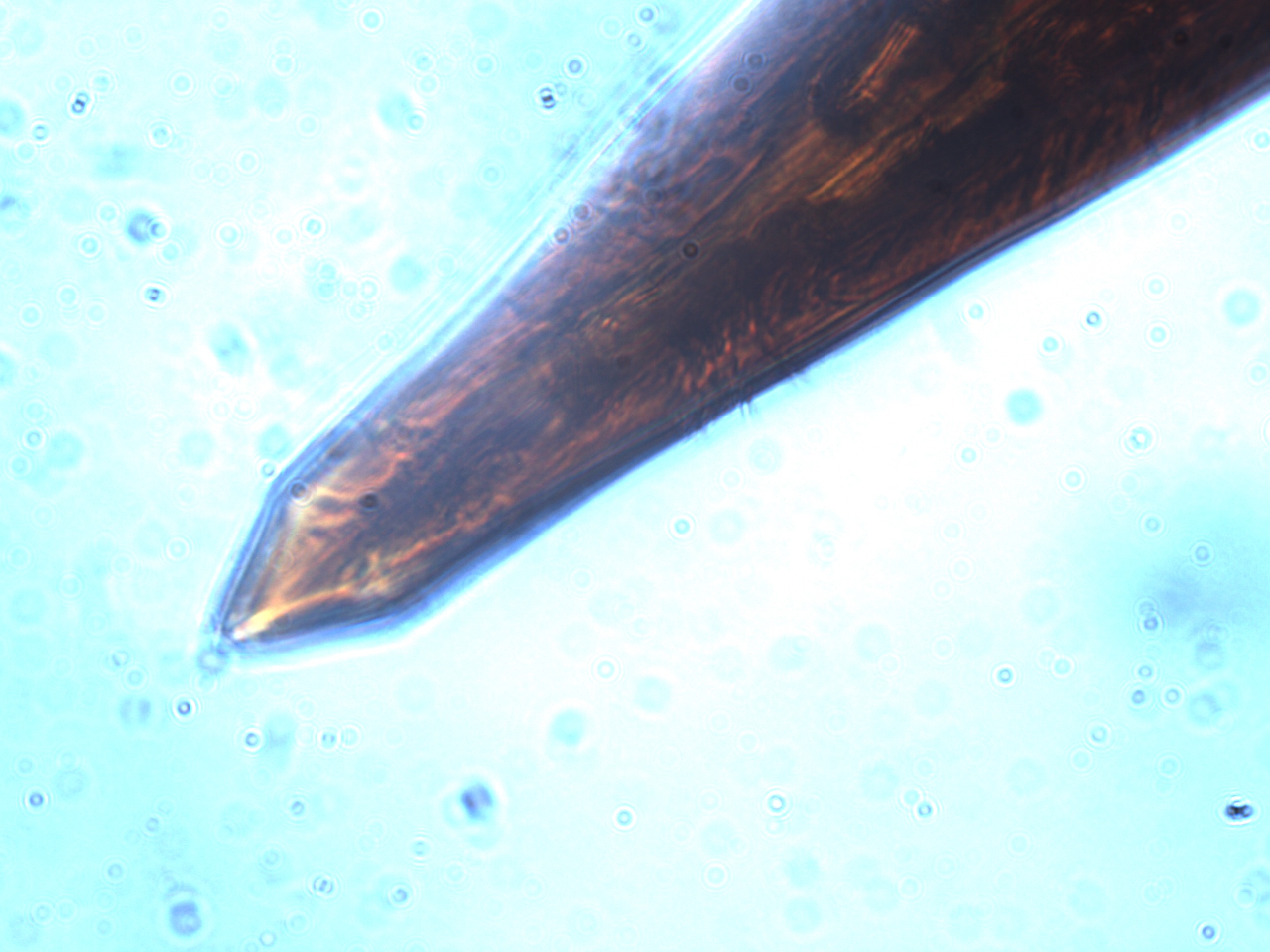

Supplement: Supplementary material 8 — Key to Neoceratitis [file zookeys-428-097-s008.zip › SF8_ZooKeys_key to Neoceratitis/key/SF8_key to Neoceratitis/Media/Images/158 aculeus tip (automontage (c) RMCA).jpg]

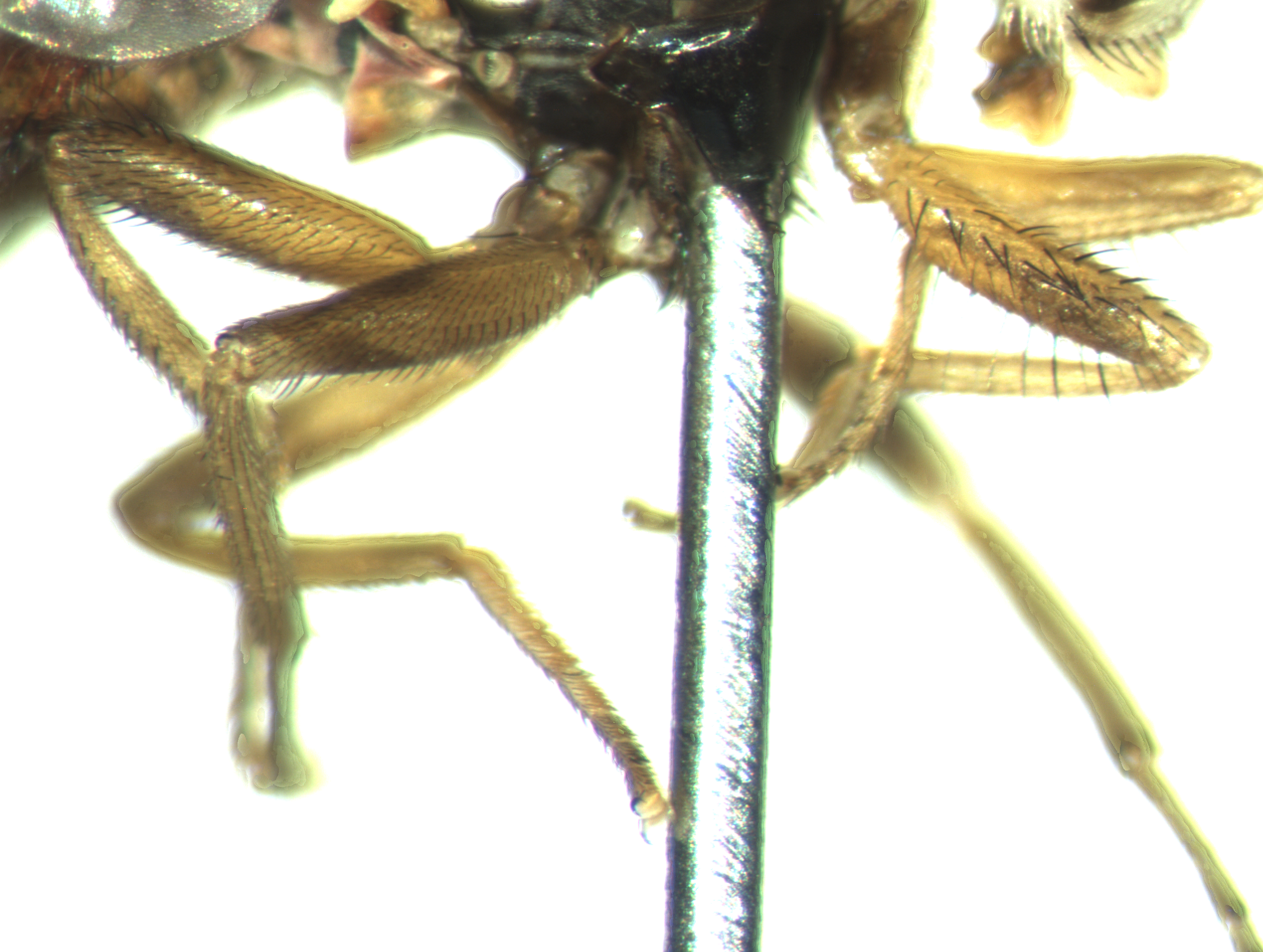

Supplement: Supplementary material 8 — Key to Neoceratitis [file zookeys-428-097-s008.zip › SF8_ZooKeys_key to Neoceratitis/key/SF8_key to Neoceratitis/Media/Images/158 legs female lateral (automontage (c) RMCA).jpg]

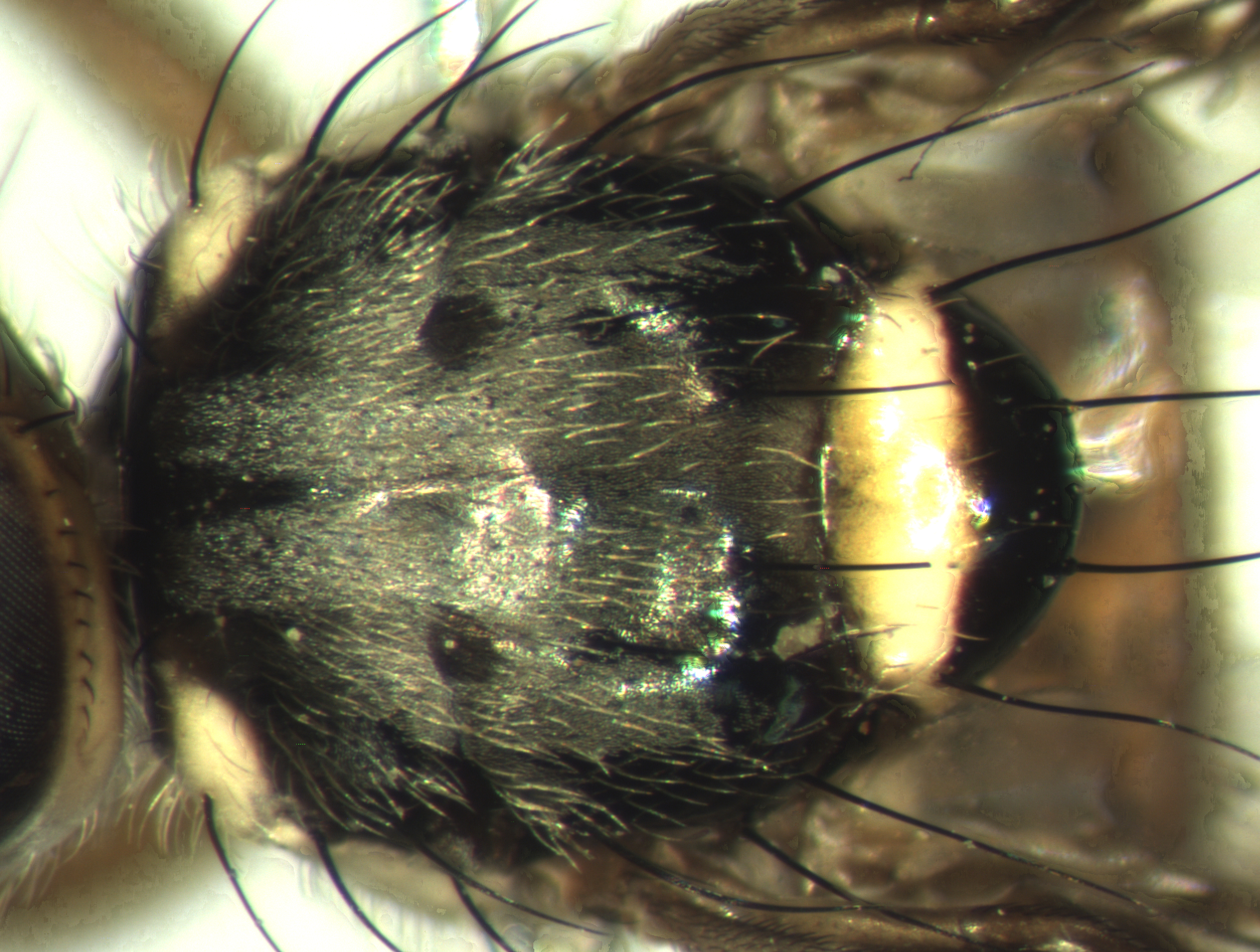

Supplement: Supplementary material 8 — Key to Neoceratitis [file zookeys-428-097-s008.zip › SF8_ZooKeys_key to Neoceratitis/key/SF8_key to Neoceratitis/Media/Images/158 mesonotum (automontage (c) RMCA).jpg]

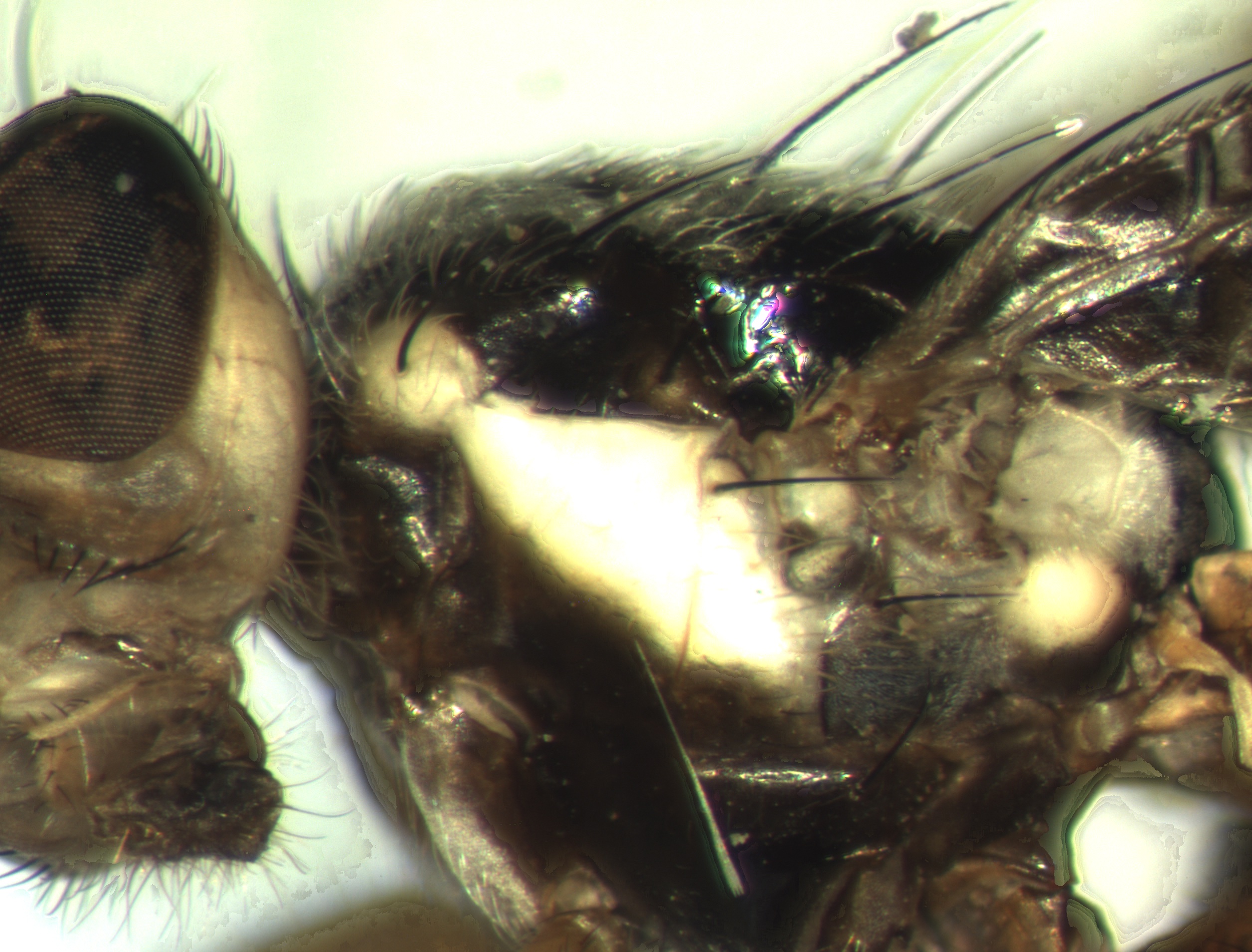

Supplement: Supplementary material 8 — Key to Neoceratitis [file zookeys-428-097-s008.zip › SF8_ZooKeys_key to Neoceratitis/key/SF8_key to Neoceratitis/Media/Images/158 thorax lateral (automontage (c) RMCA).jpg]

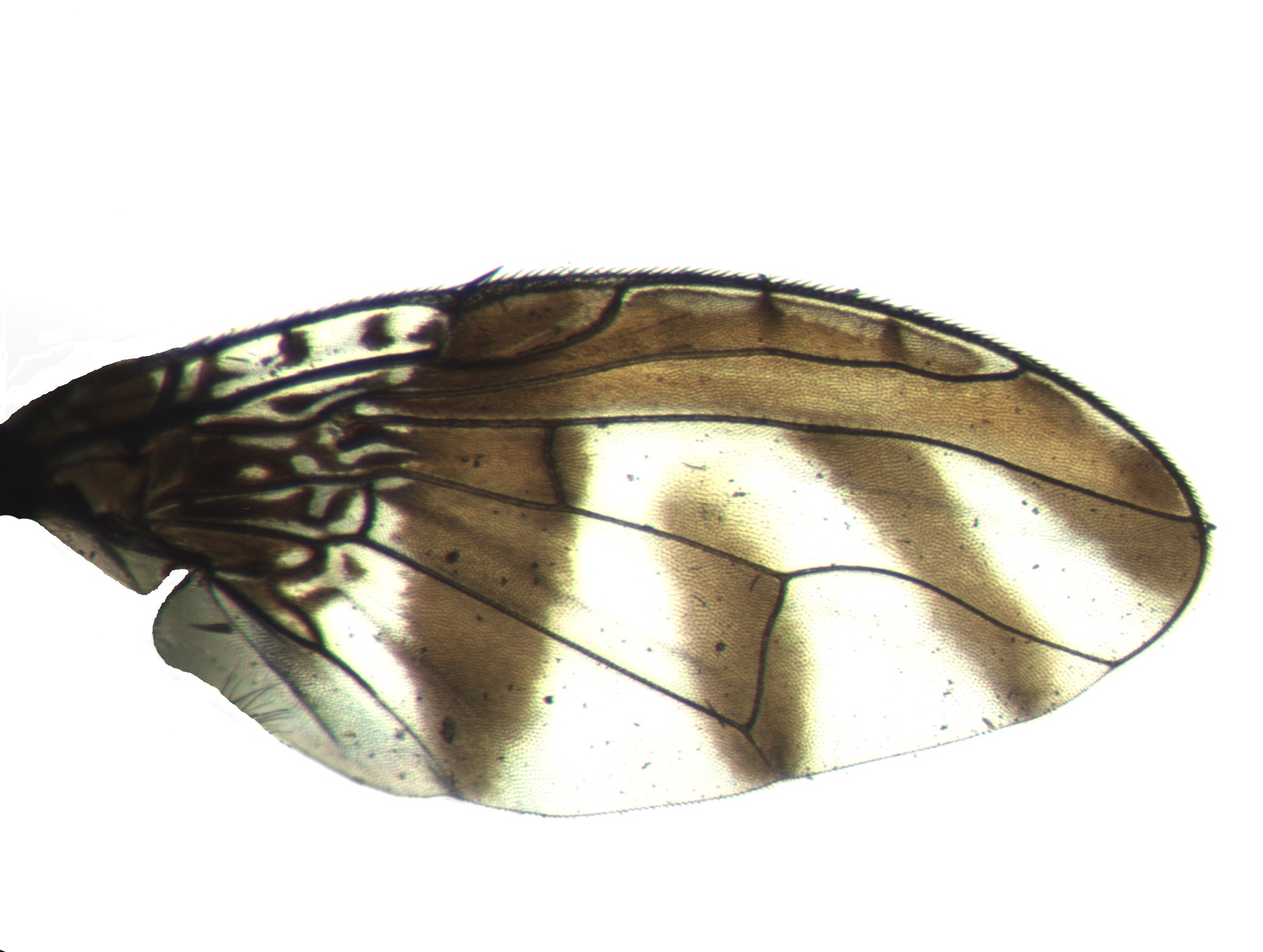

Supplement: Supplementary material 8 — Key to Neoceratitis [file zookeys-428-097-s008.zip › SF8_ZooKeys_key to Neoceratitis/key/SF8_key to Neoceratitis/Media/Images/158 wing (automontage (c) RMCA).jpg]

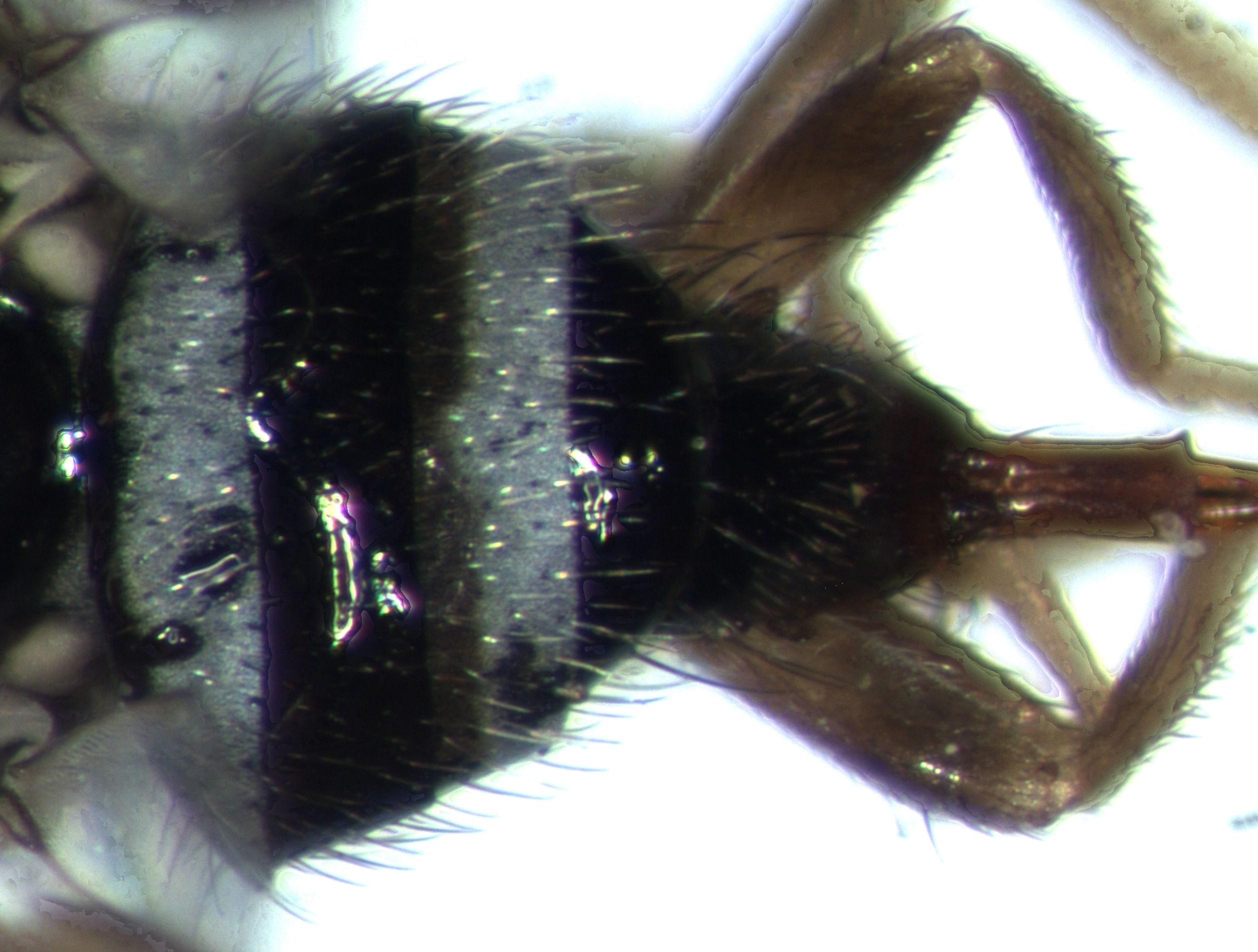

Supplement: Supplementary material 8 — Key to Neoceratitis [file zookeys-428-097-s008.zip › SF8_ZooKeys_key to Neoceratitis/key/SF8_key to Neoceratitis/Media/Images/429 abdomen female dorsal (automontage (c) RMCA).jpg]

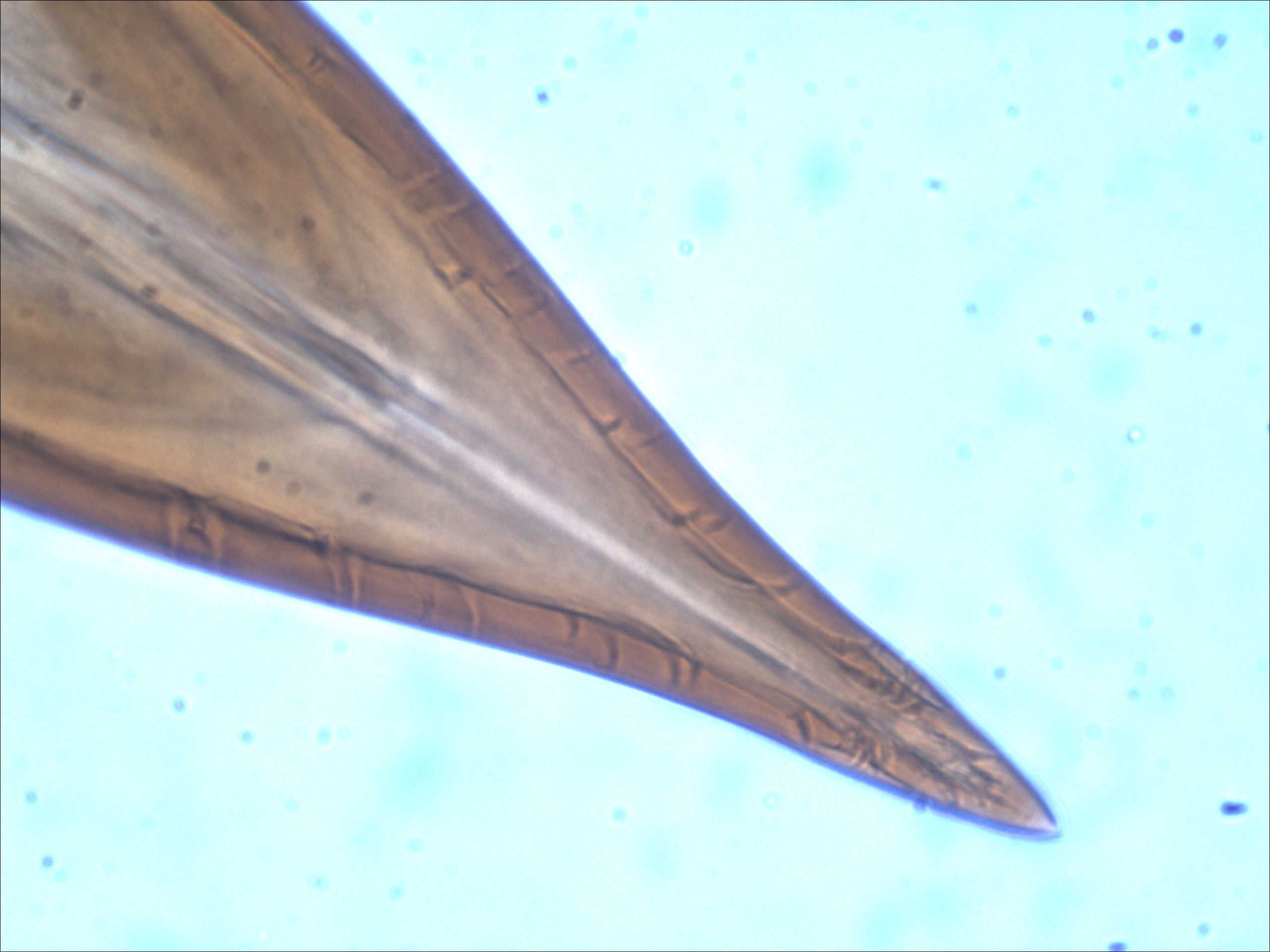

Supplement: Supplementary material 8 — Key to Neoceratitis [file zookeys-428-097-s008.zip › SF8_ZooKeys_key to Neoceratitis/key/SF8_key to Neoceratitis/Media/Images/429 aculeus tip (automontage (c) RMCA).jpg]
