# Supplementary material for: A set of multi-entry identification keys to African frugivorous flies (Diptera, Tephritidae)
Source: Zookeys. 2014 Jul 24;(428):97–108. doi: 10.3897/zookeys.428.7366 (PMC4143993; doi:10.3897/zookeys.428.7366)
Supplement: Supplementary material 8 — Key to Neoceratitis [file zookeys-428-097-s008.zip › SF8_ZooKeys_key to Neoceratitis/key/SF8_key to Neoceratitis/Media/Html/desc_Neoceratitis_lycii.html]

Natural Language Description


# A set of multi-entry identification keys to African frugivorous flies (Diptera, Tephritidae)

### Massimiliano Virgilio, Ian White, Marc De Meyer

## Neoceratitis lycii

(key to Neoceratitis) sex female or male. (key to Neoceratitis) thorax (key to Neoceratitis) 1. postpronotal lobe at least half dark, (key to Neoceratitis) 2. anepisternal setae black, (key to Neoceratitis) 3. scutellum (1) rounded, (key to Neoceratitis) 4. scutellum (2) median part white, (key to Neoceratitis) 5. scutellum (3) apical margin black. (key to Neoceratitis) wings (key to Neoceratitis) 6. posterior apical band isolated, touching anterior band only at base, (key to Neoceratitis) 7. subapical band touching discal and/or anterior bands, (key to Neoceratitis) 8. subapical band straight at base where touching discal-anterior apical bands. (key to Neoceratitis) abdomen (key to Neoceratitis) 10. (females) aculeus at most 6x as long as wide, (key to Neoceratitis) 9. (females) aculeus tip simply pointed.
