# Supplementary figures and images for: A set of multi-entry identification keys to African frugivorous flies (Diptera, Tephritidae) (part 3 of 6)
Source: Zookeys. 2014 Jul 24;(428):97–108. doi: 10.3897/zookeys.428.7366 (PMC4143993; doi:10.3897/zookeys.428.7366)

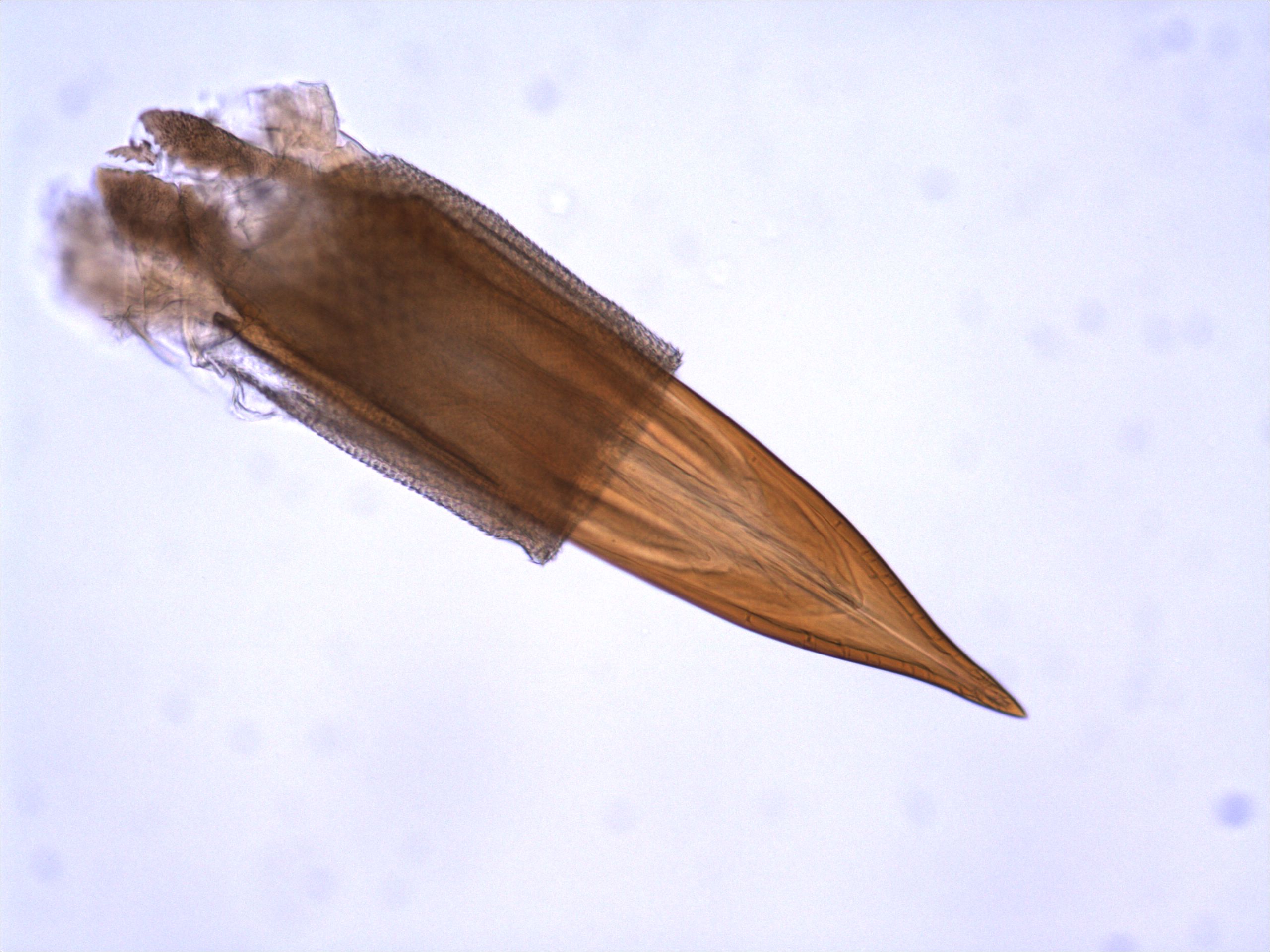

Supplement: Supplementary material 8 — Key to Neoceratitis [file zookeys-428-097-s008.zip › SF8_ZooKeys_key to Neoceratitis/key/SF8_key to Neoceratitis/Media/Images/429 aculeus(automontage (c) RMCA).jpg]

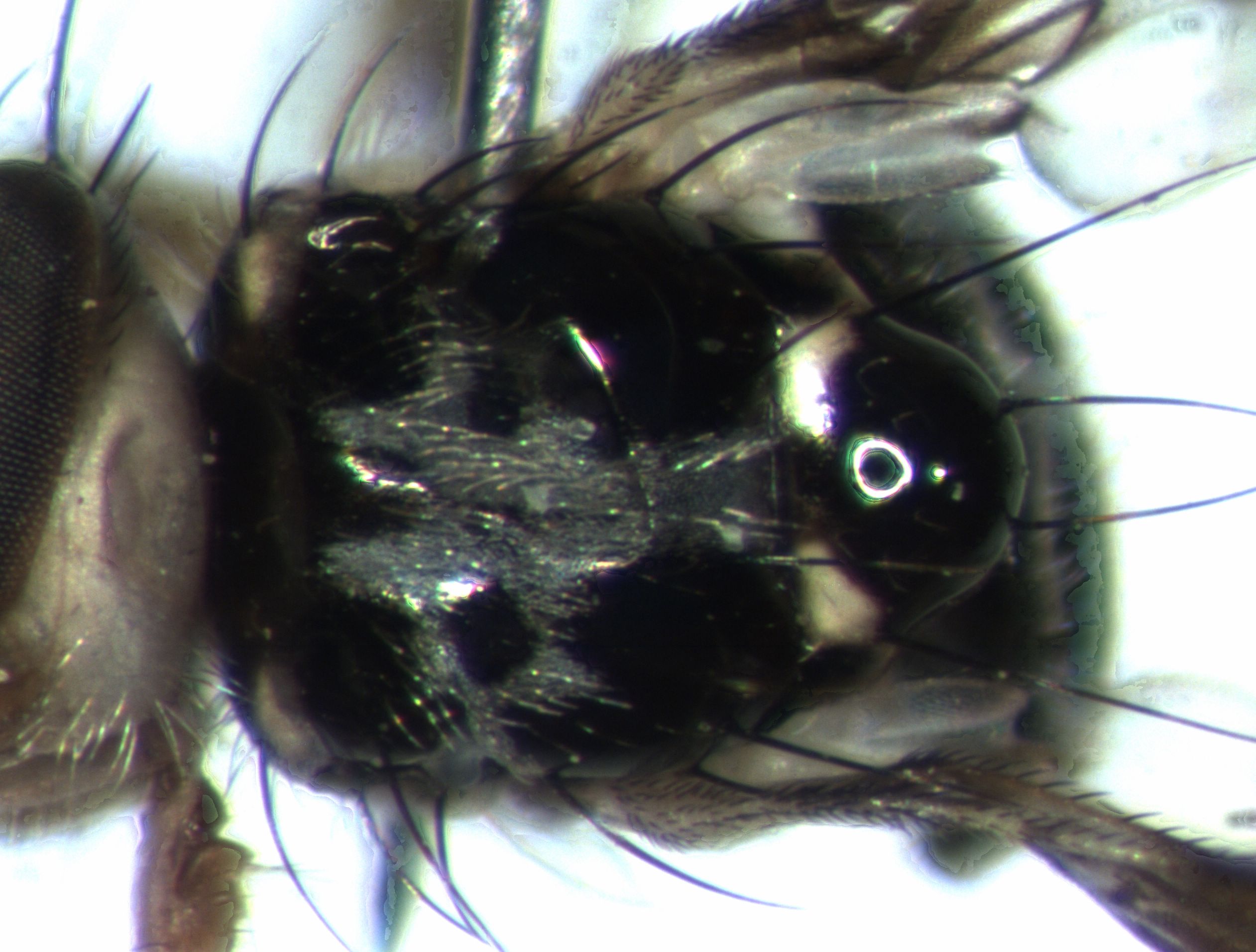

Supplement: Supplementary material 8 — Key to Neoceratitis [file zookeys-428-097-s008.zip › SF8_ZooKeys_key to Neoceratitis/key/SF8_key to Neoceratitis/Media/Images/429 mesonotum (automontage (c) RMCA).jpg]

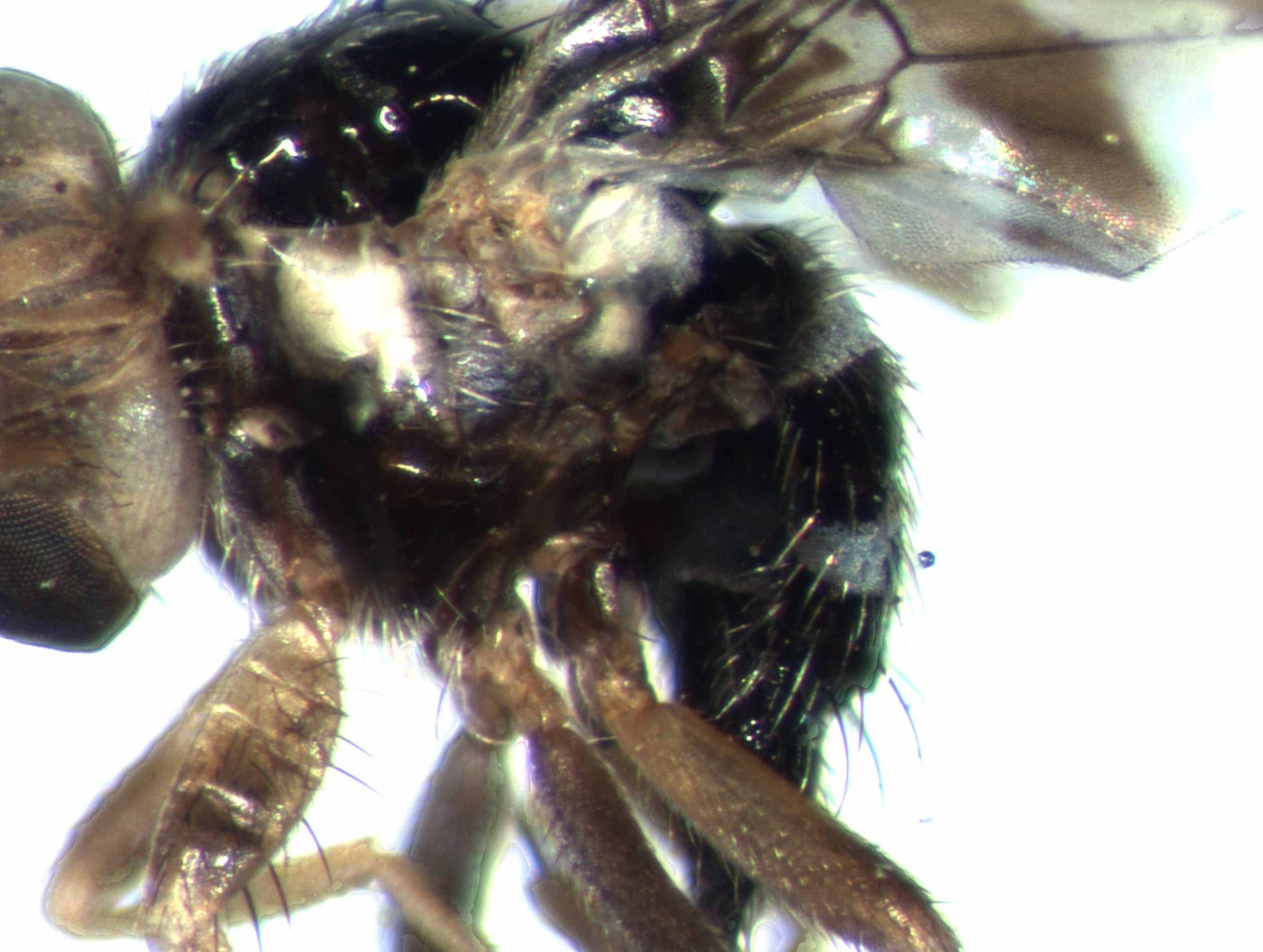

Supplement: Supplementary material 8 — Key to Neoceratitis [file zookeys-428-097-s008.zip › SF8_ZooKeys_key to Neoceratitis/key/SF8_key to Neoceratitis/Media/Images/429 thorax lateral (automontage (c) RMCA).jpg]

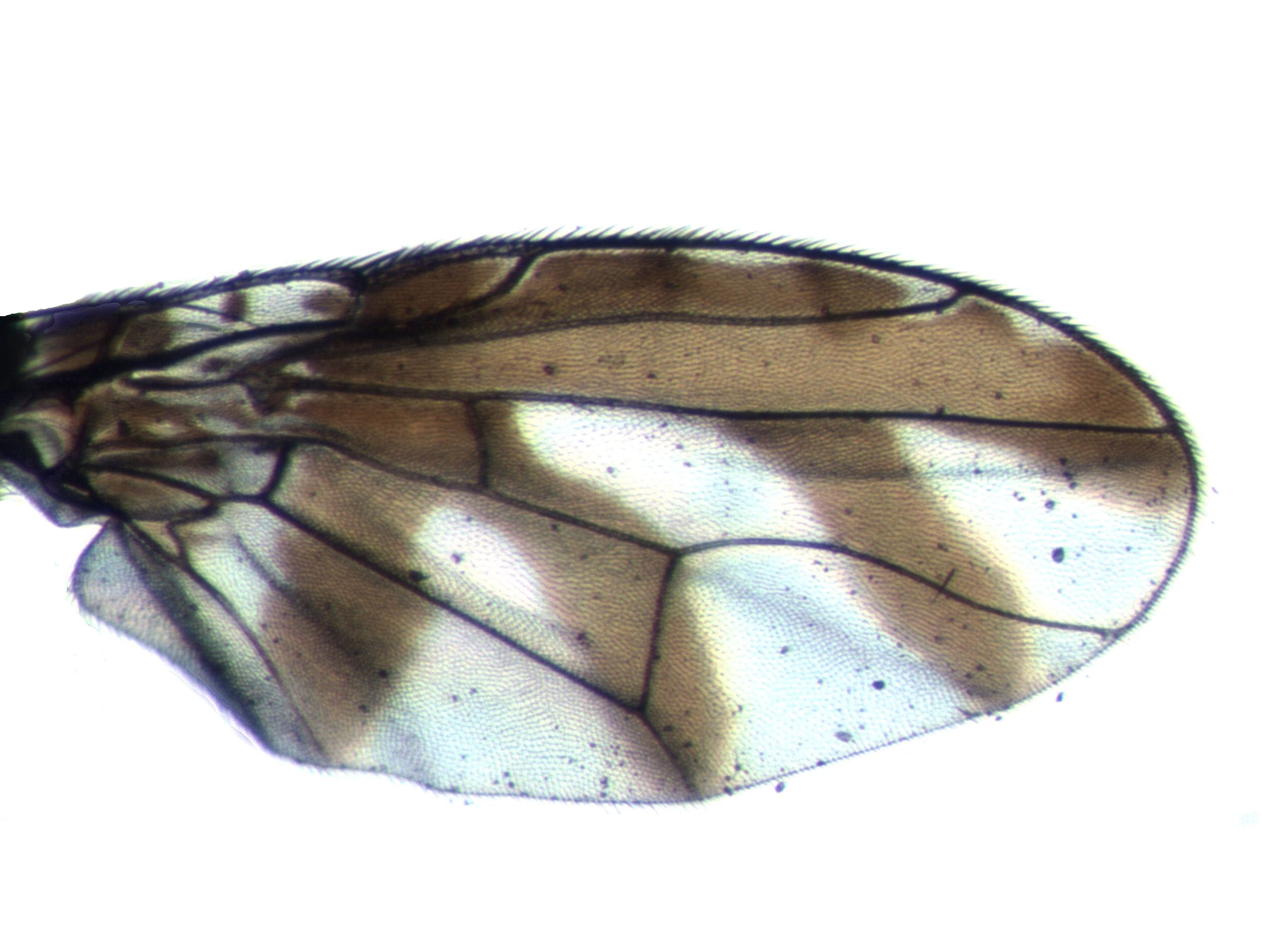

Supplement: Supplementary material 8 — Key to Neoceratitis [file zookeys-428-097-s008.zip › SF8_ZooKeys_key to Neoceratitis/key/SF8_key to Neoceratitis/Media/Images/429 wing (automontage (c) RMCA).jpg]

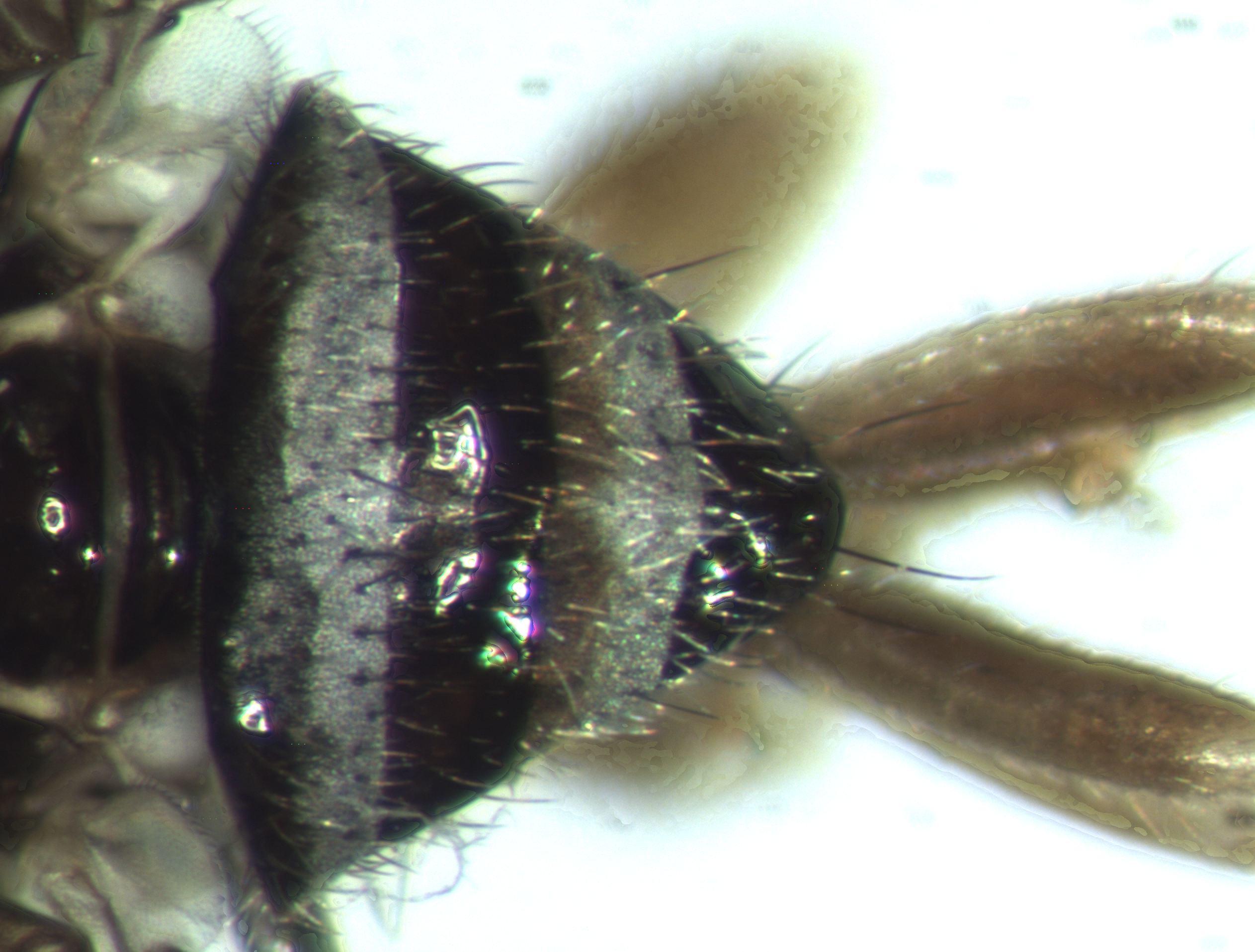

Supplement: Supplementary material 8 — Key to Neoceratitis [file zookeys-428-097-s008.zip › SF8_ZooKeys_key to Neoceratitis/key/SF8_key to Neoceratitis/Media/Images/430 abdomen male dorsal (automontage (c) RMCA).jpg]

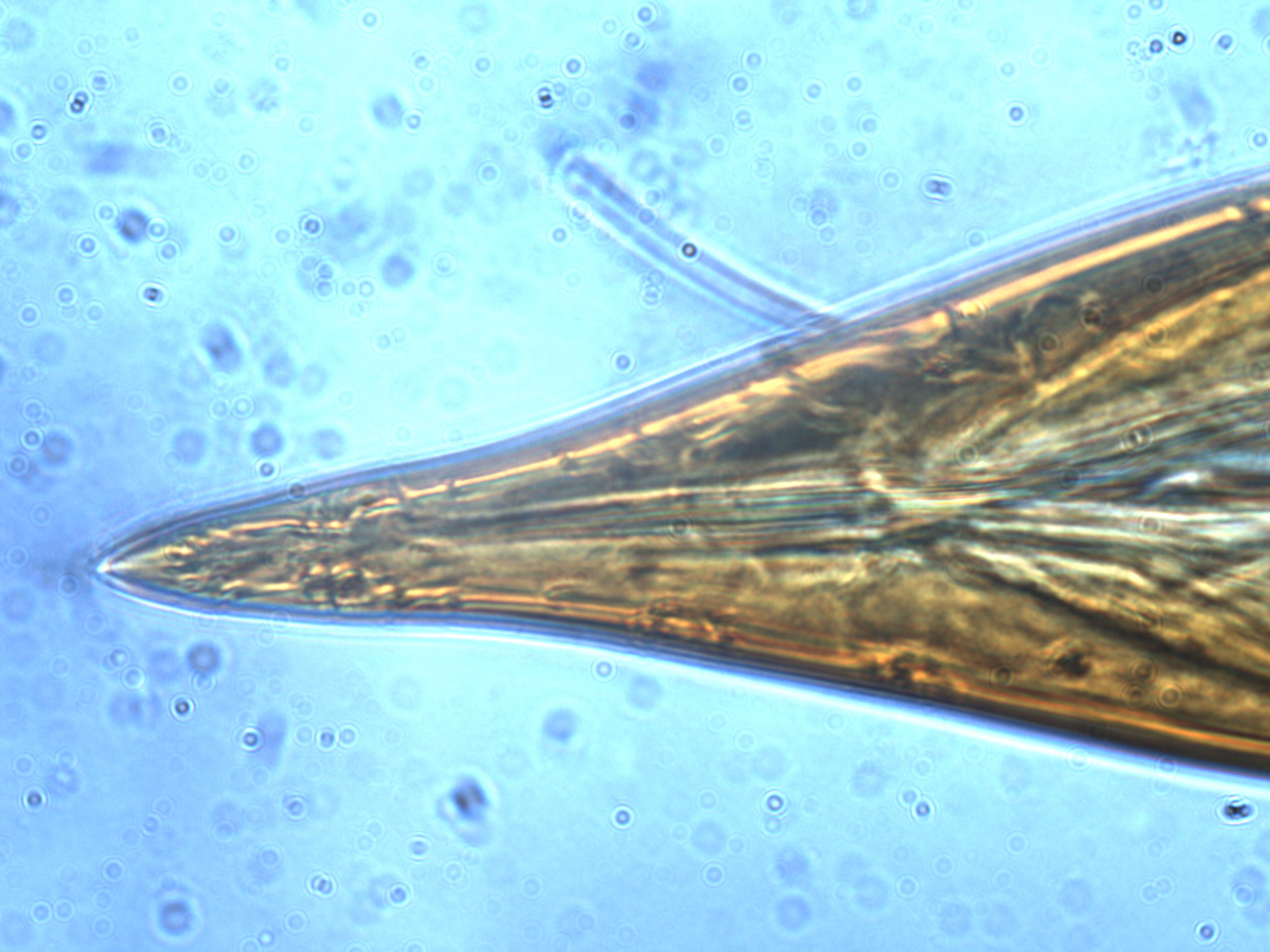

Supplement: Supplementary material 8 — Key to Neoceratitis [file zookeys-428-097-s008.zip › SF8_ZooKeys_key to Neoceratitis/key/SF8_key to Neoceratitis/Media/Images/430 aculeus tip (automontage (c) RMCA).jpg]

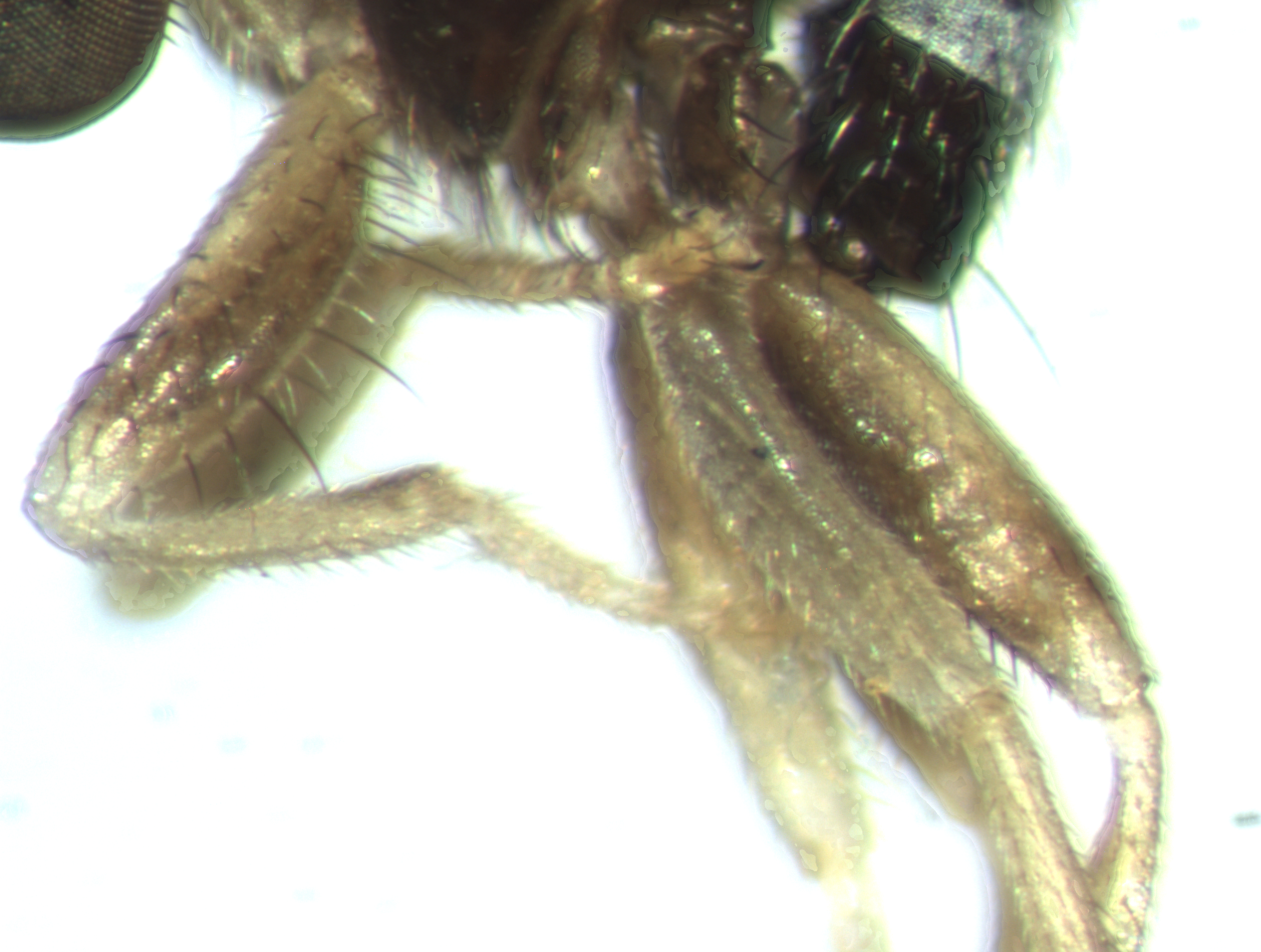

Supplement: Supplementary material 8 — Key to Neoceratitis [file zookeys-428-097-s008.zip › SF8_ZooKeys_key to Neoceratitis/key/SF8_key to Neoceratitis/Media/Images/430 legs male lateral (automontage (c) RMCA).jpg]

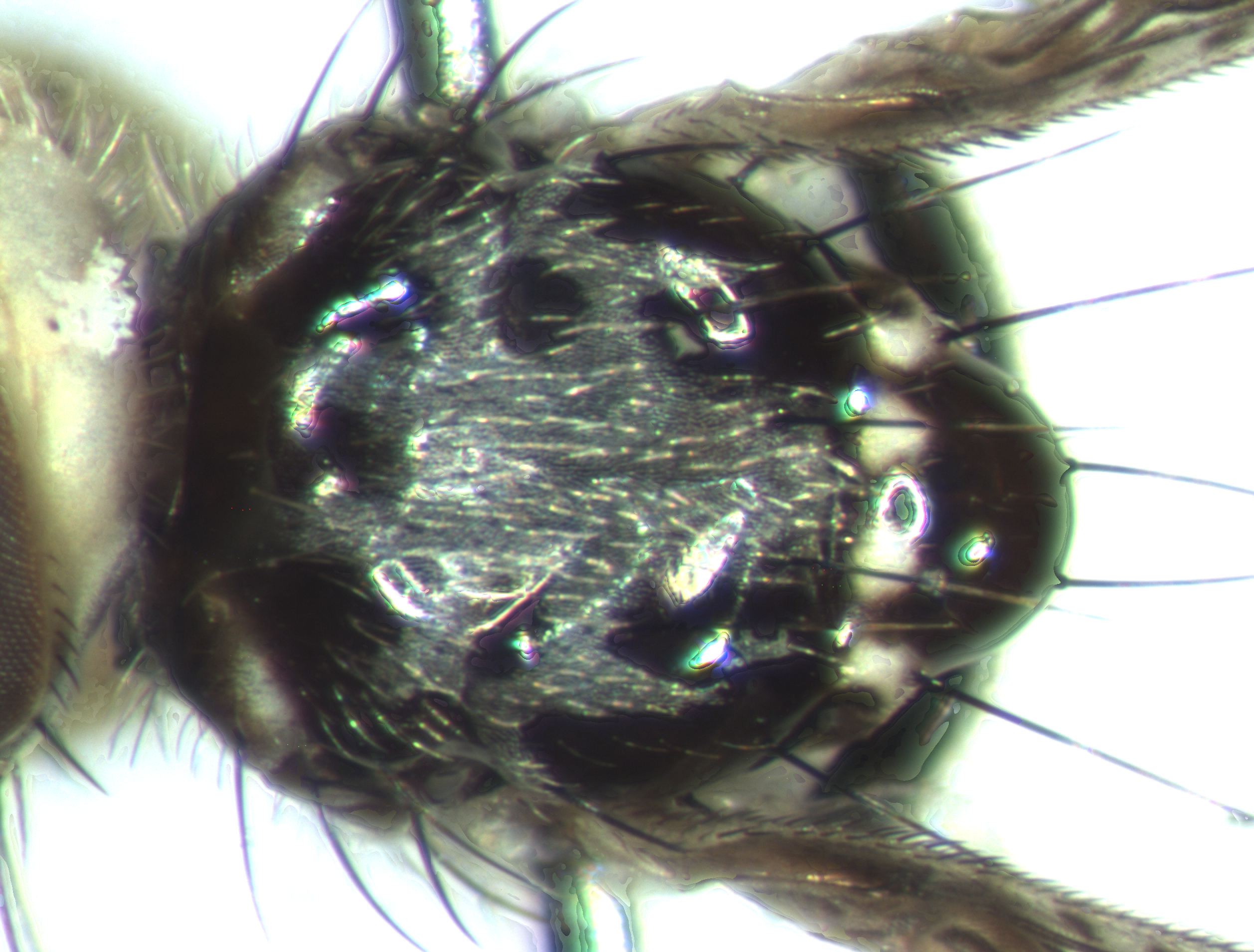

Supplement: Supplementary material 8 — Key to Neoceratitis [file zookeys-428-097-s008.zip › SF8_ZooKeys_key to Neoceratitis/key/SF8_key to Neoceratitis/Media/Images/430 mesonotum (automontage (c) RMCA).jpg]

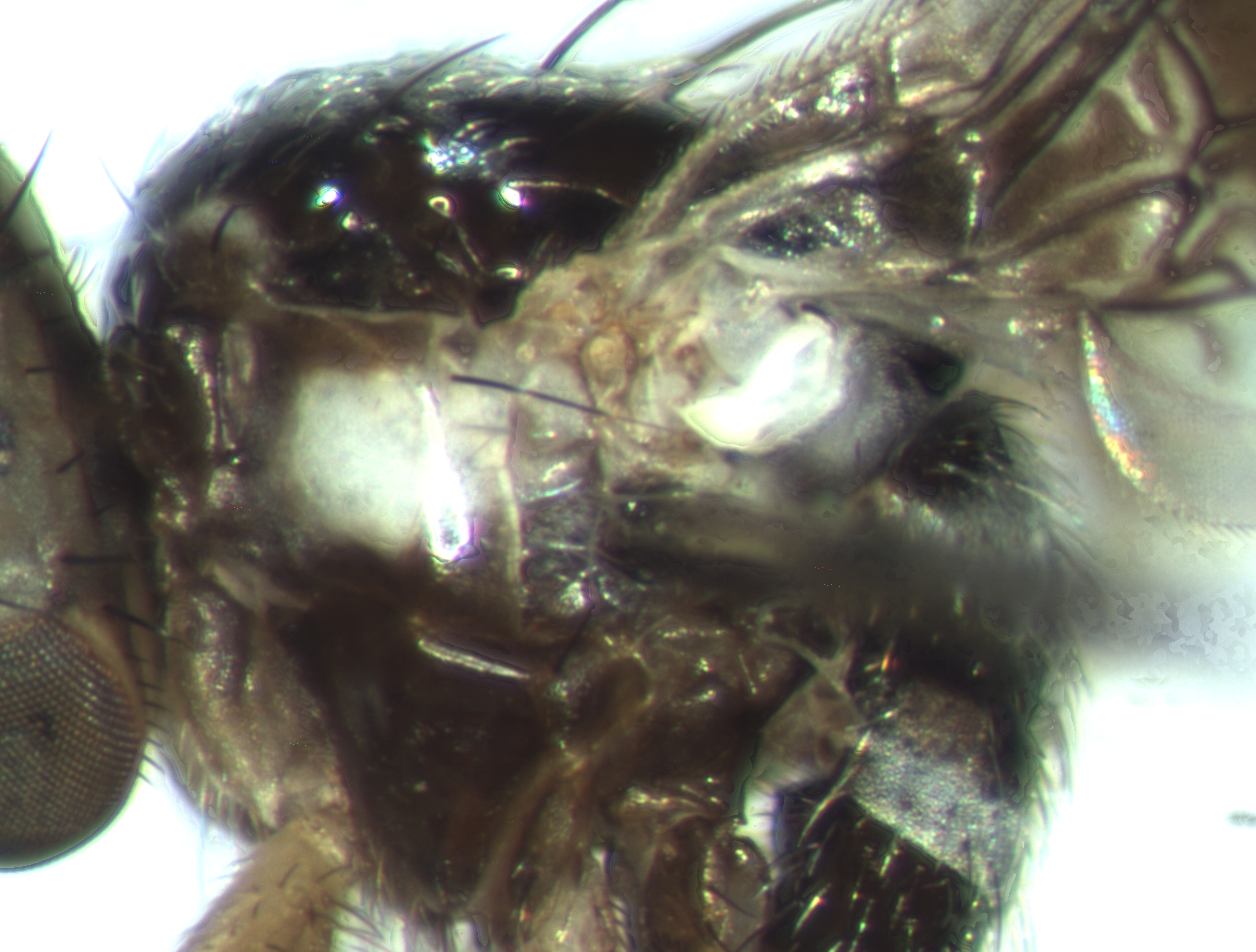

Supplement: Supplementary material 8 — Key to Neoceratitis [file zookeys-428-097-s008.zip › SF8_ZooKeys_key to Neoceratitis/key/SF8_key to Neoceratitis/Media/Images/430 thorax lateral (automontage (c) RMCA).jpg]

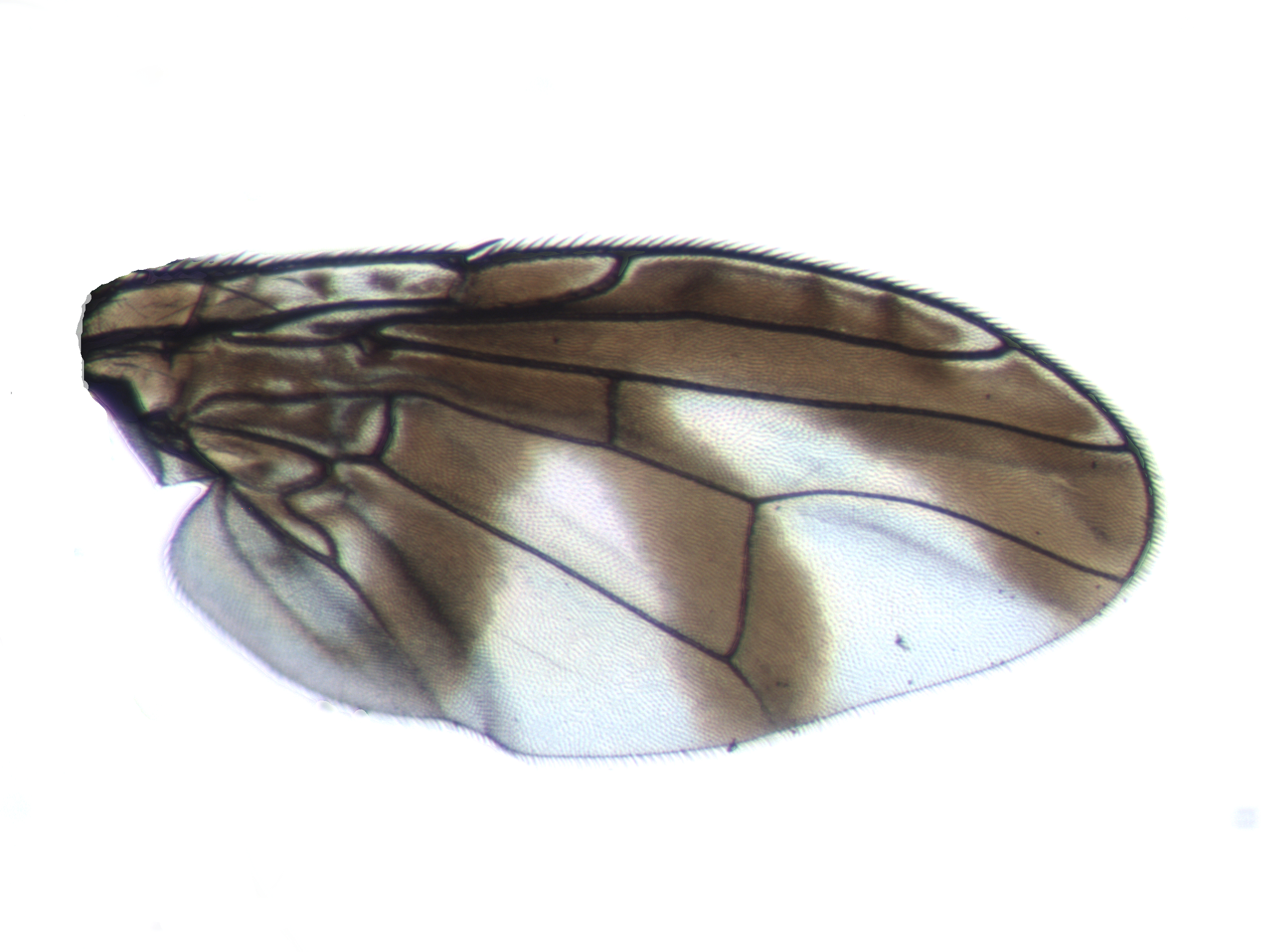

Supplement: Supplementary material 8 — Key to Neoceratitis [file zookeys-428-097-s008.zip › SF8_ZooKeys_key to Neoceratitis/key/SF8_key to Neoceratitis/Media/Images/430 wing (automontage (c) RMCA).jpg]

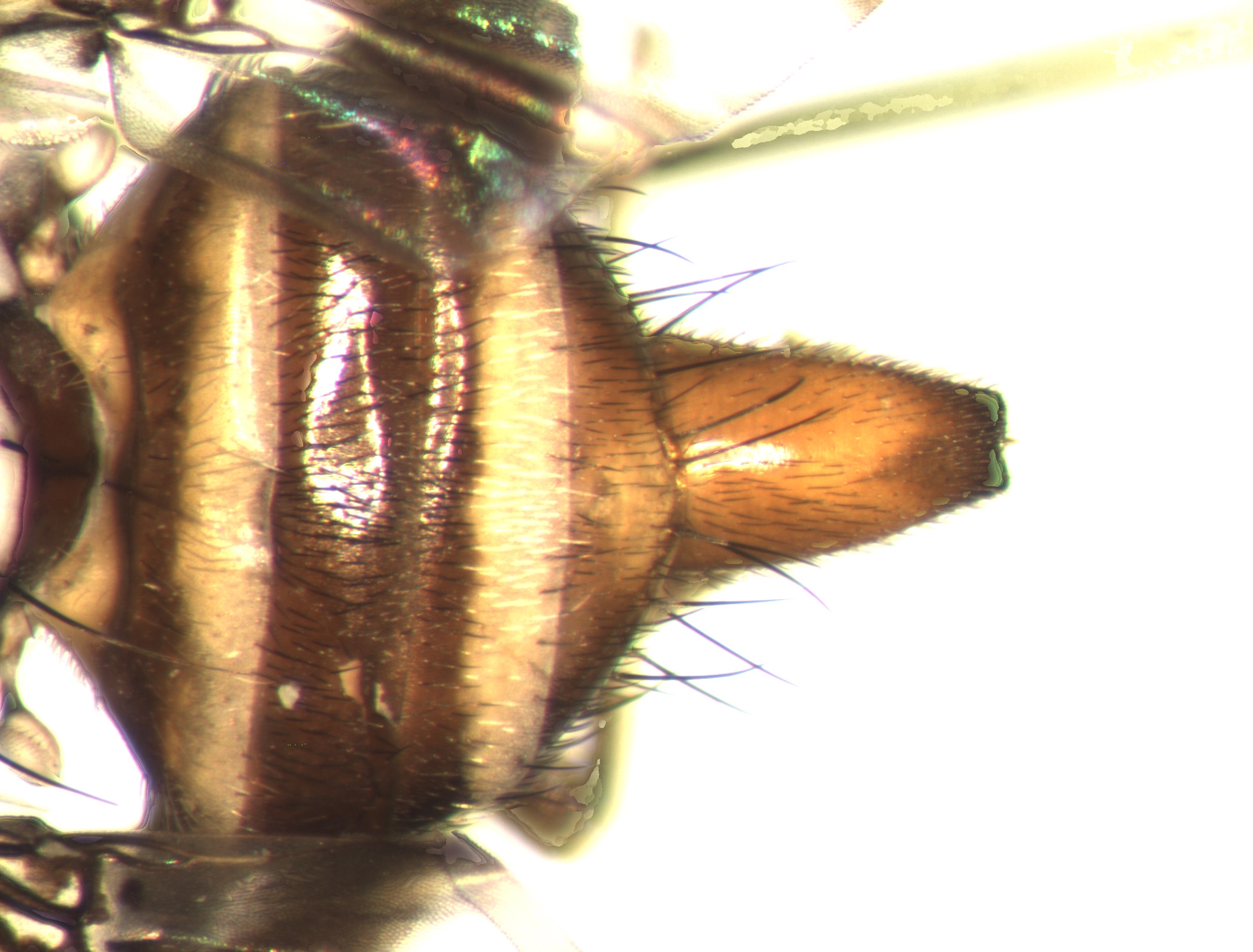

Supplement: Supplementary material 8 — Key to Neoceratitis [file zookeys-428-097-s008.zip › SF8_ZooKeys_key to Neoceratitis/key/SF8_key to Neoceratitis/Media/Images/431 abdomen female dorsal (automontage (c) RMCA).jpg]

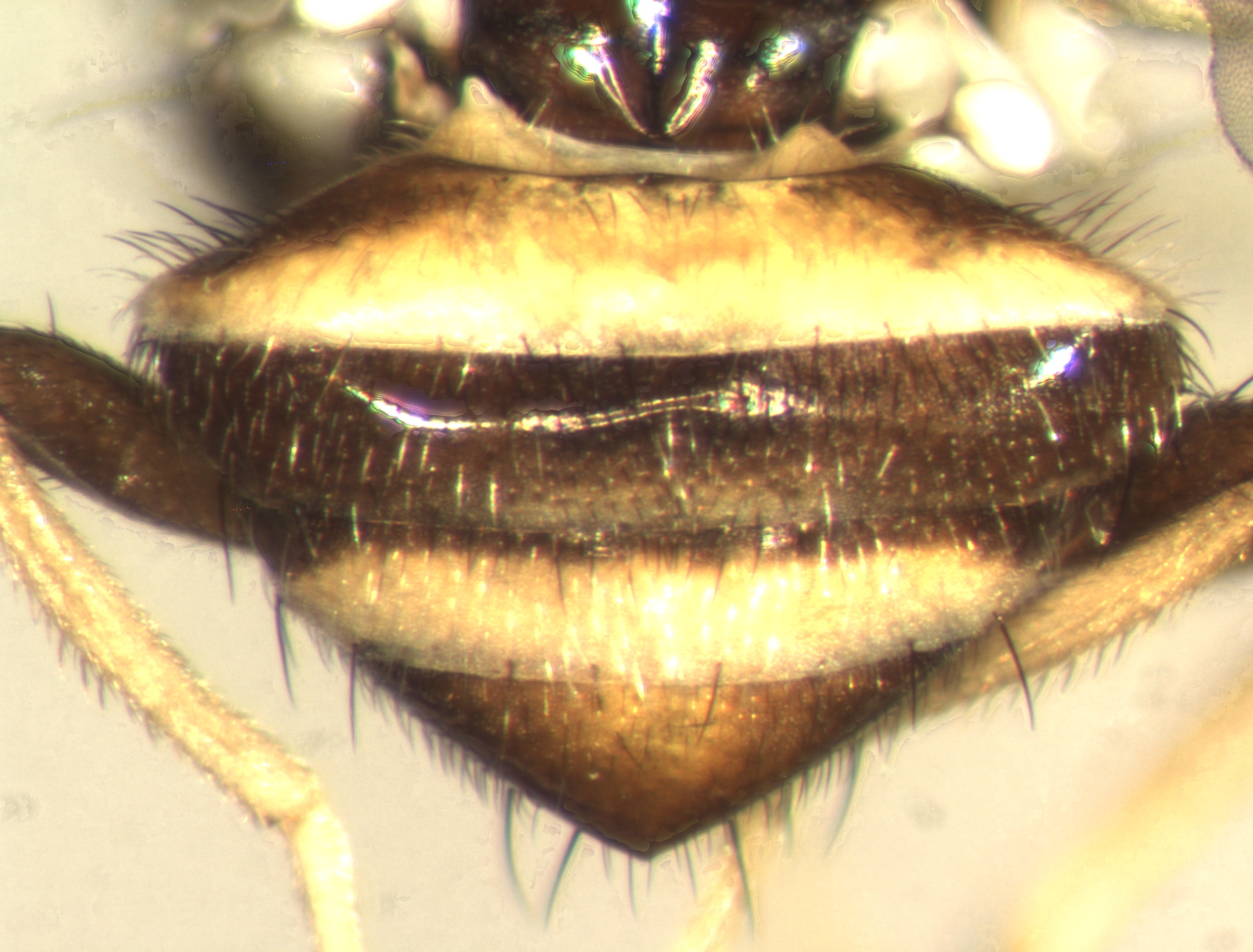

Supplement: Supplementary material 8 — Key to Neoceratitis [file zookeys-428-097-s008.zip › SF8_ZooKeys_key to Neoceratitis/key/SF8_key to Neoceratitis/Media/Images/431 abdomen male dorsal (automontage (c) RMCA).jpg]

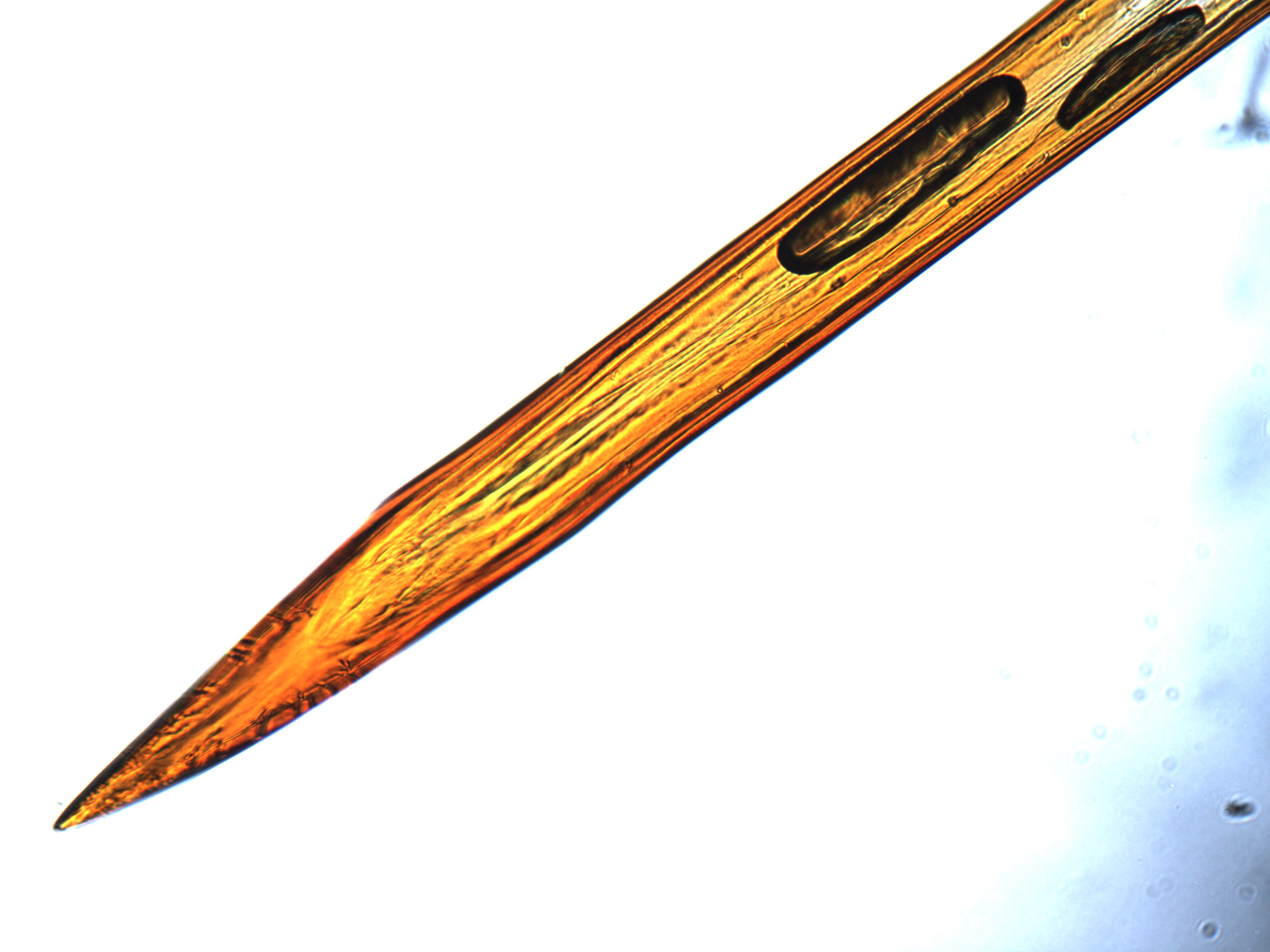

Supplement: Supplementary material 8 — Key to Neoceratitis [file zookeys-428-097-s008.zip › SF8_ZooKeys_key to Neoceratitis/key/SF8_key to Neoceratitis/Media/Images/431 aculeus(automontage (c) RMCA).jpg]

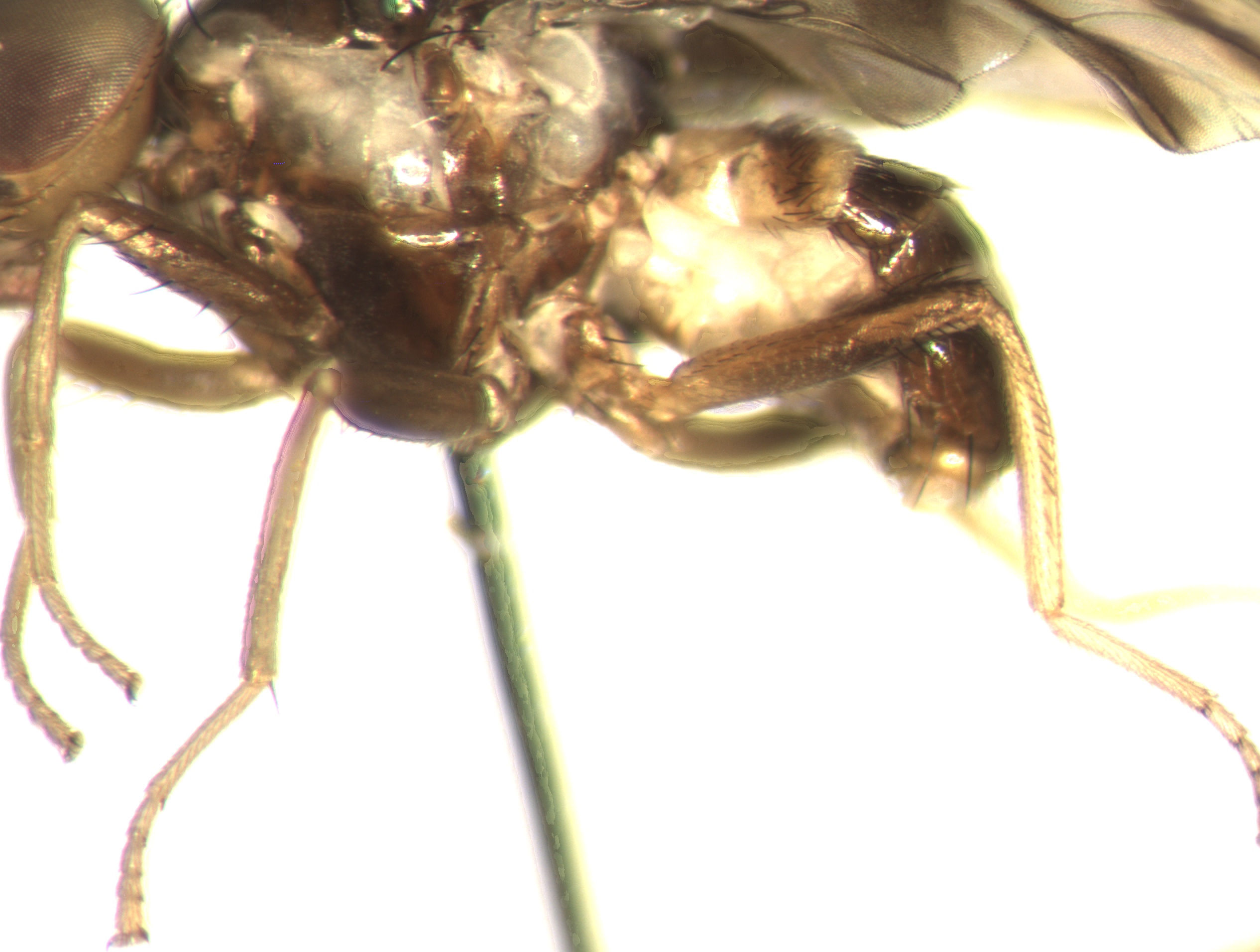

Supplement: Supplementary material 8 — Key to Neoceratitis [file zookeys-428-097-s008.zip › SF8_ZooKeys_key to Neoceratitis/key/SF8_key to Neoceratitis/Media/Images/431 legs male lateral (automontage (c) RMCA).jpg]

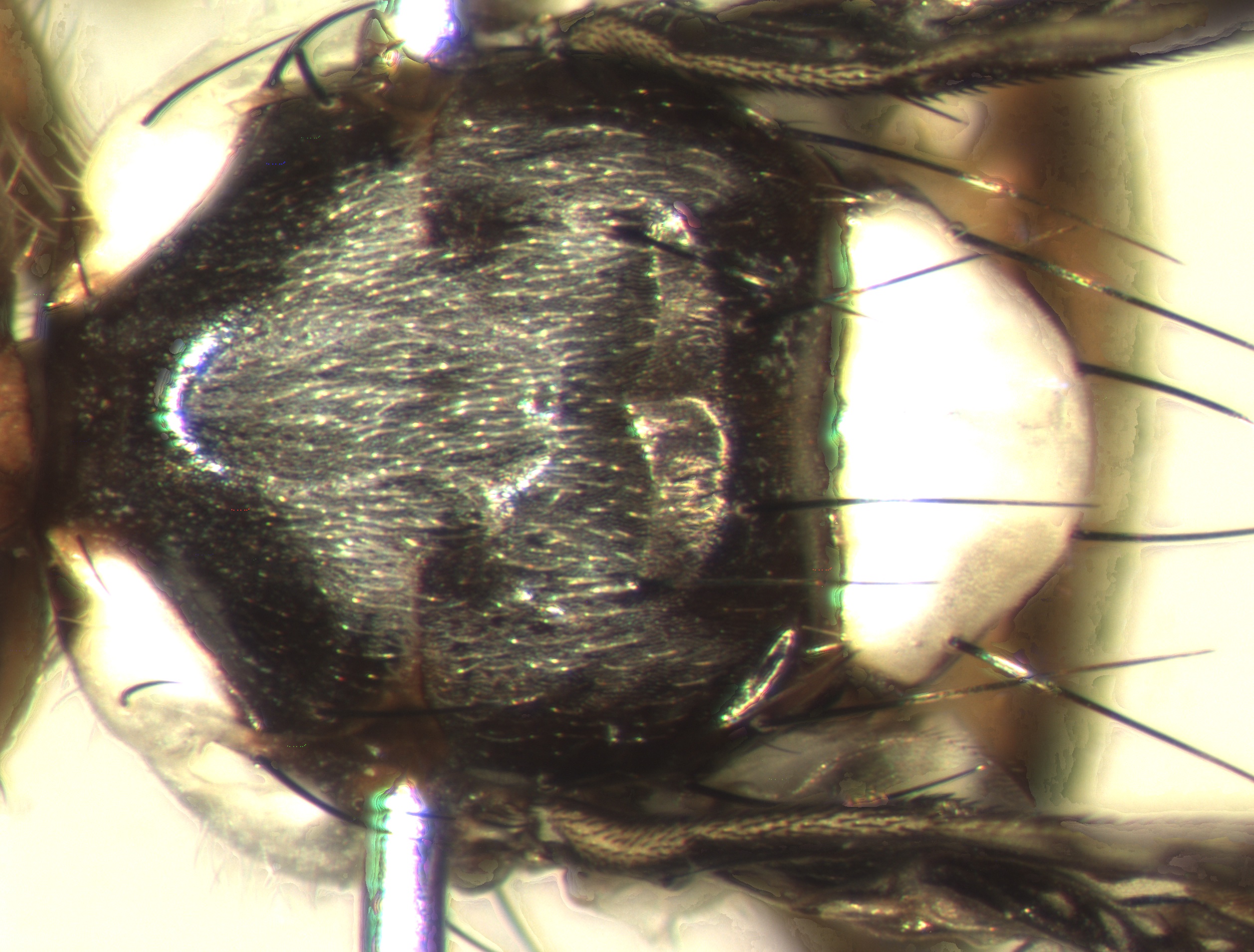

Supplement: Supplementary material 8 — Key to Neoceratitis [file zookeys-428-097-s008.zip › SF8_ZooKeys_key to Neoceratitis/key/SF8_key to Neoceratitis/Media/Images/431 mesonotum (automontage (c) RMCA).jpg]

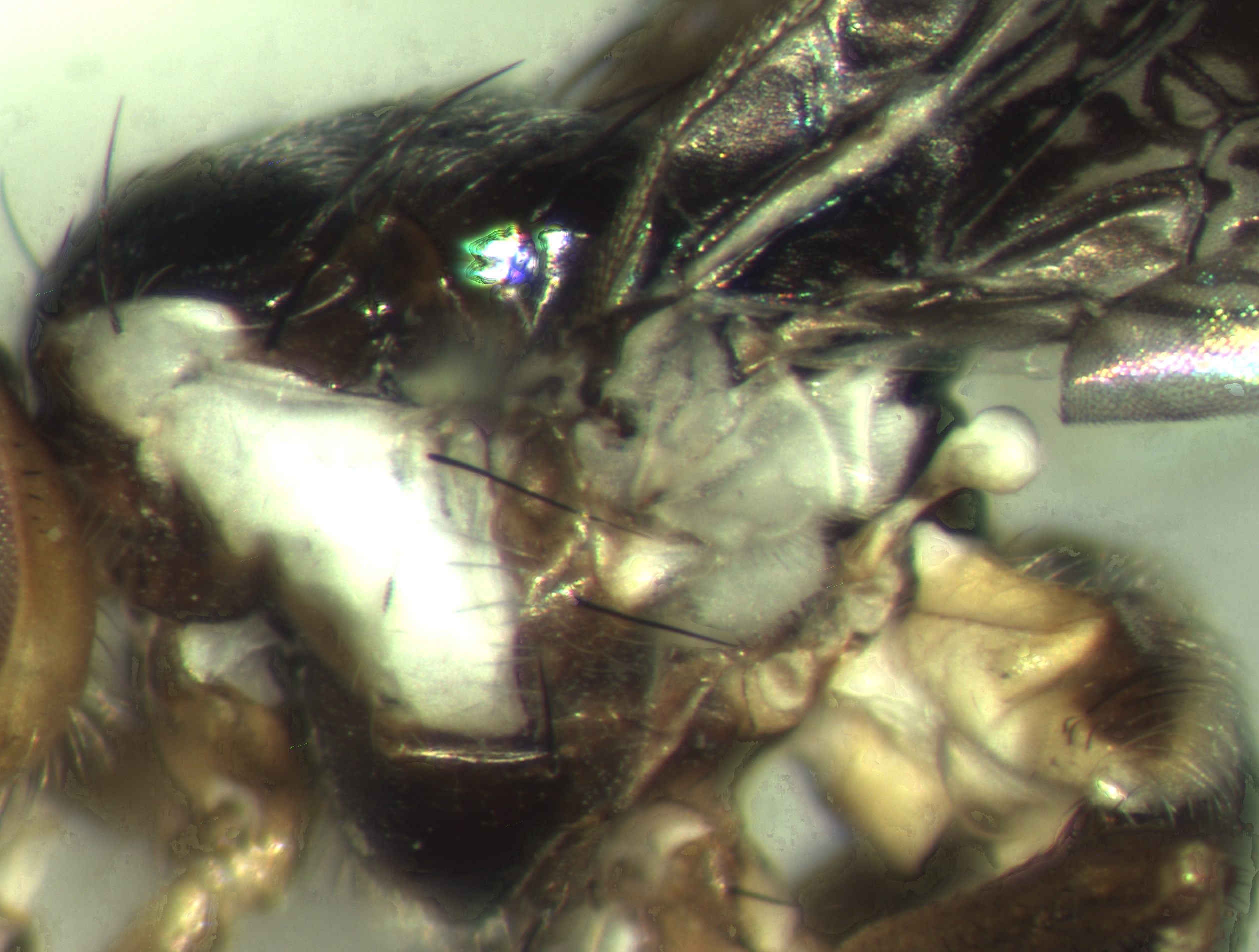

Supplement: Supplementary material 8 — Key to Neoceratitis [file zookeys-428-097-s008.zip › SF8_ZooKeys_key to Neoceratitis/key/SF8_key to Neoceratitis/Media/Images/431 thorax lateral (automontage (c) RMCA).jpg]

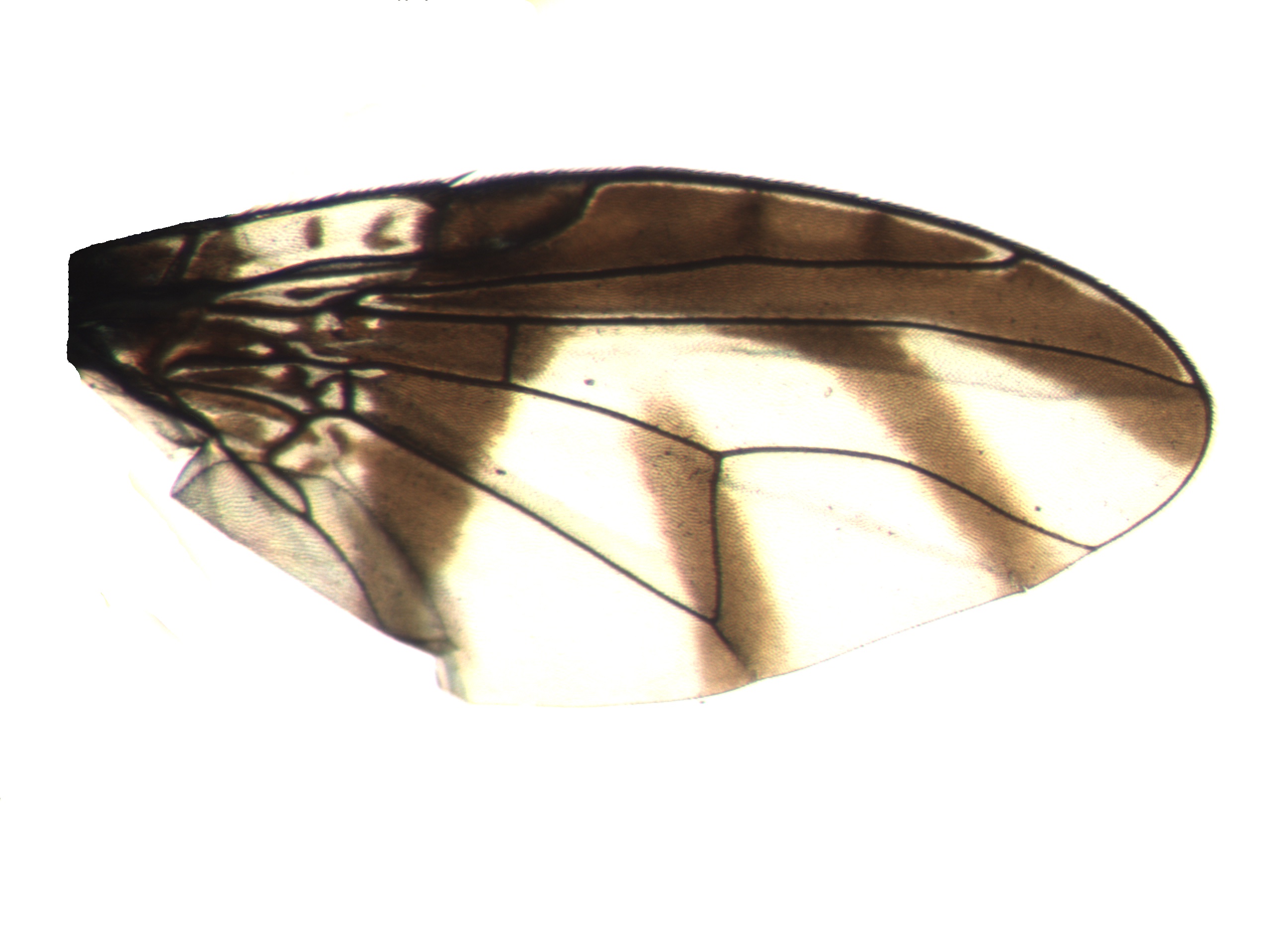

Supplement: Supplementary material 8 — Key to Neoceratitis [file zookeys-428-097-s008.zip › SF8_ZooKeys_key to Neoceratitis/key/SF8_key to Neoceratitis/Media/Images/431 wing (automontage (c) RMCA).jpg]

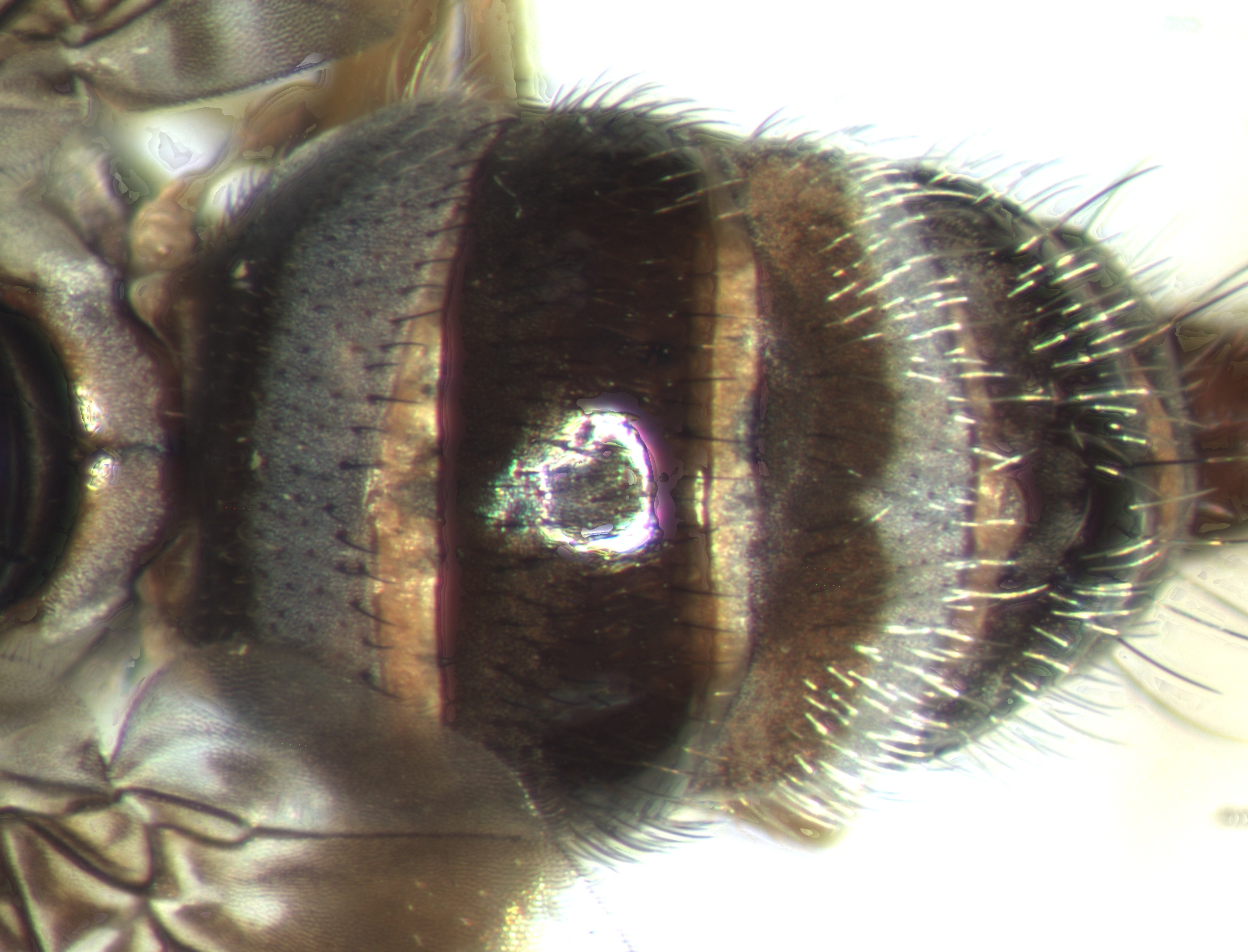

Supplement: Supplementary material 8 — Key to Neoceratitis [file zookeys-428-097-s008.zip › SF8_ZooKeys_key to Neoceratitis/key/SF8_key to Neoceratitis/Media/Images/432 abdomen female dorsal (automontage (c) RMCA).jpg]

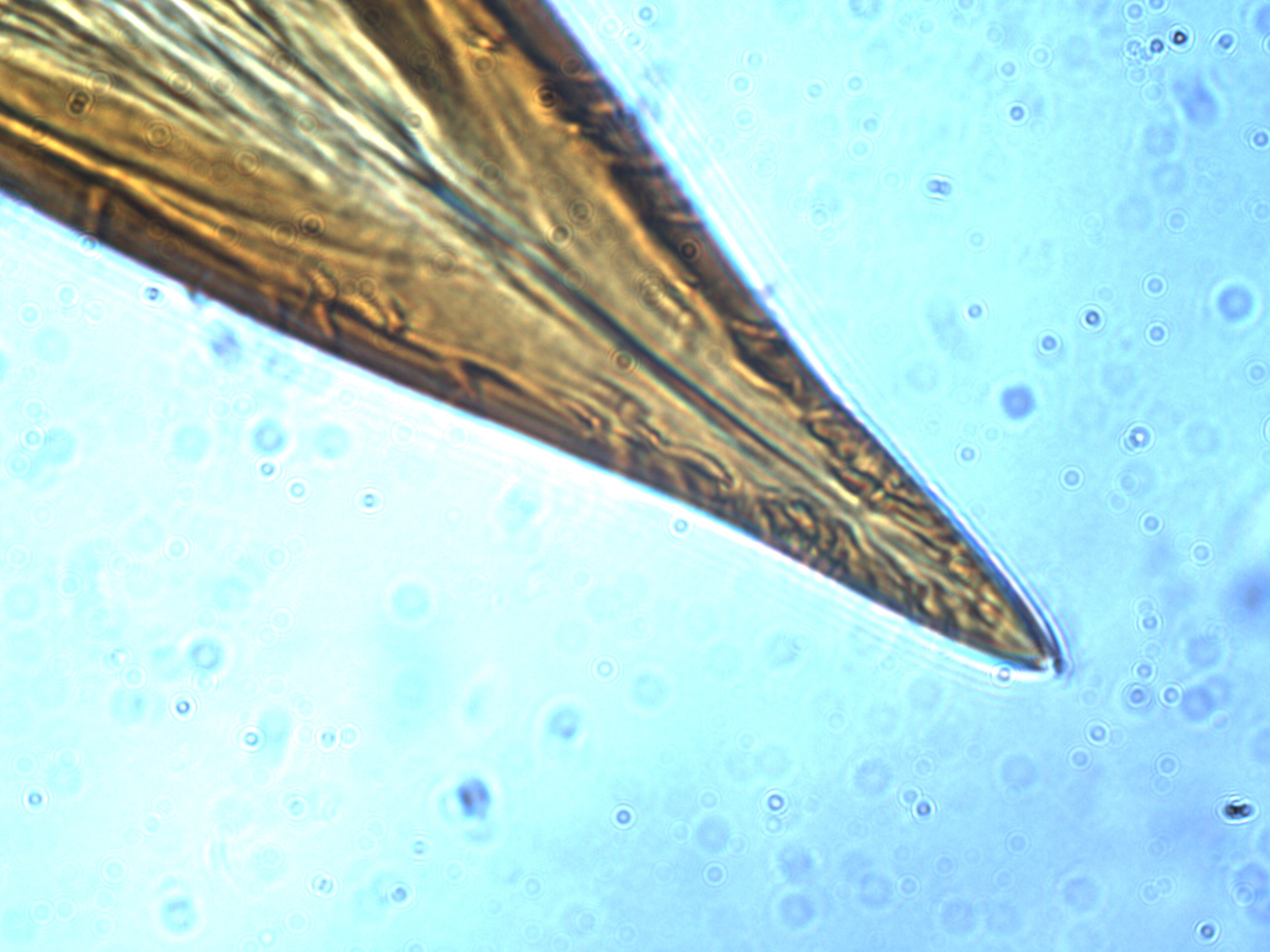

Supplement: Supplementary material 8 — Key to Neoceratitis [file zookeys-428-097-s008.zip › SF8_ZooKeys_key to Neoceratitis/key/SF8_key to Neoceratitis/Media/Images/432 aculeus tip (automontage (c) RMCA).jpg]

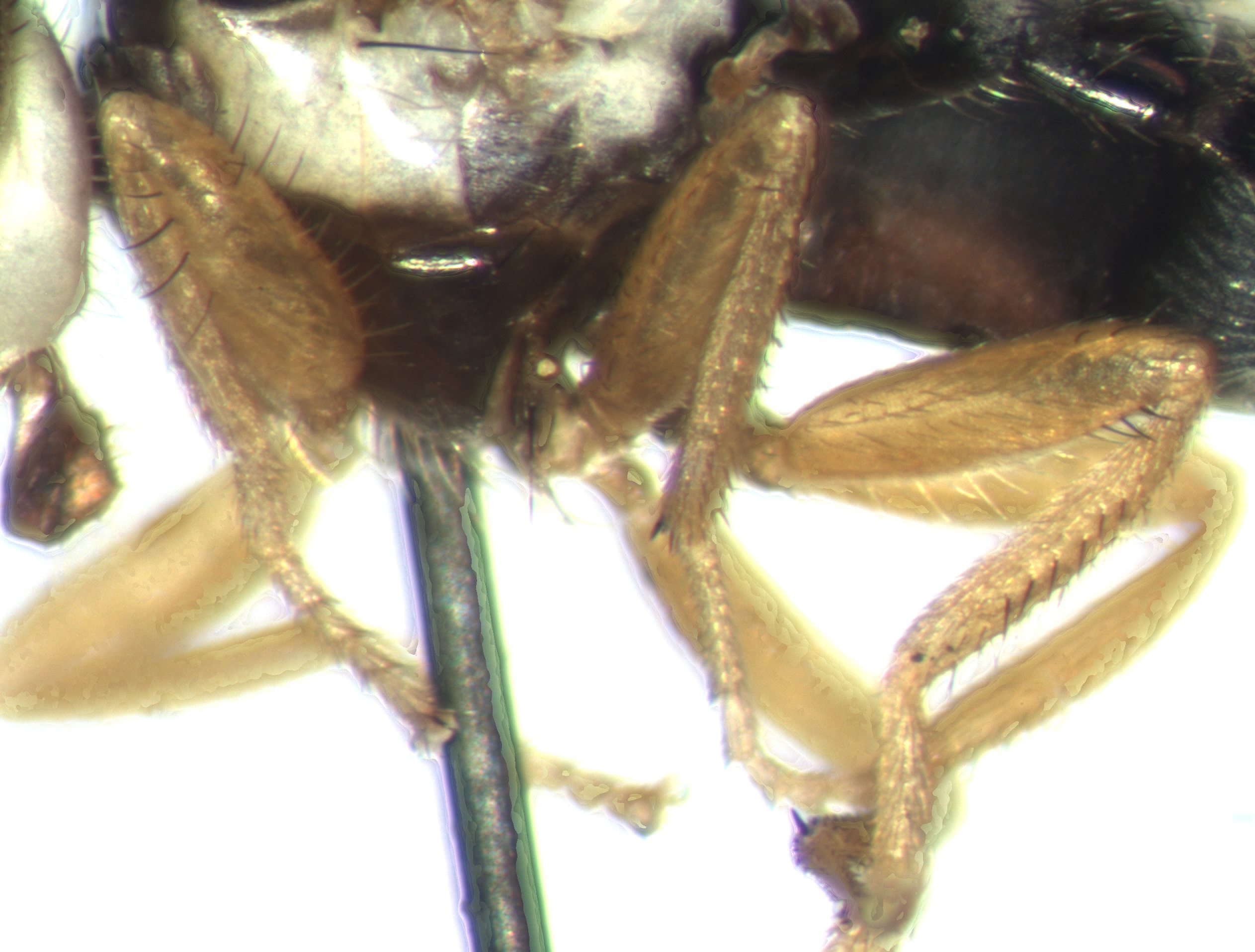

Supplement: Supplementary material 8 — Key to Neoceratitis [file zookeys-428-097-s008.zip › SF8_ZooKeys_key to Neoceratitis/key/SF8_key to Neoceratitis/Media/Images/432 legs lateral (automontage (c) RMCA).jpg]

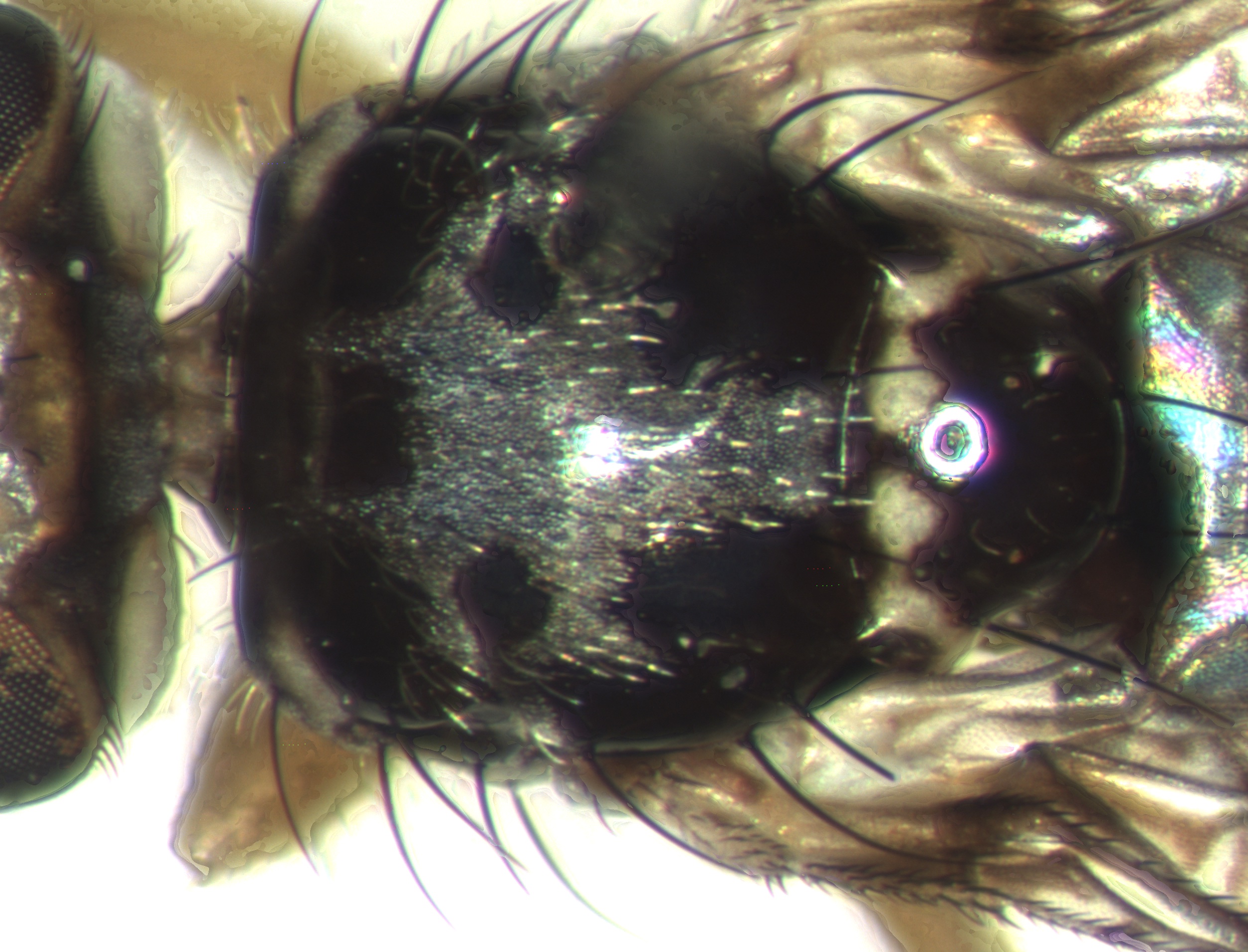

Supplement: Supplementary material 8 — Key to Neoceratitis [file zookeys-428-097-s008.zip › SF8_ZooKeys_key to Neoceratitis/key/SF8_key to Neoceratitis/Media/Images/432 mesonotum (automontage (c) RMCA).jpg]

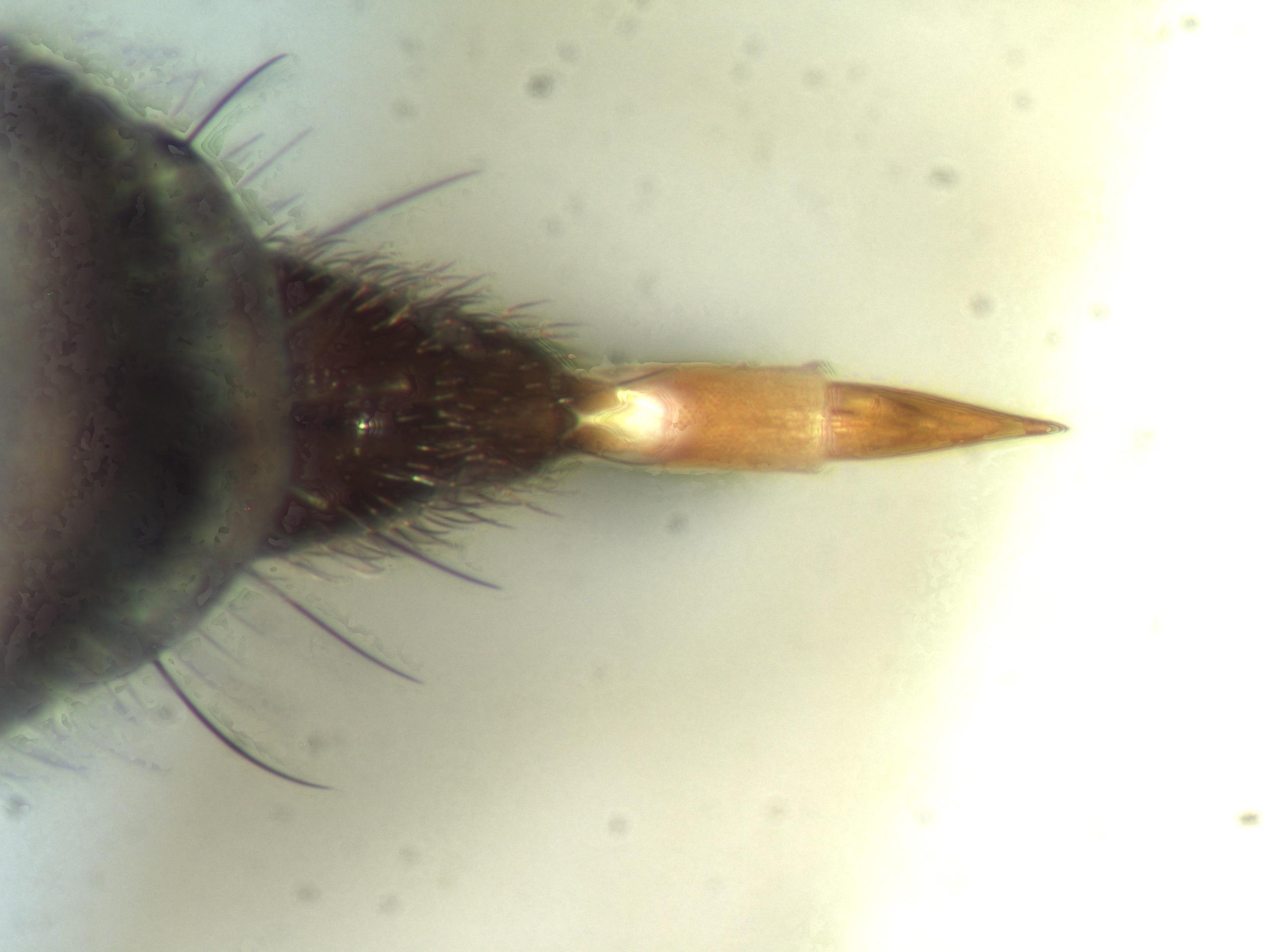

Supplement: Supplementary material 8 — Key to Neoceratitis [file zookeys-428-097-s008.zip › SF8_ZooKeys_key to Neoceratitis/key/SF8_key to Neoceratitis/Media/Images/432 oviscape and aculeus dorsal (automontage (c) RMCA).jpg]

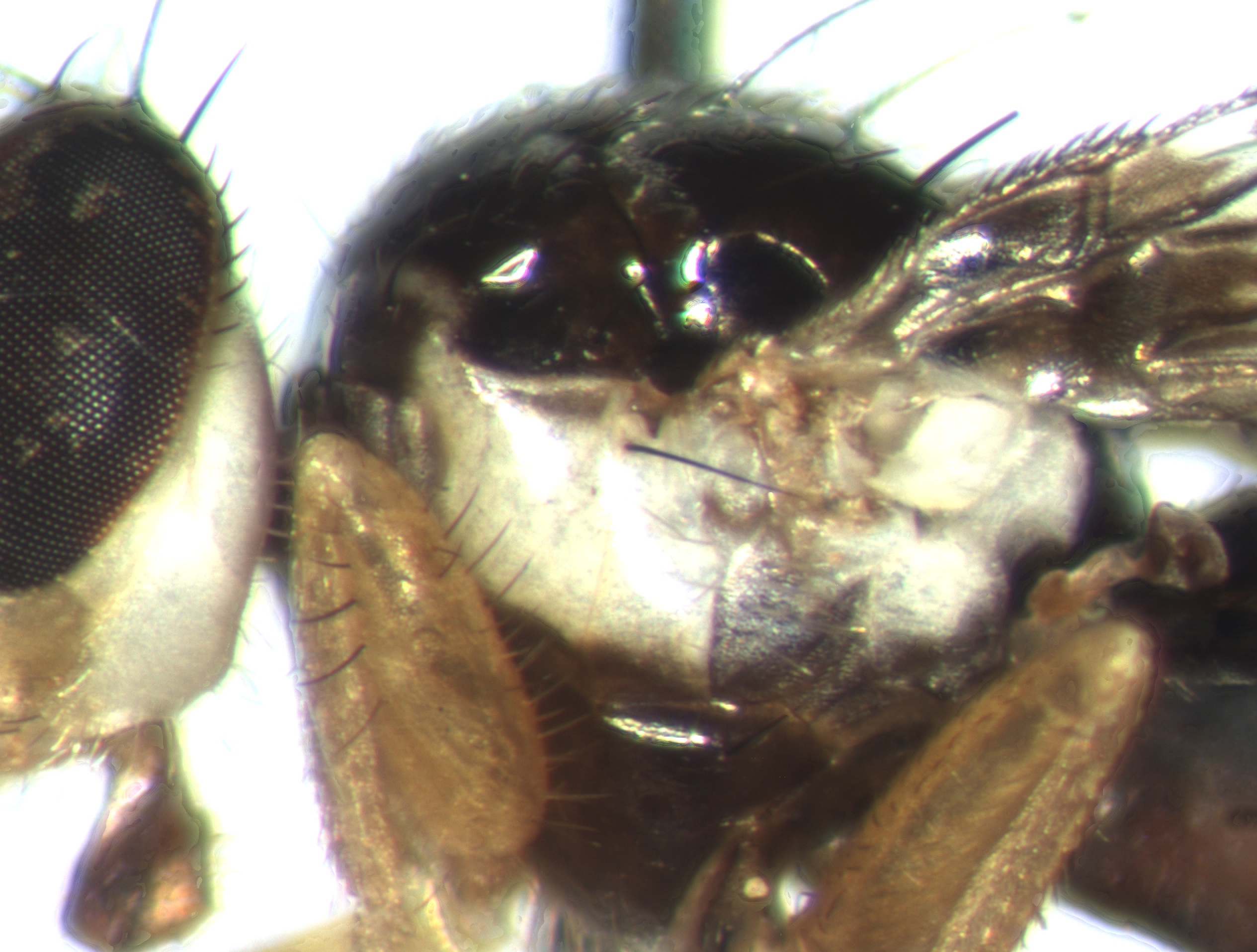

Supplement: Supplementary material 8 — Key to Neoceratitis [file zookeys-428-097-s008.zip › SF8_ZooKeys_key to Neoceratitis/key/SF8_key to Neoceratitis/Media/Images/432 thorax lateral (automontage (c) RMCA).jpg]

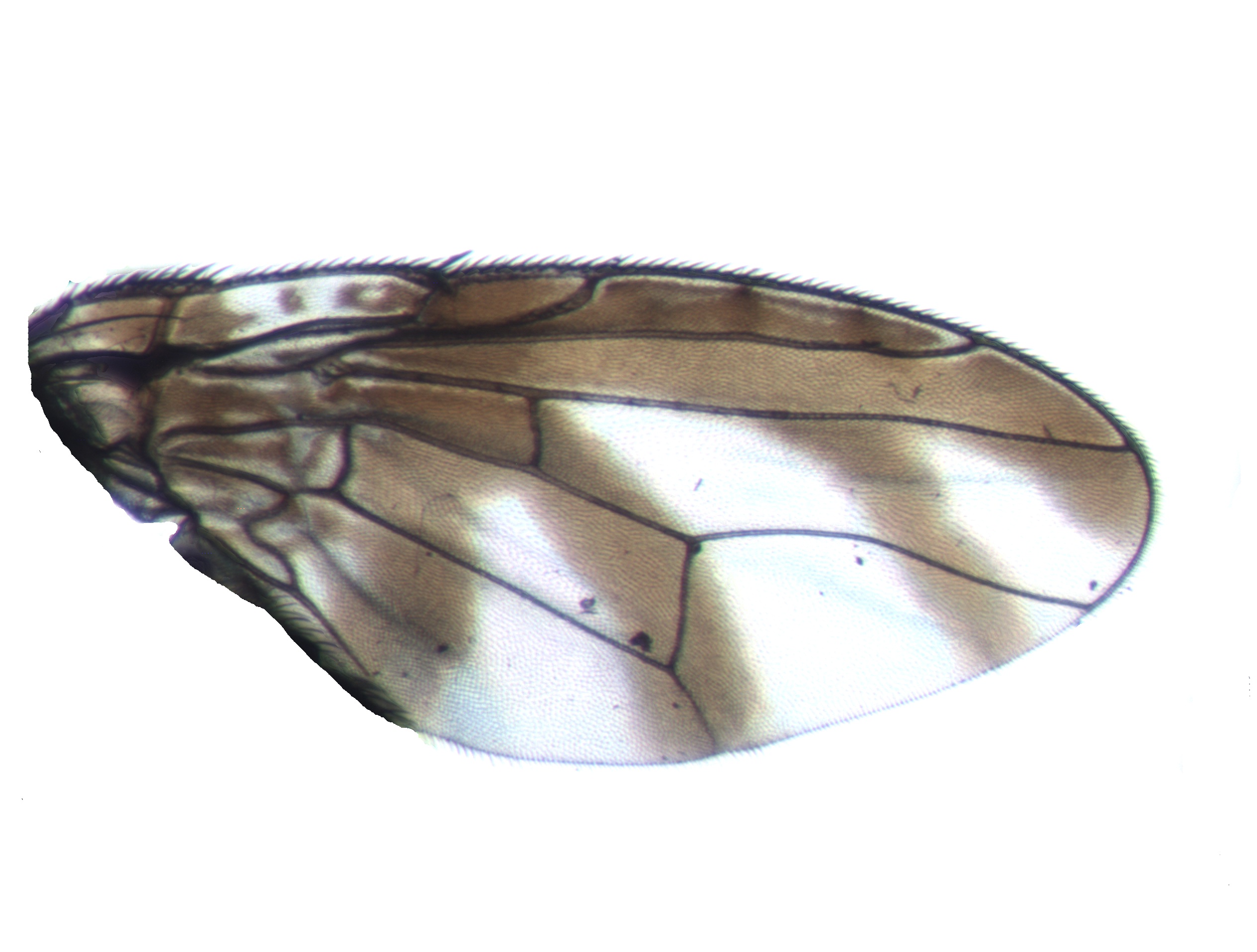

Supplement: Supplementary material 8 — Key to Neoceratitis [file zookeys-428-097-s008.zip › SF8_ZooKeys_key to Neoceratitis/key/SF8_key to Neoceratitis/Media/Images/432 wing (automontage (c) RMCA).jpg]

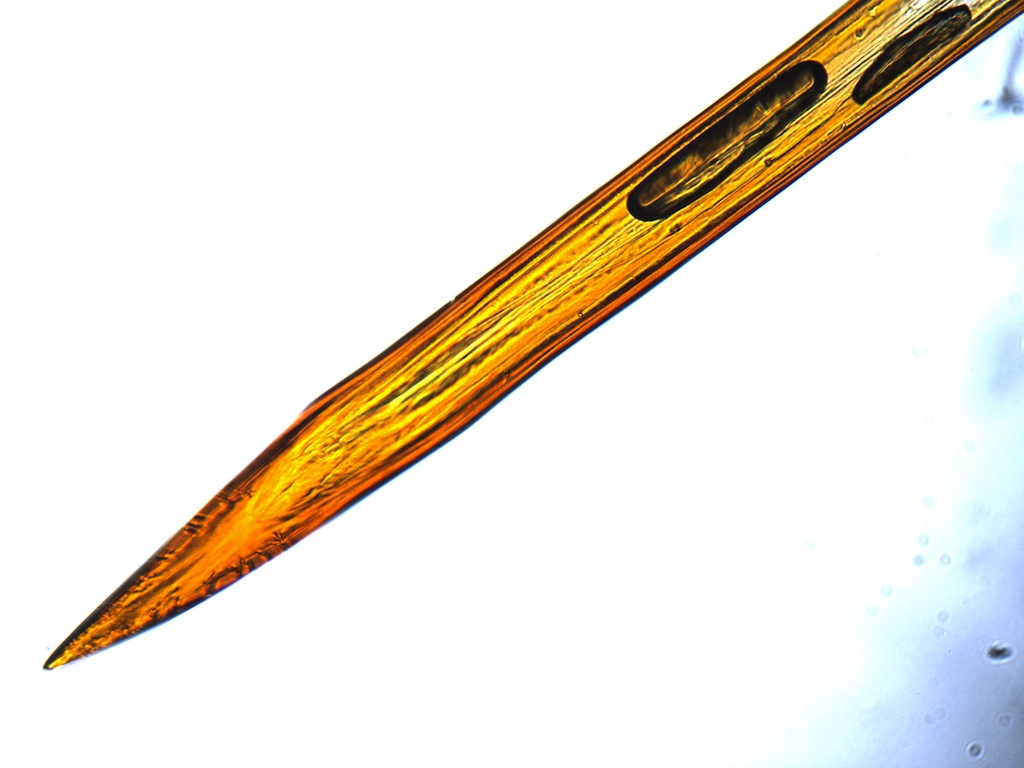

Supplement: Supplementary material 8 — Key to Neoceratitis [file zookeys-428-097-s008.zip › SF8_ZooKeys_key to Neoceratitis/key/SF8_key to Neoceratitis/Media/Images/char10_10x_431 aculeus(automontage (c) RMCA).jpg]

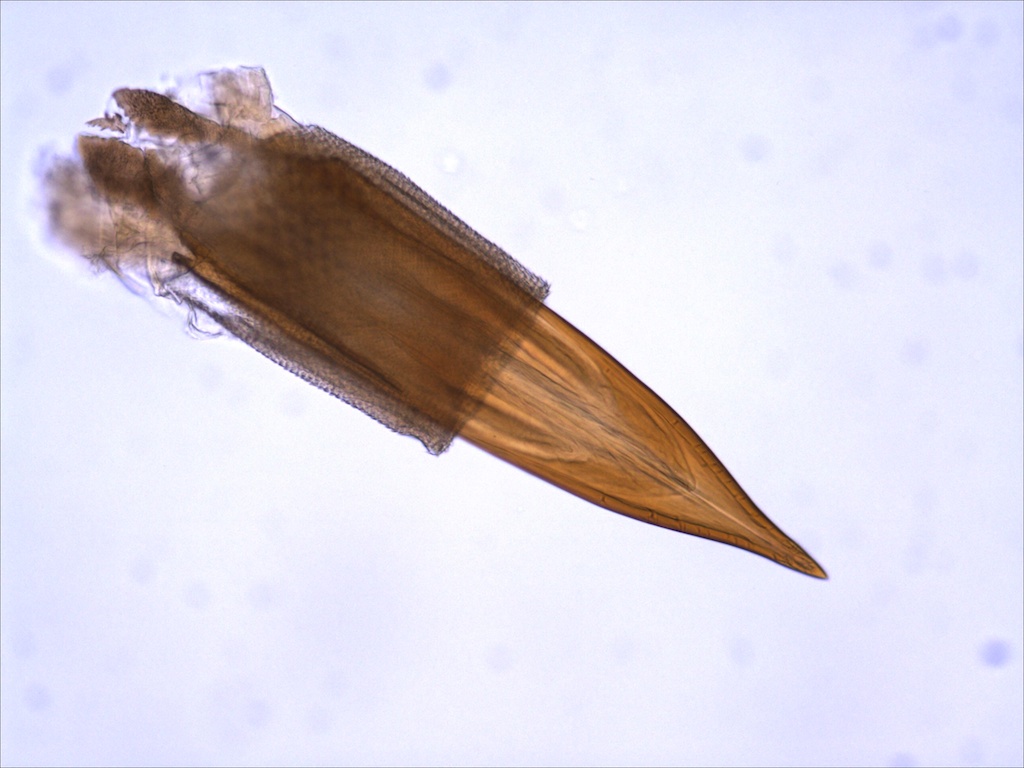

Supplement: Supplementary material 8 — Key to Neoceratitis [file zookeys-428-097-s008.zip › SF8_ZooKeys_key to Neoceratitis/key/SF8_key to Neoceratitis/Media/Images/char10_4_6x_429 aculeus(automontage (c) RMCA).jpg]

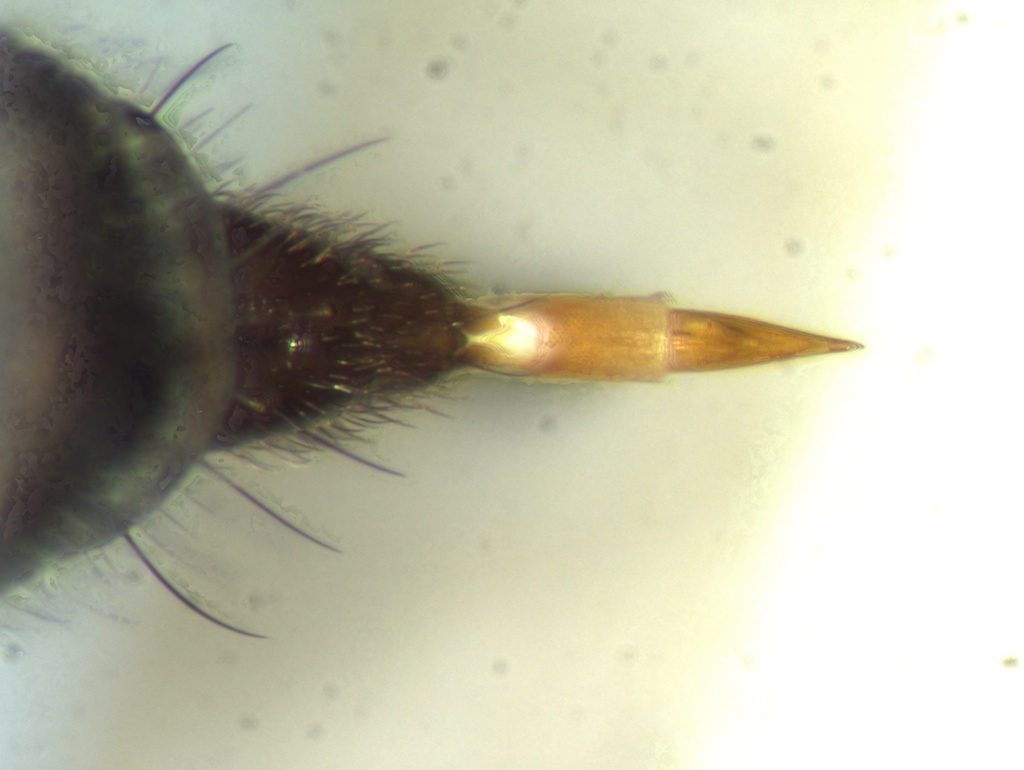

Supplement: Supplementary material 8 — Key to Neoceratitis [file zookeys-428-097-s008.zip › SF8_ZooKeys_key to Neoceratitis/key/SF8_key to Neoceratitis/Media/Images/char10_4_6x_432 oviscape and aculeus dorsal (automontage (c) RMCA).jpg]

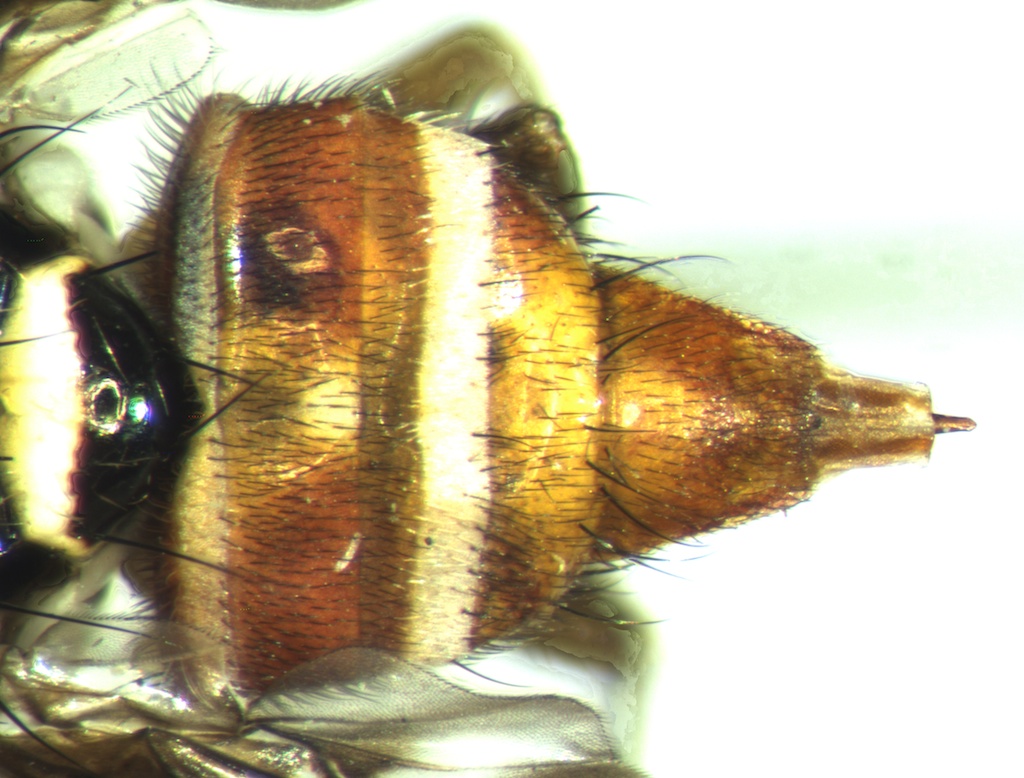

Supplement: Supplementary material 8 — Key to Neoceratitis [file zookeys-428-097-s008.zip › SF8_ZooKeys_key to Neoceratitis/key/SF8_key to Neoceratitis/Media/Images/char11_female_158 abdomen female dorsal (automontage (c) RMCA).jpg]

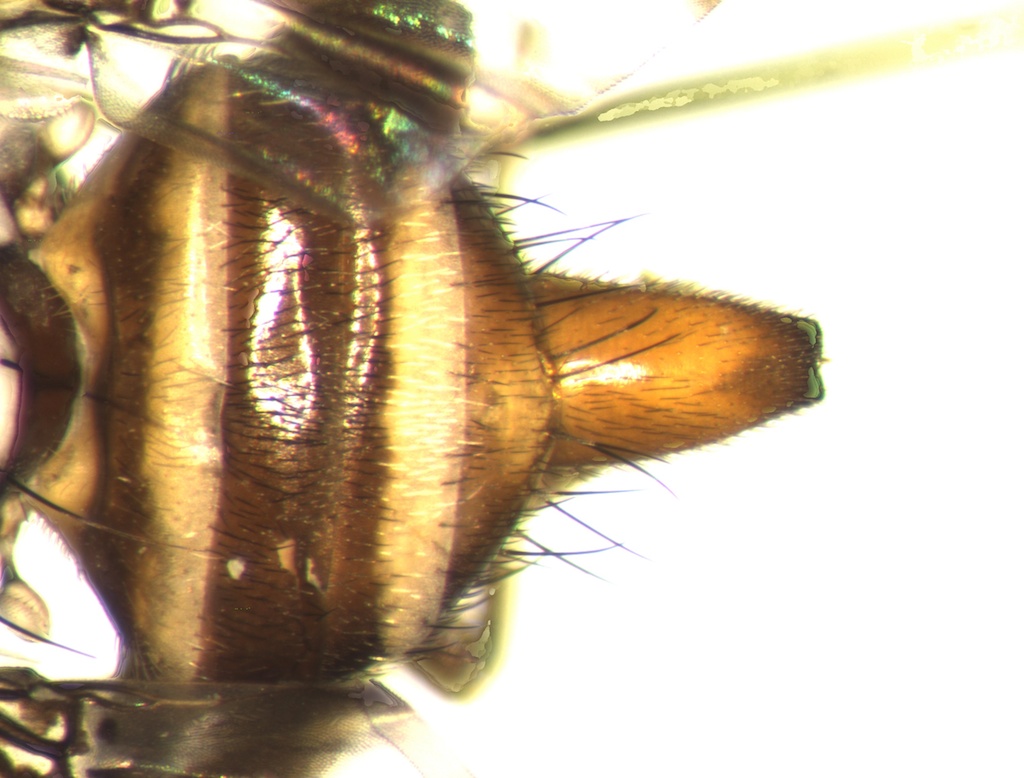

Supplement: Supplementary material 8 — Key to Neoceratitis [file zookeys-428-097-s008.zip › SF8_ZooKeys_key to Neoceratitis/key/SF8_key to Neoceratitis/Media/Images/char11_female_431 abdomen female dorsal (automontage (c) RMCA).jpg]

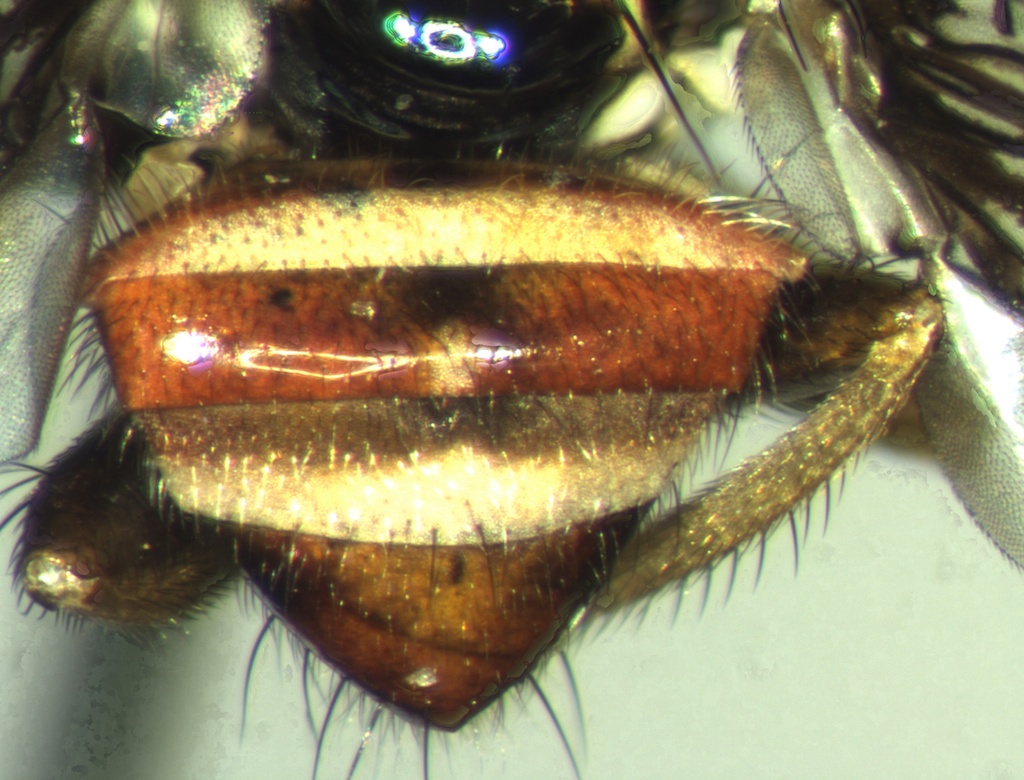

Supplement: Supplementary material 8 — Key to Neoceratitis [file zookeys-428-097-s008.zip › SF8_ZooKeys_key to Neoceratitis/key/SF8_key to Neoceratitis/Media/Images/char11_male_158 abdomen male dorsal (automontage (c) RMCA).jpg]

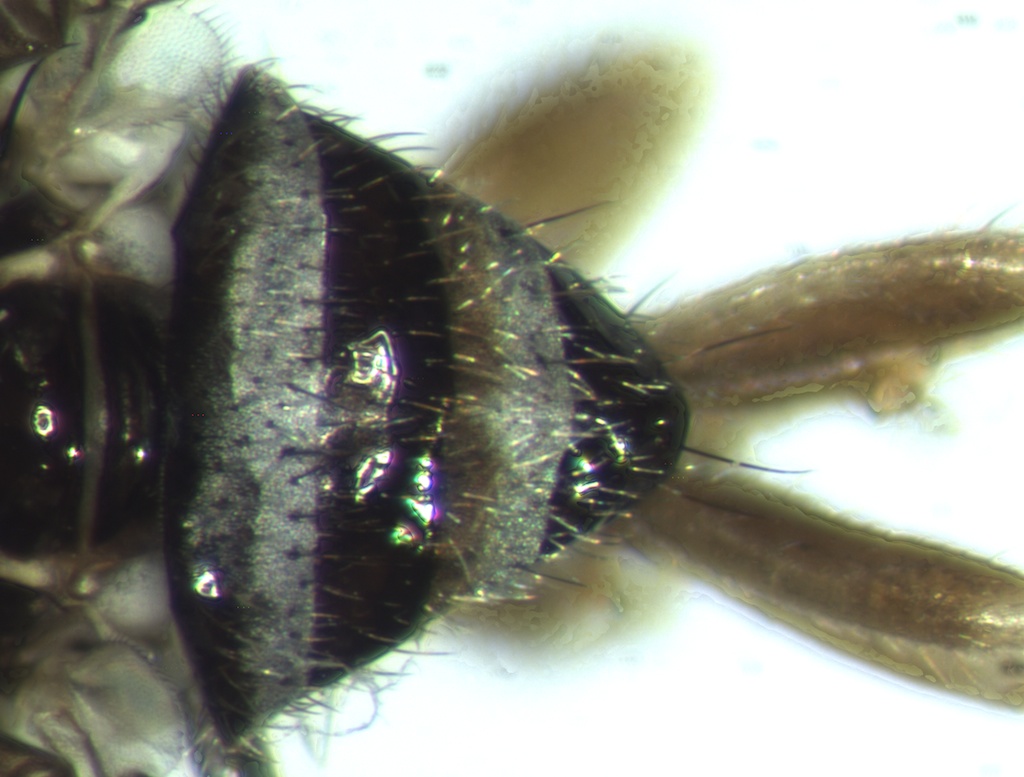

Supplement: Supplementary material 8 — Key to Neoceratitis [file zookeys-428-097-s008.zip › SF8_ZooKeys_key to Neoceratitis/key/SF8_key to Neoceratitis/Media/Images/char11_male_430 abdomen male dorsal (automontage (c) RMCA).jpg]

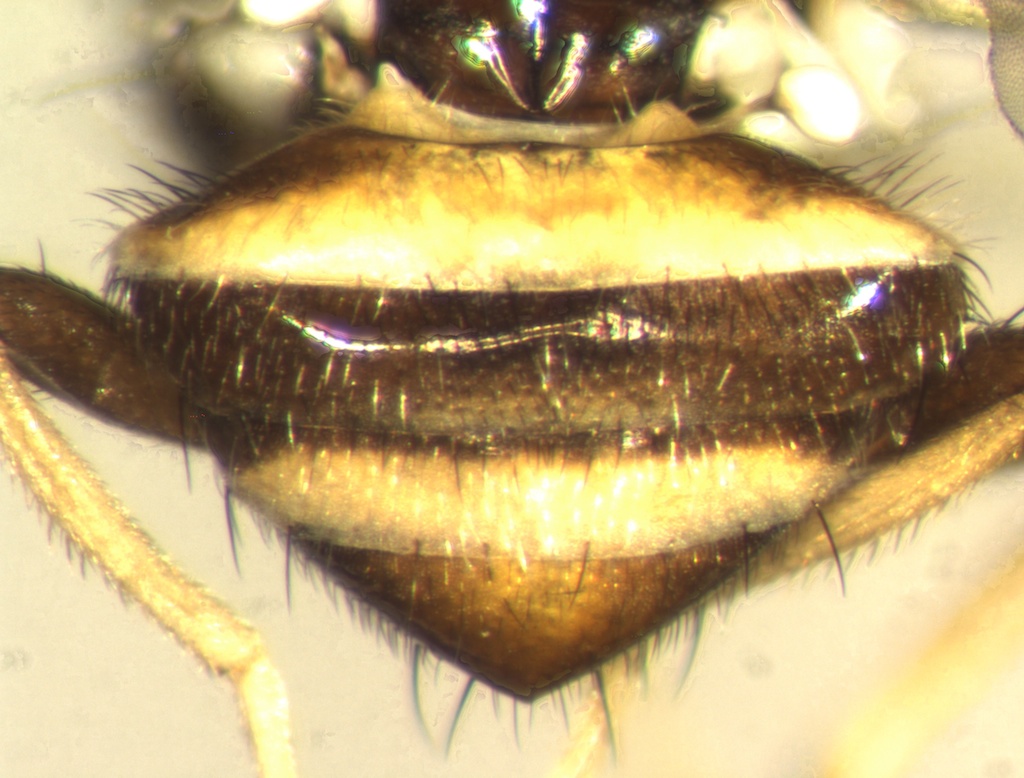

Supplement: Supplementary material 8 — Key to Neoceratitis [file zookeys-428-097-s008.zip › SF8_ZooKeys_key to Neoceratitis/key/SF8_key to Neoceratitis/Media/Images/char11_male_431 abdomen male dorsal (automontage (c) RMCA).jpg]

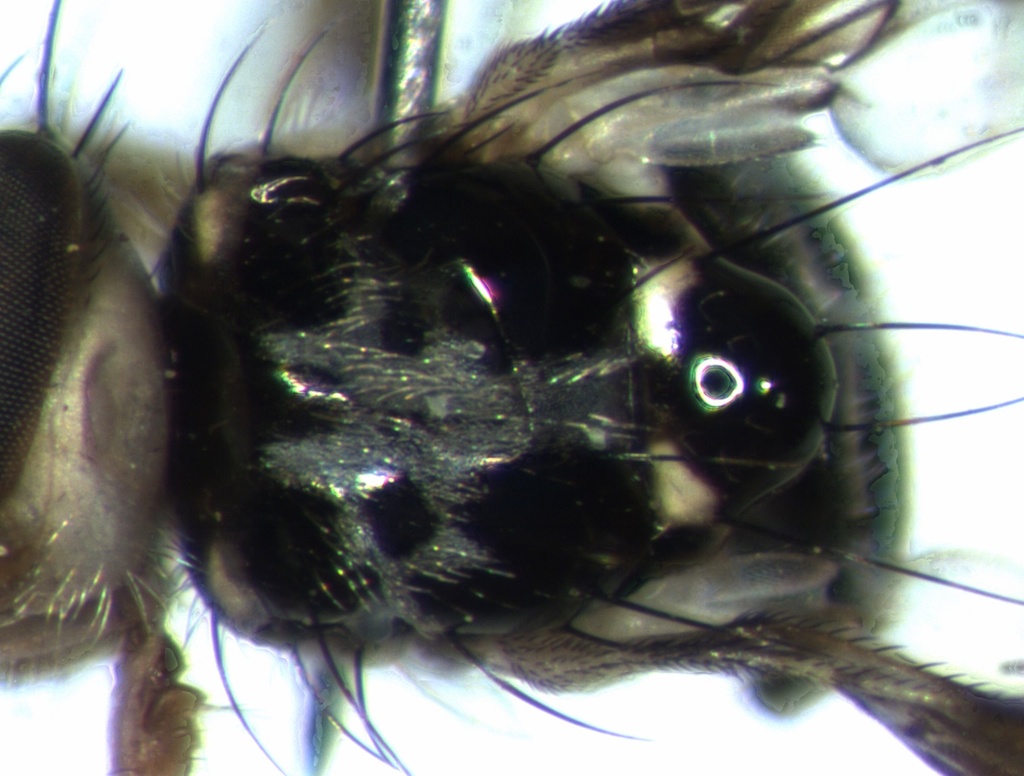

Supplement: Supplementary material 8 — Key to Neoceratitis [file zookeys-428-097-s008.zip › SF8_ZooKeys_key to Neoceratitis/key/SF8_key to Neoceratitis/Media/Images/char1_dark_429 mesonotum (automontage (c) RMCA).jpg]

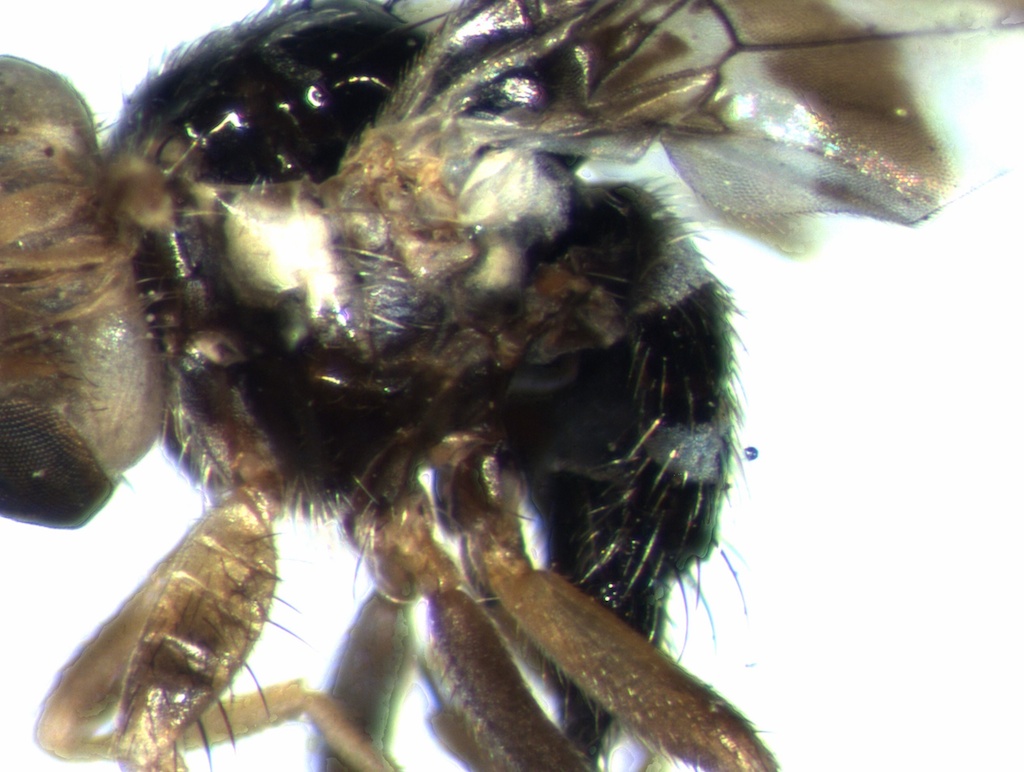

Supplement: Supplementary material 8 — Key to Neoceratitis [file zookeys-428-097-s008.zip › SF8_ZooKeys_key to Neoceratitis/key/SF8_key to Neoceratitis/Media/Images/char1_dark_429 thorax lateral (automontage (c) RMCA).jpg]

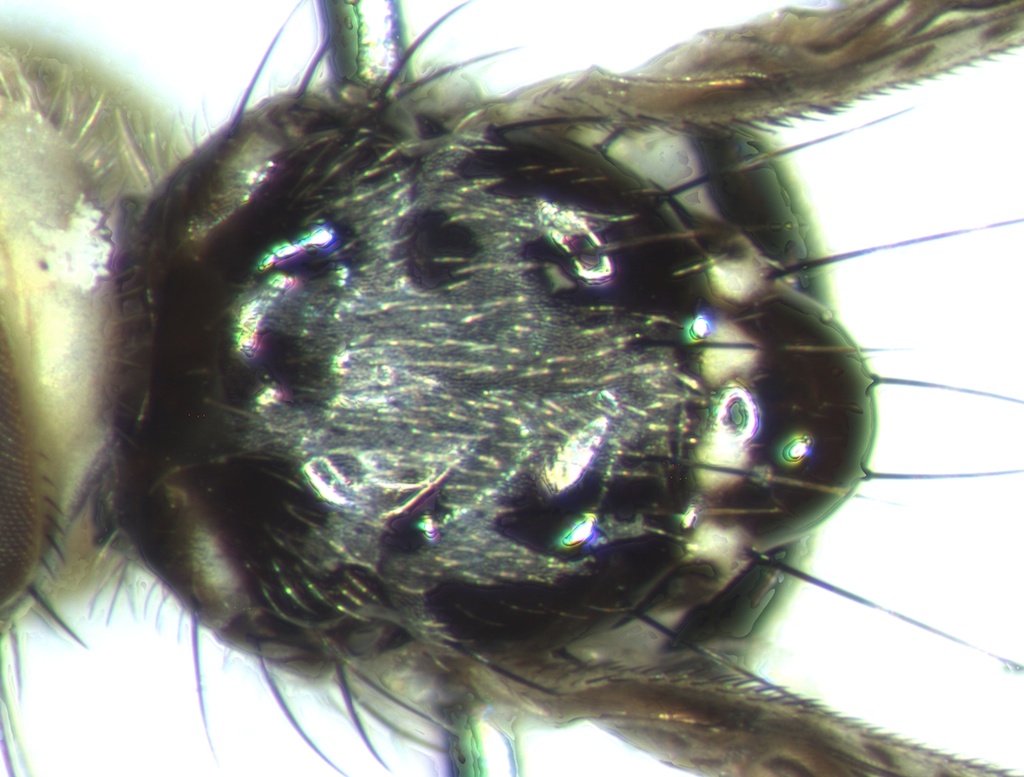

Supplement: Supplementary material 8 — Key to Neoceratitis [file zookeys-428-097-s008.zip › SF8_ZooKeys_key to Neoceratitis/key/SF8_key to Neoceratitis/Media/Images/char1_dark_430 mesonotum (automontage (c) RMCA).jpg]

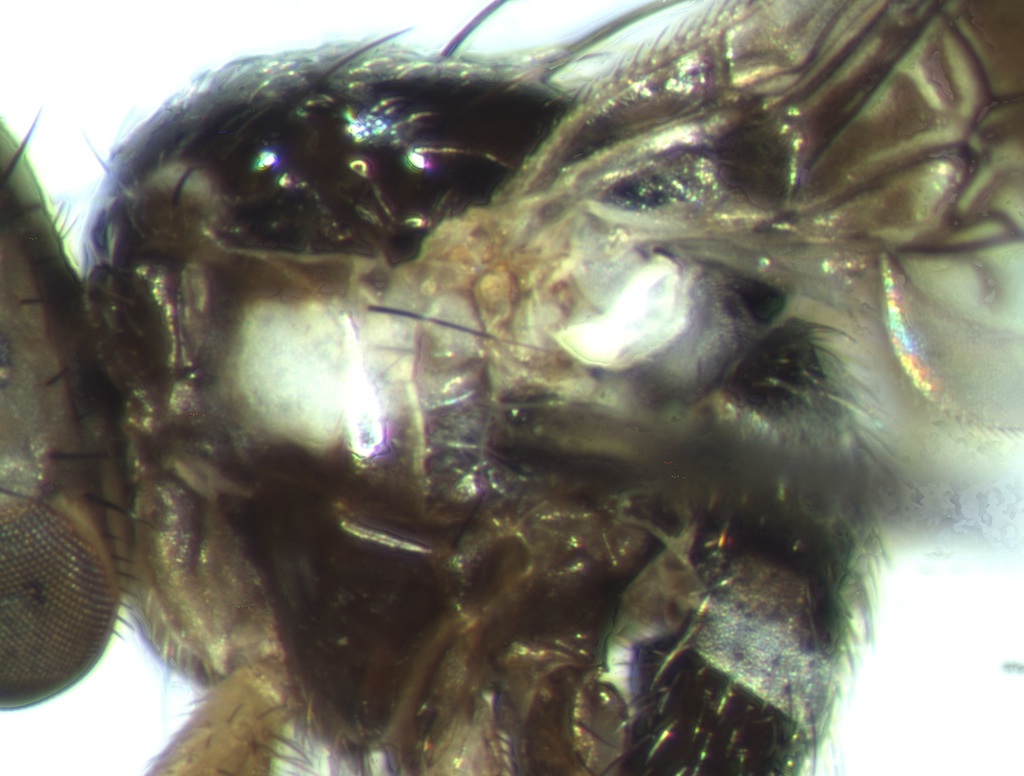

Supplement: Supplementary material 8 — Key to Neoceratitis [file zookeys-428-097-s008.zip › SF8_ZooKeys_key to Neoceratitis/key/SF8_key to Neoceratitis/Media/Images/char1_dark_430 thorax lateral (automontage (c) RMCA).jpg]

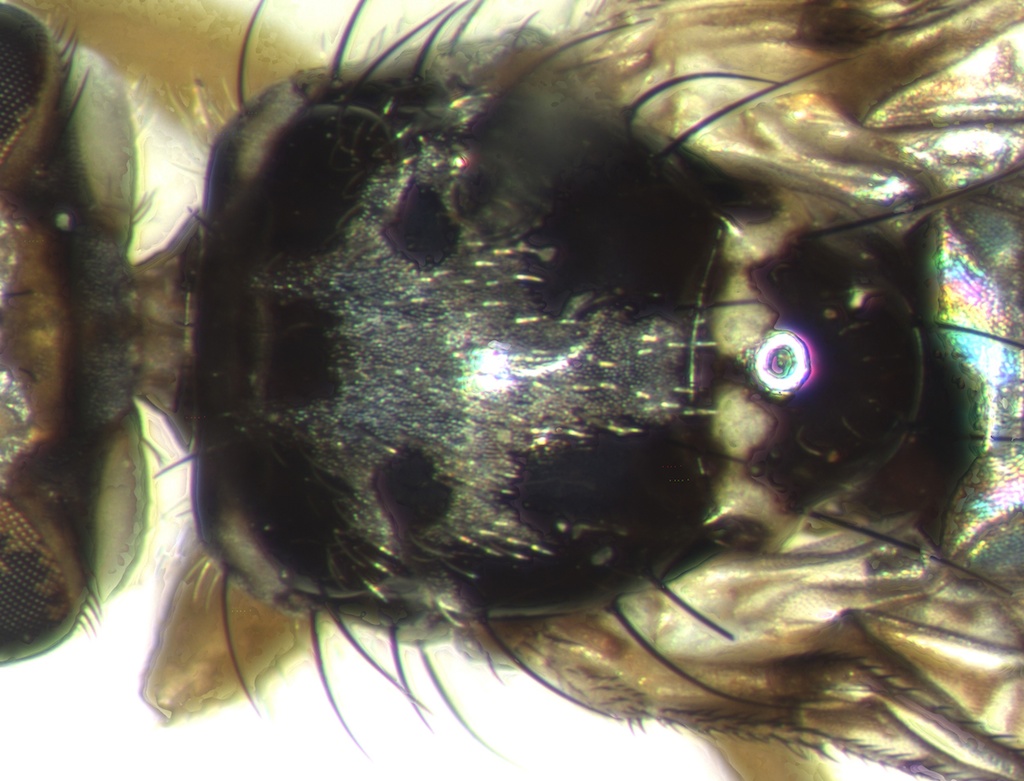

Supplement: Supplementary material 8 — Key to Neoceratitis [file zookeys-428-097-s008.zip › SF8_ZooKeys_key to Neoceratitis/key/SF8_key to Neoceratitis/Media/Images/char1_dark_432 mesonotum (automontage (c) RMCA).jpg]

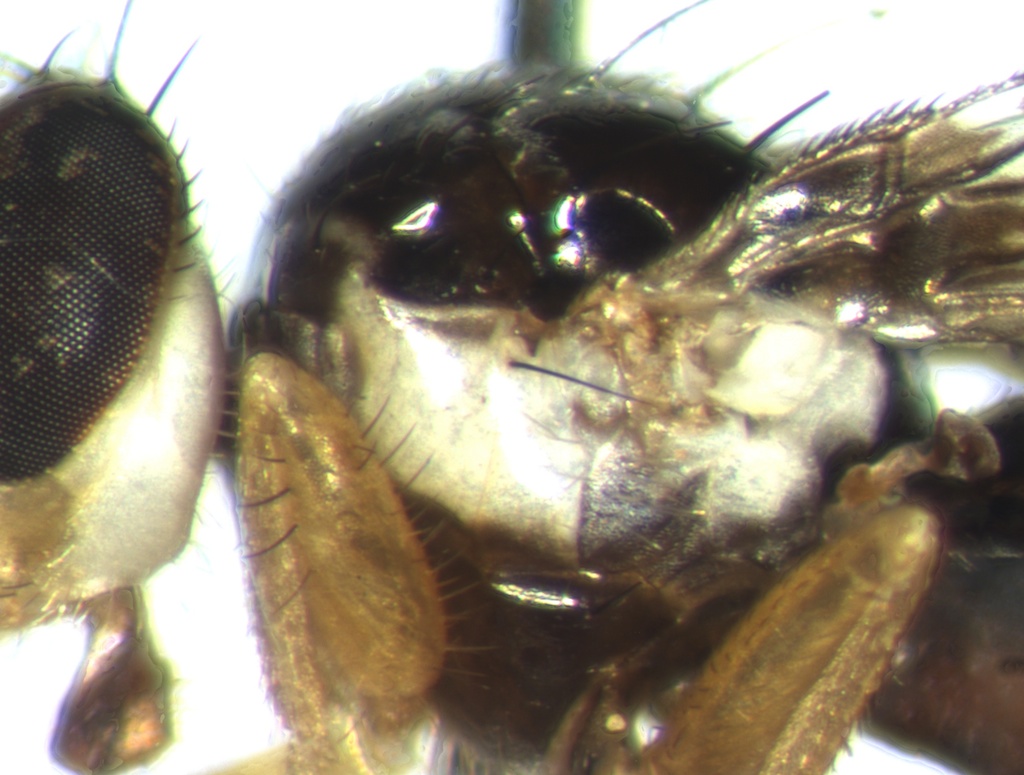

Supplement: Supplementary material 8 — Key to Neoceratitis [file zookeys-428-097-s008.zip › SF8_ZooKeys_key to Neoceratitis/key/SF8_key to Neoceratitis/Media/Images/char1_dark_432 thorax lateral (automontage (c) RMCA).jpg]

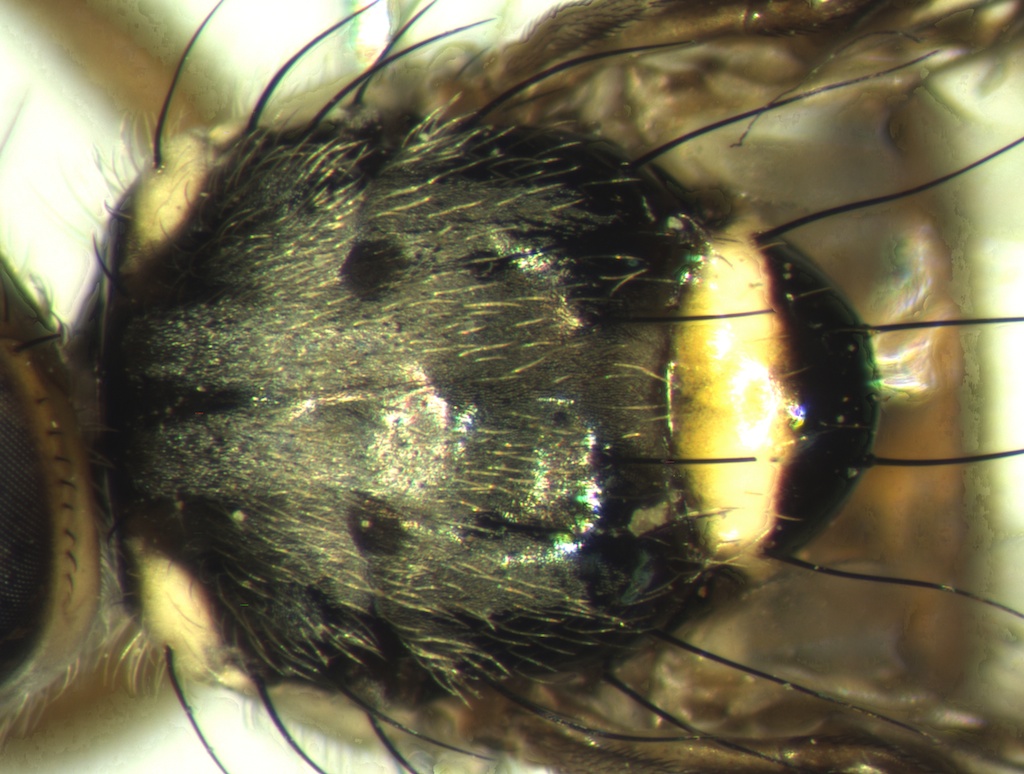

Supplement: Supplementary material 8 — Key to Neoceratitis [file zookeys-428-097-s008.zip › SF8_ZooKeys_key to Neoceratitis/key/SF8_key to Neoceratitis/Media/Images/char1_pale_158 mesonotum (automontage (c) RMCA).jpg]

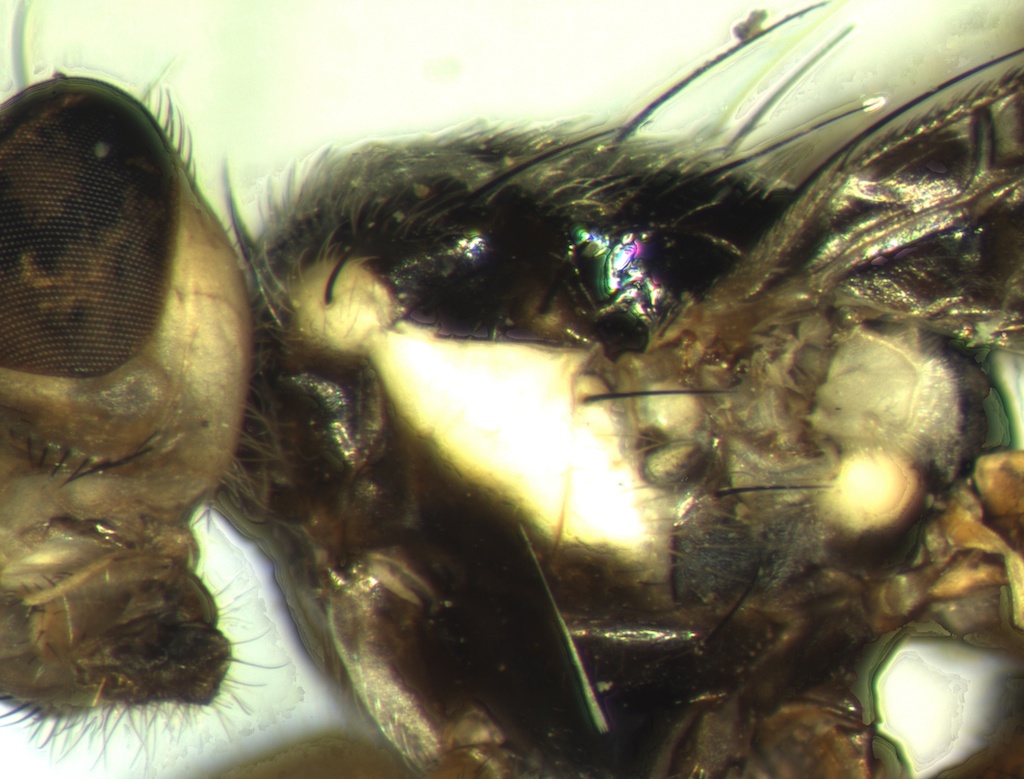

Supplement: Supplementary material 8 — Key to Neoceratitis [file zookeys-428-097-s008.zip › SF8_ZooKeys_key to Neoceratitis/key/SF8_key to Neoceratitis/Media/Images/char1_pale_158 thorax lateral (automontage (c) RMCA).jpg]

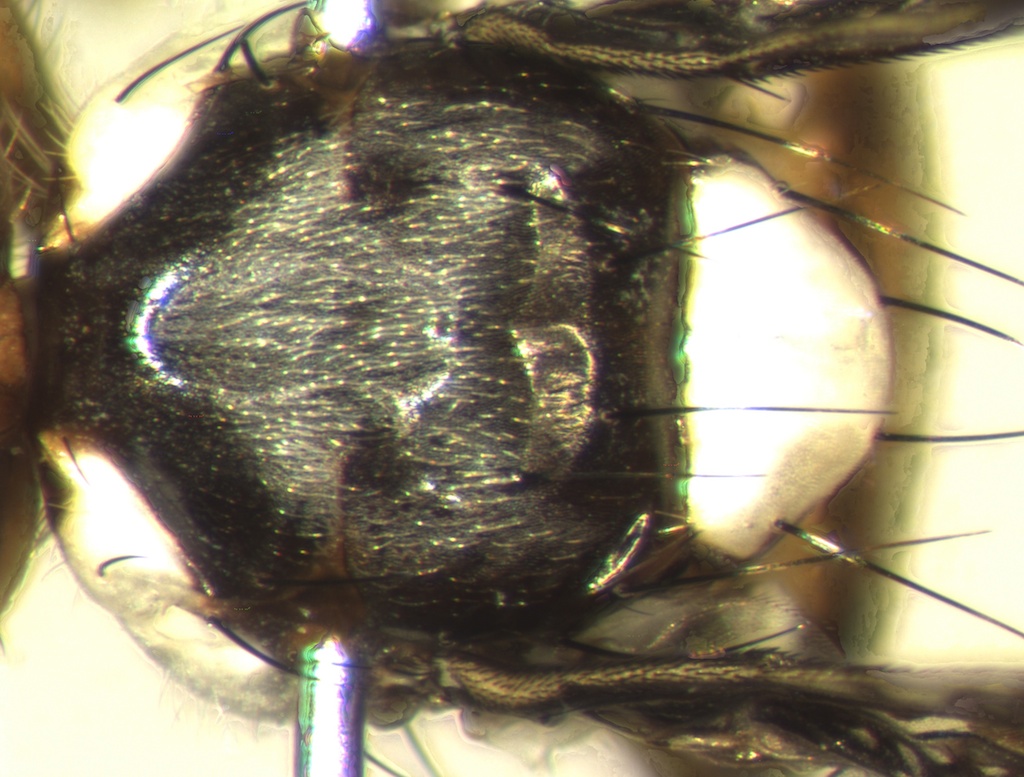

Supplement: Supplementary material 8 — Key to Neoceratitis [file zookeys-428-097-s008.zip › SF8_ZooKeys_key to Neoceratitis/key/SF8_key to Neoceratitis/Media/Images/char1_pale_431 mesonotum (automontage (c) RMCA).jpg]

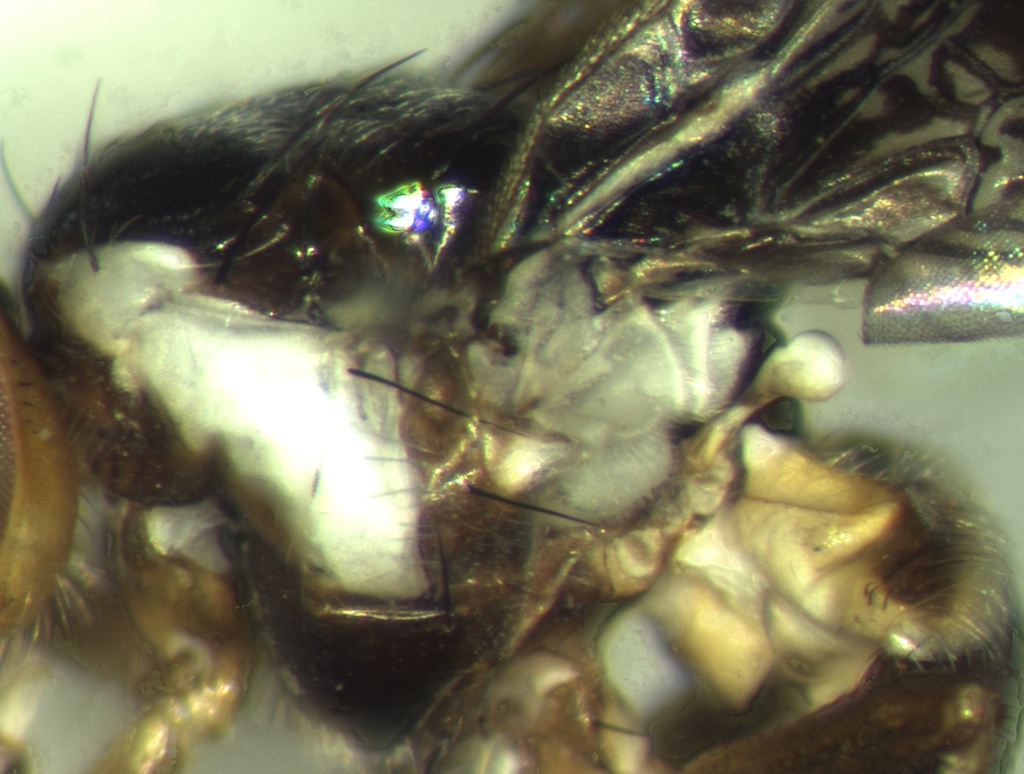

Supplement: Supplementary material 8 — Key to Neoceratitis [file zookeys-428-097-s008.zip › SF8_ZooKeys_key to Neoceratitis/key/SF8_key to Neoceratitis/Media/Images/char1_pale_431 thorax lateral (automontage (c) RMCA).jpg]

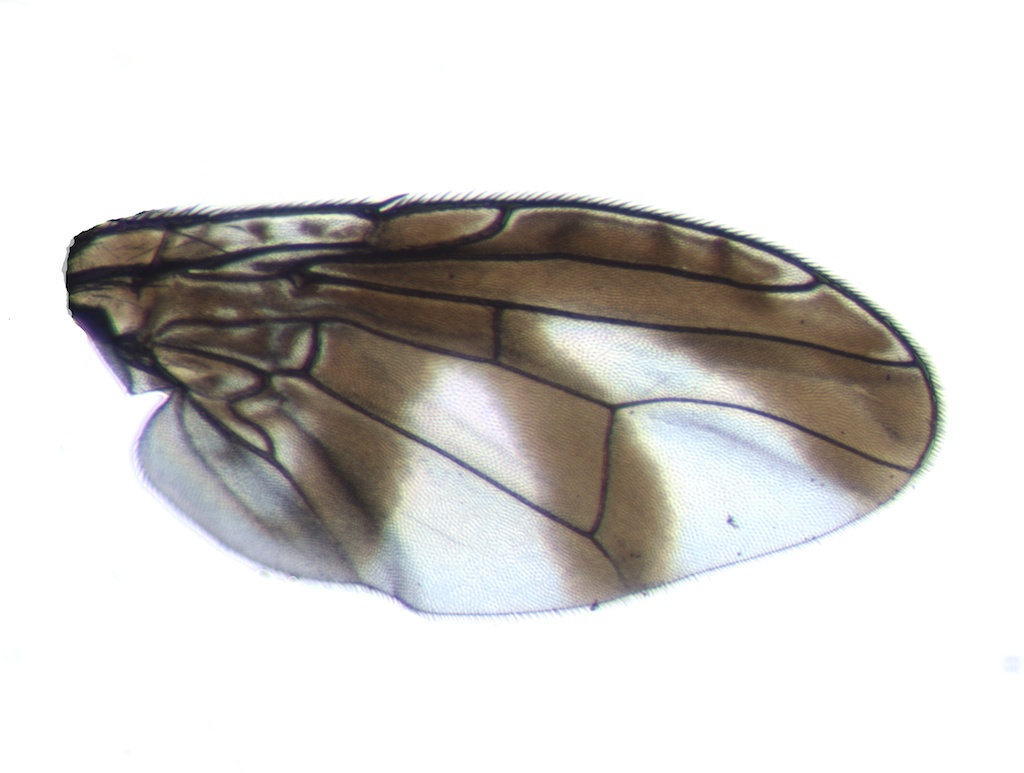

Supplement: Supplementary material 8 — Key to Neoceratitis [file zookeys-428-097-s008.zip › SF8_ZooKeys_key to Neoceratitis/key/SF8_key to Neoceratitis/Media/Images/char6_confluent_430 wing (automontage (c) RMCA).jpg]

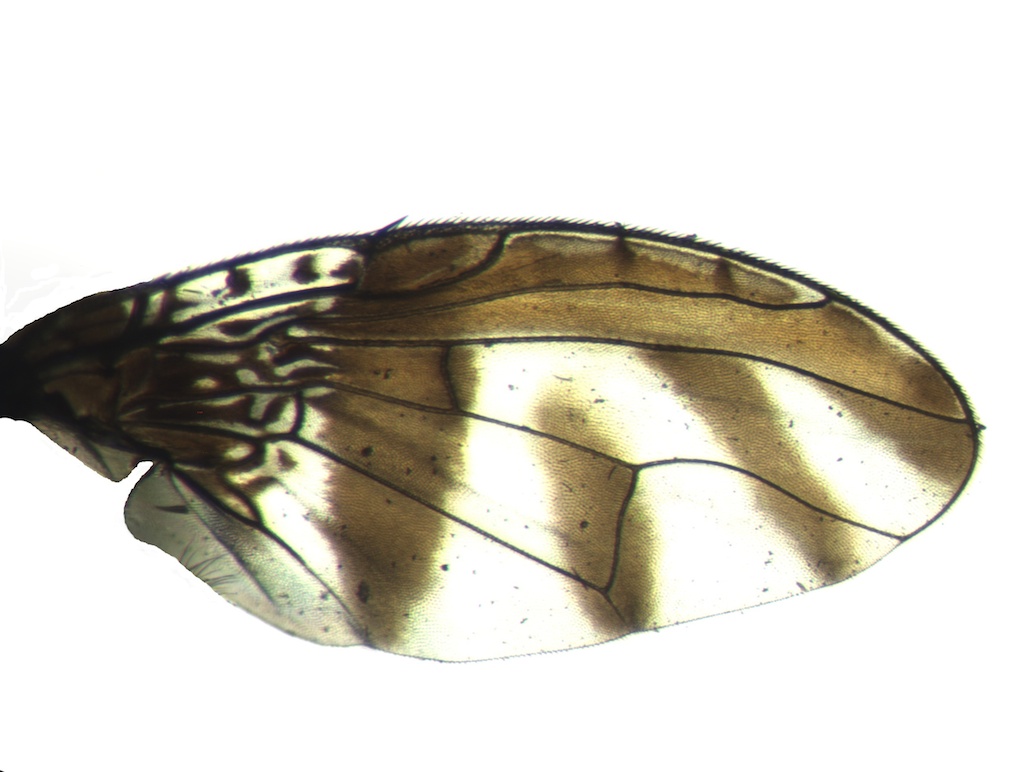

Supplement: Supplementary material 8 — Key to Neoceratitis [file zookeys-428-097-s008.zip › SF8_ZooKeys_key to Neoceratitis/key/SF8_key to Neoceratitis/Media/Images/char6_isolated_158 wing (automontage (c) RMCA).jpg]

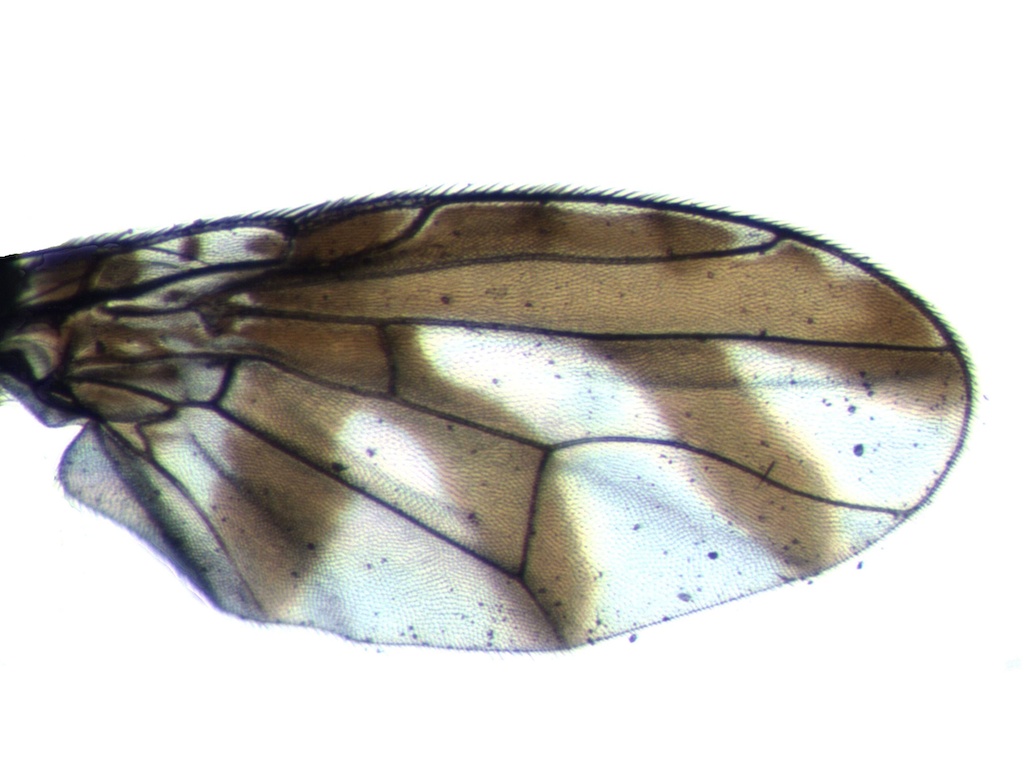

Supplement: Supplementary material 8 — Key to Neoceratitis [file zookeys-428-097-s008.zip › SF8_ZooKeys_key to Neoceratitis/key/SF8_key to Neoceratitis/Media/Images/char6_isolated_429 wing (automontage (c) RMCA).jpg]

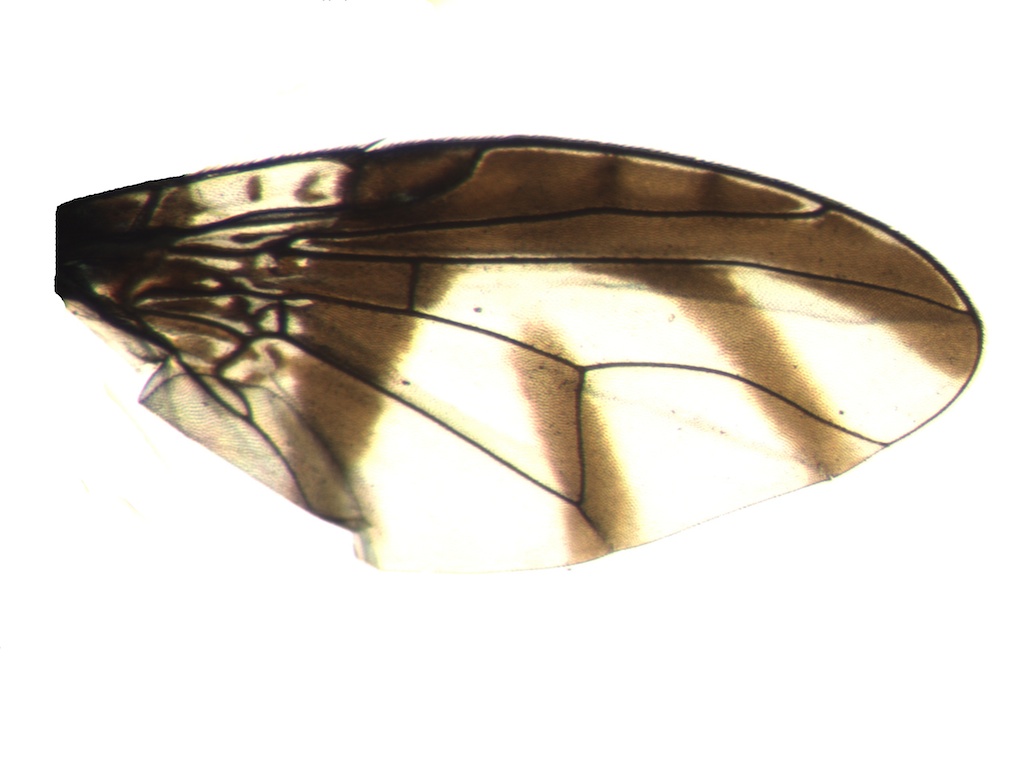

Supplement: Supplementary material 8 — Key to Neoceratitis [file zookeys-428-097-s008.zip › SF8_ZooKeys_key to Neoceratitis/key/SF8_key to Neoceratitis/Media/Images/char6_isolated_431 wing (automontage (c) RMCA).jpg]

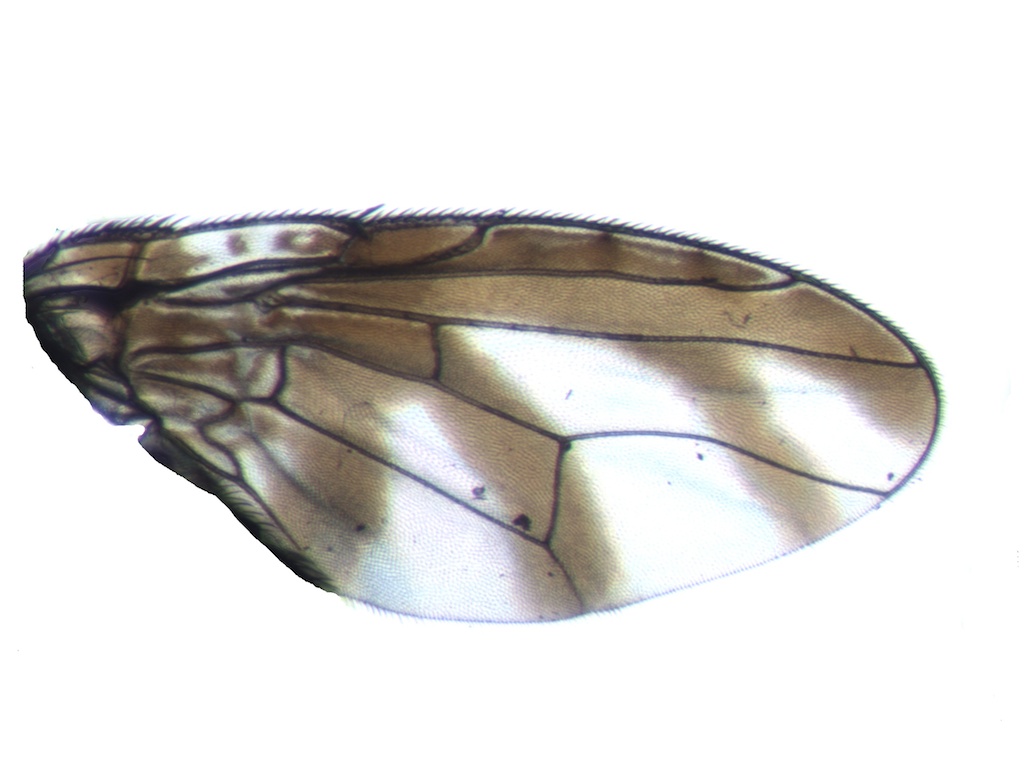

Supplement: Supplementary material 8 — Key to Neoceratitis [file zookeys-428-097-s008.zip › SF8_ZooKeys_key to Neoceratitis/key/SF8_key to Neoceratitis/Media/Images/char6_isolated_432 wing (automontage (c) RMCA).jpg]

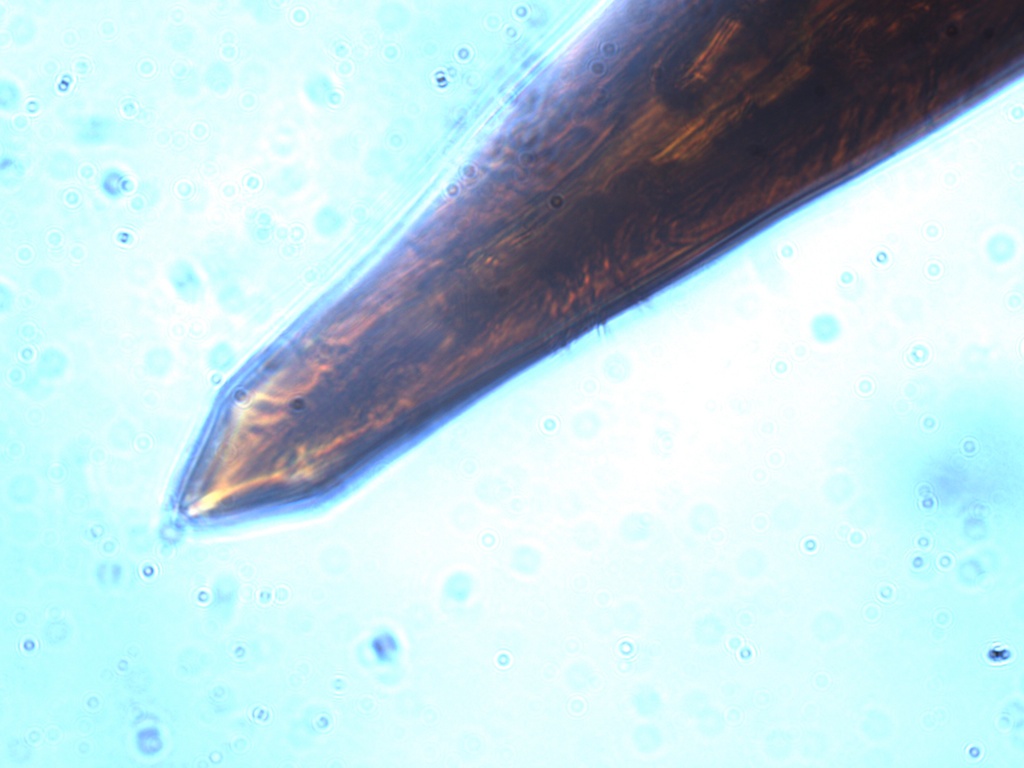

Supplement: Supplementary material 8 — Key to Neoceratitis [file zookeys-428-097-s008.zip › SF8_ZooKeys_key to Neoceratitis/key/SF8_key to Neoceratitis/Media/Images/char9_SAstep_158 aculeus tip (automontage (c) RMCA).jpg]

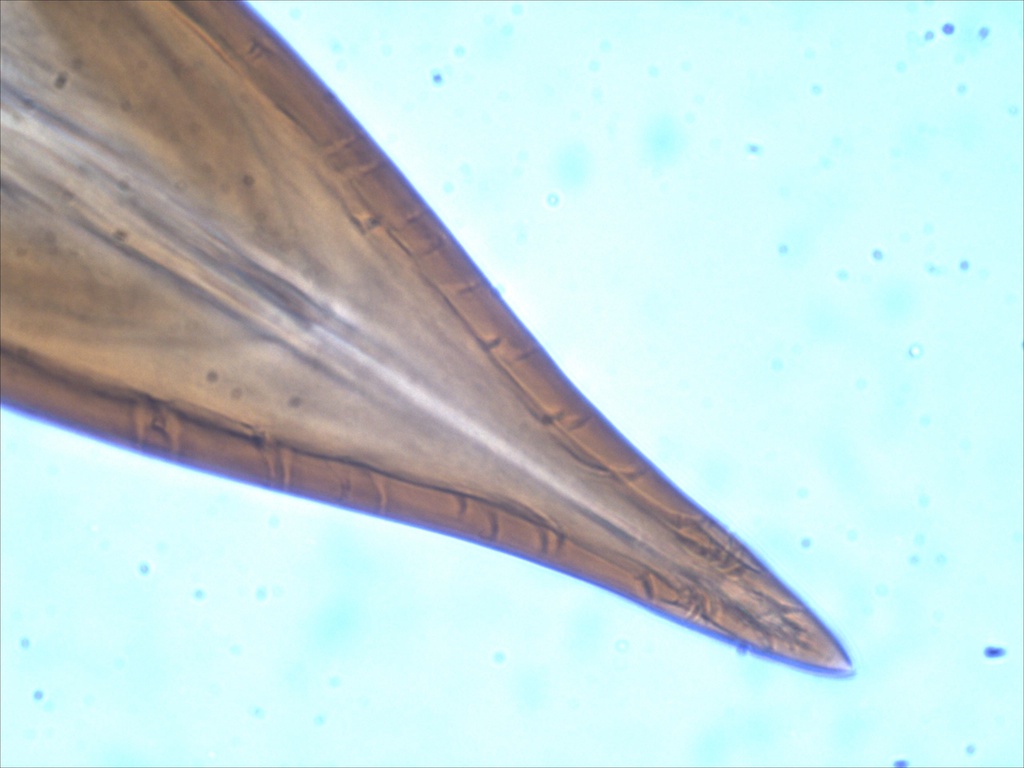

Supplement: Supplementary material 8 — Key to Neoceratitis [file zookeys-428-097-s008.zip › SF8_ZooKeys_key to Neoceratitis/key/SF8_key to Neoceratitis/Media/Images/char9_simple_429 aculeus tip (automontage (c) RMCA).jpg]

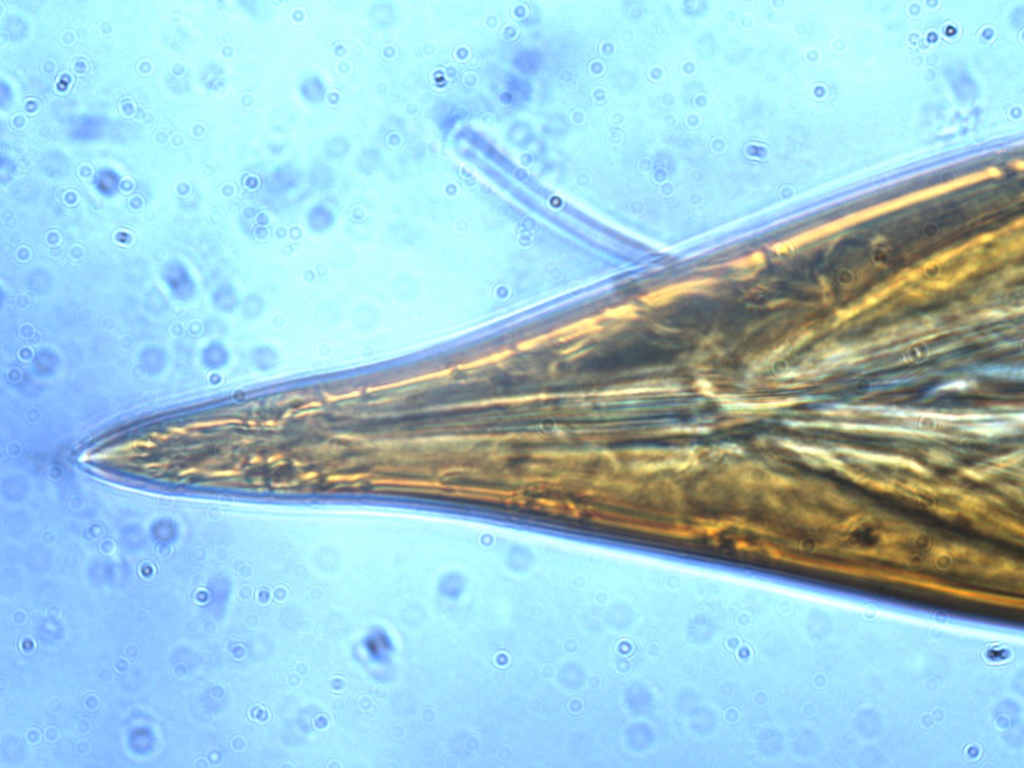

Supplement: Supplementary material 8 — Key to Neoceratitis [file zookeys-428-097-s008.zip › SF8_ZooKeys_key to Neoceratitis/key/SF8_key to Neoceratitis/Media/Images/char9_simple_430 aculeus tip (automontage (c) RMCA).jpg]

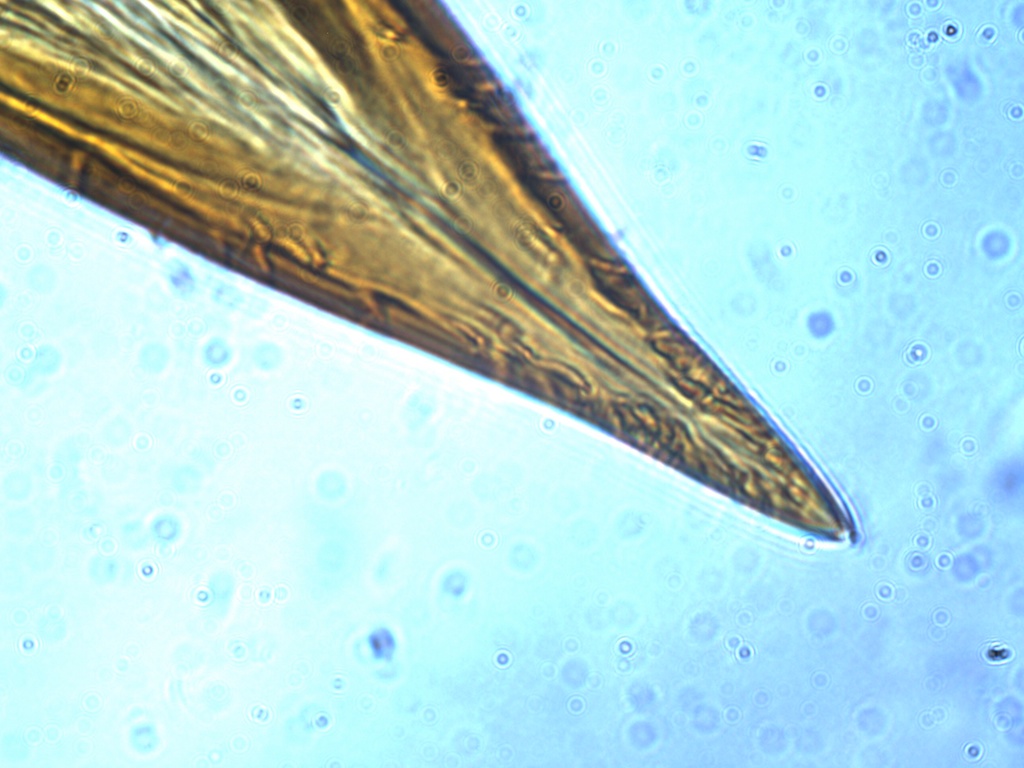

Supplement: Supplementary material 8 — Key to Neoceratitis [file zookeys-428-097-s008.zip › SF8_ZooKeys_key to Neoceratitis/key/SF8_key to Neoceratitis/Media/Images/char9_simple_432 aculeus tip (automontage (c) RMCA).jpg]

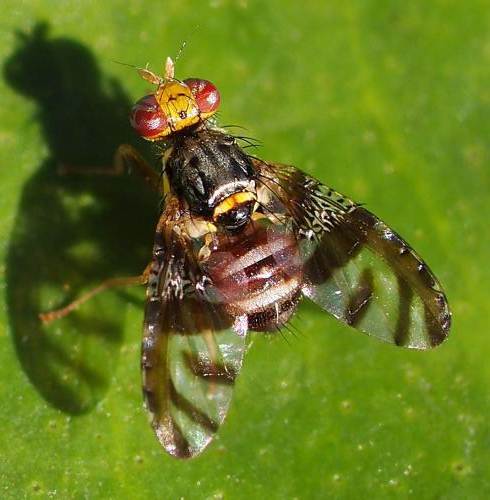

Supplement: Supplementary material 8 — Key to Neoceratitis [file zookeys-428-097-s008.zip › SF8_ZooKeys_key to Neoceratitis/key/SF8_key to Neoceratitis/Media/Images/habitus_Neoceratitis_cyanescens_copyright Dominique Martire.jpg]

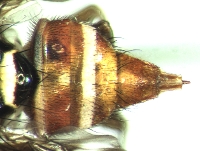

Supplement: Supplementary material 8 — Key to Neoceratitis [file zookeys-428-097-s008.zip › SF8_ZooKeys_key to Neoceratitis/key/SF8_key to Neoceratitis/Media/Thumbs/158 abdomen female dorsal (automontage (c) RMCA)_TN.jpg]

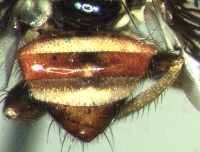

Supplement: Supplementary material 8 — Key to Neoceratitis [file zookeys-428-097-s008.zip › SF8_ZooKeys_key to Neoceratitis/key/SF8_key to Neoceratitis/Media/Thumbs/158 abdomen male dorsal (automontage (c) RMCA)_TN.jpg]

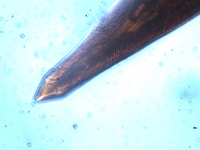

Supplement: Supplementary material 8 — Key to Neoceratitis [file zookeys-428-097-s008.zip › SF8_ZooKeys_key to Neoceratitis/key/SF8_key to Neoceratitis/Media/Thumbs/158 aculeus tip (automontage (c) RMCA)_TN.jpg]

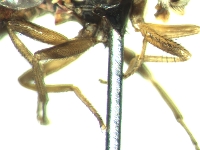

Supplement: Supplementary material 8 — Key to Neoceratitis [file zookeys-428-097-s008.zip › SF8_ZooKeys_key to Neoceratitis/key/SF8_key to Neoceratitis/Media/Thumbs/158 legs female lateral (automontage (c) RMCA)_TN.jpg]

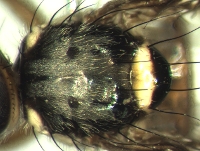

Supplement: Supplementary material 8 — Key to Neoceratitis [file zookeys-428-097-s008.zip › SF8_ZooKeys_key to Neoceratitis/key/SF8_key to Neoceratitis/Media/Thumbs/158 mesonotum (automontage (c) RMCA)_TN.jpg]

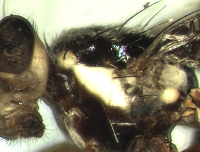

Supplement: Supplementary material 8 — Key to Neoceratitis [file zookeys-428-097-s008.zip › SF8_ZooKeys_key to Neoceratitis/key/SF8_key to Neoceratitis/Media/Thumbs/158 thorax lateral (automontage (c) RMCA)_TN.jpg]

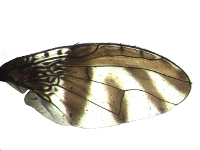

Supplement: Supplementary material 8 — Key to Neoceratitis [file zookeys-428-097-s008.zip › SF8_ZooKeys_key to Neoceratitis/key/SF8_key to Neoceratitis/Media/Thumbs/158 wing (automontage (c) RMCA)_TN.jpg]

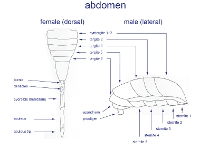

Supplement: Supplementary material 8 — Key to Neoceratitis [file zookeys-428-097-s008.zip › SF8_ZooKeys_key to Neoceratitis/key/SF8_key to Neoceratitis/Media/Thumbs/2_abdomen_M_F_TN.jpg]

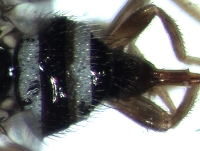

Supplement: Supplementary material 8 — Key to Neoceratitis [file zookeys-428-097-s008.zip › SF8_ZooKeys_key to Neoceratitis/key/SF8_key to Neoceratitis/Media/Thumbs/429 abdomen female dorsal (automontage (c) RMCA)_TN.jpg]

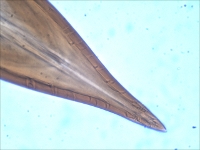

Supplement: Supplementary material 8 — Key to Neoceratitis [file zookeys-428-097-s008.zip › SF8_ZooKeys_key to Neoceratitis/key/SF8_key to Neoceratitis/Media/Thumbs/429 aculeus tip (automontage (c) RMCA)_TN.jpg]

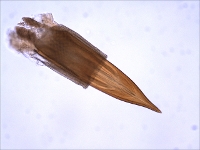

Supplement: Supplementary material 8 — Key to Neoceratitis [file zookeys-428-097-s008.zip › SF8_ZooKeys_key to Neoceratitis/key/SF8_key to Neoceratitis/Media/Thumbs/429 aculeus(automontage (c) RMCA)_TN.jpg]

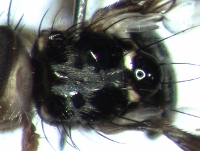

Supplement: Supplementary material 8 — Key to Neoceratitis [file zookeys-428-097-s008.zip › SF8_ZooKeys_key to Neoceratitis/key/SF8_key to Neoceratitis/Media/Thumbs/429 mesonotum (automontage (c) RMCA)_TN.jpg]

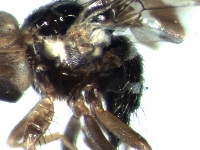

Supplement: Supplementary material 8 — Key to Neoceratitis [file zookeys-428-097-s008.zip › SF8_ZooKeys_key to Neoceratitis/key/SF8_key to Neoceratitis/Media/Thumbs/429 thorax lateral (automontage (c) RMCA)_TN.jpg]

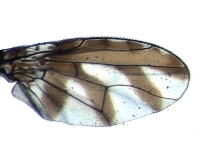

Supplement: Supplementary material 8 — Key to Neoceratitis [file zookeys-428-097-s008.zip › SF8_ZooKeys_key to Neoceratitis/key/SF8_key to Neoceratitis/Media/Thumbs/429 wing (automontage (c) RMCA)_TN.jpg]

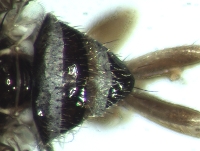

Supplement: Supplementary material 8 — Key to Neoceratitis [file zookeys-428-097-s008.zip › SF8_ZooKeys_key to Neoceratitis/key/SF8_key to Neoceratitis/Media/Thumbs/430 abdomen male dorsal (automontage (c) RMCA)_TN.jpg]

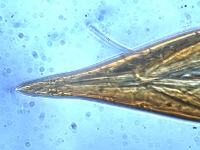

Supplement: Supplementary material 8 — Key to Neoceratitis [file zookeys-428-097-s008.zip › SF8_ZooKeys_key to Neoceratitis/key/SF8_key to Neoceratitis/Media/Thumbs/430 aculeus tip (automontage (c) RMCA)_TN.jpg]

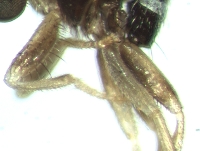

Supplement: Supplementary material 8 — Key to Neoceratitis [file zookeys-428-097-s008.zip › SF8_ZooKeys_key to Neoceratitis/key/SF8_key to Neoceratitis/Media/Thumbs/430 legs male lateral (automontage (c) RMCA)_TN.jpg]

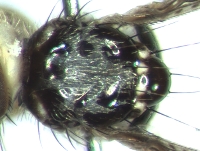

Supplement: Supplementary material 8 — Key to Neoceratitis [file zookeys-428-097-s008.zip › SF8_ZooKeys_key to Neoceratitis/key/SF8_key to Neoceratitis/Media/Thumbs/430 mesonotum (automontage (c) RMCA)_TN.jpg]

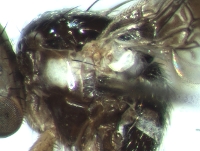

Supplement: Supplementary material 8 — Key to Neoceratitis [file zookeys-428-097-s008.zip › SF8_ZooKeys_key to Neoceratitis/key/SF8_key to Neoceratitis/Media/Thumbs/430 thorax lateral (automontage (c) RMCA)_TN.jpg]

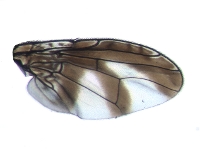

Supplement: Supplementary material 8 — Key to Neoceratitis [file zookeys-428-097-s008.zip › SF8_ZooKeys_key to Neoceratitis/key/SF8_key to Neoceratitis/Media/Thumbs/430 wing (automontage (c) RMCA)_TN.jpg]

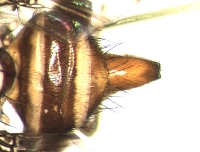

Supplement: Supplementary material 8 — Key to Neoceratitis [file zookeys-428-097-s008.zip › SF8_ZooKeys_key to Neoceratitis/key/SF8_key to Neoceratitis/Media/Thumbs/431 abdomen female dorsal (automontage (c) RMCA)_TN.jpg]

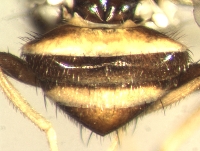

Supplement: Supplementary material 8 — Key to Neoceratitis [file zookeys-428-097-s008.zip › SF8_ZooKeys_key to Neoceratitis/key/SF8_key to Neoceratitis/Media/Thumbs/431 abdomen male dorsal (automontage (c) RMCA)_TN.jpg]

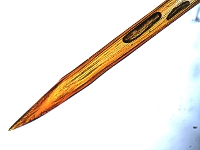

Supplement: Supplementary material 8 — Key to Neoceratitis [file zookeys-428-097-s008.zip › SF8_ZooKeys_key to Neoceratitis/key/SF8_key to Neoceratitis/Media/Thumbs/431 aculeus(automontage (c) RMCA)_TN.jpg]

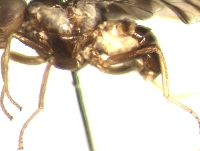

Supplement: Supplementary material 8 — Key to Neoceratitis [file zookeys-428-097-s008.zip › SF8_ZooKeys_key to Neoceratitis/key/SF8_key to Neoceratitis/Media/Thumbs/431 legs male lateral (automontage (c) RMCA)_TN.jpg]

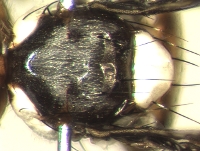

Supplement: Supplementary material 8 — Key to Neoceratitis [file zookeys-428-097-s008.zip › SF8_ZooKeys_key to Neoceratitis/key/SF8_key to Neoceratitis/Media/Thumbs/431 mesonotum (automontage (c) RMCA)_TN.jpg]

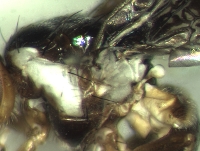

Supplement: Supplementary material 8 — Key to Neoceratitis [file zookeys-428-097-s008.zip › SF8_ZooKeys_key to Neoceratitis/key/SF8_key to Neoceratitis/Media/Thumbs/431 thorax lateral (automontage (c) RMCA)_TN.jpg]

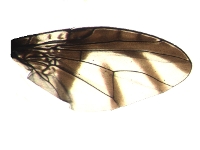

Supplement: Supplementary material 8 — Key to Neoceratitis [file zookeys-428-097-s008.zip › SF8_ZooKeys_key to Neoceratitis/key/SF8_key to Neoceratitis/Media/Thumbs/431 wing (automontage (c) RMCA)_TN.jpg]

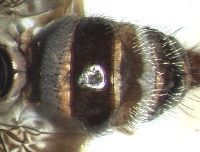

Supplement: Supplementary material 8 — Key to Neoceratitis [file zookeys-428-097-s008.zip › SF8_ZooKeys_key to Neoceratitis/key/SF8_key to Neoceratitis/Media/Thumbs/432 abdomen female dorsal (automontage (c) RMCA)_TN.jpg]

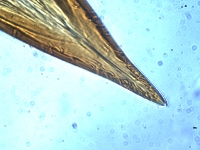

Supplement: Supplementary material 8 — Key to Neoceratitis [file zookeys-428-097-s008.zip › SF8_ZooKeys_key to Neoceratitis/key/SF8_key to Neoceratitis/Media/Thumbs/432 aculeus tip (automontage (c) RMCA)_TN.jpg]

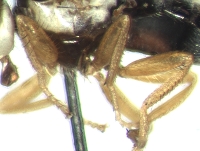

Supplement: Supplementary material 8 — Key to Neoceratitis [file zookeys-428-097-s008.zip › SF8_ZooKeys_key to Neoceratitis/key/SF8_key to Neoceratitis/Media/Thumbs/432 legs lateral (automontage (c) RMCA)_TN.jpg]

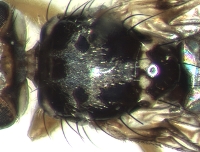

Supplement: Supplementary material 8 — Key to Neoceratitis [file zookeys-428-097-s008.zip › SF8_ZooKeys_key to Neoceratitis/key/SF8_key to Neoceratitis/Media/Thumbs/432 mesonotum (automontage (c) RMCA)_TN.jpg]

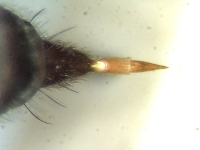

Supplement: Supplementary material 8 — Key to Neoceratitis [file zookeys-428-097-s008.zip › SF8_ZooKeys_key to Neoceratitis/key/SF8_key to Neoceratitis/Media/Thumbs/432 oviscape and aculeus dorsal (automontage (c) RMCA)_TN.jpg]

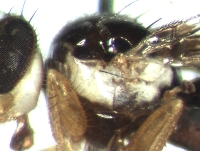

Supplement: Supplementary material 8 — Key to Neoceratitis [file zookeys-428-097-s008.zip › SF8_ZooKeys_key to Neoceratitis/key/SF8_key to Neoceratitis/Media/Thumbs/432 thorax lateral (automontage (c) RMCA)_TN.jpg]

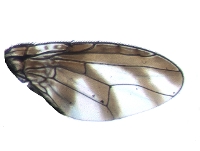

Supplement: Supplementary material 8 — Key to Neoceratitis [file zookeys-428-097-s008.zip › SF8_ZooKeys_key to Neoceratitis/key/SF8_key to Neoceratitis/Media/Thumbs/432 wing (automontage (c) RMCA)_TN.jpg]

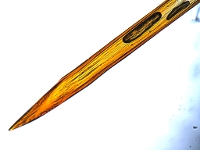

Supplement: Supplementary material 8 — Key to Neoceratitis [file zookeys-428-097-s008.zip › SF8_ZooKeys_key to Neoceratitis/key/SF8_key to Neoceratitis/Media/Thumbs/char10_10x_431 aculeus(automontage (c) RMCA)_TN.jpg]

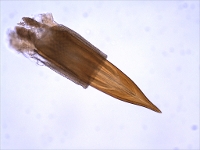

Supplement: Supplementary material 8 — Key to Neoceratitis [file zookeys-428-097-s008.zip › SF8_ZooKeys_key to Neoceratitis/key/SF8_key to Neoceratitis/Media/Thumbs/char10_4_6x_429 aculeus(automontage (c) RMCA)_TN.jpg]

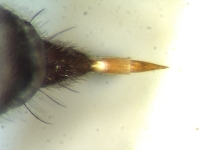

Supplement: Supplementary material 8 — Key to Neoceratitis [file zookeys-428-097-s008.zip › SF8_ZooKeys_key to Neoceratitis/key/SF8_key to Neoceratitis/Media/Thumbs/char10_4_6x_432 oviscape and aculeus dorsal (automontage (c) RMCA)_TN.jpg]

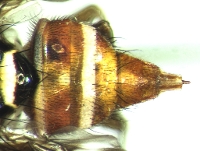

Supplement: Supplementary material 8 — Key to Neoceratitis [file zookeys-428-097-s008.zip › SF8_ZooKeys_key to Neoceratitis/key/SF8_key to Neoceratitis/Media/Thumbs/char11_female_158 abdomen female dorsal (automontage (c) RMCA)_TN.jpg]

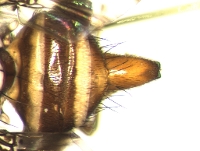

Supplement: Supplementary material 8 — Key to Neoceratitis [file zookeys-428-097-s008.zip › SF8_ZooKeys_key to Neoceratitis/key/SF8_key to Neoceratitis/Media/Thumbs/char11_female_431 abdomen female dorsal (automontage (c) RMCA)_TN.jpg]

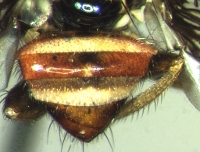

Supplement: Supplementary material 8 — Key to Neoceratitis [file zookeys-428-097-s008.zip › SF8_ZooKeys_key to Neoceratitis/key/SF8_key to Neoceratitis/Media/Thumbs/char11_male_158 abdomen male dorsal (automontage (c) RMCA)_TN.jpg]

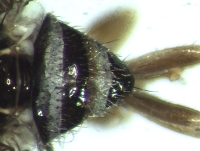

Supplement: Supplementary material 8 — Key to Neoceratitis [file zookeys-428-097-s008.zip › SF8_ZooKeys_key to Neoceratitis/key/SF8_key to Neoceratitis/Media/Thumbs/char11_male_430 abdomen male dorsal (automontage (c) RMCA)_TN.jpg]

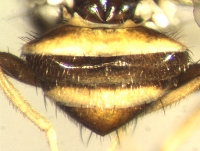

Supplement: Supplementary material 8 — Key to Neoceratitis [file zookeys-428-097-s008.zip › SF8_ZooKeys_key to Neoceratitis/key/SF8_key to Neoceratitis/Media/Thumbs/char11_male_431 abdomen male dorsal (automontage (c) RMCA)_TN.jpg]

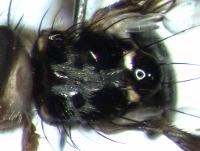

Supplement: Supplementary material 8 — Key to Neoceratitis [file zookeys-428-097-s008.zip › SF8_ZooKeys_key to Neoceratitis/key/SF8_key to Neoceratitis/Media/Thumbs/char1_dark_429 mesonotum (automontage (c) RMCA)_TN.jpg]

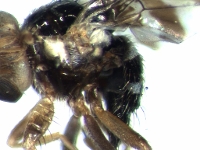

Supplement: Supplementary material 8 — Key to Neoceratitis [file zookeys-428-097-s008.zip › SF8_ZooKeys_key to Neoceratitis/key/SF8_key to Neoceratitis/Media/Thumbs/char1_dark_429 thorax lateral (automontage (c) RMCA)_TN.jpg]

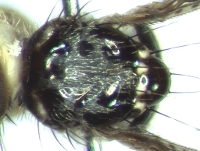

Supplement: Supplementary material 8 — Key to Neoceratitis [file zookeys-428-097-s008.zip › SF8_ZooKeys_key to Neoceratitis/key/SF8_key to Neoceratitis/Media/Thumbs/char1_dark_430 mesonotum (automontage (c) RMCA)_TN.jpg]

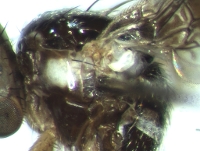

Supplement: Supplementary material 8 — Key to Neoceratitis [file zookeys-428-097-s008.zip › SF8_ZooKeys_key to Neoceratitis/key/SF8_key to Neoceratitis/Media/Thumbs/char1_dark_430 thorax lateral (automontage (c) RMCA)_TN.jpg]

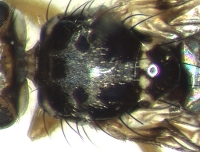

Supplement: Supplementary material 8 — Key to Neoceratitis [file zookeys-428-097-s008.zip › SF8_ZooKeys_key to Neoceratitis/key/SF8_key to Neoceratitis/Media/Thumbs/char1_dark_432 mesonotum (automontage (c) RMCA)_TN.jpg]

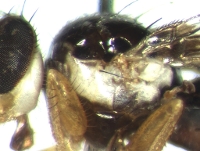

Supplement: Supplementary material 8 — Key to Neoceratitis [file zookeys-428-097-s008.zip › SF8_ZooKeys_key to Neoceratitis/key/SF8_key to Neoceratitis/Media/Thumbs/char1_dark_432 thorax lateral (automontage (c) RMCA)_TN.jpg]
