# Supplementary material for: A set of multi-entry identification keys to African frugivorous flies (Diptera, Tephritidae)
Source: Zookeys. 2014 Jul 24;(428):97–108. doi: 10.3897/zookeys.428.7366 (PMC4143993; doi:10.3897/zookeys.428.7366)
Supplement: Supplementary material 9 — Key to Perilampsis [file zookeys-428-097-s009.zip › SF9_ZooKeys_key to Perilampsis/key/SF9_key to Perilampsis/Media/Html/desc_Perilampsis_curta.html]

Natural Language Description


# A set of multi-entry identification keys to African frugivorous flies (Diptera, Tephritidae)

### Massimiliano Virgilio, Ian White, Marc De Meyer

## Perilampsis curta

(key to Perilampsis) sex male or female. (key to Perilampsis) head (key to Perilampsis) 2. (males) arista pubescent. (key to Perilampsis) thorax (key to Perilampsis) 3. katatergite dark, (key to Perilampsis) 4. anatergite dark, (key to Perilampsis) 5. one transverse band of silvery pilosity and microtrichosity anteriorly of transverse suture present, (key to Perilampsis) 7. scutellum completely white. (key to Perilampsis) legs (key to Perilampsis) 6. femora brown. (key to Perilampsis) wings (key to Perilampsis) 9. cell C brown 50%, (key to Perilampsis) 10. anterior apical band narrow, (key to Perilampsis) 12. anterior apical band (2) continuous till wing apex (at most with incomplete interruption near vein R2+3 apex, if the latter, no apical tooth), (key to Perilampsis) 13. discal band connected to anterior apical band, (key to Perilampsis) 14. posterior apical band (1) present, (key to Perilampsis) 15. posterior apical band (2) merged with anterior apical band, (key to Perilampsis) 16. subapical band (1) present, (key to Perilampsis) 17. subapical band (2) separated from discal band, (key to Perilampsis) 18. subbasal band merged with discal band, (key to Perilampsis) 19. crossvein R-M intersecting the first half of vein M. (key to Perilampsis) abdomen (key to Perilampsis) 20. abdominal tergites largely shining orange-red to red or brown-black, (key to Perilampsis) 21. abdominal tergite 4 shining brown-black, in posterior half at most with greyish band, (key to Perilampsis) 22. (females) oviscape longer than abdominal tergites 1–5 combined.
