# Supplementary material for: A set of multi-entry identification keys to African frugivorous flies (Diptera, Tephritidae)
Source: Zookeys. 2014 Jul 24;(428):97–108. doi: 10.3897/zookeys.428.7366 (PMC4143993; doi:10.3897/zookeys.428.7366)
Supplement: Supplementary material 9 — Key to Perilampsis [file zookeys-428-097-s009.zip › SF9_ZooKeys_key to Perilampsis/key/SF9_key to Perilampsis/Media/Html/Perilampsis amazuluana.htm]

Perilampsis amazuluana Munro


***Perilampsis*** ***amazuluana* Munro**

*Perilampsis* *amazuluana* Munro, 1929: 394.

 

Body length. 4.00-4.60 mm; wing length 4.00-4.80 mm.

 

Male

Head: Antennal segments yellow-orange. Arista bare. Frons
ventral part yellow-white, dorsal part yellow-orange. Two frontals, placed
parallel to medial eye margin; two orbitals, placed slightly convergent with
inner orbital more medially. Face white. Occiput pale yellow in dorsal fourth,
with two darker patches; otherwise white.

Thorax: Scutum shining black-brown, with dark pilosity;
centre with silver-grey pilosity and microtrichosity but no
distinct transverse bands. Postpronotum white. Anepisternum pale yellow, with
white band occupying posterodorsal part, its ventral margin reaching posteroventral
corner; with pale pilosity; one anepisternal seta. Anatergite and katatergite
white. Scutellum white. Subscutellum brown.

Legs: pale yellow.

Wing: Wing bands brown, well developed but with
diffused margins. Basal part wing brownish coloured, subbasal irregular dark
spots or streaks present, no distinct subbasal band. Anterior apical band
covering cells r1 and r2+3 completely, largely merged
with posterior apical band. Subapical band touching anterior apical band or
almost so. Discal band and anterior apical band united at pterostigma. Discal
band not reaching posterior wing margin, basally confluent with subbasal
streaks and spots. R-M ratio 0.82-0.85.

Abdomen: Shining brown, tergite 1 and posterior
three-fourths of tergites 2 and 4 with greyish band; tergite 5 with posteromedian
part yellow.

 

Female

As male except for the following characters: arista
very short pubescent; frons darker orange; femora, especially front and mid
femur, darker yellow. Wing with distinctly separated anterior and posterior
apical bands and subapical band. Posterior apical band touching anterior apical
band only at base; subapical band isolated. Female terminalia, oviscape shorter
than abdominal tergites, shining brown, with black pilosity. Aculeus orange, flattened, about 8 times as long as
wide; aculeus tip slightly narrowed, probably pointed.

 

(Description after De Meyer,
2009)
