# Supplementary material for: A set of multi-entry identification keys to African frugivorous flies (Diptera, Tephritidae)
Source: Zookeys. 2014 Jul 24;(428):97–108. doi: 10.3897/zookeys.428.7366 (PMC4143993; doi:10.3897/zookeys.428.7366)
Supplement: Supplementary material 9 — Key to Perilampsis [file zookeys-428-097-s009.zip › SF9_ZooKeys_key to Perilampsis/key/SF9_key to Perilampsis/Media/Html/Perilampsis atra.htm]

Perilampsis atra Munro


***Perilampsis atra*** Munro

*Perilampsis atra* Munro, 1969: 433

Body length. 3.20-4.80 mm; wing length 3.25-4.45 mm.

 

Male

Head: Antennal segments dark brown. Arista pubescent,
longest rays at most twice the width of base of arista. Frons ventral half
yellow-white, dorsal part with transverse brown band along width of orbital
bristles, area above dorsal orbital till occiput yellow-white. Two frontals,
placed parallel to medial eye margin; two orbitals, placed slightly convergent
with inner orbital more medially. Face white, dorsal third dark brown, median
part sometimes with two brown patches or transverse band. Occiput black-brown,
only margins white.

Thorax: Scutum shining black-brown, more yellowish
brown near transverse suture; dark dispersed pilosity, one broad transverse
band with silvery pilosity and microtrichosity, anteriorly of transverse
suture. Postpronotum white. Anepisternum brown, with white band occupying
posterodorsal part, its ventral margin reaching posteroventral corner or almost
so; with pale pilosity except in posteroventral corner with few dark setulae;
one anepisternal seta. Anatergite and katatergite white. Scutellum white. Subscutellum
brown.

Legs: pale yellow, femora and anterior fifth of hind
tibiae black-brown.

Wing: Wing bands brown, largely reduced. No basal
spots or streaks, except for subbasal band. Anterior apical band covering cell
r1 completely except for hyaline spot in apical end of cell;
covering cell r2+3 only partially; with subapical tooth into cell r4+5.
Posterior apical band absent. Area between subbasal band and discal band
hyaline. Discal band and anterior apical band united at pterostigma. Discal
band reaching posterior wing margin. R-M ratio 0.48-0.55.

Abdomen: Shining black-brown, posterior margin of
tergite 2 with narrow greyish band, tergite 5 with small yellow
patch posteromedially.

 

Female

As male, except for the following characters:
pubescence of arista slightly longer; mid and hind tibiae dark on anterior half
or two-thirds. Female terminalia, oviscape about two-thirds of length of
abdominal tergites, shining black-brown, with black pilosity. Aculeus orange,
flattened, about 7 times as long as broad, apex� strongly narrowed, pointed
tip.

 

(Description after
De Meyer, 2009)
