# Supplementary material for: A set of multi-entry identification keys to African frugivorous flies (Diptera, Tephritidae)
Source: Zookeys. 2014 Jul 24;(428):97–108. doi: 10.3897/zookeys.428.7366 (PMC4143993; doi:10.3897/zookeys.428.7366)
Supplement: Supplementary material 9 — Key to Perilampsis [file zookeys-428-097-s009.zip › SF9_ZooKeys_key to Perilampsis/key/SF9_key to Perilampsis/Media/Html/Perilampsis curta.htm]

Perilampsis curta Munro


***Perilampsis curta*** Munro

*Perilampsis* *curta* Munro, 1938: 165.

 

Body length. 3.80-4.40 mm; wing length 4.20-4.90 mm.

 

Male

Head: Antennal segments orange-brown. Arista short
pubescent, longest rays at most equal to width of base of arista. Frons ventral
half yellow-white, dorsal part orange brown. Two frontals, placed parallel to
medial eye margin; two orbitals, placed slightly convergent with inner orbital
more medially. Face white, with dark transverse band near antennal implant.
Occiput yellow, with pair of distinct black patches in dorsal part.

Thorax: Scutum shining brown; dark dispersed
pilosity, two transverse bands with more dense silvery pilosity and
microtrichosity, one anteriorly of transverse suture, second near
dorsocentrals. Postpronotum white. Anepisternum brown, with white band
occupying posterodorsal part, its ventral margin reaching posteroventral corner
or almost so; with pale pilosity; one anepisternal seta. Anatergite and
katatergite brown. Scutellum white. Subscutellum brown.

Legs: yellow, femora darker yellow to brown.

Wing: Wing bands brown, well developed. Basal part of
wing brown, subbasal irregular spots and streaks present. Anterior apical band
completely filling cells r1 and r2+3. Posterior apical
band touching anterior apical band. Subapical band isolated. Discal band not
reaching posterior wing margin; touching anterior apical band near pterostigma;
largely merged with subbasal spots and streaks. R-M ratio 0.88-1.0.

Abdomen: Shining black-brown (orange-brown in
holotype), posterior margin of tergites 2-4 with orange-brown and silvery band;
tergite 5 completely yellow-brown.

 

Female

As male except for following characters: arista
slightly longer pubescent. Abdomen darker coloured. Legs with knees more
distinctly darkened. Female terminalia, oviscape shorter than abdominal
tergites, black to black-brown colour, with black pilosity. Aculeus orange, flattened, about 12 times as long as
wide; aculeus tip slightly sinuate, pointed.

 

(Description after De Meyer,
2009)
