# Supplementary material for: A set of multi-entry identification keys to African frugivorous flies (Diptera, Tephritidae)
Source: Zookeys. 2014 Jul 24;(428):97–108. doi: 10.3897/zookeys.428.7366 (PMC4143993; doi:10.3897/zookeys.428.7366)
Supplement: Supplementary material 9 — Key to Perilampsis [file zookeys-428-097-s009.zip › SF9_ZooKeys_key to Perilampsis/key/SF9_key to Perilampsis/Media/Html/Perilampsis decellei.htm]

Perilampsis decellei Munro


***Perilampsis decellei*** Munro

*Perilampsis decellei* Munro, 1969: 430.

 

Body length. 3.90-4.55 mm; wing length 3.85-4.50 mm.

 

Male

Head: Antennal segments brown. Arista short
pubescent, longest rays at most equal to width of base of arista. Frons ventral
half yellow-white, dorsal part darker, near ventral orbital with brown
transverse band. Two frontals, placed parallel to medial eye margin; two
orbitals, placed slightly convergent with inner orbital more medially. Face
yellow-white, antennal groove with darker spot, occassionaly with transverse
band. Occiput largely dark, along margins yellow.

Thorax: Scutum shining brown; dark dispersed
pilosity, one transverse band with silvery pilosity and microtrichosity,
anteriorly of transverse suture. Postpronotum white. Anepisternum brown, with
white band occupying posterodorsal part with ventral margin reaching
posteroventral corner or almost so; with pale pilosity; one anepisternal seta.
Anatergite and katatergite white. Scutellum white. Subscutellum brown.

Legs: pale yellow, femora and knees brown.

Wing: Wing bands brown, well developed. Basal part of
wing brown, subbasal irregular spots and streaks present. Anterior apical band
completely filling cells r1 and r2+3. Posterior apical
band touching anterior apical band. Subapical band isolated. Discal band not
reaching posterior wing margin; touching anterior apical band near pterostigma;
largely merged with subbasal spots and streaks. R-M ratio 1-1.33.

Abdomen: Shining dark black-brown, anterior margin of
tergite 1 yellowish; posterior margin of tergites 2 and 4 with greyish band,
anteriorly more yellow; tergite 5 with median yellow patch.

 

Female

As male except for following characters: median
yellow patch in tergite 5 reaching anterior margin of tergite. Female terminalia,
oviscape at most as long as abdominal tergites, black to black-brown colour,
with black pilosity. Aculeus flattened and
broad, about 6 times longer than wide; aculeus tip broad with serrated edge.

 

(Description after De Meyer,
2009)
