# Supplementary material for: A set of multi-entry identification keys to African frugivorous flies (Diptera, Tephritidae)
Source: Zookeys. 2014 Jul 24;(428):97–108. doi: 10.3897/zookeys.428.7366 (PMC4143993; doi:10.3897/zookeys.428.7366)
Supplement: Supplementary material 9 — Key to Perilampsis [file zookeys-428-097-s009.zip › SF9_ZooKeys_key to Perilampsis/key/SF9_key to Perilampsis/Media/Html/Perilampsis deemingi.htm]

Perilampsis deemingi De Meyer


***Perilampsis deemingi*** De Meyer

*Perilampsis deemingi* De Meyer, 2009: 2439.

�

 

Body length. 3.60-4.20 mm; wing length 3.30-4.20 mm.

 

Male

Head: Antennal segments dark brown. Arista pubescent,
longest rays at most twice the width of base of arista. Frons ventral half
yellow-white, dorsal part with transverse brown band along width of orbital
bristles, area above dorsal orbital till occiput yellow-white. Two frontals,
placed parallel to medial eye margin; two orbitals, placed slightly convergent
with inner orbital more medially. Face white, dorsal third dark brown. Occiput
black-brown, only margins white.

Thorax: Scutum shining black-brown, more yellowish
brown near transverse suture; dark dispersed pilosity, one broad transverse
band with silvery pilosity and microtrichosity anteriorly of transverse suture.
Postpronotum white. Anepisternum brown, with white band occupying posterodorsal
part, its ventral margin reaching posteroventral corner or almost so; with pale
pilosity except in posteroventral corner with few dark setulae; one
anepisternal seta. Anatergite and katatergite white. Scutellum white.
Subscutellum brown.

Legs: pale yellow, femora and anterior half of mid
and hind tibiae black-brown.

Wing: Wing bands brown, largely reduced. No basal
spots or streaks, except for subbasal band. Anterior apical band covering cell
r1 almost completely; covering cell r2+3 only partially;
without subapical tooth into cell r4+5. Posterior apical band
absent. Subapical band strongly reduced, usually as narrow line, sometimes
almost completely absent. Area between subbasal band and discal band hyaline.
Discal band and anterior apical band united at pterostigma. Discal band
reaching posterior wing margin. R-M ratio 0.42-0.48.

Abdomen: Shining black-brown, posterior margin of tergite
2 with narrow greyish band, tergite 5 with small yellow
patch posteromedially.

 

Female

As male, except for the following characters: mid and
hind tibiae dark on anterior two-thirds. Female terminalia, oviscape about
two-thirds of length of abdominal tergites, shining black-brown, with black
pilosity. Aculeus orange, flattened, about 7 times as long as broad, apex
strongly narrowed, pointed tip.

 

(Description after De Meyer,
2009)
