# Supplementary material for: A set of multi-entry identification keys to African frugivorous flies (Diptera, Tephritidae)
Source: Zookeys. 2014 Jul 24;(428):97–108. doi: 10.3897/zookeys.428.7366 (PMC4143993; doi:10.3897/zookeys.428.7366)
Supplement: Supplementary material 9 — Key to Perilampsis [file zookeys-428-097-s009.zip › SF9_ZooKeys_key to Perilampsis/key/SF9_key to Perilampsis/Media/Html/Perilampsis diademata.htm]

Perilampsis diademata Bezzi


***Perilampsis
diademata*** Bezzi

*Perilampsis
diademata* Bezzi, 1924: 482.

 

Body length. 3.30-4.20
mm; wing length 3.75-4.00 mm.

 

Male

Head: Antennal segments orange-brown. Arista almost
bare, few dispersed rays shorter than width of base of arista. Frons ventral
half yellow-white, dorsal part orange-brown. Two frontals, placed parallel to
medial eye margin; two orbitals, placed slightly convergent with inner orbital
more medially. Face white, at antennal implant with pale brown transverse band.
Occiput yellow, with pair of very faint darker patches.

Thorax: Scutum shining reddish brown to brown,
sometimes paler ground colour with brown patches; dark dispersed pilosity; two
transverse bands with silvery pilosity and microtrichosity, one anteriorly of
transverse suture, second band near dorsocentrals. Postpronotum white. Anepisternum
brown, with white band occupying posterodorsal part, its ventral margin
reaching posteroventral corner; with pale pilosity; one anepisternal seta. Anatergite
and katatergite brown, at most katatergite with pale yellow spot. Scutellum
white, lateral margins yellow-brown coloured to some extent, usually for length
beyond basal scutellar seta but not beyond apical scutellar seta. Subscutellum
brown.

Legs: pale yellow, femora darker yellow.

Wing: Wing bands brown, well developed. Basal part of
wing brown, subbasal irregular spots and streaks present. Anterior apical band
completely filling cells r1 and r2+3. Posterior apical
band touching anterior apical band. Subapical band isolated. Discal band not
reaching posterior wing margin; touching anterior apical band near pterostigma;
largely merged with subbasal spots and streaks. R-M ratio 0.75-0.93.

Abdomen: Shining orange-red to red, posterior fourth
to two-thirds of tergites 2 and 4 with greyish band.

 

Female.

As male except for the following characters:
pubescence of arista slightly longer, equal to width of arista base; femora
darker than in male. Female terminalia, oviscape slightly longer than abdominal
tergites, shining black-brown, with black pilosity. Aculeus orange, flattened,
about 12 times as long as wide; aculeus tip slightly sinuate, pointed.

 

(Description after De Meyer,
2009)
