# Supplementary material for: A set of multi-entry identification keys to African frugivorous flies (Diptera, Tephritidae)
Source: Zookeys. 2014 Jul 24;(428):97–108. doi: 10.3897/zookeys.428.7366 (PMC4143993; doi:10.3897/zookeys.428.7366)
Supplement: Supplementary material 9 — Key to Perilampsis [file zookeys-428-097-s009.zip › SF9_ZooKeys_key to Perilampsis/key/SF9_key to Perilampsis/Media/Html/Perilampsis dryades.htm]

Perilampsis dryades Munro


***Perilampsis dryades*** Munro

*Perilampsis dryades* Munro, 1939: 38.

 

Body length. 3.75-4.45 mm; wing length 3.80-4.30 mm.

 

Male

Head: Antennal segments orange. Arista almost bare,
at most few dispersed rays shorter than width of base of arista. Frons ventral
two-fifths yellow-white, dorsal part orange-brown to brown. Two frontals,
placed parallel to medial eye margin; two orbitals, placed slightly convergent
with inner orbital more medially. Face white; with brown band or brown patches
near antennal implant. Occiput yellow, with two darker, largely confluent,
patches in dorsal part.

Thorax: Scutum shining brown; dark dispersed
pilosity, median part with silver-grey microtrichosity;
two transverse bands with silvery pilosity and microtrichosity, one anteriorly
of transverse suture, second band near dorsocentrals. Postpronotum white.
Anepisternum brown, with white band occupying posterodorsal part, its ventral
margin reaching posteroventral corner or almost so; with pale pilosity; one
anepisternal seta. Anatergite and katatergite white. Scutellum white.
Subscutellum brown.

Legs: pale yellow, femora yellow-brown.

Wing: Wing bands brown, well developed. Basal part of
wing brown, subbasal irregular spots and streaks present. Anterior apical band
completely filling cells r1 and r2+3. Posterior apical
band touching anterior apical band. Subapical band isolated. Discal band
reaching posterior wing margin; touching anterior apical band near pterostigma;
well separated from subbasal spots and streaks, at least in cell cu1. R-M ratio
0.77-0.88.

Abdomen: Shining dark brown, posterior fourth to two-thirds of tergites 2 and 4 with greyish band.

 

Female

As male. Female terminalia, oviscape about as long as
abdominal tergites, shining orange-brown, with black pilosity. Aculeus flattened,
about 10 times longer than wide; apical part pointed with straight sides,
sublaterally somewhat broadened; aculeus tip pointed with subapical shoulders.

 

(Description after De Meyer,
2009)
