# Supplementary material for: A set of multi-entry identification keys to African frugivorous flies (Diptera, Tephritidae)
Source: Zookeys. 2014 Jul 24;(428):97–108. doi: 10.3897/zookeys.428.7366 (PMC4143993; doi:10.3897/zookeys.428.7366)
Supplement: Supplementary material 9 — Key to Perilampsis [file zookeys-428-097-s009.zip › SF9_ZooKeys_key to Perilampsis/key/SF9_key to Perilampsis/Media/Html/Perilampsis formosula.htm]

Perilampsis formosula (Austen)


***Perilampsis formosula*** (Austen)

*Carpophthoromyia formosula* Austen, 1910: 74.

 

Body length. 3.65-4.00 mm; wing length 3.90-4.40 mm.

 

Male

Head: Antennal segments orange brown. Arista
pubescent, longest rays at most three times the width of base of arista. Frons
ventral half yellow-white, dorsal part with transverse brown or yellow-brown
band along width of orbital bristles, area above dorsal orbital till occiput
yellow-white. Two frontals, placed parallel to medial eye margin; two orbitals,
placed slightly convergent with inner orbital more medially. Face white, dorsal
third dark brown. Occiput black-brown, only margins white.

Thorax: Scutum shining black-brown, more yellowish
brown near transverse suture; dark dispersed pilosity, one broad transverse
band with silvery pilosity and microtrichosity anteriorly of transverse suture.
Postpronotum white. Anepisternum brown, with white band occupying posterodorsal
part, its ventral margin reaching posteroventral corner or almost so; with pale
pilosity except in posteroventral corner with few dark setulae; one
anepisternal seta. Anatergite and katatergite white. Scutellum white. Subscutellum
brown.

Legs: pale yellow, femora and anterior two-thirds of
mid and hind tibiae black-brown.

Wing: Wing bands brown, largely reduced. No basal
spots or streaks, except for subbasal band. Anterior apical band covering cell
r1 completely except for hyaline interruption in apical end of cell;
covering cell r2+3 only partially; with subapical tooth into cell r4+5.
Posterior apical band absent. Area between subbasal band and discal band
hyaline. Discal band and anterior apical band united at pterostigma. Discal
band reaching posterior wing margin. R-M ratio 0.5.

Abdomen: Shining black-brown, posterior margin of
tergite 2 with narrow greyish band, tergite 5 with small yellow
patch posteromedially.

 

Female

As male. Female terminalia, oviscape about two-thirds
length of abdominal tergites, shining black-brown, with black pilosity. Aculeus
orange, flattened, about 7 times as long as broad, apex strongly narrowed,
pointed tip.

 

(Description after
De Meyer, 2009)
