# Supplementary material for: A set of multi-entry identification keys to African frugivorous flies (Diptera, Tephritidae)
Source: Zookeys. 2014 Jul 24;(428):97–108. doi: 10.3897/zookeys.428.7366 (PMC4143993; doi:10.3897/zookeys.428.7366)
Supplement: Supplementary material 9 — Key to Perilampsis [file zookeys-428-097-s009.zip › SF9_ZooKeys_key to Perilampsis/key/SF9_key to Perilampsis/Media/Html/Perilampsis furcata.htm]

Perilampsis furcata Munro


***Perilampsis furcata*** Munro

*Perilampsis furcata* Munro, 1969: 431.

Body length. 3.40-4.50 mm; wing length 3.85-4.85 mm.

 

Male

Head: Antennal segments brown. Arista short
pubescent, longest rays at most equal to width of base of arista. Frons ventral
half yellow-white, dorsal part darker yellow, near ventral orbital with brown
transverse band. Two frontals, placed parallel to medial eye margin; two orbitals,
placed slightly convergent with inner orbital more medially. Face white, below
antennal implant with distinct brown transverse band. Occiput largely
black-brown, along margins yellow.

Thorax: Scutum shining brown; dark dispersed pilosity,
one transverse band with silvery pilosity and microtrichosity anteriorly of
transverse suture. Postpronotum white. Anepisternum brown, with white band
occupying posterodorsal part, its ventral margin reaching posteroventral corner
or almost so; with pale pilosity, in posteroventral corner with few dark setulae;
one anepisternal seta. Anatergite and katatergite white. Scutellum white. Subscutellum
brown.

Legs: pale yellow, femora and knees brown.

Wing: Anterior part of wing completely brownish
coloured by broad oblique band reaching from well below bcu appendix to apex of
wing, largely covering cells br and basal half dm, the latter along an oblique
line up till where cross-vein R-M touches vein M. Posterior apical band
touching former band near middle of cell r4+5. Basal part of wing
completely brownish coloured. R-M ratio 1.33-1.57.

Abdomen: Shining dark black-brown, posterior margin
of tergites 2-4 with greyish band, anteriorly more yellow; tergite 5 with
median yellow patch.

 

Female

As male except in following characters. Mesonotum,
sometimes second, less developed, transverse band near dorsocentral setae with
silvery pilosity. Female terminalia, oviscape at least as long as abdominal tergites,
black to black-brown colour, with black pilosity. Aculeus brown, stout
cylindrical, about 10 times as long as wide; aculeus tip bluntly pointed,
slightly sinuate.

 

(Description after
De Meyer, 2009)
