# Supplementary material for: A set of multi-entry identification keys to African frugivorous flies (Diptera, Tephritidae)
Source: Zookeys. 2014 Jul 24;(428):97–108. doi: 10.3897/zookeys.428.7366 (PMC4143993; doi:10.3897/zookeys.428.7366)
Supplement: Supplementary material 9 — Key to Perilampsis [file zookeys-428-097-s009.zip › SF9_ZooKeys_key to Perilampsis/key/SF9_key to Perilampsis/Media/Html/Perilampsis pulchella.htm]

Perilampsis pulchella (Austen)


***Perilampsis pulchella*** (Austen)

*Carpophthoromyia pulchella* Austen, 1910: 72.

 

Body length. 3.40-5.40 mm; wing length 3.80-5.05 mm.

 

Male

Head: Antennal segments brown. Arista pubescent, rays
about twice as long as width of base of arista. Frons ventral two-fifths
yellow-white, dorsal part orange-brown. Two frontals, placed parallel to medial
eye margin; two orbitals, placed slightly convergent with inner orbital more
medially. Face white; with broad brown band occupying two-fifths to half of
face near antennal implant. Occiput yellow, with two darker, largely confluent,
patches in dorsal part, sometimes more extensive.

Thorax: Scutum shining brown; dark dispersed pilosity;
two transverse bands with silvery pilosity and microtrichosity, one anteriorly
of transverse suture, second, less developed, band near dorsocentrals; second
band sometimes without microtrichosity or strongly reduced. Postpronotum white.
Anepisternum brown, with white band occupying posterodorsal part, its ventral
margin not reaching posteroventral corner; with pale pilosity except for few
darker setulae in posteroventral corner; one anepisternal seta. Anatergite and katatergite
white. Scutellum white. Subscutellum brown.

Legs: pale yellow, femora darker coloured, from dark
yellow to brown.

Wing: Wing bands brown, well developed. Basal part of
wing brown, subbasal irregular spots and streaks present. Anterior apical band
completely filling cells r1 and r2+3. Posterior apical
band touching anterior apical band. Subapical band isolated. Discal band
reaching posterior wing margin; touching anterior apical band near pterostigma;
well separated from subbasal spots and streaks, at least in cell cu1. R-M ratio
0.84-1.00.

Abdomen: Shining dark brown, posterior fourth to
two-thirds of tergites 2 and 4 with greyish band, anteriorly more orange; tergite
5 with median orange-brown line.

 

Female

As male. Female terminalia, oviscape about as long as
or slightly longer than abdominal tergites, shining brown, with black pilosity.
Aculeus about 15 times longer than wide; aculeus tip narrow, simply pointed.

 

(Description after De Meyer,
2009)
