# Supplementary material for: A set of multi-entry identification keys to African frugivorous flies (Diptera, Tephritidae)
Source: Zookeys. 2014 Jul 24;(428):97–108. doi: 10.3897/zookeys.428.7366 (PMC4143993; doi:10.3897/zookeys.428.7366)
Supplement: Supplementary material 9 — Key to Perilampsis [file zookeys-428-097-s009.zip › SF9_ZooKeys_key to Perilampsis/key/SF9_key to Perilampsis/Media/Html/Perilampsis rubella.htm]

Perilampsis rubella De Meyer 


***Perilampsis rubella*** De Meyer

*Perilampsis rubella* De Meyer, 2009: 2458.

Body length. 3.80 mm; wing length 3.80 mm.

 

Male

Head: Antennal segments yellow-orange. Arista short
pubescent, longest rays at most equal to half the width of base of arista.
Frons ventral half yellow-white, dorsal part darker coloured, partly reddish.
Two frontals, placed parallel to medial eye margin; one orbital. Face white,
below antennal implant with distinctly darker colouration, partly reddish.
Occiput largely yellow; only pair of small brown patches in median part.

Thorax: Scutum shining brown; dark dispersed
pilosity, two transverse bands with silvery pilosity and microtrichosity, one
anteriorly of transverse suture, second near dorsocentral setae. Postpronotum
white. Anepisternum yellow-brown, with white band occupying posterodorsal part,
its ventral margin reaching posteroventral corner; with pale pilosity; one
anepisternal seta. Anatergite and katatergite brown. Scutellum white.
Subscutellum brown.

Legs: completely pale yellow.

Wing: Anterior part of wing completely brownish
coloured by broad band reaching from base of bcu appendix to apex of wing,
covering largely cell br; only marginally basal margin of cell dm, the latter
up till where cross-vein R-M touches vein M. Posterior apical band touching
former band apical third of cell r4+5. Basal part of wing completely
brownish coloured. R-M ratio 0.88.

Abdomen: Shining orange-red, posterior margin of
tergites 2-4 with greyish band.

 

Female

Unknown.

 

(Description after De Meyer,
2009)
