# Supplementary material for: A set of multi-entry identification keys to African frugivorous flies (Diptera, Tephritidae)
Source: Zookeys. 2014 Jul 24;(428):97–108. doi: 10.3897/zookeys.428.7366 (PMC4143993; doi:10.3897/zookeys.428.7366)
Supplement: Supplementary material 9 — Key to Perilampsis [file zookeys-428-097-s009.zip › SF9_ZooKeys_key to Perilampsis/key/SF9_key to Perilampsis/Media/Html/Perilampsis tetradactyla.htm]

Perilampsis tetradactyla Munro


***Perilampsis tetradactyla***Munro

*Perilampsis tetradactyla* Munro, 1933: 40.

Body length. 2.60-4.20 mm; wing length 3.10-4.30 mm.

 

Male

Head: Antennal segments dark brown. Arista pubescent,
longest rays at most twice the width of base of arista. Frons variable, usually
ventral half yellow-white, dorsal part with transverse brown band along width
of orbital bristles and ocellar triangle; sometimes brownish part more
extensive reaching downwards to antennal implant; sometimes ventral part more
orange coloured. Two frontals, placed parallel to medial eye margin; two orbitals,
placed slightly convergent with inner orbital more medially. Face white, dorsal
third dark brown, sometimes completely brown. Occiput black-brown, only margins
white.

Thorax: Scutum shining black-brown, more yellowish
brown near transverse suture; dark dispersed pilosity, one broad transverse
band with silvery pilosity and microtrichosity anteriorly of transverse suture.
Postpronotum white. Anepisternum brown, with white band occupying posterodorsal
part, its ventral margin reaching posteroventral corner or almost so; with pale
pilosity except in posteroventral corner with few dark setulae; one anepisternal
seta. Anatergite and katatergite white. Scutellum white. Subscutellum brown.

Legs: pale yellow, femora and anterior fifth of hind
tibia black-brown, East African specimens with hind tibia more extensively
brown coloured for anterior half.

Wing: Wing bands brown, largely reduced. No basal
spots or streaks, except for subbasal band. Anterior apical band covering cell
r1 completely except for hyaline spot in apical end of cell and
hyaline separation from discal band near pterostigma; covering cell r2+3 only
partially; with subapical tooth into cell r4+5. Posterior apical
band absent. Area between subbasal band and discal band hyaline. Discal band
reaching posterior wing margin. R-M ratio 0.43-0.55.

Abdomen: Shining black-brown, posterior margin of tergite
2 with narrow greyish band, tergite 5 with small yellow
patch posteromedially.

 

Female

As male. Female terminalia, oviscape about two-thirds
of length of abdominal tergites, shining black-brown, with black pilosity. Aculeus
orange, flattened, about 6 times as long as broad, apex strongly narrowed,
pointed tip.

 

(Description after De Meyer,
2009)
