# Supplementary material for: A set of multi-entry identification keys to African frugivorous flies (Diptera, Tephritidae)
Source: Zookeys. 2014 Jul 24;(428):97–108. doi: 10.3897/zookeys.428.7366 (PMC4143993; doi:10.3897/zookeys.428.7366)
Supplement: Supplementary material 9 — Key to Perilampsis [file zookeys-428-097-s009.zip › SF9_ZooKeys_key to Perilampsis/key/SF9_key to Perilampsis/Media/Html/Perilampsis umbrina.htm]

Perilampsis umbrina Munro


***Perilampsis umbrina*** Munro

*Perilampsis umbrina* Munro, 1939: 35.

 

Body length. 3.50-4.30 mm; wing length 3.60-4.20 mm.

 

Male

Head: Antennal segments yellow. Arista bare. Frons
yellow to yellow-orange. Two frontals, placed parallel to medial eye margin;
two orbitals, placed slightly convergent with inner orbital more medially. Face
white. Occiput completely yellow in dorsal part, only faint pair of brownish
patches; otherwise white.

Thorax: Scutum shining yellowish, along margins more
yellow-brown, with dark pilosity; centre with silvery pilosity and microtrichosity,
no transverse bands. Postpronotum white. Anepisternum pale yellow, with white
band occupying posterodorsal part, its ventral margin reaching posteroventral
corner; with pale pilosity; one anepisternal seta. Anatergite and katatergite
white. Scutellum white. Subscutellum brown.

Legs: pale yellow.

Wing: Wing bands brown, well developed but with
diffused margins. Basal part wing brownish coloured, subbasal irregular dark
spots or streaks present, no distinct subbasal band. Anterior apical band
covering cells r1 and r2+3 completely, largely merged
with posterior apical band. Subapical band touching anterior apical band or
almost so. Discal band and anterior apical band united at pterostigma. Discal
band not reaching posterior wing margin, basally confluent with subbasal
streaks and spots. R-M ratio 0.79-0.87.

Abdomen: Shining brown, tergites 1-2 pale yellow
except lateral margins of tergite 2 brown, tergite 4 with posterior half
yellow, in median part yellow band reaching anterior margin, tergite 5 broadly
yellow in median part.

 

Female

As male except for following characters: mid and hind
femur with darker apical patch anteriorly. Wing with distinctly separated
anterior and posterior apical bands and subapical band. Posterior apical band
touching anterior apical band only at base; subapical band isolated. Female terminalia,
oviscape about as long as abdominal tergites, shining brown, with black pilosity.
Aculeus orange, flattened, about 8 times as long as wide; aculeus tip slightly
narrowed, almost straight, pointed.

 

(Description after De Meyer,
2009)
