# Supplementary material for: A set of multi-entry identification keys to African frugivorous flies (Diptera, Tephritidae)
Source: Zookeys. 2014 Jul 24;(428):97–108. doi: 10.3897/zookeys.428.7366 (PMC4143993; doi:10.3897/zookeys.428.7366)
Supplement: Supplementary material 9 — Key to Perilampsis [file zookeys-428-097-s009.zip › SF9_ZooKeys_key to Perilampsis/key/SF9_key to Perilampsis/Media/Html/Perilampsis unita.htm]

Perilampsis unita Munro


***Perilampsis unita*** Munro

*Perilampsis unita* Munro, 1939: 38.

Body length. 3.80-4.30 mm; wing length 3.75-4.10 mm.

 

Male

Head: Antennal segments orange-brown. Arista short
pubescent, longest rays at most equal to width of base of arista. Frons ventral
half white, dorsal part darker yellow to orange-yellow. Two frontals, placed
parallel to medial eye margin; two orbitals, placed slightly convergent with
inner orbital more medially. Face white, with brown transverse band near
antennal implant. Occiput yellow, with pair of darker, isolated, patches in
dorsal part.

Thorax: Scutum shining brown, more yellowish; dark
dispersed pilosity near transverse suture, two broad transverse bands with
silvery pilosity and microtrichosity, one anteriorly of transverse suture, second
near dorsocentrals; sometimes bands less developed or second one largely
missing. Postpronotum white, anteroventral margin brown. Anepisternum brown,
with white band occupying posterodorsal part, its ventral margin reaching posteroventral
corner or almost so; with pale pilosity except in posteroventral corner with
few dark setulae; one anepisternal seta. Anatergite and katatergite white. Scutellum
white, apical part with three brown spots, largely merged, with area in between spots yellow-brown; in dorsal
view restricted to margin. Subscutellum brown.

Legs: pale yellow, femora yellow-brown.

Wing: Wing bands brown, well developed. Basal part of
wing brown, subbasal irregular spots and streaks present. Anterior apical band
completely filling cells r1 and r2+3. Posterior apical
band touching anterior apical band. Subapical band isolated. Discal band not
reaching posterior wing margin; touching anterior apical band near pterostigma;
largely merged with subbasal spots and streaks. R-M ratio 1.00.

Abdomen: Shining brown, posterior margin to half of tergites
2 and 4 with greyish band, tergite 5 with median yellow-orange patch, anteriorly
narrowing and reaching anterior margin.

 

Female

As male. Female terminalia, oviscape shorter than
abdominal tergites, shining brown, with black pilosity. Aculeus orange, about
12 times longer than wide; aculeus tip narrow, pointed.

 

(Description after De Meyer,
2009)
