# Supplementary material for: A set of multi-entry identification keys to African frugivorous flies (Diptera, Tephritidae)
Source: Zookeys. 2014 Jul 24;(428):97–108. doi: 10.3897/zookeys.428.7366 (PMC4143993; doi:10.3897/zookeys.428.7366)
Supplement: Supplementary material 9 — Key to Perilampsis [file zookeys-428-097-s009.zip › SF9_ZooKeys_key to Perilampsis/key/SF9_key to Perilampsis/Media/Html/Perilampsis woodi.htm]

Perilampsis woodi (Bezzi)


***Perilampsis woodi*** (Bezzi)

*Carpophthoromyia woodi* Bezzi, 1924: 96.

Body length. 3.40-4.80 mm; wing length 3.85-4.90 mm.

 

Male

Head: Antennal segments brown. Arista short
pubescent, longest rays at most equal to width of base of arista. Frons ventral
half yellow-white, dorsal part darker yellow. Two frontals, placed parallel to
medial eye margin; two orbitals, placed slightly convergent with inner orbital
more medially. Face white, below antennal implant with distinct brown band.
Occiput in dorsal part with pair of black-brown patches, otherwise dark yellow.

Thorax: Scutum shining brown; dark dispersed
pilosity, two transverse bands with silvery pilosity and microtrichosity, one
anteriorly of transverse suture, second near dorsocentral setae; second one
sometimes missing. Postpronotum white. Anepisternum brown, with white band
occupying posterodorsal part, its ventral margin reaching posteroventral corner
or almost so; with pale pilosity; one anepisternal seta. Anatergite and
katatergite brown. Scutellum white. Subscutellum brown.

Legs: pale yellow, femora and knees brown.

Wing: Anterior part of wing completely brownish
coloured by broad band reaching from well below bcu appendix to apex of wing,
covering largely cells br and basal half dm, the latter along an oblique line
up till where cross-vein R-M touches vein M. Posterior apical band touching
former band near middle of cell r4+5. Basal part of wing completely
brownish coloured. R-M ratio 1.92-2.18.

Abdomen: Shining dark black-brown, posterior margin
of tergites 2-4 with greyish band, anteriorly more yellow; tergite 5 with
median yellow patch.

 

Female

As male. Female terminalia, oviscape at least as long
as abdominal tergites, black to black-brown colour, with black pilosity. Aculeus brown, stout cylindrical, about 17 times as
long as wide; aculeus tip bluntly pointed, with slightly convex lateral
margins, not sinuate.

 

(Description after De Meyer,
2009)
