# Supplementary material for: A set of multi-entry identification keys to African frugivorous flies (Diptera, Tephritidae)
Source: Zookeys. 2014 Jul 24;(428):97–108. doi: 10.3897/zookeys.428.7366 (PMC4143993; doi:10.3897/zookeys.428.7366)
Supplement: Supplementary material 10 — Key to Trirhithrum [file zookeys-428-097-s010.zip › SF10_ZooKeys_key to Trirhithrum/key/SF10_key to Trirhithrum/Media/Html/desc_Trirhithrum_nigerrimum.html]

Natural Language Description


# A set of multi-entry identification keys to African frugivorous flies (Diptera, Tephritidae)

### Massimiliano Virgilio, Ian White, Marc De Meyer

## Trirhithrum nigerrimum

(key to Trirhithrum) sex male or female. (key to Trirhithrum) head (key to Trirhithrum) 1. arista long pubescent to plumose, (key to Trirhithrum) 2. frontal setae 2 pairs, (key to Trirhithrum) 3. face white or pale yellow (at least on lower half). (key to Trirhithrum) thorax (key to Trirhithrum) 5. postpronotal lobe form entirely dark(ish) or dark centrally (usually a spot), (key to Trirhithrum) 7. scutum and abdomen reflecting metallic blue, green or purple no, (key to Trirhithrum) 8. (male) scutum with silvery-white microtrichose covering at least anteriorly no, (key to Trirhithrum) 10. scutum with 2 (or 3) silvery spots of microtrichia on suture no, (key to Trirhithrum) 11. scutellum disc color largely dark, (key to Trirhithrum) 13. (scutellum disk largely dark) scutellum marked with narrow pale lines into five areas no, (key to Trirhithrum) 15. (scutellum disk largely dark) scutellum marked with baso-lateral pale areas yes, (key to Trirhithrum) 16. (scutellum disk largely dark) scutellum margin with spots adjacent base of apical seta yes, (key to Trirhithrum) 17. (scutellum disk largely dark) scutellum with a pair of baso-medial pale spots no, (key to Trirhithrum) 18. (male) anepisternum with upper part pale and lower part dark no, (key to Trirhithrum) 20. anepisternum with 2 setae no, (key to Trirhithrum) 21. anatergite (best viewed from behind) with a bright silvery spot no, (key to Trirhithrum) 56. (female) anepisternum with upper part pale and lower part dark no. (key to Trirhithrum) wings (key to Trirhithrum) 25. (males) wing pattern diffuse, especially in costal region, banding pattern not distinct; if with a suggestion of a costal band it is very deep (extends well below R4+5, at least half way to M) yes, (key to Trirhithrum) 26. (males) basal cells from c to at least bcu cut through by a broad area of hyaline flecks making a clear separation of subbasal and discal bands no, (key to Trirhithrum) 36. (males) cell c largely dark - at most with a small hyaline spot in basal third yes, (key to Trirhithrum) 41. pterostigma markedly darker than rest of pattern yes, (key to Trirhithrum) 46. r-m crossvein separated from edge of discal crossband for a length equal to r-m crossvein no, (key to Trirhithrum) 47. (males) anal lobe colouration coloured but with a hyaline indentation or hyaline (at most narrowly coloured next to vein), (key to Trirhithrum) 49. (males) isolated dark round spot at end of vein A1+Cu2 (bulla) no, (key to Trirhithrum) 58. (females) basal cells from cell c to at least cell bcu cut through by a broad area of hyaline flecks making a clear separation of subbasal dark area and discal band no, (key to Trirhithrum) 59. (females) discal band distally aligned with a point basal to pterostigma no, (key to Trirhithrum) 60. (females) subapical band isolated no, (key to Trirhithrum) 61. (females) subapical band - base partly (but distinctly) in cell dm no, (key to Trirhithrum) 62. (females) subapical band joined to costal band away from discal crossband no, (key to Trirhithrum) 63. (females) posterior apical band present at least to beyond vein M no, (key to Trirhithrum) 67. (females) cell c largely dark (at most with a small hyaline spot in basal third) yes or no, (key to Trirhithrum) 69. (females) anal lobe colouration coloured but with a hyaline indentation, (key to Trirhithrum) 70. (females) anal lobe with hyaline indentation that extends forward through cell cu1 (sometimes into into cell dm) no. (key to Trirhithrum) legs (key to Trirhithrum) 50. (males) femora (all legs) dark, (key to Trirhithrum) 71. (females) femora (all legs) dark. (key to Trirhithrum) abdomen (key to Trirhithrum) 51. abdomen with distinct grey microtrichose spots or bands yes.
