# Supplementary material for: A set of multi-entry identification keys to African frugivorous flies (Diptera, Tephritidae)
Source: Zookeys. 2014 Jul 24;(428):97–108. doi: 10.3897/zookeys.428.7366 (PMC4143993; doi:10.3897/zookeys.428.7366)
Supplement: Supplementary material 10 — Key to Trirhithrum [file zookeys-428-097-s010.zip › SF10_ZooKeys_key to Trirhithrum/key/SF10_key to Trirhithrum/Media/Html/desc_Trirhithrum_nitidum.html]

Natural Language Description


# A set of multi-entry identification keys to African frugivorous flies (Diptera, Tephritidae)

### Massimiliano Virgilio, Ian White, Marc De Meyer

## Trirhithrum nitidum

(key to Trirhithrum) sex male or female. (key to Trirhithrum) head (key to Trirhithrum) 1. arista long pubescent to plumose, (key to Trirhithrum) 2. frontal setae 3 pairs or 2 pairs, (key to Trirhithrum) 3. face white or pale yellow (at least on lower half). (key to Trirhithrum) thorax (key to Trirhithrum) 5. postpronotal lobe form entirely dark(ish) or dark centrally (usually a spot), (key to Trirhithrum) 7. scutum and abdomen reflecting metallic blue, green or purple no, (key to Trirhithrum) 8. (male) scutum with silvery-white microtrichose covering at least anteriorly no, (key to Trirhithrum) 10. scutum with 2 (or 3) silvery spots of microtrichia on suture no, (key to Trirhithrum) 11. scutellum disc color white at least in basal quarter or largely dark, (key to Trirhithrum) 12. (scutellum disk white at least in basal quarter) scutellum pattern type apically dark or pale marks next to apical setae, (key to Trirhithrum) 18. (male) anepisternum with upper part pale and lower part dark no, (key to Trirhithrum) 20. anepisternum with 2 setae yes, (key to Trirhithrum) 21. anatergite (best viewed from behind) with a bright silvery spot no, (key to Trirhithrum) 56. (female) anepisternum with upper part pale and lower part dark no. (key to Trirhithrum) wings (key to Trirhithrum) 26. (males) basal cells from c to at least bcu cut through by a broad area of hyaline flecks making a clear separation of subbasal and discal bands no, (key to Trirhithrum) 27. (males) discal band distally aligned with a point basal to pterostigma no, (key to Trirhithrum) 28. (males) subapical band isolated no, (key to Trirhithrum) 29. (males) subapical band - base partly (but distinctly) in cell dm yes, (key to Trirhithrum) 30. (males) subapical band joined to costal band away from discal band no, (key to Trirhithrum) 31. (males) posterior apical band present at least to beyond vein M yes, (key to Trirhithrum) 33. (males) posterior apical band isolated no, (key to Trirhithrum) 34. (males) posterior apical band joined to costal band and reaching wing margin yes, (key to Trirhithrum) 36. (males) cell c largely dark - at most with a small hyaline spot in basal third no, (key to Trirhithrum) 46. r-m crossvein separated from edge of discal crossband for a length equal to r-m crossvein no, (key to Trirhithrum) 47. (males) anal lobe colouration coloured (at most narrowly hyaline next to vein), (key to Trirhithrum) 49. (males) isolated dark round spot at end of vein A1+Cu2 (bulla) no, (key to Trirhithrum) 58. (females) basal cells from cell c to at least cell bcu cut through by a broad area of hyaline flecks making a clear separation of subbasal dark area and discal band no, (key to Trirhithrum) 59. (females) discal band distally aligned with a point basal to pterostigma no, (key to Trirhithrum) 60. (females) subapical band isolated no, (key to Trirhithrum) 61. (females) subapical band - base partly (but distinctly) in cell dm yes, (key to Trirhithrum) 62. (females) subapical band joined to costal band away from discal crossband no, (key to Trirhithrum) 63. (females) posterior apical band present at least to beyond vein M yes, (key to Trirhithrum) 64. (females) posterior apical band isolated no, (key to Trirhithrum) 65. (females) posterior apical band joined to costal band and reaching wing margin yes, (key to Trirhithrum) 67. (females) cell c largely dark (at most with a small hyaline spot in basal third) no, (key to Trirhithrum) 69. (females) anal lobe colouration coloured (at most narrowly hyaline next to vein). (key to Trirhithrum) legs (key to Trirhithrum) 50. (males) femora (all legs) pale or dark, (key to Trirhithrum) 71. (females) femora (all legs) pale or dark. (key to Trirhithrum) abdomen (key to Trirhithrum) 51. abdomen with distinct grey microtrichose spots or bands yes.
