# Supplementary material for: A set of multi-entry identification keys to African frugivorous flies (Diptera, Tephritidae)
Source: Zookeys. 2014 Jul 24;(428):97–108. doi: 10.3897/zookeys.428.7366 (PMC4143993; doi:10.3897/zookeys.428.7366)
Supplement: Supplementary material 10 — Key to Trirhithrum [file zookeys-428-097-s010.zip › SF10_ZooKeys_key to Trirhithrum/key/SF10_key to Trirhithrum/Media/Html/Trirhithrum albomaculatum.htm]

Trirhithrum albomaculatum (Röder)


***Trirhithrum albomaculatum*** **(Röder)**

*Ceratitis albo-maculata* Röder, 1885: 136.

 

Wing
length=(4.5-)5.0-5.1 mm; Aculeus length=1.48 mm.

Male

Head: Arista micropubescent. Two pairs frontal setae. Face white
or pale yellow.

Thorax: Postpronotal lobe entirely dark. Scutum without
silvery-white microtrichose areas. Scutellum disk white in basal third to
two-thirds; apical dark area undivided. Anepisternum with a diagonal white stripe covering
posterior half; with one seta. Anatergite without a bright
silvery spot.

Wing: Pattern distinct. Subbasal and discal crossbands fused
posterior to Rs, and cell c
extensively hyaline; discal crossband distally aligned with a point
within pterostigma and R-M crossvein aligned with edge of discal crossband. Subapical crossband joined to discal
crossband; base deep, partly in cell dm. Posterior apical crossband reduced to a
short spur. Anal lobe coloured, at most with an indistinct hyaline indentation
or narrowly hyaline along margin. No bulla.

Legs:
Femora dark.

Abdomen:
With distinct silvery microtrichose bands on terga II and IV.

 

Female

Terminalia:
Aculeus abruptly drawn into a point; spermatheca
elongate.

 

(description after White et al., 2003)
