# Supplementary material for: A set of multi-entry identification keys to African frugivorous flies (Diptera, Tephritidae)
Source: Zookeys. 2014 Jul 24;(428):97–108. doi: 10.3897/zookeys.428.7366 (PMC4143993; doi:10.3897/zookeys.428.7366)
Supplement: Supplementary material 10 — Key to Trirhithrum [file zookeys-428-097-s010.zip › SF10_ZooKeys_key to Trirhithrum/key/SF10_key to Trirhithrum/Media/Html/Trirhithrum albonigrum.htm]

Trirhithrum albonigrum (Enderlein)


***Trirhithrum albonigrum*** **(Enderlein)**

*Ceratitis albonigra* Enderlein, 1911: 410.

 

Wing
length=7.2-8.0 mm; Aculeus length=3.44 mm.

Male

Head: Arista plumose. Three pairs frontal setae. Face variable
from dark (possibly just discoloured) to pale (lectotype is bright cream
coloured).

Thorax: Postpronotal lobe entirely dark. Scutum without
silvery-white microtrichose areas. Scutellum disk white; margin dark with three
dark patches. Anepisternum entirely dark; usually with two setae (rarely one).
Anatergite without
a bright silvery spot.

Wing: Pattern distinct. Subbasal and discal crossbands fused
throughout; cell c dark. Discal crossband distally
aligned with base of pterostigma; R-M crossvein well
distal to edge of discal crossband. Subapical crossband joined to discal
crossband; base deep, partly in cell dm. Posterior apical crossband
complete, extending from vein C to wing margin, or with a break in cell r4+5. Anal lobe largely dark, except
for trace of hyaline margin. No bulla.

Legs: Femora dark.

Abdomen: With distinct grey/silvery microtrichose bands (distinct
on tergite IV, less so on tergite II).

 

Female

Terminalia: Aculeus
very long and pointed, with a slight medial constriction; spermatheca long and recurved
(apparently similar to *T. demeyeri*).

 

(description after White et al., 2003)
