# Supplementary material for: A set of multi-entry identification keys to African frugivorous flies (Diptera, Tephritidae)
Source: Zookeys. 2014 Jul 24;(428):97–108. doi: 10.3897/zookeys.428.7366 (PMC4143993; doi:10.3897/zookeys.428.7366)
Supplement: Supplementary material 10 — Key to Trirhithrum [file zookeys-428-097-s010.zip › SF10_ZooKeys_key to Trirhithrum/key/SF10_key to Trirhithrum/Media/Html/Trirhithrum albopleurale.htm]

Trirhithrum albopleurale White


***Trirhithrum albopleurale*** **White**

*Trirhithrum albopleurale* White, 2003: 84.

 

 

Wing length=4.9-5.0 mm.

Male

Head: Arista plumose. Two pairs frontal setae. Face white or pale
yellow.

Thorax: Postpronotal lobe entirely dark. Scutum without
silvery-white microtrichose areas. Scutellum disk entirely white; apical dark
area divided by pale lines. Anepisternum
dorsal half pale; with one seta. Anatergite without a bright silvery spot.

Wing:
Pattern distinct. Subbasal and discal crossbands clearly separated anterior to
anal lobe, and cell c extensively
hyaline; discal crossband distally aligned with a point beyond pterostigma and
R-M crossvein within discal crossband. Subapical crossband joined to discal
crossband. Posterior apical crossband reduced to a short spur. Anal lobe
largely to entirely dark (sometimes with diffused paler areas). No bulla.

Legs: Femora dark.

Abdomen: With a distinct grey/silvery microtrichose band on
tergite IV.

 

Female

Terminalia: Aculeus (not dissected but tip exposed) pointed.

 

(description after White et al., 2003)
