# Supplementary material for: A set of multi-entry identification keys to African frugivorous flies (Diptera, Tephritidae)
Source: Zookeys. 2014 Jul 24;(428):97–108. doi: 10.3897/zookeys.428.7366 (PMC4143993; doi:10.3897/zookeys.428.7366)
Supplement: Supplementary material 10 — Key to Trirhithrum [file zookeys-428-097-s010.zip › SF10_ZooKeys_key to Trirhithrum/key/SF10_key to Trirhithrum/Media/Html/Trirhithrum argenteocuneatum.htm]

Trirhithrum argenteocuneatum Hancock


***Trirhithrum argenteocuneatum*** **Hancock**

*Trirhithrum argenteocuneatum* Hancock, 1984: 293.

 

Wing
length=2.7-3.5 mm; Aculeus length=0.72 mm.

Male

Head: Arista plumose. Two pairs frontal setae. Face dark.

Thorax: Postpronotal lobe entirely dark or pale laterally. Scutum
with silvery-white microtrichose covering anteriorly, shaped as an inverted V. Scutellum disk dark; margin not marked with pale spots
baso-laterally or adjacent to apical setae. Anepisternum largely red-brown;
dorsal edge pale (line continuous with that on postpronotum); one seta.
Anatergite (best viewed from behind) with a bright silvery spot.

Wing: Pattern distinct. Subbasal and discal crossbands fused
posterior to Rs and cell c extensively hyaline; discal crossband distally
aligned with a point distal to pterostigma. Subapical crossband joined to discal crossband; base narrow, largely or
entirely confined to cell r4+5. Posterior apical
crossband complete, extending from vein C to wing
margin. Anal lobe largely hyaline. An isolated dark round spot at end of vein A1+Cu2
(bulla).

Legs: Femora pale.

Abdomen: Without grey/silvery microtrichose spots/bands.

 

Female

As in male except: face pale in most of lower half (discoloured in
type series); scutum lacking any distinct microtrichose covering; femora dark;
wing without a
bulla. Aculeus short, stout and pointed (appears asymmetric under a coverslip;
dorsal view apparently similar to *T. meladiscum*;
spermatheca bulbous (similar to *T. senex*).

 

(description after White et al., 2003)
