# Supplementary material for: A set of multi-entry identification keys to African frugivorous flies (Diptera, Tephritidae)
Source: Zookeys. 2014 Jul 24;(428):97–108. doi: 10.3897/zookeys.428.7366 (PMC4143993; doi:10.3897/zookeys.428.7366)
Supplement: Supplementary material 10 — Key to Trirhithrum [file zookeys-428-097-s010.zip › SF10_ZooKeys_key to Trirhithrum/key/SF10_key to Trirhithrum/Media/Html/Trirhithrum argutum.htm]

Trirhithrum argutum (Enderlein)


***Trirhithrum argutum*** **(Enderlein)**

*Ceratitis arguta* Enderlein, 1920: 354.

 

Wing length=4.7 mm.

Female

Identical to *T. overlaeti* except as follows: White area on
anepisternum deep, extending about three-quarters of the depth. Abdomen without any microtrichose areas.

Male

Unknown.

 

(After White et al., 2003)
