# Supplementary material for: A set of multi-entry identification keys to African frugivorous flies (Diptera, Tephritidae)
Source: Zookeys. 2014 Jul 24;(428):97–108. doi: 10.3897/zookeys.428.7366 (PMC4143993; doi:10.3897/zookeys.428.7366)
Supplement: Supplementary material 10 — Key to Trirhithrum [file zookeys-428-097-s010.zip › SF10_ZooKeys_key to Trirhithrum/key/SF10_key to Trirhithrum/Media/Html/Trirhithrum basale.htm]

Trirhithrum basale Bezzi


***Trirhithrum basale*** **Bezzi**

*Trirhithrum basale* Bezzi, 1924a: 107.

 

Wing
length=3.3-3.7 mm; Aculeus length=0.54 mm.

Male

Head: Arista plumose. Two pairs frontal setae. Face dark except
for white band, which may be broken medially.

Thorax: Postpronotal lobe pale, with a dark central mark. Scutum without
silvery-white microtrichose areas. Scutellum disk dark; margin with
baso-lateral pale areas (normally as paired spots, often coalesced into a
line); no spots adjacent to base of apical seta. Anepisternum largely dark;
dorsal edge narrowly pale; one seta. Anatergite (best viewed from behind) with
a bright silvery spot.

Wing:
Pattern distinct. Subbasal and discal crossbands more or less fused posterior
to M, and cell c extensively
hyaline; discal crossband distally aligned with a point beyond pterostigma, and R-M crossvein within discal
crossband. Subapical crossband either joined to discal crossband or almost
isolated from it (usually a trace of a join). Posterior apical crossband
reduced to a short spur. Anal lobe coloured but with a hyaline indentation
(ending before vein A1+Cu2) or
broadly hyaline distally. No bulla.

Legs: Femora dark.

Abdomen: With microtrichose areas on terga II to IV, formed into
bands across tergites II and IV, interconnected by spots/stripes on tergite
III.

 

Female

Terminalia: Aculeus short and pointed; spermatheca elongate and
curved.

 

(description after White et al., 2003)
