# Supplementary material for: A set of multi-entry identification keys to African frugivorous flies (Diptera, Tephritidae)
Source: Zookeys. 2014 Jul 24;(428):97–108. doi: 10.3897/zookeys.428.7366 (PMC4143993; doi:10.3897/zookeys.428.7366)
Supplement: Supplementary material 10 — Key to Trirhithrum [file zookeys-428-097-s010.zip › SF10_ZooKeys_key to Trirhithrum/key/SF10_key to Trirhithrum/Media/Html/Trirhithrum bimaculatum.htm]

Trirhithrum bimaculatum (Röder)


***Trirhithrum bimaculatum*** **(Röder)**

*Ceratitis bimaculata* Röder, 1885: 135.

 

The type (Female) of *C. bimaculata* is lost, and was not
available for study to either Bezzi 1918, or Munro 1934, 1957. Analysis of the
description suggests that Bezzi (1918) was correct in placing the species in *Trirhithrum*,
and that it is probably related to, or perhaps conspecific with, *T.
occipitale*. Another interpretation of the identity of *C. bimaculata*
is that it matches the females of *T. coffeae*. As this latter species is
not known from coastal eastern Africa, however, this appears unlikely.
According to M. De Meyer (pers. comm.), the description indicates that it has a
complete subapical crossband, and a posterior apical band reduced to a spur,
not reaching vein M (indicating that it belongs to this genus). The
anepisternum is described as shining grey with no mention of a contrasting
paler dorsal margin. The abdomen has the hind margin of tergite II grey, and a
pair of grey spots on tergite III.

 

Munro (1934) had interpreted the description as indicating that
the subbasal and discal crossbands were separate (as in *T. senex* or*T. viride*, species which lack the apparently all dark anepisternum).
However, he later (Munro 1957) interpreted these crossbands as fused posterior
to Rs, as in *T. occipitale* or female *T. coffeae*. Consequently *C.
bimaculata* cannot be recognised or placed in the key.

 

(after White et al., 2003)
