# Supplementary material for: A set of multi-entry identification keys to African frugivorous flies (Diptera, Tephritidae)
Source: Zookeys. 2014 Jul 24;(428):97–108. doi: 10.3897/zookeys.428.7366 (PMC4143993; doi:10.3897/zookeys.428.7366)
Supplement: Supplementary material 10 — Key to Trirhithrum [file zookeys-428-097-s010.zip › SF10_ZooKeys_key to Trirhithrum/key/SF10_key to Trirhithrum/Media/Html/Trirhithrum brachypterum.htm]

Trirhithrum brachypterum Munro


***Trirhithrum brachypterum*** **Munro**

[*Ceratitis*] *Trirhithrum* *brachypterum* Munro,
1934: 480.

 

Wing length=3.0-3.7 mm.

Male

Head: Arista plumose. Two pairs frontal setae. Face dark except
for white band across centre and sometimes a trace of a second band under antennal
insertions (face often discoloured to dark or pale; dark in lectotype).

Thorax: Postpronotal lobe pale with a dark central mark. Scutum
without silvery-white microtrichose areas. Scutellum disk dark, except for a
pair of irregularly rounded baso-medial pale spots; margin with baso-lateral
pale areas (two spots or coalesced into a streak); no spots adjacent base of
apical seta. Anepisternum largely dark; dorsal edge narrowly pale; one seta.
Anatergite without a bright silvery spot.

Wing: Pattern distinct. Subbasal and discal crossbands fused
posterior to Rs and cell c extensively hyaline; discal crossband distally
aligned with apex of pterostigma and R-M crossvein aligned to edge of discal
crossband. Subapical crossband joined to discal crossband. Posterior apical
crossband reduced to a short spur. Anal lobe coloured but with a hyaline
indentation (ending before vein A1+Cu2). No bulla.

Legs: Femora dark.

Abdomen: With diffuse grey/silvery microtrichose spots, sometimes
formed into bands.

 

Female

Unknown.

 

(description after White et al., 2003)
