# Supplementary material for: A set of multi-entry identification keys to African frugivorous flies (Diptera, Tephritidae)
Source: Zookeys. 2014 Jul 24;(428):97–108. doi: 10.3897/zookeys.428.7366 (PMC4143993; doi:10.3897/zookeys.428.7366)
Supplement: Supplementary material 10 — Key to Trirhithrum [file zookeys-428-097-s010.zip › SF10_ZooKeys_key to Trirhithrum/key/SF10_key to Trirhithrum/Media/Html/Trirhithrum coffeae.htm]

Trirhithrum coffeae Bezzi


***Trirhithrum coffeae*** **Bezzi**

*Trirhithrum nigerrimum* var *coffeae* Bezzi,
1918: 241.

 

Wing
length=2.6-3.8 mm; Aculeus length0.70-0.82 mm.

Male

Head: Arista long plumose. Two pairs frontal setae. Face mostly
white.

Thorax: Postpronotal lobe entirely dark or sometimes with a narrow
pale margin. Scutum without silvery-white microtrichose areas. Scutellum disk
dark; margin with baso-lateral pale areas (normally streaks); spots adjacent to
bases of apical setae. Anepisternum largely dark but sometimes with a very narrow
pale line across dorsal margin; usually with one seta (a single specimen with
two has been examined). Anatergite without a bright silvery spot.

Wing: Pattern diffuse, especially in costal region; banding
pattern not distinct. Cell c largely hyaline. With a distinct dark mark on C
at/before end of Sc and with a contrastingly dark area near base of cells cu1;
pterostigma not markedly darker than rest of pattern. Anal lobe largely hyaline
or coloured but with a hyaline indentation. No bulla.

Legs: Femora dark.

Abdomen: With distinct grey microtrichose stripes. Terminalia:
Similar to *T. psychotriae* White.

 

Female

Head, thorax, legs & abdomen: As in male. Wing pattern
distinct. Subbasal and discal crossbands not fully separated posterior to Rs
and cell c extensively hyaline; cell bc with dark area extended well into basal
half of cell; a distinct dark mark on C at/before end of Sc; pterostigma not
markedly darker than rest of pattern; basal area of cell r1
immediately above vein R2+3/R4+5 bifurcation with a dark
spot that is at most narrowly connected to large dark area of cell r1;
discal crossband distally aligned with a point within pterostigma and R-M
crossvein within discal crossband; often somewhat darkened in cell cu1.
Subapical crossband usually joined to discal crossband (rarely slightly
separated); base narrow, usually largely or entirely confined to cell r4+5.
Posterior apical crossband reduced to a short spur. Anal lobe coloured but with
a hyaline indentation (ending before or only slightly anterior to vein A1+Cu2).
No bulla. Terminalia: Aculeus short, stout and pointed (appears asymmetric
under a coverslip; dorsal view apparently similar to *T. leonense*;
spermatheca curved and bulbous (similar to *T. occipitale*).

 

(description after White et al., 2003)
