# Supplementary material for: A set of multi-entry identification keys to African frugivorous flies (Diptera, Tephritidae)
Source: Zookeys. 2014 Jul 24;(428):97–108. doi: 10.3897/zookeys.428.7366 (PMC4143993; doi:10.3897/zookeys.428.7366)
Supplement: Supplementary material 10 — Key to Trirhithrum [file zookeys-428-097-s010.zip › SF10_ZooKeys_key to Trirhithrum/key/SF10_key to Trirhithrum/Media/Html/Trirhithrum crescentis.htm]

Trirhithrum crescentis Hancock


***Trirhithrum crescentis*** **Hancock**

*Trirhithrum crescentis* Hancock, 1984: 294.

 

Wing length=3.2 mm.

Male

Head: Arista plumose. Two pairs of frontal setae. Face dark (may
be discoloured).

Thorax: Postpronotal lobe pale with a dark spot. Scutum with an
ill-defined whitish microtrichose covering. Scutellum disk dark; margin not
marked with pale spots baso-laterally or adjacent to apical setae. Anepisternum
largely dark; dorsal edge narrowly pale; one seta. Anatergite without a silvery
spot.

Wing: Pattern distinct. Subbasal and discal crossbands fused
posterior to Rs and cell c extensively hyaline; discal crossband distally
aligned to apex of pterostigma and R-M crossvein within discal crossband.
Subapical crossband joined to discal crossband; base narrow, largely or
entirely confined to cell r4+5. Posterior apical crossband complete,
extending from vein C to wing margin. Anal lobe coloured, but with a hyaline
indentation (ending before vein A1+Cu2). No bulla.

Legs: Femora pale.

Abdomen: With distinct grey microtrichose bands on terga II and
IV.

 

Female

Unknown; expected to be similar to male, but likely to have dark
femora.

 

(description after White et al., 2003)
