# Supplementary material for: A set of multi-entry identification keys to African frugivorous flies (Diptera, Tephritidae)
Source: Zookeys. 2014 Jul 24;(428):97–108. doi: 10.3897/zookeys.428.7366 (PMC4143993; doi:10.3897/zookeys.428.7366)
Supplement: Supplementary material 10 — Key to Trirhithrum [file zookeys-428-097-s010.zip › SF10_ZooKeys_key to Trirhithrum/key/SF10_key to Trirhithrum/Media/Html/Trirhithrum culcasiae.htm]

Trirhithrum culcasiae White & Copeland


***Trirhithrum culcasiae*** **White & Copeland**

*Trirhithrum culcasiae* White & Copeland, 2003: 92.

 

Wing
length=2.8-4.0 mm; �Aculeus length=0.68 mm.

Male

Head: Arista long plumose. Two pairs frontal setae. Face black
except for pale lateral and dorsal margins.

Thorax: Postpronotal lobe with a dark central mark. Scutum with
thin microtrichose covering. Scutellum disk largely dark and marked with a
narrow wavy yellow transverse line. Anepisternum largely dark; dorsal quarter
pale; one seta.

Wing: Pattern distinct. Subbasal and discal crossbands
indistinctly separated by numerous hyaline flecks; cell c extensively hyaline;
discal crossband beyond pterostigma, and R-M crossvein within discal crossband. Subapical crossband
joined to discal crossband; base
deep, partly in cell dm. Posterior apical crossband
reduced to a short spur. Anal lobe coloured but with a hyaline indentation
(ending before vein A1+Cu2). No bulla.

Legs: Femora dark.

Abdomen: With reticulate grey/silvery microtrichose pattern.

 

Female

Terminalia: Aculeus fairly short and pointed (appears asymmetric
under a coverslip; dorsal view apparently similar to *T. leonense*;
spermatheca with a bulbous apex, apparently similar to *T. occipitale*.

�

(description after White et al., 2003)
