# Supplementary material for: A set of multi-entry identification keys to African frugivorous flies (Diptera, Tephritidae)
Source: Zookeys. 2014 Jul 24;(428):97–108. doi: 10.3897/zookeys.428.7366 (PMC4143993; doi:10.3897/zookeys.428.7366)
Supplement: Supplementary material 10 — Key to Trirhithrum [file zookeys-428-097-s010.zip › SF10_ZooKeys_key to Trirhithrum/key/SF10_key to Trirhithrum/Media/Html/Trirhithrum demeyeri.htm]

Trirhithrum demeyeri White & Hancock


***Trirhithrum demeyeri*** **White & Hancock.**

*Trirhithrum demeyeri* White & Hancock, 2003: 93.

 

 

Wing
length=5.4-7.0 mm; Aculeus length=2.14 mm.

Male

Head: Arista long pubescent to plumose. Three pairs frontal setae.
Face variable from dark to pale.

Thorax: Postpronotal lobe entirely dark. Scutum without
silvery-white microtrichose areas. Scutellum disk white; margin dark between
setae. Anepisternum entirely dark; two setae. Anatergite without a bright
silvery spot.

Wing: Pattern distinct. Subbasal and discal crossbands fused
throughout; cell c largely dark. Discal crossband distally aligned with a point
basal to pterostigma; R-M crossvein well distal to edge of discal crossband.
Subapical crossband joined to discal crossband; base deep, partly in cell dm.
Posterior apical crossband reduced to a short spur. Anal lobe largely to
entirely dark. No bulla.

Legs: Femora dark.

Abdomen: With distinct grey/silvery microtrichose spots/bands on
tergite IV.

 

Female

Terminalia: Aculeus long and bluntly pointed; spermatheca curved and
bulbous apically (the apical papilla may be a variable feature).

 

(description after White et al., 2003)
