# Supplementary material for: A set of multi-entry identification keys to African frugivorous flies (Diptera, Tephritidae)
Source: Zookeys. 2014 Jul 24;(428):97–108. doi: 10.3897/zookeys.428.7366 (PMC4143993; doi:10.3897/zookeys.428.7366)
Supplement: Supplementary material 10 — Key to Trirhithrum [file zookeys-428-097-s010.zip › SF10_ZooKeys_key to Trirhithrum/key/SF10_key to Trirhithrum/Media/Html/Trirhithrum dimorphum.htm]

Trirhithrum dimorphum Munro


***Trirhithrum dimorphum*** **Munro**

[*Ceratitis*] *Trirhithrum* *dimorphum* Munro,
1934: 484

 

Wing length=2.7-3.2 mm.

Male

Head: Arista long plumose. Two pairs of frontal setae. Face dark.

Thorax: Postpronotal lobe with a dark central mark. Scutum lacking
any distinct microtrichose covering. Scutellum disk dark; margin with
baso-lateral pale areas (normally a streak); no spots adjacent to bases of
apical setae. Anepisternum largely dark; dorsal edge narrowly pale; one seta.
Anatergite (best viewed from behind) with a bright silvery spot.

Wing: Pattern distinct. Subbasal and discal crossbands fused
posterior to Rs and cell c extensively hyaline; discal crossband distally
aligned with a point within pterostigma. Subapical crossband joined to discal
crossband; base deep, partly in cell dm. Posterior apical crossband extending
to beyond vein M but not reaching wing margin. Anal lobe largely hyaline. An
isolated dark round spot at end of vein A1+Cu2 (bulla).

Legs: Femora pale.

Abdomen: Without grey/silvery microtrichose spots/bands.

 

Female

Same as male except: Anepisternum entirely dark; femora dark; wing
without a bulla; base of subapical crossband narrow, not extended into cell dm.
The face appears slightly pale (only available specimen is discoloured).

 

(description after White et al., 2003)
