# Supplementary material for: A set of multi-entry identification keys to African frugivorous flies (Diptera, Tephritidae)
Source: Zookeys. 2014 Jul 24;(428):97–108. doi: 10.3897/zookeys.428.7366 (PMC4143993; doi:10.3897/zookeys.428.7366)
Supplement: Supplementary material 10 — Key to Trirhithrum [file zookeys-428-097-s010.zip › SF10_ZooKeys_key to Trirhithrum/key/SF10_key to Trirhithrum/Media/Html/Trirhithrum divisum.htm]

Trirhithrum divisum Munro


***Trirhithrum divisum*** **Munro**

[*Ceratitis*] *Trirhithrum divisum* Munro, 1934: 486

 

Wing length=3.5-4.1 mm.

Female Identical to *T. occipitale* except as follows: Cell c
with a small central mark and large basal and apical marks; apical partly
divided and approximated to central mark; base of subapical crossband deep,
partly in cell dm. Anepisternum with a narrow but usually distinct pale dorsal
margin (except holotype *T. torina*); terga II and IV with microtrichose
spots or bands; no microtrichiae on tergite III. Aculeus (not dissected but tip
exposed in holotype *T. divisum*) apparently drawn to a much narrower
point than *T. occipitale*; apex apparently similar in shape to *T.
leonense*.

Male

Unknown.

 

(description after White et al., 2003)
