# Supplementary material for: A set of multi-entry identification keys to African frugivorous flies (Diptera, Tephritidae)
Source: Zookeys. 2014 Jul 24;(428):97–108. doi: 10.3897/zookeys.428.7366 (PMC4143993; doi:10.3897/zookeys.428.7366)
Supplement: Supplementary material 10 — Key to Trirhithrum [file zookeys-428-097-s010.zip › SF10_ZooKeys_key to Trirhithrum/key/SF10_key to Trirhithrum/Media/Html/Trirhithrum fraternum.htm]

Trirhithrum fraternum Munro


***Trirhithrum fraternum*** **Munro**

[*Ceratitis*] *Trirhithrum fraternum* Munro, 1934: 482

 

Wing
length=3.7-4.2 mm; Aculeus length=0.94 mm.

Male

Head: Arista plumose. Two pairs frontal setae. Face white.

Thorax: Postpronotal lobe entirely dark or sometimes with an indistinct
pale margin. Scutum without silvery-white microtrichose areas. Scutellum disk
dark; margin with baso-lateral pale spots; spots adjacent to bases of apical
setae. Anepisternum entirely dark; one seta. Anatergite without a bright
silvery spot.

Wing: Pattern distinct. Subbasal and discal crossbands fused
posterior to Rs and cell c extensively hyaline; cell bc with dark area not
extended into basal half of cell. Discal crossband distally aligned with a
point within pterostigma and R-M crossvein within discal crossband. Subapical
crossband not joined to discal crossband (rarely almost joined). Posterior
apical crossband reduced to a short spur. Anal lobe coloured but with a hyaline
indentation (ending before vein A1+Cu2). No bulla.

Legs: Femora dark.

Abdomen: With distinct grey microtrichose stripes.

 

Female

Terminalia: Aculeus fairly short and pointed (appears asymmetric
when viewed under a coverslip; dorsal view apparently similar to *T. leonense*);
spermatheca curved and bulbous (similar to *T. occipitale*).

 

(description after White et al., 2003)
