# Supplementary material for: A set of multi-entry identification keys to African frugivorous flies (Diptera, Tephritidae)
Source: Zookeys. 2014 Jul 24;(428):97–108. doi: 10.3897/zookeys.428.7366 (PMC4143993; doi:10.3897/zookeys.428.7366)
Supplement: Supplementary material 10 — Key to Trirhithrum [file zookeys-428-097-s010.zip › SF10_ZooKeys_key to Trirhithrum/key/SF10_key to Trirhithrum/Media/Html/Trirhithrum homogeneum.htm]

Trirhithrum homogeneum Bezzi


***Trirhithrum homogeneum*** **Bezzi**

*Trirhithrum homogeneum* Bezzi, 1924a: 105

 

Wing
length=4.7-5.4 mm; Aculeus length=0.74 mm.

Male

Head: Arista long pubescent to plumose. Two pairs frontal setae (a
single specimen has been examined with three on the left side). Face pale, at
least in lower half.

Thorax: Postpronotal lobe entirely dark. Scutum without
silvery-white microtrichose areas. Scutellum disk dark; margin without
baso-lateral pale spots; no spots adjacent to bases of apical setae.
Anepisternum entirely dark; 2-3 setae (lectotype badly damaged and this
character not checked; a single specimen [Zimbabwe, Vumba, observed with just
one]). Anatergite without a bright silvery spot.

Wing: Pattern distinct. Subbasal and discal crossbands fused
throughout; cell c entirely dark. Discal crossband distally aligned to base of
pterostigma; R-M crossvein well distal to edge of discal crossband. Subapical
crossband joined to discal crossband; base deep, partly in cell dm. Posterior
apical crossband variable; usually reduced to a short spur off the costal band;
rarely extending to vein M, with a trace beyond (as in lectotype), but not
reaching wing margin as any more than a trace. Anal lobe entirely dark. No
bulla.

Legs: Femora dark.

Abdomen: Without any microtrichose bands or spots; tergite IV
often with a pale (orange) stripe medially on tergite IV.

 

Female

Terminalia: Aculeus apex serrate; spermatheca apically bulbous.

(description after White et al., 2003)
