# Supplementary material for: A set of multi-entry identification keys to African frugivorous flies (Diptera, Tephritidae)
Source: Zookeys. 2014 Jul 24;(428):97–108. doi: 10.3897/zookeys.428.7366 (PMC4143993; doi:10.3897/zookeys.428.7366)
Supplement: Supplementary material 10 — Key to Trirhithrum [file zookeys-428-097-s010.zip › SF10_ZooKeys_key to Trirhithrum/key/SF10_key to Trirhithrum/Media/Html/Trirhithrum inscriptum.htm]

Trirhithrum inscriptum (Graham)


***Trirhithrum inscriptum*** **(Graham)**

*Ceratitis inscripta* Graham, 1910: 164

 

Wing
length=3.7-4.0 mm; Aculeus length=0.84 mm.

Male

Head: Arista long plumose. Two pairs frontal setae. Face usually
dark except for white band, which may be broken medially; rarely pale in whole
of lower half.

Thorax: Postpronotal lobe with a dark central mark. Scutum with
thin microtrichose covering. Scutellum disk largely dark and usually divided by narrow yellow lines into five areas (sometimes these lines
are reduced to crescent-shaped markings; sometimes apparently
absent but possibly only due to staining). Anepisternum largely dark; dorsal edge narrowly pale; one seta.
Anatergite (best viewed posteriorly) with a bright silvery
spot.

Wing: Pattern distinct. Subbasal and discal crossbands
indistinctly separated by numerous hyaline flecks; cell c extensively hyaline;
discal crossband distally aligned with a point beyond pterostigma, and R-M crossvein within
discal crossband. Subapical crossband usually joined to discal crossband; base
narrow, largely or entirely confined to cell r4+5. Posterior apical
crossband reduced to a short spur. Anal lobe coloured but with a hyaline
indentation (ending posterior
to vein A1+Cu2). No bulla.

Legs: Femora dark.

Abdomen: With reticulate grey/silvery microtrichose pattern.

 

Female

Terminalia: Aculeus fairly short and pointed (appears asymmetric
under a coverslip; dorsal view apparently similar to *T. leonense*);
spermatheca sinuate with a bulbous apex.

 

(description after White et al., 2003)
