# Supplementary material for: A set of multi-entry identification keys to African frugivorous flies (Diptera, Tephritidae)
Source: Zookeys. 2014 Jul 24;(428):97–108. doi: 10.3897/zookeys.428.7366 (PMC4143993; doi:10.3897/zookeys.428.7366)
Supplement: Supplementary material 10 — Key to Trirhithrum [file zookeys-428-097-s010.zip › SF10_ZooKeys_key to Trirhithrum/key/SF10_key to Trirhithrum/Media/Html/Trirhithrum iridescens.htm]

Trirhithrum iridescens Hancock


***Trirhithrum iridescens*** **Hancock**

*Trirhithrum iridescens* Hancock, 1984: 296

 

Wing
length=3.1-4.4 mm; Aculeus length=0.50 mm.

Male

Head: Arista long pubescent. Two pairs frontal setae. Face dark
except for a white band, which may be broken medially.

Thorax: Postpronotal lobe pale laterally, or around margin, leaving a dark central
mark. Scutum without silvery-white microtrichose areas. Scutellum disk white in
basal third to half (sometimes indistinctly darkened along mid-line suggesting
two large white coalesced spots); entirely dark apically. Anepisternum largely
dark; dorsal quarter pale; one seta. Anatergite (best viewed from behind) with
a bright silvery spot.

Wing: Pattern distinct. Subbasal and discal crossbands fused
posterior to Rs, and cell c
extensively hyaline; discal crossband distally aligned with apex of pterostigma, and R-M crossvein within
or slightly beyond discal crossband. Subapical crossband joined to discal
crossband; base narrow, largely or entirely confined to cell r4+5.
Posterior apical crossband extending to beyond vein M but not reaching wing
margin. Anal lobe coloured but with a hyaline indentation (ending before vein A1+Cu2).
No bulla.

Legs: Femora dark.

Abdomen: With distinct grey microtrichose bands on terga II and
IV.

 

Female

Terminalia: Aculeus very short, stout and pointed (appears
slightly asymmetric under a coverslip; spermatheca long bulbous; the apical
papilla may be a variable feature).

 

(description after White et al., 2003)
