# Supplementary material for: A set of multi-entry identification keys to African frugivorous flies (Diptera, Tephritidae)
Source: Zookeys. 2014 Jul 24;(428):97–108. doi: 10.3897/zookeys.428.7366 (PMC4143993; doi:10.3897/zookeys.428.7366)
Supplement: Supplementary material 10 — Key to Trirhithrum [file zookeys-428-097-s010.zip › SF10_ZooKeys_key to Trirhithrum/key/SF10_key to Trirhithrum/Media/Html/Trirhithrum leonense.htm]

Trirhithrum leonense White & Hancock


***Trirhithrum leonense*** **White & Hancock**

*Trirhithrum leonense* White & Hancock, 2003: 98.

Wing
length=2.9-3.9 mm; R; Aculeus length=0.90 mm.

Male

Head: Arista long pubescent to plumose. Two pairs frontal setae.
Face white.

Thorax: Postpronotal lobe entirely dark. Scutum without
silvery-white microtrichose areas. Scutellum disk dark; margin with
baso-lateral pale
spots adjacent to bases of apical setae. Anepisternum
largely dark; dorsal edge narrowly pale; one seta. Anatergite without a bright
silvery spot.

Wing:
Pattern distinct. Subbasal and discal crossbands
fused posteriorly, and cell c extensively hyaline; cell bc with dark area not
extended into basal half of cell. Discal crossband distally aligned with a
point within pterostigma, and R-M crossvein within discal crossband. Subapical crossband
joined to discal crossband (and often closely approximated or even narrowly
joined to costal band beyond its base); base narrow but partly in cell dm.
Posterior apical crossband reduced to a short spur. Anal lobe coloured, but
with a hyaline indentation (ending before vein A1+Cu2).
No bulla.

Legs: Femora dark.

Abdomen: With distinct grey microtrichose stripes.

 

Female

Terminalia: Aculeus fairly short (appears slightly asymmetric
under a coverslip); spermatheca curved and bulbous (similar to *T. occipitale*).

 

(description after White et al., 2003)
