# Supplementary material for: A set of multi-entry identification keys to African frugivorous flies (Diptera, Tephritidae)
Source: Zookeys. 2014 Jul 24;(428):97–108. doi: 10.3897/zookeys.428.7366 (PMC4143993; doi:10.3897/zookeys.428.7366)
Supplement: Supplementary material 10 — Key to Trirhithrum [file zookeys-428-097-s010.zip › SF10_ZooKeys_key to Trirhithrum/key/SF10_key to Trirhithrum/Media/Html/Trirhithrum manganum.htm]

Trirhithrum manganum Munro


***Trirhithrum manganum*** **Munro**

*Trirhithrum manganum* Munro, 1954: 543

 

Wing
length=3.8-4.5 mm; Aculeus length=1.04 mm.

Male

Head: Arista plumose. Two pairs frontal setae. Face pale, at least
in lower half.

Thorax: Postpronotal lobe entirely dark. Scutum with three
(lateral and a medial) silvery spots of microtrichia on suture (damaged in
holotype). Scutellum disk dark; margin without baso-lateral pale areas; without
spots adjacent to bases of apical setae. Anepisternum entirely dark; with one
seta. Anatergite without a bright silvery spot.

Wing: Pattern more or less diffuse; poorly defined costal band very deep, extending
well posterior to vein R4+5 (mid-way to vein M); crossbands
indistinct. No bulla.

Legs: Femora dark.

Abdomen: With distinct grey microtrichose bands.

Female

Head, thorax, legs and abdomen as in male, except scutum with only
two (lateral) silvery spots of microtrichia on suture. Wing Pattern distinct.
Subbasal and discal crossbands fused posterior to Rs, and cell c extensively
hyaline; discal crossband distally aligned with a point beyond pterostigma, and R-M crossvein within
discal crossband. Subapical crossband joined to discal crossband; base narrow,
largely or entirely confined to cell r4+5. Posterior apical
crossband complete, extending from vein C to wing margin. Anal lobe coloured
but with a hyaline indentation (ending before vein A1+Cu2).
No bulla. Female Terminalia with aculeus fairly short and pointed (appears
asymmetric under a coverslip; dorsal view apparently similar to *T. leonense*;
spermatheca curved and long bulbous.

 

(description after White et al., 2003)
