# Supplementary material for: A set of multi-entry identification keys to African frugivorous flies (Diptera, Tephritidae)
Source: Zookeys. 2014 Jul 24;(428):97–108. doi: 10.3897/zookeys.428.7366 (PMC4143993; doi:10.3897/zookeys.428.7366)
Supplement: Supplementary material 10 — Key to Trirhithrum [file zookeys-428-097-s010.zip › SF10_ZooKeys_key to Trirhithrum/key/SF10_key to Trirhithrum/Media/Html/Trirhithrum meladiscum.htm]

Trirhithrum meladiscum Munro


***Trirhithrum meladiscum*** **Munro**

*Trirhithrum meladiscum* Munro, 1938: 166

 

Wing
length=3.5-4.1 mm; Aculeus length=0.76 mm.

Male

Head: Arista plumose. Two pairs frontal setae. Face dark.

Thorax: Postpronotal lobe pale with a dark central mark. Scutum
without any distinct microtrichose covering. Scutellum disk dark; margin with
baso-lateral pale areas (pair of spots or coalesced into a streak); no spots
adjacent to bases of apical setae. Anepisternum yellow-brown, with narrow very
pale line across dorsal margin; one seta. Anatergite (best viewed from behind)
often with a bright silvery spot.

Wing: Pattern distinct. Subbasal and discal crossbands more or
less separated posterior to Rs; discal crossband distally aligned with a point
within pterostigma. Subapical crossband joined to discal crossband; base
narrow, largely or entirely confined to cell r4+5. Posterior apical
crossband extending to beyond vein M and usually reaching wing margin. Anal
lobe largely hyaline. An isolated dark round spot at end of vein A1+Cu2
(bulla).

Legs: Femora pale.

Abdomen: With grey microtrichose band on tergite II (may be broken
centrally); sometimes with very small spots on tergite III.

 

Female

Same as male except: face pale (not always distinct); wing without
a bulla; femora dark. Terminalia with aculeus short, stout and pointed (may
appear asymmetric under a coverslip); spermatheca bulbous (similar to *T.
senex*).

�

(description after White et al., 2003)
