# Supplementary material for: A set of multi-entry identification keys to African frugivorous flies (Diptera, Tephritidae)
Source: Zookeys. 2014 Jul 24;(428):97–108. doi: 10.3897/zookeys.428.7366 (PMC4143993; doi:10.3897/zookeys.428.7366)
Supplement: Supplementary material 10 — Key to Trirhithrum [file zookeys-428-097-s010.zip › SF10_ZooKeys_key to Trirhithrum/key/SF10_key to Trirhithrum/Media/Html/Trirhithrum micans.htm]

Trirhithrum micans Munro


***Trirhithrum micans*** **Munro**

*Trirhithrum micans* Munro, 1957: 872

 

Wing length=4.5-5.1 mm.

Male

Head: Arista long plumose. Two pairs frontal setae. Face white.

Thorax: Postpronotal lobe pale with a dark central mark. Scutum
without silvery-white microtrichose areas. Scutellum disk dark; margin with
baso-lateral pale spots; spots adjacent to bases of apical setae. Anepisternum
largely dark; dorsal edge narrowly pale; one seta. Anatergite (best viewed from
behind) with a bright silvery spot.

Wing: Pattern distinct. Subbasal and discal crossbands fused
posterior to Rs and cell c extensively hyaline; discal crossband distally
aligned with a point near apex of pterostigma and R-M crossvein within discal
crossband. Subapical crossband not joined to discal crossband. Posterior apical
crossband reduced to a short spur. Anal lobe partly coloured, with a hyaline
indentation (ending before vein A1+Cu2) and broadly
hyaline margin. No bulla. Legs: Femora dark.

Abdomen: With distinct grey microtrichose stripes.

 

Female

Terminalia: Aculeus pointed (not dissected but apex exposed in
paratype).

 

(description after White et al., 2003)
