# Supplementary material for: A set of multi-entry identification keys to African frugivorous flies (Diptera, Tephritidae)
Source: Zookeys. 2014 Jul 24;(428):97–108. doi: 10.3897/zookeys.428.7366 (PMC4143993; doi:10.3897/zookeys.428.7366)
Supplement: Supplementary material 10 — Key to Trirhithrum [file zookeys-428-097-s010.zip › SF10_ZooKeys_key to Trirhithrum/key/SF10_key to Trirhithrum/Media/Html/Trirhithrum nigerrimum.htm]

Trirhithrum nigerrimum (Bezzi)


***Trirhithrum nigerrimum*** **(Bezzi)**

*Ceratitis nigra* var. *nigerrima* Bezzi, 1913: 26
(partim, *vide* *T. coffeae*)

 

Female: Wing length=2.8-3.6 mm; Aculeus length=0.62-0.78 mm. Male:
Wing length=2.4-3.4 mm.

Male

Head: Arista long plumose. Two pairs frontal setae. Face white, at
least in lower half.

Thorax: Postpronotal lobe entirely dark. Scutum without
silvery-white microtrichose areas. Scutellum disk dark; margin with
baso-lateral pale spots; spots adjacent to bases of apical setae. Anepisternum
entirely dark; with 1 seta. Anatergite lacking a bright silvery spot.

Wing: Pattern diffuse, especially in costal region; banding
pattern not distinct. Cell c largely dark, at most with a small hyaline spot.
Without a distinct dark mark on C at/before end of Sc and without a
contrastingly dark area near base of cells dm or cu1; pterostigma
markedly darker than rest of pattern. Anal lobe variable from almost entirely
hyaline to almost entirely dark. No bulla.

Legs: Femora dark.

Abdomen: With distinct grey microtrichose stripes or pattern on
terga II, III and IV.

Female

Head, thorax, legs and abdomen mostly as male; postpronotal lobe
sometimes narrowly pale around margin. Wing pattern distinct. Subbasal and
discal crossbands fused posterior to Rs and cell c extensively dark (but
considerably less so than male); no distinct dark mark on C at/before end of
Sc; pterostigma markedly darker than rest of pattern; cell c largely dark; if
extensively hyaline, then with central dark spot and basal and apical dark
areas more or less connected by a diffusely darkened area; basal area of cell r1
immediately above vein R2+3/R4+5 bifurcation with a dark
spot that is broadly connected to large dark area of cell r1. Discal
crossband distally aligned with a point within of pterostigma and R-M crossvein
within discal crossband. Subapical crossband joined to discal crossband; base
narrow, largely or entirely confined to cell r4+5. Posterior apical
crossband reduced to a short spur. Anal lobe coloured but with a hyaline
indentation (ending before vein A1+Cu2). No bulla.
Terminalia with aculeus short, stout and pointed (appears asymmetric under a
coverslip; dorsal view apparently similar to *T. leonense*); spermatheca
curved and bulbous (similar to *T. occipitale*).

 

(description after White et al., 2003)
