# Supplementary material for: A set of multi-entry identification keys to African frugivorous flies (Diptera, Tephritidae)
Source: Zookeys. 2014 Jul 24;(428):97–108. doi: 10.3897/zookeys.428.7366 (PMC4143993; doi:10.3897/zookeys.428.7366)
Supplement: Supplementary material 10 — Key to Trirhithrum [file zookeys-428-097-s010.zip › SF10_ZooKeys_key to Trirhithrum/key/SF10_key to Trirhithrum/Media/Html/Trirhithrum nigrum.htm]

Trirhithrum nigrum (Graham)


***Trirhithrum nigrum*** **(Graham)**

*Ceratitis nigra* Graham, 1910: 162

 

Wing
length=4.2-5.6 mm; Aculeus length=1.1 mm.

Male

Head: Arista plumose. Two pairs frontal setae (most males have a
third seta on left only). Face dark.

Thorax: Postpronotal lobe entirely dark or pale around margin
leaving a dark central mark. Scutum without silvery-white microtrichose areas.
Scutellum disk dark; margin without baso-lateral pale spots; no spots adjacent
to bases of apical setae. Anepisternum entirely dark; one seta. Anatergite
without a bright silvery spot.

Wing: Pattern distinct. Subbasal and discal crossbands fused
throughout; cell c largely to entirely dark. Discal crossband distally aligned
to a point near base of pterostigma; R-M crossvein well distal to edge of
discal crossband. Subapical crossband joined to discal crossband; base deep,
partly in cell dm. Posterior apical crossband reduced to a short spur. Anal
lobe largely to entirely dark. No bulla.

Legs: Femora dark.

Abdomen: With distinct grey/silvery microtrichose spots on tergite
IV tending to form a band.

 

Female

Terminalia: Aculeus fairly short and pointed; spermatheca curved
and apically long bulbous.

 

(description after White et al., 2003)
