# Supplementary material for: A set of multi-entry identification keys to African frugivorous flies (Diptera, Tephritidae)
Source: Zookeys. 2014 Jul 24;(428):97–108. doi: 10.3897/zookeys.428.7366 (PMC4143993; doi:10.3897/zookeys.428.7366)
Supplement: Supplementary material 10 — Key to Trirhithrum [file zookeys-428-097-s010.zip › SF10_ZooKeys_key to Trirhithrum/key/SF10_key to Trirhithrum/Media/Html/Trirhithrum nitidum.htm]

Trirhithrum nitidum (Röder)


***Trirhithrum nitidum*** **(Röder)**

*Ceratitis nitida* Röder, 1885: 134

 

Wing
length=4.7-7.2 mm; Aculeus length=2.40-3.44 mm.

Male

Head: Arista plumose. Three pairs frontal setae (one specimen
[SANC] has been examined with two pairs). Face pale.

Thorax: Postpronotal lobe entirely dark or narrowly pale around
margin, leaving a dark central mark. Scutum without silvery-white microtrichose
areas. Scutellum disk entirely dark in small specimens (wing length under 5.0
mm). Scutellum of larger specimens white in basal two-thirds; margin dark, at
most with pale marks next to setae, but not completely divided into separate
dark areas. Anepisternum entirely dark; two setae. Anatergite without a bright
silvery spot.

Wing: Pattern distinct. Subbasal and discal crossbands fused
posterior to Rs and cell c extensively hyaline; discal crossband distally
aligned with a point within pterostigma and R-M crossvein aligned with edge of
discal crossband. Subapical crossband joined to discal crossband; base deep,
partly in cell dm. Posterior apical crossband narrow but complete, extending
from vein C to wing margin. Anal lobe largely to entirely dark. No bulla.

Legs: Femora fulvous (not distinctly dark or pale).

Abdomen: With distinct grey microtrichose band on tergite IV.

 

Female

Terminalia: Aculeus very long and blunt pointed, similar to *T.
demeyeri*; spermatheca short and apically bulbous.

 

(description after White et al., 2003)
