# Supplementary material for: A set of multi-entry identification keys to African frugivorous flies (Diptera, Tephritidae)
Source: Zookeys. 2014 Jul 24;(428):97–108. doi: 10.3897/zookeys.428.7366 (PMC4143993; doi:10.3897/zookeys.428.7366)
Supplement: Supplementary material 10 — Key to Trirhithrum [file zookeys-428-097-s010.zip › SF10_ZooKeys_key to Trirhithrum/key/SF10_key to Trirhithrum/Media/Html/Trirhithrum notandum.htm]

Trirhithrum notandum Munro


***Trirhithrum notandum*** **Munro**

*Trirhithrum notandum* Munro, 1957: 873

 

Wing length=3.4-3.7 mm; Aculeus length=0.70 mm.

Male

Head: Arista long plumose. Two pairs frontal setae. Face white.

Thorax: Postpronotal lobe pale, with a dark central spot. Scutum
with a thin silvery microtrichose covering posterior to suture. Scutellum disk
dark; margin with baso-lateral pale areas (normally a pair of spots); spots
adjacent to bases of apical setae. Anepisternum largely dark; at most with a
trace of a narrow pale dorsal margin; 1 seta. Anatergite without a bright
silvery spot.

Wing: Pattern diffuse, especially in costal region; banding
pattern not distinct. Without a distinct dark mark on C at/before end of Sc;
pterostigma not markedly darker than rest of pattern. Anal lobe largely
coloured but with a hyaline indentation. No bulla.

Legs: Femora dark.

Abdomen: With distinct grey microtrichose stripes.

 

Female

Head, thorax, legs and abdomen same as male except as follows:
Postpronotal lobe sometimes entirely dark. Scutum without any silvery
microtrichiae. Wing pattern distinct. Subbasal and discal crossbands not
separated posterior to Rs and cell c extensively hyaline; a distinct dark mark
on C at/before end of Sc; pterostigma not markedly darker than rest of pattern;
anterior apical band sometimes broken near end of R2+3; discal
crossband distally aligned with a point within pterostigma and R-M crossvein
within discal crossband. Subapical crossband not joined to discal crossband
(sometimes closely approximated). Posterior apical crossband reduced to a short
spur. Anal lobe coloured but with a hyaline indentation that extends forward
through cell cu1 (sometimes into cell dm). No bulla. Terminalia with
aculeus short and gradually drawn to a point; spermatheca curved and bulbous.

(description after White et al., 2003)
