# Supplementary material for: A set of multi-entry identification keys to African frugivorous flies (Diptera, Tephritidae)
Source: Zookeys. 2014 Jul 24;(428):97–108. doi: 10.3897/zookeys.428.7366 (PMC4143993; doi:10.3897/zookeys.428.7366)
Supplement: Supplementary material 10 — Key to Trirhithrum [file zookeys-428-097-s010.zip › SF10_ZooKeys_key to Trirhithrum/key/SF10_key to Trirhithrum/Media/Html/Trirhithrum obscurum.htm]

Trirhithrum obscurum (Enderlein)


***Trirhithrum obscurum*** **(Enderlein)**

*Ceratitis obscura* Enderlein, 1911: 411.

Wing
length= 5.0-5.7 mm; Aculeus length=1.80 mm.

Male

Head: Arista plumose. Two pairs frontal setae. Face pale in lower
half (some specimens appear dark due to discolouration).

Thorax: Postpronotal lobe entirely dark or narrowly pale around
margin leaving a dark central mark. Scutum without silvery-white microtrichose
areas. Scutellum disk dark; margin with baso-lateral pale spots (two spots or
coalesced into a streak); no spots adjacent to bases of apical setae.
Anepisternum entirely dark; one seta. Anatergite without a bright silvery spot.

Wing: Pattern distinct. Subbasal and discal crossbands fused
posterior to Rs and cell c extensively hyaline; discal crossband distally
aligned with a point near apex of pterostigma and R-M crossvein within or only
slightly beyond discal crossband. Subapical crossband joined to discal
crossband; base deep, partly in cell dm. Posterior apical crossband reduced to
a short spur. Anal lobe entirely dark. No bulla.

Legs: Femora dark.

Abdomen: With distinct grey/silvery microtrichose band on tergite
IV.

 

Female

Terminalia: Aculeus faily long and pointed, and slightly curved
down in lateral view (appears asymmetric under a coverslip; spermatheca sinuate.

(description after White et al., 2003)
