# Supplementary material for: A set of multi-entry identification keys to African frugivorous flies (Diptera, Tephritidae)
Source: Zookeys. 2014 Jul 24;(428):97–108. doi: 10.3897/zookeys.428.7366 (PMC4143993; doi:10.3897/zookeys.428.7366)
Supplement: Supplementary material 10 — Key to Trirhithrum [file zookeys-428-097-s010.zip › SF10_ZooKeys_key to Trirhithrum/key/SF10_key to Trirhithrum/Media/Html/Trirhithrum occipitale.htm]

Trirhithrum occipitale Bezzi 


***Trirhithrum occipitale*** **Bezzi**

*Trirhithrum occipitale* Bezzi, 1918: 239

 

Wing
length=3.2-4.5 mm; Aculeus length=0.96 mm.

Male

Head: Arista long plumose. Two pairs frontal setae. Face white.

Thorax: Postpronotal lobe entirely dark or with a pale margin leaving
a dark central spot. Scutum without silvery-white microtrichose areas.
Scutellum disk dark; margin often with baso-lateral pale spots; often with
spots adjacent to bases of apical setae. Anepisternum entirely dark; one seta.
Anatergite without a bright silvery spot.

Wing: Pattern distinct. Subbasal and discal crossbands fused
posterior to Rs; cell c with basal and apical dark areas well separated from a
central spot; basal area of cell r1 immediately above vein R2+3/R4+5
bifurcation with a dark spot that is at most narrowly connected to large dark
area of cell r1; pterostigma not markedly darker than rest of
pattern; discal crossband distally aligned with a point near apex of
pterostigma and R-M crossvein within discal crossband. Subapical crossband joined
to discal crossband; base narrow, largely or entirely confined to cell r4+5.
Posterior apical crossband reduced to a short spur but sometimes with a trace
of colour reaching M. Anal lobe coloured but with a hyaline indentation (ending
before vein A1+Cu2). No bulla.

Legs: Femora dark.

Abdomen: With distinct grey microtrichose spots on terga II, III
and IV, which may be coalesced into stripes.

 

Female

Terminalia. Aculeus short, stout and pointed (does not appear
asymmetric under a coverslip indicating that it is dorso-ventrally flattened);
spermatheca slightly curved, and bulbous.

 

(description after White et al., 2003)
