# Supplementary material for: A set of multi-entry identification keys to African frugivorous flies (Diptera, Tephritidae)
Source: Zookeys. 2014 Jul 24;(428):97–108. doi: 10.3897/zookeys.428.7366 (PMC4143993; doi:10.3897/zookeys.428.7366)
Supplement: Supplementary material 10 — Key to Trirhithrum [file zookeys-428-097-s010.zip › SF10_ZooKeys_key to Trirhithrum/key/SF10_key to Trirhithrum/Media/Html/Trirhithrum ochriceps.htm]

Trirhithrum ochriceps (Enderlein)


***Trirhithrum ochriceps*** **(Enderlein)**

*Ceratitis ochriceps* Enderlein, 1920: 347

 

Wing length=5.6 mm.

Male

Head: Arista long pubescent. Two pairs frontal setae. Face white
(appearance of a carina assumed to be distortion).

Thorax: Postpronotal lobe more or less paler than scutum. Scutum
without silvery-white microtrichose areas. Scutellum disk dark; margin without
baso-lateral pale spots; no spots adjacent to bases of apical setae.
Anepisternum entirely dark; two setae. Anatergite without a bright silvery spot.

Wing: Pattern distinct. Subbasal and discal crossbands not well
separated posterior to Rs and cell c extensively hyaline; discal crossband
distally aligned with a point near apex of pterostigma and R-M crossvein
slightly beyond discal crossband. Subapical crossband joined to discal
crossband; base deep, partly in cell dm. Posterior apical crossband broad and
complete, extending from vein C to wing margin. Anal lobe largely dark with an
ill defined hyaline indent. No bulla.

Legs: Femora dark.

Abdomen: Without distinct grey/silvery microtrichose spots or
bands.

 

Female

Unknown.

 

(description after White et al., 2003)
