# Supplementary material for: A set of multi-entry identification keys to African frugivorous flies (Diptera, Tephritidae)
Source: Zookeys. 2014 Jul 24;(428):97–108. doi: 10.3897/zookeys.428.7366 (PMC4143993; doi:10.3897/zookeys.428.7366)
Supplement: Supplementary material 10 — Key to Trirhithrum [file zookeys-428-097-s010.zip › SF10_ZooKeys_key to Trirhithrum/key/SF10_key to Trirhithrum/Media/Html/Trirhithrum overlaeti.htm]

Trirhithrum overlaeti Munro


***Trirhithrum overlaeti*** **Munro**

[*Ceratitis*] *Trirhithrum overlaeti* Munro, 1934: 477

 

Wing
length=4.2-5.1 mm; Aculeus length=1.00 mm.

Male

Head: Arista plumose. Two pairs frontal setae. Face white or pale
yellow (holotype somewhat carinate due to distortion).

Thorax: Postpronotal lobe entirely dark. Scutum without
silvery-white microtrichose areas. Scutellum disk entirely white; apical dark
area partly divided by pale marks, or divided by complete lines. Anepisternum
dorsal half pale; with one seta. Anatergite without a bright silvery spot.

Wing: Pattern distinct. Subbasal and discal crossbands clearly
separated anterior to anal lobe and cell c extensively hyaline; discal
crossband distally aligned with a point beyond pterostigma and R-M crossvein
within discal crossband. Subapical crossband not joined to discal crossband.
Posterior apical crossband represented as a spur off the costal band; often
also indicated as at least a trace of colour between M and the wing margin, in
which case it is not connected to the spur off the costal band. Anal lobe
largely to entirely dark (sometimes with diffused paler areas). No bulla.

Legs: Femora dark.

Abdomen: With a distinct grey/silvery microtrichose bands on
tergite IV.

 

Female

Terminalia: Aculeus short, stout and pointed (does not appear
asymmetric under a coverslip indicating that it is dorso-ventrally flattened);
spermatheca recurved in basal part, and apically curved.

 

(description after White et al., 2003)
