# Supplementary material for: A set of multi-entry identification keys to African frugivorous flies (Diptera, Tephritidae)
Source: Zookeys. 2014 Jul 24;(428):97–108. doi: 10.3897/zookeys.428.7366 (PMC4143993; doi:10.3897/zookeys.428.7366)
Supplement: Supplementary material 10 — Key to Trirhithrum [file zookeys-428-097-s010.zip › SF10_ZooKeys_key to Trirhithrum/key/SF10_key to Trirhithrum/Media/Html/Trirhithrum psychotriae.htm]

Trirhithrum psychotriae White


***Trirhithrum psychotriae*** **White**

*Trirhithrum psychotriae* White, 2003: 114.

 

 

Wing
length=3.1-3.6 mm; Aculeus length=0.74 mm.

Male

Head: Arista long plumose. Two pairs frontal setae. Face mostly
white.

Thorax: Postpronotal lobe with a narrow pale margin. Scutum
without silvery-white microtrichose areas. Scutellum disk dark; margin with
baso-lateral pale areas (normally a streak); spots adjacent to bases of apical
setae. Anepisternum largely dark except for a very narrow pale line across
dorsal margin; with one seta. Anatergite without a bright silvery spot.

Wing: Pattern diffuse, especially in costal region; banding
pattern not distinct. Cell c largely hyaline. With a distinct dark mark on C
at/before end of Sc and with a contrastingly dark area near base of cell dm;
pterostigma not markedly darker than rest of pattern. Anal lobe largely hyaline
or coloured but with a hyaline indentation. No bulla.

Legs: Femora dark.

Abdomen: With distinct grey microtrichose stripes.

 

Female

Head, thorax, legs and abdomen as in male. Wing pattern distinct.
Subbasal and discal crossbands not fully separated posterior to Rs and cell c
extensively hyaline; cell bc with dark area extended well into basal half of
cell; a distinct dark mark on C at/before end of Sc; pterostigma not markedly
darker than rest of pattern; discal crossband distally aligned with a point
within pterostigma and R-M crossvein within discal crossband; often somewhat
darkened in cell dm. Subapical crossband joined to discal crossband; base
narrow, usually largely or entirely confined to cell r4+5. Posterior
apical crossband reduced to a short spur. Anal lobe coloured but with a hyaline
indentation (ending before or only slightly anterior to vein A1+Cu2).
No bulla. Terminalia with aculeus short, stout and pointed (appears asymmetric
under a coverslip; dorsal view apparently similar to *T. leonense*);
spermatheca curved and bulbous (similar to *T. occipitale*).

 

(description after White et al., 2003)
