# Supplementary material for: A set of multi-entry identification keys to African frugivorous flies (Diptera, Tephritidae)
Source: Zookeys. 2014 Jul 24;(428):97–108. doi: 10.3897/zookeys.428.7366 (PMC4143993; doi:10.3897/zookeys.428.7366)
Supplement: Supplementary material 10 — Key to Trirhithrum [file zookeys-428-097-s010.zip › SF10_ZooKeys_key to Trirhithrum/key/SF10_key to Trirhithrum/Media/Html/Trirhithrum quadrimaculatum.htm]

Trirhithrum quadrimaculatum White


***Trirhithrum quadrimaculatum*** **White**

*Trirhithrum quadrimaculatum* White, 2003: 115.

 

Wing
length=3.9-4.5 mm; Aculeus length=1.08 mm.

Male

Head: Arista long pubescent to plumose. Two pairs frontal setae.
Face entirely white.

Thorax: Postpronotal lobe pale either along lateral margin, or
around entire margin. Scutum without silvery-white microtrichose areas.
Scutellum disk dark; margin with baso-lateral pale spots; spots adjacent to
bases of apical setae. Anepisternum largely dark; dorsal edge narrowly pale; one
seta. Anatergite without a bright silvery spot. Katatergite with a small pale
spot.

Wing: Pattern distinct. Subbasal and discal crossbands fused
posteriorly and cell c extensively hyaline; cell bc with dark area not extended
into basal half of cell. Discal crossband distally aligned with a point within
pterostigma and R-M crossvein within discal crossband. Subapical crossband
joined to discal crossband; base narrow, largely or entirely confined to cell r4+5.
Posterior apical crossband present to beyond vein M but not reaching wing
margin. Anal lobe coloured but with a hyaline indentation (ending before vein A1+Cu2).
No bulla. Legs: Femora dark.

Abdomen: With distinct grey microtrichose spots which tend to
coalesce into stripes.

 

Female

Terminalia: Aculeus short, stout and apically pointed (does not
appear asymmetric under a coverslip indicating that it is dorso-ventrally
flattened); spermatheca apically bulbous.

 

 

(description after White et al., 2003)
