# Supplementary material for: A set of multi-entry identification keys to African frugivorous flies (Diptera, Tephritidae)
Source: Zookeys. 2014 Jul 24;(428):97–108. doi: 10.3897/zookeys.428.7366 (PMC4143993; doi:10.3897/zookeys.428.7366)
Supplement: Supplementary material 10 — Key to Trirhithrum [file zookeys-428-097-s010.zip › SF10_ZooKeys_key to Trirhithrum/key/SF10_key to Trirhithrum/Media/Html/Trirhithrum resplendens.htm]

Trirhithrum resplendens Hancock


***Trirhithrum resplendens*** **Hancock**

*Trirhithrum resplendens* Hancock, 1984: 298

 

Wing length=4.0-5.0 mm.

Male

Head: Arista micropubescent. Two pairs frontal setae. Face white.

Thorax: Postpronotal lobe entirely dark. Scutum without
silvery-white microtrichose areas. Scutellum disk dark; margin without
baso-lateral pale spots; no spots adjacent to bases of apical setae.
Anepisternum pale in dorsal third; pale area narrowing anteriorly; one seta.
Anatergite without a bright silvery spot.

Wing: Pattern distinct. Subbasal and discal crossbands fused
posterior to Rs and cell c extensively hyaline; discal crossband distally
aligned with apex of pterostigma; R-M crossvein usually well distal to edge of
discal crossband. Subapical crossband joined to discal crossband; base deep,
partly in cell dm. Posterior apical crossband complete, extending from vein C
to wing margin. Anal lobe largely hyaline. No bulla.

Legs: Femora dark.

Abdomen: With distinct grey/silvery microtrichose bands on terga
II and IV.

Female

Unknown.

 

(description after White et al., 2003)
