# Supplementary material for: A set of multi-entry identification keys to African frugivorous flies (Diptera, Tephritidae)
Source: Zookeys. 2014 Jul 24;(428):97–108. doi: 10.3897/zookeys.428.7366 (PMC4143993; doi:10.3897/zookeys.428.7366)
Supplement: Supplementary material 10 — Key to Trirhithrum [file zookeys-428-097-s010.zip › SF10_ZooKeys_key to Trirhithrum/key/SF10_key to Trirhithrum/Media/Html/Trirhithrum scintillans.htm]

Trirhithrum scintillans Munro


***Trirhithrum scintillans*** **Munro**

[*Ceratitis*] *Trirhithrum scintillans* Munro, 1934: 483

 

Wing length=3.7-4.3 mm.

Male and female Identical to *T. occipitale* except as
follows: Apical half of cell c with a small central mark, and large basal and
apical dark markings (apical and central marks approximated); base of subapical
crossband deep, partly in cell dm; abdomen without any spots or bands of
microtrichia (the specimens show no signs of being greasy or rubbed, and so
this is assumed to be a genuine absence rather than an artefact caused by poor
preparation or preservation). Female terminalia with aculeus pointed (not
dissected but apex exposed in paralectotype).

 

(description after White et al., 2003)
