# Supplementary material for: A set of multi-entry identification keys to African frugivorous flies (Diptera, Tephritidae)
Source: Zookeys. 2014 Jul 24;(428):97–108. doi: 10.3897/zookeys.428.7366 (PMC4143993; doi:10.3897/zookeys.428.7366)
Supplement: Supplementary material 10 — Key to Trirhithrum [file zookeys-428-097-s010.zip › SF10_ZooKeys_key to Trirhithrum/key/SF10_key to Trirhithrum/Media/Html/Trirhithrum senex.htm]

Trirhithrum senex Munro


***Trirhithrum senex*** **Munro**

*Trirhithrum senex* Munro, 1938: 167

 

Measurements (Female): Wing length=2.8-3.5 mm; Aculeus length=0.78
mm; (Male): Wing length=2.7-3.3 mm.

Male

Head: Arista long plumose. Two pairs frontal setae. Face dark or
indistinctly pale in lower half.

Thorax: Postpronotal lobe pale with a dark central mark. Scutum
with distinct silvery-white microtrichose covering anterior to dorso-central
setae but area not inverted V-shaped. Scutellum disk dark; margin with
baso-lateral pale areas (pair of spots or coalesced into a streak); no spots
adjacent to bases of apical setae. Anepisternum largely fulvous, with a narrow
pale yellow dorsal margin; one seta. Anatergite (best viewed from behind) often
with a bright silvery spot.

Wing: Pattern distinct. Subbasal and discal crossbands clearly
separated and cell c extensively hyaline; short discal crossband distally
aligned with a point within pterostigma. Subapical crossband joined to discal
crossband; base narrow, largely or entirely confined to cell r4+5.
Posterior apical crossband extending to beyond vein M but not always reaching
wing margin (reaches in holotype). Anal lobe hyaline. An isolated dark round
spot at end of vein A1+Cu2 (bulla).

Legs: Femora pale.

Abdomen: A deep microtrichose band on tergite II and large,
usually ill-defined, spots on terga III and IV.

Female

As in male, except: face pale; microtrichose area of prescutum
present but indistinct; posterior apical crossband never extended to wing
margin and sometimes ending before vein M (but closer to M than R4+5);
wing without a bulla; femora dark; abdominal microtrichia pattern well defined.
Terminalia with aculeus short, stout and pointed (appears asymmetric under a
coverslip; dorsal view apparently similar to *T. meladiscum*; spermatheca
bulbous.

 

(description after White et al., 2003)
