# Supplementary material for: A set of multi-entry identification keys to African frugivorous flies (Diptera, Tephritidae)
Source: Zookeys. 2014 Jul 24;(428):97–108. doi: 10.3897/zookeys.428.7366 (PMC4143993; doi:10.3897/zookeys.428.7366)
Supplement: Supplementary material 10 — Key to Trirhithrum [file zookeys-428-097-s010.zip › SF10_ZooKeys_key to Trirhithrum/key/SF10_key to Trirhithrum/Media/Html/Trirhithrum stecki.htm]

Trirhithrum stecki White


***Trirhithrum stecki*****White**

*Trirhithrum stecki*
White, 2003: 119.

 

Wing
length=4.6-7.4 mm; Aculeus length=2.02 mm.

Male

Head: Arista long pubescent to plumose. Two pairs frontal setae.
Face pale.

Thorax: Postpronotal lobe entirely dark. Scutum without silvery-white
microtrichose areas. Scutellum disc white; margin dark between setae.
Anepisternum with a diagonal white stripe covering posterior half; one seta.
Anatergite without a bright silvery spot.

Wing: Pattern distinct. Subbasal and discal crossbands fused
posteriorly and cell c extensively hyaline; discal crossband distally aligned
with a point within pterostigma and R-M crossvein aligned to edge of discal
crossband. Subapical crossband joined to discal crossband; base deep, partly in
cell dm. Posterior apical crossband represented by both a spur off the costal
band and by a separate (isolated) diagonal line which crosses vein M and more
or less reaches the wing margin. Anal lobe largely dark, at most tending to
hyaline along wing margin. No bulla.

Legs: Femora dark.

Abdomen: With grey/silvery microtrichose bands (not very
distinct).

 

Female

Terminalia: Aculeus fairly long and pointed; spermatheca large,
curved and slightly bulbous at apex (similar to *T. validum*).

 

(description after White et al., 2003)
