# Supplementary material for: A set of multi-entry identification keys to African frugivorous flies (Diptera, Tephritidae)
Source: Zookeys. 2014 Jul 24;(428):97–108. doi: 10.3897/zookeys.428.7366 (PMC4143993; doi:10.3897/zookeys.428.7366)
Supplement: Supplementary material 10 — Key to Trirhithrum [file zookeys-428-097-s010.zip › SF10_ZooKeys_key to Trirhithrum/key/SF10_key to Trirhithrum/Media/Html/Trirhithrum stubbsi.htm]

Trirhithrum stubbsi White & Hancock


***Trirhithrum*** ***stubbsi* White & Hancock**

*Trirhithrum* *stubbsi* White & Hancock, 2003: 119.

 

Wing
length=3.6-3.8 mm; Aculeus length=0.60 mm.

Male

Head: Arista long pubescent to plumose. Two pairs frontal setae.
Face white in lower half.

Thorax: Postpronotal lobe largely dark, pale laterally. Scutum
without silvery-white microtrichose areas. Scutellum disk dark; margin with
paired baso-lateral pale spots; no spots adjacent to bases of apical setae.
Anepisternum largely dark, with a fairly broad pale dorsal margin; one seta.
Anatergite without a bright silvery spot.

Wing: Pattern distinct. Subbasal and discal crossbands fused
posteriorly and cell c extensively hyaline; discal crossband distally aligned
with a point within pterostigma and R-M crossvein within discal crossband.
Subapical crossband not joined to discal crossband. Posterior apical crossband
reduced to a short spur. Anal lobe coloured but with a hyaline indentation
(ending before vein A1+Cu2). No bulla.

Legs: Femora dark.

Abdomen: With distinct grey microtrichose spots (almost coalesced
into a band) on tergite IV.

 

Female

Terminalia: Aculeus short, stout and apically pointed (does not
appear asymmetric under a coverslip indicating that it is dorso-ventrally
flattened; spermatheca apically bulbous and slightly curved.

 

(description after White et al., 2003)
