# Supplementary material for: A set of multi-entry identification keys to African frugivorous flies (Diptera, Tephritidae)
Source: Zookeys. 2014 Jul 24;(428):97–108. doi: 10.3897/zookeys.428.7366 (PMC4143993; doi:10.3897/zookeys.428.7366)
Supplement: Supplementary material 10 — Key to Trirhithrum [file zookeys-428-097-s010.zip › SF10_ZooKeys_key to Trirhithrum/key/SF10_key to Trirhithrum/Media/Html/Trirhithrum teres.htm]

Trirhithrum teres Munro


***Trirhithrum teres*** **Munro**

*Trirhithrum teres* Munro, 1938: 165

 

Wing
length=3.1-3.5 mm; Aculeus length=0.76 mm.

Male

Head: Arista plumose. Two pairs frontal setae. Face dark except
for white band across centre and a second white band across the dorsal margin.

Thorax: Postpronotal lobe pale with a dark central mark. Scutum
with a reticulate pattern of sparce microtrichia. Scutellum disk dark, except
for a pair of baso-medial pale spots; margin with baso-lateral pale areas (two
spots or coalesced into a streak); no spots adjacent base of apical seta.
Anepisternum largely dark; dorsal edge narrowly pale; one seta. Anatergite with
a bright silvery spot.

Wing: Pattern distinct. Subbasal and discal crossbands more or
less fused posterior to Rs; cell c extensively hyaline; discal crossband
distally aligned with a point within pterostigma and R-M crossvein aligned to
edge of discal crossband. Subapical crossband joined to discal crossband; base
deep, partly in cell dm. Posterior apical crossband reduced to a short spur.
Anal lobe coloured but with a hyaline indentation (ending before vein A1+Cu2).
No bulla.

Legs: Femora dark.

Abdomen: With silvery microtrichose bands on terga II and IV.

 

Female

Aculeus short, stout and apically pointed (similar to *T. basale*);
spermatheca apically bulbous and slightly curved (similar to *T. occipitale*).

(description after White et al., 2003)
