# Supplementary material for: A set of multi-entry identification keys to African frugivorous flies (Diptera, Tephritidae)
Source: Zookeys. 2014 Jul 24;(428):97–108. doi: 10.3897/zookeys.428.7366 (PMC4143993; doi:10.3897/zookeys.428.7366)
Supplement: Supplementary material 10 — Key to Trirhithrum [file zookeys-428-097-s010.zip › SF10_ZooKeys_key to Trirhithrum/key/SF10_key to Trirhithrum/Media/Html/Trirhithrum transiens.htm]

Trirhithrum transiens Munro


***Trirhithrum transiens*** **Munro**

*Trirhithrum transiens* Munro, 1957: 874

 

(Female): Wing length=3.8 mm; (Male): Wing length=3.4-3.8 mm.

Male

Head: Arista long pubescent. Two pairs frontal setae. Face dark.

Thorax: Postpronotal lobe pale laterally, or marginally leaving a
dark central mark. Scutum with an indistinct reticulate pattern of
microtrichiae. Scutellum disk dark; margin with baso-lateral pale streak; no
spots adjacent to bases of apical setae. Anepisternum entirely fulvous to dull
grey; one seta. Anatergite (best viewed from behind) often with a bright
silvery spot.

Wing: Pattern distinct. Subbasal and discal crossbands clearly
separated and cell c extensively hyaline; discal crossband distally aligned
with a point within pterostigma and R-M crossvein within discal crossband.
Subapical crossband joined to costal band. Posterior apical crossband complete,
extending from vein C to wing margin. Anal lobe crossed by discal crossband and
by a mark extended from the bcu extension. No bulla.

Legs: Femora pale (yellowish).

Abdomen: With grey microtrichose bands.

Female

Same as in male except as follows: anepisternum dark in lower
half, pale in dorsal half; femora dark (dull brown).

 

(description after White et al., 2003)
