# Supplementary material for: A set of multi-entry identification keys to African frugivorous flies (Diptera, Tephritidae)
Source: Zookeys. 2014 Jul 24;(428):97–108. doi: 10.3897/zookeys.428.7366 (PMC4143993; doi:10.3897/zookeys.428.7366)
Supplement: Supplementary material 10 — Key to Trirhithrum [file zookeys-428-097-s010.zip › SF10_ZooKeys_key to Trirhithrum/key/SF10_key to Trirhithrum/Media/Html/Trirhithrum validum.htm]

Trirhithrum validum Bezzi


***Trirhithrum validum*** **Bezzi**

*Trirhithrum validum* Bezzi, 1920: 236

 

Wing
length=(6.6)8.0-8.5 mm; Aculeus length=2.38 mm.

Male

Head: Arista plumose. Two pairs frontal setae. Face pale in at
least lower half.

Thorax: Postpronotal lobe entirely dark. Scutum without
silvery-white microtrichose areas. Scutellum disc white; margin with three dark
marks. Anepisternum with a diagonal white stripe covering posterior half; one
seta. Anatergite without a bright silvery spot.

Wing: Pattern distinct. Subbasal and discal crossbands fused
posteriorly and cell c extensively hyaline; discal crossband distally aligned
with apex of pterostigma and R-M crossvein aligned to edge of discal crossband.
Subapical crossband joined to discal crossband; base deep, partly in cell dm.
Posterior apical crossband narrow but complete, extending from vein C to wing
margin. Anal lobe largely dark, at most tending to hyaline along wing margin.
No bulla. Legs: Femora dark.

Abdomen: With very distinct silvery microtrichose band on tergite
IV and sometimes also II.

 

Female

Terminalia: Aculeus fairly long and pointed (similar to *T.
stecki*); spermatheca large and curved.

 

(description after White et al., 2003)
