# Supplementary material for: A set of multi-entry identification keys to African frugivorous flies (Diptera, Tephritidae)
Source: Zookeys. 2014 Jul 24;(428):97–108. doi: 10.3897/zookeys.428.7366 (PMC4143993; doi:10.3897/zookeys.428.7366)
Supplement: Supplementary material 10 — Key to Trirhithrum [file zookeys-428-097-s010.zip › SF10_ZooKeys_key to Trirhithrum/key/SF10_key to Trirhithrum/Media/Html/Trirhithrum viride.htm]

Revision of the afrotropical genus Trirhithrum Bezzi (Diptera:
Tephritidae, Ceratitidini)


***Trirhithrum viride*** **Munro**

[*Ceratitis*] *Trirhithrum viride* Munro, 1934: 479

 

Wing length=3.4 mm.

Female

All characters identical to, or fitting within the known range of
variation of *T. senex*, except as follows: posterior apical crossband
short, ending closer to vein R4+5 than to vein M. Scutum without a
microtrichose sheen. Female terminalia with aculeus pointed (not dissected but
apex exposed in holotype).

 

Male

Unknown; likely to be similar to female, but with a bulla.

 

(description after White et al., 2003)
